# Catalytic Enantioselective Intramolecular Aza‐Michael Addition to α,β‐Unsaturated Esters

**DOI:** 10.1002/anie.5995443

**Published:** 2026-06-07

**Authors:** Evan G. W. Rutter, Cameron J. MacRae, Haoran Xiong, Daniel Rozsar, Katherine F. P. Clarke, Darren J. Dixon

**Affiliations:** ^1^ Department of Chemistry Chemistry Research Laboratory University of Oxford Oxford UK; ^2^ Graduate School of Pharmaceutical Sciences Tohoku University Sendai Japan

**Keywords:** enantioselective catalysis, enantioselectivity, Michael addition, nitrogen heterocycles, organocatalysis

## Abstract

A bifunctional iminophosphorane (BIMP)‐catalyzed enantioselective intramolecular aza‐Michael reaction of sulfonamides to tethered α,β‐unsaturated esters is described. Reactivity toward traditionally challenging ester Michael acceptors is achieved through careful catalyst design, enabling the synthesis of enantioenriched pyrrolidines, piperidines, and indoline derivatives in excellent yields (up to 99%) and enantiomeric ratios (up to 97.5:2.5 er) under operationally simple conditions. The broad reaction scope demonstrates excellent tolerance to variation in the sulfonamide group, the Michael acceptor, and substituents on the tether in between. Furthermore, the synthetic utility of the method is highlighted by gram‐scale reactions at reduced catalyst loadings and by downstream derivatization of multiple enantiopure products.

## Graphical abstract

A general method for accessing enantioenriched saturated *N*‐heterocycles via a catalytic enantioselective intramolecular aza‐Michael reaction to α,β‐unsaturated esters is described. A superbasic bifunctional iminophosphorane (BIMP) catalyst was key to enabling reactivity of the high *p*K_a_ sulfonamide pronucleophile, whilst delivering good to excellent yields and enantioselectivities of the sulfonyl protected cyclic amine products.

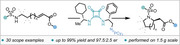

## 1 Introduction

**SCHEME 1:**
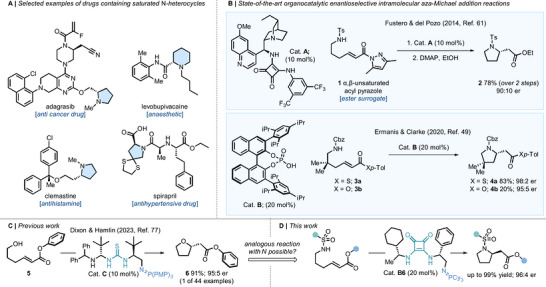
(A) Selected examples of small molecule drugs containing saturated *N*‐heterocycles. (B) State‐of‐the‐art organocatalytic enantioselective intramolecular aza‐Michael reactions and limitations with respect to α,β‐unsaturated ester Michael acceptors. (C) Previous work by our group on catalytic enantioselective intramolecular oxa‐Michael reaction to α,β‐unsaturated esters and amides. (D) This work.

Chiral saturated nitrogen heterocycles are ubiquitous in natural products [1, 2, 3] and pharmaceuticals [4, 5, 6, 7], including the KRAS^G12C^ inhibitor adagrasib [8], the local anaesthetic drug levobupivacaine [9], and the histamine H1 antagonist clemastine [10] (Scheme 1A). A 2024 review reports that 82% of FDA‐approved small molecule drugs from 2013 to 2023 contain a nitrogen heterocycle, with piperidines and pyrrolidines ranking as the second and third most prevalent, respectively [4]. In recent years, spirocyclic pyrrolidines and piperidines, for example, the ACE inhibitor spirapril [10] (Scheme 1A), have received increasing attention in the pharmaceutical sector due to their stereochemically defined three‐dimensional structures, which often exhibit improved binding to biological targets [11, 12, 13, 14]. Consequently, the development of novel catalytic methods for constructing these motifs remains of significant importance [15, 16, 17].

Amongst the most direct strategies for the synthesis of enantioenriched saturated *N*‐heterocycles [18, 19, 20, 21, 22, 23, 24, 25, 26, 27, 28, 29, 30, 31, 32, 33, 34, 35, 36, 37, 38, 39] is the catalytic enantioselective intramolecular aza‐Michael reaction (IMAMR) [17]. Although catalytic enantioselective IMAMR processes involving α,β‐unsaturated aldehydes [40, 41, 42, 43], ketones [44, 45, 46, 47, 48], and thioesters [49, 50, 51] are well developed, the analogous reaction employing synthetically attractive and more readily accessible α,β‐unsaturated esters remains comparatively unexplored [52, 53, 54, 55, 56, 57, 58, 59]. This limitation likely arises from the reduced electrophilicity of the ester‐based Michael acceptors [60].

State‐of‐the‐art approaches (Scheme 1B) include the catalytic enantioselective IMAMR to more activated α,β‐unsaturated pyrazole amides reported by Fustero and del Pozo [61]. However, in order to unmask the corresponding ester product, alcoholysis of the pyrazole amide is required in a subsequent step, resulting in a less atom‐efficient process overall. Additionally, Ermanis and Clarke disclosed a chiral phosphoric acid‐catalysed IMAMR to α,β‐unsaturated thioesters [49, 50, 51], achieving excellent yields and enantioselectivities. In contrast, applying this catalytic system to the corresponding ester Michael acceptors resulted in a dramatic decrease in yield from 83% to 20%, underscoring both the diminished electrophilicity of α,β‐unsaturated esters relative to their aldehyde, ketone, and thioester counterparts, and the ongoing challenge of developing a general catalytic method for this transformation.

Building on recent advances in iminophosphorane catalysis [62, 63, 64, 65, 66, 67, 68, 69, 70, 71, 72, 73, 74, 75, 76], in 2023 our group reported a general method for the catalytic enantioselective intramolecular oxa‐Michael reaction to α,β‐unsaturated esters and amides (Scheme 1C) [77], highlighting the advantages of employing a high‐*p*K_BH+_ bifunctional iminophosphorane (BIMP) catalyst to activate high‐*p*K_a_ pronucleophiles [63]. The reported findings suggested that an analogous aza‐Michael reaction might also be feasible (Scheme 1D). Herein, we describe the catalytic enantioselective IMAMR of suitably protected amine‐derived pronucleophiles using highly basic BIMP catalysts and present our findings on the development of this transformation.

## 2 Results and Discussion

Preliminary studies examining various *N*‐protected amines (*N*‐Boc, *N*‐Ph, *N*‐SO_2_Ph) led us to focus on the cyclization of sulfonamide model substrate **7a** into enantioenriched pyrrolidine **8a**. Initial evaluation of a small set of BIMP catalysts revealed that thiourea‐derived catalyst **B1** promoted the conversion of precursor **7a** to the enantioenriched pyrrolidine **8a** in 66% conversion and 29:71 er (Table 1, entry 1). Modification of the hydrogen bond donor moiety to a squaramide group (catalyst **B2**) afforded similar results (Table 1, entry 2). Improved performance was observed when the phosphine component was replaced with the more electron‐rich tricyclohexylphosphine (catalyst **B3**), delivering **8a** in 91% conversion and 75.5:24.5 er (Table 1, entry 3).

**TABLE 1:** Selected experiments for the optimization of the catalytic enantioselective intramolecular aza‐Michael reaction to α,β‐unsaturated esters.

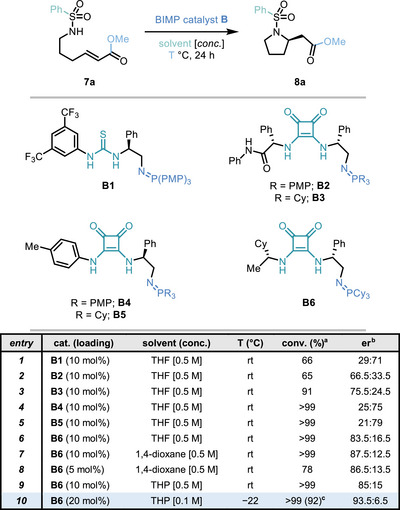

^a^ Determined by ^1^H NMR analysis of the crude reaction mixture.

^b^ Determined by HPLC on a chiral stationary phase.

^c^ Isolated yield.

Squaramide catalysts **B4** and **B5** both achieved full conversion, providing **8a** in 25:75 and 21:79 er, respectively (Table 1, entries 4 and 5), with the superior performance of **B5** attributed to the incorporation of the tricyclohexylphosphine unit. Increasing the steric bulk around the additional stereocentre adjacent to the squaramide (catalyst **B6**) further enhanced the enantioselectivity to 83.5:16.5 er (Table 1, entry 6).

Following systematic optimization of solvent, temperature, concentration and catalyst loading (Table 1, entries 7–10, see Supporting Information for full details of optimisation), we were pleased to find that substrate **7a** underwent full conversion to **8a** with 93.5:6.5 er with catalyst **B6** (20 mol%) when the reaction was conducted in tetrahydropyran (THP) at a concentration of 0.1 M and a temperature of −22°C (Table 1, entry 10).

**SCHEME 2:**
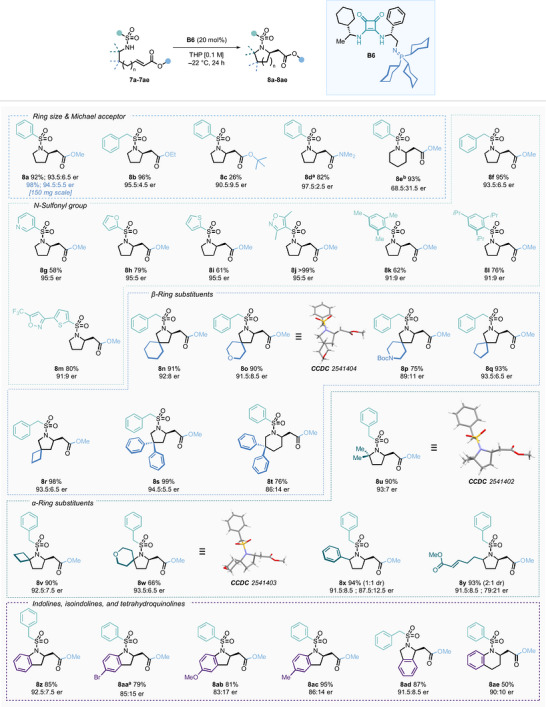
Scope of BIMP catalysed enantioselective intramolecular aza‐Michael addition to *α,β*‐unsaturated esters; reactions were carried out on a 0.1 mmol scale; all yields refer to isolated yields; er were determined by HPLC analysis on a chiral stationary phase; dr were determined by crude ^1^H NMR analysis; variations from standard conditions: ^(a)^7 days; ^(b)^room temperature.

With the optimized conditions in hand, we next explored the scope of the reaction (Scheme 2). We first examined the influence of ring size and the alkoxy substituent of the Michael acceptor. Encouragingly, pyrrolidine precursors **7b** and **7c,** bearing ethyl and *tert*‐butyl ester Michael acceptors, respectively, were converted to pyrrolidines **8b** and **8c** with good levels of enantiocontrol (>90:10 er). Although **8b** was obtained in excellent yield (96%), the more sterically demanding and less electrophilic *tert*‐butyl ester [60] delivered **8c** in a more modest 26% yield.

We were also pleased to find that pyrrolidine precursor **7d**, bearing a tertiary amide Michael acceptor, underwent cyclization to furnish the corresponding enantioenriched pyrrolidine in 82% yield and 97.5:2.5 er. An extended reaction time of 7 days was, however, required to compensate for the reduced reactivity of the unsaturated amide [60].

Changing the ring size from five‐ to six‐membered (**7e**) required an elevated reaction temperature (room temperature) to achieve >90% yield of **8e**; however, this resulted in a notable decrease in enantioselectivity (68.5:31.5 er). Finally, the reaction was readily performed on a 150 mg scale, affording enantioenriched pyrrolidine **8a** in 98% yield with a slight improvement in enantiomeric ratio to 94.5:5.5 er.

Variation of the sulfonyl group was broadly tolerated, with both electron‐rich and electron‐deficient heteroaromatic substituents proving compatible under the reaction conditions. Substrates bearing benzyl, pyridine, furan, thiophene, and isoxazole‐derived sulfonyl groups (**7f‐7j**) underwent smooth cyclization to furnish products **8f‐8j** in moderate to excellent yields (58%–99%) and with consistently high levels of enantiocontrol (>90:10 er). We next examined more sterically demanding aromatic groups. Encouragingly, increasingly hindered aromatic groups were also well tolerated (**7k‐7m**), affording pyrrolidines **8k‐8m** without substantial loss in efficiency or selectivity.

After examining the variation of the Michael acceptor and sulfonyl group, we next explored the effect of substitution along the amine tether. Precursors **7n‐7p**, bearing cyclohexane, tetrahydropyran, and *N*‐Boc piperidine substituents at the β‐position relative to the amine, underwent efficient cyclization to furnish the corresponding spiropyrrolidines **8n‐8p** in good yields (75%–91%). High levels of enantiocontrol were maintained for substrates **8n** and **8o** (>90:10 er), whilst spiropyrrolidine **8p** containing the *N*‐Boc piperidine unit was obtained with a slightly diminished enantiomeric ratio (89:11 er). Single‐crystal x‐ray diffraction (SCXRD) analysis of a recrystallised sample of **8o** established the absolute stereochemical configuration of the major enantiomer as (*R*) [78].

Variation of the ring size at the β‐position was also well tolerated, with five‐ and four‐membered ring containing substrates **7q** and **7r** smoothyl cyclizing to afford spiropyrrolidines **8q** and **8r** in excellent yields and with high levels of enantiocontrol (>90:10 er). Gratifyingly, pyrrolidine and piperidine precursors **7s** and **7t** bearing a bulky *gem*‐diphenyl substituent at the β‐position underwent smooth cyclization to deliver pyrrolidine **8s** in 99% yield and 94.5:5.5 er, and piperidine **8t** in 76% yield and 86:14 er. Notably, enantioenriched piperidine **8t** could also be formed at −22°C in 76% yield, enabling improved enantioselectivity (86:14 er) relative to its unsubstituted analogue **8e**, likely due to a reduced kinetic barrier to cyclization [79].

The effects of introducing substituents in the α‐position relative to the amine were next examined. Pyrrolidine precursor **7u** bearing a *gem*‐dimethyl group underwent smooth conversion to **8u** in 90% yield and 93:7 er. Replacing this substituent with a cyclobutyl group (**7v**) also afforded **8v** in excellent yield (90%) and enantioselectivity (92.5:7.5 er), whilst tetrahydropyran‐substituted precursor **7w** delivered **8w** in good yield (66%), and with good enantiocontrol (93.5:6.5 er). SCXRD analysis of **8u** and **8w** confirmed the absolute stereochemical configuration of both products as (*R*) [78].

Racemic substrate **7x**, bearing a phenyl substituent α to the nitrogen, was converted to **8x** (and epi‐**8x**) in 94% yield as a 1:1 mixture of inseparable diastereomers, each formed with high enantioselectivity. Unfortunately, the difference in reaction rates between the two enantiomers of the starting material was insufficient to enable an effective kinetic resolution of **7x** using this catalyst system. Similarly, desymmetrization precursor **7y** afforded pyrrolidine **8y** (and epi‐**8y**) in 93% yield as a 2:1 mixture of inseparable diastereomers. Although the major diastereomer was formed with good enantiocontrol (91.5:8.5 er), the small difference in reaction rates leading to the two diastereomers precluded the development of an efficient desymmetrization process.

With general reactivity established, we envisioned extending this methodology to a different substrate class incorporating an aromatic ring within the tether, thereby providing access to enantiomerically enriched benzannulated nitrogen heterocycles. Indoline precursor **7z** was converted to **8z** in 85% yield and with good enantiocontrol (92.5:7.5 er). Substituents on the aromatic ring were also well tolerated. Precursors **7aa** (*p*‐Br), **7ab** (*p*‐OMe), and **7ac** (*p*‐Me) furnished products **8aa**‐**8ac** in good yields (79%‐95%). The electron‐deficient *para*‐bromo aniline **7aa** required an extended reaction time of 7 days and delivered slightly lower enantioselectivity than the unsubstituted analogues (>83:17 er).

The reactivity of isoindoline precursor **7ad** was subsequently examined, affording **8ad** in good yield (87%) whilst maintaining high enantioselectivity (91.5:8.5 er). Finally, sulfonyl‐protected aniline **7ae** was converted to tetrahydroquinoline **8ae** in 50% yield ‐ consistent with the lower reactivity observed for other six‐membered ring precursors ‐ whilst delivering a pleasing 90:10 er.

**SCHEME 3:**
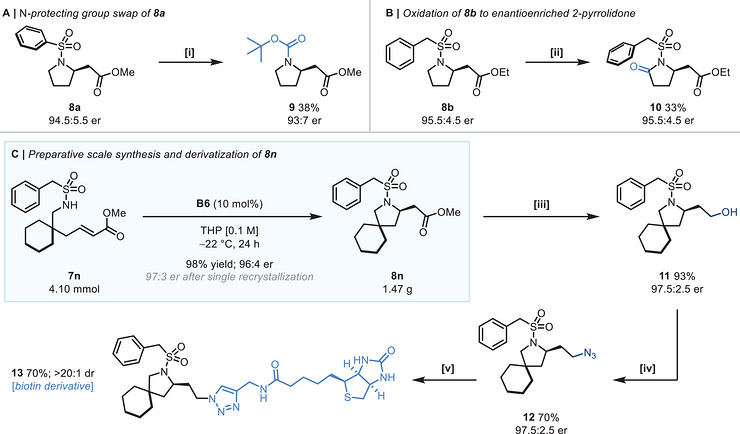
(A) *N*‐protecting group swap of **8a**. (B) Oxidation of **8b** to enantioenriched 2‐pyrrolidone. (C) Preparative scale synthesis and derivatization of **8n**. All yields refer to isolated yields; er were determined by HPLC or SFC analysis on a chiral stationary phase; dr were determined by crude ^1^H NMR analysis. (i) Mg (25 equiv.), MeOH (0.05 M), ultrasound, 1 h; *then* Boc_2_O (1.5 equiv.), NEt_3_ (3 equiv.), DMAP (20 mol%), CH_2_Cl_2_ (0.05 M), 16 h. (ii) ketoABNO (25 mol%), *m*CPBA (4.3 equiv.), MeCN (0.1 M) r.t., 20 h. (iii) LiAlH_4_ (2 equiv.), THF (0.1 M), 0°C, 15 min. (iv) PPh_3_ (1.5 equiv.), DIAD (1.5 equiv.), DPPA (1.5 equiv.), THF (0.1 M), r.t., 17 h. (v) (3a*S*,4*S*,6a*R*)‐Hexahydro‐2‐oxo‐*N*‐2‐propyn‐1‐yl‐1*H*‐thieno[3,4‐*d*]imidazole‐4‐pentanamide (1 equiv.), sodium ascorbate (25 mol%), CuSO_4_.5H_2_O (10 mol%), H_2_O/THF 1/1 (0.175 M), r.t., 20 h.

Having established a broad substrate scope, we next investigated the synthetic utility of the methodology by evaluating its scalability and derivatization of enantioenriched products. Removal of the sulfonyl group in pyrrolidine **8a** was accomplished by sonication with magnesium in methanol. The resulting intermediate was subsequently protected with Boc_2_O to afford enantioenriched *N*‐Boc‐protected pyrrolidine **9** in 38% yield, without erosion of enantiomeric ratio (Scheme 3A). In addition, ethyl‐ester containing **8b** was oxidised to the corresponding lactam **10** in 33% yield using a 9‐azabicyclo[3,3,1]nonan‐3‐one‐9‐oxyl (ketoABNO)/*m*CPBA‐mediated oxidation protocol developed by Lin et al. (Scheme 3B) [80].

Finally, we were pleased to find that reducing the catalyst loading to 10 mol% enabled the conversion of spiropyrrolidine precursor **7n** to enantioenriched spiropyrrolidine **8n** on a 1.5 g scale in 98% yield and 96:4 er (Scheme 3C). The enantiomeric ratio was further improved to 97:3 er following a single recrystallization from hexane. The enantioenriched material was then transformed into the corresponding azide **12** in 65% yield over two steps without loss of optical purity. This sequence involved the reduction of the ester to alcohol **11** using LiAlH_4_, followed by a Mitsunobu reaction with diphenylphosphoryl azide. The resulting azide underwent a copper‐catalysed [3+2] cycloaddition with biotin‐derived alkyne (3a*S*,4*S*,6a*R*)‐hexahydro‐2‐oxo‐*N*‐2‐propyn‐1‐yl‐1*H*‐thieno[3,4‐*d*]imidazole‐4‐pentanamide, furnishing biotin derivative **13** in 70% yield and with excellent diastereoselectivity (>20:1 dr).

## 3 Conclusion

In conclusion, we have developed the first general catalytic enantioselective intramolecular aza‐Michael reaction of α,β‐unsaturated esters without the need for preactivation strategies. Through careful modification of a modular bifunctional iminophosphorane catalyst and extensive reaction optimisation, we achieved high yields (up to 99%) and enantioselectivities (up to 97.5:2.5 er) across a broad scope of 31 substrates, demonstrating the robustness and synthetic utility of the method. Notably, this protocol is operationally simple: all reactions were performed under air with technical‐grade solvents, underscoring the tolerance of the reaction to trace moisture. The reaction is readily scalable to a 1.5 g scale with reduced catalyst loading, delivering improved yield (98%) and enantiomeric ratio (96:4 er; 97:3 er after a single recrystallization). This practical and efficient method provides streamlined access to highly enantioenriched nitrogen‐containing heterocycles, particularly pyrrolidines and spiropyrrolidines, which are valuable scaffolds in pharmaceutical chemistry [4] and natural product synthesis [1]. Efforts to expand the range of enantioselective transformations amenable to BIMP catalysis are ongoing in our laboratory, and the results will be reported in due course.

## Author Contributions

**Evan G. W. Rutter**: investigation, methodology, formal analysis, and writing – original draft, review and editing. **Cameron J. MacRae**: investigation, methodology, and formal analysis. **Haoran Xiong**: methodology, investigation, and formal analysis. **Daniel Rozsar**: investigation, methodology, formal analysis, supervision, and conceptualization. **Katherine F. P. Clarke**: data curation and formal analysis. **Darren J. Dixon**: conceptualization, supervision, project management, and writing – original draft, review and editing.

## Conflicts of Interest

The authors declare no conflicts of interest.

## Supporting information




**Supporting File**: anie72927‐sup‐0001‐SuppMat.pdf.

## Data Availability

The data that support the findings of this study are available in the Supporting Information of this article.

## References

[1] C. Bhat and S. G. Tilve , “Recent Advances in the Synthesis of Naturally Occurring Pyrrolidines, Pyrrolizidines and Indolizidine Alkaloids Using Proline as a Unique Chiral Synthon,” RSC Advances 4 (2014): 5405–5452, 10.1039/c3ra44193h.

[2] B. Gao , B. Yang , X. Feng , and C. Li , “Recent Advances in the Biosynthesis Strategies of Nitrogen Heterocyclic Natural Products,” Natural Product Reports 39 (2022): 139–162, 10.1039/D1NP00017A. 34374396

[3] B. De , “Alkaloids,” in Chemical Diversity of Plant Specialized Metabolites: A Biosynthetic Approach (Royal Society of Chemistry, 2023), 218–272, 10.1039/9781837671472.

[4] C. M. Marshall , J. G. Federice , C. N. Bell , P. B. Cox , and J. T. Njardarson , “An Update on the Nitrogen Heterocycle Compositions and Properties of U.S. FDA‐Approved Pharmaceuticals (2013–2023),” Journal of Medicinal Chemistry 67 (2024): 11622–11655, 10.1021/acs.jmedchem.4c01122. 38995264

[5] E. Vitaku , D. T. Smith , and J. T. Njardarson , “Analysis of the Structural Diversity, Substitution Patterns, and Frequency of Nitrogen Heterocycles Among U.S. FDA Approved Pharmaceuticals,” Journal of Medicinal Chemistry 57 (2014): 10257–10274, 10.1021/jm501100b. 25255204

[6] M. M. Heravi and V. Zadsirjan , “Prescribed Drugs Containing Nitrogen Heterocycles: An Overview,” RSC Advances 10 (2020): 44247–44311, 10.1039/D0RA09198G. 35557843 PMC9092475

[7] G. Li Petri , M. V. Raimondi , V. Spanò , R. Holl , P. Barraja , and A. Montalbano , “Pyrrolidine in Drug Discovery: A Versatile Scaffold for Novel Biologically Active Compounds,” Topics in Current Chemistry 379 (2021): 34. 34373963 10.1007/s41061-021-00347-5 PMC8352847

[8] J. B. Fell , J. P. Fischer , B. R. Baer , et al., “Identification of the Clinical Development Candidate MRTX849 , a Covalent KRAS G12C Inhibitor for the Treatment of Cancer,” Journal of Medicinal Chemistry 63 (2020): 6679–6693, 10.1021/acs.jmedchem.9b02052. 32250617

[9] S. J. S. Bajwa and J. Kaur , “Clinical Profile of Levobupivacaine in Regional Anesthesia: A Systematic Review,” Journal of Anaesthesiology, Clinical Pharmacology 29 (2013): 530–539, 10.4103/0970-9185.119172. 24249993 PMC3819850

[10] H. F. Schran , L. Petryk , C.‐T. Chang , R. O'Connor , and M. B. Gelbert , “The Pharmacokinetics and Bioavailability of Clemastine and Phenylpropanolamine in Single‐Component and Combination Formulations,” Journal of Clinical Pharmacology 36 (1996): 911–922, 10.1002/j.1552-4604.1996.tb04758.x. 8930778

[11] M. T. Varela , G. G. Dias , L. F. N. de Oliveira , et al., “Spirocyclic Compounds as Innovative Tools in Drug Discovery for Medicinal Chemists,” European Journal of Medicinal Chemistry 287 (2025): 117368, 10.1016/j.ejmech.2025.117368. 39952099

[12] Y.‐J. Zheng and C. M. Tice , “The Utilization of Spirocyclic Scaffolds in Novel Drug Discovery,” Expert Opinion On Drug Discovery 11 (2016): 831–834, 10.1080/17460441.2016.1195367. 27233084

[13] K. Hiesinger , D. Dar'in , E. Proschak , and M. Krasavin , “Spirocyclic Scaffolds in Medicinal Chemistry,” Journal of Medicinal Chemistry 64 (2021): 150–183, 10.1021/acs.jmedchem.0c01473. 33381970

[14] C. Rodríguez‐Arias , R. Miguélez , Y. Holota , P. K. Mykhailiuk , and P. Barrio , “Approach to Heterospirocycles for Medicinal Chemistry,” Organic Letters 27 (2025): 10342–10347, 10.1021/acs.orglett.5c03125. 40910574 PMC12455649

[15] S. S. Berry and S. Jones , “Current Applications of Kinetic Resolution in the Asymmetric Synthesis of Substituted Pyrrolidines,” Organic & Biomolecular Chemistry 19 (2021): 10493–10515, 10.1039/D1OB01943K. 34842884

[16] J. C. Carretero , N. Rodríguez , and J. Adrio , “Metal Catalyzed Asymmetric 1,3‐dipolar Cycloaddition of Azomethine Ylides: Structural Diversity at the Dipole Partner,” Chemical Communications 61 (2025): 3821–3831, 10.1039/D4CC06484D. 39945035

[17] M. Sánchez‐Roselló , M. Escolano , D. Gaviña , and C. Del Pozo , “Two Decades of Progress in the Asymmetric Intramolecular Aza‐Michael Reaction,” Chemical Record 22 (2022): e202100161, 10.1002/tcr.202100161. 34415097

[18] J. M. Longmire , B. Wang , and X. Zhang , “Highly Enantioselective Ag(I)‐Catalyzed [3 + 2] Cycloaddition of Azomethine Ylides,” Journal of the American Chemical Society 124 (2002): 13400–13401, 10.1021/ja025969x. 12418889

[19] C. Chen , X. Li , and S. L. Schreiber , “Catalytic Asymmetric [3+2] Cycloaddition of Azomethine Ylides. Development of a Versatile Stepwise, Three‐Component Reaction for Diversity‐Oriented Synthesis,” Journal of the American Chemical Society 125 (2003): 10174–10175, 10.1021/ja036558z. 12926931

[20] Z.‐M. Zhang , B. Xu , S. Xu , H.‐H. Wu , and J. Zhang , “Diastereo‐ and Enantioselective Copper(I)‐Catalyzed Intermolecular [3+2] Cycloaddition of Azomethine Ylides With β‐Trifluoromethyl β,β‐Disubstituted Enones,” Angewandte Chemie International Edition 55 (2016): 6324–6328, 10.1002/anie.201602542. 27073118

[21] K. Morisaki , Y. Furuki , R. Kousaka , S. Nagai , Y. Oonishi , and Y. Sato , “Reflexive Chirality Transfer (RCT): Asymmetric 1,3‐Dipolar Cycloaddition of α‐Amino Acid Schiff Base With Nonchiral Copper Catalyst,” Journal of the American Chemical Society 147 (2025): 12740–12748, 10.1021/jacs.5c00965. 40168187

[22] A. Inoue , K. Hosono , S. Furuya , and S.‐I. Fukuzawa , “Synthesis of Spirocyclic Pyrrolidine Compounds via Silver‐Catalyzed Asymmetric [3 + 2] Cycloaddition Reaction of Imino Esters With α‐Alkylidene Succinimides,” Journal of Organic Chemistry 89 (2024): 1249–1255, 10.1021/acs.joc.3c02456. 38174971

[23] Y. Suzuki , K. Kanemoto , A. Inoue , K. Imae , and S.‐I. Fukuzawa , “Silver/ThioClickFerrophos‐Catalyzed 1,3‐Dipolar Cycloaddition and Tandem Addition–Elimination Reaction of Morita–Baylis–Hillman Adducts,” Journal of Organic Chemistry 86 (2021): 14586–14596, 10.1021/acs.joc.1c01440. 34661412

[24] H. Zhu , P. Chen , and G. Liu , “Palladium‐Catalyzed Intramolecular Aminoacetoxylation of Unactivated Alkenes With Hydrogen Peroxide as Oxidant,” Organic Letters 17 (2015): 1485–1488, 10.1021/acs.orglett.5b00373. 25742490

[25] X. Qi , C. Chen , C. Hou , L. Fu , P. Chen , and G. Liu , “Enantioselective Pd(II)‐Catalyzed Intramolecular Oxidative 6‐ endo Aminoacetoxylation of Unactivated Alkenes,” Journal of the American Chemical Society 140 (2018): 7415–7419, 10.1021/jacs.8b03767. 29812946

[26] X. Yang , P. Chen , and G. Liu , “Asymmetric 1,n‐Remote Aminoacetoxylation of Unactivated Internal Alkenes Enabled by Palladium Catalysis,” Angewandte Chemie International Edition 63 (2024): e202408305, 10.1002/anie.202408305. 38760326

[27] M. Kojima and K. Mikami , “Enantioselective Intramolecular Hydroamination of N‐Alkenyl Ureas Catalyzed by Tropos BIPHEP–Gold(I) Complexes With Au–Au Interaction,” Synlett 23 (2012): 57–61.

[28] S. D. Lee , J. C. Timmerman , and R. A. Widenhoefer , “Enantioselective Intramolecular Hydroamination of Unactivated Alkenes Catalyzed by Mono‐ and Bis(gold) Phosphine Complexes,” Advanced Synthesis & Catalysis 356 (2014): 3187–3192, 10.1002/adsc.201400268.

[29] M.‐A. Abadie , X. Trivelli , F. Medina , et al., “Gold(I)‐Catalysed Asymmetric Hydroamination of Alkenes: A Silver‐ and Solvent‐Dependent Enantiodivergent Reaction,” Chemistry: A European Journal 23 (2017): 10777–10788, 10.1002/chem.201701301. 28488394

[30] X. Shen and S. L. Buchwald , “Rhodium‐Catalyzed Asymmetric Intramolecular Hydroamination of Unactivated Alkenes,” Angewandte Chemie International Edition 49 (2010): 564–567, 10.1002/anie.200905402. 20014268 PMC3394429

[31] K. Manna , W. C. Everett , G. Schoendorff , A. Ellern , T. L. Windus , and A. D. Sadow , “Highly Enantioselective Zirconium‐Catalyzed Cyclization of Aminoalkenes,” Journal of the American Chemical Society 135 (2013): 7235–7250, 10.1021/ja4000189. 23631736

[32] K. Manna , S. Xu , and A. D. Sadow , “A Highly Enantioselective Zirconium Catalyst for Intramolecular Alkene Hydroamination: Significant Isotope Effects on Rate and Stereoselectivity,” Angewandte Chemie International Edition 50 (2011): 1865–1868, 10.1002/anie.201006163. 21328658

[33] M. C. Wood , D. C. Leitch , C. S. Yeung , J. A. Kozak , and L. L. Schafer , “Chiral Neutral Zirconium Amidate Complexes for the Asymmetric Hydroamination of Alkenes,” Angewandte Chemie International Edition 46 (2007): 354–358, 10.1002/anie.200603017. 17029322

[34] B. Cui , Y. Zheng , H. Sun , et al., “Catalytic Enantioselective Intramolecular Hydroamination of Alkenes Using Chiral Aprotic Cyclic Urea Ligand on Manganese (II),” Nature Communications 15 (2024): 6647, 10.1038/s41467-024-50757-4. PMC11300822 39103345

[35] L. Ackermann and A. Althammer , “Phosphoric Acid Diesters as Efficient Catalysts for Hydroaminations of Nonactivated Alkenes and an Application to Asymmetric Hydroaminations,” Synlett 2008 (2008): 995–998, 10.1055/s-2008-1072505.

[36] J.‐S. Lin , P. Yu , L. Huang , P. Zhang , B. Tan , and X.‐Y. Liu , “Brønsted Acid Catalyzed Asymmetric Hydroamination of Alkenes: Synthesis of Pyrrolidines Bearing a Tetrasubstituted Carbon Stereocenter,” Angewandte Chemie International Edition 54 (2015): 7847–7851, 10.1002/anie.201501762. 26013971

[37] Z.‐L. Yu , Y.‐F. Cheng , N.‐C. Jiang , et al., “Desymmetrization of Unactivated Bis‐alkenes via Chiral Brønsted Acid‐Catalysed Hydroamination,” Chemical Science 11 (2020): 5987–5993, 10.1039/D0SC00001A. 34094089 PMC8159283

[38] S. Guria , A. N. Volkov , R. Khudaverdyan , et al., “Enantioselective, Bro̷Nsted Acid‐Catalyzed Anti‐Selective Hydroamination of Alkenes,” Journal of the American Chemical Society 146 (2024): 17180–17188, 10.1021/jacs.4c03306. 38875460

[39] F. C. Raps , A. Rivas‐Souchet , C. M. Jones , and T. K. Hyster , “Emergence of a Distinct Mechanism of C–N Bond Formation in Photoenzymes,” Nature 637 (2025): 362–368, 10.1038/s41586-024-08138-w. 39378905 PMC11771027

[40] K. Takasu , S. Maiti , and M. Ihara , “Asymmetric Intramolecular Aza‐Michael Reaction Using Environmentally Friendly Organocatalysis,” Heterocycles 59 (2003): 51–55.

[41] S. Fustero , D. Jiménez , J. Moscardó , S. Catalán , and C. del Pozo , “Enantioselective Organocatalytic Intramolecular Aza‐Michael Reaction: A Concise Synthesis of (+)‐Sedamine, (+)‐Allosedamine, and (+)‐Coniine,” Organic Letters 9 (2007): 5283–5286, 10.1021/ol702447y. 17985918

[42] E. C. Carlson , L. K. Rathbone , H. Yang , N. D. Collett , and R. G. Carter , “Improved Protocol for Asymmetric, Intramolecular Heteroatom Michael Addition Using Organocatalysis: Enantioselective Syntheses of Homoproline, Pelletierine, and Homopipecolic Acid,” Journal of Organic Chemistry 73 (2008): 5155–5158, 10.1021/jo800749t. 18529081 PMC2474690

[43] S. Fustero , J. Moscardó , D. Jiménez , M. D. Pérez‐Carrión , M. Sánchez‐Roselló , and C. Del Pozo , “Organocatalytic Approach to Benzofused Nitrogen‐Containing Heterocycles: Enantioselective Total Synthesis of (+)‐Angustureine,” Chemistry: A European Journal 14 (2008): 9868–9872, 10.1002/chem.200801480. 18830991

[44] Q. Cai , C. Zheng , and S.‐L. You , “Enantioselective Intramolecular Aza‐Michael Additions of Indoles Catalyzed by Chiral Phosphoric Acids,” Angewandte Chemie International Edition 49 (2010): 8666–8669, 10.1002/anie.201003919. 20931585

[45] J.‐D. Liu , Y.‐C. Chen , G.‐B. Zhang , et al., “Asymmetric Organocatalytic Intramolecular Aza‐Michael Addition of Enone Carbamates: Catalytic Enantioselective Access to Functionalized 2‐Substituted Piperidines,” Advanced Synthesis & Catalysis 353 (2011): 2721–2730, 10.1002/adsc.201100282.

[46] S. Fustero , C. del Pozo , C. Mulet , R. Lazaro , and M. Sánchez‐Roselló , “Microwave‐Assisted Organocatalytic Enantioselective Intramolecular Aza‐Michael Reaction With α,β‐Unsaturated Ketones,” Chemistry: A European Journal 17 (2011): 14267–14272. 22083999 10.1002/chem.201101292

[47] R. Miyaji , K. Asano , and S. Matsubara , “Asymmetric Indoline Synthesis via Intramolecular Aza‐Michael Addition Mediated by Bifunctional Organocatalysts,” Organic Letters 15 (2013): 3658–3661, 10.1021/ol401538b. 23844669

[48] H. Liu , C. Zeng , J. Guo , M. Zhang , and S. Yu , “Enantioselective Synthesis of 2‐Substituted Pyrrolidinesvia Domino Cross Metathesis/Intramolecular Aza‐Michael Addition,” RSC Advances 3 (2013): 1666–1668, 10.1039/C2RA22374K.

[49] C. J. Maddocks , K. Ermanis , and P. A. Clarke , “Asymmetric “Clip‐Cycle” Synthesis of Pyrrolidines and Spiropyrrolidines,” Organic Letters 22 (2020): 8116–8121, 10.1021/acs.orglett.0c03090. 32991808

[50] S. Ravi , C. J. Maddocks , I. J. S. Fairlamb , W. P. Unsworth , and P. A. Clarke , “Asymmetric ‘Clip‐Cycle’ synthesis of 3‐spiropiperidines,” Organic & Biomolecular Chemistry 23 (2025): 649–653, 10.1039/D4OB01608D. 39589281

[51] L. C. Duff , S. Yilmaz , A. Agora , et al., “‘Clip‐Cycle’ Approaches to Functionalised Pyrrolidines, Pyrrolizidines and Indolizidines,” Organic & Biomolecular Chemistry 24 (2026): 608–612, 10.1039/D5OB01746G. 41408862

[52] M. Bandini , A. Eichholzer , M. Tragni , and A. Umani‐Ronchi , “Enantioselective Phase‐Transfer‐Catalyzed Intramolecular Aza‐Michael Reaction: Effective Route to Pyrazino‐Indole Compounds,” Angewandte Chemie International Edition 47 (2008): 3238–3241, 10.1002/anie.200705685. 18348111

[53] M. Bandini , A. Bottoni , A. Eichholzer , G. P. Miscione , and M. Stenta , “Asymmetric Phase‐Transfer‐Catalyzed Intramolecular N‐Alkylation of Indoles and Pyrroles: A Combined Experimental and Theoretical Investigation,” Chemistry: A European Journal 16 (2010): 12462–12473, 10.1002/chem.201000560. 20839181

[54] X. Xiao , X. Liu , S. Dong , Y. Cai , L. Lin , and X. Feng , “Asymmetric Synthesis of 2,3‐Dihydroquinolin‐4‐One Derivatives Catalyzed by a Chiral Bisguanidium Salt,” Chemistry: A European Journal 18 (2012): 15922–15926, 10.1002/chem.201203216. 23154811

[55] G. Su , C. J. Thomson , K. Yamazaki , et al., “A Bifunctional Iminophosphorane Squaramide Catalyzed Enantioselective Synthesis of Hydroquinazolines via Intramolecular Aza‐Michael Reaction to α,β‐unsaturated Esters,” Chemical Science 12 (2021): 6064–6072, 10.1039/D1SC00856K. 33996002 PMC8098679

[56] T. K. Roy , B. Parhi , and P. Ghorai , “Cinchonamine Squaramide Catalyzed Asymmetric Aza‐Michael Reaction: Dihydroisoquinolines and Tetrahydropyridines,” Angewandte Chemie International Edition 57 (2018): 9397–9401, 10.1002/anie.201805020. 29882619

[57] C. K. Chung , Z. Liu , K. W. Lexa , et al., “Asymmetric Hydrogen Bonding Catalysis for the Synthesis of Dihydroquinazoline‐Containing Antiviral, Letermovir,” Journal of the American Chemical Society 139 (2017): 10637–10640, 10.1021/jacs.7b05806. 28737937

[58] T. T. Metsänen , K. W. Lexa , C. B. Santiago , et al., “Combining Traditional 2D and Modern Physical Organic‐derived Descriptors to Predict Enhanced Enantioselectivity for the Key aza‐Michael Conjugate Addition in the Synthesis of Prevymis™ (letermovir),” Chemical Science 9 (2018): 6922–6927, 10.1039/C8SC02089B. 30210766 PMC6124913

[59] H. Joshi , A. Manna , S. Nagamalla , A. A. Thomas , and S. Sathyamoorthi , “A Catalytic, Enantioselective Sulfamate Tethered Aza ‐Michael Cyclization,” Organic Letters 26 (2024): 10708–10713, 10.1021/acs.orglett.4c03558. 39660506 PMC11662505

[60] D. S. Allgäuer , H. Jangra , H. Asahara , et al., “Quantification and Theoretical Analysis of the Electrophilicities of Michael Acceptors,” Journal of the American Chemical Society 139 (2017): 13318–13329, 10.1021/jacs.7b05106. 28921959

[61] M. Sánchez‐Roselló , C. Mulet , M. Guerola , C. Del Pozo , and S. Fustero , “Microwave‐Assisted Tandem Organocatalytic Peptide‐Coupling Intramolecular Aza‐Michael Reaction: Α,β‐Unsaturated N ‐Acyl Pyrazoles as Michael Acceptors,” Chemistry: A European Journal 20 (2014): 15697–15701, 10.1002/chem.201404596. 25336358

[62] M. G. Núñez , A. J. M. Farley , and D. J. Dixon , “Bifunctional Iminophosphorane Organocatalysts for Enantioselective Synthesis: Application to the Ketimine Nitro‐Mannich Reactio,” Journal of the American Chemical Society 135 (2013): 16348–16351. 24107070 10.1021/ja409121s PMC3931333

[63] M. Formica , D. Rozsar , G. Su , A. J. M. Farley , and D. J. Dixon , “Bifunctional Iminophosphorane Superbase Catalysis: Applications in Organic Synthesis,” Accounts of Chemical Research 53 (2020): 2235–2247, 10.1021/acs.accounts.0c00369. 32886474

[64] T. Takeda and M. Terada , “Development of a Chiral Bis(guanidino)Iminophosphorane as an Uncharged Organosuperbase for the Enantioselective Amination of Ketones,” Journal of the American Chemical Society 135 (2013): 15306–15309, 10.1021/ja408296h. 24074350

[65] P. de Jesús Cruz , W. R. Cassels , C.‐H. Chen , and J. S. Johnson , “Doubly Stereoconvergent Crystallization Enabled by Asymmetric Catalysis,” Science 376 (2022): 1224–1230. 35679416 10.1126/science.abo5048 PMC9467684

[66] A. Budeev , J. Dong , D. Häussinger , and C. Sparr , “Catalyst Control Over Pentavalent Stereocentres,” Nature Communications 14 (2023): 8013, 10.1038/s41467-023-43750-w. PMC10696079 38049395

[67] D. Uraguchi , S. Sakaki , and T. Ooi , “Chiral Tetraaminophosphonium Salt‐Mediated Asymmetric Direct Henry Reaction,” Journal of the American Chemical Society 129 (2007): 12392–12393, 10.1021/ja075152+. 17880221

[68] Y. Zhang , X.‐Y. Wu , and J. Han , “Chiral Iminophosphorane Catalyzed Asymmetric Henry Reaction of α,β‐alkynyl Ketoesters,” Chinese Chemical Letters 30 (2019): 1519–1522, 10.1016/j.cclet.2019.04.042.

[69] Z. Wu , S. Fang , J. He , et al., “Desymmetrization/Kinetic Resolution of Planar Chiral [2.2]Paracyclophanes by Bioinspired Peptide‐Iminophosphorane Catalysis,” Angewandte Chemie International Edition 64 (2025): e202423702, 10.1002/anie.202423702. 39945721

[70] M. Formica , T. Rogova , H. Shi , et al., “Catalytic Enantioselective Nucleophilic Desymmetrization of Phosphonate Esters,” Nature Chemistry 15 (2023): 714–721, 10.1038/s41557-023-01165-6. PMC10159838 37127757

[71] M. Formica , B. Ferko , T. Marsh , T. A. Davidson , K. Yamazaki , and D. J. Dixon , “Second Generation Catalytic Enantioselective Nucleophilic Desymmetrization at Phosphorus (V): Improved Generality, Efficiency and Modularity,” Angewandte Chemie International Edition 63 (2024): e202400673, 10.1002/anie.202400673. 38381534

[72] K. Yuan , A. I. Ristache , S. M. Kosc , A. Crumpton , and D. J. Dixon , “Bifunctional Iminophosphorane Superbases Enable the Highly Enantioselective Sulfa‐Michael Addition to Fully Substituted Cyclopropene Carboxylic Acid Derivatives,” Journal of the American Chemical Society 147 (2025): 40045–40050, 10.1021/jacs.5c07849. 41123596 PMC12593379

[73] D. Rozsar , A. J. M. Farley , I. McLauchlan , B. D. A. Shennan , K. Yamazaki , and D. J. Dixon , “Bifunctional Iminophosphorane‐Catalyzed Enantioselective Nitroalkane Addition to Unactivated α,β‐Unsaturated Esters**,” Angewandte Chemie International Edition 62 (2023): e202303391, 10.1002/anie.202303391. 36929179 PMC10946890

[74] C. Y. X. Poh , D. Rozsar , J. Yang , K. E. Christensen , and D. J. Dixon , “Bifunctional Iminophosphorane Catalyzed Amide Enolization for Enantioselective Cyclohexadienone Desymmetrization,” Angewandte Chemie International Edition 63 (2024): e202315401, 10.1002/anie.202315401. 38055190

[75] J. C. Golec , D.‐H. Tan , K. Yamazaki , et al., “Catalytic Enantioselective Synthesis of Alkylidenecyclopropanes,” Nature 645 (2025): 932–938, 10.1038/s41586-025-09485-y. 40789330 PMC12460155

[76] J. Che , S. Fang , Z. Liu , et al., “Biomimetic Peptide‐Iminophosphorane Superbases Enable Atroposelective Synthesis of C–O Axially Chiral Diaryl Ethers,” ACS Catalysis 15 (2025): 19005–19016, 10.1021/acscatal.5c06219.

[77] G. Su , M. Formica , K. Yamazaki , T. A. Hamlin , and D. J. Dixon , “Catalytic Enantioselective Intramolecular Oxa‐Michael Reaction to α,β‐Unsaturated Esters and Amides,” Journal of the American Chemical Society 145 (2023): 12771–12782, 10.1021/jacs.3c03182. 37253087 PMC10273320

[78] Deposition Numbers 2541404 (for **8o**), 2541402 (for **8u**), 2541403 (for **8w**) contain the supplementary crystallographic data for this paper. These data are provided free of charge by the joint Cambridge Crystallographic Data Centre and Fachinformationszentrum Karlsruhe.

[79] R. M. Beesley , C. K. Ingold , and J. F. Thorpe , “CXIX.—The Formation and Stability of Spiro‐compounds. Part I. spiro‐Compounds From Cyclohexane,” Journal of the Chemical Society Transactions 107 (1915): 1080–1106, 10.1039/CT9150701080.

[80] J. Rein , B. Górski , Y. Cheng , Z. Lei , F. Buono , and S. Lin , “Oxoammonium‐Catalyzed Oxidation of N‐Substituted Amines,” Journal of the American Chemical Society 146 (2024): 31412–31419, 10.1021/jacs.4c11758. 39527490 PMC11912027

