# Supplementary material for: Catalytic Enantioselective Intramolecular Aza‐Michael Addition to α,β‐Unsaturated Esters
Source: Angew Chem Int Ed Engl. 2026 Jun 7;65(30):e5995443. doi: 10.1002/anie.5995443 (PMC13383253; doi:10.1002/anie.5995443)
Supplement: Supplementary file 1 — Supporting File: anie72927‐sup‐0001‐SuppMat.pdf. [file ANIE-65-e5995443-s001.pdf]

Supporting information for

Catalytic Enantioselective Intramolecular Aza-Michael Addition to α,β-Unsaturated Esters

Evan G. W. Rutter, Cameron MacRae, Haoran Xiong, Daniel Rozsar, Katherine F. P. Clarke, and Darren J. Dixon*

Department of Chemistry, Chemistry Research Laboratory,

University of Oxford, 12 Mansfield Road, Oxford OX1 3TA, UK

E-mail: darren.dixon@chem.ox.ac.uk

[back to table of contents]

Table of Contents

Table of Contents 1
General Experimental 2
Reaction Optimisation 3
General Procedures 8
Synthesis of Catalyst Precursors 9
Synthesis of Unsubstituted Pyrrolidine and Piperidine Precursors 11
Synthesis of *β*-Disubstituted Pyrrolidine and Piperidine Precursors 23
Synthesis of *α*-Disubstituted Pyrrolidine Precursors 34
Synthesis of Indoline, Isoindoline, and Tetrahydroquinoline Precursors 47
Synthesis of Enantioenriched Pyrrolidines and Piperidines 55
150 mg Scale Synthesis of Compound **8a** 79
Preparative Scale Synthesis of Compound **8n** 80
Synthesis of Product Derivatives 81
Unsuccessful Substrates 86
Single Crystal X-Ray Diffraction Data 87
^{1}H, ^{13}C and ^{19}F NMR Spectra of Novel Compounds 90
HPLC and SFC Traces 189
References 225

[back to table of contents]

General Experimental

Reagents and Solvents

All reactions were performed under an atmosphere of nitrogen unless otherwise stated. Reagents were used directly from commercial sources without purification, unless otherwise stated. Anhydrous solvents were obtained from an MBRAUN-SPS solvent purification system or from bottles with septa (Acros Organics, Thermo Fisher Scientific, Sigma Aldrich).

Chromatography

Thin-layer chromatography (TLC) was performed on Merck Kieselgel 60 PF254 pre-coated aluminium-backed TLC sheets. The plates were visualised with UV light, basic aqueous permanganate or anisaldehyde. Flash column chromatography (FCC) was carried out using Merk Geduran Si 60 silica gel, (40-63 μm). Enantiomeric excesses (ee) were obtained by chiral HPLC, performed on an Agilent 1200 series instrument using a selected chiral stationary phase column. The ee values were obtained by comparing against an appropriate racemic sample. Details of the stationary phase column used are described in the specific experiment. Chiral SFC (supercritical fluid chromatography) separations were conducted on a Waters Acquity UPC2 system using Waters Empower software. Chiralpak® columns (150x3 mm, particle size 3 μm) were used as specified in the text. Solvents used were of HPLC grade (Fisher Scientific, Sigma Aldrich or Rathburn).

Spectroscopy and Spectrometry

^{1}H, ^{13}C, ^{19}F, ^{31}P NMR spectroscopy was undertaken at ambient temperature using Bruker Avance III HD nanobay (equipped with a 9.4 T magnet, ^{1}H: 400.2 MHz, ^{13}C: 100.6 MHz, ^{19}F: 376.5 MHz, ^{31}P: 162.0 MHz), Bruker Avance III NMR (equipped with 11.75T magnet, ^{1}H: 500.3 MHz, ^{13}C: 125.8 MHz, ^{19}F: 565 MHz), Bruker NEO 600 (fitted with broadband helium cryoprobe equipped with a 14.1T magnet, ^{1}H: 600.4 MHz, ^{13}C: 151.0 MHz) NMR spectrometers. Chemical shifts (δ) are given in parts per million (ppm), relative to TMS (δ = 0) and referenced to residual solvent peaks ($CDCl_3$: δ_H 7.26 ppm, δ_C 77.16 ppm; [D_6]-DMSO: δ_H 2.50 ppm, δ_C 39.52 ppm) or ^{19}F NMR spectra are referenced to the internal standard fluorobenzene (PhF, δ_F -113.15 ppm). Coupling constants (J) are quoted in Hertz (Hz) to the nearest 0.1 Hz. NMR assignments use a numbering system independent from IUPAC, and are reported as (multiplicity, coupling constants, integration, assignments where applicable). The multiplicities are reported using the abbreviations d = doublet, t = triplet, q = quartet, p = quintet, h = sextet, hept = septet, dd = doublet of doublets, dt = doublet of 3 triplets, dq = doublet of quartets, ddd = doublet of doublet of doublets, m = multiplet. The structural assignment of novel compounds was corroborated through COSY, HSQC and HMBC experiments.

Melting Points and Specific Rotations

Melting points (M.P.) were measured using a Leica Galen III hot-stage 37 microscope apparatus and are reported in degrees Celsius (°C), uncorrected. Specific rotations were calculated from optical rotations measured on a Bellingham and Stanley ADP430 digital instrument with a 0.5 dm cell length.

Naming of Compounds

Compound names are generated by ChemDraw Professional 23.0.1 software.

Crystallography

Low temperature[1] single crystal X-ray diffraction data were collected using a Rigaku Oxford Diffraction SuperNova diffractometer. Raw frame data were reduced using CrysAlisPro and the structures were solved using 'Superflip'[2] before refinement with CRYSTALS as per the SI (CIF).[3-4]

[back to table of contents]

Reaction Optimisation

General procedure for optimization of the intramolecular aza-Michael reaction of sulfonamide 7a:

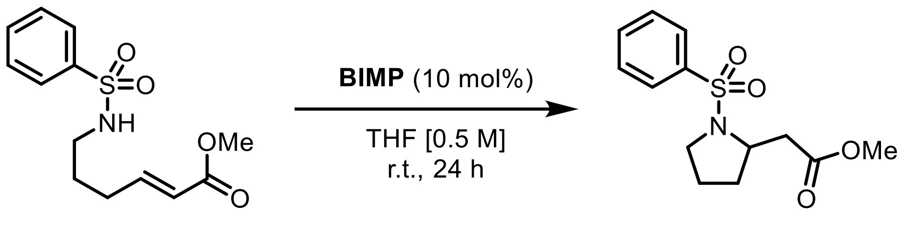

7a **8a**

To a stock solution of sulfonamide **7a** (28.3 mg, 0.1 mmol) in THF (0.5 M) was added to the appropriate BIMP catalyst (0.01 mmol, 10 mol%) in a vial which was then sealed under air at room temperature. The reaction mixture was stirred at room temperature for 24 h. The reaction was quenched by passing down a short silica plug and eluted with EtOAc : pentane. The eluent was concentrated in vacuo and analysed without further purification.

Catalyst Screen

Table S1. Detailed catalyst screen results for the intramolecular aza-Michael reaction of sulfonamide **7a**.**[a]** determined by ^{1}H NMR analysis of crude reaction mixture. **[b]** determined by HPLC analysis on a chiral stationary phase. PMP = *para*-methoxy phenyl; Cy = cyclohexyl.

entry	azide	phosphine	Conv. (%)[a]	e.r.[b]	entry	azide	phosphine	Conv. (%)[a]	e.r.[b]
1	**A1**	$P(PMP)_3$	66	71:29	**16**	**A12**	PCy_3	91	75.5:24.5
2	**A2**	$P(PMP)_3$	90	61:39	**17**	**A13**	$P(PMP)_3$	82	64.5:35.5
3	**A3**	$P(PMP)_3$	82	47:53	**18**	**A13**	PCy_3	82	64.5:35.5
4	**A3**	PCy_3	36	66:34	**19**	**A14**	$P(PMP)_3$	43	73:27
5	**A4**	$P(PMP)_3$	92	50:50	**20**	**A14**	PCy_3	99	70.5:28.5
6	**A5**	$P(PMP)_3$	80	47:53	**21**	**A15**	$P(PMP)_3$	99	64:36
7	**A6**	$P(PMP)_3$	100	75:25	**22**	**A15**	PCy_3	99	68.5:31.5
8	**A7**	$P(PMP)_3$	40	74:26	**23**	**A16**	$P(PMP)_3$	73	66.5:33.5
9	**A8**	$P(PMP)_3$	100	73:27	**24**	**A16**	PCy_3	99	81:19
10	**A9**	$P(PMP)_3$	14	52:48	**25**	**A17**	**PCy_3**	**99**	**83.5:16.5**
11	**A10**	$P(PMP)_3$	100	67.5:32.5	**26**	**A18**	PCy_3	94	68:32
12	**A10**	PCy_3	100	70:30	**27**	**A19**	$P(PMP)_3$	99	62
13	**A11**	$P(PMP)_3$	100	75:25	**28**	**A19**	PCy_3	99	65:35
14	**A11**	PCy_3	100	79:21	**29**	**A20**	PCy_3	99	81:19
15	**A12**	$P(PMP)_3$	65	66.5:33.5	**30**	**A21**	PCy_3	99	80.5:18.5

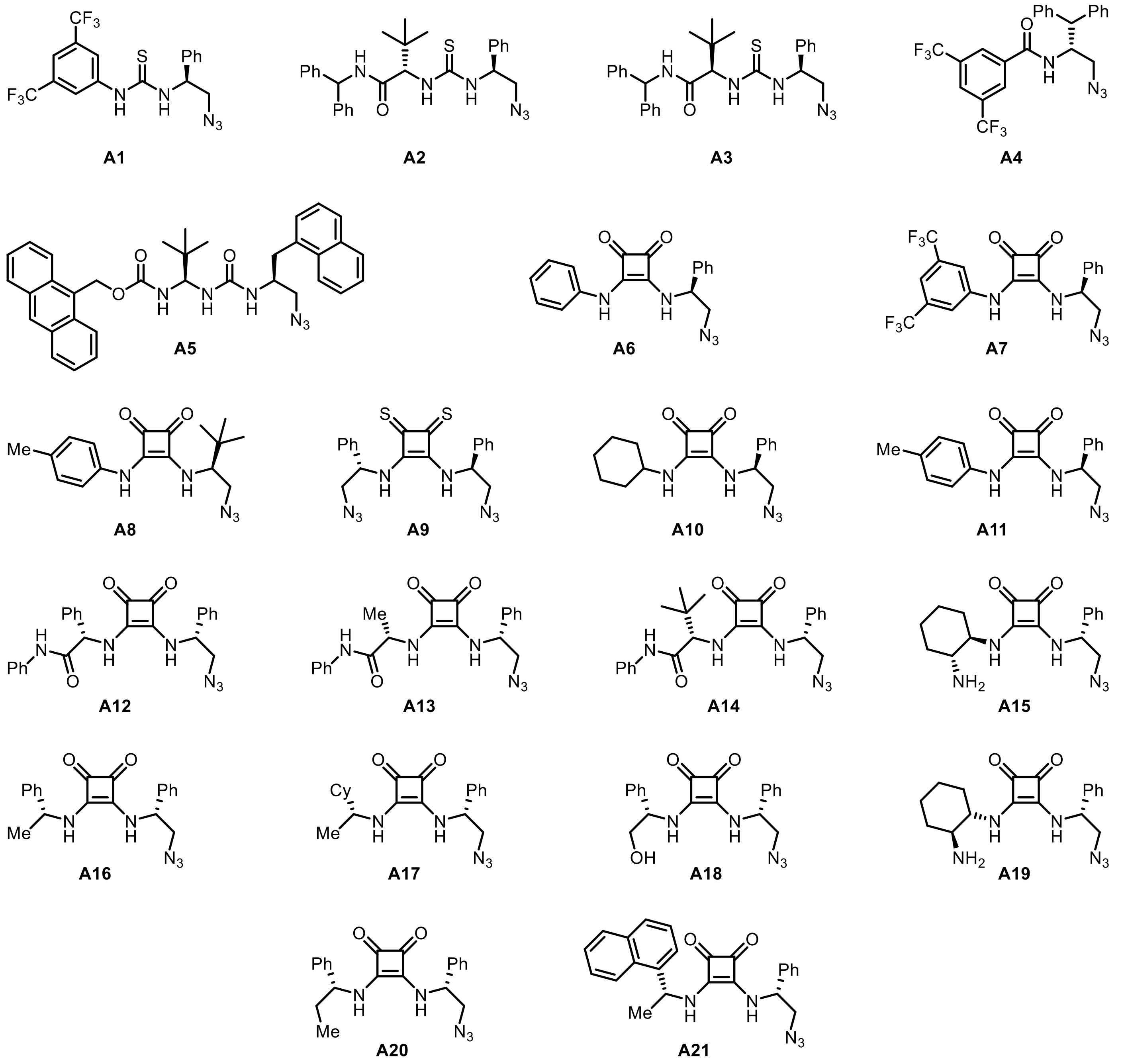

Figure S1. Selected azide pre-catalysts assessed in the optimisation of the intramolecular aza-Michael reaction of sulfonamide **7a.**

[back to table of contents]

Phosphine Screen

Table S2. Detailed phosphine screen results for the intramolecular aza-Michael reaction of sulfonamide **7a.[a]** determined by ^{1}H NMR analysis of crude reaction mixture. **[b]** determined by HPLC analysis on a chiral stationary phase. PMP = *para*-methoxy phenyl; Cy = cyclohexyl.

entry	azide	phosphine	Conv. (%)[a]	e.r.[b]
**1**	**A12**	P(PMP)$_3$	65	66.5:33.5
**2**	**A12**	**P1**	60	66:34
**3**	**A12**	P(*p*-tol)$_3$	28	67.5:32.5
**4**	**A12**	**P2**	64	63:37
**5**	**A12**	**P3**	54	55:45
**6**	**A12**	**P4**	68	66:34
**7**	**A12**	P(*p*-Cl-C$_6$H$_4$)$_3$	0	n.d.
**8**	**A12**	PCy$_3$	91	75.5:24.5
**9**	**A12**	P(cyclopentyl)$_3$	99	70:30
**10**	**A12**	PPh$_3$	12	57.5:42.5
**11**	**A17**	**PCy$_3$**	**99**	**83.5:16.5**

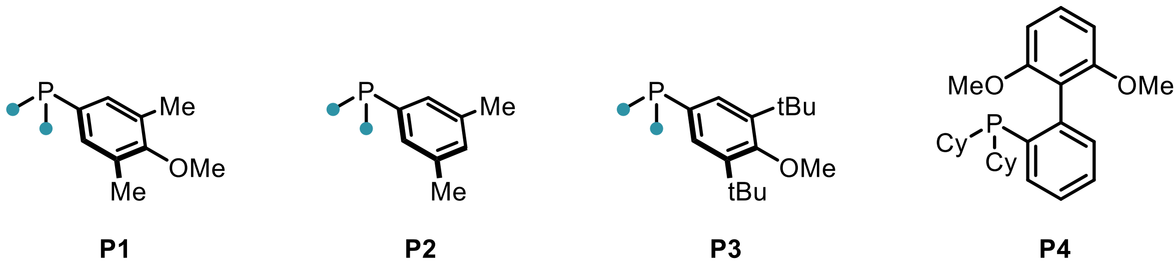

Figure S2. Selected phosphines assessed in the optimisation of the intramolecular aza-Michael reaction of sulfonamide **7a.**

[back to table of contents]

Solvent and Conditions Optimisation

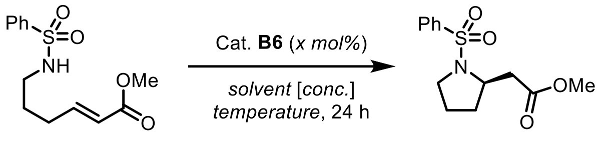

7a 8a

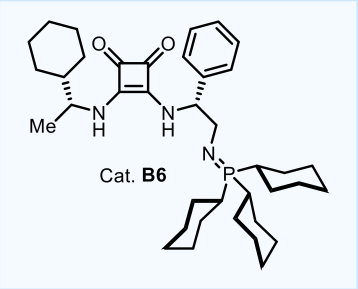

Table S3. Detailed solvent and conditions optimization results for the intramolecular aza-Michael reaction of sulfonamide **7a**. [a] determined by ^{1}H NMR analysis of crude reaction mixture. [b] determined by HPLC analysis on chiral stationary phase. r.t. = room temperature. TBME = tert-butyl methyl ether; THP = tetrahydropyran; CPME = cyclopentyl methyl ether; DME = 1,2-dimethoxyethane.

entry	cat. loading (mol%)	solvent	concentration (M)	temperature (°C)	conv.[a]	e.r.[b]
**1**	10	THF	0.5	r.t.	99	83.5:16.5
**2**	10	Toluene	0.5	r.t.	99	80.5:19.5
**3**	10	EtOAc	0.5	r.t.	99	78.5:21.5
**4**	10	TBME	0.5	r.t.	99	82:18
**5**	10	DCM	0.5	r.t.	99	73.5:26.5
**6**	10	Et_2O	0.5	r.t.	99	82:18
**7**	10	DMF	0.5	r.t.	99	66.5:33.5
**8**	10	MeCN	0.5	r.t.	99	69.5:30.5
**9**	10	1,4-dioxane	0.5	r.t.	99	87.5:12.5
**10**	10	2-Me-THF	0.5	r.t.	99	85.5:14.5
**11**	10	THP	0.5	r.t.	99	85:15
**12**	10	CPME	0.5	r.t.	99	83:17
**13**	10	2-Me-THF	0.5	40	99	79:21
**14**	10	2-Me-THF	0.5	-5	99	82
**15**	10	2-Me-THF	0.5	-22	50	94:6
**16**	20	2-Me-THF	0.5	-22	99	91:9
**17**	20	2-Me-THF	0.5	-30	45	91:9
**18**	20	Diisoproyl ether	0.1	-22	68	90:10
**19**	20	DME	0.5	-22	99	87:13
**20**	20	Diglyme	0.5	-22	99	90.5:9.5
**21**	20	Anisole	0.5	-22	99	89:11
**22**	20	Dioxane/2-Me-THF 1/1 (v/v)	0.5	-22	99	92.5:7.5

entry	cat. loading (mol%)	solvent	concentration (M)	temperature (°C)	conv.[a]	e.r.[b]
**23**	20	Dioxane/THP 1/1 (v/v)	0.5	-22	99	93.5:6.5
**24**	20	THP	0.5	-22	99	92:8
**25**	20	THP	0.2	-22	99	93:7
**26**	**20**	**THP**	**0.1**	**-22**	**99**	**93.5:6.5**
**27**	5	1,4-dioxane	0.5	r.t.	78	86.5:13.5
**28**	20	1,4-dioxane	0.5	r.t.	99	85:15
**29**	10	1,4-dioxane	0.2	r.t.	56	87:13
**30**	10	1,4-dioxane	1.0	r.t.	99	84:16

[back to table of contents]

General Procedures

General Procedure 1 Hoveyda Grubbs II Metathesis

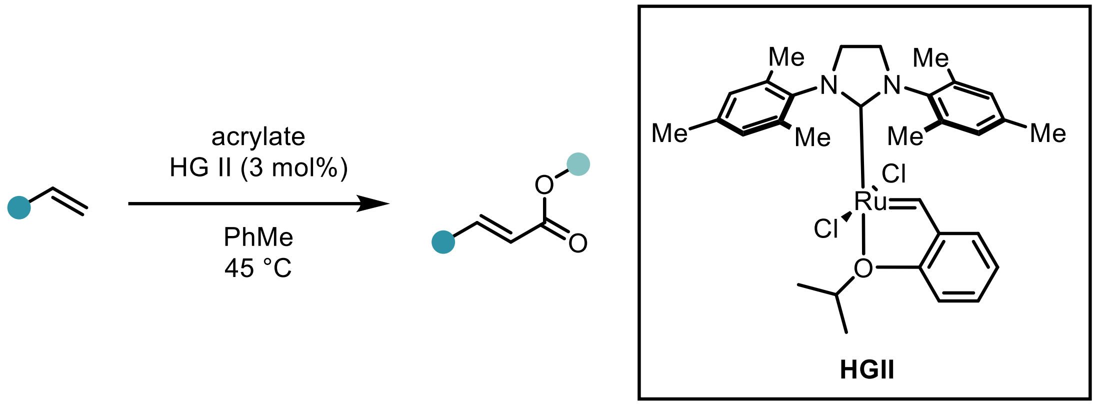

In a flame dried Schlenk flask under a nitrogen atmosphere, the appropriate alkene (1 equiv) was dissolved in the neat appropriate acrylate (10 equiv.) and a minimum volume of toluene. A solution of Hoveyda-Grubbs second generation catalyst (3 mol%) dissolved in a minimum volume of toluene was added dropwise over1 h, and the reaction mixture stirred at 45 °C overnight. Once complete, the reaction mixture was directly transferred to a column of silica gel and purified by FCC according to the specific experiment to yield pure unsaturated ester.

Synthesis of Catalyst Precursors

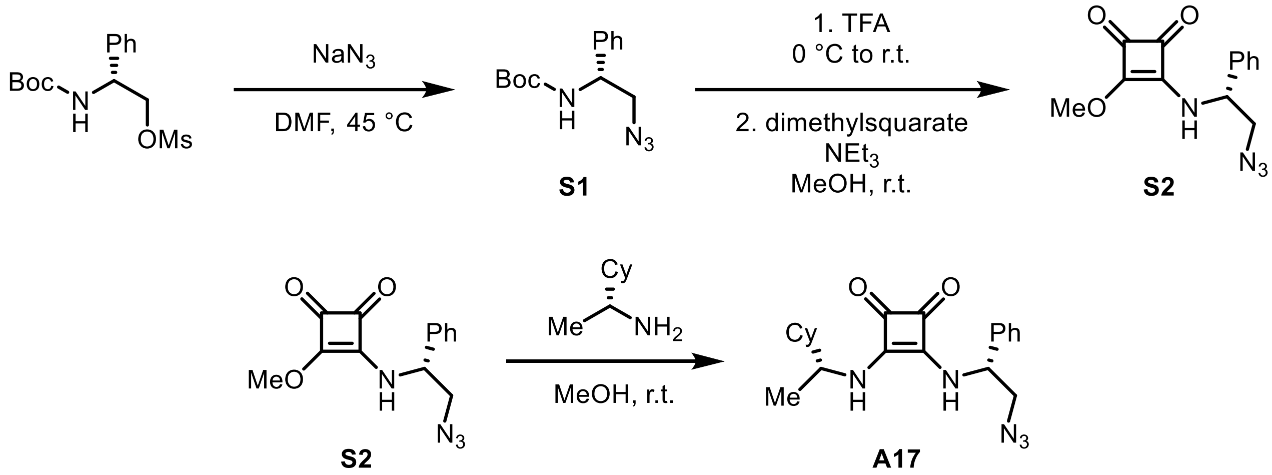

tert-butyl (*R*)-(2-azido-1-phenylethyl)carbamate (S1)

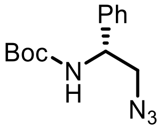

Compound **S1** was synthesised according to a modified literature procedure.[5] (*R*)-2-((tert-butoxycarbonyl)amino)-2-phenylethyl methanesulfonate (7.92 g, 25 mmol) was dissolved in DMF (75 mL). To the solution was added sodium azide (1.98 g, 30 mmol) and the solution was stirred behind a blast shield at r.t for 16 h. Once complete, LiCl (aq., 20%, 170 mL) was added, the aqueous phase was extracted with Et_2O (4 × 100 mL), the combined organic layers were washed with brine (100 mL), dried over $MgSO_4$ and concentrated *in vacuo*. The crude residue was purified by FCC (pentane/EtOAc 10% to 25%) to afford **S1** as a white solid (4.63 g, 17.7 mmol, 70%).

^{1}H NMR (400 MHz, $CDCl_3$) δ 7.40 – 7.35 (m, 2H), 7.31 (td, J = 5.9, 1.5 Hz, 3H), 5.04 (s, 1H), 4.87 (s, 1H), 3.63 (t, J = 5.6 Hz, 2H), 1.44 (s, 9H).

^{13}C NMR (101 MHz, $CDCl_3$) δ 155.2, 129.0, 128.2, 126.7, 80.3, 55.8, 54.3, 28.5.

HRMS (ESI) calcd. for $[M+Na]^+$ $(C_{13}H_{18}N_4O_2Na)^+$ 285.1322, found 285.1327.

$[\alpha]_D^{20}$ = 12.8 (c = 0.99, $CHCl_3$).

The data is consistent with the literature.[5]

[back to table of contents]

(*R*)-3-((2-azido-1-phenylethyl)amino)-4-methoxycyclobut-3-ene-1,2-dione (S2)

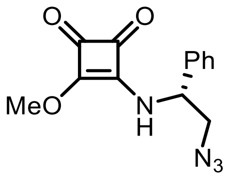

Compound **S2** was synthesised according to a literature procedure.[5] To an ROUND-BOTTOM FLASK containing compound **S1** (3.93 g, 15.0 mmol) at 0 °C behind a blast shield was added neat TFA (15 mL). The reaction mixture was allowed to warm to r.t. and was stirred for 2 hours. The TFA was removed under a stream of N_2, and then concentrated down further *in vacuo*. The residue was taken up in MeOH (200 mL), and added dropwise via a dropping funnel over 2 hours to a three-neck ROUND-BOTTOM FLASK containing dimethyl squarate (4.26 g, 30 mmol) and NEt_3 (15 mL, 105 mmol) dissolved in MeOH (75 mL). The reaction mixture was stirred at r.t. for 4 hours, before being concentrated *in vacuo*. Purification by FCC (CH_2Cl_2 to DCM/MeCN 10%) afforded **S2** as a viscous yellow oil (4.03 g, 14.8 mmol, 99%).

^{1}H NMR (400 MHz, $CDCl_3$) δ 7.97 (s, 1H), 7.46 – 7.30 (m, 5H), 4.87 (s, 1H), 4.40 (s, 3H), 3.89 – 3.78 (m, 1H), 3.68 (s, 1H).

^{13}C NMR (101 MHz, $CDCl_3$) δ 190.2, 182.8, 178.8, 171.7, 137.9, 129.3, 128.9, 126.6, 61.0, 59.3, 55.5.

HRMS (ESI) calcd. for $[M+K]^+$ $(C_{13}H_{12}N_4O_3K)^+$ 311.0541, found 311.0555.

$[\alpha]_D^{20}$ = -64.2 (c = 1.01, $CHCl_3$).

The data is consistent with the literature.[5]

3-(((*R*)-2-azido-1-phenylethyl)amino)-4-(((*R*)-1-cyclohexylethyl)amino)cyclobut-3- ene-1,2-dione (A17)

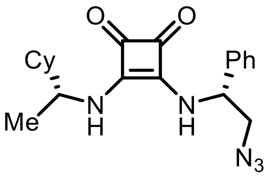

Compound **S2** (2.73 g, 10.0 mmol) was dissolved in MeOH (50 mL). To the resultant solution was added *R*-(-)-1-cyclohexylethylamine (1.50 mL, 10.0 mmol), and the reaction mixture was stirred at r.t for 2 h. Following this, the reaction mixture was filtered under reduced pressure, and the precipitate was washed with ice cold MeOH to afford azide **A17**, which was used without further purification (2.51 g, 6.83 mmol, 68%).

^{1}H NMR (600 MHz, [D_6]-DMSO) δ 7.83 (s, 1H), 7.47 – 7.38 (m, 4H), 7.37 – 7.30 (m, 2H), 5.32 (s, 1H), 3.94 – 3.87 (m, 1H), 3.83 (d, *J* = 6.9 Hz, 2H), 1.76 – 1.64 (m, 4H), 1.63 – 1.57 (m, 1H), 1.35 (m, 1H), 1.24 – 1.03 (m, 6H), 0.99 – 0.88 (m, 2H).

^{13}C NMR (151 MHz, [D_6]-DMSO) δ 182.6, 181.8, 167.7, 166.6, 139.2, 128.8, 128.0, 126.6, 56.5, 55.2, 54.1, 43.3, 28.5, 28.0, 25.9, 25.6, 18.9.

HRMS (ESI) calcd. for $[M+H]^+$ $(C_{20}H_{26}N_5O_2)^+$ 368.2081, found 368.2085.

FT-IR (thin film): ν_{max} (cm^{-1}) = 3166, 2934, 2088, 1640, 1556, 1476, 1250, 759, 697.

$[\alpha]_D^{20}$ = -7.33 (c = 0.99, DMSO).

[back to table of contents]

Synthesis of Unsubstituted Pyrrolidine and Piperidine Precursors

General Procedure 2

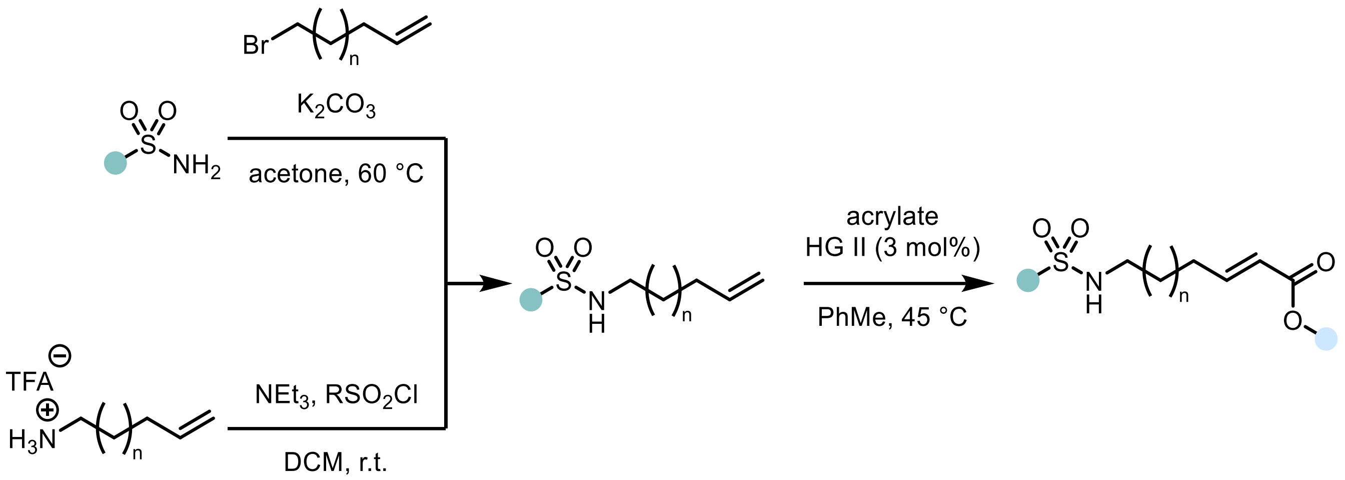

General Procedure 2A To a solution of the appropriate sulfonamide (1 equiv.) in acetone (1 M) was added K_2CO_3 (2 equiv.) and the alkyl bromide (1.1 equiv.). The reaction mixture was stirred at 60 °C for 18 h, after which the reaction mixture was filtered, the filter pad rinsed with DCM, and concentrated *in vacuo*. The *N*-alkylated sulfonamides were obtained as described in the specific experiment.

General Procedure 2B To a solution of the appropriate amine (1 equiv.) and triethylamine (3 equiv.) in DCM (0.3 M) at 0 °C was added corresponding sulfonyl chloride (1.5 equiv.). The reaction mixture was allowed to warm to room temperature overnight. Once complete, the reaction was quenched with H_2O, extracted with CH_2Cl_2 three times and washed with brine. The combined organics were dried over Na_2SO_4, filtered, and concentrated *in vacuo*. The crude residue was purified by FCC according to the specific experiment to give pure sulfonamide.

N-(pent-4-en-1-yl)benzenesulfonamide (S3)

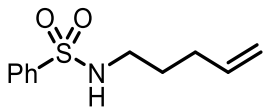

Alkene **S3** was prepared according to **General Procedure 2A** from benzenesulfonamide (3.16 g, 20.0 mmol) and 5-bromopent-1- ene (2.60 mL, 22.0 mmol) and was purified by FCC (pentane/EtOAc 10% to 30%) to afford **S3** as a pale yellow oil (1.10 g, 4.88 mmol, 24%).

^{1}H NMR (400 MHz, $CDCl_3$) δ 7.91 – 7.84 (m, 2H), 7.59 – 7.53 (m, 1H), 7.53 – 7.45 (m, 2H), 5.67 (ddt, J = 17.0, 10.3, 6.7 Hz, 1H), 5.10 (t, J = 6.2 Hz, 1H), 4.98 – 4.87 (m, 2H), 2.98 – 2.89 (m, 2H), 2.07 – 1.96 (m, 2H), 1.54 (p, J = 7.2 Hz, 2H).

^{13}C NMR (101 MHz, $CDCl_3$) δ 140.0, 137.3, 132.6, 129.2, 127.1, 115.6, 42.7, 30.6, 28.7.

HRMS (ESI) calcd. for $[M+Na]^+$ $(C_{11}H_{15}NO_2SNa)^+$ 248.0716, found 248.0723.

The data is consistent with the literature.[6]

[back to table of contents]

methyl (*E*)-6-(phenylsulfonamido)hex-2-enoate (7a)

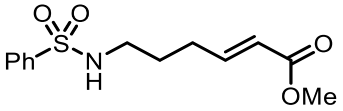

Compound **1a** was prepared according to **General Procedure 1** from compound **S3** (905.6 mg, 4.00 mmol) and was purified by FCC (pentane/EtOAc 20% to 40%) to afford **7a** as a dark red oil (1.00 g, 3.53 mmol, 88%).

^{1}H NMR (400 MHz, $CDCl_3$) δ 7.89 – 7.83 (m, 2H), 7.61 – 7.55 (m, 1H), 7.54 – 7.47 (m, 2H), 6.83 (dt, *J* = 15.6, 6.9 Hz, 1H), 5.76 (dt, *J* = 15.7, 1.6 Hz, 1H), 4.96 (t, *J* = 6.3 Hz, 1H), 3.70 (s, 3H), 2.96 (q, *J* = 6.8 Hz, 2H), 2.23 – 2.16 (m, 2H), 1.68 – 1.58 (m, 2H).

^{13}C NMR (101 MHz, $CDCl_3$) δ 167.0, 147.7, 140.0, 132.8, 129.3, 127.1, 121.9, 51.6, 42.6, 29.1, 28.1.

HRMS (ESI) calcd. for $[M+H]^+$ $(C_{13}H_{18}NO_4S)^+$ 284.0951, found 284.0960.

FT-IR (thin film): ν_{max} (cm^{-1}) = 3280, 2950, 1722, 1657, 1447, 1328, 1288, 1161, 1095, 757, 722, 691.

phenylmethanesulfonamide (S4)

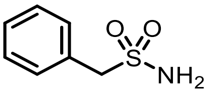

Sulfonamide **S4** was prepared according to the modified literature procedure.[7] α-toluenesulfonyl chloride (5.02 g, 26.3 mmol) was dissolved in THF (0.75 M). To this solution was added aqueous NH_4OH (35% v/v, 35 mL), and the reaction mixture was stirred at room temperature for 48 h. Once complete, the mixture was diluted with water (50 mL), extracted with EtOAc (3 × 100 mL), and washed with brine (3 × 100 mL) to afford **S4** (3.42 g, 20.0 mmol, 76%) as a white solid which was used in subsequent steps without further purification.

^{1}H NMR (400 MHz, $CDCl_3$) δ 7.47 – 7.34 (m, 5H), 4.70 (s, 2H), 4.30 (s, 2H).

^{13}C NMR (101 MHz, $CDCl_3$) δ 130.9, 129.5, 129.1, 61.1.

HRMS (ESI) calcd. for $[M+Na]^+$ $(C_7H_9NO_2SNa)^+$ 194.0246, found 194.0247.

The data is consistent with the literature.[7]

[back to table of contents]

_N_-(pent-4-en-1-yl)-1-phenylmethanesulfonamide (S5)

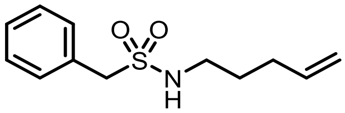

Alkene **S5** was prepared according to **General Procedure 2A** from sulfonamide **S4** (2.59 g, 15.0 mmol) and 5-bromopent-1-ene (2.00 mL, 16.5 mmol) and was purified by FCC (pentane/EtOAc 20% to 30%) to afford **S5** as an off white solid (1.05 g, 4.39 mmol, 29%).

^{1}H NMR (400 MHz, $CDCl_3$) δ 7.38 (s, 5H), 5.74 (ddt, *J* = 16.9, 10.2, 6.7 Hz, 1H), 5.06 – 4.95 (m, 2H), 4.24 (s, 2H), 4.16 (t, *J* = 6.2 Hz, 1H), 3.03 – 2.95 (m, 2H), 2.09 – 2.00 (m, 2H), 1.58 (p, *J* = 7.2 Hz, 2H).

^{13}C NMR (101 MHz, $CDCl_3$) δ 137.3, 130.7, 129.6, 129.0, 128.9, 115.8, 58.8, 43.3, 30.7, 29.6.

HRMS (ESI) calcd. for $[M+H]^+$ $(C_{12}H_{18}NO_2S)^+$ 240.1053, found 240.1049.

FT-IR (thin film): ν_{max} (cm^{-1}) = 3233, 2981, 2104, 1440, 1305, 1136, 1075, 911, 782, 740, 695.

M.P. 68-70 °C.

ethyl (_E_)-6-((phenylmethyl)sulfonamido)hex-2-enoate (7b)

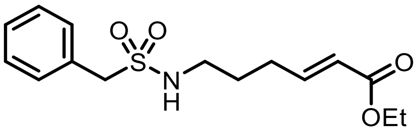

Compound **7b** was prepared according to **General Procedure 1** from compound **S5** (120 mg, 0.50 mmol) and ethyl acrylate (0.60 mL, 5.00 mmol), and was purified by FCC (pentane/EtOAc 15% to 30%) to afford **7b** as a dark red oil (144 mg, 0.46 mmol, 93%).

^{1}H NMR (600 MHz, $CDCl_3$) δ 7.39 (s, 5H), 6.86 (dt, *J* = 15.7, 6.9 Hz, 1H), 5.80 (dt, *J* = 15.6, 1.6 Hz, 1H), 4.25 (s, 2H), 4.19 (q, *J* = 7.1 Hz, 2H), 4.09 (t, *J* = 6.3 Hz, 1H), 2.98 (q, *J* = 6.6 Hz, 2H), 2.23 – 2.17 (m, 2H), 1.66 – 1.59 (m, 2H), 1.29 (t, *J* = 7.1 Hz, 3H).

^{13}C NMR (151 MHz, $CDCl_3$) δ 166.5, 147.2, 130.7, 129.5, 129.1, 129.0, 122.5, 60.5, 59.0, 43.2, 29.1, 29.0, 14.4.

HRMS (ESI) calcd. for $[M+Na]^+$ $(C_{15}H_{21}NO_4SNa)^+$ 334.1084, found 334.1084.

FT-IR (thin film) ν_{max} (cm^{-1}) = 3282, 2981, 1713, 1652, 1322, 1273, 1153, 1126, 1042, 781, 698.

[back to table of contents]

tert-butyl (E)-6-(phenylsulfonamido)hex-2-enoate (7c)

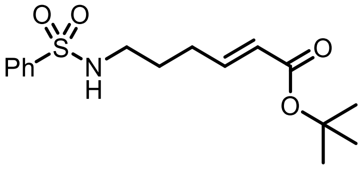

Compound **7c** was prepared according to **General Procedure 1**, from compound **S3** (338 mg, 1.5 mmol) and was purified by FCC (pentane/EtOAc 20% to 50%) to afford **7c** (404 mg, 1.24 mmol, 83%) as a bronze-brown oil.

^{1}H NMR (600 MHz, $CDCl_3$) δ 7.88 – 7.84 (m, 2H), 7.61 – 7.57 (m, 1H), 7.55 – 7.50 (m, 2H), 6.73 (dt, *J* = 15.6, 6.9 Hz, 1H), 5.68 (dt, *J* = 15.6, 1.6 Hz, 1H), 4.55 (t, *J* = 6.3 Hz, 1H), 2.98 (q, *J* = 6.8 Hz, 2H), 2.17 (qd, *J* = 7.0, 1.6 Hz, 2H), 1.63 (p, *J* = 7.2 Hz, 2H), 1.47 (s, 9H).

^{13}C NMR (151 MHz, $CDCl_3$) δ 165.9, 145.9, 140.1, 132.9, 129.3, 127.2, 124.2, 80.4, 42.7, 29.0, 28.3, 28.3.

HRMS (ESI) calcd. for $[M+Na]^+$ $(C_{16}H_{23}NO_4SNa)^+$ 348.1240, found 348.1242.

FT-IR (thin film): ν_{max} (cm^{-1}) = 3235, 1806, 1704, 1599, 1378, 1329, 1158, 739.

(E)-N,N-dimethyl-6-(phenylsulfonamido)hex-2-enamide (7d)

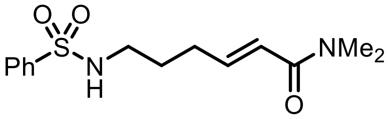

In a flame dried flask under nitrogen, alkene **S3** (113 mg, 0.5 mmol) and *N*,*N*-dimethylacrylamide (0.2 mL, 1.94 mmol) were dissolved in PhMe (1 M). To this solution was added a solution of HG-II (32 mg, 10 mol%) in PhMe (3 mL). The reaction mixture was stirred at 60 °C for 48 hours, following which it was cooled to room temperature and loaded directly onto a column of silica gel. The crude mixture was purified by FCC (petroleum ether/EtOAc 50%-100%) to afford **7d** as a dark brown oil (54.4 mg, 0.18 mmol, 37%)

^{1}H NMR (400 MHz, $CDCl_3$) δ 7.85 (d, *J* = 7.6 Hz, 2H), 7.56 (t, *J* = 7.3 Hz, 1H), 7.50 (t, *J* = 7.4 Hz, 2H), 6.72 (dt, *J* = 14.5, 7.1 Hz, 1H), 6.25 (d, *J* = 15.1 Hz, 1H), 5.15 (t, *J* = 6.0 Hz, 1H), 3.04 (s, 3H), 2.97 (s, 3H), 2.99 – 2.91 (m, 2H), 2.22 (q, *J* = 7.2 Hz, 2H), 1.62 (p, *J* = 7.1 Hz, 2H).

^{13}C NMR (101 MHz, $CDCl_3$) δ 166.7, 144.3, 140.2, 132.7, 129.2, 127.1, 121.5, 42.6, 37.5, 35.8, 29.3, 28.5.

HRMS (ESI) calcd. for $[M+H]^+$ $(C_{14}H_{21}N_2O_3S)^+$ 297.1267, found 297.1272.

FT-IR (thin film) ν_{max} (cm^{-1}) = 2939, 2849, 1660, 1606, 1329, 1161, 1092, 1048, 871, 692.

[back to table of contents]

_N_-(hex-5-en-1-yl)benzenesulfonamide (S6)

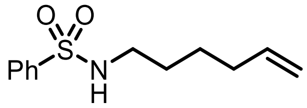

Alkene **S6** was prepared according to **General Procedure 2A**, using phenylsulfonamide (4.71 g, 30 mmol) and 6-bromo-1-hexene (4.00 mL, 30 mmol). After 18 h, H_2O (100 mL) was added to the reaction mixture and the aqueous layer was extracted with EtOAc (3 × 60 mL). The combined organic layers were washed with brine (3 × 80 mL), dried over $MgSO_4$, filtered and concentrated *in vacuo*. Purification by FCC (pentane/EtOAc 20% to 40%) afforded **S6** as a colourless oil (15.1 mmol, 50%).

^{1}H NMR (400 MHz, CDCl3) δ 7.90 – 7.84 (m, 2H), 7.61 – 7.47 (m, 3H), 5.70 (ddt, J = 16.9, 10.2, 6.7 Hz, 1H), 4.99 – 4.88 (m, 2H), 4.70 – 4.59 (m, 1H), 3.00 – 2.90 (m, 2H), 2.02 – 1.92 (m, 2H), 1.52 – 1.41 (m, 2H), 1.41 – 1.30 (m, 2H).

^{13}C NMR (101 MHz, CDCl3) δ 140.1, 138.2, 132.7, 129.2, 127.2, 115.1, 43.2, 33.2, 29.1, 25.8.

The data is consistent with the literature.[8]

methyl (*E*)-7-(phenylsulfonamido)hept-2-enoate (7e)

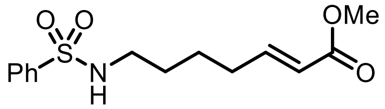

Compound **7e** was prepared according to **General Procedure 1** from compound **S6** (2.54 g, 10.6 mmol). Purification by FCC (pentane/EtOAc 20% to 60%) afforded **7e** (7.5 mmol, 71%) as a pale bronze solid.

^{1}H NMR (400 MHz, $CDCl_3$) δ 7.89 – 7.84 (m, 2H), 7.62 – 7.56 (m, 1H), 7.55 – 7.49 (m, 2H), 6.87 (dt, *J* = 15.7, 7.0 Hz, 1H), 5.77 (dt, *J* = 15.7, 1.6 Hz, 1H), 4.51 (t, *J* = 6.2 Hz, 1H), 3.72 (s, 3H), 2.97 (q, *J* = 6.5 Hz, 2H), 2.19 – 2.11 (m, 2H), 1.54 – 1.39 (m, 4H).

^{13}C NMR (101 MHz, $CDCl_3$) δ 167.1, 148.6, 140.1, 132.8, 129.3, 127.2, 121.6, 51.6, 43.0, 31.6, 29.2, 25.0.

HRMS (ESI) calcd. for $[M+H]^+$ $(C_{14}H_{20}NO_4S)^+$ requires m/z 298.1108, found m/z 298.1101.

FT-IR (thin film): ν_{max} (cm^{-1}) = 3288, 2950, 2866, 1717, 1650, 1320, 1160, 1095, 772, 721, 688.

M.P. 35-37 °C.

[back to table of contents]

methyl (*E*)-6-((phenylmethyl)sulfonamido)hex-2-enoate (7f)

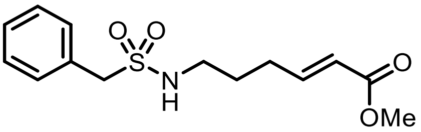

Compound **7f** was prepared according to **General Procedure 1** from compound **S5** (568 mg, 2.50 mmol) and was purified by FCC (pentane/EtOAc 20% to 30%) to afford **7f** as an off white solid (583 mg, 1.96 mmol, 83%).

^{1}H NMR (400 MHz, $CDCl_3$) δ 7.39 (s, 5H), 6.87 (dt, *J* = 15.6, 6.9 Hz, 1H), 5.81 (dt, *J* = 15.6, 1.6 Hz, 1H), 4.25 (s, 2H), 4.08 (t, *J* = 6.2 Hz, 1H), 3.73 (s, 3H), 3.02 – 2.94 (m, 2H), 2.24 – 2.17 (m, 2H), 1.67 – 1.58 (m, 2H).

^{13}C NMR (101 MHz, $CDCl_3$) δ 166.9, 147.6, 130.7, 129.5, 129.1, 129.0, 122.1, 59.0, 51.7, 43.2, 29.1, 29.0.

HRMS (ESI) calcd. for $[M+H]^+$ $(C_{14}H_{20}NO_4S)^+$ 298.1108, found 298.1099.

FT-IR (thin film): ν_{max} (cm^{-1}) = 3227, 2956, 2875, 2360, 2341, 1722, 1655, 1440, 1304, 1134, 784, 700.

M.P. 51-53 °C.

pent-4-en-1-aminium trifluoroacetate (S7)

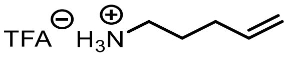

To a solution of 5-bromo-1-pentene (1.54 mL, 13 mmol, 1 eq.) in DMF (43 mL, 0.3 M), behind a blast shield, under N_2, was added sodium azide (930 mg, 14.3 mmol, 1.1 equiv). The reaction mixture was warmed to 40 °C and stirred for 4 h. The reaction was quenched by addition of H_2O (150 mL)and the mixture was extracted with Et_2O (3 × 50 mL). The combined organic layer was washed with brine (2 × 100 mL), dried over $MgSO_4$ and filtered. The intermediate was carefully concentrated *in vacuo* in a round-bottom flask, until a solution of approximate concentration 0.2 M was achieved (note – the azide was not isolated due to potential volatility and explosivity). The round-bottom flask was then charged with a stirrer bar and PPh_3 (4.1 g, 15.6 mmol, 1.2 eq.) and the reaction mixture was stirred at r.t. for 18 h. After this time 2M HCl (50 mL) was then added, and the reaction mixture left for 5 min under stirring. The aqueous layer was extracted and washed with EtOAc (3 × 100 mL) before being re-basified to pH 12 with 6M NaOH (50 mL). The mixture was extracted with Et_2O (3 × 75 mL). The organic layer was washed with brine (2 × 75 mL), dried over $MgSO_4$ and filtered into a round bottom flask fitted with a stirrer bar. To this solution was then added TFA (1.09 mL, 14.3 mmol, 1.1 eq.). After 15 min of stirring, the reaction mixture was concentrated *in vacuo* yielding the TFA salt of pent-4-en-1- amine **S7** as a colourless oil (10.6 mmol, 82%). The crude product was used in subsequent steps without purification.

[back to table of contents]

_N_-(pent-4-en-1-yl)pyridine-3-sulfonamide (S8)

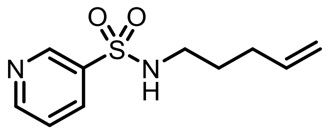

Compound **S8** was synthesised according to **General Procedure 2B** from the crude ammonium salt **S7** (199mg, 1.00 mmol) and 3-pyridine sulfonyl chloride (266 mg, 1.50 mmol) and was purified by FCC (pentane/EtOAc 50%) to afford **S8** as a brown oil (105 mg, 460 µmol, 46%).

^{1}H NMR (400 MHz, $CDCl_3$) δ 9.07 (dd, *J* = 2.4, 1.1 Hz, 1H), 8.80 (dt, *J* = 4.8, 1.3 Hz, 1H), 8.18 – 8.12 (m, 1H), 7.47 (ddd, *J* = 8.0, 4.8, 0.8 Hz, 1H), 5.70 (ddtd, *J* = 17.1, 10.4, 6.7, 1.1 Hz, 1H), 5.07 – 4.86 (m, 3H), 3.07 – 2.97 (m, 2H), 2.10 – 2.01 (m, 2H), 1.60 (p, *J* = 7.0 Hz, 2H).

^{13}C NMR (101 MHz, $CDCl_3$) δ 153.3, 148.1, 137.1, 137.0, 134.9, 123.9, 116.0, 42.8, 30.7, 28.8.

HRMS (ESI) calcd. for $[M+Na]^+$ $(C_{10}H_{14}N_2O_2SNa)^+$ 249.0673, found 249.0673.

methyl (*E*)-6-(pyridine-3-sulfonamido)hex-2-enoate (7g)

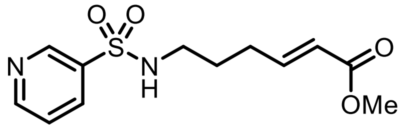

Compound **7g** was synthesised according to **General Procedure 1** from compound **S8** (90 mg, 0.400 mmol) and was purified by FCC (pentane/EtOAc 20%) to afford **7g** as a brown oil (34 mg, 120 µmol, 30%).

^{1}H NMR (400 MHz, $CDCl_3$) δ 9.09 (s, 1H), 8.83 (s, 1H), 8.17 – 8.13 (m, 1H), 7.49 (dd, *J* = 8.1, 4.8 Hz, 1H), 6.86 (dt, *J* = 15.7, 6.9 Hz, 1H), 5.80 (dt, *J* = 15.6, 1.6 Hz, 1H), 4.67 (t, *J* = 6.2 Hz, 1H), 3.72 (s, 3H), 3.09 – 2.99 (m, 2H), 2.24 (qd, *J* = 7.1, 1.6 Hz, 2H), 1.74 – 1.64 (m, 2H).

^{13}C NMR (101 MHz, $CDCl_3$) δ 167.0, 153.3, 148.0, 147.5, 136.9, 134.9, 124.0, 122.0, 51.7, 42.6, 29.0, 28.2.

HRMS (ESI) calcd. for $[M+H]^+$ $(C_{12}H_{17}N_2O_4S)^+$ 285.0904, found 285.0901.

_N_-(pent-4-en-1-yl)furan-2-sulfonamide (S9)

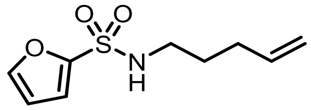

Compound **S9** was synthesised according to **General Procedure 2B** from crude ammonium salt **S7** (199 mg, 1.00 mmol) and furan-2-sulphonyl chloride (250 mg, 1.50 mmol) and was purified by FCC (pentane/EtOAc 85%) to afford **S9** as a yellow oil (105 mg, 460 µmol, 46%).

^{1}H NMR (400 MHz, $CDCl_3$) δ 7.55 (dd, *J* = 1.8, 0.9 Hz, 1H), 7.03 (dd, *J* = 3.5, 0.9 Hz, 1H), 6.50 (dd, *J* = 3.5, 1.8 Hz, 1H), 5.73 (ddt, *J* = 16.9, 10.2, 6.7 Hz, 1H), 5.04 – 4.95 (m, 2H), 4.67 (s, 1H), 3.10 – 3.03 (m, 2H), 2.10 – 2.01 (m, 2H), 1.63 – 1.54 (m, 3H).

^{13}C NMR (101 MHz, $CDCl_3$) δ 148.6, 146.1, 137.2, 116.5, 115.9, 111.3, 42.8, 30.7, 28.9.

HRMS (ESI) calcd. for $[M+Na]^+$ $(C_9H_{13}NO_3SNa)^+$ 238.0508, found 238.0518.

[back to table of contents]

methyl (*E*)-6-(furan-2-sulfonamido)hex-2-enoate (7h)

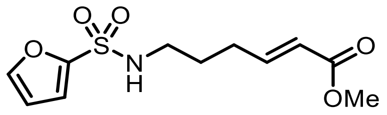

Compound **7h** was synthesised according to **General Procedure 1** from compound **S9** (86 mg, 0.400 mmol) and was purified by FCC (pentane/EtOAc 66%) to afford **7h** as a brown oil (89 mg, 320 μmol, 80%).

^{1}H NMR (400 MHz, $CDCl_3$) δ 7.57 – 7.54 (m, 1H), 7.04 (dt, *J* = 3.5, 0.8 Hz, 1H), 6.87 (dt, *J* = 15.7, 7.0 Hz, 1H), 6.51 (dd, *J* = 3.5, 1.8 Hz, 1H), 5.82 (dt, *J* = 15.6, 1.6 Hz, 1H), 4.79 – 4.71 (m, 1H), 3.72 (d, *J* = 0.6 Hz, 3H), 3.08 (q, *J* = 6.7 Hz, 2H), 2.28 – 2.20 (m, 2H), 1.67 (p, *J* = 7.2 Hz, 2H).

^{13}C NMR (101 MHz, $CDCl_3$) δ 166.9, 148.5, 147.5, 146.2, 122.1, 116.6, 111.4, 51.7, 42.7, 29.1, 28.2.

HRMS (ESI) calcd. for $[M+Na]^+$ $(C_{11}H_{15}NO_5SNa)^+$ 296.0563, found 296.0576.

***N*-(pent-4-en-1-yl)thiophene-2-sulfonamide (S10)**

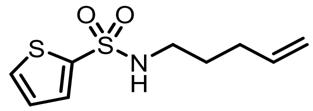

Compound **S10** was synthesised according to **General Procedure 2B** from crude ammonium salt **S7** (199 mg, 1.00 mmol) and 2-thiophenesulphonyl chloride (274 mg, 1.50 mmol) and was purified by FCC (pentane/EtOAc 85%) to afford **S10** as an orange oil (138 mg, 597 μmol, 60%).

^{1}H NMR (400 MHz, $CDCl_3$) δ 7.63 – 7.58 (m, 2H), 7.09 (dd, *J* = 5.0, 3.7 Hz, 1H), 5.72 (ddt, *J* = 17.0, 10.2, 6.7 Hz, 1H), 5.04 – 4.93 (m, 2H), 4.66 (s, 1H), 3.05 (q, *J* = 6.8 Hz, 2H), 2.12 – 2.04 (m, 2H), 1.61 (p, *J* = 7.2 Hz, 2H).

^{13}C NMR (101 MHz, $CDCl_3$) δ 141.1, 137.3, 132.2, 131.9, 127.5, 115.9, 43.1, 30.8, 28.7.

HRMS (ESI) calcd. for $[M+K]^+$ $(C_9H_{13}NO_2S_2Na)^+$ 270.0019, found 270.0009.

methyl (*E*)-6-(thiophene-2-sulfonamido)hex-2-enoate (7i)

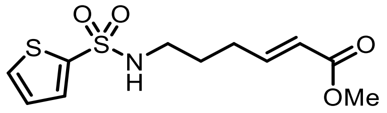

Compound **7i** was synthesised according to **General Procedure 1** from compound **S10** (92 mg, 0.400 mmol) and was purified by FCC (pentane/EtOAc 50%) to afford **7i** as a grey solid (116 mg, 400 μmol, quant.).

^{1}H NMR (400 MHz, $CDCl_3$) δ 7.62 – 7.57 (m, 2H), 7.09 (dd, *J* = 5.0, 3.8 Hz, 1H), 6.87 (dt, *J* = 15.7, 6.9 Hz, 1H), 5.80 (dt, *J* = 15.7, 1.6 Hz, 1H), 4.80 (s, 1H), 3.72 (s, 3H), 3.06 (q, *J* = 6.7 Hz, 2H), 2.28 – 2.20 (m, 2H), 1.73 – 1.63 (m, 2H).

^{13}C NMR (101 MHz, $CDCl_3$) δ 167.0, 147.6, 141.0, 132.3, 132.1, 127.6, 122.0, 51.7, 42.9, 29.1, 28.1.

HRMS (ESI) calcd. for $[M+Na]^+$ $(C_{11}H_{15}NO_4S_2Na)^+$ 312.0335, found 312.0349.

M.P. 60-62 °C.

[back to table of contents]

3,5-dimethyl-*N*-(pent-4-en-1-yl)isoxazole-4-sulfonamide (S11)

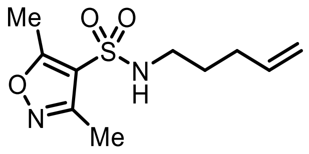

Compound **S11** was synthesised according to **General Procedure 2B** from crude ammonium salt **S7** (398 mg, 2.00 mmol) and 3,5-dimethylisoxazole-4-sulfonyl chloride (470 mg, 2.40 mmol) and was purified by FCC (pentane/EtOAc 75%) to afford **S11** as a yellow oil (105 mg, 460 µmol, 46%).

^{1}H NMR (400 MHz, $CDCl_3$) δ 5.73 (ddt, *J* = 16.2, 10.6, 6.7 Hz, 1H), 5.05 – 4.96 (m, 2H), 4.64 (t, *J* = 6.4 Hz, 1H), 3.03 – 2.94 (m, 2H), 2.63 (s, 3H), 2.40 (s, 3H), 2.13 – 2.04 (m, 2H), 1.62 (p, *J* = 7.2 Hz, 2H).

^{13}C NMR (101 MHz, $CDCl_3$) δ 173.3, 157.7, 137.0, 116.1, 42.4, 30.8, 28.7, 12.8, 10.9.

HRMS (ESI) calcd. for $[M+Na]^+$ $(C_{10}H_{16}N_2O_3SNa)^+$ 267.0774, found 267.0774.

methyl (*E*)-6-((3,5-dimethylisoxazole)-4-sulfonamido)hex-2-enoate (7j)

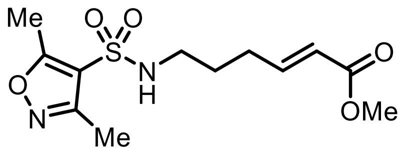

Compound **7j** was synthesised according to **General Procedure 1** from compound **S11** (272 mg, 0.116 mmol) and was purified by FCC (pentane/EtOAc 75%) to afford **7j** as a brown oil (105 mg, 38 µmol, 32%).

^{1}H NMR (400 MHz, $CDCl_3$) δ 6.87 (dt, *J* = 15.7, 6.9 Hz, 1H), 5.81 (dt, *J* = 15.7, 1.6 Hz, 1H), 5.07 (t, *J* = 6.2 Hz, 1H), 3.71 (s, 3H), 3.01 – 2.93 (m, 2H), 2.62 (s, 3H), 2.38 (s, 3H), 2.29 – 2.21 (m, 2H), 1.69 (p, *J* = 7.2 Hz, 2H).

^{13}C NMR (101 MHz, $CDCl_3$) δ 173.3, 166.9, 157.6, 147.4, 122.1, 116.1, 51.7, 42.2, 29.1, 28.1, 12.8, 10.9.

HRMS (ESI) calcd. for $[M+Na]^+$ $(C_{12}H_{18}N_2O_5SNa)^+$ 325.0829, found 325.0823.

2,4,6-trimethyl-*N*-(pent-4-en-1-yl)benzenesulfonamide (S12)

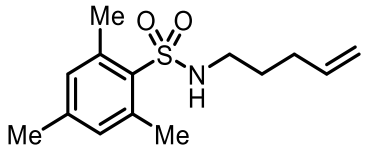

Compound **S12** was synthesised according to **General Procedure 2B** from crude ammonium salt **S7** (539 mg, 3.00 mmol) and 2,4,6-trimethylbenzenesulfonyl chloride (787 mg, 3.60 mmol) and was purified by FCC (pentane/EtOAc 90%) to afford **S12** as a white solid (227 mg, 850 µmol, 28%).

^{1}H NMR (400 MHz, $CDCl_3$) δ 6.97 – 6.94 (m, 2H), 5.69 (ddt, *J* = 16.2, 11.0, 6.7 Hz, 1H), 4.98 – 4.94 (m, 1H), 4.92 (t, *J* = 1.4 Hz, 1H), 4.57 (d, *J* = 7.1 Hz, 1H), 2.94 – 2.87 (m, 2H), 2.63 (s, 6H), 2.30 (s, 3H), 2.05 – 1.99 (m, 2H), 1.55 (p, *J* = 7.2 Hz, 2H).

^{13}C NMR (101 MHz, $CDCl_3$) δ 142.3, 139.2, 137.4, 133.8, 132.1, 115.7, 42.1, 30.9, 28.8, 23.1, 21.0.

HRMS (ESI) calcd. for $[M+K]^+$ $(C_{14}H_{21}NO_2SK)^+$ 306.0925, found 306.0934.

M.P. 80-82 °C.

[back to table of contents]

methyl (*E*)-6-((2,4,6-trimethylphenyl)sulfonamido)hex-2-enoate (7k)

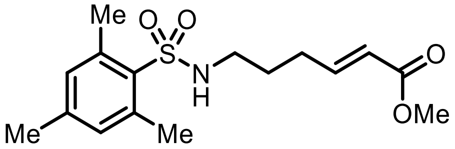

Compound **7k** was synthesised according to **General Procedure 1** from compound **S12** (133 mg, 0.500 mmol) and was purified by FCC (pentane/EtOAc 75%) to afford **7k** as a white solid (151 mg, 0.467 mmol, 94%).

^{1}H NMR (400 MHz, $CDCl_3$) δ 6.96 (s, 2H), 6.82 (dt, *J* = 15.7, 6.9 Hz, 1H), 5.72 (dt, *J* = 15.7, 1.6 Hz, 1H), 4.60 (s, 1H), 3.71 (s, 3H), 2.92 (q, *J* = 6.8 Hz, 2H), 2.63 (s, 6H), 2.30 (s, 3H), 2.22 – 2.11 (m, 2H), 1.61 (p, *J* = 7.1 Hz, 2H).

^{13}C NMR (101 MHz, $CDCl_3$) δ 166.9, 147.7, 142.4, 139.2, 133.7, 132.2, 121.9, 51.6, 42.0, 29.2, 28.2, 23.1, 21.1.

HRMS (ESI) calcd. for $[M+H]^+$ $(C_{16}H_{24}NO_4S)^+$ 326.1421, found 326.1419.

M.P. 86-88 °C.

2,4,6-triisopropyl-*N*-(pent-4-en-1-yl)benzenesulfonamide (S13)

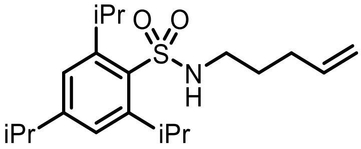

Compound **S13** was synthesised according to **General Procedure 2B** from compound **S7** (539 mg, 3.00 mmol) and 2,4,6-triisopropylbenzenesulfonyl chloride (1.08 g, 3.60 mmol) and was purified by FCC (pentane/EtOAc 90%) to afford **S13** as a white solid (437 mg, 1.24 mmol, 41%).

^{1}H NMR (400 MHz, $CDCl_3$) δ 7.16 (s, 2H), 5.71 (ddt, *J* = 15.9, 10.6, 6.6 Hz, 1H), 4.99 – 4.95 (m, 1H), 4.93 (t, *J* = 1.4 Hz, 1H), 4.41 – 4.27 (m, 1H), 4.16 (hept, *J* = 6.8 Hz, 2H), 3.02 – 2.95 (m, 2H), 2.90 (p, *J* = 6.9 Hz, 1H), 2.10 – 2.01 (m, 2H), 1.59 (p, *J* = 7.3 Hz, 2H), 1.26 (dd, *J* = 6.8, 4.9 Hz, 18H).

^{13}C NMR (101 MHz, $CDCl_3$) δ 152.8, 150.4, 137.4, 132.4, 123.9, 115.7, 42.4, 34.3, 30.9, 29.8, 29.0, 25.0, 23.7.

HRMS (ESI) calcd. for $[M+H]^+$ $(C_{20}H_{34}NO_2S)^+$ 352.2305, found 352.2310.

M.P. 84-86 °C.

methyl (*E*)-6-((2,4,6-triisopropylphenyl)sulfonamido)hex-2-enoate (71)

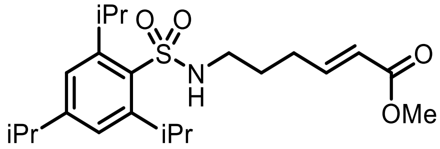

Compound **71** was synthesised according to **General Procedure 1** from compound **S13** (175 mg, 0.500 mmol) was purified by FCC (pentane/EtOAc 75%) to afford **71** as a white solid (270 mg, 0.488 mmol, 98%).

^{1}H NMR (400 MHz, $CDCl_3$) δ 7.16 (s, 2H), 6.85 (dt, *J* = 15.7, 7.0 Hz, 1H), 5.76 (dt, *J* = 15.7, 1.6 Hz, 1H), 4.54 (t, *J* = 6.1 Hz, 1H), 4.14 (hept, *J* = 6.8 Hz, 2H), 3.70 (s, 3H), 3.02 – 2.95 (m, 2H), 2.89 (p, *J* = 6.9 Hz, 1H), 2.27 – 2.15 (m, 2H), 1.65 (p, *J* = 7.2 Hz, 2H), 1.27 – 1.24 (m, 18H).

^{13}C NMR (101 MHz, $CDCl_3$) δ 166.9, 152.9, 150.3, 147.7, 132.4, 123.9, 121.9, 51.6, 42.3, 34.2, 29.7, 29.3, 28.3, 25.0, 23.7.

HRMS (ESI) calcd. for $[M+Na]^+$ $(C_{22}H_{35}NO_4SNa)^+$ 432.2179, found 432.2180.

M.P. 94-96 °C.

***N*-(pent-4-en-1-yl)-5-(5-(trifluoromethyl)isoxazol-3-yl)thiophene-2-sulfonamide (S14)**

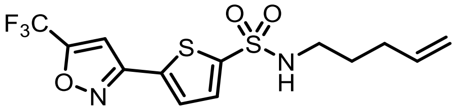

Compound **S14** was synthesised according to **General Procedure 2B** from crude ammonium salt **S7** (104 mg, 0.580 mmol) and 5-(5-(trifluoromethyl)isoxazol-3-yl)thiophene-2-sulfonyl chloride (221 mg, 0.696 mmol) and was purified by FCC (pentane/EtOAc 75%) to afford **S14** as a white solid (150 mg, 410 μmol, 71%).

^{1}H NMR (400 MHz, $CDCl_3$) δ 7.61 (d, *J* = 3.9 Hz, 1H), 7.46 (d, *J* = 3.9 Hz, 1H), 6.99 – 6.97 (m, 1H), 5.74 (ddt, *J* = 17.0, 10.2, 6.7 Hz, 1H), 5.06 – 4.96 (m, 2H), 4.74 (t, *J* = 6.1 Hz, 1H), 3.12 (td, *J* = 7.1, 6.2 Hz, 2H), 2.14 – 2.06 (m, 2H), 1.65 (p, *J* = 7.2 Hz, 2H).

^{13}C NMR (101 MHz, $CDCl_3$) δ 156.93, 144.24, 137.11, 134.52, 132.19, 128.27, 116.06, 103.61 (q, *J* = 1.9 Hz), 43.18, 30.77, 28.75.

^{19}F NMR (376 MHz, $CDCl_3$) δ -64.24 (s, 3F).

HRMS (ESI) calcd. for $[M+H]^+$ $(C_{13}H_{14}F_3N_2O_3S_2)^+$ 367.0392, found 367.0389.

M.P. 118-119 °C

methyl (*E*)-6-((5-(5-(trifluoromethyl)isoxazol-3-yl)thiophene)-2-sulfonamido)hex-2-enoate (7m)

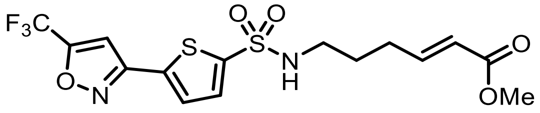

Compound **7m** was synthesised according to **General Procedure 1** from compound **S14** (119 mg, 0.325 mmol) and was purified by FCC (pentane/EtOAc 66%) to afford **7m** as a white solid (137 mg, 0.325 mmol, quant.).

^{1}H NMR (400 MHz, $CDCl_3$) δ 7.61 (d, *J* = 3.9 Hz, 1H), 7.46 (d, *J* = 3.9 Hz, 1H), 7.00 – 6.98 (m, 1H), 6.88 (dt, *J* = 15.6, 6.9 Hz, 1H), 5.82 (dt, *J* = 15.7, 1.6 Hz, 1H), 4.86 (t, *J* = 6.0 Hz, 1H), 3.71 (s, 3H), 3.12 (q, *J* = 6.8 Hz, 2H), 2.32 – 2.22 (m, 2H), 1.72 (p, *J* = 7.2 Hz, 2H).

^{13}C NMR (101 MHz, $CDCl_3$) δ 166.93, 156.89, 147.35, 144.03, 134.68, 132.31, 128.32, 122.21, 103.73 – 103.53 (m), 51.68, 42.97, 29.10, 28.14.

^{19}F NMR (376 MHz, $CDCl_3$) δ -64.25 (s, 3F).

HRMS (ESI) calcd. for $[M+H]^+$ $(C_{13}H_{14}F_3N_2O_3S_2)^+$ 425.0447, found 425.0447.

M.P. 94-96 °C.

Synthesis of *β*-Disubstituted Pyrrolidine and Piperidine Precursors

General Procedure 3

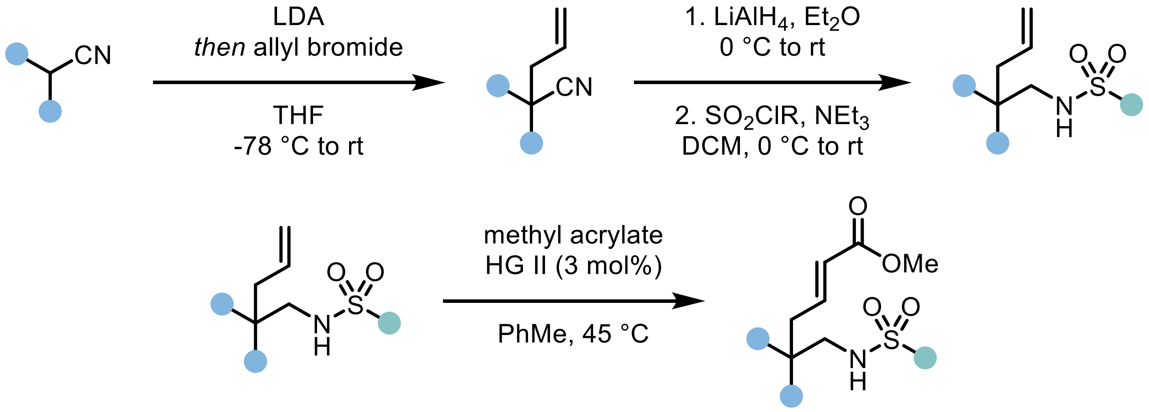

General Procedure 3A In a flame dried flask under a nitrogen atmosphere, a solution of diisopropylamine (1.25 equiv.) in THF (0.4 M) was cooled to -78 °C. To this solution was added *n*BuLi (2.5 M in hexanes, 1.2 equiv.) dropwise over 15 mins. After stirring for 30 mins, the appropriate nitrile (1 equiv.) was added dropwise over 15 mins while stirring at -78 °C. After stirring for a further 30 mins, allyl bromide (2 equiv.) was added dropwise over 10 mins, and the reaction mixture left to warm to room temperature overnight. Once complete, the reaction was quenched with an sat. aqueous NH_4Cl solution (50 mL), extracted with Et_2O (3 × 50 mL) and washed with brine (100 mL). The combined organics were dried over anhydrous Na_2SO_4, filtered, and concentrated *in vacuo*. The crude residue was purified by FCC according to the specific experiment to give the pure alkene.

General Procedure 3B To a solution of $LiAlH_4$ (1 M in Et_2O, 2 equiv.) at 0 °C under a nitrogen atmosphere was added a solution of the appropriate alkene (1 equiv.) in Et_2O (2 M) dropwise over 5 mins. The Reaction mixture was stirred at 0 °C and monitored by TLC. Once complete, the reaction was quenched with water (1 mL/g LAH), followed by 15% aq. NaOH (1 mL/g LAH) and water (3 mL/g LAH). The mixture was dried over anhydrous Na_2SO_4, filtered and concentrated *in vacuo*. The crude amine was used in the subsequent step without further purification.

In a flame dried flask under a nitrogen atmosphere, the appropriate crude amine (1 equiv.) was dissolved in CH_2Cl_2 (0.3 M). To this solution was added triethylamine (2 equiv.) and the solution cooled to 0 °C. To this solution was added phenylmethanesulfonyl chloride (1.5 equiv.) and the mixture allowed to warm to room temperature overnight. Once complete, the reaction was diluted with CH_2Cl_2 and quenched with saturated aqueous $NaHCO_3$. The crude mixture was extracted with CH_2Cl_2 (3 × 50 mL), washed with brine (100 mL), dried over anhydrous Na_2SO_4, filtered and concentrated *in vacuo*. The crude residue was purified by FCC according to the specific experiment to give the pure sulfonamide.

[back to table of contents]

1-allylcyclohexane-1-carbonitrile (S15)

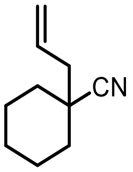

Compound **S15** was synthesised according to **General Procedure 3A** from cyclohexane carbonitrile (2.40 mL, 20.0 mmol) and purified by FCC (pentane/Et_2O 5%) to afford **S15** as a pale yellow oil (2.79 g, 18.7 mmol, 93%).

^{1}H NMR (400 MHz, $CDCl_3$) δ 5.87 (ddt, J = 17.3, 10.1, 7.4 Hz, 1H), 5.23 – 5.11 (m, 2H), 2.27 (d, J = 7.4 Hz, 2H), 1.99 – 1.89 (m, 2H), 1.78 – 1.57 (m, 5H), 1.31 – 1.18 (m, 3H).

^{13}C NMR (101 MHz, CDCl3) δ 132.1, 123.4, 119.7, 44.7, 39.0, 35.5, 25.4, 23.1.

HRMS (ESI) calcd. for $[M+H]^+$, $(C_{10}H_{16}N)^+$ 150.1277, found 150.1275.

The data is consistent with the literature.[9]

***N*-((1-allylcyclohexyl)methyl)-1-phenylmethanesulfonamide (S16)**

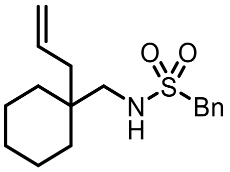

Compound **S16** was synthesised according to **General Procedure 3B** from compound **S15** (1.49 g, 10.0 mmol) and was purified by FCC (DCM to DCM/MeCN 25%) to afford **S16** as an off white solid (924 mg, 3.0 mmol 30% over 2 steps).

^{1}H NMR (600 MHz, $CDCl_3$) δ 7.39 (s, 5H), 5.74 (ddt, *J* = 17.5, 10.1, 7.5 Hz, 1H), 5.01 (ddt, *J* = 10.1, 2.1, 1.0 Hz, 1H), 4.96 (ddt, *J* = 17.0, 2.2, 1.4 Hz, 1H), 4.24 (s, 2H), 4.11 (t, *J* = 6.8 Hz, 1H), 2.88 (d, *J* = 6.8 Hz, 2H), 2.01 (dt, *J* = 7.5, 1.2 Hz, 2H), 1.48 – 1.35 (m, 6H), 1.31 – 1.27 (m, 4H).

^{13}C NMR (151 MHz, $CDCl_3$) δ 134.3, 130.6, 129.6, 129.0, 128.9, 118.0, 58.5, 50.0, 40.7, 36.8, 33.3, 26.1, 21.4.

HRMS (ESI) calcd. for $[M+H]^+$, $(C_{17}H_{26}NO_2S)^+$ 308.1679, found 308.1691.

FT-IR (thin film) ν_{max} (cm^{-1}) = 3295, 2928, 2859, 1455, 1328, 1155, 1127, 917, 698.

M.P. 78-80 °C.

[back to table of contents]

methyl (*E*)-4-(1-(((phenylmethyl)sulfonamido)methyl)cyclohexyl)but-2-enoate (7n)

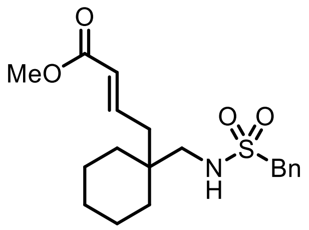

Compound **7n** was synthesised according to **General Procedure 1** from compound **S16** (502 mg, 1.63 mmol) and was purified by FCC (pentane/Et_2O 30%) to afford **7n** as a white solid (314 mg, 0.859 mmol, 53%).

^{1}H NMR (600 MHz, $CDCl_3$) δ 7.39 (s, 5H), 6.88 (dt, *J* = 15.7, 7.9 Hz, 1H), 5.82 (dt, *J* = 15.5, 1.4 Hz, 1H), 4.25 (s, 2H), 4.07 (t, *J* = 6.9 Hz, 1H), 3.75 (s, 3H), 2.86 (d, *J* = 6.9 Hz, 2H), 2.16 (dd, *J* = 7.9, 1.4 Hz, 2H), 1.49 – 1.34 (m, 6H), 1.33 – 1.27 (m, 4H).

^{13}C NMR (151 MHz, $CDCl_3$) δ 166.7, 144.9, 130.6, 129.5, 129.1, 129.0, 124.1, 58.7, 51.7, 50.0, 38.4, 37.6, 33.1, 25.9, 21.4.

HRMS (ESI) calcd. for $[M+H]^+$, $(C_{19}H_{28}NO_4S)^+$ 366.1734, found 366.1745.

FT-IR (thin film) ν_{max} (cm^{-1}) = 3284, 2931, 2860, 1720, 1496, 1456, 1329, 1155, 913, 735, 699.

M.P. 94-96 °C.

4-allyltetrahydro-2*H*-pyran-4-carbonitrile (S17)

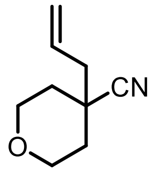

Compound **S17** was synthesised according to **General Procedure 3A** from 4-cyanotetrahydropyran (2.25 g, 20.0 mmol) and was purified by FCC (pentane/EtOAc 10%) to afford **S17** as a pale yellow oil (2.46 g, 16.3 mmol, 80%, 98 wt% purity calculated by qNMR).

^{1}H NMR (600 MHz, $CDCl_3$) δ 5.87 (ddt, *J* = 17.4, 10.2, 7.4 Hz, 1H), 5.24 (d, *J* = 10.1 Hz, 1H), 5.21 (dd, *J* = 16.8, 1.2 Hz, 1H), 3.97 – 3.90 (m, 2H), 3.69 (td, *J* = 12.2, 1.9 Hz, 2H), 2.32 (d, *J* = 7.4 Hz, 2H), 1.84 (dd, *J* = 13.7, 2.1 Hz, 2H), 1.58 (ddd, *J* = 13.8, 12.1, 4.5 Hz, 2H).

^{13}C NMR (151 MHz, $CDCl_3$) δ 131.0, 122.3, 120.6, 64.8, 44.5, 36.8, 35.2.

HRMS (ESI) calcd. for $[M+H]^+$, $(C_9H_{14}NO)^+$ 152.1070, found 152.1063.

FT-IR (thin film) ν_{max} (cm^{-1}) = 2958, 2926, 2854, 1446, 1243, 1107, 1061, 1034, 1015, 927.

[back to table of contents]

N-((4-allyltetrahydro-2H-pyran-4-yl)methyl)-1-phenylmethanesulfonamide (S18)

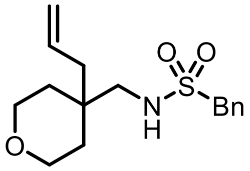

Compound **S18** was synthesised according to **General Procedure 3B** from compound **S17** (1.52 g, 10.0 mmol) and was purified by trituration in Et_2O to afford **S18** as an off white solid (1.36 g, 4.4 mmol, 44% over 2 steps).

^{1}H NMR (600 MHz, $CDCl_3$) δ 7.39 (s, 5H), 5.72 (ddt, *J* = 17.5, 10.1, 7.5 Hz, 1H), 5.06 (t, *J* = 10.1 Hz, 1H), 5.00 (dd, *J* = 17.0, 1.3 Hz, 1H), 4.25 (s, 3H), 3.63 (h, *J* = 7.4 Hz, 4H), 2.93 (d, *J* = 6.9 Hz, 2H), 2.10 (dt, *J* = 7.5, 1.2 Hz, 2H), 1.48 – 1.39 (m, 4H).

^{13}C NMR (151 MHz, $CDCl_3$) δ 133.3, 130.6, 129.5, 129.1, 129.0, 118.8, 63.4, 58.8, 49.2, 46.1, 40.4, 34.9, 33.2.

HRMS (ESI) calcd. for $[M+H]^+$, $(C_{16}H_{24}NO_3S)^+$ 310.1471, found 310.1459.

FT-IR (thin film) ν_{max} (cm^{-1}) = 3290, 2933, 2858, 1495, 1330, 1154, 1127, 1105, 1073, 916, 734, 699.

M.P. 126-128 °C.

methyl (*E*)-4-(4-(((phenylmethyl)sulfonamido)methyl)tetrahydro-2*H*-pyran-4-yl)- but-2-enoate (7o)

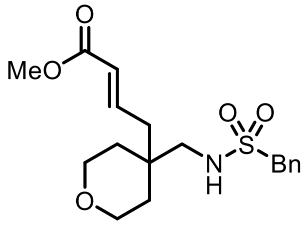

Compound **7o** was synthesised according to **General Procedure 1** from compound **S18** (503 mg, 1.62 mmol) and was purified by FCC (pentane/EtOAc 40%) followed by trituration in Et_2O to afford **7o** as an off white solid (230 mg, 0.626 mmol, 39%).

^{1}H NMR (600 MHz, $CDCl_3$) δ 7.43 – 7.35 (m, 5H), 6.88 – 6.81 (m, 1H), 5.84 (d, *J* = 15.6 Hz, 1H), 4.31 (t, *J* = 6.9 Hz, 1H), 4.26 (s, 2H), 3.75 (s, 3H), 3.62 (t, *J* = 5.5 Hz, 4H), 2.91 (d, *J* = 6.9 Hz, 2H), 2.28 – 2.23 (m, 2H), 1.50 – 1.38 (m, 4H).

^{13}C NMR (151 MHz, $CDCl_3$) δ 166.5, 143.7, 130.6, 129.3, 129.2, 129.1, 124.7, 63.2, 58.9, 51.8, 49.1, 38.0, 35.6, 33.0.

HRMS (ESI) calcd. for $[M+H]^+$ $(C_{18}H_{26}NO_5S)^+$ 368.1526, found 368.1527.

FT-IR (thin film) ν_{max} (cm^{-1}) = 3286, 2951, 1719, 1655, 1437, 1331, 1154, 1127, 915, 735, 699.

M.P. 94-96 °C.

[back to table of contents]

tert-butyl 4-allyl-4-cyanopiperidine-1-carboxylate (S19)

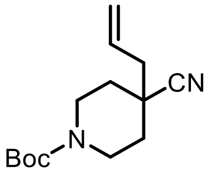

Compound **S19** was synthesised according to **General Procedure 3A** from 1-boc-4-cyanopiperidine (4.22 g, 20.0 mmol) and was purified by FCC (pentane/EtOAc 5% to 20%) to afford **S19** as a pale yellow oil (4.37 g, 17.5 mmol, 87%, contains approx. 8 wt% EtOAc by NMR).

^{1}H NMR (500 MHz, $CDCl_3$) δ 5.89 – 5.77 (m, 1H), 5.22 – 5.13 (m, 2H), 4.07 (s, 2H), 2.98 (s, 2H), 2.29 – 2.26 (m, 2H), 1.88 – 1.80 (m, 2H), 1.41 (d, *J* = 0.9 Hz, 9H), 1.40 – 1.33 (m, 2H).

^{13}C NMR (126 MHz, $CDCl_3$) δ 154.4, 131.0, 121.9, 120.5, 80.0, 44.0, 40.9, 37.7, 34.4, 28.4.

HRMS (ESI) calcd. for $[M+Na]^+$ $(C_{14}H_{22}N_2O_2Na)^+$ 273.1574, found 273.1571.

FT-IR (thin film) ν_{max} (cm^{-1}) = 2978, 1697, 1421, 1366, 1248, 1159, 973, 923.

tert-butyl 4-allyl-4-(((phenylmethyl)sulfonamido)methyl)piperidine-1-carboxylate (S20)

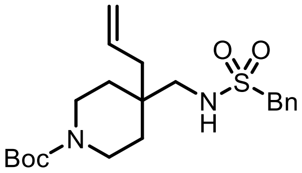

Compound **S20** was synthesised according to **General Procedure 3B** from compound **S19** (2.53 g, 10.0 mmol) and was purified by FCC (pentane/EtOAc 20% to 40%) to afford **S20** as an off white solid (294 mg, 0.72 mmol, 7% over 2 steps).

^{1}H NMR (600 MHz, $CDCl_3$) δ 7.39 (m, 5H), 5.71 (ddt, *J* = 17.5, 10.2, 7.5 Hz, 1H), 5.06 (d, *J* = 9.7 Hz, 1H), 4.99 (dd, *J* = 17.1, 1.7 Hz, 1H), 4.25 (s, 2H), 4.17 (t, *J* = 6.8 Hz, 1H), 3.36 (t, *J* = 5.9 Hz, 4H), 2.88 (d, *J* = 12.2 Hz, 2H), 2.05 (d, *J* = 7.5 Hz, 2H), 1.44 (s, 9H), 1.41 – 1.33 (m, 4H).

^{13}C NMR (151 MHz, $CDCl_3$) δ 155.0, 133.3, 130.6, 129.4, 129.1, 129.1, 118.9, 79.7, 58.8, 49.0, 40.0, 35.7, 32.4, 28.6.

HRMS (ESI) calcd. for $[M+Na]^+$ $(C_{21}H_{32}N_2O_4SNa)^+$ 431.1975, found 431.1981.

FT-IR (thin film) ν_{max} (cm^{-1}) = 3294, 2976, 1688, 1427, 1329, 1279, 1246, 1160, 917, 735.

M.P. 102-104 °C.

[back to table of contents]

tert-butyl (*E*)-4-(4-methoxy-4-oxobut-2-en-1-yl)-4-(((phenylmethyl)sulfonamido)-methyl)piperidine-1-carboxylate (7p)

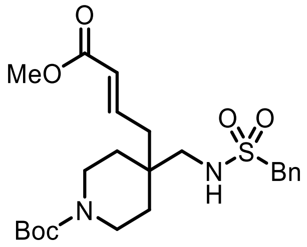

Compound **7p** was synthesised according to **General Procedure 1** from compound **S20** (154 mg, 0.370 mmol) and was purified by FCC (pentane/EtOAc 30% to 40%) to afford **7p** as an off white solid (93.0 mg, 0.199 mmol, 53%).

^{1}H NMR (600 MHz, $CDCl_3$) δ 7.39 (m, 5H), 6.84 (dt, *J* = 15.6, 7.9 Hz, 1H), 5.83 (dt, *J* = 15.5, 1.4 Hz, 1H), 4.27 (s, 2H), 4.08 (t, *J* = 7.0 Hz, 1H), 3.76 (s, 3H), 3.42 – 3.30 (m, 4H), 2.88 (s, 2H), 2.23 – 2.18 (m, 2H), 1.45 (s, 9H), 1.43 – 1.36 (m, 4H).

^{13}C NMR (151 MHz, $CDCl_3$) δ 166.4, 154.9, 143.5, 130.6, 129.3, 129.2, 129.2, 124.8, 79.9, 59.0, 51.8, 49.0, 37.7, 36.4, 32.2, 28.6.

HRMS (ESI) calcd. for $[M+Na]^+$ $(C_{23}H_{34}N_2O_6SNa)^+$ 489.2030, found 489.2046.

FT-IR (thin film) ν_{max} (cm^{-1}) = 3291, 2978, 1689, 1433, 1332, 1279, 1156, 914, 735.

M.P. 120-122 °C.

1-allylcyclopentane-1-carbonitrile (S21)

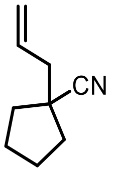

Compound **S21** was synthesised according to **General Procedure 3A** from cyclopentane carbonitrile (2.1 mL, 20.0 mmol) and was purified by FCC (pentane/EtOAc 10%) to afford **S21** as a pale yellow oil (2.52 g, 18.6 mmol, 93%).

^{1}H NMR (400 MHz, $CDCl_3$) δ 5.94 – 5.80 (m, 1H), 5.19 (d, J = 1.1 Hz, 1H), 5.17 – 5.13 (m, 1H), 2.32 (dt, J = 7.3, 1.1 Hz, 2H), 2.14 – 2.04 (m, 2H), 1.90 – 1.57 (m, 6H).

^{13}C NMR (101 MHz, CDCl3) δ 133.0, 125.1, 119.3, 42.6, 42.5, 37.6, 24.3.

HRMS (ESI) calcd. for $[M+Na]^+$ $(C_9H_{13}NNa)^+$ 158.0940, found 158.0947.

The data is consistent with the literature.[10]

[back to table of contents]

N-((1-allylcyclopentyl)methyl)-1-phenylmethanesulfonamide (S22)

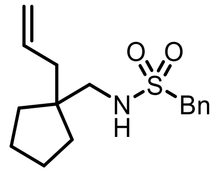

Compound **S22** was synthesised according to **General Procedure 3B** from **S21** (1.36 g, 10.0 mmol) and was purified by FCC (pentane/EtOAc 10% to 30%) to afford **S22** as an off white solid (1.49 g, 5.1 mmol, 51% over 2 steps).

^{1}H NMR (600 MHz, $CDCl_3$) δ 7.38 (s, 5H), 5.74 (ddt, J = 17.4, 10.2, 7.4 Hz, 1H), 5.05 – 4.96 (m, 2H), 4.24 (s, 2H), 4.16 (t, J = 6.6 Hz, 1H), 2.87 (d, J = 6.6 Hz, 2H), 2.05 (dt, J = 7.4, 1.3 Hz, 2H), 1.64 – 1.57 (m, 4H), 1.43 – 1.35 (m, 4H).

^{13}C NMR (151 MHz, $CDCl_3$) δ 135.3, 130.7, 129.6, 129.0, 128.9, 117.9, 58.6, 50.6, 46.0, 42.4, 35.2, 24.9.

HRMS (ESI) calcd. for $[M+H]^+$ $(C_{16}H_{24}NO_2S)^+$ 294.1522, found 294.1519.

FT-IR (thin film) ν_{max} (cm^{-1}) = 3287, 2954, 2870, 1496, 1329, 1155, 1127, 1073, 916, 698.

M.P. 82-84 °C.

methyl (*E*)-4-(1-(((phenylmethyl)sulfonamido)methyl)cyclopentyl)but-2-enoate (7q)

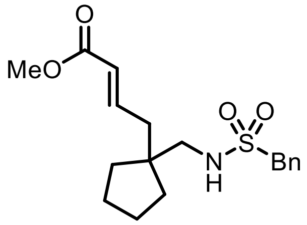

Compound **7q** was synthesised according to **General Procedure 1** from compound **S22** (501 mg, 1.70 mmol) and was purified by FCC (pentane/EtOAc 10% to 30%) followed by trituration in Et_2O to afford **7q** as an off white solid (260 mg, 0.740 mmol, 43%).

^{1}H NMR (600 MHz, $CDCl_3$) δ 7.38 (s, 5H), 6.87 (dt, J = 15.5, 7.7 Hz, 1H), 5.82 (d, J = 15.7 Hz, 1H), 4.29 (t, J = 5.9 Hz, 1H), 4.23 (s, 2H), 3.74 (d, J = 0.7 Hz, 3H), 2.83 (d, J = 6.6 Hz, 2H), 2.20 (dd, J = 7.8, 1.5 Hz, 2H), 1.64 – 1.56 (m, 4H), 1.43 – 1.39 (m, 4H).

^{13}C NMR (151 MHz, $CDCl_3$) δ 166.7, 145.7, 130.6, 129.4, 129.1, 129.0, 123.9, 58.7, 51.7, 50.7, 46.4, 40.1, 35.1, 24.9.

HRMS (ESI) calcd. for $[M+NH_4]^+$ $(C_{18}H_{29}N_2O_4S)^+$ 369.1843, found 369.1842.

FT-IR (thin film) ν_{max} (cm^{-1}) = 3286, 2951, 1719, 1615, 1496, 1436, 1327, 1278, 1155, 1127, 734, 699.

M.P. 58-60 °C.

[back to table of contents]

1-allylcyclobutane-1-carbonitrile (S23)

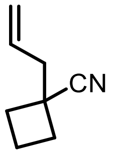

Compound **S23** was synthesised according to **General Procedure 3A** from cyclobutene carbonitrile (1.68 g, 20.0 mmol) and was purified by FCC (pentane/EtOAc 10%) to afford **S23** as a pale yellow oil (2.05 g, 16.9 mmol, 82%).

^{1}H NMR (400 MHz, $CDCl_3$) δ 5.84 – 5.68 (m, 1H), 5.20 – 5.12 (m, 2H), 2.46 – 2.39 (m, 4H), 2.10 – 1.95 (m, 4H).

^{13}C NMR (101 MHz, CDCl3) δ 131.9, 124.2, 119.3, 41.7, 35.0, 31.2, 16.5.

HRMS (ESI) calcd. for $[M+H]^+$, $(C_8H_{12}N)^+$ 122.0964, found 122.0959.

The data is consistent with the literature.[10]

***N*-((1-allylcyclobutyl)methyl)-1-phenylmethanesulfonamide (S24)**

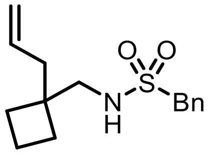

Compound **S24** was synthesised according to **General Procedure 3B** from compound **S23** (1.15 g, 10 mmol) and was purified by FCC (pentane/EtOAc 5% to 20%) to afford **S24** as an off white solid (727 mg, 2.6 mmol, 26% over 2 steps).

^{1}H NMR (600 MHz, $CDCl_3$) δ 7.42 – 7.36 (m, 5H), 5.72 (ddt, *J* = 17.4, 10.2, 7.3 Hz, 1H), 5.06 – 5.00 (m, 2H), 4.26 (s, 2H), 4.15 (p, *J* = 6.6 Hz, 1H), 3.00 (dd, *J* = 6.5, 1.4 Hz, 2H), 2.17 (d, *J* = 7.3 Hz, 1H), 1.85 (dtd, *J* = 16.7, 8.5, 3.6 Hz, 2H), 1.76 (t, *J* = 7.3 Hz, 4H).

^{13}C NMR (151 MHz, $CDCl_3$) δ 134.3, 130.7, 129.6, 129.0, 128.9, 117.9, 58.7, 50.5, 41.9, 41.3, 28.8, 15.0.

HRMS (ESI) calcd. for $[M+H]^+$, $(C_{15}H_{22}NO_2S)^+$ 280.1366, found 280.1367.

FT-IR (thin film) ν_{max} (cm^{-1}) = 3289, 2976, 2933, 1455, 1433, 1329, 1154, 1127, 1073, 919, 698.

M.P. 46-48 °C.

[back to table of contents]

methyl (*E*)-4-(1-(((phenylmethyl)sulfonamido)methyl)cyclobutyl)but-2-enoate (7r)

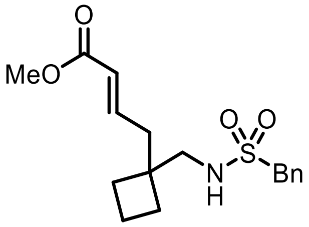

Compound **7r** was synthesised according to **General Procedure 1** from compound **S24** (502 mg, 1.79 mmol) and was purified by FCC (pentane/EtOAc 30% to 40%) followed by trituration in Et_2O to afford **7r** as an off white solid (196 mg, 0.581 mmol, 32%).

^{1}H NMR (600 MHz, $CDCl_3$) δ 7.39 (s, 5H), 6.85 (dt, *J* = 15.5, 7.7 Hz, 1H), 5.85 (dt, *J* = 15.6, 1.4 Hz, 1H), 4.25 (s, 2H), 4.23 – 4.16 (m, 1H), 3.74 (s, 3H), 2.96 (dd, *J* = 6.4, 1.2 Hz, 2H), 2.31 (dd, *J* = 7.7, 1.4 Hz, 2H), 1.91 – 1.82 (m, 2H), 1.78 (t, *J* = 7.2 Hz, 4H).

^{13}C NMR (151 MHz, $CDCl_3$) δ 166.7, 144.8, 144.8, 130.7, 129.4, 129.1, 129.0, 124.0, 58.8, 51.7, 50.4, 41.6, 39.7, 28.6, 15.0.

HRMS (ESI) calcd. for $[M+H]^+$, $(C_{17}H_{24}NO_4S)^+$ 338.1421, found 338.1422.

FT-IR (thin film) ν_{max} (cm^{-1}) = 3287, 2951, 1720, 1496, 1455, 1330, 1273, 1155, 1128, 734, 699.

M.P. 98-100 °C.

2,2-diphenylpent-4-enenitrile (S25)

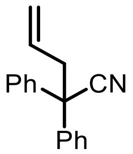

Compound **S25** was synthesised according to **General Procedure 3A** from diphenylacetonitrile (3.88 g, 20.0 mmol) and was purified by FCC (pentane/EtOAc 5%) to afford **S25** as a pale yellow oil (4.67 g, 20.0 mmol, quant).

^{1}H NMR (400 MHz, $CDCl_3$) δ 7.45 – 7.28 (m, 10H), 5.74 (ddt, J = 17.1, 10.1, 7.0 Hz, 1H), 5.27 – 5.15 (m, 2H), 3.16 (dt, J = 7.0, 1.2 Hz, 2H).

^{13}C NMR (101 MHz, $CDCl_3$) δ 139.8, 131.9, 128.9, 128.0, 127.1, 122.0, 120.5, 51.8, 44.0.

HRMS (ESI) calcd. for $[M+H]^+$, $(C_{17}H_{16}N)^+$ 234.1277, found 234.1281.

The data is consistent with the literature.[11]

N-(2,2-diphenylpent-4-en-1-yl)-1-phenylmethanesulfonamide (S26)

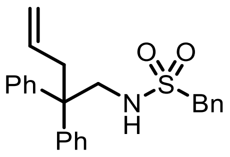

Compound **S26** was synthesised according to **General Procedure 3B** from compound **S25** (2.40 g, 10 mmol) and was purified by FCC (pentane/Et_2O 40% to 50%) followed by trituration in Et_2O to afford **S26** as a white solid (781 mg, 2.0 mmol, 20% over 2 steps).

^{1}H NMR (400 MHz, CDCl3) δ 7.34 – 7.26 (m, 7H), 7.26 – 7.16 (m, 4H), 7.13 – 7.06 (m, 4H), 5.33 (ddt, J = 16.4, 10.1, 7.2 Hz, 1H), 5.10 – 4.96 (m, 2H), 4.10 (s, 2H), 3.69 (d, J = 5.8 Hz, 2H), 3.65 – 3.60 (m, 1H), 2.91 (d, J = 7.2 Hz, 2H).

^{13}C NMR (101 MHz, CDCl3) δ 144.8, 133.3, 130.5, 129.2, 129.0, 128.8, 128.6, 128.0, 126.9, 119.3, 58.5, 50.1, 49.8, 41.2.

HRMS (ESI) calcd. for $[M+H]^+$, $(C_{24}H_{26}NO_2S)^+$ 392.1679, found 392.1678.

The data is consistent with the literature.[12]

methyl (E)-5,5-diphenyl-6-((phenylmethyl)sulfonamido)hex-2-enoate (7s)

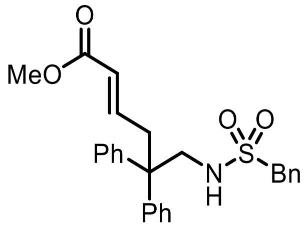

Compound **7s** was synthesised according to **General Procedure 1** from compound **S26** (497 mg, 1.28 mmol) and was purified by FCC (pentane/EtOAc 20% to 40%) to afford **7s** as a white solid (495 mg, 1.10 mmol, 87%).

^{1}H NMR (600 MHz, $CDCl_3$) δ 7.36 – 7.27 (m, 7H), 7.26 – 7.20 (m, 4H), 7.06 (dd, J = 8.2, 1.4 Hz, 4H), 6.51 (dt, J = 15.2, 7.4 Hz, 1H), 5.81 (dt, J = 15.5, 1.4 Hz, 1H), 4.15 (s, 2H), 3.66 (s, 3H), 3.62 (s, 3H), 3.05 – 3.02 (m, 2H).

^{13}C NMR (151 MHz, $CDCl_3$) δ 166.5, 143.9, 143.9, 130.5, 129.1, 129.1, 128.9, 128.8, 127.8, 127.3, 125.0, 58.7, 51.6, 50.6, 50.1, 39.6.

HRMS (ESI) calcd. for $[M+H]^+$, $(C_{26}H_{28}NO_4S)^+$ 450.1734, found 450.1734.

FT-IR (thin film) ν_{max} (cm^{-1}) = 3283, 1718, 1655, 1437, 1333, 1156, 912, 734, 699.

M.P. 118-120 °C.

[back to table of contents]

methyl (*E*)-6,6-diphenyl-7-(phenylsulfonamido)hept-2-enoate (7t)

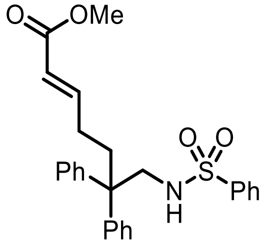

Compound **7t** was synthesised according to **General Procedure 1** from literature known alkene *N*-(2,2-diphenylhex-5-en-1-yl)benzenesulfonamide[13] (587 mg, 1.50 mmol) and was purified by FCC (pentane/EtOAc 75%) to afford **7st** as a white solid (572 mg, 127 μmol, 84%).

^{1}H NMR (400 MHz, $CDCl_3$) δ 7.76 – 7.71 (m, 2H), 7.61 – 7.54 (m, 1H), 7.52 – 7.46 (m, 2H), 7.30 – 7.18 (m, 6H), 7.07 – 7.01 (m, 4H), 6.77 (dt, *J* = 15.7, 6.8 Hz, 1H), 5.67 (dt, *J* = 15.6, 1.5 Hz, 1H), 3.91 (t, *J* = 6.5 Hz, 1H), 3.70 (s, 3H), 3.58 (d, *J* = 6.6 Hz, 2H), 2.27 – 2.20 (m, 2H), 1.86 – 1.76 (m, 2H).

^{13}C NMR (101 MHz, $CDCl_3$) δ 167.1, 148.7, 144.5, 139.6, 132.9, 129.3, 128.7, 127.7, 127.1, 127.1, 121.2, 51.5, 49.7, 49.4, 34.9, 27.0.

HRMS (ESI) calcd. for $[M+H]^+$ $(C_{26}H_{27}NO_4S)^+$ 450.1734, found 450.1731.

M.P. 70-72 °C.

[back to table of contents]

Synthesis of *α*-Disubstituted Pyrrolidine Precursors

General Procedure 4

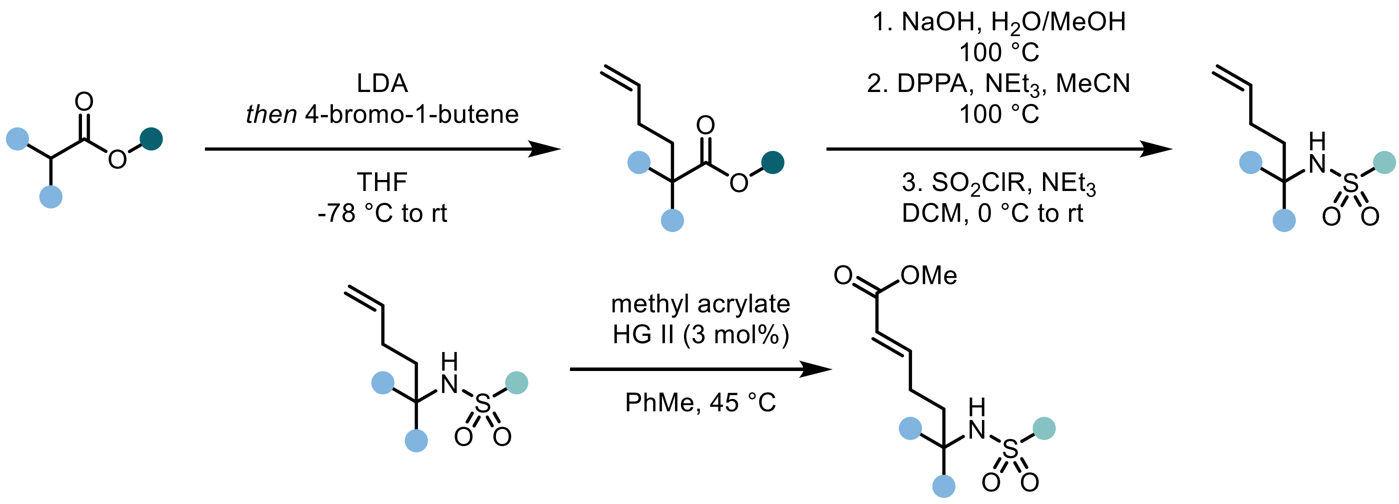

General Procedure 4A In a flame dried flask under a nitrogen atmosphere, a solution of diisopropylamine (1.25 equiv.) in THF (0.4 M) was cooled to -78 °C. To this solution was added *n*BuLi (2.5 M in hexanes, 1.2 equiv.) dropwise over 15 mins. After stirring for 30 mins, the respective ester (1 equiv.) was added dropwise over 15 mins while stirring at -78 °C. After stirring for a further 30 mins, 4-bromo-1-butene (1.5 equiv.) was added dropwise over 10 mins, and the reaction mixture left to warm to room temperature overnight. Once complete, the reaction was quenched with aqueous sat. NH_4Cl (50 mL), extracted with Et_2O (3 × 50 mL) and washed with brine (100 mL). The combined organics were dried over Na_2SO_4, filtered, and concentrated *in vacuo*. The crude residue was purified by FCC according to the specific experiment to give the pure alkene.

General Procedure 4B To a solution of the appropriate alkene (1 equiv.) in water/methanol 1:2 (v/v) was added solid NaOH (2 equiv.). The reaction mixture was heated to reflux while stirring. Once complete, the mixture was allowed to cool to room temperature, and acidified with 1 M HCl to pH 1. The aqueous phase was extracted with Et_2O (3 × 100 mL), the combined organic layers were washed with brine (100 mL), dried over Mg_2SO_4, filtered and concentrated *in vacuo*. The crude carboxylic acid was used in the subsequent step without further purification.

To a solution of the appropriate crude carboxylic acid (1 equiv.) in acetonitrile (0.5 M) was added triethylamine (1.1 equiv.) and diphenylphosphoryl azide (1.9 equiv.), and the reaction mixture stirred at room temperature for 15 minutes. After 15 minutes, the temperature was raised to 50 °C for 3 hours, following which the reaction mixture was allowed to cool to room temperature, and acidified with 1 M HCl (1 equiv.). The reaction mixture was then heated to reflux overnight. Once complete, the reaction was quenched with 1 M NaOH, the aqueous phase extracted with Et_2O (3 × 50 mL), then the combined organic layers were washed with brine (100 mL), dried over Na_2SO_4, filtered and concentrated *in vacuo*. The crude amine was used in the subsequent step without further purification.

In a flame dried flask under a nitrogen atmosphere, the appropriate crude amine (1 equiv.) was dissolved in DCM (0.3 M). To this solution was added triethylamine (2 equiv.) and the solution cooled to 0 °C. To this solution was added phenylmethanesulfonyl chloride (1.5 equiv.) and the mixture allowed to warm to room temperature overnight. Once complete, the reaction was diluted with DCM and quenched with saturated $NaHCO_3$. The crude mixture was extracted with DCM (3 × 50 mL), washed with brine (100 mL), dried over Na_2SO_4, filtered and concentrated *in vacuo*. The crude residue was purified by FCC according to the specific experiment to give the pure sulfonamide.

[back to table of contents]

ethyl 2,2-dimethylhex-5-enoate (S27)

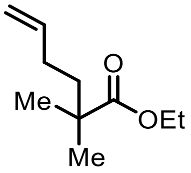

Compound **S27** was synthesised according to **General Procedure 4A** from ethyl isobutyrate (2.70 mL, 20.0 mmol) and was purified by FCC (pentane/Et_2O 5% to 10%) to afford **S27** as a pale yellow oil (2.90 g, 17.0 mmol, 85%).

^{1}H NMR (400 MHz, $CDCl_3$) δ 5.77 (ddt, J = 16.8, 10.2, 6.5 Hz, 1H), 5.04 – 4.86 (m, 2H), 4.10 (q, J = 7.1 Hz, 2H), 2.03 – 1.92 (m, 2H), 1.64 – 1.54 (m, 2H), 1.23 (t, J = 7.1 Hz, 3H), 1.16 (s, 6H).

^{13}C NMR (101 MHz, $CDCl_3$) δ 177.9, 138.7, 114.5, 60.3, 42.1, 39.9, 29.5, 25.2, 14.3.

HRMS (ESI) calcd. for $[M+H]^+$ $(C_{10}H_{19}O_2)^+$ 171.1380, found 171.1379.

The data is consistent with the literature.[10]

***N*-(2-methylhex-5-en-2-yl)-1-phenylmethanesulfonamide (S28)**

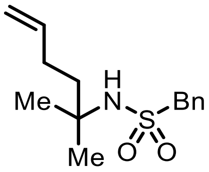

Compound **S28** was synthesised according to **General Procedure 4B** from compound **S27** (714 mg, 5.00 mmol) and was purified by FCC (pentane/EtOAc 10% to 30%) to afford **S28** as a white solid (243 mg, 0.9 mmol, 18% over 3 steps).

^{1}H NMR (600 MHz, $CDCl_3$) δ 7.44 – 7.34 (m, 5H), 5.78 (ddt, *J* = 16.8, 10.2, 6.5 Hz, 1H), 5.01 (dd, *J* = 17.1, 1.7 Hz, 1H), 4.96 (dd, *J* = 10.1, 1.5 Hz, 1H), 4.25 (s, 2H), 3.84 (s, 1H), 2.10 – 2.05 (m, 2H), 1.69 – 1.65 (m, 2H), 1.34 (s, 6H).

^{13}C NMR (151 MHz, $CDCl_3$) δ 138.1, 130.9, 130.0, 128.8, 128.8, 115.1, 62.2, 57.6, 42.4, 28.6, 27.8.

HRMS (ESI) calcd. for $[M+Na]^+$ $(C_{14}H_{21}NO_2SNa)^+$ 290.1185, found 290.1185.

FT-IR (thin film) ν_{max} (cm^{-1}) = 3291, 2978, 1325, 1146, 1126, 1000, 914, 698.

M.P. 38-40 °C.

[back to table of contents]

methyl (*E*)-6-methyl-6-((phenylmethyl)sulfonamido)hept-2-enoate (7u)

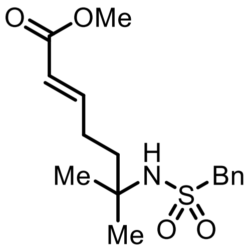

Compound **7u** was synthesised according to **General Procedure 1** from compound **S28** (250 mg, 0.93 mmol) and was purified by FCC (pentane/EtOAc 10% to 30%) to afford **7u** as an off white solid (255 mg, 0.784 mmol, 84%).

^{1}H NMR (600 MHz, $CDCl_3$) δ 7.41 – 7.31 (m, 5H), 6.91 (dt, *J* = 15.7, 6.8 Hz, 1H), 5.80 (dt, *J* = 15.7, 1.6 Hz, 1H), 4.21 (s, 2H), 4.18 (s, 1H), 3.70 (s, 3H), 2.24 – 2.17 (m, 2H), 1.73 – 1.68 (m, 2H), 1.31 (s, 6H).

^{13}C NMR (151 MHz, $CDCl_3$) δ 167.0, 148.6, 130.9, 129.9, 128.8, 121.3, 62.1, 57.2, 51.5, 41.1, 27.8, 27.1.

HRMS (ESI) calcd. for $[M+H]^+$ $(C_{16}H_{24}NO_4S)^+$ 326.1421, found 326.1416.

FT-IR (thin film) ν_{max} (cm^{-1}) = 3279, 2979, 2359, 1724, 1657, 1436, 1328, 1138, 997, 736, 699.

M.P. 82-84 °C.

ethyl 1-(but-3-en-1-yl)cyclobutane-1-carboxylate (S29)

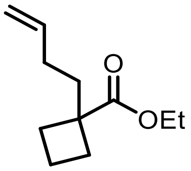

Compound **S29** was synthesised according to **General Procedure 4A** from ethyl cyclobutanecarboxylate (2.76 g, 20.0 mmol) and was purified by FCC (pentane/EtOAc 5%) to afford **S29** as a pale yellow oil (2.76 g, 15.1 mmol, 75%).

^{1}H NMR (400 MHz, CDCl3) δ 5.85 – 5.71 (m, 1H), 5.04 – 4.86 (m, 2H), 4.12 (q, J = 7.1 Hz, 2H), 2.47 – 2.34 (m, 2H), 1.99 – 1.78 (m, 8H), 1.24 (t, J = 7.1 Hz, 3H).

^{13}C NMR (101 MHz, CDCl3) δ 177.1, 138.4, 114.6, 60.3, 47.6, 37.4, 30.1, 29.4, 15.7, 14.4.

HRMS (ESI) calcd. for $[M+H]^+$ $(C_{11}H_{19}O_2)^+$ 183.1380, found 183.1375.

The data is consistent with the literature.[14]

[back to table of contents]

N-(1-(but-3-en-1-yl)cyclobutyl)-1-phenylmethanesulfonamide (S30)

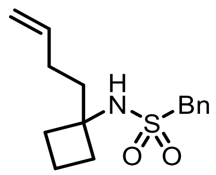

Compound **S30** was synthesised according to **General Procedure 4B** compound **S29** (776 mg, 5.00 mmol) and was purified by FCC (pentane/EtOAc 10% to 30%) to afford **S30** as a colourless oil that solidifies on standing (613 mg, 2.2 mmol, 44% over 3 steps, contains ~3 wt% EtOAc by ^{1}H NMR).

^{1}H NMR (600 MHz, $CDCl_3$) δ 7.43 – 7.35 (m, 5H), 5.82 (ddt, *J* = 16.8, 10.2, 6.5 Hz, 1H), 5.04 (dq, *J* = 17.1, 1.7 Hz, 1H), 4.97 (dq, *J* = 10.2, 1.4 Hz, 1H), 4.25 (s, 2H), 4.16 (d, *J* = 9.8 Hz, 1H), 2.28 – 2.21 (m, 2H), 2.12 – 2.06 (m, 2H), 2.02 – 1.96 (m, 2H), 1.88 – 1.83 (m, 3H), 1.77 – 1.68 (m, 1H).

^{13}C NMR (151 MHz, $CDCl_3$) δ 138.2, 131.0, 129.7, 128.8, 128.8, 115.2, 62.3, 60.2, 38.5, 33.4, 28.3, 14.8.

HRMS (ESI) calcd. for $[M+H]^+$ $(C_{15}H_{22}NO_2S)^+$ 280.1366, found 280.1354.

FT-IR (thin film) ν_{max} (cm^{-1}) = 3272, 2941, 1414, 1323, 1149, 1125, 982, 911, 783, 697.

M.P. 28-30 °C.

methyl (*E*)-5-(1-((phenylmethyl)sulfonamido)cyclobutyl)pent-2-enoate (7v)

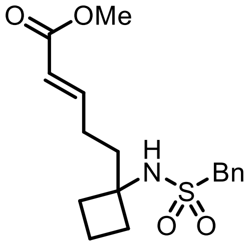

Compound **7v** was synthesised according to **General Procedure 1** from compound **S30** (252 mg, 0.91 mmol) and was purified by FCC (pentane/EtOAc 10% to 30%) to afford **7v** as an off white solid (233 mg, 0.69 mmol, 76%).

^{1}H NMR (600 MHz, $CDCl_3$) δ 7.43 – 7.35 (m, 5H), 6.95 (dt, *J* = 15.6, 6.8 Hz, 1H), 5.84 (dt, *J* = 15.7, 1.6 Hz, 1H), 4.24 (s, 2H), 4.22 (s, 1H), 3.72 (s, 3H), 2.25 – 2.15 (m, 4H), 2.01 – 1.94 (m, 2H), 1.93 – 1.82 (m, 3H), 1.79 – 1.68 (m, 1H).

^{13}C NMR (151 MHz, $CDCl_3$) δ 167.0, 148.6, 130.9, 129.6, 128.9, 128.9, 121.4, 62.2, 59.9, 51.6, 37.7, 33.5, 26.9, 14.8.

HRMS (ESI) calcd. for $[M+Na]^+$ $(C_{17}H_{23}NO_4SNa)^+$ 360.1240, found 360.1225.

FT-IR (thin film) ν_{max} (cm^{-1}) = 3279, 2946, 2360, 1723, 1656, 1436, 1328, 1126, 912, 737.

M.P. 54-56 °C.

[back to table of contents]

methyl 4-(but-3-en-1-yl)tetrahydro-2*H*-pyran-4-carboxylate (S31)

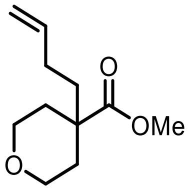

Compound **S31** was synthesised according to **General Procedure 4A** from methyl tetrahydro-2H-pyran-4-carboxylate (2.7 mL, 20.0 mmol) and was purified by FCC (pentane/EtOAc 20% to 40%) to afford **S31** as a pale yellow oil (3.84 g, 19.4 mmol, 97%).

^{1}H NMR (400 MHz, $CDCl_3$) δ 5.75 (ddt, J = 16.8, 10.2, 6.5 Hz, 1H), 5.04 – 4.92 (m, 2H), 3.83 (dt, J = 11.9, 3.8 Hz, 2H), 3.72 (s, 3H), 3.43 (td, J = 11.9, 2.2 Hz, 2H), 2.09 (dq, J = 13.7, 2.6 Hz, 2H), 1.96 (dddd, J = 11.9, 6.5, 3.8, 1.5 Hz, 2H), 1.66 – 1.58 (m, 2H), 1.52 (ddd, J = 13.7, 11.3, 4.4 Hz, 2H).

^{13}C NMR (101 MHz, $CDCl_3$) δ 176.2, 138.0, 115.0, 65.6, 51.9, 45.0, 40.1, 34.4, 28.2.

HRMS (ESI) calcd. for $[M+Na]^+$ $(C_{11}H_{18}O_3Na)^+$ 221.1148, found 221.1157.

The data is consistent with the literature.[15]

***N*-(4-(but-3-en-1-yl)tetrahydro-2*H*-pyran-4-yl)-1-phenylmethanesulfonamide (S32)**

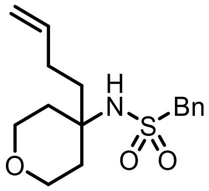

Compound **S32** was synthesised according to **General Procedure 4B** from compound **S31** (1.98 g, 10.0 mmol) and was purified by FCC (hexane/EtOAc 10% to 40%) to afford **S32** as a colourless oil (1.09 mg, 3.6 mmol, 36% over 3 steps).

^{1}H NMR (600 MHz, $CDCl_3$) δ 7.46 – 7.42 (m, 2H), 7.42 – 7.38 (m, 3H), 5.82 (ddt, *J* = 16.8, 10.2, 6.5 Hz, 1H), 5.05 (dq, *J* = 17.1, 1.6 Hz, 1H), 4.99 (dq, *J* = 10.2, 1.4 Hz, 1H), 4.27 (s, 2H), 3.81 (s, 1H), 3.74 – 3.63 (m, 4H), 2.17 – 2.10 (m, 2H), 1.95 – 1.86 (m, 4H), 1.78 – 1.71 (m, 2H).

^{13}C NMR (151 MHz, $CDCl_3$) δ 137.9, 130.9, 129.5, 129.0, 129.0, 115.3, 63.6, 62.7, 58.2, 38.1, 36.2, 27.5.

HRMS (ESI) calcd. for $[M+Na]^+$ $(C_{16}H_{23}NO_3SNa)^+$ 332.1291, found 332.1289.

FT-IR (thin film) ν_{max} (cm^{-1}) = 3265, 2980, 2360, 2341, 1306, 1146, 1125.

[back to table of contents]

ethyl (*E*)-5-(4-((phenylmethyl)sulfonamido)tetrahydro-2*H*-pyran-4-yl)pent-2-enoate (7w)

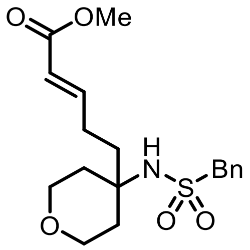

Compound **7w** was synthesised according to **General Procedure 1** from compound **S32** (232 mg, 0.75 mmol) and was purified by FCC (hexane/EtOAc 40% to 70%) to afford **7w** as a dark brown oil (185 mg, 0.50 mmol, 67%).

^{1}H NMR (600 MHz, $CDCl_3$) δ 7.44 (dd, *J* = 6.6, 3.2 Hz, 2H), 7.40 (dd, *J* = 5.0, 2.1 Hz, 3H), 6.95 (dt, *J* = 15.7, 6.8 Hz, 1H), 5.85 (dt, *J* = 15.7, 1.6 Hz, 1H), 4.28 (s, 2H), 3.75 (s, 1H), 3.72 (s, 3H), 3.70 – 3.66 (m, 4H), 2.31 – 2.24 (m, 2H), 1.98 – 1.94 (m, 2H), 1.89 – 1.83 (m, 2H), 1.73 (dt, *J* = 13.4, 6.3 Hz, 2H).

^{13}C NMR (151 MHz, $CDCl_3$) δ 167.0, 148.2, 130.9, 129.4, 129.1, 129.0, 121.7, 63.5, 62.7, 58.0, 51.6, 37.5, 36.2, 26.0.

HRMS (ESI) calcd. for $[M+Na]^+$ $(C_{18}H_{25}NO_5SNa)^+$ 390.1346, found 390.1353.

FT-IR (thin film) ν_{max} (cm^{-1}) = 3270, 2954, 1719, 1436, 1305, 1146.

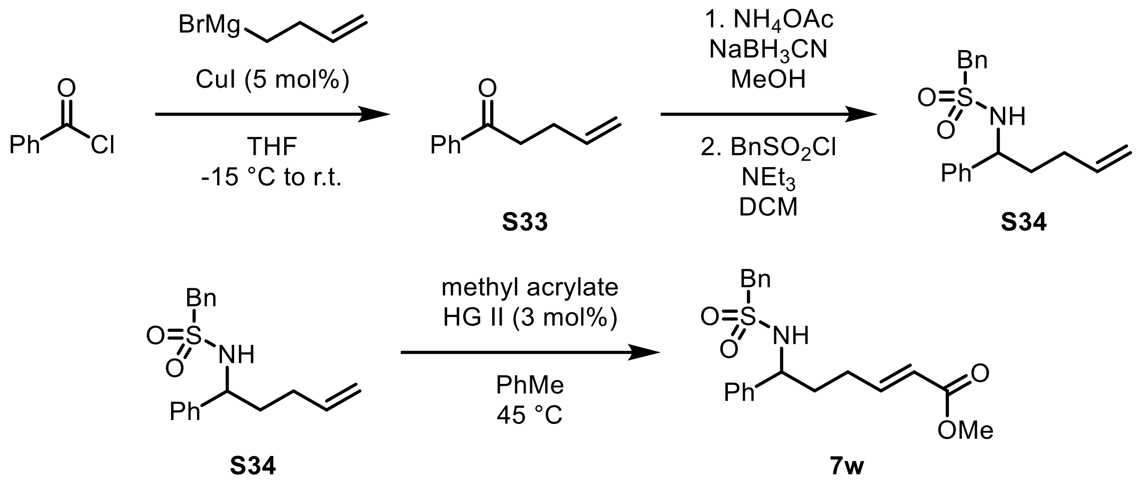

1-phenylpent-4-en-1-one (S33)

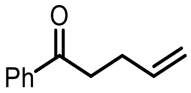

Compound **S33** was synthesised according to the modified literature procedure.[16] A round-bottom flask under a nitrogen atmosphere containing magnesium turnings (498 mg, 20.4 mmol) and iodine (~2 mg) was heated gently until purple vapours appeared, and then stirred vigorously for 10 minutes. To this mixture was added a solution of 4-bromobutene (2 mL, 20.0 mmol) in THF (20 mL) dropwise over 30 mins at room temperature. The reaction mixture was then stirred at room temperature for 1 hour. The reaction mixture was then added dropwise over 1 hour an round-bottom flask containing benzoyl chloride (2.3 mL, 20.0 mmol) and CuI (192 mg, 1.00 mmol) in THF (20 mL) at -15 °C. The reaction mixture was stirred overnight, and was then diluted with DCM (25 mL) and quenched with HCl (1 M, 25 mL). The aqueous phase was extracted with DCM (3 × 30 mL), the combined organic layers were washed with brine (100 mL), dried over $MgSO_4$, filtered and concentrated *in vacuo*. The crude residue was purified by FCC (hexane/EtOAc 0% to 5%) to afford **S33** as a pale yellow oil (1.56 g, 9.74 mmol, 49%).

^{1}H NMR (400 MHz, $CDCl_3$) δ 8.01 – 7.93 (m, 2H), 7.61 – 7.52 (m, 1H), 7.47 (dd, J = 8.2, 6.8 Hz, 2H), 5.91 (ddt, J = 16.9, 10.2, 6.5 Hz, 1H), 5.14 – 4.98 (m, 2H), 3.12 – 3.04 (m, 2H), 2.56 – 2.45 (m, 2H).

^{13}C NMR (101 MHz, $CDCl_3$) δ 199.6, 137.5, 137.1, 133.2, 128.7, 128.2, 115.4, 37.9, 28.

HRMS (ESI) calcd. for $[M+H]^+$ $(C_{11}H_{13}O)^+$ 161.0961, found 161.0961.

The data is consistent with the literature.[17]

[back to table of contents]

1-phenyl-*N*-(1-phenylpent-4-en-1-yl)methanesulfonamide (S34)

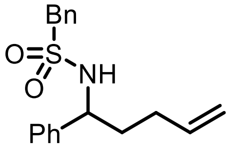

To a solution of compound **S33** (1.13 g, 7.00 mmol) in MeOH (20 mL) was added ammonium acetate (7.76 g, 100 mmol) and sodium cyanoborohydride (750 mg, 11.9 mmol). The reaction mixture was stirred at room temperature for 24 h, following which it was quenched in sat. aq. NH_4Cl solution (50 mL). The mixture was extracted with EtOAc (5 × 25 mL), and the combined organic layer was acidified to pH 2 with 1 M HCl. The acidified organic layer was then extracted with water (3 × 25 mL), and the combined aqueous layer was basified to pH 12 with solid KOH. This was extracted with Et_2O (3 × 50 mL), washed with brine (50 mL), dried over Na_2SO_4, filtered and concentrated *in vacuo* to afford primary amine **int-S34** as a yellow oil (474 mg) which was used without further purification.

To a solution of amine **int-S34** (403 mg, 2.50 mmol) in DCM (10 mL) was added NEt_3 (0.7 mL, 5.00 mmol), and the reaction mixture cooled to 0 °C. Phenylmethane sulfonyl chloride (727 mg, 3.75 mmol) was added, and the reaction mixture allowed to warm to room temperature overnight. Once complete, the solution was diluted with DCM (25 mL), and sat. $NaHCO_3$ (50 mL) was added. The aqueous layer was extracted with DCM (3 × 50 mL), the combined organics were washed with brine (100 mL), dried over Na_2SO_4, filtered and concentrated *in vacuo*. The crude residue was purified by FCC (pentane/Et_2O 30% to 50%) to afford **S34** as a white solid (497 mg, 1.58 mmol, 26% over two steps).

^{1}H NMR (600 MHz, $CDCl_3$) δ 7.43 – 7.39 (m, 2H), 7.37 – 7.34 (m, 1H), 7.31 – 7.20 (m, 5H), 7.02 – 6.98 (m, 2H), 5.76 (ddt, *J* = 17.0, 10.5, 6.5 Hz, 1H), 5.02 – 4.96 (m, 2H), 4.47 – 4.41 (m, 2H), 3.99 (d, *J* = 13.9 Hz, 1H), 3.84 (d, *J* = 13.8 Hz, 1H), 2.08 – 1.89 (m, 3H), 1.87 – 1.79 (m, 1H).

^{13}C NMR (151 MHz, $CDCl_3$) δ 141.4, 137.2, 130.8, 129.2, 129.1, 128.7, 128.7, 128.3, 127.1, 115.8, 60.0, 58.3, 36.9, 30.3.

HRMS (ESI) calcd. for $[M+H]^+$ $(C_{18}H_{22}NO_2S)^+$ 316.1366, found 316.1378.

FT-IR (thin film) ν_{max} (cm^{-1}) = 3277, 1495, 1319, 1153, 1126, 698.

M.P. 52-54 °C.

[back to table of contents]

methyl (*E*)-6-phenyl-6-((phenylmethyl)sulfonamido)hex-2-enoate (7x)

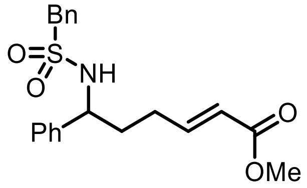

Compound **7x** was synthesised according to **General Procedure 1** from compound **S34** (314 mg, 1.00 mmol) and was purified by FCC (pentane/EtOAc 10% to 20%) to afford **7x** as an off white solid (345 mg, 0.92 mmol, 93%).

^{1}H NMR (600 MHz, $CDCl_3$) δ 7.43 – 7.40 (m, 2H), 7.39 – 7.35 (m, 1H), 7.31 – 7.27 (m, 1H), 7.22 (ddt, *J* = 6.5, 5.5, 1.5 Hz, 4H), 6.98 – 6.95 (m, 2H), 6.87 (dt, *J* = 15.7, 6.9 Hz, 1H), 5.78 (dt, *J* = 15.7, 1.6 Hz, 1H), 4.44 (d, *J* = 8.1 Hz, 1H), 4.39 (q, *J* = 7.5 Hz, 1H), 4.03 (d, *J* = 13.9 Hz, 1H), 3.86 (d, *J* = 13.9 Hz, 1H), 3.72 (s, 3H), 2.26 – 2.17 (m, 1H), 2.17 – 2.09 (m, 1H), 2.03 – 1.95 (m, 1H), 1.90 – 1.83 (m, 1H).

^{13}C NMR (151 MHz, $CDCl_3$) δ 167.0, 147.5, 140.9, 130.8, 129.4, 128.9, 128.7, 128.7, 128.6, 126.9, 122.0, 60.1, 58.3, 51.6, 36.0, 28.9.

HRMS (ESI) calcd. for $[M+H]^+$ $(C_{20}H_{24}NO_4S)^+$ 374.1421, found 374.1416.

FT-IR (thin film) ν_{max} (cm^{-1}) = 3266, 2342, 1821, 1657, 1495, 1321, 1153.

M.P. 108-110 °C.

[back to table of contents]

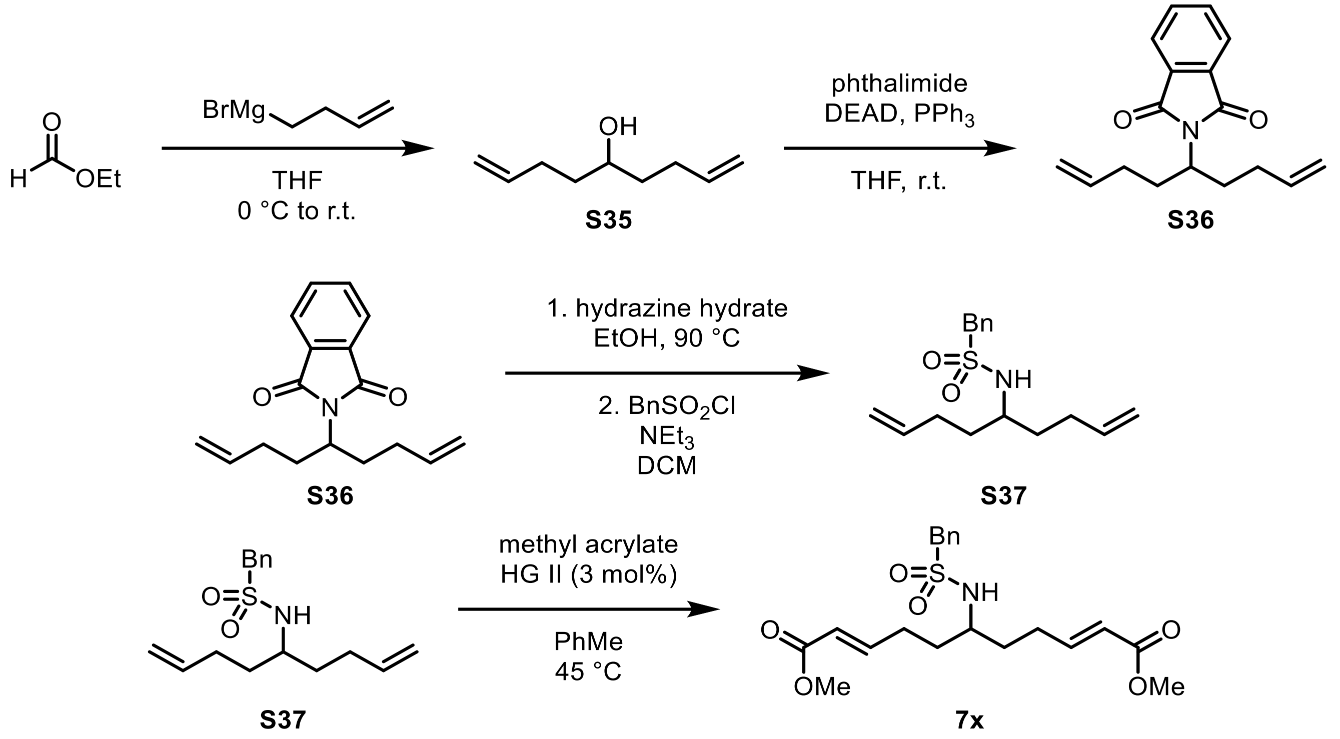

nona-1,8-dien-5-ol (S35)

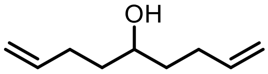

Alcohol **S35** was prepared according to the literature procedure.[18] A round-bottom flask containing Mg turnings (1.21 g, 50.0 mmol) and iodine (~ 2 mg) was heated gently until purple vapours appeared, at which point it was stirred vigorously for 10 minutes. THF (40 mL) was added, and to this suspension was added a solution of 4-bromobutene (5.1 mL, 50.0 mmol) in THF (10 mL) dropwise over 15 mins. The reaction mixture was stirred at room temperature for 1 hour, following which it was cooled to 0 °C, and a solution of ethyl formate (1.6 mL, 20.0 mmol) in THF (5 mL) was added dropwise over 15 mins, and the resultant mixture was stirred for 2 h. Once complete, the mixture was quenched with sat. NH_4Cl (50 mL), the aqueous layer was extracted with Et_2O (3 × 50 mL), the combined organic layer was washed with brine (50 mL), dried over Na_2SO_4, filtered and concentrated *in vacuo*. The crude residue was purified by FCC (hexane/EtOAc 0% to 10%) to afford **S35** as a colourless oil (1.93 g, 13.8 mmol, 69%).

^{1}H NMR (400 MHz, $CDCl_3$) δ 5.84 (ddt, J = 17.0, 10.2, 6.6 Hz, 2H), 5.05 (dq, J = 17.0, 1.7 Hz, 2H), 4.97 (ddd, J = 10.2, 2.1, 1.1 Hz, 2H), 3.65 (tq, J = 8.6, 4.5 Hz, 1H), 2.28 – 2.06 (m, 4H), 1.62 – 1.44 (m, 5H).

^{13}C NMR (101 MHz, $CDCl_3$) δ 138.7, 114.9, 71.1, 36.6, 30.2.

HRMS (ESI) calcd. for $[M+H]^+$ $(C_9H_{17}O)^+$ 141.1274, found 141.1270.

The data is consistent with the literature.[18]

[back to table of contents]

2-(nona-1,8-dien-5-yl)isoindoline-1,3-dione (S36)

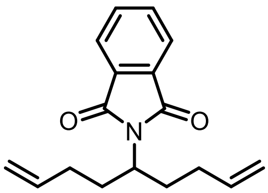

Compound **S36** was prepared according to the literature procedure.[16] To a solution of phthalimide (2.06 g, 14.0 mmol) and triphenyl phosphine (3.65 g, 14.0 mmol) in THF (65 mL) was added alcohol **S35** (1.40 g, 10.0 mmol), followed by dropwise addition of DEAD (2.2 mL, 14.0 mmol) in toluene (3.3 mL) over 20 mins. The reaction mixture was stirred at room temperature for 24 h, following which the solvent was removed under reduced pressure, the crude oil was triturated with hexane/Et_2O (2/1), and the precipitated solids were removed by filtration, and washed with the same solvent mixture. The crude mixture was concentrated *in vacuo* and was purified by FCC (hexane/EtOAc 0% to 10%) to afford **S36** as a pale yellow oil (1.38 g, 5.12 mmol, 51%).

^{1}H NMR (400 MHz, $CDCl_3$) δ 7.82 (dd, *J* = 5.4, 3.1 Hz, 2H), 7.71 (dd, *J* = 5.5, 3.0 Hz, 2H), 5.75 (ddt, *J* = 16.9, 10.2, 6.6 Hz, 2H), 4.96 (dq, *J* = 17.1, 1.6 Hz, 2H), 4.92 – 4.88 (m, 2H), 4.30 – 4.20 (m, 1H), 2.31 – 2.16 (m, 2H), 2.06 – 1.98 (m, 4H), 1.86 – 1.74 (m, 2H).

^{13}C NMR (101 MHz, $CDCl_3$) δ 168.9, 137.6, 134.0, 132.0, 123.3, 115.3, 51.4, 31.7, 31.0.

HRMS (ESI) calcd. for $[M+Na]^+$ $(C_{17}H_{19}NO_2Na)^+$ 292.1308, found 292.1308.

FT-IR (thin film) ν_{max} (cm^{-1}) = 2930.75, 1708.72, 1468.61, 1395.86, 909.63, 731.82.

[back to table of contents]

N-(nona-1,8-dien-5-yl)-1-phenylmethanesulfonamide (S37)

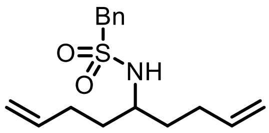

Compound **S36** (1.23 g, 4.5 mmol) was dissolved in EtOH (50 mL). To this mixture was added hydrazine hydrate (0.48 mL, 9.70 mmol), and the reaction mixture was stirred at reflux for 24 h. Once complete, the reaction mixture was cooled to r.t., acidified to pH 1 with 1 M HCl, and the organic solvents were removed *in vacuo*. The crude residue was taken up in H_2O (50 mL), washed with Et_2O (2 × 50 mL), and basified to pH 14 with NaOH. The aqueous layer was extracted with Et_2O (3 × 100 mL), the combined organics were dried over Na_2SO_4, filtered, and concentrated *in vacuo* to afford amine **int-S37** as a yellow oil (349 mg) which was used without purification in the subsequent step.

Amine **int-S37** (277 mg, 2.00 mmol) and NEt_3 (0.55 mL, 4.00 mmol) were dissolved in DCM (10 mL) and the solution was cooled to 0 °C. To this solution was added phenylmethane sulfonyl chloride (574 mg, 3.00 mmol), and the reaction mixture was allowed to warm to r.t. overnight. Once complete, the reaction was diluted with DCM (15 mL) and quenched with saturated aqueous $NaHCO_3$ (50 mL). The aqueous layer was extracted with DCM (3 × 50 mL), the combined organics were washed with brine (100 mL), dried over Na_2SO_4, filtered and concentrated *in vacuo*. The crude residue was purified by FCC (pentane/EtOAc 10% to 20%) to afford **S37** as a colourless oil (364 mg, 1.24 mmol, 34% over two steps).

^{1}H NMR (600 MHz, $CDCl_3$) δ 7.44 – 7.40 (m, 2H), 7.40 – 7.35 (m, 3H), 5.77 (ddt, *J* = 16.9, 10.2, 6.6 Hz, 2H), 5.03 (dq, *J* = 17.1, 1.7 Hz, 2H), 4.99 (dq, *J* = 10.2, 1.4 Hz, 2H), 4.25 (s, 1H), 4.01 – 3.87 (m, 1H), 3.47 – 3.32 (m, 1H), 2.15 – 1.99 (m, 4H), 1.62 – 1.55 (m, 2H), 1.55 – 1.45 (m, 2H).

^{13}C NMR (151 MHz, $CDCl_3$) δ 137.7, 130.8, 129.5, 128.9, 128.8, 115.5, 60.6, 54.0, 34.6, 29.7.

HRMS (ESI) calcd. for $[M+H]^+$ $(C_{16}H_{24}NO_2S)^+$ 294.1522, found 294.1521.

FT-IR (thin film) ν_{max} (cm^{-1}) = 3278, 2977, 1640, 1455, 1316, 1152, 1126, 909, 697.

[back to table of contents]

dimethyl (2*E*,9*E*)-6-((phenylmethyl)sulfonamido)undeca-2,9-dienedioate (7y)

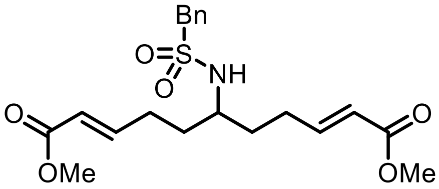

Compound **7y** was synthesised according to **General Procedure 1** from compound **S37** (291 mg, 1.00 mmol) and was purified by FCC (hexane/EtOAc 20% to 35%) to afford **7y** as a viscous brown oil (377 mg, 0.92 mmol, 93%).

^{1}H NMR (400 MHz, DMSO) δ 7.42 – 7.30 (m, 5H), 7.13 (d, *J* = 8.2 Hz, 1H), 6.88 (dt, *J* = 15.1, 6.8 Hz, 2H), 5.86 (d, *J* = 15.7 Hz, 2H), 4.32 (s, 2H), 3.64 (s, 6H), 3.24 (q, *J* = 6.8 Hz, 1H), 2.21 (q, *J* = 7.5 Hz, 4H), 1.63 – 1.44 (m, 4H).

^{13}C NMR (101 MHz, DMSO) δ 166.2, 149.3, 130.8, 130.5, 128.2, 128.0, 120.8, 59.0, 52.4, 51.2, 32.9, 27.7.

HRMS (ESI) calcd. for $[M+Na]^+$ $(C_{20}H_{27}NO_6SNa)^+$ 432.1451, found 432.1452.

FT-IR (thin film) ν_{max} (cm^{-1}) = 3276, 2950, 1719, 1656, 1495, 1318, 1279, 1151, 698.

Synthesis of Indoline, Isoindoline, and Tetrahydroquinoline Precursors

General Procedure 5

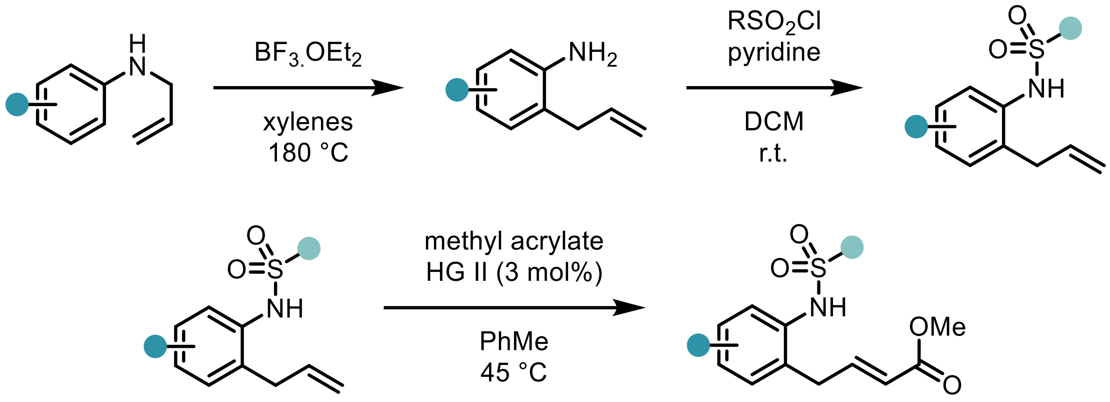

General Procedure 5A According to the literature procedure,[19] to a solution of the appropriate freshly distilled *N*-allyl aniline (1 equiv) in xylenes (0.5 M) was added $BF_3.OEt_2$ (1.2 equiv.). The reaction mixture was heated to 180 °C in a round-bottom flask fitted with a reflux condenser overnight, and once complete was cooled to 0 °C and basified with 1 M NaOH until pH 14. The aqueous phase was extracted with Et_2O (3 × 100 mL), the combined organics were dried over Na_2SO_4, filtered, and concentrated *in vacuo*. The crude residue was purified according to the specific experiment.

General Procedure 5B To a solution of the appropriate aniline (1 equiv.) and pyridine (2 equiv.) in DCM (0.2 M) at 0 °C was added the appropriate sulfonyl chloride (1.1 equiv.). The reaction mixture was allowed to warm to room temperature overnight. Once complete, the reaction was quenched with H_2O, extracted with CH_2Cl_2 three times and washed with brine. The combined organics were dried over Na_2SO_4, filtered, and concentrated *in vacuo*. The crude residue was purified by column of silica gel according to the specific experiment to give pure sulfonamide.

2-allylaniline (S38)

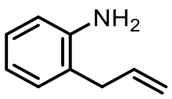

Compound **S38** was prepared according to **General Procedure 5A** from *N*-allyl aniline (3.40 mL, 25.0 mmol) and was purified by FCC (pentane/Et_2O 5% to 15%) to afford **S38** as a yellow oil (2.05 g, 15.4 mmol, 62%).

^{1}H NMR (400 MHz, $CDCl_3$) δ 7.07 (td, J = 7.5, 5.8 Hz, 2H), 6.76 (tt, J = 7.5, 1.2 Hz, 1H), 6.69 (d, J = 7.8 Hz, 1H), 6.04 – 5.89 (m, 1H), 5.19 – 5.07 (m, 2H), 3.67 (s, 2H), 3.32 (dd, J = 6.2, 1.9 Hz, 2H).

^{13}C NMR (101 MHz, $CDCl_3$) δ 144.9, 136.1, 130.3, 127.7, 124.1, 119.0, 116.2, 115.9, 36.6.

HRMS (ESI) calcd. for $[M+H]^+$ $(C_9H_{12}N)^+$ 134.0964, found 134.0969.

The data is consistent with the literature.[19]

[back to table of contents]

N-(2-allylphenyl)-1-phenylmethanesulfonamide (S39)

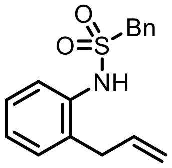

Compound **S39** was prepared according to **General Procedure 5B** from aniline **S38** (1.36 g, 10.2 mmol) and phenylmethane sulfonyl chloride (2.77 g, 15.0 mmol) and was purified by FCC (pentane/Et_2O 10% to 30%) and the impurities were triturated off with a minimum volume of pentane/Et_2O 1/1 to afford **S39** as an off white solid (999 mg, 3.48 mmol, 34%).

^{1}H NMR (600 MHz, $CDCl_3$) δ 7.52 (dd, *J* = 8.1, 1.2 Hz, 1H), 7.40 – 7.32 (m, 3H), 7.30 – 7.22 (m, 3H), 7.18 – 7.15 (m, 1H), 7.13 (td, *J* = 7.4, 1.2 Hz, 1H), 6.23 (s, 1H), 5.76 (ddt, *J* = 17.2, 10.1, 6.1 Hz, 1H), 5.04 (dq, *J* = 10.1, 1.5 Hz, 1H), 4.90 (dq, *J* = 17.2, 1.7 Hz, 1H), 4.38 (s, 2H), 3.11 (dt, *J* = 6.1, 1.7 Hz, 2H).

^{13}C NMR (151 MHz, $CDCl_3$) δ 135.9, 135.3, 131.0, 130.8, 129.3, 129.1, 129.0, 128.8, 128.2, 125.3, 120.3, 117.6, 58.1, 36.7.

HRMS (ESI) calcd. for $[M+H]^+$ $(C_{16}H_{18}NO_2S)^+$ 288.1053, found 288.1043.

FT-IR (thin film) ν_{max} (cm^{-1}) = 3293, 1585, 1334, 1154, 915, 696.

M.P. 54-56 °C.

methyl (*E*)-4-(2-((phenylmethyl)sulfonamido)phenyl)but-2-enoate (7z)

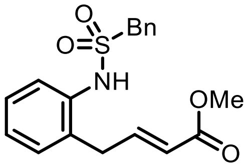

Compound **7z** was synthesised according to a modified version of **General Procedure 1** from compound **S39** (864 mg, 3.00 mmol). The reaction was quenched with potassium 2-isocyanoacetate (146 mg, 1.20 mmol) in MeOH (2 mL) and stirred at rt for 10 mins, before being concentrated *in vacuo*. The crude residue was purified by FCC (pentane/EtOAc 10% to 20%) to afford **7z** as a viscous yellow oil (453 mg, 1.31 mmol, 44%).

^{1}H NMR (400 MHz, $CDCl_3$) δ 7.46 (dd, *J* = 8.0, 1.2 Hz, 1H), 7.40 – 7.32 (m, 3H), 7.32 – 7.26 (m, 3H), 7.22 – 7.14 (m, 2H), 6.97 (dt, *J* = 15.7, 6.2 Hz, 1H), 5.93 (s, 1H), 5.64 (dt, *J* = 15.7, 1.8 Hz, 1H), 4.38 (s, 2H), 3.71 (s, 3H), 3.29 (dd, *J* = 6.2, 1.8 Hz, 2H).

^{13}C NMR (101 MHz, $CDCl_3$) δ 166.5, 145.4, 135.0, 131.0, 130.8, 129.8, 129.2, 129.1, 128.6, 128.6, 126.3, 123.0, 122.3, 58.3, 51.8, 34.1.

HRMS (ESI) calcd. for $[M+H]^+$ $(C_{18}H_{20}NO_4S)^+$ 346.1108, found 346.1095.

FT-IR (thin film) ν_{max} (cm^{-1}) = 1715, 1493, 1331, 1273, 1151, 1130, 913, 756, 730, 697.

[back to table of contents]

N-(2-allyl-4-bromophenyl)benzenesulfonamide (S40)

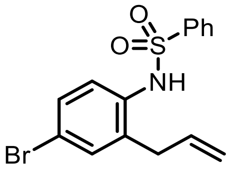

Compound **S40** was synthesised according to **General Procedure 5B** from the known aniline, 2-allyl-4-bromoaniline[20] (4.50 g, 21.4 mmol) and was purified by FCC (PhMe) to afford **S40** as a yellow solid (2.50 g, 7.08 mmol, 33%).

^{1}H NMR (400 MHz, $CDCl_3$) δ 7.73 – 7.65 (m, 2H), 7.54 (td, J = 7.3, 1.4 Hz, 1H), 7.43 (t, J = 7.9 Hz, 2H), 7.32 – 7.22 (m, 2H), 7.20 – 7.17 (m, 1H), 6.56 (s, 1H), 5.69 (ddt, J = 16.5, 10.2, 6.1 Hz, 1H), 5.09 (dd, J = 10.1, 1.6 Hz, 1H), 4.90 (dd, J = 17.2, 1.7 Hz, 1H), 2.93 (d, J = 6.1 Hz, 2H).

^{13}C NMR (101 MHz, $CDCl_3$) δ 139.5, 134.8, 134.7, 134.0, 133.4, 133.3, 130.8, 129.3, 127.1, 126.5, 120.0, 117.9, 35.8.

HRMS (ESI) calcd. for $[M+H]^+$ $(C_{15}H_{15}BrNO_2S)^+$ 352.0001, found 351.9998.

M.P. 83-85 °C.

methyl (E)-4-(5-bromo-2-(phenylsulfonamido)phenyl)but-2-enoate (7aa)

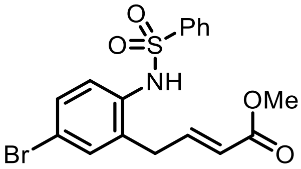

Compound **7aa** was synthesised according to **General Procedure 1** from compound **S40** (352 mg, 1.00 mmol) and was purified by FCC (pentane/EtOAc 30%) to afford **7aa** as a yellow oil (176 mg, 0.500 mmol, 50%).

^{1}H NMR (400 MHz, $CDCl_3$) δ 7.73 – 7.67 (m, 2H), 7.62 – 7.55 (m, 1H), 7.46 (dd, J = 8.4, 7.2 Hz, 2H), 7.29 (dd, J = 8.6, 2.3 Hz, 1H), 7.22 (d, J = 2.3 Hz, 1H), 7.05 (d, J = 8.5 Hz, 1H), 6.84 (dt, J = 15.7, 6.3 Hz, 1H), 6.65 (s, 1H), 5.62 (dt, J = 15.7, 1.8 Hz, 1H), 3.71 (s, 3H), 3.28 (dd, J = 6.4, 1.8 Hz, 2H).

^{13}C NMR (101 MHz, $CDCl_3$) δ 166.5, 145.1, 139.2, 135.4, 133.5, 133.4, 133.3, 131.2, 128.1, 127.3, 123.1, 121.0, 51.8, 33.6.

HRMS (ESI) calcd. for $[M+H]^+$ $(C_{17}H_{17}BrNO_4S)^+$ 410.0056, found 410.0053.

[back to table of contents]

N-(2-allyl-4-methoxyphenyl)benzenesulfonamide (S41)

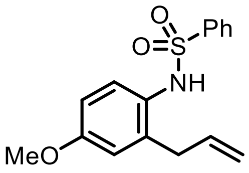

Compound **S41** was synthesised according to **General Procedure 5B** from the known aniline, 2-allyl-4-methoxyaniline[20] (483 mg, 3.00 mmol) and was purified by FCC (pentane/EtOAc 15%) to afford **S41** as a yellow solid (940 mg, 3.00 mmol, quant.).

^{1}H NMR (400 MHz, $CDCl_3$) δ 7.70 – 7.65 (m, 2H), 7.59 – 7.52 (m, 1H), 7.47 – 7.40 (m, 2H), 7.21 (d, *J* = 8.8 Hz, 1H), 6.72 (dd, *J* = 8.8, 3.0 Hz, 1H), 6.62 (d, *J* = 2.9 Hz, 1H), 6.29 (s, 1H), 5.72 (ddt, *J* = 17.1, 10.1, 6.2 Hz, 1H), 5.08 (dq, *J* = 10.2, 1.6 Hz, 1H), 4.91 (dq, *J* = 17.2, 1.7 Hz, 1H), 3.77 (s, 3H), 2.90 (dt, *J* = 6.2, 1.7 Hz, 2H).

^{13}C NMR (101 MHz, $CDCl_3$) δ 158.6, 139.9, 136.3, 135.7, 133.0, 129.1, 128.5, 127.2, 117.1, 115.9, 112.5, 55.5, 36.3.

HRMS (ESI) calcd. for $[M+H]^+$ $(C_{16}H_{18}NO_3S)^+$ 304.1002, found 304.1005.

M.P. 79-80 °C.

methyl (*E*)-4-(5-methoxy-2-(phenylsulfonamido)phenyl)but-2-enoate (7ab)

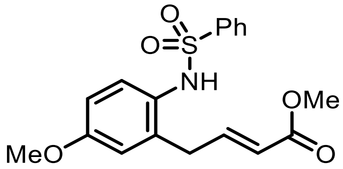

Compound **7ab** was synthesised according to **General Procedure 1** from compound **S41** (303 mg, 1.00 mmol) and was purified by FCC (pentane/EtOAc 30%) to afford **7ab** as a yellow oil (163 mg, 0.450 mmol, 45%).

^{1}H NMR (400 MHz, $CDCl_3$) δ 7.72 – 7.66 (m, 2H), 7.60 – 7.54 (m, 1H), 7.48 – 7.42 (m, 2H), 6.92 (d, *J* = 8.7 Hz, 1H), 6.89 (dt, *J* = 15.6, 6.4 Hz, 1H), 6.70 – 6.61 (m, 2H), 6.21 (s, 1H), 5.64 (dt, *J* = 15.7, 1.7 Hz, 1H), 3.76 (s, 3H), 3.71 (s, 3H), 3.29 (dd, *J* = 6.4, 1.8 Hz, 2H).

^{13}C NMR (101 MHz, $CDCl_3$) δ 166.7, 159.2, 146.2, 139.6, 136.8, 133.1, 129.7, 129.2, 127.4, 126.4, 122.6, 116.0, 112.9, 55.5, 51.7, 34.2.

HRMS (ESI) calcd. for $[M+H]^+$ $(C_{18}H_{20}NO_5S)^+$ 362.1057, found 362.1058.

[back to table of contents]

N-(2-allyl-4-methylphenyl)benzenesulfonamide (S42)

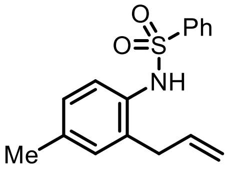

Compound **S42** was synthesised according to **General Procedure 5B** from known aniline, 2-allyl-4-methylaniline[20] (441 g, 3.00 mmol) and was purified by FCC (pentane/EtOAc 15%) to afford **S42** as a yellow solid (861 mg, 3.00 mmol, quant.).

^{1}H NMR (400 MHz, $CDCl_3$) δ 7.73 – 7.68 (m, 2H), 7.59 – 7.51 (m, 1H), 7.47 – 7.40 (m, 2H), 7.24 (d, *J* = 8.1 Hz, 1H), 7.00 (dd, *J* = 8.2, 2.1 Hz, 1H), 6.88 (d, *J* = 2.1 Hz, 1H), 6.44 (s, 1H), 5.75 (ddt, *J* = 17.2, 10.1, 6.1 Hz, 1H), 5.08 (dq, *J* = 10.1, 1.6 Hz, 1H), 4.92 (dq, *J* = 17.2, 1.7 Hz, 1H), 2.93 (dt, *J* = 6.2, 1.8 Hz, 2H), 2.28 (s, 3H).

^{13}C NMR (101 MHz, $CDCl_3$) δ 139.9, 136.7, 135.9, 133.0, 132.9, 132.1, 131.2, 129.1, 128.4, 127.2, 125.6, 117.0, 36.2, 21.0.

HRMS (ESI) calcd. for $[M+H]^+$ $(C_{16}H_{18}NO_2S)^+$ 288.1053, found 288.1050.

M.P. 78-79 °C.

methyl (*E*)-4-(5-methyl-2-(phenylsulfonamido)phenyl)but-2-enoate (7ac)

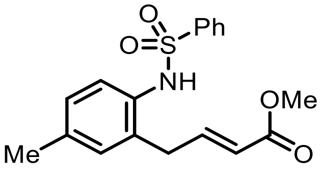

Compound **7ab** was synthesised according to **General Procedure 1** from compound **S42** (287 mg, 1.00 mmol) and was purified by FCC (pentane/EtOAc 30%) to afford **7ac** as a yellow oil (280 mg, 0.813 mmol, 81%).

^{1}H NMR (400 MHz, $CDCl_3$) δ 7.73 – 7.68 (m, 2H), 7.59 – 7.53 (m, 1H), 7.48 – 7.41 (m, 2H), 7.00 – 6.85 (m, 4H), 6.43 – 6.34 (m, 1H), 5.62 (dt, *J* = 15.6, 1.7 Hz, 1H), 3.70 (s, 3H), 3.29 (dd, *J* = 6.4, 1.8 Hz, 2H), 2.27 (s, 3H).

^{13}C NMR (101 MHz, $CDCl_3$) δ 166.7, 146.4, 139.6, 137.8, 133.7, 133.1, 131.3, 129.2, 128.7, 127.4, 127.1, 122.5, 51.7, 33.9, 21.1.

HRMS (ESI) calcd. for $[M+H]^+$ $(C_{18}H_{20}NO_4S)^+$ 346.1108, found 346.1105.

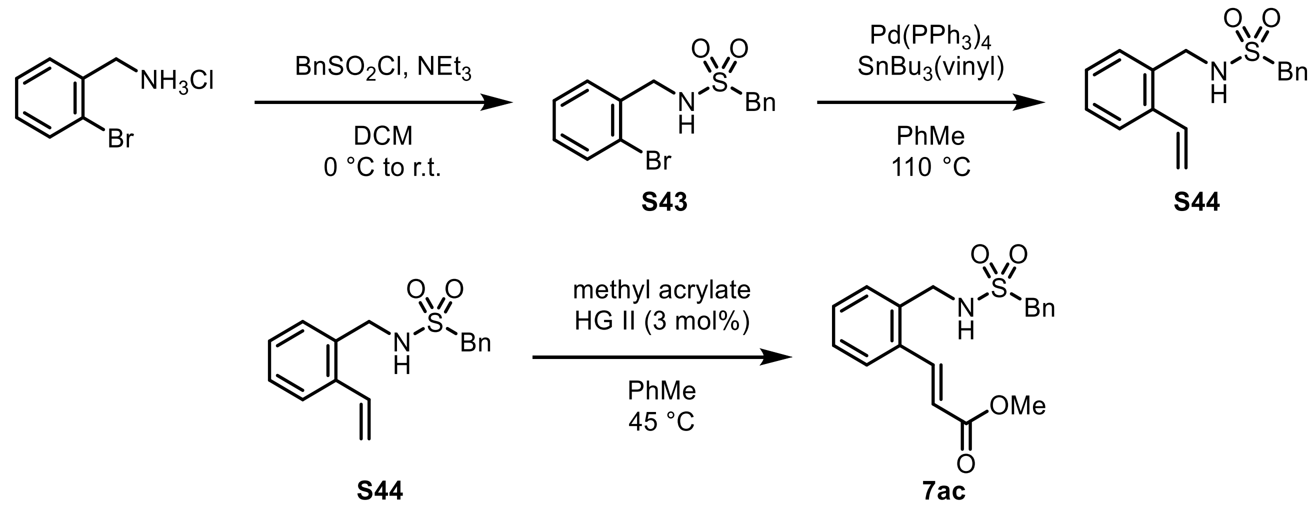

N-(2-bromobenzyl)-1-phenylmethanesulfonamide (S43)

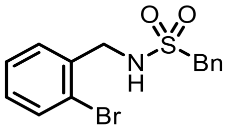

To a solution of 2-bromobenzylamine hydrochloride (2.24 g, 10.1 mmol) and NEt_3 (4.2 mL, 30.0 mmol) in DCM (50 mL) at 0 °C was added phenylmethane sulfonyl chloride (2.88 g, 15.0 mmol). The solution was allowed to warm to rt, and after 16 h was diluted with DCM (25 mL) and quenched with saturated aqueous $NaHCO_3$ (50 mL). The aqueous phase was extracted with DCM (3 × 50 mL), the combined organics were washed with brine (100 mL), dried over Na_2SO_4, filtered and concentrated *in vacuo*. The crude residue was purified by FCC (pentane/EtOAc 10% to 30%) to afford **S43** as a white solid (3.04 g, 8.94 mmol, 89%).

^{1}H NMR (600 MHz, $CDCl_3$) δ 7.58 (dd, J = 8.0, 1.2 Hz, 1H), 7.41 (dd, J = 7.6, 1.7 Hz, 1H), 7.37 – 7.31 (m, 4H), 7.24 – 7.19 (m, 3H), 4.65 (t, J = 6.5 Hz, 1H), 4.29 (d, J = 6.5 Hz, 2H), 4.16 (s, 2H).

^{13}C NMR (151 MHz, $CDCl_3$) δ 136.5, 133.2, 130.9, 130.7, 130.1, 129.1, 129.0, 129.0, 128.2, 124.1, 59.9, 48.0.

HRMS (ESI) calcd. for $[M+Na]^+$ $(C_{14}H_{14}BrNO_2SNa)^+$ 361.9821, found 361.9820.

FT-IR (thin film) ν_{max} (cm^{-1}) = 3285, 1440, 1329, 1152, 1126, 1026, 751, 697.

M.P. 58-60 °C.

[back to table of contents]

1-phenyl-*N*-(2-vinylbenzyl)methanesulfonamide (S44)

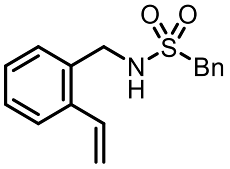

To a solution of compound **S43** (1.70 g, 5.00 mmol) and tetrakis(triphenylphosphine)palladium(0) (297 mg, 0.25 mmol) in toluene (18 mL) under a nitrogen atmosphere was added tributyl(vinyl)tin (1.6 mL, 5.5 mmol). The reaction mixture was refluxed at 110 °C for 24 h. Once complete, the reaction mixture was cooled to room temperature, filtered through a plug of silica/K_2CO_3 (9/1) with EtOAc, and subsequently concentrated *in vacuo*. The crude residue was purified by FCC (SiO_2/K_2CO_3 9/1, hexane/EtOAc 10% to 20%) to afford **S44** as a pale yellow solid (873 mg, 3.04 mmol, 61%).

^{1}H NMR (600 MHz, $CDCl_3$) δ 7.52 (dd, *J* = 7.8, 1.2 Hz, 1H), 7.37 – 7.31 (m, 4H), 7.30 – 7.26 (m, 2H), 7.25 – 7.21 (m, 2H), 6.88 (dd, *J* = 17.3, 11.0 Hz, 1H), 5.65 (dd, *J* = 17.3, 1.2 Hz, 1H), 5.36 (dd, *J* = 10.9, 1.2 Hz, 1H), 4.28 – 4.24 (m, 3H), 4.18 (s, 2H).

^{13}C NMR (151 MHz, $CDCl_3$) δ 137.3, 133.6, 133.5, 130.7, 129.7, 129.3, 129.0, 128.9, 128.9, 128.4, 126.7, 117.9, 59.5, 45.6.

HRMS (ESI) calcd. for $[M+H]^+$ $(C_{16}H_{18}NO_2S)^+$ 288.1053, found 288.1056.

FT-IR (thin film) ν_{max} (cm^{-1}) = 3291, 1411, 1326, 1152, 1126, 917, 775, 697.

M.P. 80-82 °C.

methyl (*E*)-3-(2-(((phenylmethyl)sulfonamido)methyl)phenyl)acrylate (7ad)

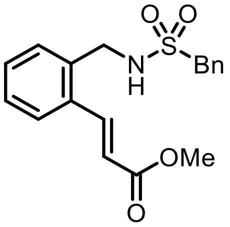

Compound **7ad** was synthesised according to a modified version of **General Procedure 1** from compound **S44** (717 mg, 2.50 mmol). The reaction was quenched with potassium 2-isocyanoacetate (120 mg, 1 mmol) in MeOH (3 mL) and stirred at rt for 10 mins, before being concentrated *in vacuo*. The crude residue was purified by FCC (pentane/EtOAc 20% to 30%) to afford **7ad** as a viscous yellow oil (722 mg, 2.09 mmol, 84%).

^{1}H NMR (400 MHz, $CDCl_3$) δ 7.89 (d, *J* = 15.8 Hz, 1H), 7.62 – 7.55 (m, 1H), 7.42 – 7.28 (m, 8H), 6.37 (d, *J* = 15.7 Hz, 1H), 4.43 (t, *J* = 5.9 Hz, 1H), 4.27 (d, *J* = 5.9 Hz, 2H), 4.21 (s, 2H), 3.82 (s, 3H).

^{13}C NMR (101 MHz, $CDCl_3$) δ 167.0, 140.8, 135.8, 133.5, 130.8, 130.6, 130.1, 129.1, 129.0, 129.0, 129.0, 127.2, 121.0, 59.8, 52.0, 45.2.

HRMS (ESI) calcd. for $[M+H]^+$ $(C_{18}H_{20}NO_4S)^+$ 346.1108, found 346.1106.

FT-IR (thin film) ν_{max} (cm^{-1}) = 1701, 1631, 1434, 1318, 1280, 1172, 1151, 976, 765, 697.

[back to table of contents]

methyl (*E*)-5-(2-(phenylsulfonamido)phenyl)pent-2-enoate (7ae)

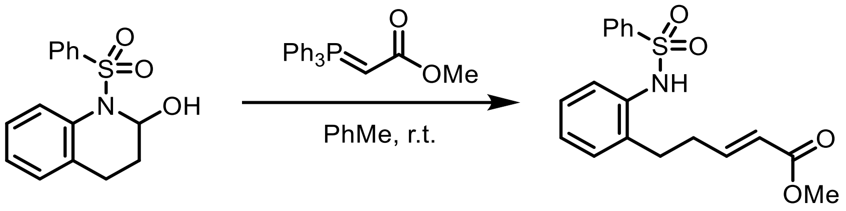

To a solution of known hemiaminal 1-(phenylsulfonyl)-1,2,3,4-tetrahydroquinolin-2-ol[21] (284 mg, 1.00 mmol) in PhMe (5.00 mL) at 0 °C was added methyl (Triphenylphosphoranylidene)acetate (668 mg, 2.00 mmol) at room temperature. The reaction mixture was stirred at same temperature overnight. Once complete, the reaction mixture was concentrated *in vacuo*. The crude residue was purified by FCC (pentane/Et_2O 25%) to afford **7ae** as a white solid (295 mg, 85.5 μmol, 86%).

^{1}H NMR (400 MHz, $CDCl_3$) δ 7.77 – 7.66 (m, 2H), 7.60 – 7.53 (m, 1H), 7.50 – 7.39 (m, 2H), 7.19 – 7.07 (m, 4H), 6.86 (dt, *J* = 15.7, 6.8 Hz, 1H), 6.47 (s, 1H), 5.73 (dt, *J* = 15.7, 1.6 Hz, 1H), 3.72 (s, 3H), 2.58 (dd, *J* = 9.0, 6.6 Hz, 2H), 2.33 – 2.24 (m, 2H).

^{13}C NMR (101 MHz, $CDCl_3$) δ 167.0, 147.7, 139.6, 135.8, 133.8, 133.2, 129.9, 129.2, 127.4, 127.3, 127.3, 126.2, 121.9, 51.7, 32.3, 29.3.

HRMS (ESI) calcd. for $[M+Na]^+$ $(C_{12}H_{18}N_2O_5SNa)^+$ 325.0829, found 325.0823.

M.P. 82-84 °C.

[back to table of contents]

Synthesis of Enantioenriched Pyrrolidines and Piperidines

General Procedure 6

A 1.75 mL vial fitted with a stirrer bar was charged with azide **A17** (7.4 mg, 0.02 mmol), tricyclohexylphosphine (5.6 mg, 0.02 mmol) and THF (0.5 mL). The vial was sealed under air and the resulting solution stirred at 40 °C for 24 h. After this time, the reaction mixture was concentrated under a stream of N_2 and subsequently *in vacuo*, affording the BIMP as a crude mixture. To this a stock solution of the appropriate substrate (0.1 mmol) in THP (0.1 M) was added. The reaction mixture was sealed under air and stirred for 24 h in a freezer set to a temperature of -22 °C. The reaction was quenched by passing down a short silica plug and eluted with EtOAc. The eluent was concentrated *in vacuo* and purified by FCC according to the specific experiment.

methyl (*R*)-2-(1-(phenylsulfonyl)pyrrolidin-2-yl)acetate (8a)

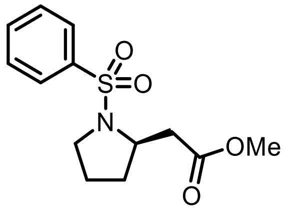

Pyrrolidine **8a** was prepared according to **General Procedure 6** from compound **7a** (27.7 mg, 97.8 µmol) and was purified by FCC (pentane/EtOAc 40%) to afford **8a** as a colourless oil (25.4 mg, 89.6 µmol, 92%, 93.5:6.5 e.r.).

^{1}H NMR (600 MHz, $CDCl_3$) δ 7.88 – 7.84 (m, 2H), 7.60 (tt, *J* = 7.5, 2.0 Hz, 1H), 7.56 – 7.51 (m, 2H), 3.98 (ddt, *J* = 9.9, 7.6, 4.0 Hz, 1H), 3.69 (s, 3H), 3.50 – 3.43 (m, 1H), 3.14 (dt, *J* = 10.0, 7.4 Hz, 1H), 3.08 (dd, *J* = 16.1, 3.9 Hz, 1H), 2.52 (dd, *J* = 16.1, 9.9 Hz, 1H), 1.85 – 1.72 (m, 2H), 1.66 (ddt, *J* = 10.6, 6.3, 2.9 Hz, 1H), 1.56 – 1.51 (m, 1H).

^{13}C NMR (151 MHz, $CDCl_3$) δ 171.8, 137.3, 132.9, 129.3, 127.7, 56.8, 51.8, 49.3, 41.3, 31.8, 23.9.

HRMS (ESI) calcd. for $[M+H]^+$ $(C_{13}H_{18}NO_4S)^+$ 284.0951, found 284.0937.

HPLC CHIRALPAK® OD, Hexane/IPA 95/5, 1 mL/min, λ = 220 nm, t_R (minor) = 21.2 min, t_R (major) = 28.5 min.

FT-IR (thin film) ν_{max} (cm^{-1}) = 2980, 1736, 1446, 1346, 1162, 723, 694.

$[\alpha]_D^{20}$ = +85.3 (c = 0.69, $CHCl_3$).

[back to table of contents]

ethyl (*R*)-2-(1-(benzylsulfonyl)pyrrolidin-2-yl)acetate (8b)

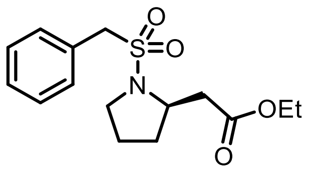

Pyrrolidine **8b** was prepared according to **General Procedure 6** from compound **7b** (31.9 mg, 102 μmol) and was purified by FCC (pentane/EtOAc 30%) to afford **8b** as a colourless oil (30.6 mg, 98.3 μmol, 96%, 95.5:4.5 e.r.).

^{1}H NMR (600 MHz, $CDCl_3$) δ 7.44 – 7.39 (m, 2H), 7.39 – 7.35 (m, 3H), 4.28 (d, *J* = 13.9 Hz, 1H), 4.25 (d, *J* = 13.9 Hz, 1H), 4.16 – 4.06 (m, 2H), 4.01 (ddt, *J* = 9.7, 7.9, 4.0 Hz, 1H), 3.22 (ddd, *J* = 9.8, 7.1, 5.1 Hz, 1H), 3.13 (dt, *J* = 9.8, 7.3 Hz, 1H), 2.77 (dd, *J* = 15.9, 4.2 Hz, 1H), 2.28 (dd, *J* = 15.9, 9.6 Hz, 1H), 2.02 (ddt, *J* = 12.6, 8.6, 7.4 Hz, 1H), 1.86 – 1.73 (m, 2H), 1.69 (dddd, *J* = 12.1, 6.7, 5.1, 3.9 Hz, 1H), 1.24 (t, *J* = 7.1 Hz, 3H).

^{13}C NMR (151 MHz, $CDCl_3$) δ 171.2, 130.9, 129.3, 128.9, 60.6, 56.8, 56.8, 49.3, 40.8, 31.9, 24.3, 14.3.

HRMS (ESI) calcd. for $[M+Na]^+$ $(C_{15}H_{21}NO_4SNa)^+$ 334.1084, found 334.1071.

HPLC CHIRALPAK® IA, Hexane/IPA 90/10, 1 mL/min, λ = 220 nm, t_R (minor) = 23.0 min, t_R (major) = 26.9 min.

FT-IR (thin film) ν_{max} (cm^{-1}) = 2980, 1729, 1378, 1335, 1202, 1150, 1128, 780, 699.

$[\alpha]_D^{20}$ = +38.4 (c = 0.82, $CHCl_3$).

tert-butyl (*R*)-2-(1-(phenylsulfonyl)pyrrolidin-2-yl)acetate (8c)

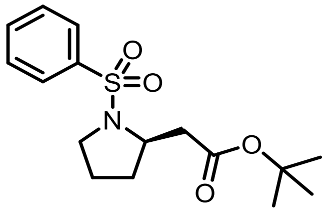

Pyrrolidine **8c** was prepared according to **General Procedure 6** from compound **7c** (32.8 mg, 101 μmol) and was purified by FCC (pentane/EtOAc 10% to 30%) to afford **8c** as a colourless oil (8.4 mg, 25.8 μmol, 26%, 90.5:9.5 e.r.).

^{1}H NMR (600 MHz, $CDCl_3$) δ 7.88 – 7.84 (m, 2H), 7.60 (tt, *J* = 7.4, 1.9 Hz, 1H), 7.56 – 7.50 (m, 2H), 3.95 (ddd, *J* = 10.5, 7.6, 3.8 Hz, 1H), 3.49 – 3.42 (m, 1H), 3.13 (ddd, *J* = 10.2, 7.6, 6.8 Hz, 1H), 3.02 (dd, *J* = 16.0, 3.7 Hz, 1H), 2.40 (dd, *J* = 16.0, 10.4 Hz, 1H), 1.84 – 1.71 (m, 2H), 1.68 – 1.62 (m, 1H), 1.57 – 1.49 (m, 1H), 1.45 (s, 9H).

^{13}C NMR (151 MHz, $CDCl_3$) δ 170.8, 137.3, 132.8, 129.2, 127.7, 81.0, 56.9, 49.3, 42.6, 31.7, 28.3, 23.9.

HRMS (ESI) calcd. for $[M+Na]^+$ $(C_{16}H_{23}NO_4SNa)^+$ 348.1240, found 348.1242.

HPLC CHIRALPAK® OD, Hexane/IPA 95/5, 1 mL/min, λ = 220 nm, t_R (minor) = 9.2 min, t_R (major) = 13.9 min.

FT-IR (thin film) ν_{max} (cm^{-1}) = 2979, 1726, 1349, 1163, 1094, 991, 758, 723, 693.

$[\alpha]_D^{20}$ = +56.5 (c = 0.17, $CHCl_3$).

[back to table of contents]

(*R*)-*N*,*N*-dimethyl-2-(1-(phenylsulfonyl)pyrrolidin-2-yl)acetamide (8d)

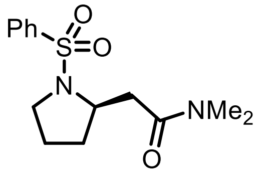

Pyrrolidine **8d** was prepared according to a modified version of **General Procedure 6** from compound **7d** (28.9 mg, 97.5 µmol) for 7 days and was purified by FCC (DCM/MeCN 10%-20%) to afford **8d** as a colourless oil (23.8 mg, 80.3 µmol, 82%, 97.5:2.5 er)

^{1}H NMR (400 MHz, $CDCl_3$) δ 7.89 – 7.82 (m, 2H), 7.63 – 7.56 (m, 1H), 7.56 – 7.50 (m, 2H), 4.07 – 3.97 (m, 1H), 3.53 – 3.44 (m, 1H), 3.14 (dd, *J* = 16.0, 3.1 Hz, 1H), 3.11 – 3.02 (m, 4H), 2.94 (s, 3H), 2.49 (dd, *J* = 15.9, 10.4 Hz, 1H), 1.87 – 1.68 (m, 3H), 1.55 – 1.46 (m, 1H).

^{13}C NMR (101 MHz, $CDCl_3$) δ 170.8, 136.9, 132.9, 129.3, 127.7, 57.6, 49.5, 40.9, 37.4, 35.3, 32.1, 23.8.

HRMS (ESI) calcd. for $[M+Na]^+$ $(C_{14}H_{20}N_2O_3SNa)^+$ 319.1087, found 319.1086.

HPLC CHIRALPAK® AS-H, Hexane/IPA 80/20, 1 mL/min, λ = 220 nm, t_R (major) = 25.0 min, t_R (minor) = 33.3 min.

FT-IR (thin film) ν_{max} (cm^{-1}) = 2964, 1644, 1339, 1163, 913, 735.

$[\alpha]_D^{20}$ = +63.9 (c = 1.29, $CHCl_3$).

methyl (*R*)-2-(1-(phenylsulfonyl)piperidin-2-yl)acetate (8e)

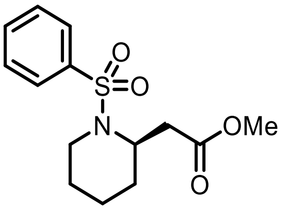

Piperidine **8e** was prepared according to a modified version of **General Procedure 6** at r.t. from compound **7e** (30.5 mg, 103 µmol) and was purified by FCC (pentane/EtOAc 30%) to afford **8e** as a colourless oil (28.5 mg, 95.8 µmol, 93%, 68.5:31.5 e.r.).

^{1}H NMR (600 MHz, $CDCl_3$) δ 7.85 – 7.81 (m, 2H), 7.55 (tt, *J* = 7.5, 2.1 Hz, 1H), 7.52 – 7.46 (m, 2H), 4.57 – 4.49 (m, 1H), 3.85 – 3.79 (m, 1H), 3.63 (s, 3H), 2.96 (ddd, *J* = 13.9, 13.0, 2.4 Hz, 1H), 2.63 (dd, *J* = 14.9, 9.2 Hz, 1H), 2.51 (dd, *J* = 14.9, 5.9 Hz, 1H), 1.58 – 1.46 (m, 5H), 1.38 – 1.27 (m, 1H).

^{13}C NMR (151 MHz, $CDCl_3$) δ 171.3, 141.3, 132.5, 129.2, 127.1, 52.0, 49.9, 41.2, 35.1, 27.9, 24.8, 18.4.

HRMS (ESI) calcd. for $[M+H]^+$ $(C_{14}H_{20}NO_4S)^+$ 298.1108, found 298.1120.

HPLC CHIRALPAK® IA, Hexane/IPA 90/10, 1 mL/min, λ = 220 nm, t_R (minor) = 15.2 min, t_R (major) = 24.1 min.

FT-IR (thin film) ν_{max} (cm^{-1}) = 2952, 1737, 1445, 1338, 1157, 931, 753, 693.

$[\alpha]_D^{20}$ = +2.91 (c = 0.55, $CHCl_3$).

methyl (*R*)-2-(1-(benzylsulfonyl)pyrrolidin-2-yl)acetate (8f)

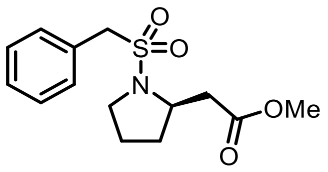

Pyrrolidine **8f** was prepared according to **General Procedure 5** from compound **7f** (30.1 mg, 101 µmol) and was purified by FCC (pentane/EtOAc 30%) to afford **8f** as a colourless oil (28.6 mg, 96.2 µmol, 95%, 93.5:6.5 e.r.).

^{1}H NMR (600 MHz, $CDCl_3$) δ 7.43 – 7.39 (m, 2H), 7.39 – 7.36 (m, 3H), 4.28 (d, *J* = 13.9 Hz, 1H), 4.25 (d, *J* = 13.9 Hz, 1H), 4.01 (ddt, *J* = 9.5, 7.9, 4.0 Hz, 1H), 3.65 (s, 3H), 3.23 (ddd, *J* = 9.8, 7.1, 5.1 Hz, 1H), 3.13 (dt, *J* = 9.8, 7.3 Hz, 1H), 2.76 (dd, *J* = 15.9, 4.2 Hz, 1H), 2.29 (dd, *J* = 15.9, 9.5 Hz, 1H), 2.07 – 1.98 (m, 1H), 1.86 – 1.73 (m, 2H), 1.69 (dddd, *J* = 12.1, 6.6, 5.1, 3.9 Hz, 1H).

^{13}C NMR (151 MHz, $CDCl_3$) δ 171.7, 130.9, 129.2, 128.9, 56.8, 56.7, 51.7, 49.3, 40.6, 31.9, 24.3.

HRMS (ESI) calcd. for $[M+H]^+$ $(C_{14}H_{20}NO_4S)^+$ 298.1108, found 298.1101.

HPLC CHIRALPAK® IA, Hexane/IPA 95/5, 1 mL/min, λ = 220 nm, t_R (minor) = 27.7 min, t_R (major)= 31.8 min.

FT-IR (thin film) ν_{max} (cm^{-1}) = 2980, 2364, 2256, 1736, 1335, 914, 781, 733.

$[\alpha]_D^{20}$ = +43.3 (c = 0.56, $CHCl_3$).

methyl (*R*)-2-(1-(thiophen-2-ylsulfonyl)pyrrolidin-2-yl)acetate (8g)

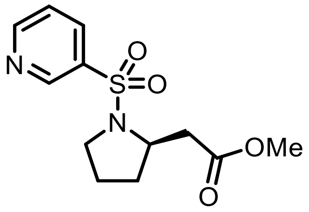

Pyrrolidine **8g** was synthesised according to **General Procedure 6** from compound **7g** (28.5 mg, 100 µmol) and was purified by FCC (pentane/Et_2O 30%) to afford **8g** as a yellow oil (16.5 mg, 57.9 µmol, 58%, 95:5 e.r.).

^{1}H NMR (400 MHz, $CDCl_3$) δ 9.07 (d, *J* = 1.6 Hz, 0H), 8.83 (dd, *J* = 4.8, 1.7 Hz, 1H), 8.15 (ddd, *J* = 8.0, 2.3, 1.6 Hz, 1H), 7.49 (ddd, *J* = 8.0, 4.8, 0.9 Hz, 1H), 3.97 (ddt, *J* = 9.6, 7.2, 4.0 Hz, 1H), 3.69 (s, 3H), 3.51 (ddd, *J* = 10.3, 6.6, 4.9 Hz, 1H), 3.20 – 3.10 (m, 1H), 3.05 (dd, *J* = 16.1, 4.0 Hz, 1H), 2.53 (dd, *J* = 16.1, 9.7 Hz, 1H), 1.92 – 1.65 (m, 3H), 1.64 – 1.55 (m, 1H).

^{13}C NMR (101 MHz, $CDCl_3$) δ 171.5, 153.5, 148.3, 135.3, 134.0, 124.0, 56.9, 51.9, 49.4, 41.1, 31.9, 23.9.

HRMS (ESI) calcd. for $[M+Na]^+$ $(C_{12}H_{16}N_2O_4S_2Na)^+$ 307.0723, found 307.0724.

HPLC CHIRALPAK® AD-H, Hexane/IPA 80/20, 1 mL/min, λ = 220 nm, t_R (minor) = 16.9 min, t_R (major) = 27.5 min.

$[\alpha]_D^{20}$ = +10.5 (c = 2.01, $CHCl_3$).

[back to table of contents]

methyl (*R*)-2-(1-(furan-2-ylsulfonyl)pyrrolidin-2-yl)acetate (8h)

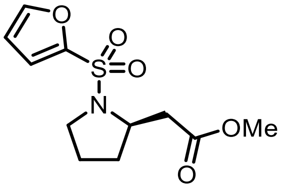

Pyrrolidine **8h** was synthesised according to **General Procedure 6** from compound **7h** (24.0 mg, 87.9 μmol) and was purified by FCC (pentane/Et_2O 20%) to afford **8h** as a yellow oil (18.9 mg, 69.2 μmol, 79%, 95:5 e.r.).

^{1}H NMR (400 MHz, $CDCl_3$) δ 7.57 – 7.54 (m, 1H), 7.05 (d, *J* = 3.5 Hz, 1H), 6.52 (dd, *J* = 3.5, 1.8 Hz, 1H), 4.11 (ddd, *J* = 10.2, 7.6, 4.0 Hz, 1H), 3.68 (s, 3H), 3.47 (dt, *J* = 10.4, 5.9 Hz, 1H), 3.34 (dt, *J* = 10.4, 7.1 Hz, 1H), 3.02 (dd, *J* = 16.1, 3.9 Hz, 1H), 2.52 (dd, *J* = 16.1, 10.1 Hz, 1H), 2.00 – 1.79 (m, 2H), 1.79 – 1.69 (m, 1H), 1.66 – 1.56 (m, 1H).

^{13}C NMR (101 MHz, $CDCl_3$) δ 171.6, 147.5, 146.4, 117.3, 111.3, 57.1, 51.8, 49.3, 41.0, 31.9, 24.2.

HRMS (ESI) calcd. for $[M+H]^+$ $(C_{11}H_{16}NO_5S)^+$ 274.0744, found 274.0753.

HPLC CHIRALPAK® OD, Hexane/IPA 90/10, 1 mL/min, λ = 220 nm, t_R (minor) = 16.0 min, t_R (major) = 28.8 min.

$[\boldsymbol{\alpha}]_{\boldsymbol{D}}^{\mathbf{20}}$ = +35.1 (c = 0.81, $CHCl_3$).

methyl (*R*)-2-(1-(thiophen-2-ylsulfonyl)pyrrolidin-2-yl)acetate (8i)

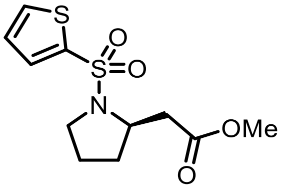

Pyrrolidine **8i** was synthesised according to **General Procedure 6** from compound **7i** (28.3 mg, 100 μmol) and was purified by FCC (pentane/Et_2O 20%) to afford **8i** as a yellow oil (17.3 mg, 61.2 μmol, 61%, 95:5 e.r.).

^{1}H NMR (400 MHz, $CDCl_3$) δ 7.64 – 7.59 (m, 2H), 7.14 (dd, *J* = 5.0, 3.7 Hz, 1H), 3.99 (ddt, *J* = 10.0, 7.6, 4.0 Hz, 1H), 3.69 (s, 3H), 3.55 – 3.47 (m, 1H), 3.26 – 3.17 (m, 1H), 3.07 (dd, *J* = 16.2, 3.9 Hz, 1H), 2.54 (dd, *J* = 16.2, 10.0 Hz, 1H), 1.90 – 1.77 (m, 2H), 1.75 – 1.65 (m, 1H), 1.64 – 1.50 (m, 2H).

^{13}C NMR (101 MHz, $CDCl_3$) δ 171.7, 137.1, 132.6, 131.9, 127.6, 57.1, 51.8, 49.6, 41.1, 31.8, 24.0.

HRMS (ESI) calcd. for $[M+Na]^+$ $(C_{11}H_{15}NO_4S_2Na)^+$ 312.0335, found 312.0339.

HPLC CHIRALPAK® IA, Hexane/IPA 98/2, 1 mL/min, λ = 220 nm, t_R (minor) = 41.5 min, t_R (major) = 46.4 min.

$[\boldsymbol{\alpha}]_{\boldsymbol{D}}^{\mathbf{20}}$ = +14.7 (c = 1.15, $CHCl_3$).

[back to table of contents]

methyl (*R*)-2-(1-((3,5-dimethylisoxazol-4-yl)sulfonyl)pyrrolidin-2-yl)acetate (8j)

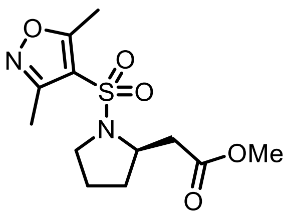

Pyrrolidine **8j** was synthesised according to **General Procedure 6** from compound **7j** (30.2 mg, 100 µmol) and was purified by FCC (pentane/Et_2O 50%) to afford **8j** as a yellow oil (30.2 mg, 100 µmol, quant., 95:5 e.r.).

^{1}H NMR (400 MHz, $CDCl_3$) δ 4.04 (ddt, *J* = 9.8, 7.5, 3.6 Hz, 1H), 3.67 (s, 3H), 3.45 – 3.38 (m, 1H), 3.18 (ddd, *J* = 9.8, 7.9, 6.5 Hz, 1H), 2.98 (dd, *J* = 15.9, 3.8 Hz, 1H), 2.67 (s, 3H), 2.48 (dd, *J* = 16.0, 9.9 Hz, 1H), 2.44 (s, 3H), 2.06 – 1.84 (m, 2H), 1.83 – 1.71 (m, 2H).

^{13}C NMR (101 MHz, $CDCl_3$) δ 173.8, 171.4, 158.0, 114.9, 56.2, 51.9, 48.9, 40.9, 32.0, 24.0, 13.2, 11.6.

HRMS (ESI) calcd. for $[M+Na]^+$ $(C_{12}H_{18}N_2O_5SNa)^+$ 325.0829, found 325.0824.

HPLC CHIRALPAK® AS-H, Hexane/IPA 95/5, 1 mL/min, λ = 220 nm, t_R (minor) = 24.9 min, t_R (major) = 26.3 min.

$[\boldsymbol{\alpha}]_{\boldsymbol{D}}^{\mathbf{20}}$ = +14.7 (c = 1.15, $CHCl_3$).

methyl (*R*)-2-(1-(mesitylsulfonyl)pyrrolidin-2-yl)acetate (8k)

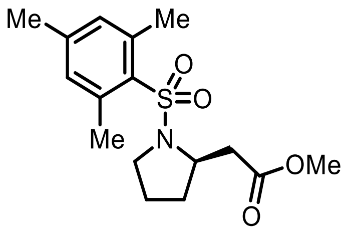

Pyrrolidine **8k** was synthesised according to **General Procedure 6** from compound **7k** (32.5 mg, 100 µmol) and was purified by FCC (pentane/Et_2O 50%) to afford **8k** as a white solid (20.0 mg, 61.5 µmol, 62%, 91:9 e.r.).

^{1}H NMR (400 MHz, $CDCl_3$) δ 6.97 – 6.92 (m, 2H), 4.29 (ddt, *J* = 10.1, 7.5, 3.7 Hz, 1H), 3.61 (s, 3H), 3.33 (dt, *J* = 9.8, 7.4 Hz, 1H), 3.13 (ddd, *J* = 9.8, 6.9, 5.0 Hz, 1H), 2.69 – 2.64 (m, 1H), 2.64 (s, 6H), 2.36 – 2.30 (m, 1H), 2.29 (s, 3H), 2.13 (ddt, *J* = 12.4, 9.2, 7.5 Hz, 1H), 1.95 – 1.81 (m, 2H), 1.81 – 1.71 (m, 1H).

^{13}C NMR (101 MHz, $CDCl_3$) δ 171.5, 142.8, 140.4, 133.0, 132.1, 55.5, 51.7, 47.9, 39.8, 32.0, 29.8, 24.1, 23.0, 21.1.

HRMS (ESI) calcd. for $[M+H]^+$ $(C_{16}H_{24}NO_4S)^+$ 326.1421, found 326.1426.

HPLC CHIRALPAK® IA, Hexane/IPA 99/1, 1 mL/min, λ = 220 nm, t_R (minor) = 38.1 min, t_R (major) = 46.9 min.

$[\boldsymbol{\alpha}]_{\boldsymbol{D}}^{\mathbf{20}}$ = +2.72 (c = 1.00, $CHCl_3$)

M.P. 82-84 °C

[back to table of contents]

methyl (*R*)-2-(1-((2,4,6-triisopropylphenyl)sulfonyl)pyrrolidin-2-yl)acetate (8l)

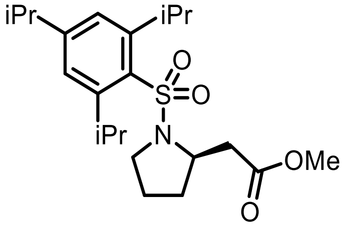

Pyrrolidine **8l** was synthesised according to **General Procedure 6** from compound **7l** (40.4 mg, 100 μmol) and was purified by FCC (pentane/Et_2O 50%) to afford **8l** as a colourless oil (30.8 mg, 76.2 μmol, 76%, 91:9 e.r.).

^{1}H NMR (400 MHz, $CDCl_3$) δ 7.15 (s, 2H), 4.41 – 4.32 (m, 1H), 4.24 (hept, *J* = 6.8 Hz, 2H), 3.63 (s, 3H), 3.34 (dt, *J* = 9.7, 7.3 Hz, 1H), 3.13 (ddd, *J* = 9.7, 7.0, 4.8 Hz, 1H), 2.89 (hept, *J* = 6.9 Hz, 1H), 2.74 (dd, *J* = 15.6, 3.8 Hz, 1H), 2.35 (dd, *J* = 15.6, 10.5 Hz, 1H), 2.15 (ddt, *J* = 12.4, 9.3, 7.5 Hz, 1H), 1.97 – 1.81 (m, 2H), 1.76 (dddd, *J* = 11.5, 6.5, 4.7, 3.4 Hz, 1H), 1.24 (d, *J* = 6.8 Hz, 18H).

^{13}C NMR (101 MHz, $CDCl_3$) δ 171.5, 153.4, 151.4, 131.6, 124.0, 55.3, 51.7, 48.1, 39.9, 34.3, 32.0, 29.8, 29.4, 25.1, 24.8, 24.1, 23.7.

HRMS (ESI) calcd. for $[M+Na]^+$ $(C_{22}H_{35}NO_4SNa)^+$ 432.2179, found 432.2180.

HPLC CHIRALPAK® IA, Hexane/IPA 99/1, 1 mL/min, λ = 220 nm, t_R (major) = 9.7 min, t_R (minor) = 11.7 min.

$[\alpha]_D^{20}$ = +1.49 (c = 1.54, $CHCl_3$).

[back to table of contents]

methyl (*R*)-2-(1-((5-(5-(trifluoromethyl)isoxazol-3-yl)thiophen-2-yl)sulfonyl)pyrrolidin-2-yl)acetate (8m)

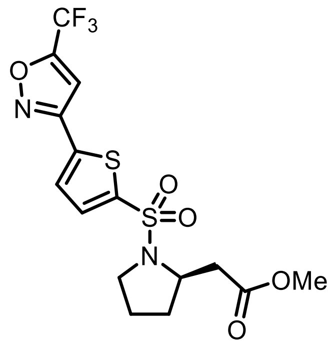

Pyrrolidine **8m** was synthesised according to **General Procedure 6** from compound **7m** (42.9 mg, 100 μmol) and was purified by FCC (pentane/Et_2O 30%) to afford **8m** as a yellow oil (34.0 mg, 80.2 μmol, 80%, 91:8 e.r.).

^{1}H NMR (400 MHz, $CDCl_3$) δ 7.62 (dd, *J* = 3.9, 0.8 Hz, 1H), 7.51 (d, *J* = 3.9 Hz, 1H), 7.02 – 6.98 (m, 1H), 4.01 (ddt, *J* = 9.7, 7.6, 3.9 Hz, 1H), 3.70 (s, 3H), 3.54 (ddd, *J* = 10.9, 6.5, 4.8 Hz, 1H), 3.25 (dt, *J* = 10.3, 7.3 Hz, 1H), 3.05 (dd, *J* = 16.2, 3.9 Hz, 1H), 2.55 (dd, *J* = 16.2, 9.9 Hz, 1H), 1.95 – 1.82 (m, 2H), 1.78 – 1.70 (m, 1H), 1.64 (tdd, *J* = 8.3, 6.3, 4.2 Hz, 1H).

^{13}C NMR (101 MHz, $CDCl_3$) δ 171.51, 160.08 (q, *J* = 43.1 Hz), 156.91, 140.12, 134.61, 132.58, 128.39, 117.63 (q, *J* = 270.8 Hz), 103.66, 103.64, 57.32, 51.87, 49.69, 41.02, 31.84, 23.96.

^{19}F NMR (376 MHz, $CDCl_3$) δ -64.23 (s, 3F).

HRMS (ESI) calcd. for $[M+H]^+$ $(C_{15}H_{16}F_3N_2O_5S_2)^+$ 425.0447, found 425.0453.

HPLC CHIRALPAK® IA, Hexane/IPA 90/10, 1 mL/min, λ = 220 nm, t_R (minor) = 17.8 min, t_R (major) = 22.1 min.

$[\alpha]_D^{20}$ = +40.6 (c = 1.70, $CHCl_3$).

[back to table of contents]

methyl (*R*)-2-(2-(benzylsulfonyl)-2-azaspiro[4.5]decan-3-yl)acetate (8n)

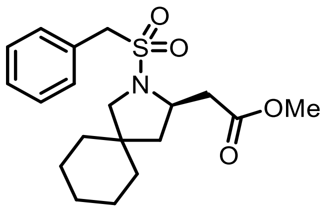

Pyrrolidine **8n** was synthesised according to **General Procedure 6** from compound **7n** (37.6 mg, 103 μmol) and was purified by FCC (pentane/EtOAc 30%) to afford **8n** as a white solid (34.3 mg, 94.0 μmol, 91%, 92:8 e.r.).

^{1}H NMR (600 MHz, $CDCl_3$) δ 7.41 – 7.35 (m, 5H), 4.23 (s, 2H), 4.22 – 4.17 (m, 1H), 3.66 (s, 3H), 3.25 (dd, *J* = 10.2, 1.3 Hz, 1H), 2.79 (d, *J* = 10.2 Hz, 1H), 2.72 (dd, *J* = 15.7, 4.5 Hz, 1H), 2.19 (dd, *J* = 15.7, 8.7 Hz, 1H), 2.07 (ddd, *J* = 12.9, 7.5, 1.2 Hz, 1H), 1.50 – 1.23 (m, 11H).

^{13}C NMR (151 MHz, $CDCl_3$) δ 171.8, 131.0, 129.4, 128.8, 128.8, 58.8, 58.5, 55.8, 51.7, 44.3, 42.2, 40.7, 36.4, 34.5, 26.0, 23.8, 22.9.

HRMS (ESI) calcd. for $[M+H]^+$ $(C_{26}H_{28}NO_4S)^+$ 450.1734, found 450.1732.

HPLC CHIRALPAK® AD-H, Hexane/IPA 95/5, 1 mL/min, λ = 220 nm, t_R (minor) = 26.9 min, t_R (major) = 30.5 min.

FT-IR (thin film) ν_{max} (cm^{-1}) = 2930, 1735, 1495, 1330., 1149, 1128, 742, 698.

M.P. 44-46 °C.

$[\alpha]_D^{20}$ = +37.8 (c = 0.96, $CHCl_3$).

[back to table of contents]

methyl (*R*)-2-(2-(benzylsulfonyl)-8-oxa-2-azaspiro[4.5]decan-3-yl)acetate (8o)

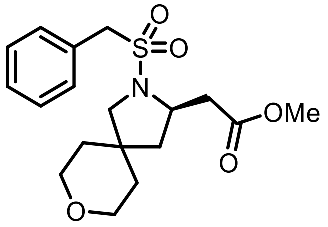

Pyrrolidine **8o** was prepared according to **General Procedure 6** from compound **7o** (35.1 mg, 95.5 µmol) and was purified by FCC (pentane/EtOAc 40%) to afford **8o** as a white solid (31.6 mg, 86.0 µmol, 90%, 91.5: 8.5 e.r.).

^{1}H NMR (600 MHz, $CDCl_3$) δ 7.42 – 7.37 (m, 5H), 4.29 – 4.21 (m, 3H), 3.67 (s, 3H), 3.70 – 3.60 (m, 2H), 3.54 (ddd, *J* = 11.8, 6.6, 5.0 Hz, 1H), 3.44 (ddd, *J* = 11.8, 8.4, 3.3 Hz, 1H), 3.33 (dd, *J* = 10.4, 1.4 Hz, 1H), 2.79 (d, *J* = 10.4 Hz, 1H), 2.68 (dd, *J* = 15.8, 4.6 Hz, 1H), 2.23 – 2.14 (m, 2H), 1.58 – 1.51 (m, 1H), 1.50 – 1.43 (m, 4H).

^{13}C NMR (151 MHz, $CDCl_3$) δ 171.6, 131.0, 129.2, 128.9, 128.9, 65.3, 64.9, 59.2, 58.1, 55.4, 51.8, 43.9, 40.4, 40.0, 35.8, 34.3.

HRMS (ESI) calcd. for $[M+H]^+$ $(C_{18}H_{26}NO_5S)^+$ 368.1526, found 368.1529.

HPLC CHIRALCEL® OD, Hexane/IPA 80/20, 1 mL/min, λ = 220 nm, t_R (major) = 22.7 min, t_R (minor) = 28.5 min.

FT-IR (thin film) ν_{max} (cm^{-1}) = 3699, 1733, 1438, 1329, 1148, 1106, 913, 743.

M.P. = 80-82 °C.

$[\alpha]_D^{20}$ = +31.8 (c = 0.88, $CHCl_3$).

[back to table of contents]

tert-butyl (*R*)-2-(benzylsulfonyl)-3-(2-methoxy-2-oxoethyl)-2,8-diazaspiro[4.5]decane-8-carboxylate (8p)

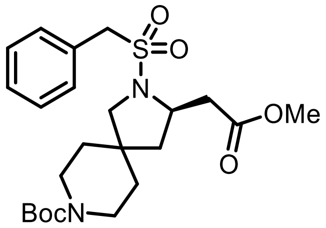

Pyrrolidine **8p** was prepared according to **General Procedure 6** from compound **7p** (44.2 mg, 94.7 µmol) and was purified by FCC (pentane/EtOAc 40%) to afford **8p** as an off white solid (33.1 mg, 70.9 µmol, 75%, 89:11 e.r.).

^{1}H NMR (600 MHz, DMSO, 373 K) δ 7.44 – 7.41 (m, 2H), 7.40 – 7.34 (m, 3H), 4.42 (d, *J* = 13.8 Hz, 1H), 4.39 (d, *J* = 13.8 Hz, 1H), 4.16 – 4.09 (m, 1H), 3.61 (s, 3H), 3.41 – 3.31 (m, 3H), 3.21 (dddd, *J* = 28.3, 13.5, 7.6, 4.3 Hz, 2H), 3.01 (d, *J* = 10.5 Hz, 1H), 2.73 (dd, *J* = 15.6, 4.4 Hz, 1H), 2.36 (dd, *J* = 15.6, 8.8 Hz, 1H), 2.14 – 2.08 (m, 1H), 1.55 (dd, *J* = 13.0, 7.3 Hz, 1H), 1.48 – 1.44 (m, 2H), 1.44 – 1.37 (m, 2H), 1.41 (s, 9H).

^{13}C NMR (151 MHz, DMSO, 373 K) δ 170.2, 153.5, 130.3, 129.3, 127.7, 127.5, 78.1, 56.8, 56.3, 54.6, 50.6, 42.0, 40.6, 40.2, 40.1, 34.2, 33.1, 27.6.

HRMS (ESI) calcd. for $[M+Na]^+$ $(C_{23}H_{34}N_2O_6SNa)^+$ 489.2030, found 489.2049.

HPLC CHIRALPAK® AD-H, Hexane/IPA 80/20, 1 mL/min, λ = 220 nm, t_R (major) = 14.4 min, t_R (minor) = 19.0 min.

FT-IR (thin film) ν_{max} (cm^{-1}) = 2977, 1735, 1687, 1424, 1330, 1247, 1157, 913.

M.P. = 98-100 °C.

$[\alpha]_D^{20}$ = +28.0 (c = 0.97, $CHCl_3$).

[back to table of contents]

methyl (*R*)-2-(2-(benzylsulfonyl)-2-azaspiro[4.4]nonan-3-yl)acetate (8q)

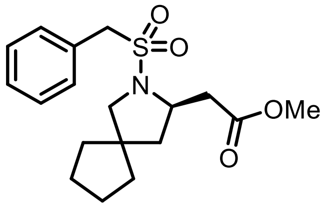

Pyrrolidine **8q** was prepared according to **General Procedure 6** from compound **7q** (35.0 mg, 99.6 µmol) and was purified by FCC (pentane/EtOAc 30%) to afford **8q** as a colourless oil (32.6 mg, 92.8 µmol, 93%, 93.5:6.5 e.r.).

^{1}H NMR (600 MHz, $CDCl_3$) δ 7.42 – 7.35 (m, 5H), 4.24 (s, 2H), 4.17 (dtd, *J* = 9.1, 7.3, 4.4 Hz, 1H), 3.65 (s, 3H), 3.09 (dd, *J* = 9.7, 1.1 Hz, 1H), 2.90 (d, *J* = 9.8 Hz, 1H), 2.77 (dd, *J* = 15.7, 4.4 Hz, 1H), 2.24 (dd, *J* = 15.7, 9.2 Hz, 1H), 2.09 (ddd, *J* = 12.6, 7.5, 1.1 Hz, 1H), 1.66 – 1.53 (m, 7H), 1.51 – 1.40 (m, 2H).

^{13}C NMR (151 MHz, $CDCl_3$) δ 171.8, 131.0, 129.3, 128.8, 128.8, 60.0, 58.2, 56.7, 51.7, 49.3, 44.6, 40.5, 37.0, 36.5, 25.0, 24.6.

HRMS (ESI) calcd. for $[M+Na]^+$ $(C_{18}H_{25}NO_4SNa)^+$ 374.1397, found 374.1398.

HPLC CHIRALPAK® AD-H, Hexane/IPA 90/10, 1 mL/min, λ = 220 nm, 41 t_R (minor) = 14.8 min, t_R (major) = 16.5 min.

FT-IR (thin-film) ν_{max} (cm^{-1}) = 2957, 1734, 1329, 1150, 1128, 1016, 914, 740, 669.

$[\alpha]_D^{20}$ = +35.6 (c = 0.95, $CHCl_3$).

methyl (*R*)-2-(6-(benzylsulfonyl)-6-azaspiro[3.4]octan-7-yl)acetate (8r)

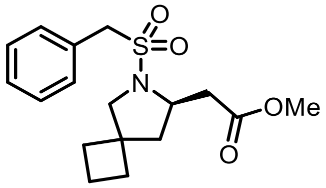

Pyrrolidine **8r** was prepared according to **General Procedure 6** from compound **7r** (33.4 mg, 99.1 µmol) and was purified by FCC (pentane/EtOAc 30%) to afford **8r** as a colourless oil (32.7 mg, 97.0 µmol, 98%, 93.5:6.5 e.r.).

^{1}H NMR (600 MHz, $CDCl_3$) δ 7.42 – 7.36 (m, 5H), 4.25 (d, *J* = 13.8 Hz, 1H), 4.22 (d, *J* = 13.8 Hz, 1H), 4.07 (dddd, *J* = 9.7, 7.6, 5.5, 4.5 Hz, 1H), 3.66 (s, 3H), 3.17 (q, *J* = 9.8 Hz, 2H), 2.76 (dd, *J* = 15.8, 4.5 Hz, 1H), 2.24 (dd, *J* = 15.8, 9.4 Hz, 1H), 2.12 (dd, *J* = 12.7, 7.6 Hz, 1H), 1.98 – 1.94 (m, 2H), 1.91 (ddd, *J* = 8.4, 5.6, 2.6 Hz, 2H), 1.88 – 1.81 (m, 2H), 1.71 (dd, *J* = 12.8, 5.5 Hz, 1H).

^{13}C NMR (151 MHz, $CDCl_3$) δ 171.7, 131.0, 129.3, 128.9, 128.9, 60.1, 57.5, 56.5, 51.8, 44.5, 44.1, 40.6, 31.8, 31.6, 16.4.

HRMS (ESI) calcd. for $[M+H]^+$ $(C_{17}H_{24}NO_4S)^+$ 338.1421, found 338.1415.

HPLC CHIRALPAK® IA, Hexane/IPA 95/5, 1 mL/min, λ = 220 nm, t_R (minor) = 20.9 min, t_R (major) = 24.1 min.

FT-IR (thin film) ν_{max} (cm^{-1}) = 2981, 1733, 1334, 1149, 913, 743, 634.

$[\alpha]_D^{20}$ = +21.5 (c = 0.87, $CHCl_3$).

[back to table of contents]

methyl (*R*)-2-(1-(benzylsulfonyl)-4,4-diphenylpyrrolidin-2-yl)acetate (8s)

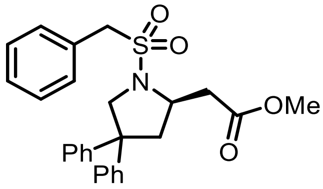

Pyrrolidine **8s** was synthesised according to **General Procedure 6** from compound **7s** (44.7 mg, 99.3 µmol) and was purified by FCC (pentane/EtOAc 30%) to afford **8s** as a white solid (44.3 mg, 98.4 µmol, 99%, 94.5:5.5 e.r.).

^{1}H NMR (600 MHz, $CDCl_3$) δ 7.39 – 7.36 (m, 2H), 7.34 – 7.30 (m, 5H), 7.31 – 7.27 (m, 4H), 7.19 (ddt, *J* = 8.4, 7.6, 1.2 Hz, 4H), 4.19 – 4.10 (m, 2H), 3.98 – 3.91 (m, 2H), 3.76 (d, *J* = 13.7 Hz, 1H), 3.65 (s, 3H), 3.19 (ddd, *J* = 13.0, 7.0, 1.6 Hz, 1H), 2.91 (dd, *J* = 16.3, 4.0 Hz, 1H), 2.36 (dd, *J* = 13.0, 7.7 Hz, 1H), 2.18 (dd, *J* = 16.3, 9.5 Hz, 1H).

^{13}C NMR (151 MHz, $CDCl_3$) δ 171.8, 145.1, 144.3, 130.9, 129.0, 129.0, 128.9, 128.8, 128.8, 127.2, 126.9, 126.9, 126.7, 59.0, 56.9, 56.3, 53.3, 51.8, 43.9, 40.2.

HRMS (ESI) calcd. for $[M+Na]^+$ $(C_{19}H_{27}NO_4SNa)^+$ 388.1553, found 388.1555.

HPLC CHIRALPAK® IA, Hexane/IPA 80/20, 1 mL/min, λ = 220 nm, t_R (major) = 12.8 min, t_R (minor) = 16.6 min.

FT-IR (thin film) ν_{max} (cm^{-1}) = 3031, 1732, 1495, 1332, 1200, 1152, 1124, 698.

M.P. = 40- 42 °C.

$[\alpha]_D^{20}$ = -18.5 (c = 1.27, $CHCl_3$).

[back to table of contents]

methyl (*R*)-2-(5,5-diphenyl-1-(phenylsulfonyl)piperidin-2-yl)acetate (8t)

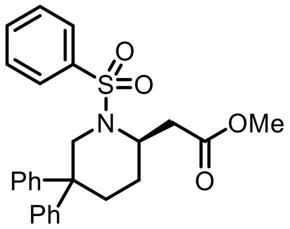

Piperidine **8t** was synthesised according to **General Procedure 6** from compound **7t** (44.0 mg, 100 µmol) and was purified by FCC (pentane/Et_2O 2%) to afford **8t** as a white solid (33.4 mg, 75.9 µmol, 76%, 86:14 e.r.).

^{1}H NMR (400 MHz, $CDCl_3$) δ 7.65 – 7.58 (m, 2H), 7.53 – 7.46 (m, 1H), 7.41 – 7.35 (m, 4H), 7.27 – 7.21 (m, 4H), 7.20 – 7.12 (m, 4H), 4.70 (dd, *J* = 13.4, 2.5 Hz, 1H), 4.25 (ddd, *J* = 10.9, 5.6, 2.4 Hz, 1H), 3.62 (s, 3H), 3.14 (d, *J* = 13.4 Hz, 1H), 2.70 (dd, *J* = 15.3, 10.7 Hz, 1H), 2.47 (dq, *J* = 14.3, 3.3 Hz, 1H), 2.42 – 2.32 (m, 1H), 2.27 (td, *J* = 13.9, 3.5 Hz, 1H), 1.78 – 1.67 (m, 1H), 1.60 – 1.52 (m, 1H).

^{13}C NMR (101 MHz, $CDCl_3$) δ 171.4, 147.2, 143.5, 139.7, 132.8, 129.2, 128.7, 128.6, 128.0, 127.7, 126.7, 126.5, 126.3, 52.0, 49.4, 48.7, 46.0, 33.1, 29.0, 24.8.

HRMS (ESI) calcd. for $[M+Na]^+$ $(C_{26}H_{27}NO_4SNa)^+$ 472.1553, found 472.1553.

HPLC CHIRALPAK® OD, Hexane/IPA 95/5, 1 mL/min, λ = 220 nm, t_R (major) = 22.8 min, t_R (minor) = 30.8 min.

$[\alpha]_D^{20}$ = +22.1 (c = 1.67, $CHCl_3$).

M.P. 116-118 °C.

[back to table of contents]

methyl (*R*)-2-(1-(benzylsulfonyl)-5,5-dimethylpyrrolidin-2-yl)acetate (8u)

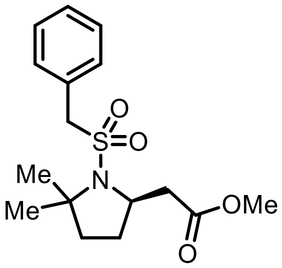

Pyrrolidine **8u** was prepared according to **General Procedure 6** from compound **7u** (32.4 mg, 99.6 μmol) and was purified by FCC (pentane/EtOAc 30%) to afford **8u** as a white solid (29.2 mg, 89.7 μmol, 90%, 93:7 e.r.).

^{1}H NMR (600 MHz, $CDCl_3$) δ 7.43 (dd, *J* = 7.3, 2.4 Hz, 2H), 7.40 – 7.36 (m, 3H), 4.26 (d, *J* = 1.0 Hz, 2H), 3.90 (m, 1H), 3.64 (s, 3H), 2.94 (dd, *J* = 16.1, 3.6 Hz, 1H), 2.38 (dd, *J* = 16.1, 10.5 Hz, 1H), 2.02 – 1.86 (m, 2H), 1.79 (ddd, *J* = 12.2, 6.1, 2.2 Hz, 1H), 1.62 (ddd, *J* = 11.2, 6.3, 3.5 Hz, 1H), 1.51 (s, 3H), 1.36 (s, 3H).

^{13}C NMR (151 MHz, $CDCl_3$) δ 171.8, 130.9, 129.6, 128.8, 128.8, 65.7, 59.9, 58.6, 51.7, 40.2, 31.6, 28.2, 26.7.

HRMS (ESI) calcd. for $[M+H]^+$ $(C_{16}H_{24}NO_4S)^+$ 326.1421, found 326.1412.

HPLC CHIRALCEL® OD, Hexane/IPA 90/10, 1 mL/min, λ = 220 nm, t_R (major) = 13.6 min, t_R (minor) = 22.0 min.

FT-IR (thin-film) ν_{max} (cm^{-1}) = 3063, 1733, 1541, 1336, 1148, 913, 668.

M.P. = 72-74 °C.

$[\alpha]_D^{20}$ = +6.77 (c = 0.89, $CHCl_3$).

[back to table of contents]

methyl (*R*)-2-(5-(benzylsulfonyl)-5-azaspiro[3.4]octan-6-yl)acetate (8v)

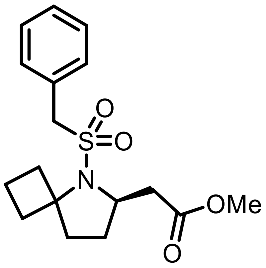

Pyrrolidine **8v** was prepared according to **General Procedure 6** from compound **7v** (34.6 mg, 102 μmol) and was purified by FCC (pentane/EtOAc 30%) to afford **8v** as a white solid (31.4 mg, 93.1 μmol, 90%, 92.5:7.5 e.r.).

^{1}H NMR (600 MHz, $CDCl_3$) δ 7.41 – 7.34 (m, 5H), 4.26 (d, *J* = 13.7 Hz, 1H), 4.22 (d, *J* = 13.7 Hz, 1H), 3.86 (ddt, *J* = 10.7, 7.4, 3.6 Hz, 1H), 3.64 (s, 3H), 2.76 (q, *J* = 10.3 Hz, 1H), 2.28 (dd, *J* = 16.0, 10.1 Hz, 1H), 2.10 – 2.00 (m, 2H), 1.97 – 1.90 (m, 1H), 1.90 – 1.83 (m, 1H), 1.76 – 1.67 (m, 2H), 1.63 – 1.54 (m, 2H).

^{13}C NMR (151 MHz, $CDCl_3$) δ 171.7, 131.0, 129.3, 128.9, 128.9, 66.6, 59.8, 58.8, 51.7, 40.9, 38.1, 37.5, 33.9, 28.2, 14.6.

HRMS (ESI) calcd. for $[M+H]^+$ $(C_{17}H_{24}NO_4S)^+$ 338.1421, found 338.1417.

HPLC CHIRALPAK® IA, Hexane/IPA 98/2, 1 mL/min, λ = 220 nm, t_R (minor) = 33.4 min, t_R (major) = 40.9 min.

FT-IR (thin film) ν_{max} (cm^{-1}) = 2980, 1733, 1541, 1339, 1200, 1149, 1015, 913, 782.

M.P. = 108-110 °C.

$[\alpha]_D^{20}$ = +5.25 (c = 0.88, $CHCl_3$).

[back to table of contents]

methyl (*R*)-2-(1-(benzylsulfonyl)-8-oxa-1-azaspiro[4.5]decan-2-yl)acetate (8w)

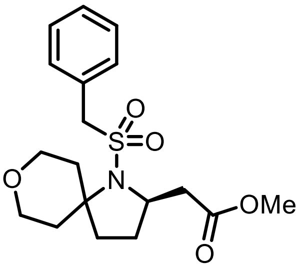

Pyrrolidine **8w** was prepared according to **General Procedure 6** from compound **7w** (35.0 mg, 95.3 µmol) and was purified by FCC (pentane/EtOAc, 40% to 50%) to afford **8w** as an off white solid (23.2 mg, 63.1 µmol, 66%, 93.5:6.5 e.r.).

^{1}H NMR (600 MHz, $CDCl_3$) δ 7.44 – 7.40 (m, 2H), 7.40 – 7.35 (m, 3H), 4.31 (d, *J* = 13.7 Hz, 1H), 4.24 (d, *J* = 13.7 Hz, 1H), 3.94 – 3.86 (m, 2H), 3.65 (s, 3H), 3.43 (td, *J* = 12.2, 2.3 Hz, 1H), 3.33 (ddd, *J* = 12.9, 11.8, 2.0 Hz, 1H), 3.00 (ddd, *J* = 16.2, 3.7, 1.1 Hz, 1H), 2.79 (td, *J* = 13.0, 5.0 Hz, 1H), 2.44 (tdd, *J* = 12.4, 5.2, 1.5 Hz, 1H), 2.36 (dd, *J* = 16.1, 10.6 Hz, 1H), 2.28 – 2.22 (m, 1H), 1.87 (ttd, *J* = 12.0, 6.9, 1.2 Hz, 1H), 1.79 (tdd, *J* = 12.7, 6.2, 1.6 Hz, 1H), 1.71 – 1.65 (m, 1H), 1.43 (dq, *J* = 13.1, 2.1 Hz, 1H), 1.12 (dq, *J* = 12.5, 2.1 Hz, 1H).

^{13}C NMR (151 MHz, $CDCl_3$) δ 171.6, 131.0, 129.4, 128.9, 128.9, 67.1, 66.3, 66.3, 60.6, 58.4, 51.8, 41.6, 40.3, 34.2, 33.8, 28.2.

HRMS (ESI) calcd. for $[M+H]^+$ $(C_{18}H_{26}NO_5S)^+$ 368.1526, found 368.1540.

HPLC CHIRALPAK® IA, Hexane/IPA 90/10, 1 mL/min, λ = 220 nm, t_R (major) = 34.7 min, t_R (minor) = 45.1 min.

FT-IR (thin film) ν_{max} (cm^{-1}) = 2951, 1734, 1456, 1337, 1146, 1105, 720, 699.

M.P. = 162-164 °C.

$[\alpha]_D^{20}$ = +4.58 (c = 0.39, $CHCl_3$).

[back to table of contents]

methyl 2-((2*R*)-1-(benzylsulfonyl)-5-phenylpyrrolidin-2-yl)acetate (8x)

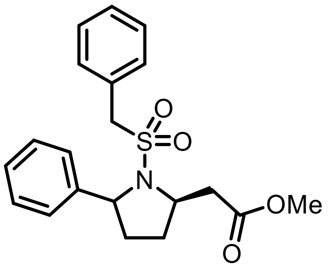

Pyrrolidine **8x** was prepared according to **General Procedure 6** from compound **7x** (37.3 mg, 99.9 µmol) and was purified by FCC (pentane/EtOAc 30%) to afford **8x** as a pale yellow oil (1:1 **A:B** mixture of inseparable diastereomers) (35.2 mg, 94.3 µmol, 94%, 87.5:12.5 ; 91.5:8.5 e.r.).

^{1}H NMR (600 MHz, $CDCl_3$) δ 7.41 – 7.28 (m, 18H, ArH, **A** *and* **B**), 7.22 – 7.18 (m, 2H, ArH, **B**), 4.82 (dd, *J* = 8.3, 1.6 Hz, 1H, CH, **B**), 4.75 (t, *J* = 6.9 Hz, 1H, CH, **A**), 4.38 (ddt, *J* = 9.4, 7.4, 4.7 Hz, 1H, CH, **A**), 4.25 (dddd, *J* = 10.5, 6.9, 3.7, 1.4 Hz, 1H, CH, **B**), 4.02 (d, *J* = 13.9 Hz, 1H, CH_2, **A**), 3.99 (d, *J* = 13.9 Hz, 1H, CH_2, **A**), 3.74 (d, *J* = 13.5 Hz, 1H, CH_2, **B**), 3.68 (s, 3H, CH_3, **A** *or* **B**), 3.68 (s, 3H, CH_3, **A** *or* **B**), 3.61 (d, *J* = 13.5 Hz, 1H, CH_2, **B**), 3.22 – 3.17 (m, 1H, CH_2, **B**), 2.79 (dd, *J* = 15.7, 4.6 Hz, 1H, CH_2, **A**), 2.50 – 2.36 (m, 4H, CH_2, **A** *and* **B**), 2.26 – 2.14 (m, 2H, CH_2, **A**), 2.01 – 1.92 (m, 2H, CH_2, **A** *and* **B**), 1.88 – 1.83 (m, 1H, CH_2, **B**), 1.78 – 1.71 (m, 1H, CH_2, **A**).

^{13}C NMR (151 MHz, $CDCl_3$) δ 171.8 (C=O, **B**), 171.6 (C=O, **A)**, 142.1 (ArC, **A** *or* **B**), 141.9 (ArC, **A** *or* **B**), 131.0 (ArC, **A** *or* **B**), 130.8 (ArC, **A** *or* **B**), 129.2 (ArC, **A** *or* **B**), 128.9 (ArC, **A** *or* **B**), 128.8 (ArC, **A** *or* **B**), 128.8 (ArC, **A** *or* **B**), 128.7 (ArC, **A** *or* **B**), 128.7 (ArC, **A** *or* **B**), 128.7 (ArC, **A** *or* **B**), 128.1 (ArC, **A** *or* **B**), 127.8 (ArC, **A** *or* **B**), 127.7 (ArC, **A** *or* **B**), 127.0 (ArC, **A** *or* **B**), 65.3 (CH, **A**), 63.5 (CH, **B**), 60.1 (CH_2, **B**), 58.2 (CH, **B**), 58.1 (CH_2, **A**), 58.0 (CH, **A**), 51.9 (CH_3, **A** *or* **B**), 51.8 (CH_3, **A** *or* **B**), 41.1 (CH_2, **A**), 39.5 (CH_2, **B**), 34.5 (CH_2, **A**), 32.2 (CH_2, **B**), 30.9 (CH_2, **A**), 30.6 (CH_2, **B**).

HRMS (ESI) calcd. for $[M+H]^+$ $(C_{20}H_{24}NO_4S)^+$ 374.1421, found 374.1420.

HPLC CHIRALPAK® IH-3, Hexane/IPA 95/5, 0.3 mL/min, λ = 220 nm, t_R (**B**, major) = 16.2 min, t_R (**A**, minor) = 20.0 min, t_R (**A**, major) = 27.0 min, t_R (**B**, minor) = 31.4 min.

FT-IR (thin film) ν_{max} (cm^{-1}) = 2980, 1734, 1455, 1341, 1151, 1075, 914, 743.

[back to table of contents]

methyl (E)-5-((5*R*)-1-(benzylsulfonyl)-5-(2-methoxy-2-oxoethyl)pyrrolidin-2-yl)pent-2-enoate (8y)

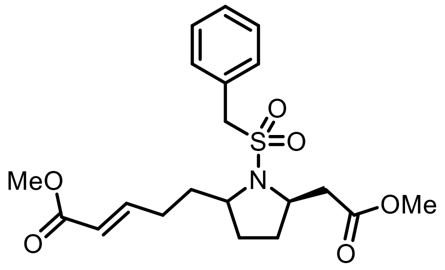

Pyrrolidine **8y** was prepared according to **General Procedure 6** from compound **7y** (40.8 mg, 99.6 μmol) and was purified by FCC (pentane/EtOAc 30%) to afford **8y** as a pale yellow oil (2:1 **A:B** mixture of inseparable diastereomers) (37.9 mg, 92.6 μmol, 93%, 91.5:8.5 ; 79:21 e.r.).

^{1}H NMR (600 MHz, $CDCl_3$) [integrals given normalised to minor diastereomer B] δ 7.46 – 7.34 (m, 15H, ArH, **A** *and* **B**), 6.89 (dt, J = 15.7, 6.8 Hz, 1H, C=CH, **B**), 6.84 (dt, J = 15.7, 6.7 Hz, 2H, C=CH, **A**), 5.80 (dt, J = 15.7, 1.6 Hz, 1H, C=CH, **B**), 5.77 (dt, J = 15.6, 1.6 Hz, 2H, C=CH, **A**), 4.32 (d, J = 13.9 Hz, 2H, CH_2, **A**), 4.25 (s, 2H, CH_2, **B**), 4.22 (d, J = 13.9 Hz, 2H, CH_2, **A**), 4.06 (tdd, J = 7.4, 5.6, 4.4 Hz, 1H, CH, **B**), 3.93 – 3.86 (m, 2H, CH, **A**), 3.74 (s, 6H, CH_3, **A**), 3.73 (s, 3H, CH_3, **B**), 3.68 (s, 3H, CH_3, **B**), 3.65 (s, 6H, CH_3, **A**), 3.65 – 3.60 (m, 2H, CH, **A**), 3.53 (ddt, J = 9.9, 7.4, 4.3 Hz, 1H, CH, **B**), 3.04 (ddd, J = 16.0, 3.9, 1.0 Hz, 2H, CH_2, **A**), 2.92 (dd, J = 16.0, 4.4 Hz, 1H, CH_2, **B**), 2.36 (dd, J = 16.0, 9.5 Hz, 1H, CH_2, **B**), 2.26 (dd, J = 15.9, 10.1 Hz, 2H, CH_2, **A**), 2.23 – 2.15 (m, 2H, CH_2, **B**), 2.13 – 2.07 (m, 1H, CH_2, **B**), 2.10 – 2.05 (m, 4H, CH_2,**A**), 2.04 – 1.96 (m, 2H, CH_2, **A**), 1.96 – 1.89 (m, 2H, CH_2, **A**), 1.91 – 1.84 (m, 1H, CH_2, **B**), 1.83 – 1.78 (m, 1H, CH_2, **B**), 1.76 – 1.68 (m, 1H, CH_2, **B**), 1.70 – 1.66 (m, 2H, CH_2, **A**), 1.66 – 1.61 (m, 2H, CH_2, **A**), 1.59 (dtd, J = 12.8, 6.5, 4.6 Hz, 1H, CH_2, **B**), 1.51 – 1.42 (m, 1H, CH_2, **B**), 1.21 (dddd, J = 12.9, 10.9, 9.1, 5.7 Hz, 2H, CH_2, **A**).

^{13}C NMR (151 MHz, $CDCl_3$) δ 171.7 (C=O, **A**), 171.6 (C=O, **B**), 167.1 (C=O, **B**), 167.0 (C=O, **A**), 148.2 (C=C, **B**), 147.7 (C=C, **A**), 130.9 (ArC, **B**), 130.9 (ArC, **A**), 129.4 (ArC, **A**), 129.0 (ArC, **B**), 129.0 (ArC, **A**), 128.9 (ArC, **A**), 128.9 (ArC, **B**), 128.8 (ArC, **B**), 121.6 (ArC, **A**), 121.5 (ArC, **B**), 61.6 (CH, **B**), 60.6 (CH_2, **A**), 60.3 (CH, **A**), 57.9 (CH, **B**), 56.8 (CH, **A**), 55.7 (CH_2, **B**), 51.8 (CH_3, **B**), 51.8 (CH_3, **A**), 51.6 (CH_3, **A**), 51.6 (CH_3, **B**), 41.8 (CH_2, **B**), 39.0 (CH_2, **A**), 35.2 (CH_2, **B**), 31.9 (CH_2, **A**), 30.9 (CH_2, **B**), 29.8 (CH_2, **B**), 29.4 (CH_2, **A**), 29.4 (CH_2, **A**), 29.0 (CH_2, **B**), 27.2 (CH_2, **A**).

HRMS (ESI) calcd. for $[M+Na]^+$ $(C_{20}H_{27}NO_6SNa)^+$ 432.1451, found 432.1438.

HPLC CHIRALPAK® AD, Hexane/IPA 90/10, 1 mL/min, λ = 220 nm, t_R (**A**, major) = 38.7 min, t_R (**B**, minor) = 41.9 min, t_R (**A**, minor) = 54.6 min, t_R (**B**, major) = 60.4 min.

FT-IR (thin film) ν_{max} (cm^{-1}) = 2980, 2888, 1724, 1655, 1339, 1151, 913, 743.

[back to table of contents]

methyl (*R*)-2-(1-(benzylsulfonyl)indolin-2-yl)acetate (8z)

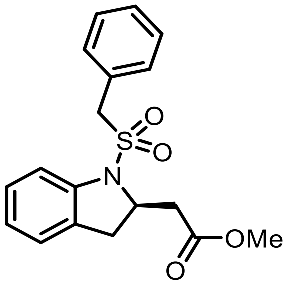

Indoline **8z** was prepared according to **General Procedure 6** from compound **7z** (37.1 mg, 104 µmol) and was purified by FCC (pentane/EtOAc 20%) to afford **8z** as a white solid (31.5 mg, 91.2 µmol, 85%, 92.5:7.5 e.r.).

^{1}H NMR (600 MHz, $CDCl_3$) δ 7.42 (d, *J* = 8.0 Hz, 1H), 7.36 – 7.33 (m, 1H), 7.31 – 7.27 (m, 2H), 7.23 – 7.19 (m, 1H), 7.16 – 7.10 (m, 3H), 7.05 (td, *J* = 7.4, 1.0 Hz, 1H), 4.36 (d, *J* = 14.1 Hz, 1H), 4.31 (d, *J* = 14.1 Hz, 1H), 4.12 (tdd, *J* = 9.8, 4.1, 3.1 Hz, 1H), 3.62 (s, 3H), 2.98 (dd, *J* = 16.5, 9.7 Hz, 1H), 2.85 (dd, *J* = 16.1, 4.1 Hz, 1H), 2.64 (dd, *J* = 16.5, 3.1 Hz, 1H), 2.53 (dd, *J* = 16.1, 10.0 Hz, 1H).

^{13}C NMR (151 MHz, $CDCl_3$) δ 171.1, 141.1, 130.8, 130.5, 129.2, 129.0, 128.2, 128.1, 125.7, 124.3, 114.6, 60.1, 56.2, 51.8, 41.8, 34.7.

HRMS (ESI) calcd. for $[M+H]^+$ $(C_{18}H_{20}NO_4S)^+$ 346.1108, found 346.1102.

HPLC CHIRALPAK® IA, Hexane/IPA 90/10, 1 mL/min, λ = 220 nm, t_R (minor) = 12.9 min, t_R (major) = 14.9 min.

FT-IR (thin film) ν_{max} (cm^{-1}) = 1734, 1479, 1457, 1351, 1155, 1104, 784, 698.

M.P. 108-110 °C.

$[\alpha]_D^{20}$ = -45.9 (c = 0.64, $CHCl_3$).

[back to table of contents]

methyl (*R*)-2-(5-bromo-1-(phenylsulfonyl)indolin-2-yl)acetate (8aa)

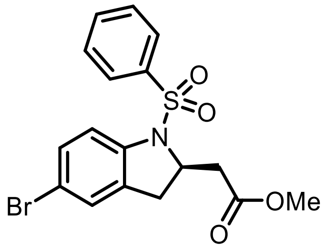

Indoline 8**aa** was synthesised according to a modified version of **General Procedure 6** from compound **7aa** (41.0 mg, 100 µmol) for 7 days and was purified by FCC (pentane/Et_2O 30%) to afford **8aa** as a colourless oil (32.3 mg, 78.8 µmol, 79%, 85:15 e.r.).

^{1}H NMR (400 MHz, $CDCl_3$) δ 7.70 – 7.65 (m, 2H), 7.56 (dd, *J* = 8.3, 5.5 Hz, 2H), 7.42 (t, *J* = 7.7 Hz, 2H), 7.34 (dd, *J* = 8.6, 2.0 Hz, 1H), 7.16 (d, *J* = 2.0 Hz, 1H), 4.57 (tt, *J* = 9.7, 3.6 Hz, 1H), 3.69 (s, 3H), 3.06 (dd, *J* = 16.3, 4.0 Hz, 1H), 2.93 (dd, *J* = 16.8, 9.4 Hz, 1H), 2.67 (dd, *J* = 16.5, 10.3 Hz, 1H), 2.60 (dd, *J* = 17.3, 3.5 Hz, 1H).

^{13}C NMR (101 MHz, $CDCl_3$) δ 171.2, 140.3, 137.4, 133.6, 133.6, 131.1, 129.4, 128.5, 127.1, 118.6, 117.9, 59.1, 52.0, 41.2, 34.6.

HRMS (ESI) calcd. for $[M+H]^+$ $(C_{17}H_{17}BrNO_4S)^+$ 410.0056, found 410.0047.

HPLC CHIRALPAK® OD, Hexane/IPA 95/5, 1 mL/min, λ = 220 nm, t_R (major) = 14.7 min, t_R (minor) = 18.4 min.

$[\alpha]_D^{20}$ = –46.2 (c = 1.61, $CHCl_3$).

methyl (*R*)-2-(5-methoxy-1-(phenylsulfonyl)indolin-2-yl)acetate (8ab)

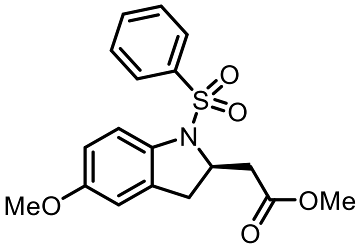

Indoline **8ab** was synthesised according to **General Procedure 6** from compound **7ab** (36.1 mg, 100 µmol) and was purified by FCC (pentane/Et_2O 75%) to afford **8ab** as a colourless oil (29.3 mg, 81.2 µmol, 81%, 83:17 e.r.).

^{1}H NMR (400 MHz, $CDCl_3$) δ 7.65 – 7.60 (m, 2H), 7.58 (d, *J* = 8.8 Hz, 1H), 7.52 (tt, *J* = 7.3, 1.7 Hz, 1H), 7.40 – 7.35 (m, 2H), 6.76 (dd, *J* = 8.9, 2.7 Hz, 1H), 6.59 – 6.54 (m, 1H), 4.56 (dddd, *J* = 10.0, 9.1, 4.3, 2.6 Hz, 1H), 3.75 (s, 3H), 3.69 (s, 3H), 3.00 (dd, *J* = 16.2, 4.3 Hz, 1H), 2.78 (dd, *J* = 16.6, 9.2 Hz, 1H), 2.63 (dd, *J* = 16.2, 10.0 Hz, 1H), 2.50 (dd, *J* = 16.7, 2.6 Hz, 1H).

^{13}C NMR (101 MHz, $CDCl_3$) δ 171.4, 157.8, 137.4, 134.3, 133.3, 133.2, 129.1, 127.3, 118.7, 113.3, 111.0, 59.2, 55.7, 51.9, 41.1, 34.9.

HRMS (ESI) calcd. for $[M+H]^+$ $(C_{18}H_{20}NO_5S)^+$ 362.1057, found 362.1058.

HPLC CHIRALPAK® OD, Hexane/IPA 95/5, 1 mL/min, λ = 220 nm, t_R (major) = 19.1 min, t_R (minor) = 23.7 min.

$[\alpha]_D^{20}$ = –43.1 (c = 1.46, $CHCl_3$).

[back to table of contents]

methyl (*R*)-2-(5-methyl-1-(phenylsulfonyl)indolin-2-yl)acetate (8ac)

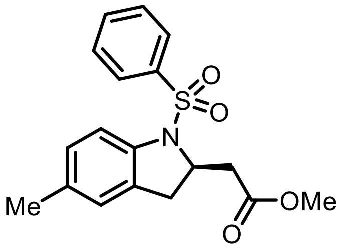

Indoline **8ac** was synthesised according to **General Procedure 6** from compound **7ac** (34.5 mg, 100 µmol) and was purified by FCC (pentane/Et_2O 75%) to afford **8ac** as a colourless oil (32.9 mg, 95.4 µmol, 95%, 86:14 e.r.).

^{1}H NMR (400 MHz, $CDCl_3$) δ 7.69 – 7.64 (m, 2H), 7.58 – 7.48 (m, 2H), 7.38 (t, *J* = 7.8 Hz, 2H), 7.05 – 7.00 (m, 1H), 6.84 (s, 1H), 4.56 (dddd, *J* = 10.0, 9.3, 4.2, 2.9 Hz, 1H), 3.69 (s, 3H), 3.04 (dd, *J* = 16.2, 4.2 Hz, 1H), 2.86 (dd, *J* = 16.5, 9.3 Hz, 1H), 2.64 (dd, *J* = 16.2, 10.1 Hz, 1H), 2.53 (dd, *J* = 16.5, 2.9 Hz, 1H), 2.27 (s, 3H).

^{13}C NMR (101 MHz, $CDCl_3$) δ 171.4, 138.6, 137.7, 134.9, 133.2, 131.4, 129.1, 128.7, 127.2, 126.0, 117.2, 59.0, 51.9, 41.3, 34.7, 21.1.

HRMS (ESI) calcd. for $[M+H]^+$ $(C_{18}H_{20}NO_4S)^+$ 346.1108, found 346.1104.

HPLC CHIRALPAK® OD, Hexane/IPA 95/5, 1 mL/min, λ = 220 nm, t_R (major) = 12.2 min, t_R (minor) = 14.4 min.

$[\alpha]_D^{20}$ = –37.6 (c = 1.47, $CHCl_3$).

[back to table of contents]

methyl (*S*)-2-(2-(benzylsulfonyl)isoindolin-1-yl)acetate (8ad)

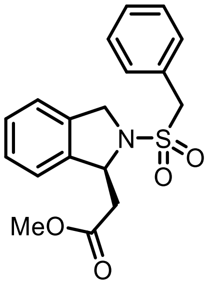

Isoindoline **8ad** was prepared according to **General Procedure 6** from compound **7ad** (36.7 mg, 106 μmol) and was purified by FCC (pentane/EtOAc 20%) to afford **8ad** as a white solid (32.1 mg, 92.9 μmol, 87%, 91.5:8.5 e.r.).

^{1}H NMR (600 MHz, $CDCl_3$) δ 7.32 – 7.24 (m, 3H), 7.23 – 7.18 (m, 4H), 7.18 – 7.15 (m, 1H), 7.12 – 7.08 (m, 1H), 5.22 (ddd, *J* = 7.4, 4.4, 2.4 Hz, 1H), 4.50 (ddd, *J* = 14.2, 2.8, 1.0 Hz, 1H), 4.37 (d, *J* = 14.0 Hz, 1H), 4.32 (d, *J* = 14.2 Hz, 1H), 4.29 (d, *J* = 14.2 Hz, 1H), 3.66 (s, 3H), 2.96 (dd, *J* = 16.1, 4.5 Hz, 1H), 2.70 (dd, *J* = 16.1, 7.6 Hz, 1H).

^{13}C NMR (151 MHz, $CDCl_3$) δ 171.1, 139.5, 136.3, 130.8, 128.8, 128.8, 128.8, 128.4, 128.1, 122.8, 122.5, 62.6, 56.6, 54.2, 51.8, 42.3.

HRMS (ESI) calcd. for $[M+Na]^+$ $(C_{18}H_{19}NO_4SNa)^+$ 368.0927, found 368.0914.

HPLC CHIRALPAK® IA, Hexane/IPA 90/10, 1 mL/min, λ = 220 nm, t_R (minor) = 18.4 min, t_R (major) = 23.2 min.

FT-IR (thin film) ν_{max} (cm^{-1}) = 2341, 1733, 1338, 1152, 1130, 698.

M.P. 64- 66 °C.

$[\alpha]_D^{20}$ = +90.1 (c = 0.73, $CHCl_3$).

[back to table of contents]

methyl (*R*)-2-(1-(phenylsulfonyl)-1,2,3,4-tetrahydroquinolin-2-yl)acetate (8ae)

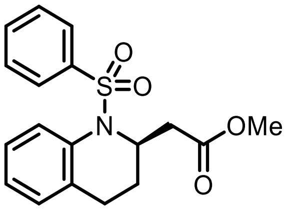

Tetrahydroquinoline **8ae** was synthesised according to **General Procedure 6** from compound **7ae** (34.8 mg, 100 μmol) and was purified by FCC (pentane/Et_2O 33%) to afford **8ae** as a yellow oil (17.1 mg, 50 μmol, 50%, 90:10 e.r.).

^{1}H NMR (400 MHz, $CDCl_3$) δ 7.71 (dd, *J* = 8.1, 1.3 Hz, 1H), 7.55 – 7.45 (m, 3H), 7.40 – 7.32 (m, 2H), 7.28 – 7.21 (m, 1H), 7.13 (td, *J* = 7.5, 1.3 Hz, 1H), 6.96 (dd, *J* = 7.4, 1.5 Hz, 1H), 4.67 – 4.57 (m, 1H), 3.67 (s, 3H), 2.86 (dd, *J* = 15.4, 5.3 Hz, 1H), 2.49 (dd, *J* = 15.4, 8.7 Hz, 1H), 2.33 (ddd, *J* = 15.8, 6.7, 5.1 Hz, 1H), 1.97 (dtd, *J* = 13.3, 6.6, 5.0 Hz, 1H), 1.65 (ddd, *J* = 15.1, 9.0, 5.0 Hz, 1H), 1.42 (dddd, *J* = 13.5, 9.0, 7.3, 5.1 Hz, 1H).

^{13}C NMR (101 MHz, $CDCl_3$) δ 171.3, 138.8, 135.0, 133.9, 132.9, 129.0, 128.0, 127.9, 127.2, 127.1, 126.3, 53.3, 51.9, 40.8, 28.5, 24.6.

HRMS (ESI) calcd. for $[M+Na]^+$ $(C_{12}H_{18}N_2O_5SNa)^+$ 325.0829, found 325.0824.

HPLC CHIRALPAK® IA, Hexane/IPA 98/2, 1 mL/min, λ = 220 nm, t_R (major) = 25.7 min, t_R (minor) = 28.5 min.

$[\alpha]_D^{20}$ = +23.5 (c = 0.886, $CHCl_3$).

150 mg Scale Synthesis of Compound **8a**

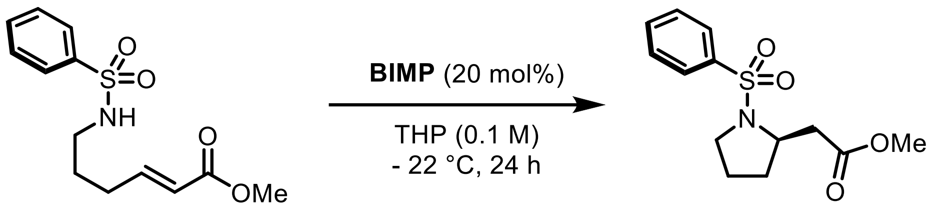

Azide **A17** (39 mg, 106 μmol) and tricyclohexylphosphine (30 mg, 106 μmol) were dissolved in anhydrous THF (2.5 mL) in a sealed 10 mL vial. The reaction mixture was stirred at 40 °C for 18 hours, following which the solvent was removed *in vacuo* and subsequently dried under reduced pressure for 20 mins. To this vial was added a solution of sulfonamide 7a (150 mg, 529 μmol) in technical grade THP (5.3 mL). The reaction mixture was sealed under air and stirred at -22 °C for 24 hours. The reaction was quenched by passing down a short silica plug and eluting with EtOAc. The crude reside was purified by FCC (pentane/EtOAc 10-20%) to afford pyrrolidine **8a** as a colourless oil (147 mg, 519 μmol, 98%, 94.5:5.5 er). Spectroscopic data matches that of **8a**.

[back to table of contents]

Preparative Scale Synthesis of Compound **8n**

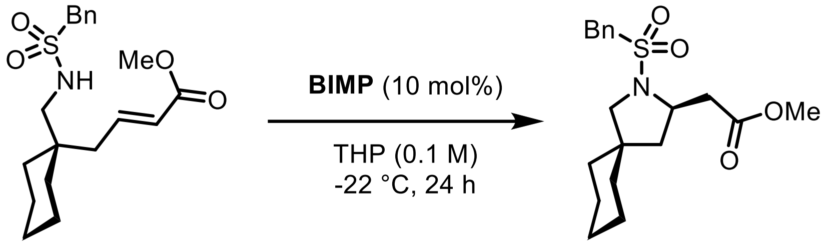

Azide **A17** (154 mg, 0.41 mmol) and tricyclohexylphosphine (119 mg, 0.41 mmol) were dissolved in anhydrous THF (16 mL) under a nitrogen atmosphere. The reaction mixture was stirred at 40 °C for 19 hours, following which the solvent was removed *in vacuo* and subsequently dried under reduced pressure for 20 mins. The crude residue was taken up in technical grade THP (41 mL) and cooled to -22 °C. To this solution was added compound **7n** (1.50 g, 4.10 mmol). The reaction mixture was sealed under air and stirred at -22 °C for 24 hours. The reaction was quenched by passing down a short silica plug and eluting with EtOAc. The crude reside was purified by FCC (pentane/EtOAc 30%) to afford pyrrolidine **8n** as a white powder (1.47 g, 4.02 mmol, 98%, 96:4 er). The white solid was then refluxed in hexane (20 mL) until complete dissolution, and was then cooled to 0 °C to afford pyrrolidine **8n** as a white crystalline solid (1.18 g, 3.22 mmol, 79%, 97:3 e.r.). Spectroscopic data matches that of **8n**.

[back to table of contents]

Synthesis of Product Derivatives

tert-butyl (*R*)-2-(2-methoxy-2-oxoethyl)pyrrolidine-1-carboxylate (9)

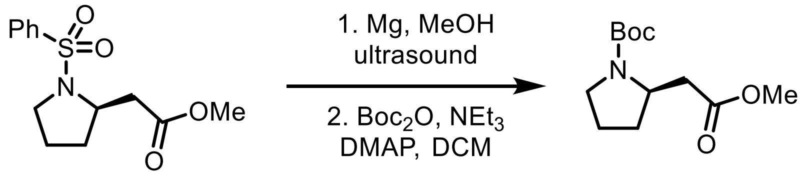

To a vial under argon containing magnesium turnings (108 mg, 4.40 mmol) was added a solution of sulfonamide **8a** (51.1 mg, 180 µmol) in MeOH (3.5 mL). The reaction mixture was sonicated at room temperature for 1 hour, following which it was quenched with aqueous 1 M HCl solution (5 mL), basified to pH 9 with saturated aqueous $NaHCO_3$, and extracted with $CHCl_3$/IPA 3/1 (5 x 50 mL). The combined organic layers were dried over anhydrous Na_2SO_4, filtered, and concentrated *in vacuo*. The crude oil was taken up in DCM (5 mL), and to this solution was added NEt_3 (74 µL, 0.5 mmol), DMAP (5 mg, 0.04 mmol), and a solution of Boc_2O (58 mg, 0.26 mmol) in DCM (1 mL). The reaction mixture was stirred at room temperature for 16 hours, following which aqueous saturated $NaHCO_3$ (25 mL) was added. The aqueous phase was extracted with DCM (3 x 25 mL), the combined organic layers were washed with brine (25 mL), dried over Na_2SO_4, filtered and concentrated *in vacuo*. The crude residue was purified by fcc (pentane/EtOAc 5%-20%) to afford *N*-Boc protected pyrrolidine **9** as a colourless oil (16.8 mg, 69.0 µmol, 38%).

^{1}H NMR (600 MHz, DMSO, 373 K) δ 4.05 – 3.98 (m, 1H), 3.61 (s, 3H), 3.32 – 3.27 (m, 1H), 3.26 – 3.20 (m, 1H), 2.71 (dd, *J* = 14.8, 4.0 Hz, 1H), 2.38 (dd, *J* = 14.8, 8.8 Hz, 1H), 2.05 – 1.97 (m, 1H), 1.88 – 1.66 (m, 3H), 1.42 (s, 9H).

^{13}C NMR (151 MHz, DMSO, 373 K) δ 170.6, 152.9, 78.0, 53.3, 50.5, 45.5, 38.0, 30.0, 27.7, 22.1.

HRMS (ESI) calcd. for $[M+Na]^+$ $(C_{12}H_{21}NO_4Na)^+$ 266.1363, found 266.1357.

HPLC CHIRALPAK® AD-H, Hexane/IPA 98/2, 1 mL/min, λ = 220 nm, t_R (minor) = 9.3 min, t_R (major) = 11.8 min.

$[\alpha]_D^{20}$ = +16.5 (c=0.4, $CHCl_3$).

The data is consistent with the literature.[22]

[back to table of contents]

ethyl 2-(1-(benzylsulfonyl)-5-oxopyrrolidin-2-yl)acetate (10)

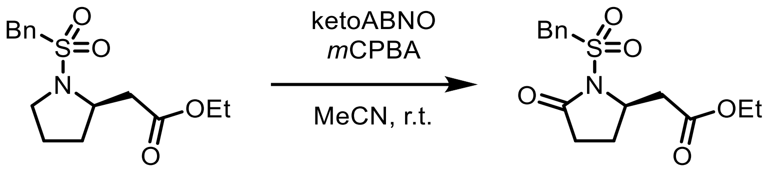

Compound **10** was prepared according to the modified literature procedure.[23] In a 1 mL vial, pyrrolidine **8b** (15.3 mg, 49.1 μmol) and ketoABNO (1.8 mg, 11.7 μmol) were dissolved in MeCN (0.5 mL). To this solution was added *m*CPBA (36.5 mg, 211 μmol) in one portion. The reaction mixture was sealed under air and stirred for 20 hours, after which it was quenched by addition of saturated aqueous $Na_2S_2O_4$ (0.5 mL). The mixture was stirred for 20 mins, then diluted with sat $Na_2S_2O_4$ (10 mL), extracted with EtOAc (3 × 10 mL) then the combined organics were washed with $NaHCO_3$ (10 mL), brine (10 mL), dried over Na_2SO_4 and concentrated *in vacuo*. The crude residue was purified by preparative TLC (hexane/EtOAc 40% + NEt_3 1% v/v) to afford compound **10** as a white solid (5.2 mg, 16.0 μmol, 33%, 95.5:4.5 e.r.).

^{1}H NMR (600 MHz, $CDCl_3$) δ 7.45 – 7.35 (m, 5H), 4.74 – 4.67 (m, 2H), 4.14 – 4.01 (m, 3H), 2.60 (ddd, *J* = 17.8, 11.1, 9.3 Hz, 1H), 2.45 – 2.43 (m, 1H), 2.42 – 2.40 (m, 1H), 2.09 (dd, *J* = 16.2, 10.1 Hz, 1H), 2.07 – 2.01 (m, 1H), 1.78 (ddt, *J* = 13.4, 9.2, 2.2 Hz, 1H), 1.21 (t, *J* = 7.1 Hz, 3H).

^{13}C NMR (151 MHz, $CDCl_3$) δ 175.0, 170.0, 130.9, 129.7, 129.2, 127.9, 61.0, 58.8, 56.4, 38.6, 30.3, 24.4, 14.2.

HRMS (ESI) calcd. for $[M+H]^+$ $(C_{15}H_{20}NO_5S)^+$ 326.1057, found 326.1049.

HPLC CHIRALPAK® IA, Hexane/IPA 80/20, 1 mL/min, λ = 220 nm, t_R (minor) = 14.5 min, t_R (major) = 17.2 min.

FT-IR (thin film) ν_{max} (cm^{-1}) = 2923, 1730, 1354, 1182, 1165, 1109, 957, 700.

M.P. 70-72 °C.

$[\alpha]_D^{20}$ = +72.3 (c=0.16, $CHCl_3$).

[back to table of contents]

(*R*)-2-(2-(benzylsulfonyl)-2-azaspiro[4.5]decan-3-yl)ethan-1-ol (11)

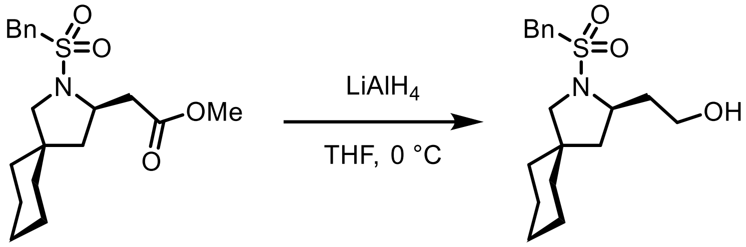

To a solution of pyrrolidine **8n** (154 mg, 0.42 mmol) in THF (4 mL) at 0 °C was added $LiAlH_4$ (2.4 M in THF, 340 μL) dropwise over 5 mins. The solution was stirred at 0 °C for 15 minutes, before being diluted with Et_2O (5 mL) and quenched with H_2O (50 μL), then 15% aqueous NaOH (50 μL), then H_2O (150 μL). The mixture was dried over Na_2SO_4, filtered and concentrated *in vacuo* to afford crude alcohol **11** as a colourless oil which was used without further purification (132 mg, 0.39 mmol, 93%, 97.5:2.5 e.r.).

^{1}H NMR (600 MHz, $CDCl_3$) δ 7.42 – 7.34 (m, 5H), 4.25 (d, *J* = 13.7 Hz, 1H), 4.20 (d, *J* = 13.7 Hz, 1H), 4.03 (qd, *J* = 7.7, 5.6 Hz, 1H), 3.56 – 3.47 (m, 2H), 3.30 (dd, *J* = 10.5, 1.3 Hz, 1H), 2.68 (d, *J* = 10.6 Hz, 1H), 1.99 (ddd, *J* = 12.7, 7.6, 1.3 Hz, 1H), 1.91 – 1.83 (m, 1H), 1.57 – 1.50 (m, 1H), 1.49 – 1.23 (m, 11H).

^{13}C NMR (151 MHz, $CDCl_3$) δ 131.0, 129.5, 128.8, 128.7, 59.9, 59.2, 58.5, 56.8, 44.6, 42.7, 39.7, 36.5, 34.5, 26.0, 23.9, 23.0.

HRMS (ESI) calcd. for $[M+Na]^+$ $(C_{18}H_{27}NO_3SNa)^+$ 360.1604, found 360.1596.

SFC CHIRALPAK® IC, 1500 psi, 30 °C, CO_2/MeOH 99/1 to 70/30 over 5 mins, then 70/30 to 50/50 over 0.5 min, then hold 50/50 for 2.5 min, then 50/50 to 100/0 over 0.1 min, then hold 100/0 for 0.9 min, 1.5 mL/min, λ = 220 nm, t_R (minor) = 6.1 min, t_R (major) = 6.2 min.

FT-IR (thin film) ν_{max} (cm^{-1}) = 3502, 2925, 2854, 1453, 1328, 1147, 1125, 1057, 1030, 697.

$[\alpha]_D^{20}$ = +15.8 (c = 1.13, $CHCl_3$).

[back to table of contents]

(*R*)-3-(2-azidoethyl)-2-(benzylsulfonyl)-2-azaspiro[4.5]decane (12)

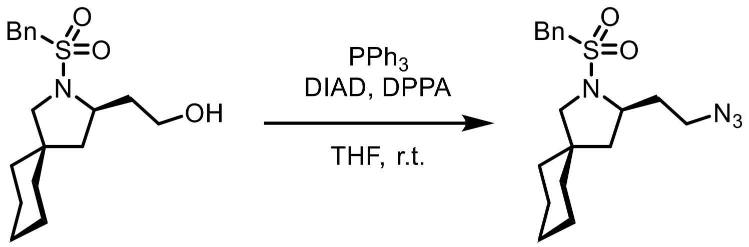

To a solution of alcohol **11** (99.2 mg, 0.29 mmol) and triphenylphosphine (121 mg, 0. 44 mmol) in THF (3 mL) under nitrogen at 0 °C was added diisopropyl azodicarboxylate (90 μL, 0.44 mmol) and diphenylphosphoryl azide (100 μL, 0.44 mmol). The reaction mixture was stirred at 0 °C for 10 mins, before being warmed to room temperature and stirred for 17 hours. The solvent was removed *in vacuo* and the crude residue was purified by FCC (pentane/EtOAc 0% to 10%) to afford azide **12** as a colourless oil (74.2 mg, 0.20 mmol, 70%, 97.5:2.5 e.r.).

^{1}H NMR (600 MHz, $CDCl_3$) δ 7.38 (s, 5H), 4.22 (d, *J* = 13.9 Hz, 1H), 4.19 (d, *J* = 13.8 Hz, 1H), 3.91 – 3.85 (m, 1H), 3.36 (dd, *J* = 10.4, 1.4 Hz, 1H), 3.14 – 3.05 (m, 2H), 2.70 (d, *J* = 10.3 Hz, 1H), 1.99 – 1.93 (m, 1H), 1.91 – 1.84 (m, 1H), 1.51 – 1.24 (m, 12H).

^{13}C NMR (151 MHz, $CDCl_3$) δ 131.0, 129.5, 128.8, 128.8, 59.3, 58.3, 57.3, 48.4, 44.1, 42.6, 36.4, 35.2, 34.1, 26.1, 23.9, 22.9.

HRMS (ESI) calcd. for $[M+H]^+$ $(C_{18}H_{27}N_4O_2S)^+$ 363.1849, found 363.1846.

HPLC CHIRALPAK® AS-H, Hexane/IPA 90/10, 1 mL/min, λ = 220 nm, t_R (major) = 17.9 min, t_R (minor) = 21.9 min.

FT-IR (thin film) ν_{max} (cm^{-1}) = 2927, 2855, 2095, 1454, 1330, 1149, 1126, 697.

$[\alpha]_D^{20}$ = +23.8 (c = 0.69, $CHCl_3$).

[back to table of contents]

N-((1-(2-((R)-2-(benzylsulfonyl)-2-azaspiro[4.5]decan-3-yl)ethyl)-1H-1,2,3-triazol-4-yl)methyl)-5-((3aS,4S,6aR)-2-oxohexahydro-1H-thieno[3,4-d]imidazol-4- yl)pentanamide (13)

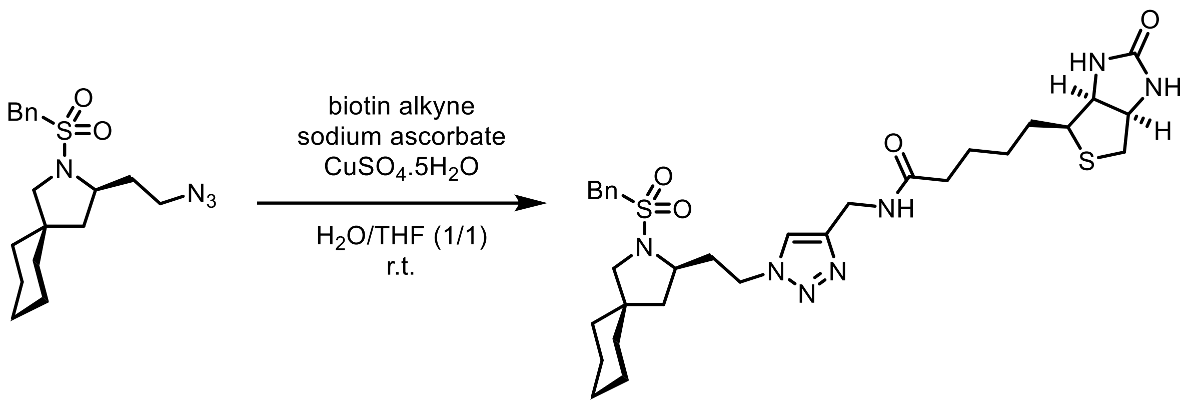

In a 3 mL vial, $CuSO_4.5H_2O$ (3.6 mg, 13 µmol), sodium ascorbate (6.5 mg, 33 µmol) and biotin alkyne (5-((3aS,4S,6aR)-2-oxohexahydro-1H-thieno[3,4-d]imidazol-4-yl)-N-(prop-2-yn-1- yl)pentanamide (39.5 mg, 140 µmol) were dissolved in water (0.4 mL). To this solution was added azide **12** (49.5 mg, 0.14 mmol) in THF (0.4 mL), and the vial was sealed under argon. The mixture was stirred at room temperature for 20 hours, before being diluted with water (25 mL) and extracted with EtOAc (3 × 25 mL). The combined organic layers were dried over Na_2SO_4, filtered and concentrated *in vacuo*. The crude residue was purified by FCC (DCM/MeOH 0% to 10%) to afford compound **13** as a white solid (61.6 mg, 95 µmol, 70%, >20:1 d.r.).

^{1}H NMR (600 MHz, $CDCl_3$) δ 7.73 (t, *J* = 5.9 Hz, 1H), 7.48 (s, 1H), 7.40 – 7.30 (m, 5H), 7.09 (s, 1H), 6.39 (s, 1H), 4.56 – 4.48 (m, 2H), 4.39 (dd, *J* = 15.0, 5.6 Hz, 1H), 4.32 (ddd, *J* = 7.9, 4.5, 1.3 Hz, 1H), 4.20 (s, 2H), 4.11 (t, *J* = 7.5 Hz, 2H), 3.85 – 3.77 (m, 1H), 3.36 (dd, *J* = 10.5, 1.2 Hz, 1H), 3.14 – 3.10 (m, 1H), 2.90 (dd, *J* = 12.8, 4.9 Hz, 1H), 2.76 (dd, *J* = 11.6, 3.6 Hz, 2H), 2.24 – 2.17 (m, 2H), 2.10 – 2.02 (m, 1H), 1.96 – 1.89 (m, 1H), 1.80 – 1.71 (m, 2H), 1.70 – 1.61 (m, 3H), 1.50 – 1.22 (m, 12H).

^{13}C NMR (151 MHz, $CDCl_3$) δ 173.4, 164.7, 145.4, 131.0, 129.3, 128.9, 128.9, 122.6, 61.8, 60.4, 58.8, 58.4, 56.8, 55.9, 47.3, 44.0, 42.7, 40.8, 36.6, 36.4, 35.9, 34.5, 34.2, 28.3, 28.1, 26.0, 25.5, 23.9, 22.9.

HRMS (ESI) calcd. for $[M+K]^+$ $(C_{31}H_{45}N_7O_4S_2K)^+$ 682.2606, found 682.2607.

FT-IR (thin film) ν_{max} (cm^{-1}) = 3265, 2927, 1697, 1454, 1329, 1146, 1125., 912, 730.

M.P. 94-96 °C.

$[\alpha]_D^{20}$ = +31.7 (c = 1.04, $CHCl_3$).

[back to table of contents]

Unsuccessful Substrates

In this section examples are shown that did not undergo the desired aza-Michael addition, displaying the limitations of our methodology. All examples below showed 0% conversion to the desired N-heterocyclic product.

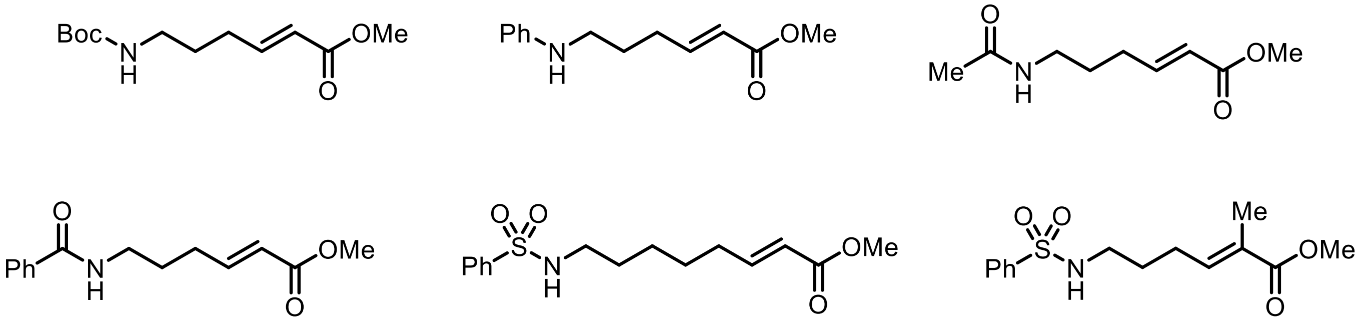

[back to table of contents]

Single Crystal X-Ray Diffraction Data

Single Crystal X-Ray Diffraction Data and Structure Refinement of Compound **8o**

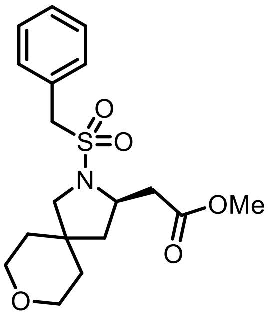

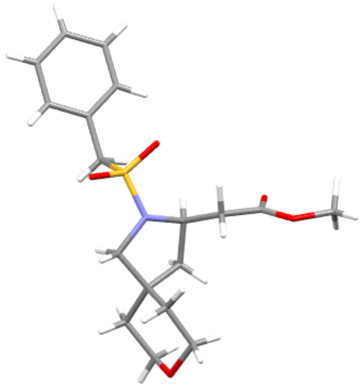

CCDC	2541404	
Empirical formula	$C_{16}H_{23}N_{1}O_{4}S$	
Formula Weight	325.43	
Temperature	150 K	
Wavelength	1.54180 Å	
Crystal System	Orthorhombic	
Space Group	P 21 21 21	
Unit cell dimensions	a = 7.79240(10) Å	α = 90 °
	b = 11.3204(2) Å	β = 90 °
	c = 18.9645(3) Å	γ = 90 °
Volume	1819.53(3) $Å^3$	
Z	4	
Density (calculated)	1.341 mg/m^3	
Absorption coefficient	1.824 mm^{-1}	
F(000)	784	
Crystal size	0.1 × 0.15 × 0.1 mm^3	
Theta range for data collection	4.549 to 76.609 °	
Index ranges	-9≤h≤9, -14≤k≤14, -23≤l≤23	
Reflections collected	52242	
Independent reflections	3483	
Completeness to theta = 75.076 °	99.9%	
Absorption correction	Multi-scan	
Refinement method	Full-matrix least-squares on F_2	
Data / restraints / parameters	3483 / 0 / 200	
Goodness-of-fit on F_2	1.0264	
Final R indices [I>2sigma(I)]	R1 = 0.0362, wR2 = 0.1027	
R indices (all data)	R1 = 0.0388, wR2 = 0.1053	
Absolute structure parameter	-0.005(6)	

[back to table of contents]

Single Crystal X-Ray Diffraction Data and Structure Refinement of Compound **8u**

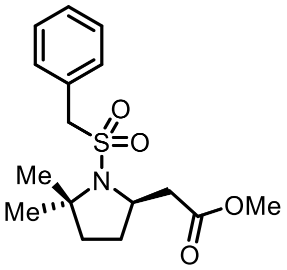

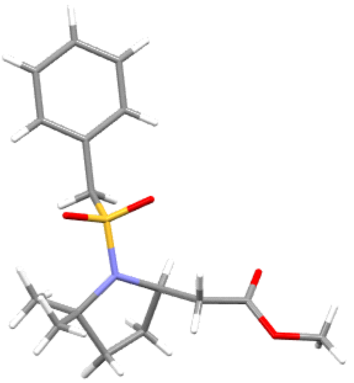

CCDC	2541402	
Empirical formula	$C_{18}H_{25}N_1O_5S$	
Formula Weight	367.47	
Temperature	150 K	
Wavelength	1.54184 Å	
Crystal System	Orthorhombic	
Space Group	P 21 21 21	
Unit cell dimensions	a = 7.90600(10) Å	α = 90 °
	b = 12.50980(10) Å	β = 90 °
	c = 18.39720(10) Å	γ = 90 °
Volume	1819.53(3) $Å^3$	
Z	4	
Density (calculated)	1.341 mg/m^3	
Absorption coefficient	1.824 mm^{-1}	
F(000)	784	
Crystal size	0.3 × 0.15 × 0.2 mm^3	
Theta range for data collection	4.274 to 76.041 °	
Index ranges	-9≤h≤9, -15≤k≤15, -22≤l≤23	
Reflections collected	68695	
Independent reflections	3757	
Completeness to theta = 74.520 °	99.9%	
Absorption correction	Multi-scan	
Refinement method	Full-matrix least-squares on F_2	
Data / restraints / parameters	3757 / 0 / 227	
Goodness-of-fit on F_2	0.8161	
Final R indices [I>2sigma(I)]	R1 = 0.0228, wR2 = 0.0647	
R indices (all data)	R1 = 0.0230, wR2 = 0.0651	
Absolute structure parameter	-0.002(2)	

[back to table of contents]

Single Crystal X-Ray Diffraction Data and Structure Refinement of Compound **8w**

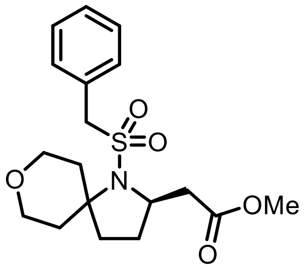

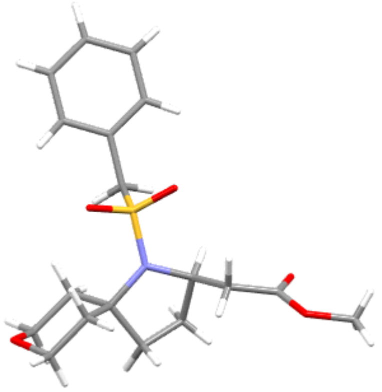

CCDC	2541403	
Empirical formula	$C_{18}H_{25}N_1O_5S$	
Formula Weight	367.47	
Temperature	150 K	
Wavelength	1.54184 Å	
Crystal System	Orthorhombic	
Space Group	P 21 21 21	
Unit cell dimensions	a = 7.90600(10) Å	α = 90 °
	b = 12.50980(10) Å	β = 90 °
	c = 18.39720(10) Å	γ = 90 °
Volume	1819.53(3) $Å^3$	
Z	4	
Density (calculated)	1.341 mg/m^3	
Absorption coefficient	1.824 mm^{-1}	
F(000)	784	
Crystal size	0.3 × 0.15 × 0.2 mm^3	
Theta range for data collection	4.274 to 76.041 °	
Index ranges	-9≤h≤9, -15≤k≤15, -22≤l≤23	
Reflections collected	68695	
Independent reflections	3757	
Completeness to theta = 74.520 °	99.9%	
Absorption correction	Multi-scan	
Refinement method	Full-matrix least-squares on F_2	
Data / restraints / parameters	3757 / 0 / 227	
Goodness-of-fit on F_2	0.8161	
Final R indices [I>2sigma(I)]	R1 = 0.0228, wR2 = 0.0647	
R indices (all data)	R1 = 0.0230, wR2 = 0.0651	
Absolute structure parameter	-0.002(2)	

[back to table of contents]

^{1}H, ^{13}C and ^{19}F NMR Spectra of Novel Compounds

[back to table of contents]

^{1}H NMR (600 MHz, $CDCl_3$) of compound **A17**

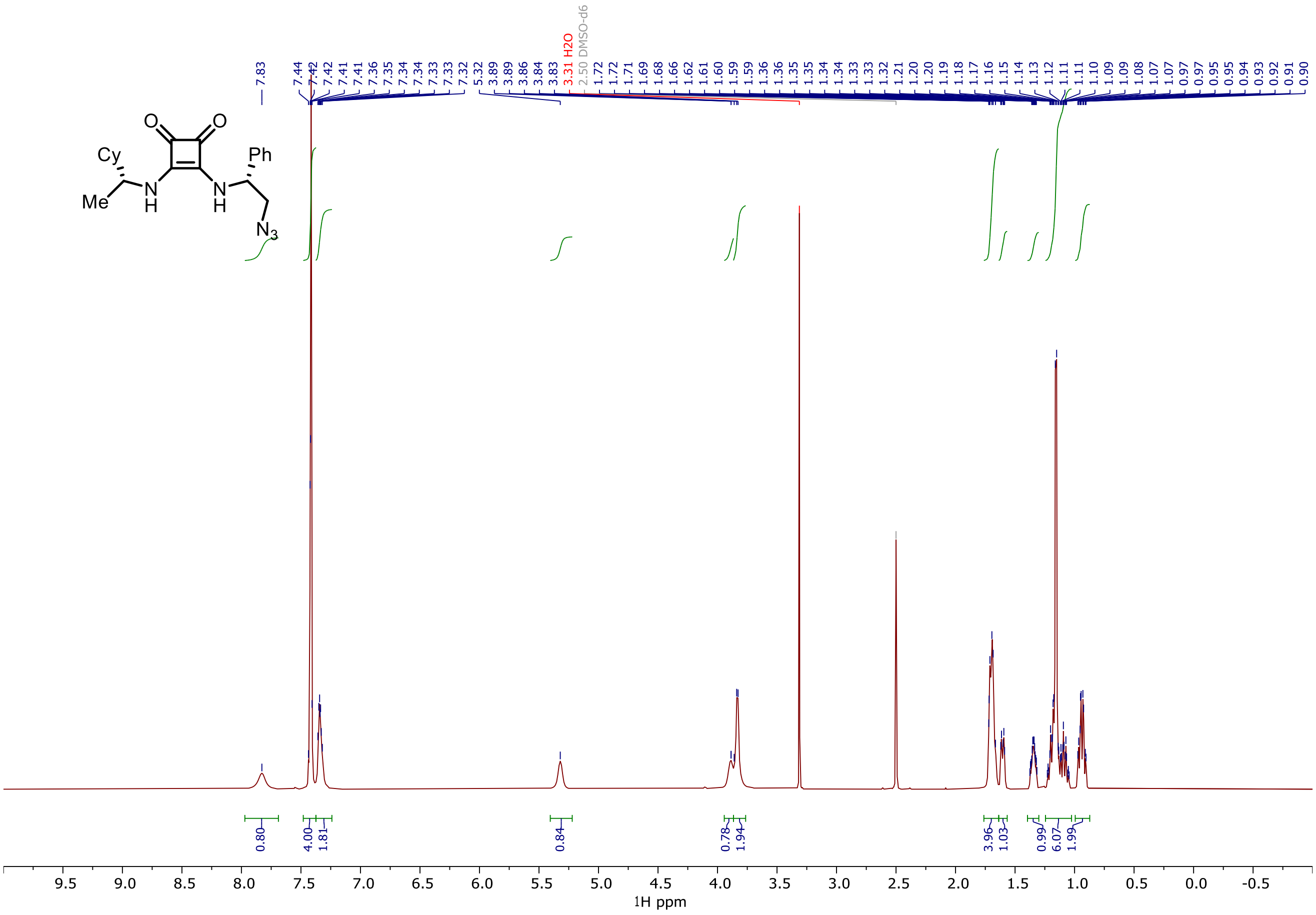

^{13}C NMR (151 MHz, $CDCl_3$) of compound **A17**

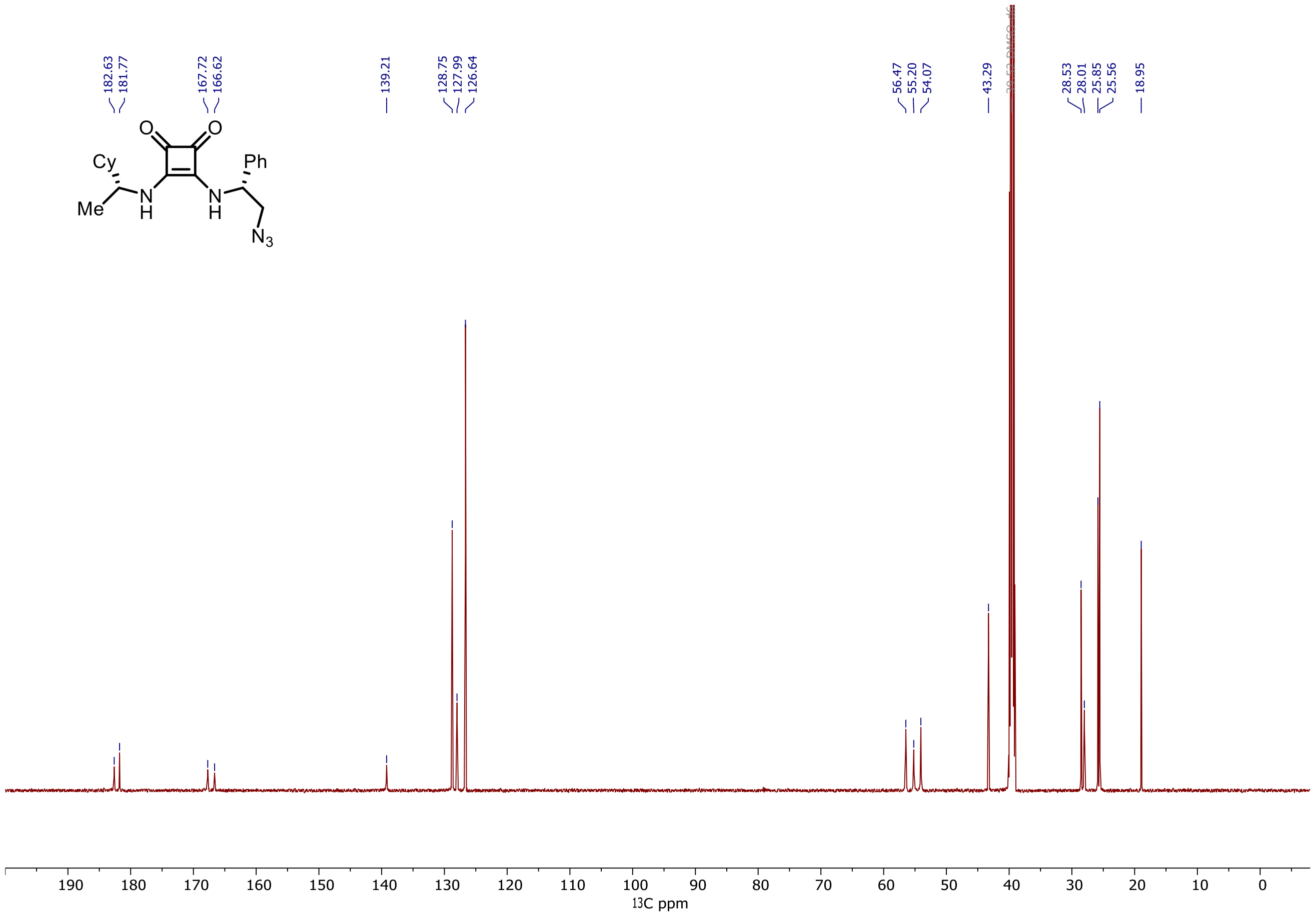

[back to table of contents]

^{1}H NMR (400 MHz, $CDCl_3$) of compound **7a**

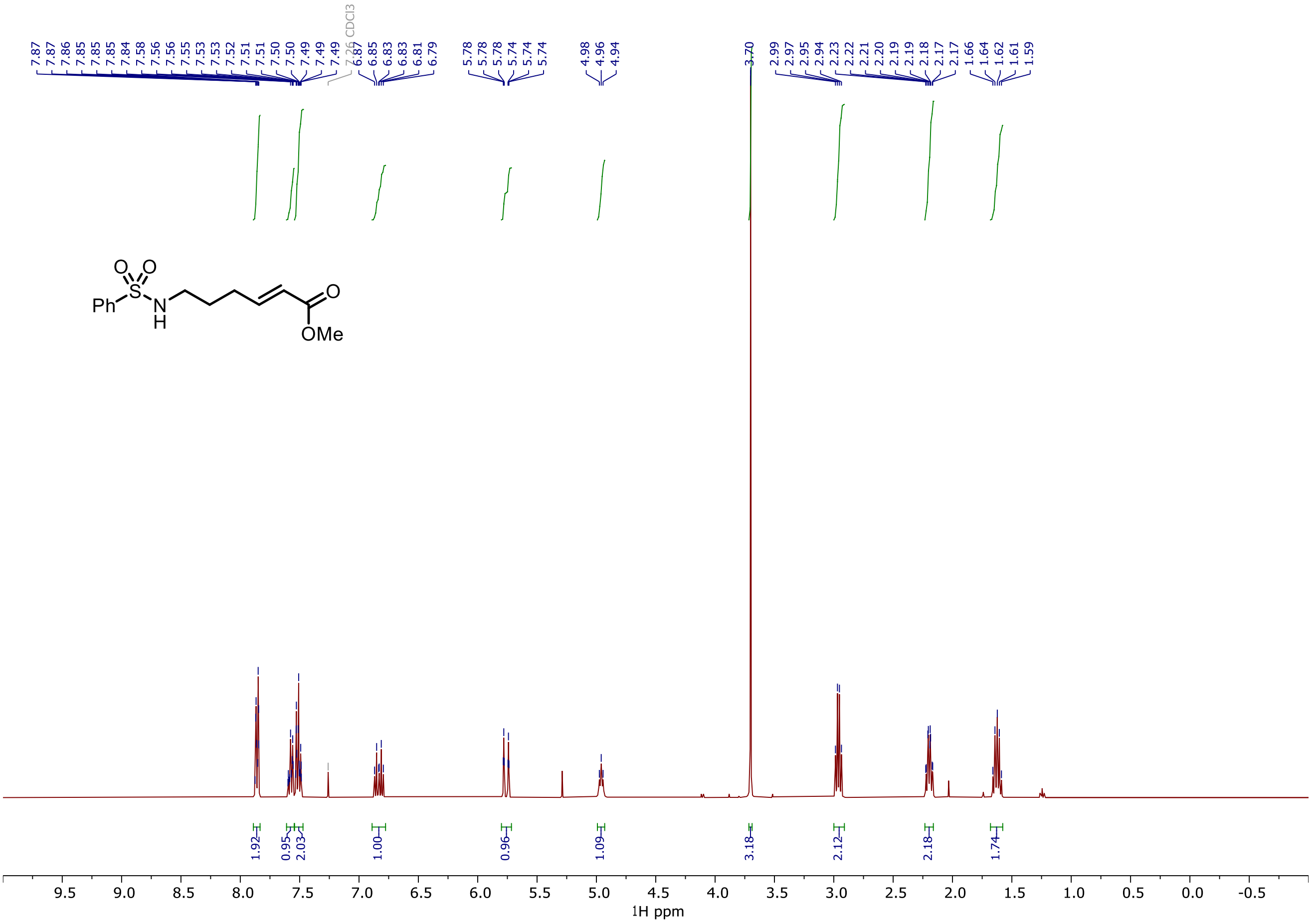

^{13}C NMR (101 MHz, $CDCl_3$) of compound **7a**

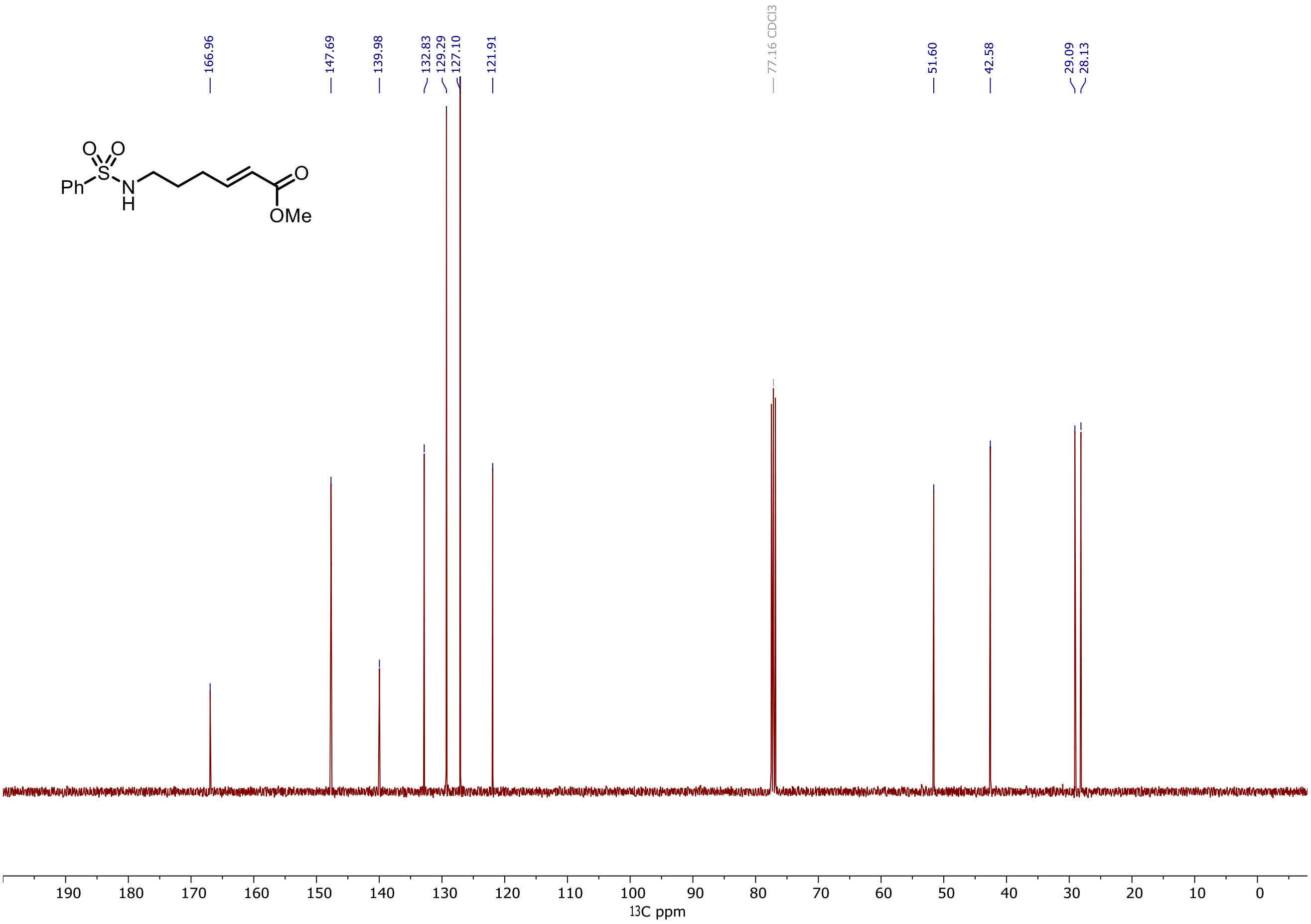

[back to table of contents]

^{1}H NMR (400 MHz, $CDCl_3$) of compound **S5**

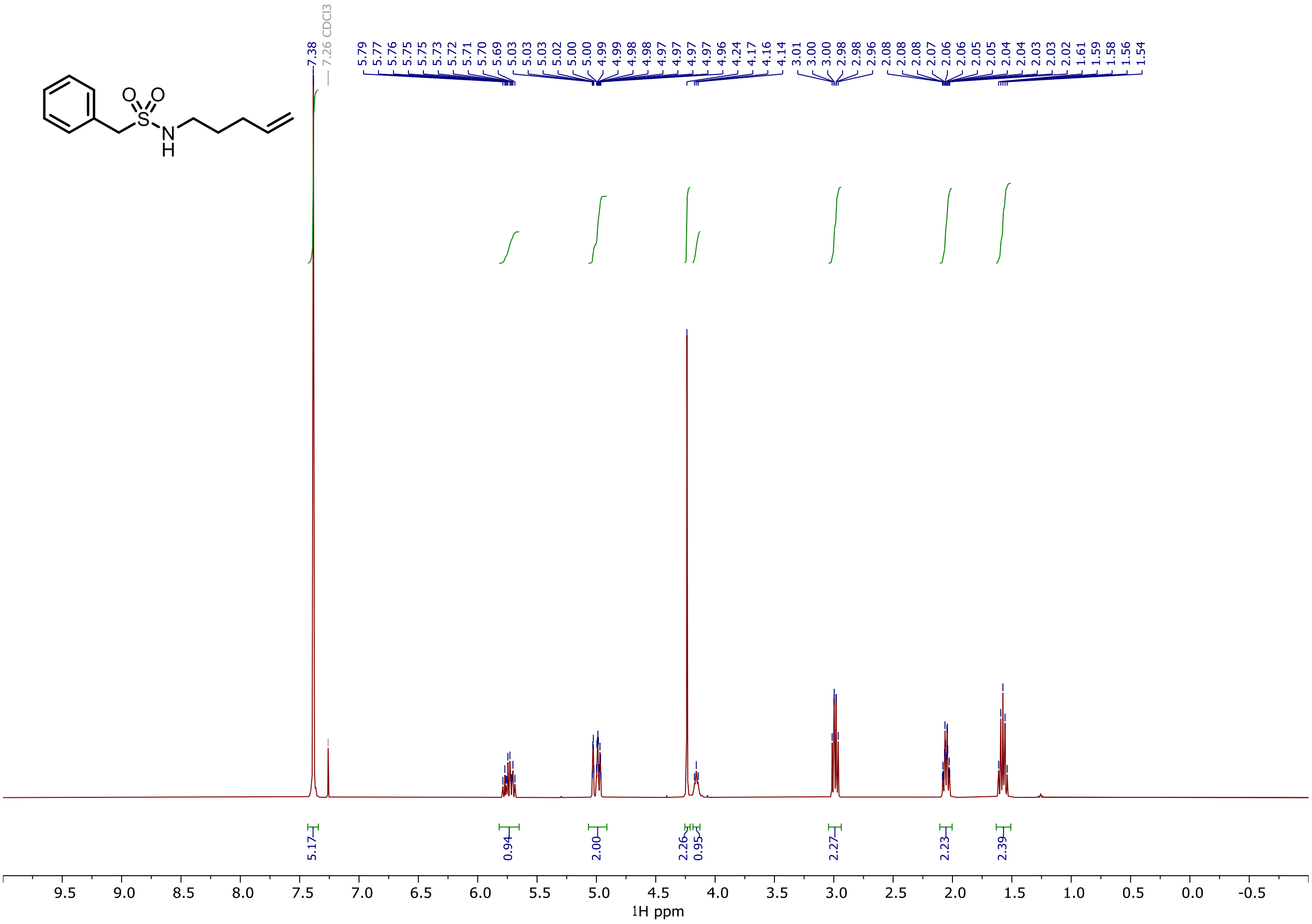

^{13}C NMR (101 MHz, $CDCl_3$) of compound **S5**

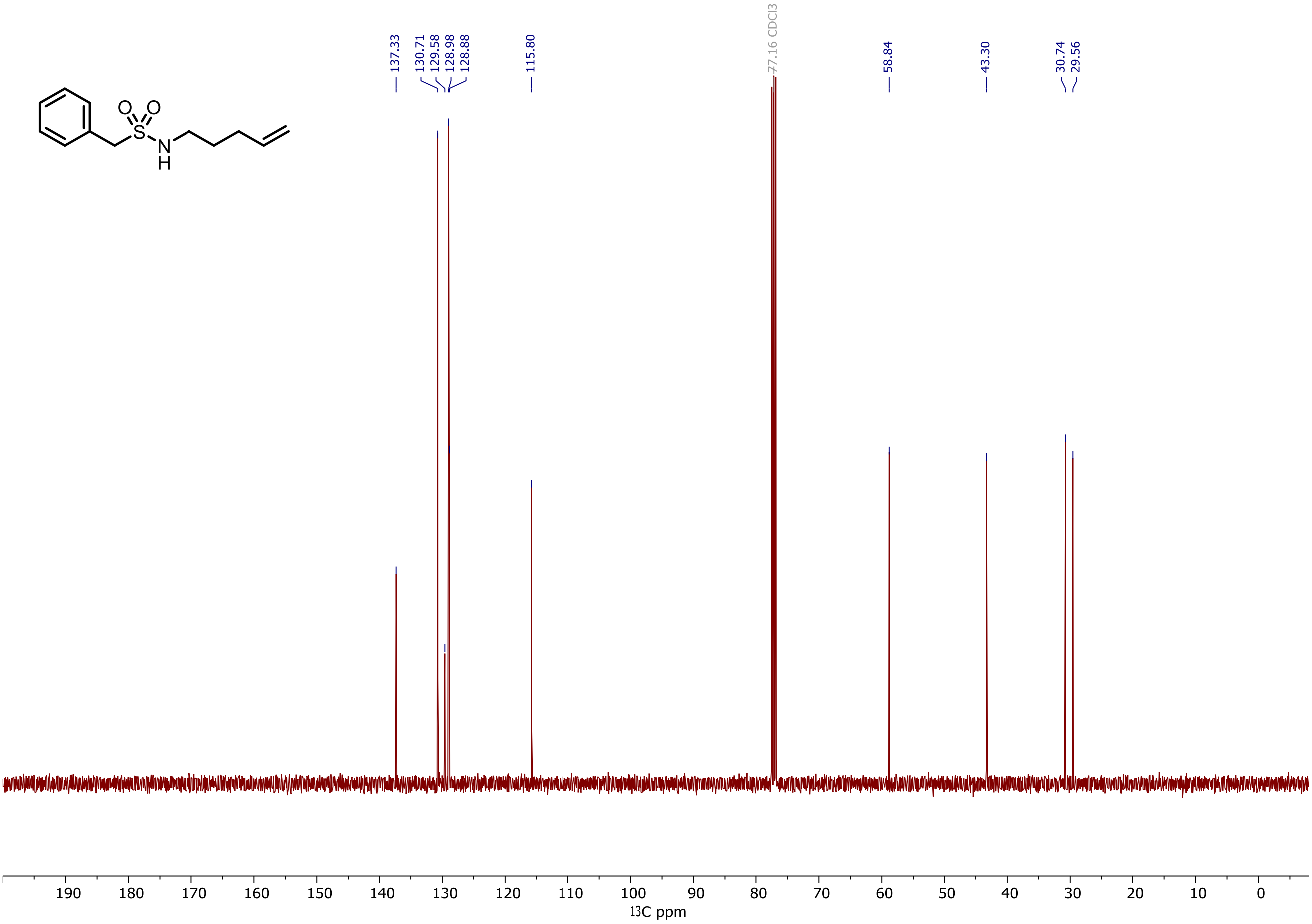

[back to table of contents]

^{1}H NMR (600 MHz, $CDCl_3$) of compound **7b**

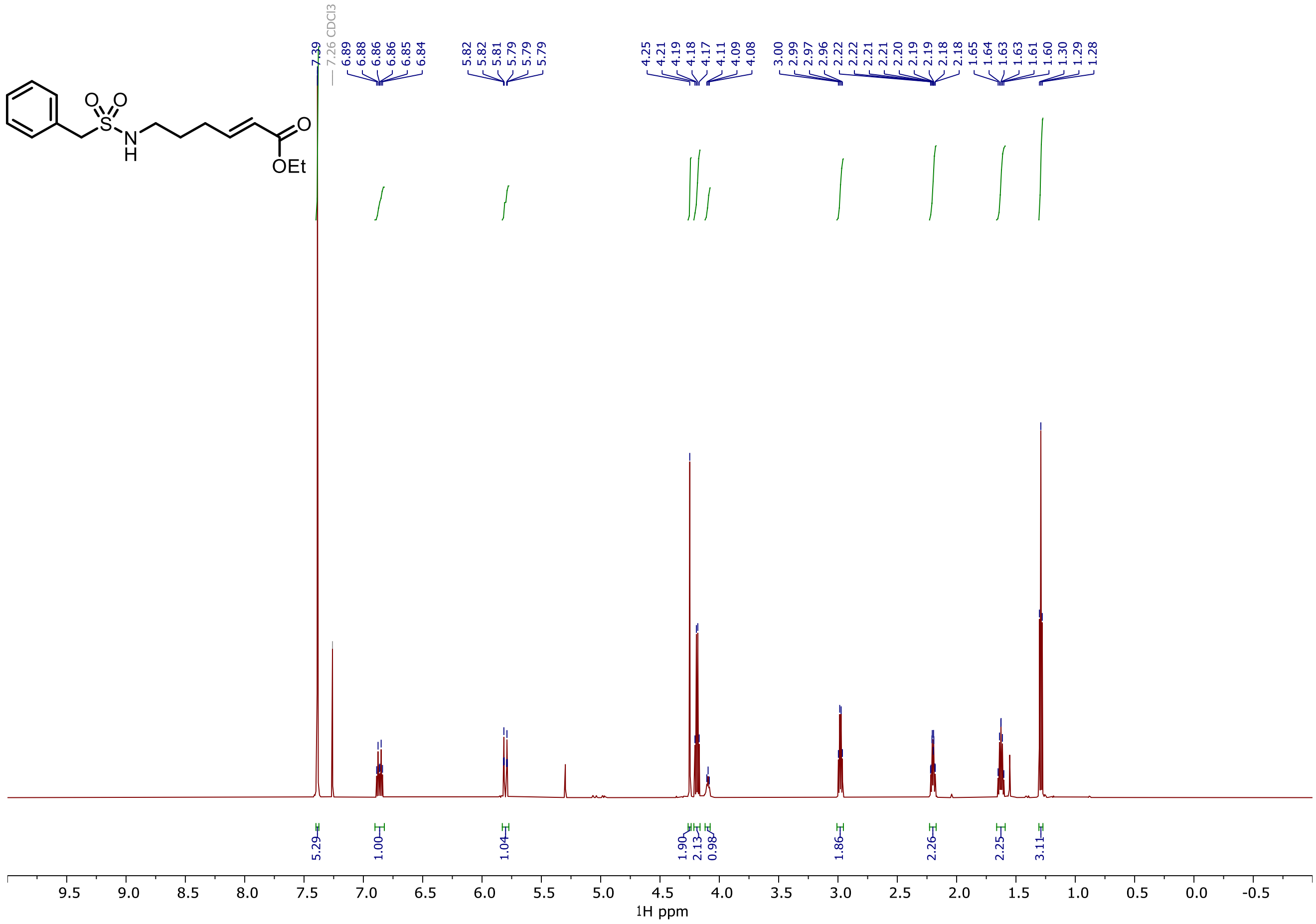

^{13}C NMR (151 MHz, $CDCl_3$) of compound **7b**

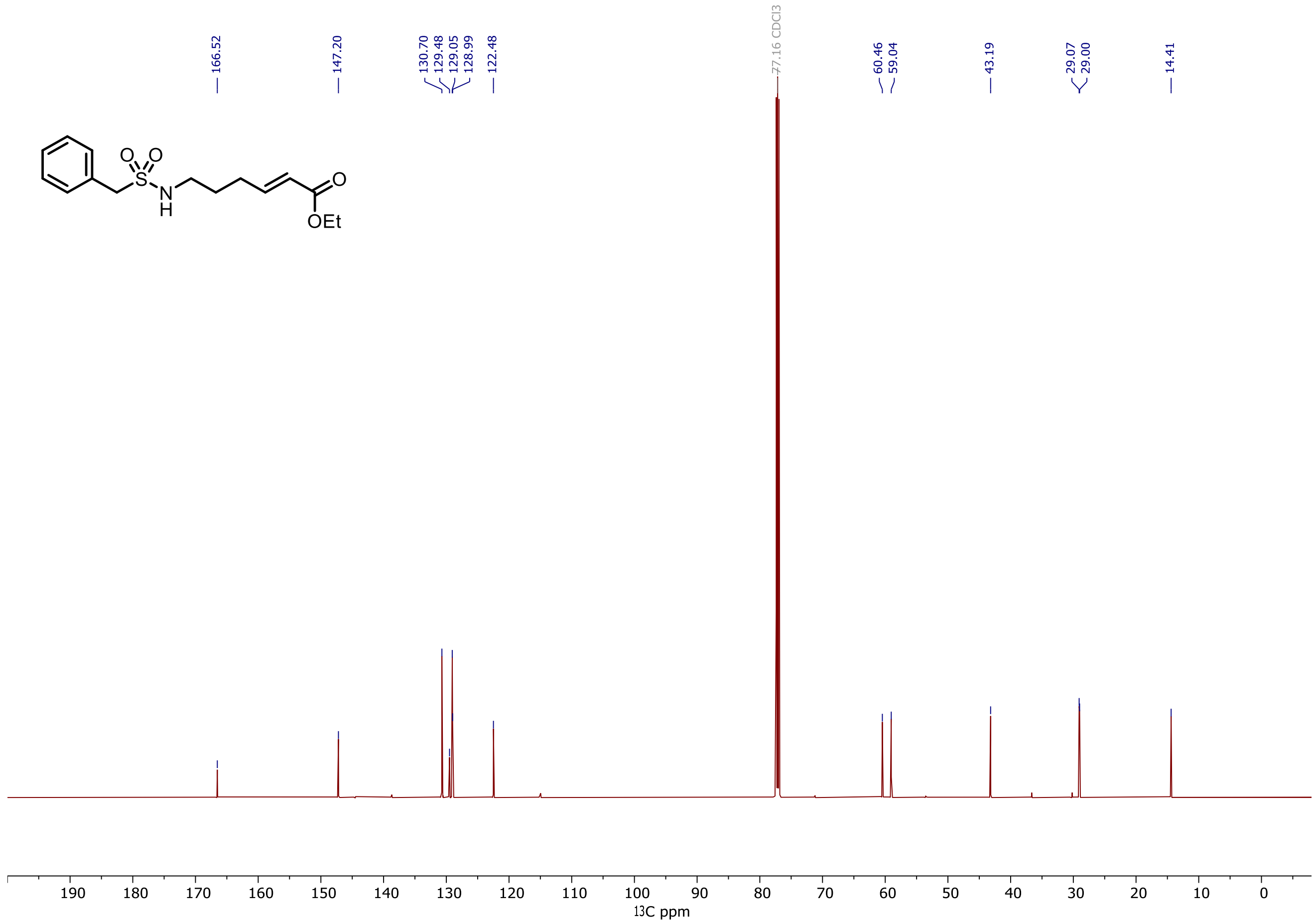

[back to table of contents]

[1]**H NMR** (600 MHz, $CDCl_3$) of compound **7c**

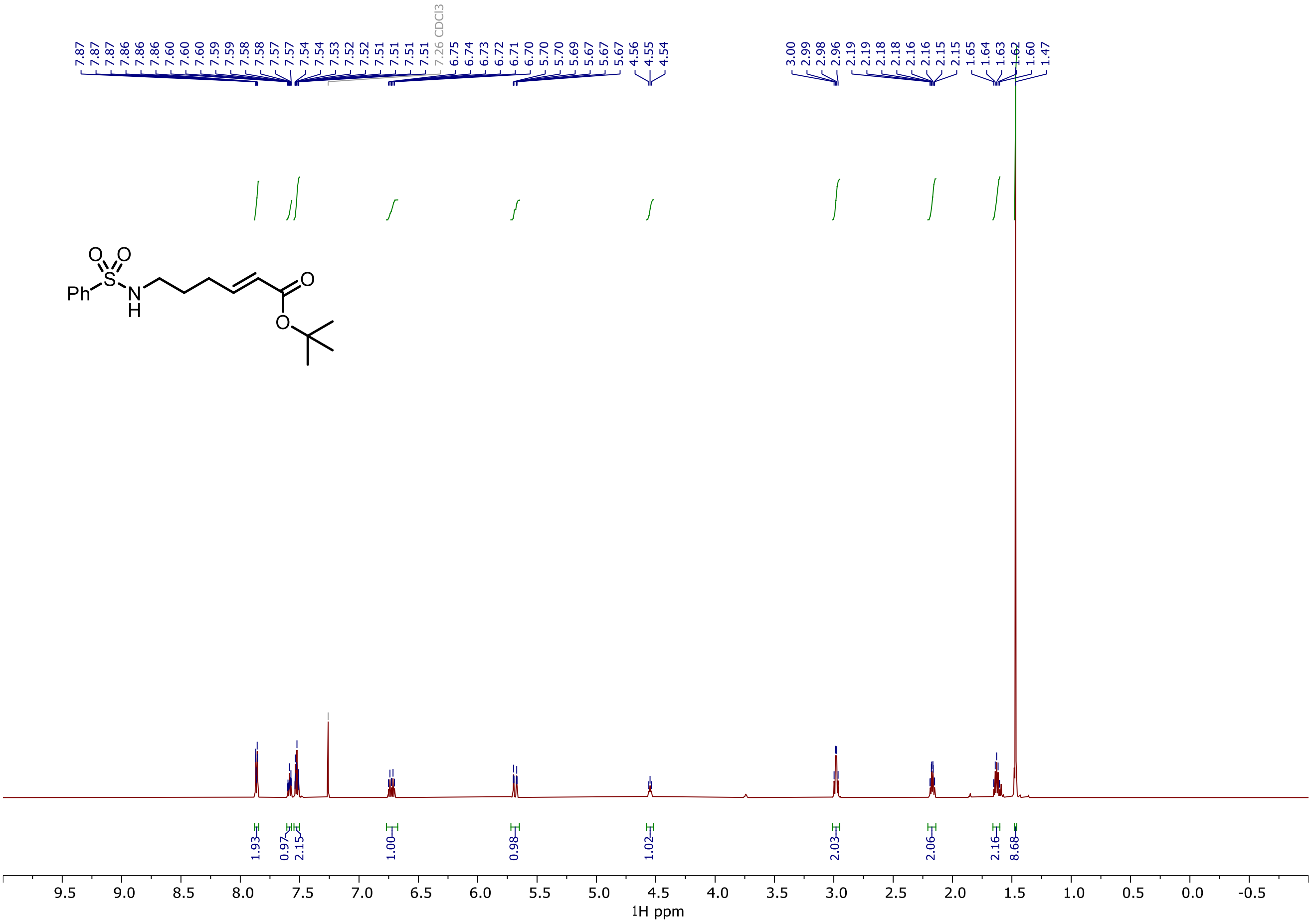

[13]**C NMR** (151 MHz, $CDCl_3$) of compound **7c**

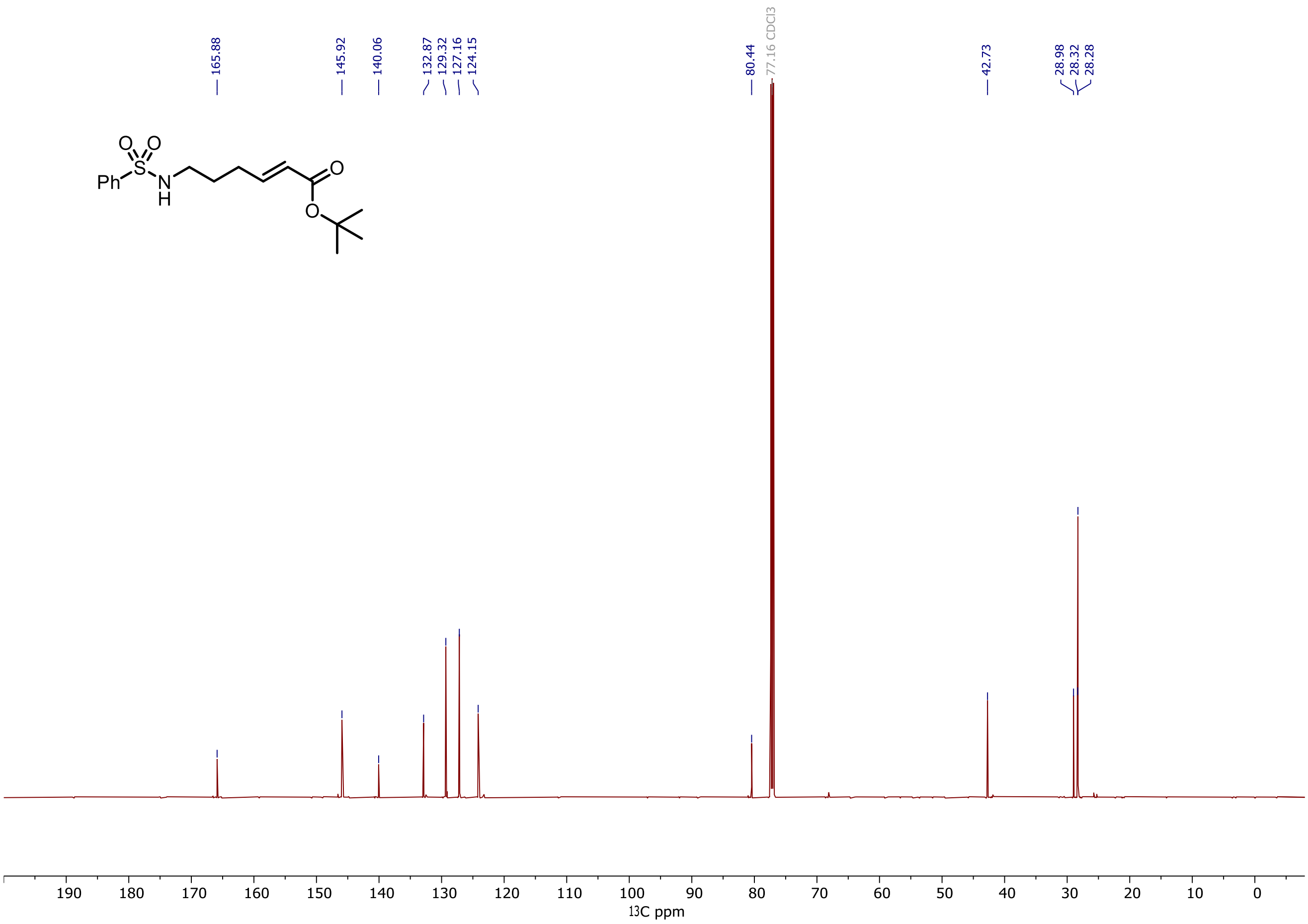

[back to table of contents]

^{1}H NMR (400 MHz, $CDCl_3$) of compound **7d**

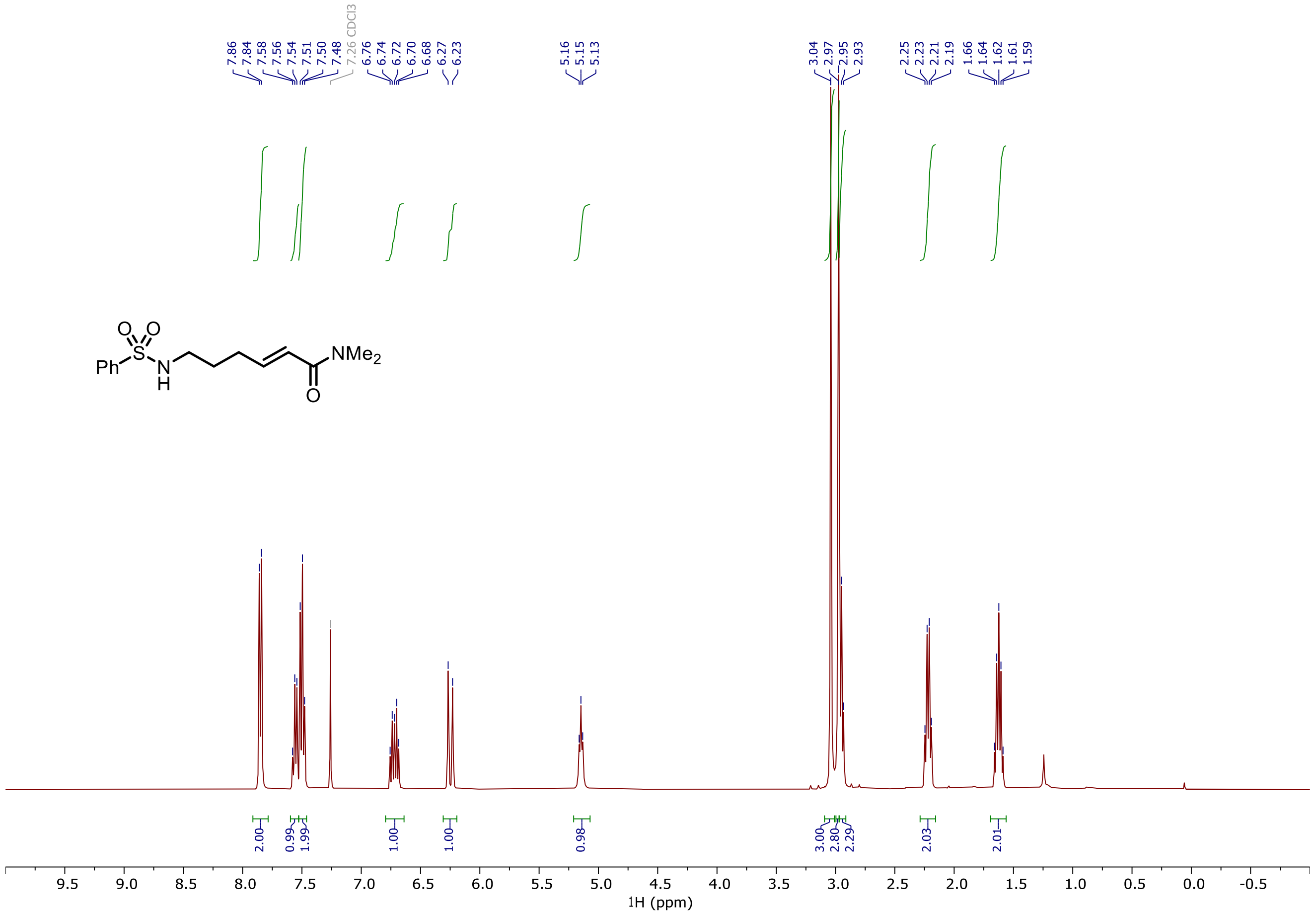

^{13}C NMR (101 MHz, $CDCl_3$) of compound **7d**

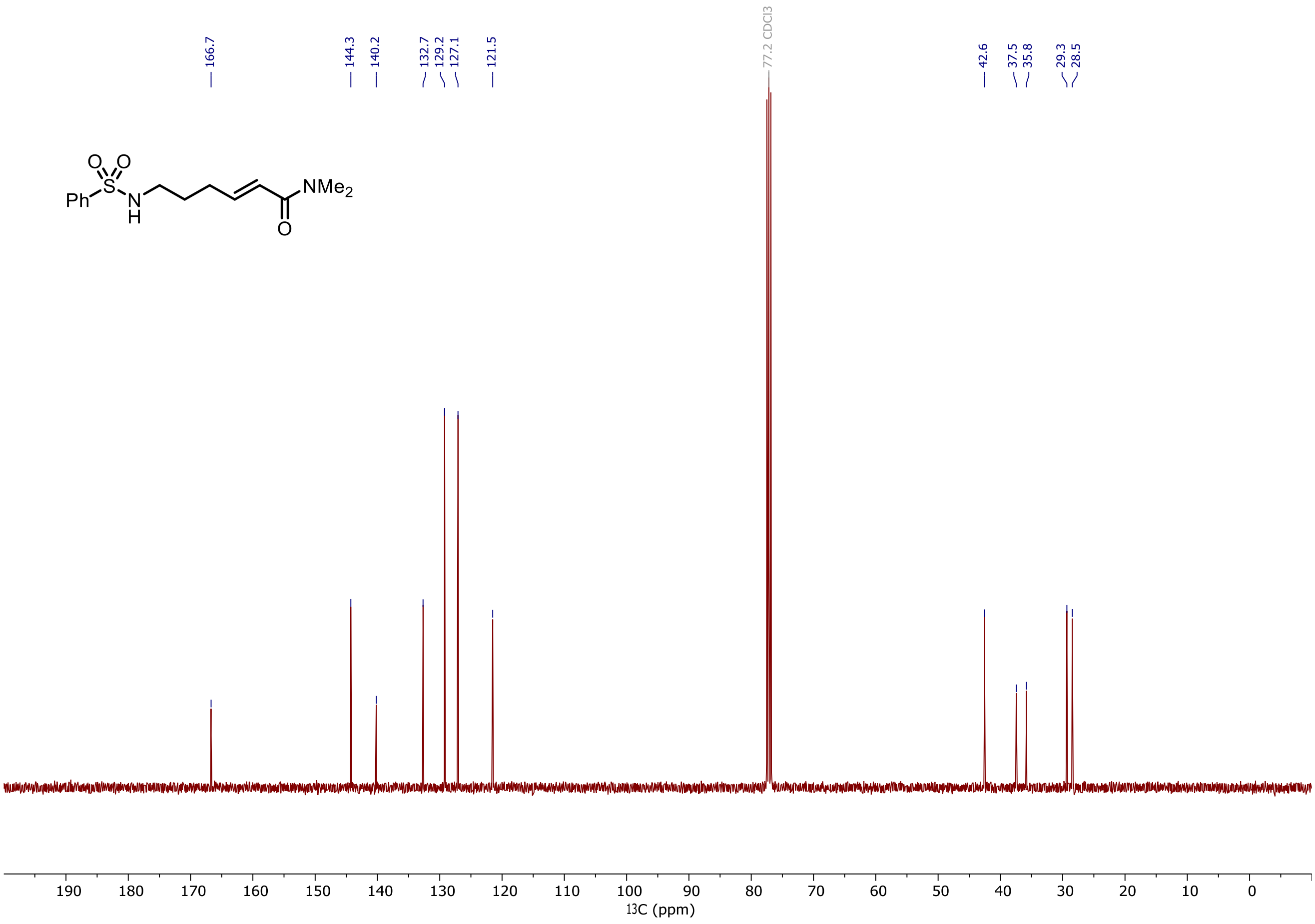

[back to table of contents]

^{1}H NMR (400 MHz, $CDCl_3$) of compound **7e**

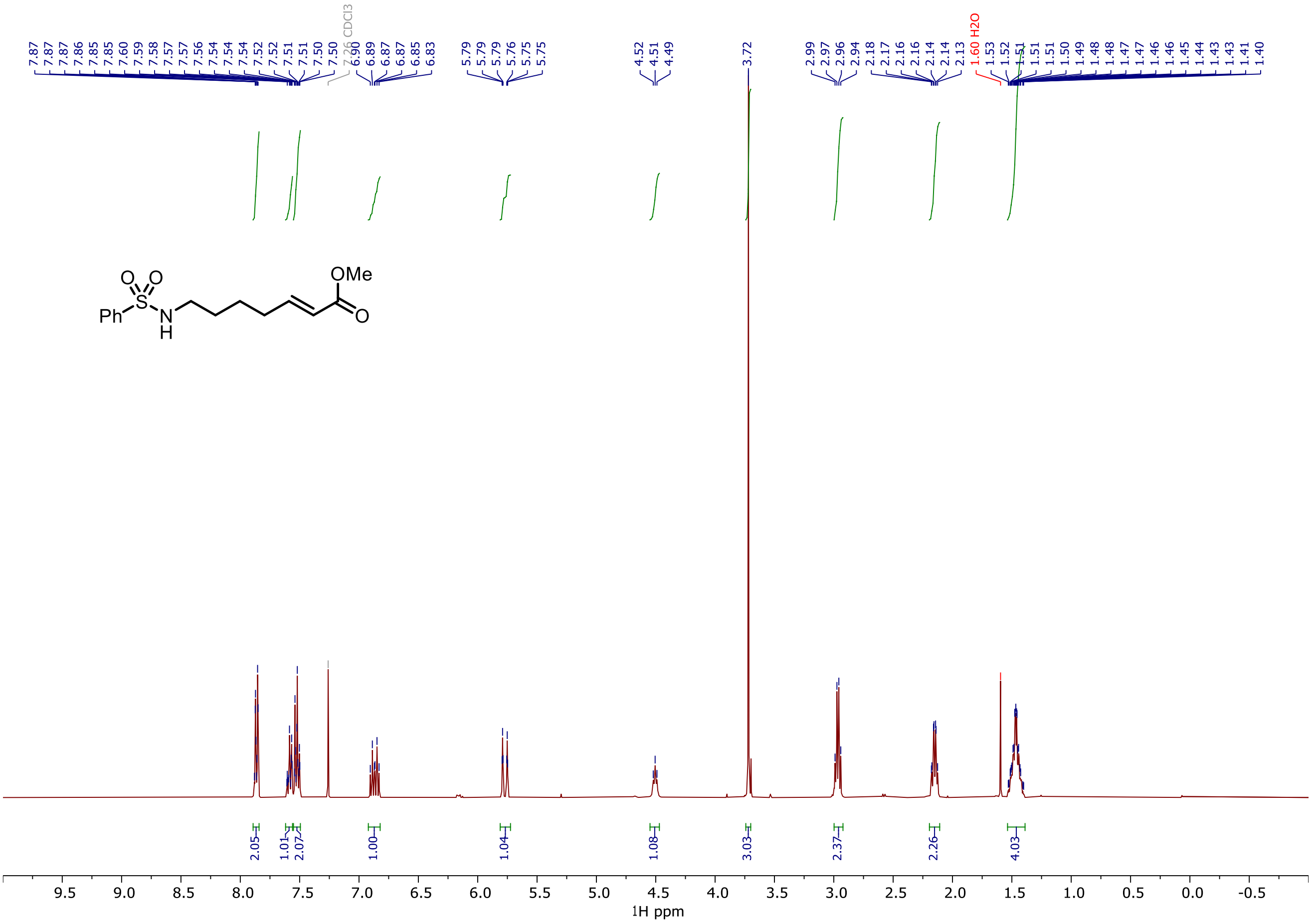

^{13}C NMR (101 MHz, $CDCl_3$) of compound **7e**

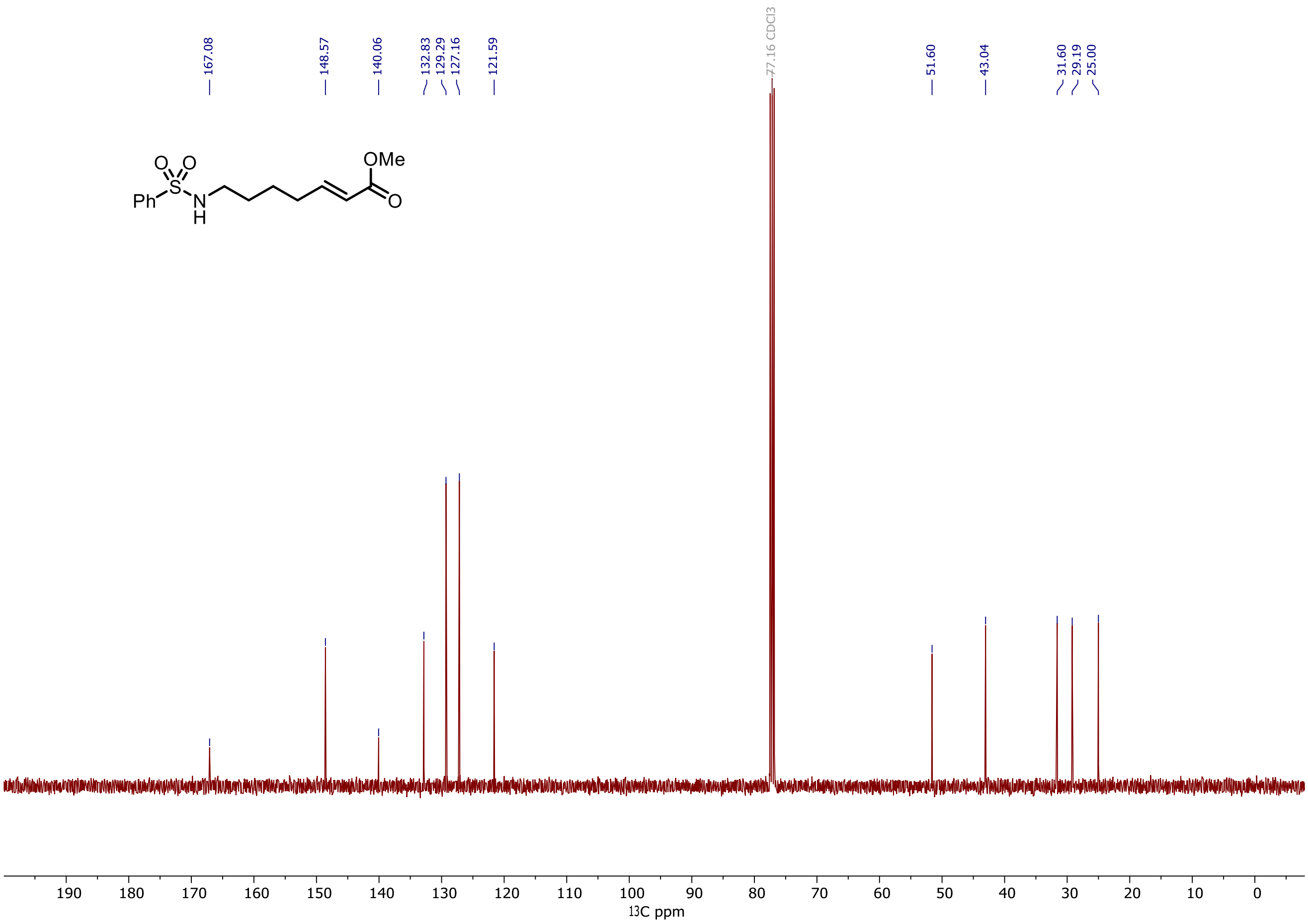

[back to table of contents]

^{1}H NMR (400 MHz, $CDCl_3$) of compound **7f**

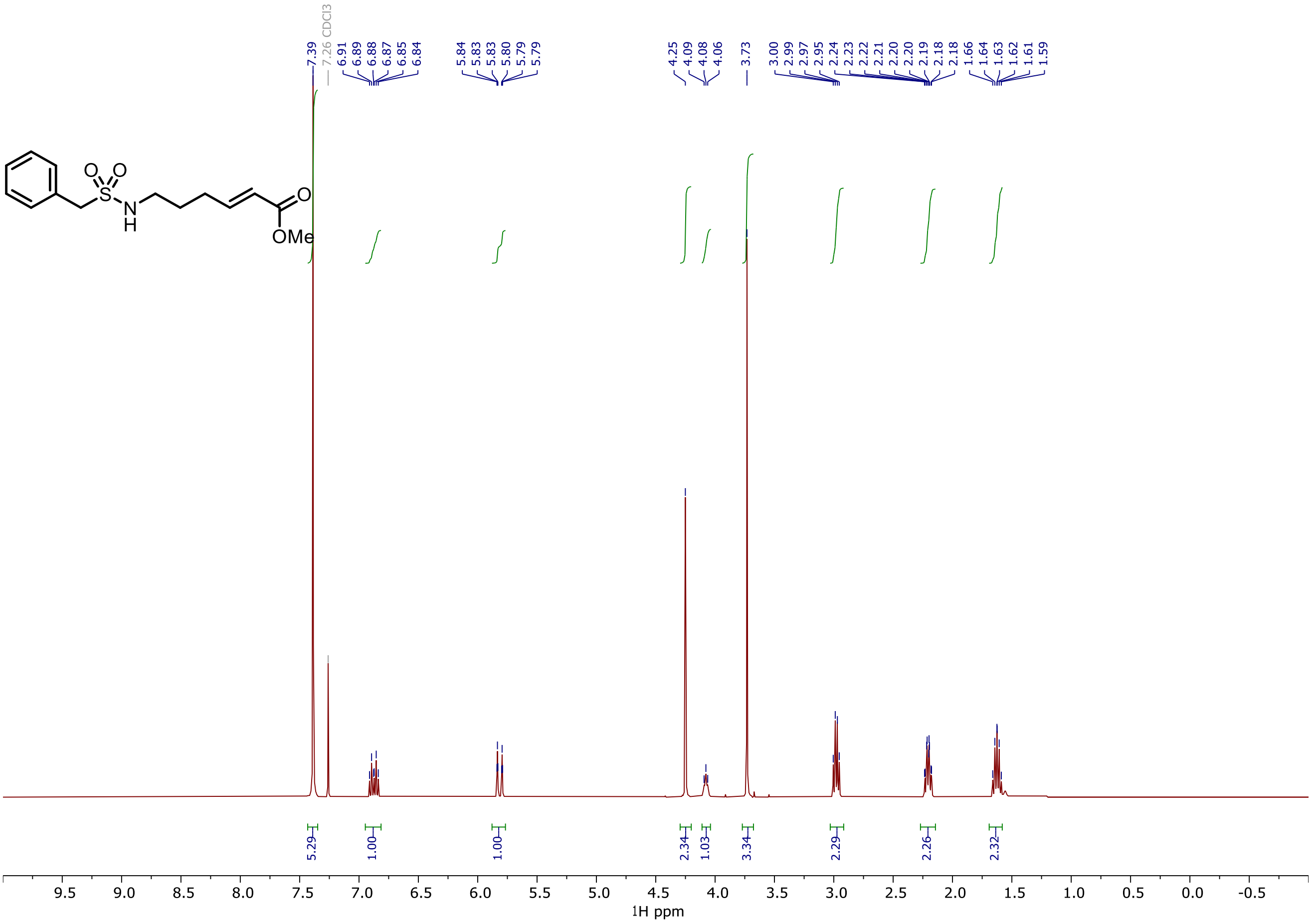

^{13}C NMR (101 MHz, $CDCl_3$) of compound **7f**

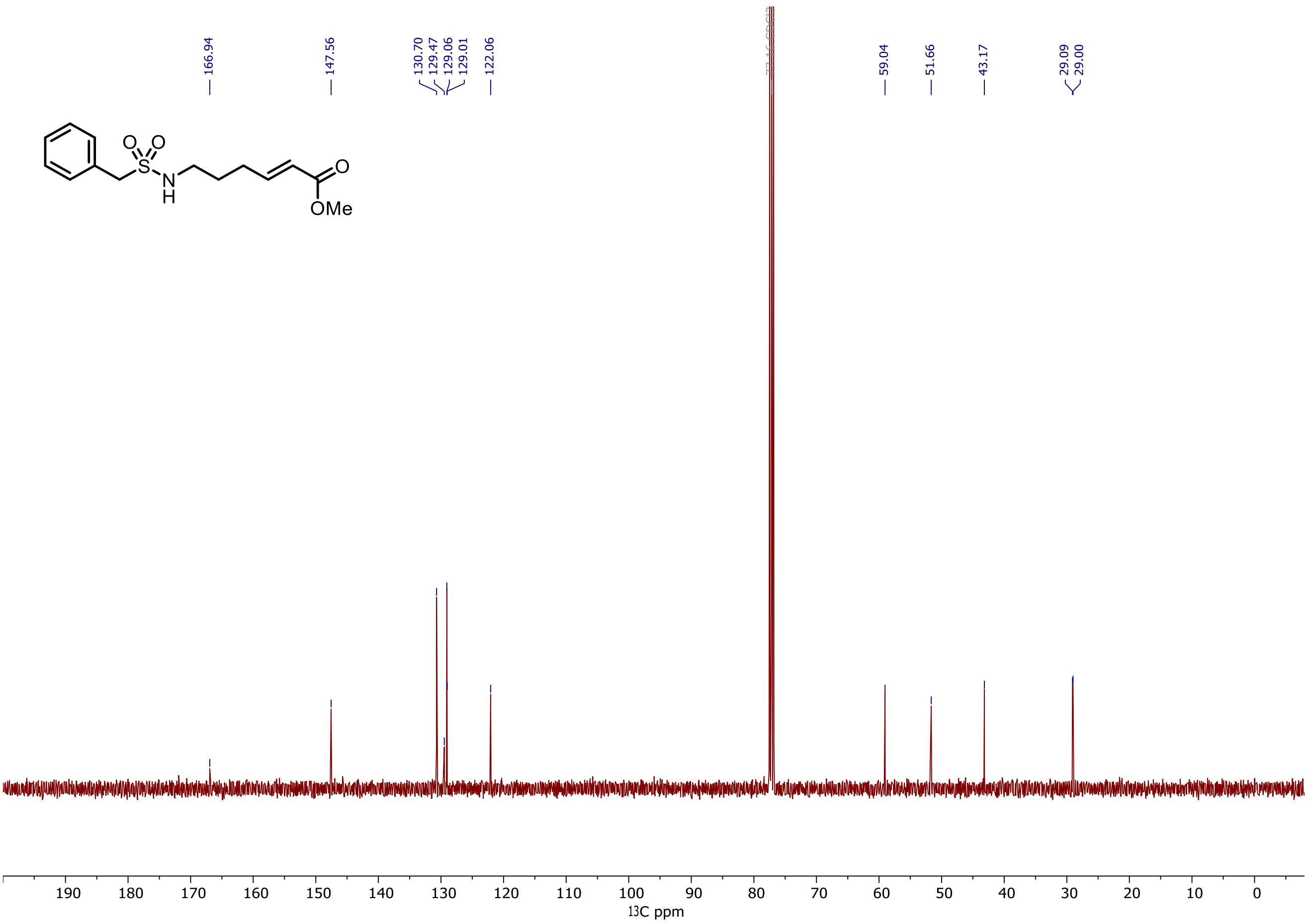

[back to table of contents]

^{1}H NMR (400 MHz, $CDCl_3$) of compound **S8**

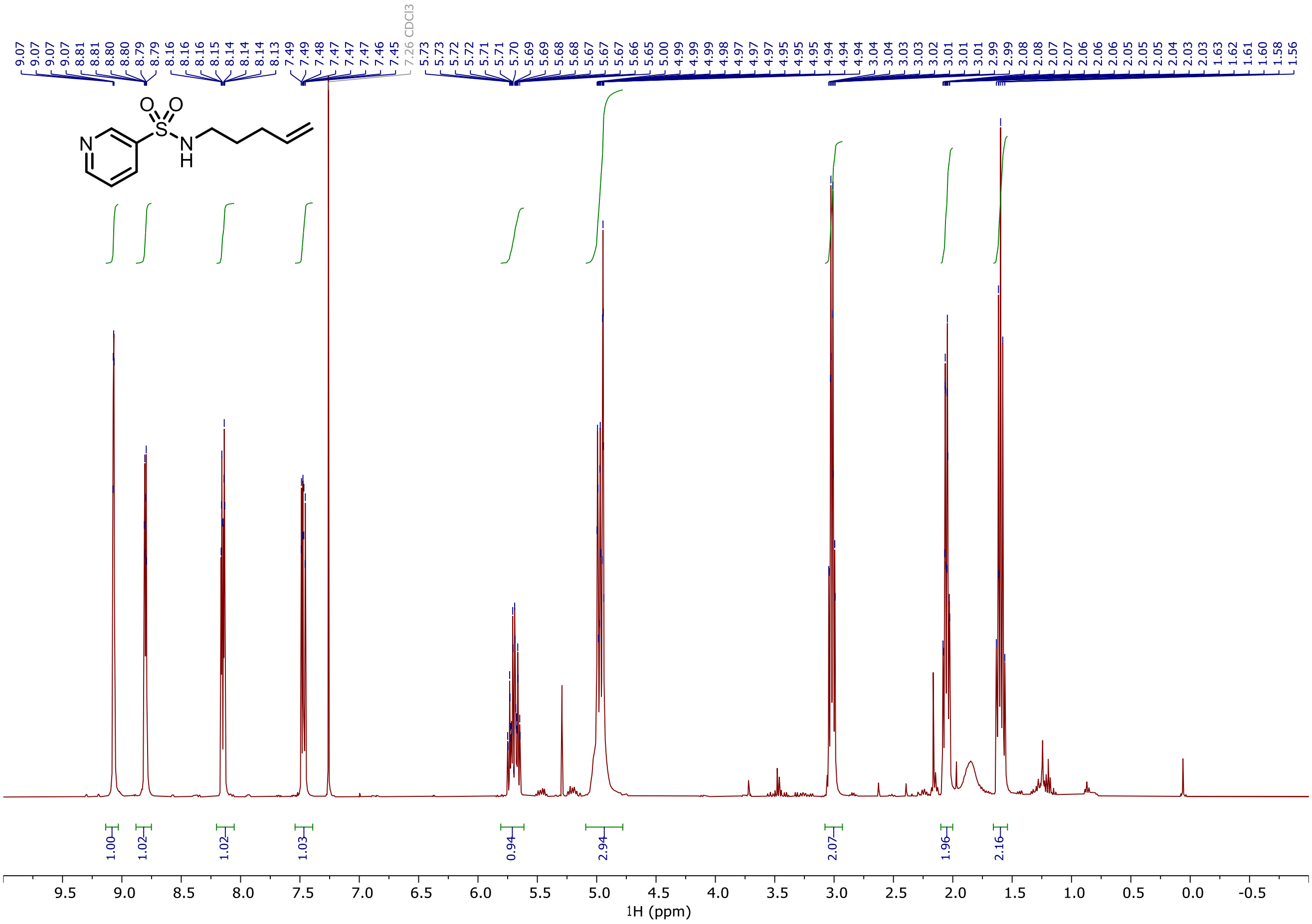

^{13}C NMR (101 MHz, $CDCl_3$) of compound **S8**

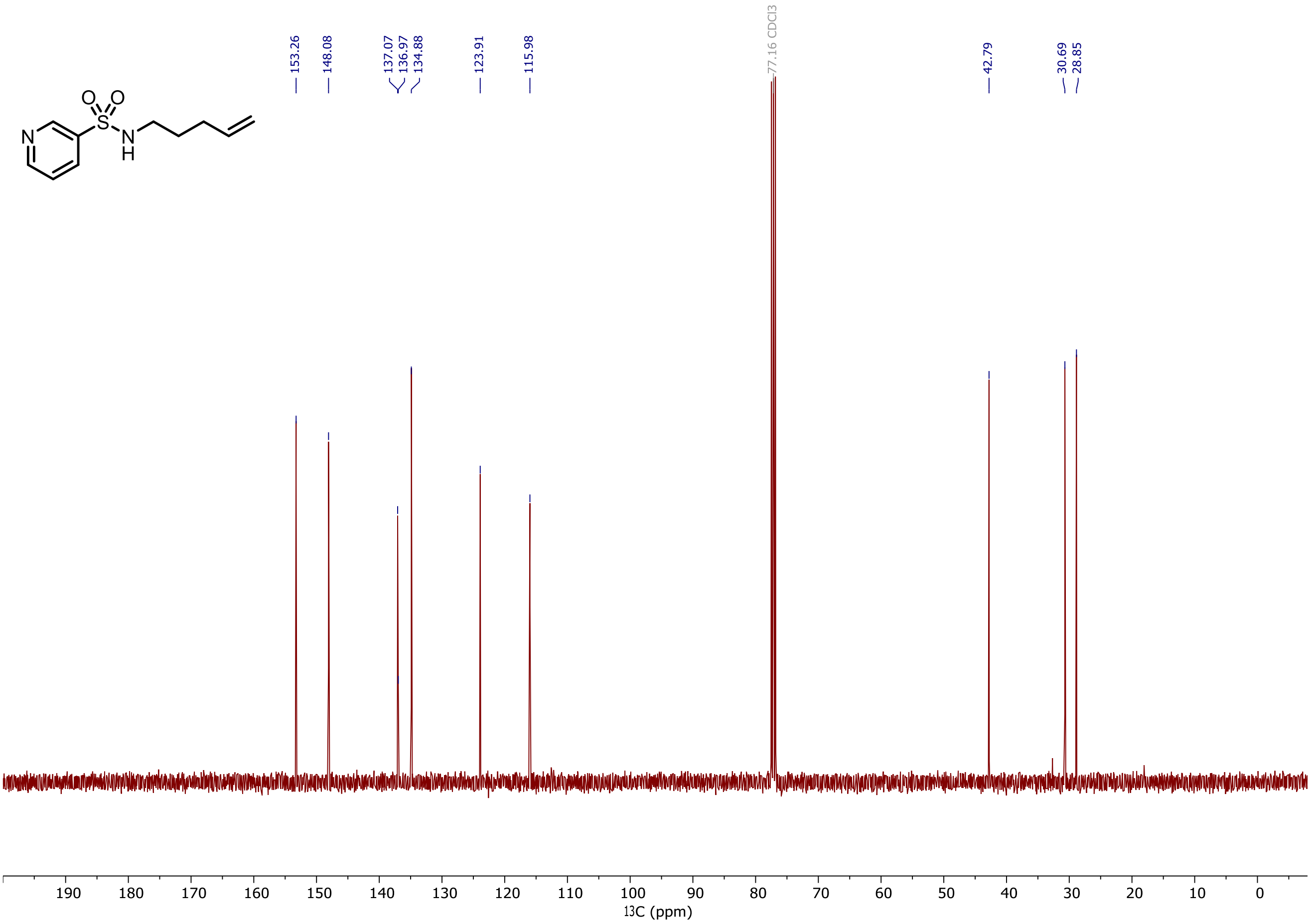

[back to table of contents]

^{1}H NMR (400 MHz, $CDCl_3$) of compound **7g**

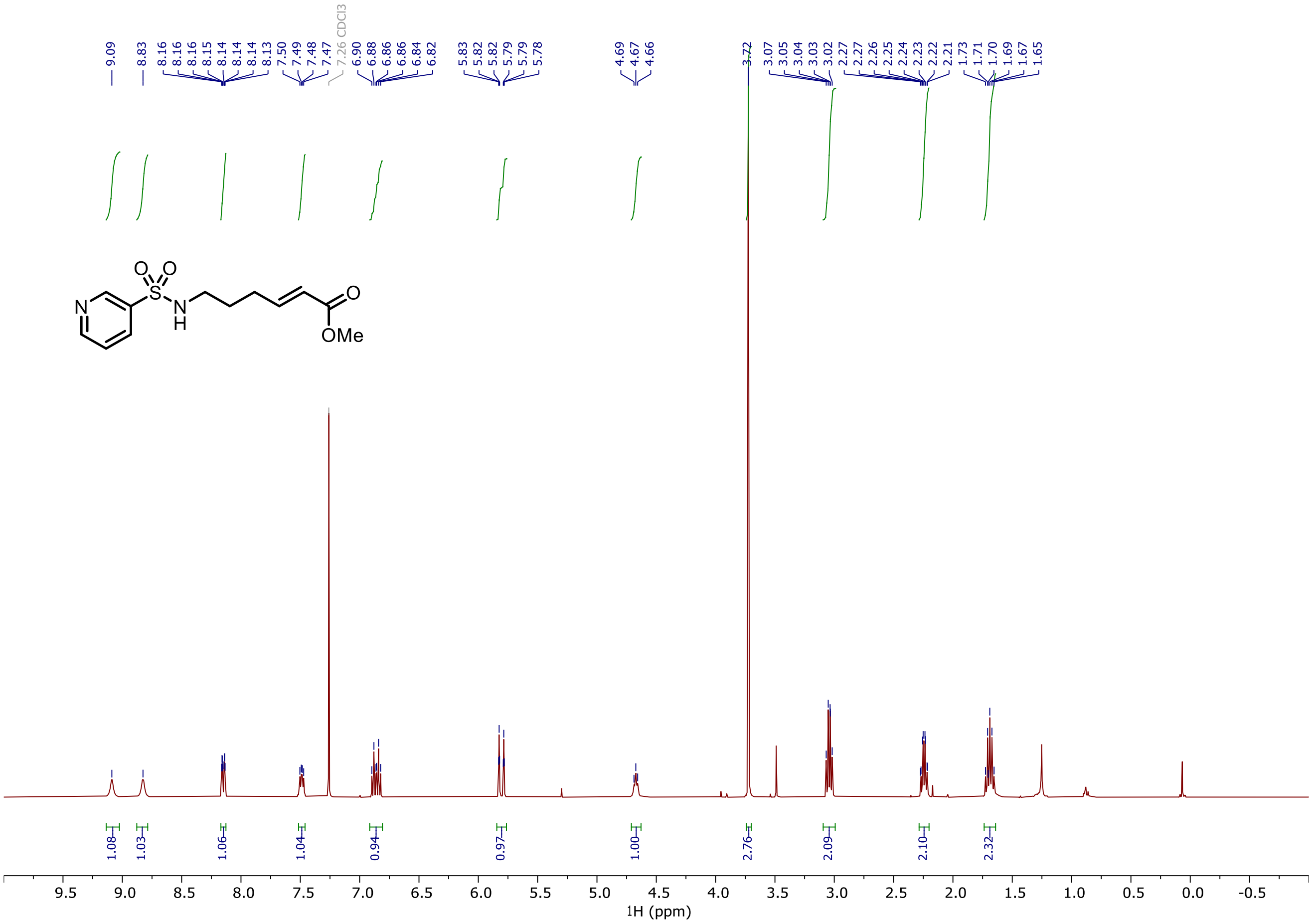

^{13}C NMR (101 MHz, $CDCl_3$) of compound **7g**

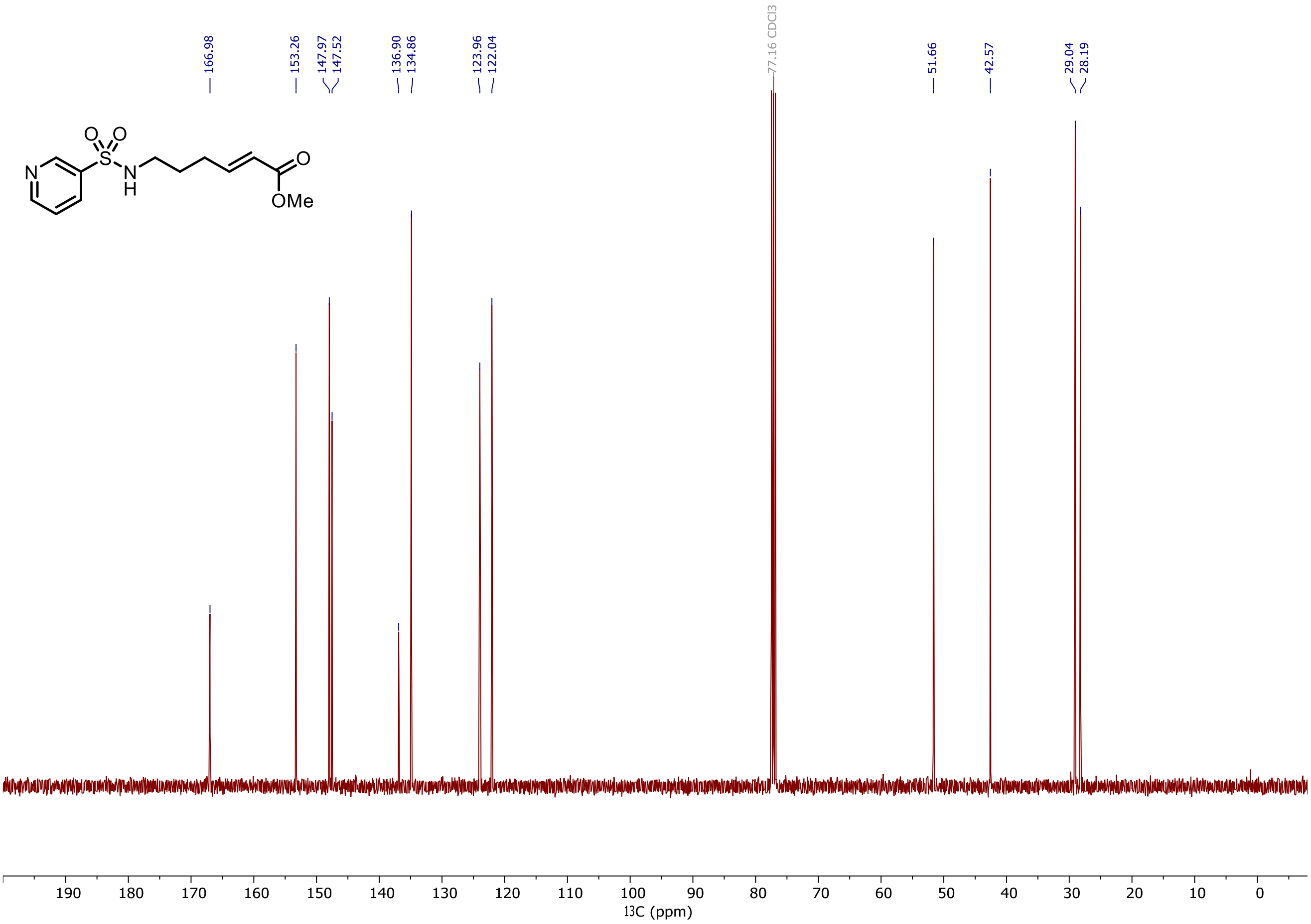

[back to table of contents]

^{1}H NMR (400 MHz, $CDCl_3$) of compound **S9**

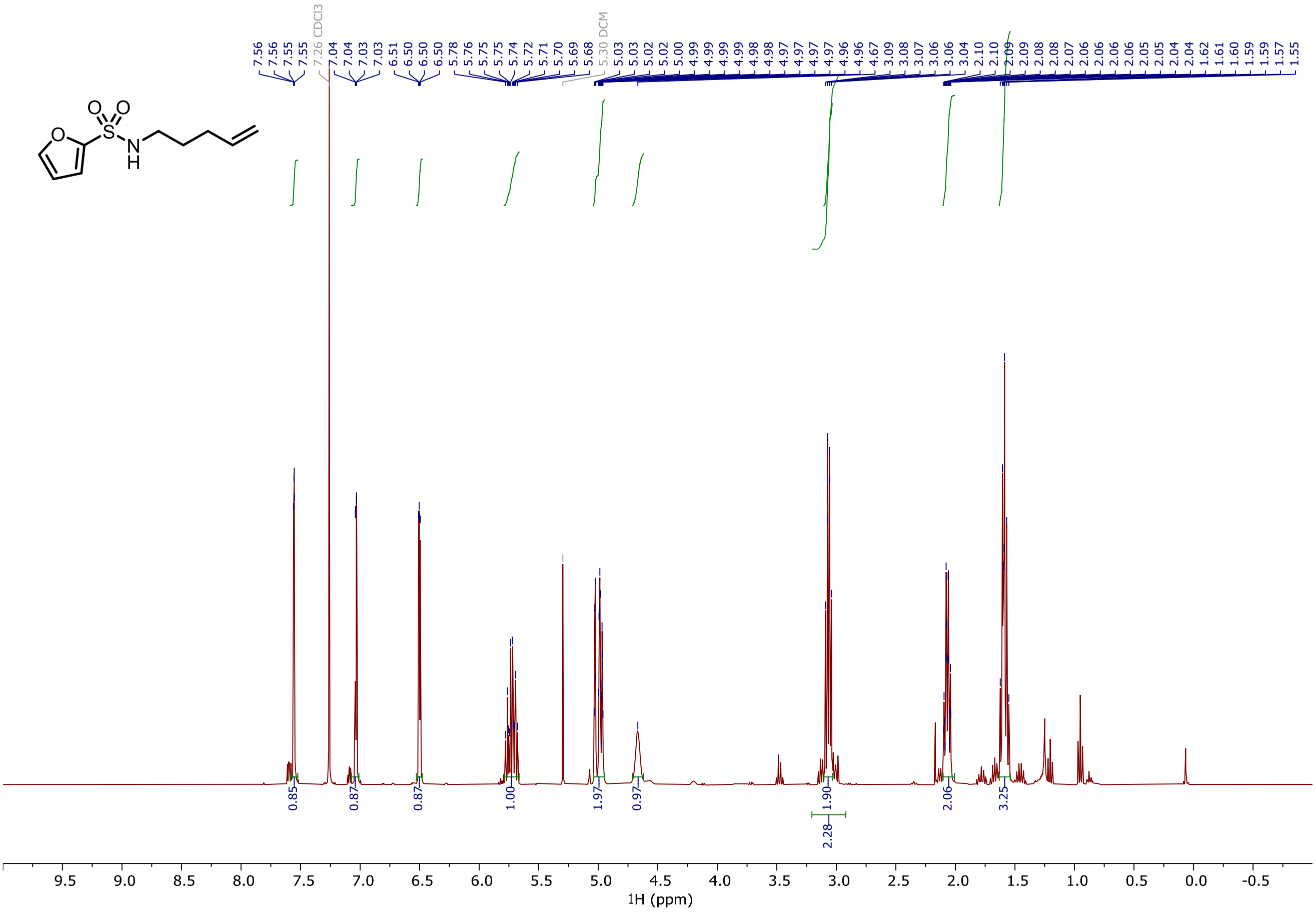

^{13}C NMR (101 MHz, $CDCl_3$) of compound **S9**

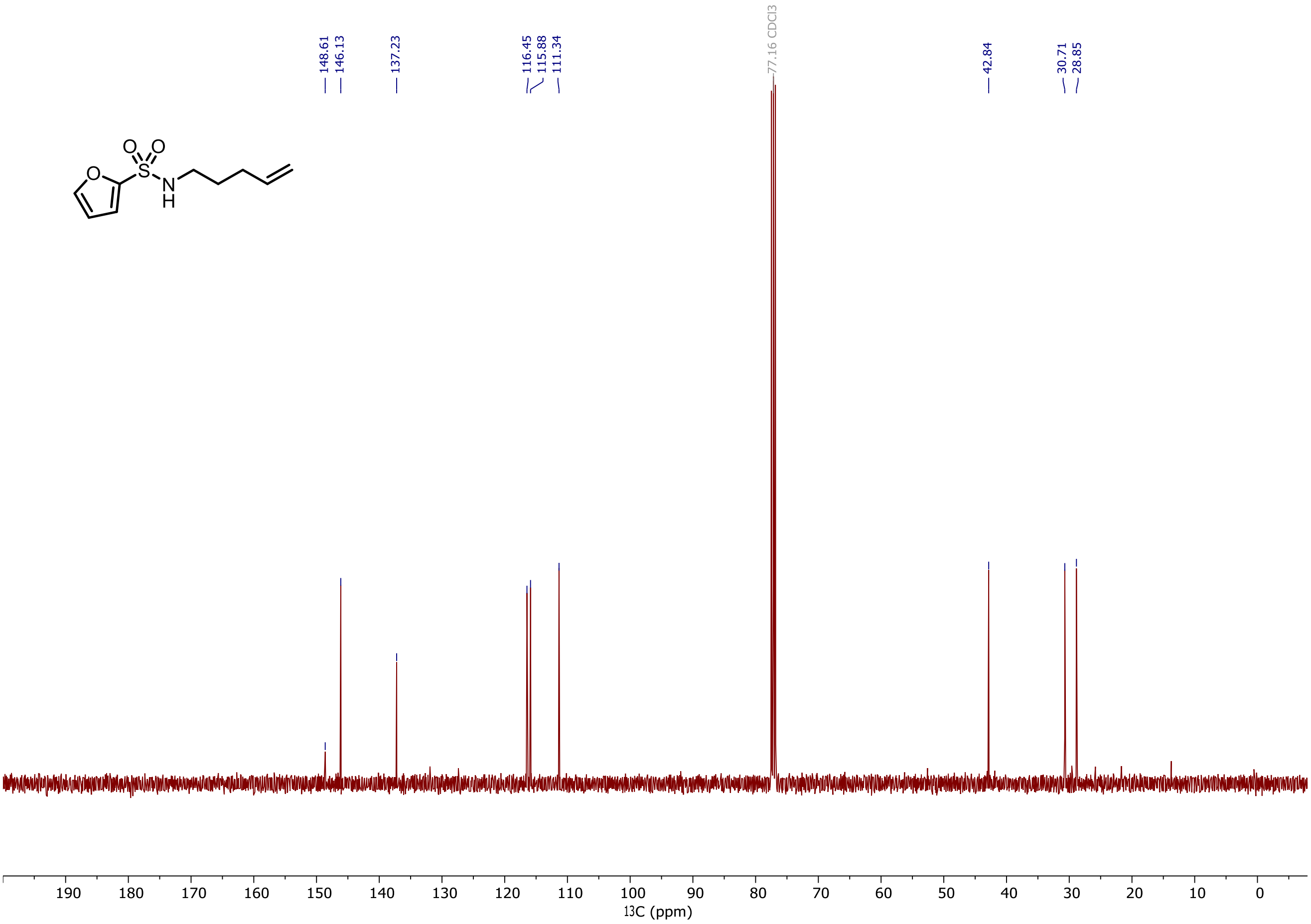

[back to table of contents]

^{1}H NMR (400 MHz, $CDCl_3$) of compound **7h**

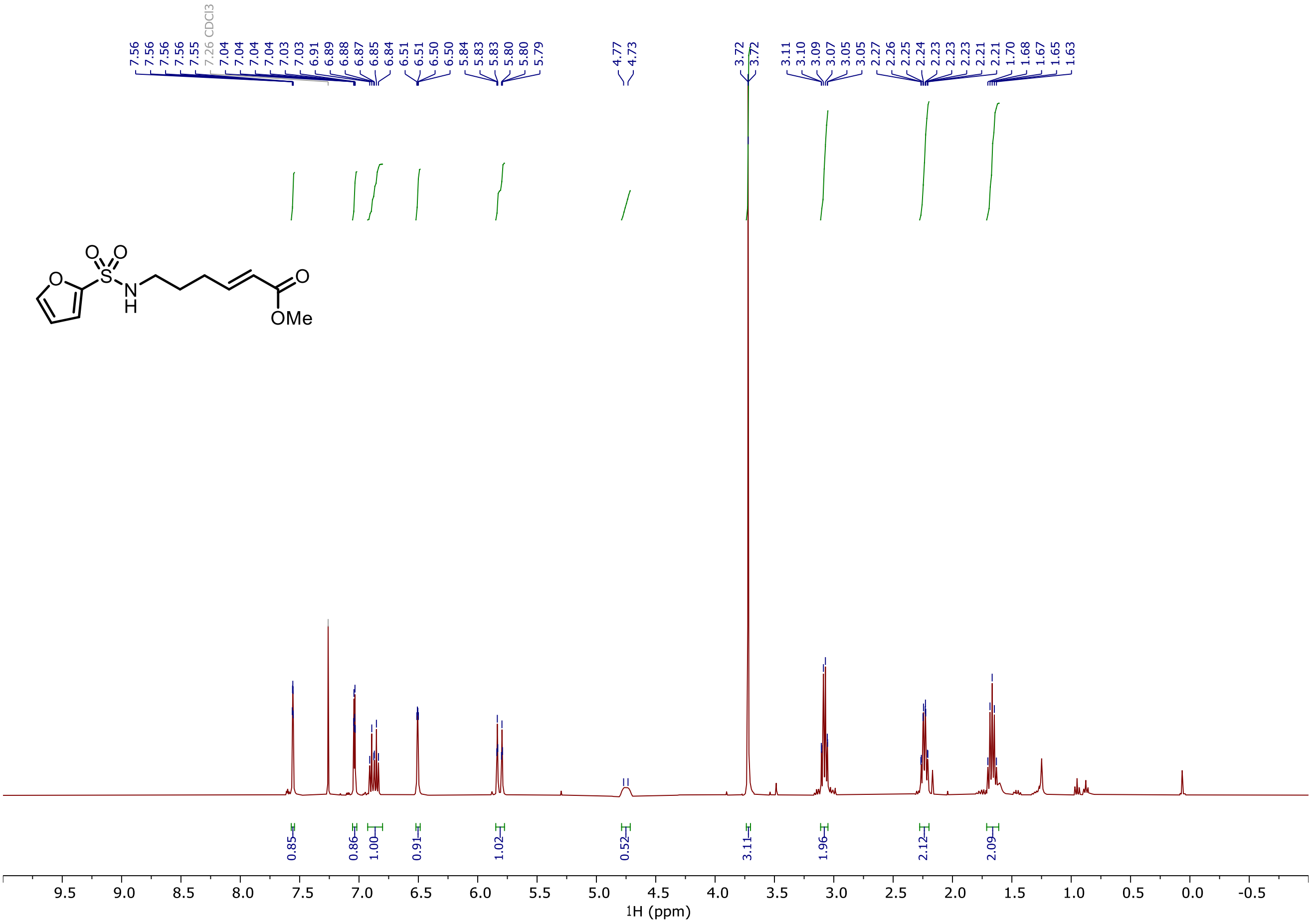

^{13}C NMR (101 MHz, $CDCl_3$) of compound **7h**

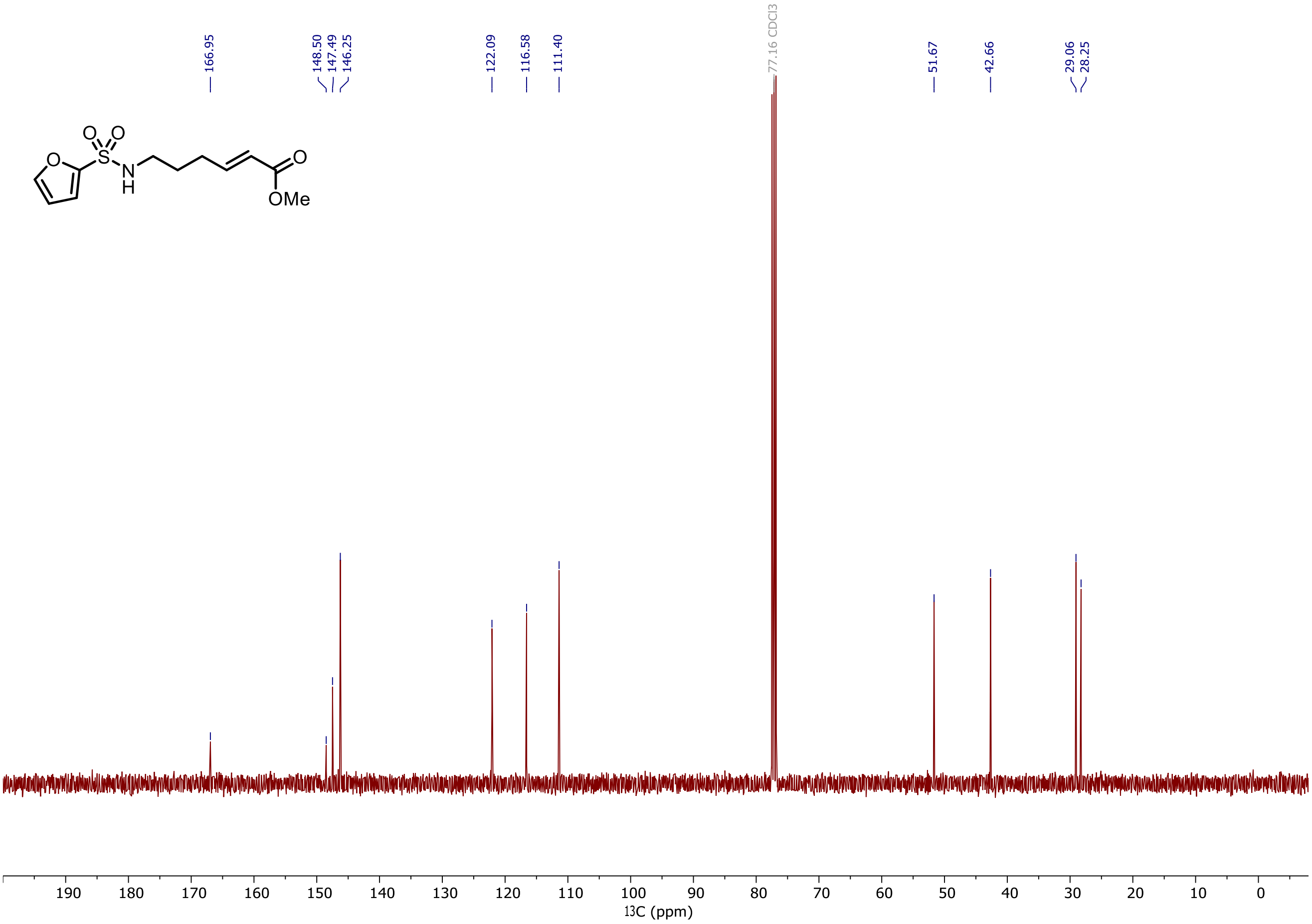

[back to table of contents]

^{1}H NMR (400 MHz, $CDCl_3$) of compound **S10**

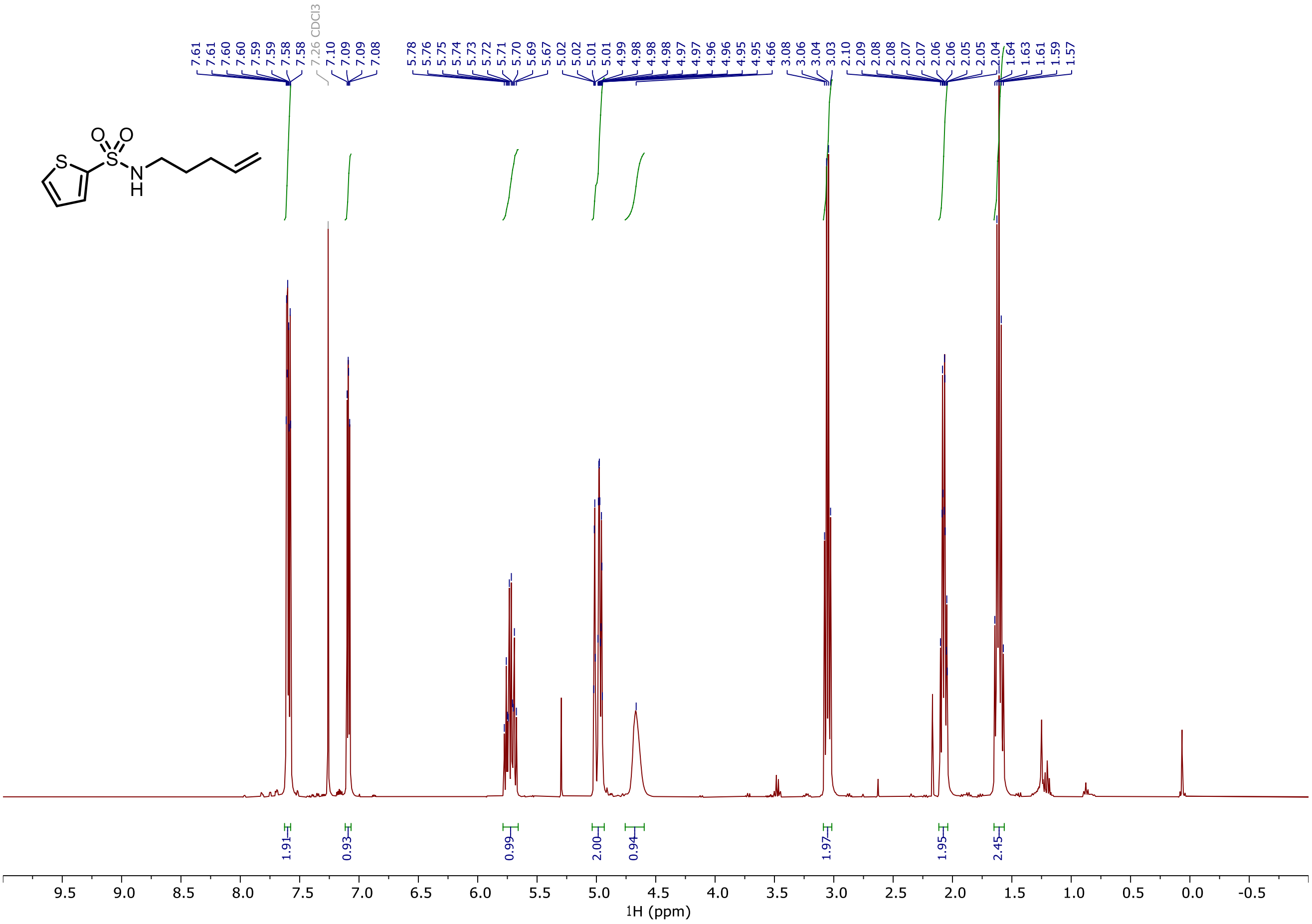

^{13}C NMR (101 MHz, $CDCl_3$) of compound **S10**

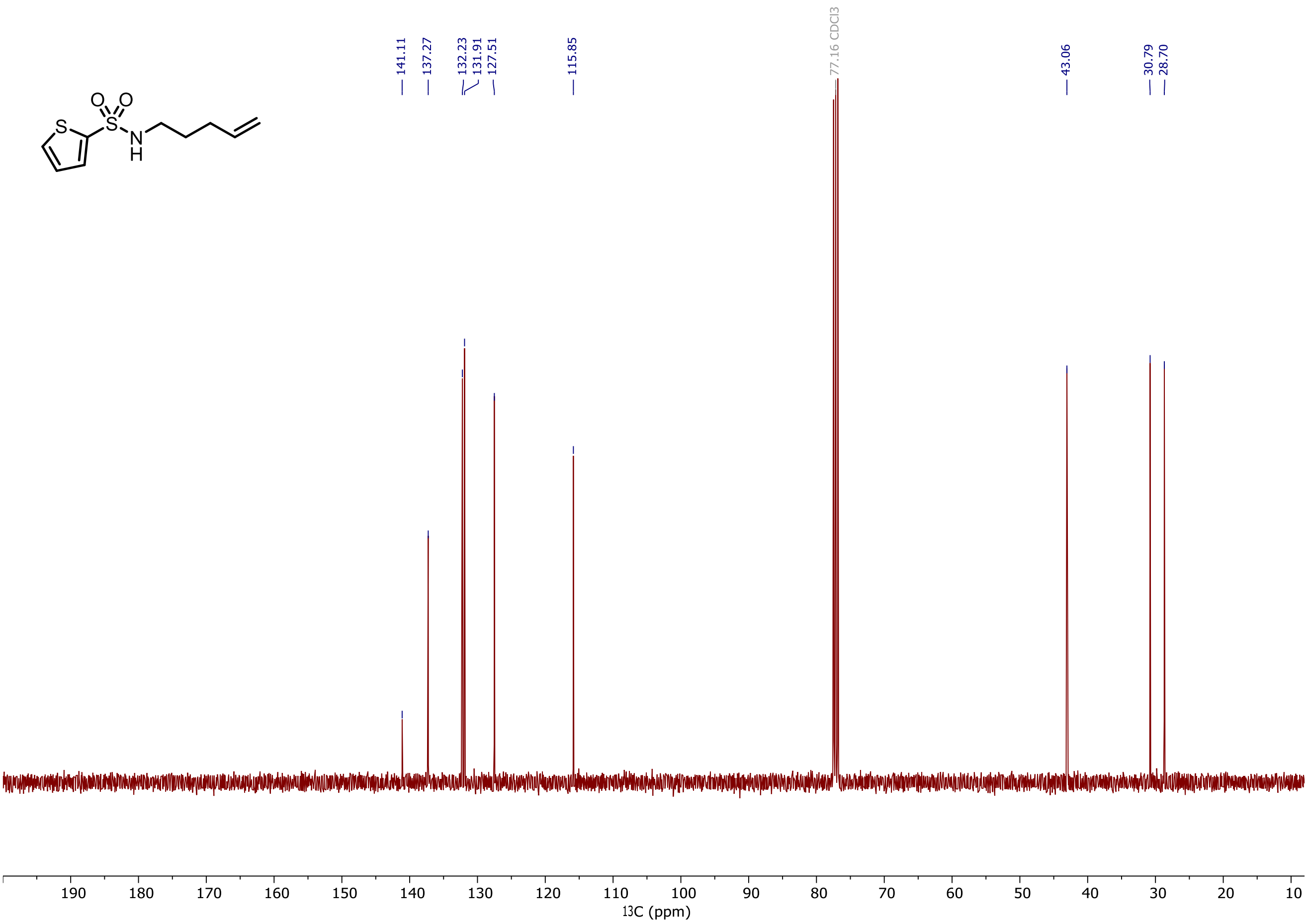

[back to table of contents]

¹H NMR (400 MHz, $CDCl_3$) of compound **7i**

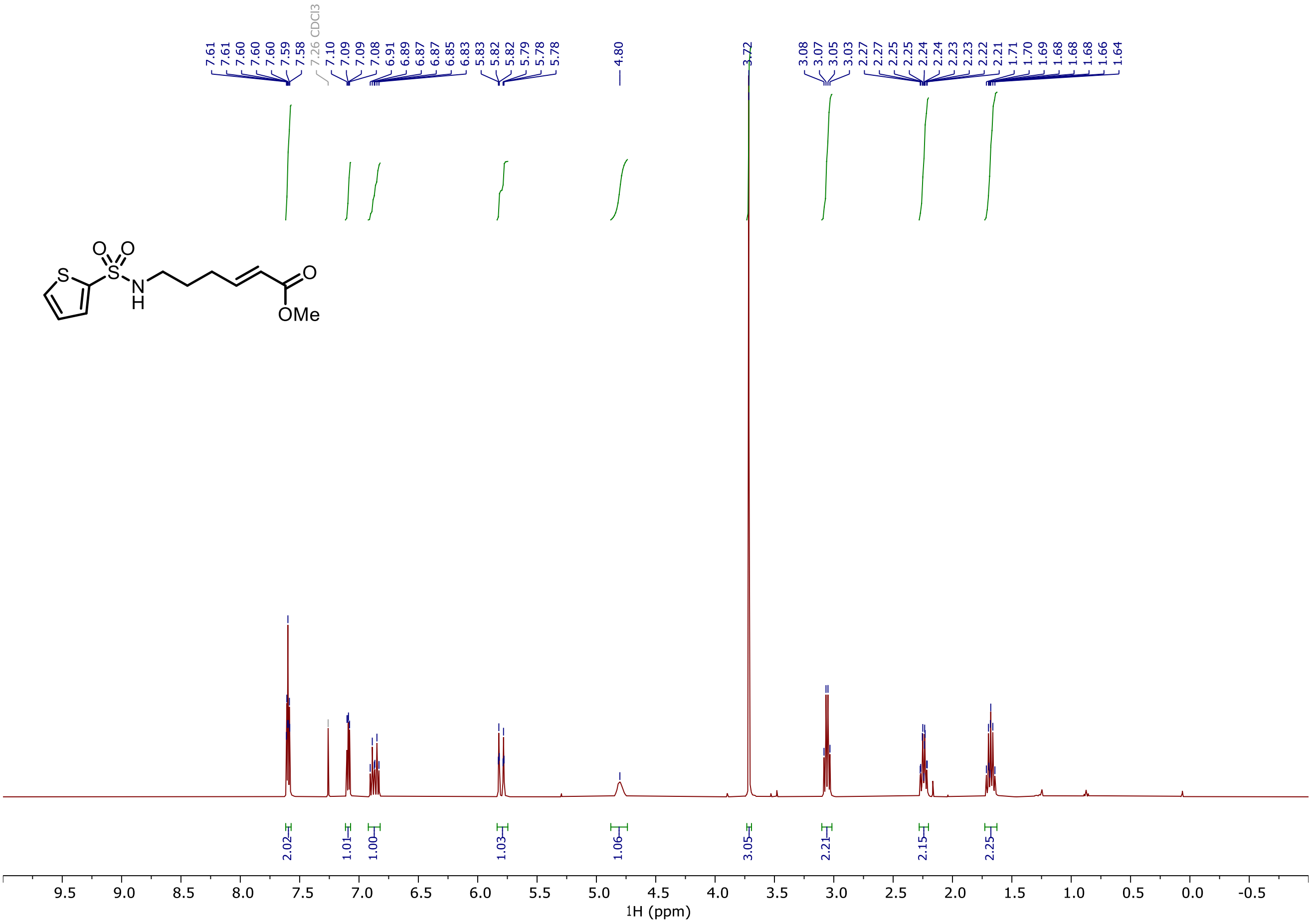

¹³C NMR (101 MHz, $CDCl_3$) of compound **7i**

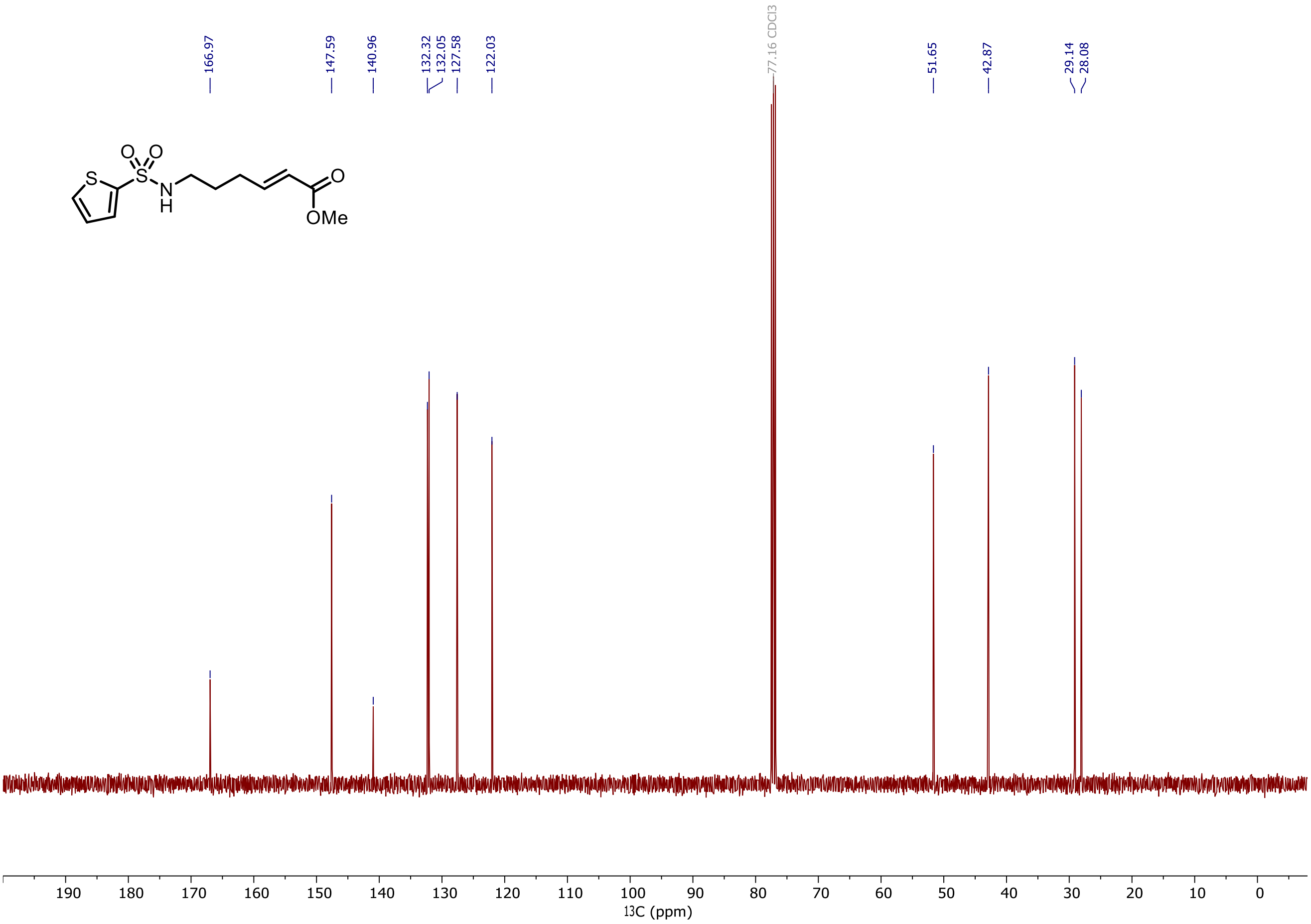

[back to table of contents]

¹H NMR (400 MHz, $CDCl_3$) of compound **S11**

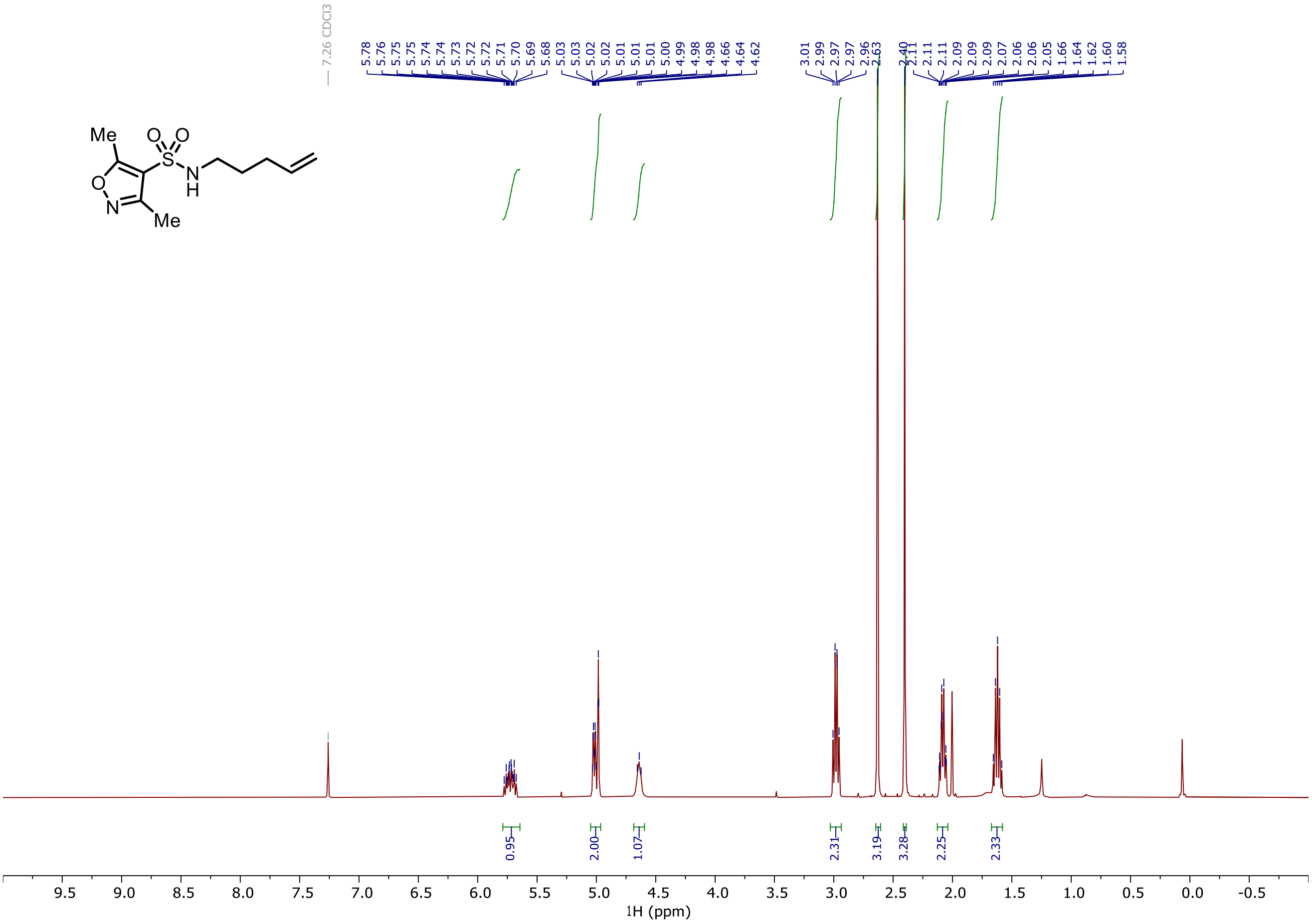

¹³C NMR (101 MHz, $CDCl_3$) of compound **S11**

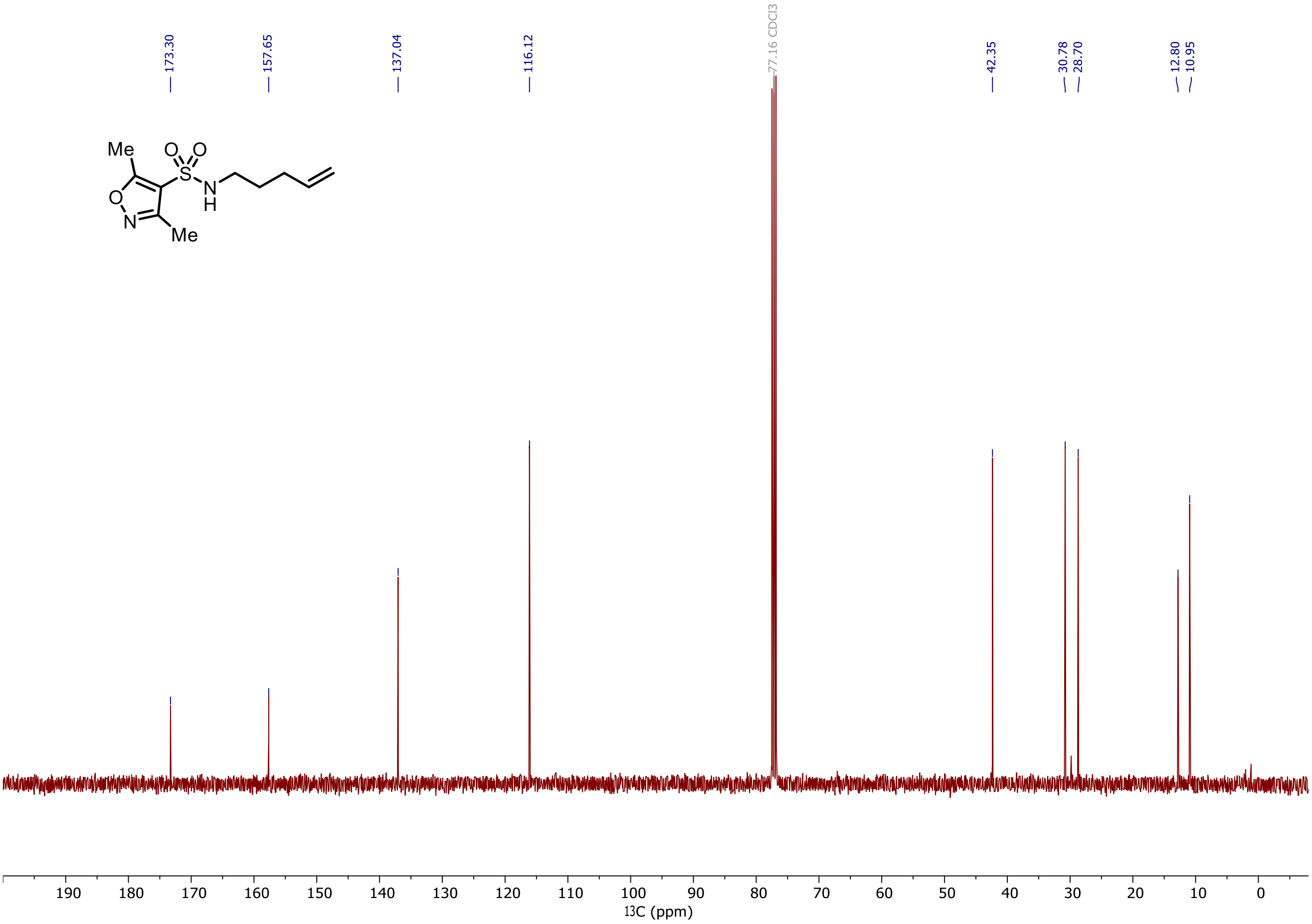

[back to table of contents]

^{1}H NMR (400 MHz, $CDCl_3$) of compound **7j**

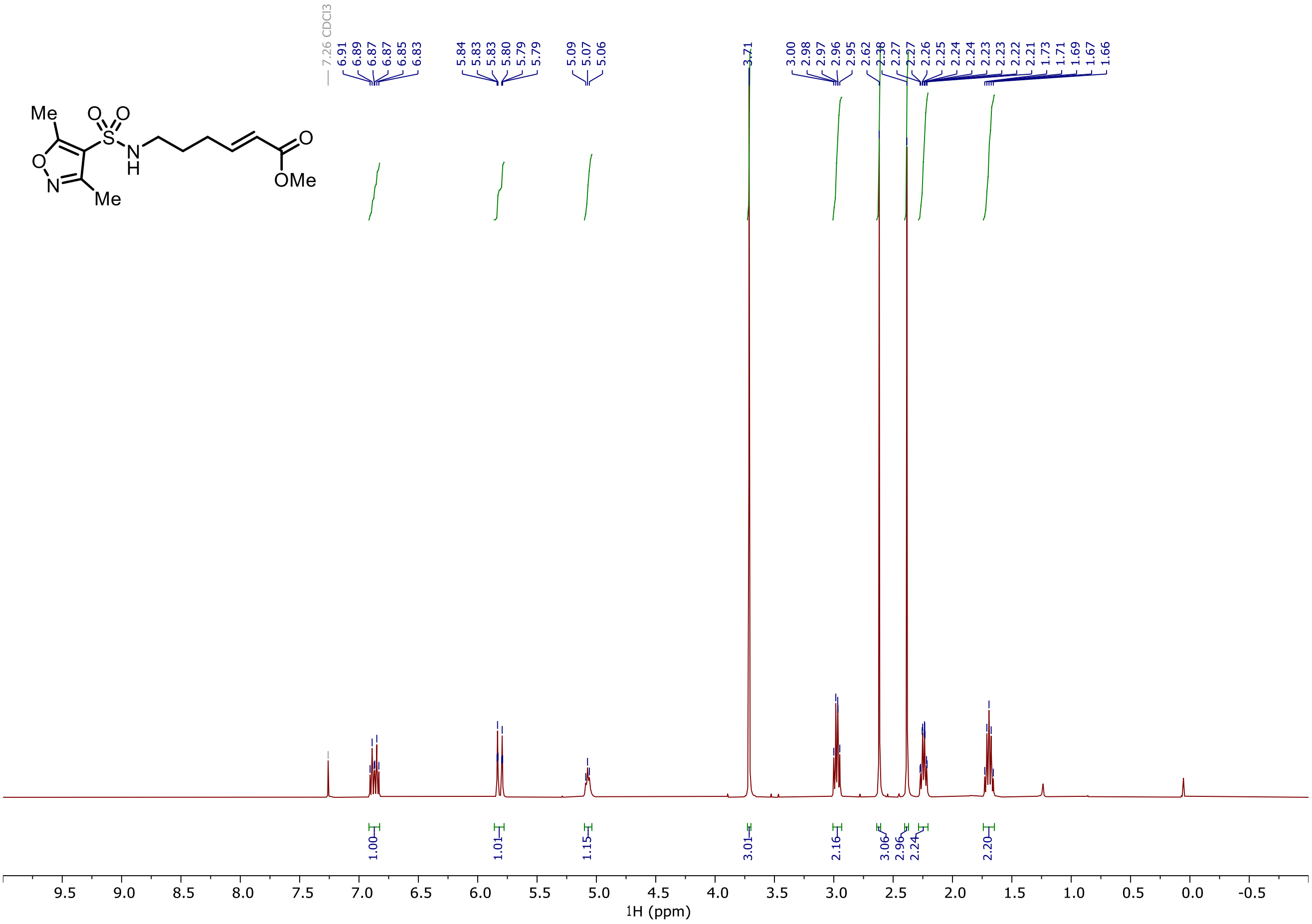

^{13}C NMR (101 MHz, $CDCl_3$) of compound **7j**

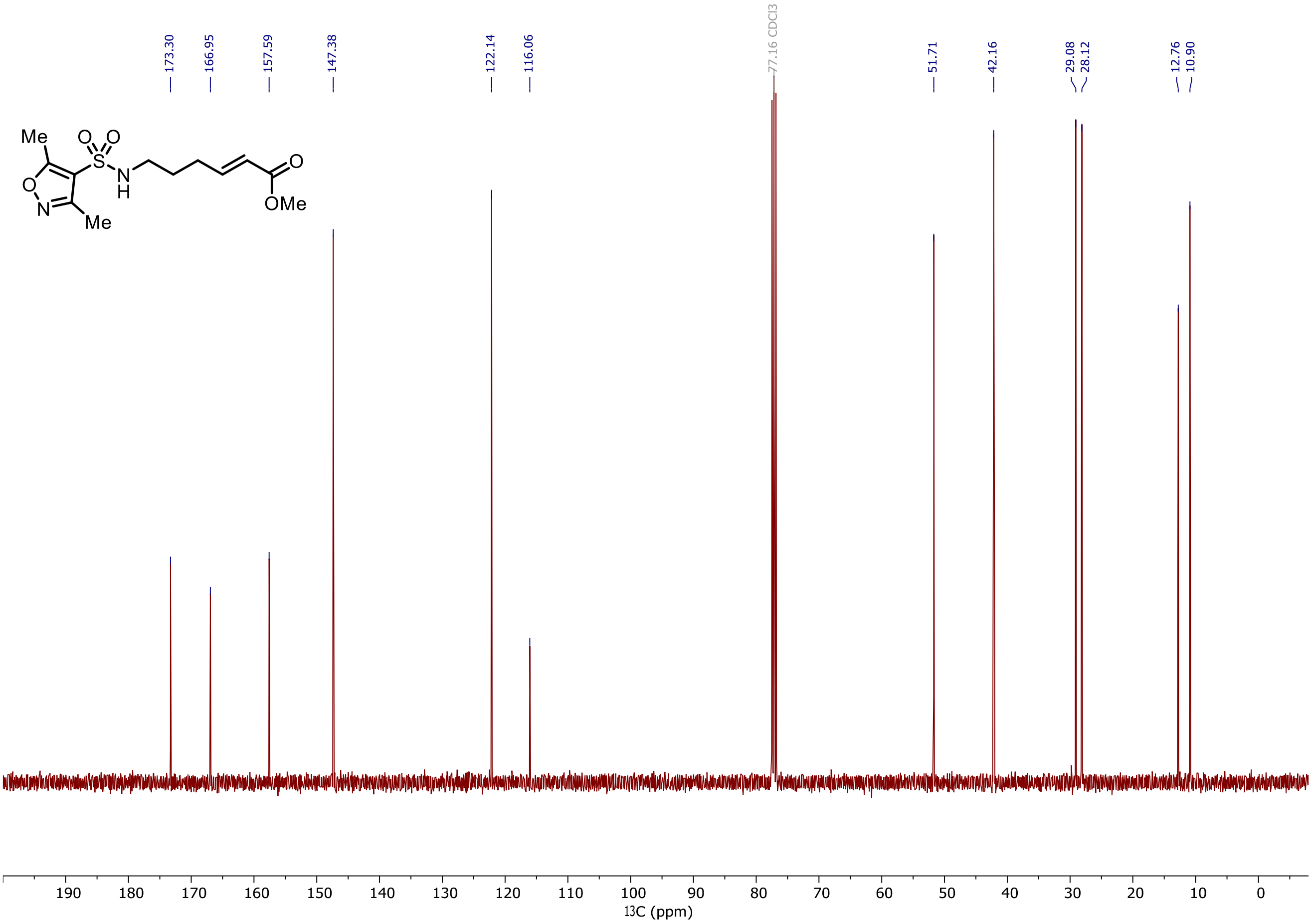

[back to table of contents]

^{1}H NMR (400 MHz, $CDCl_3$) of compound **S12**

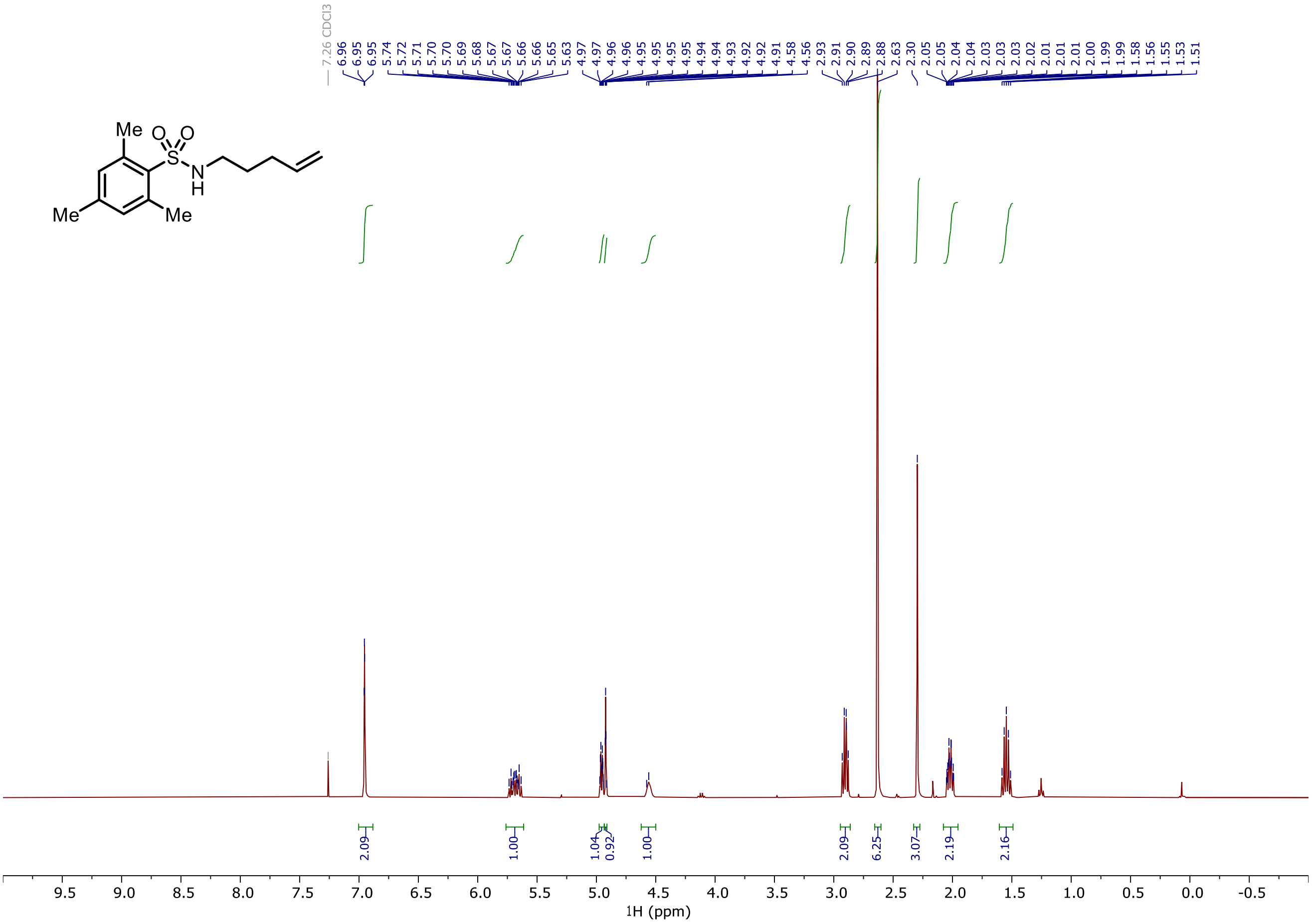

^{13}C NMR (101 MHz, $CDCl_3$) of compound **S12**

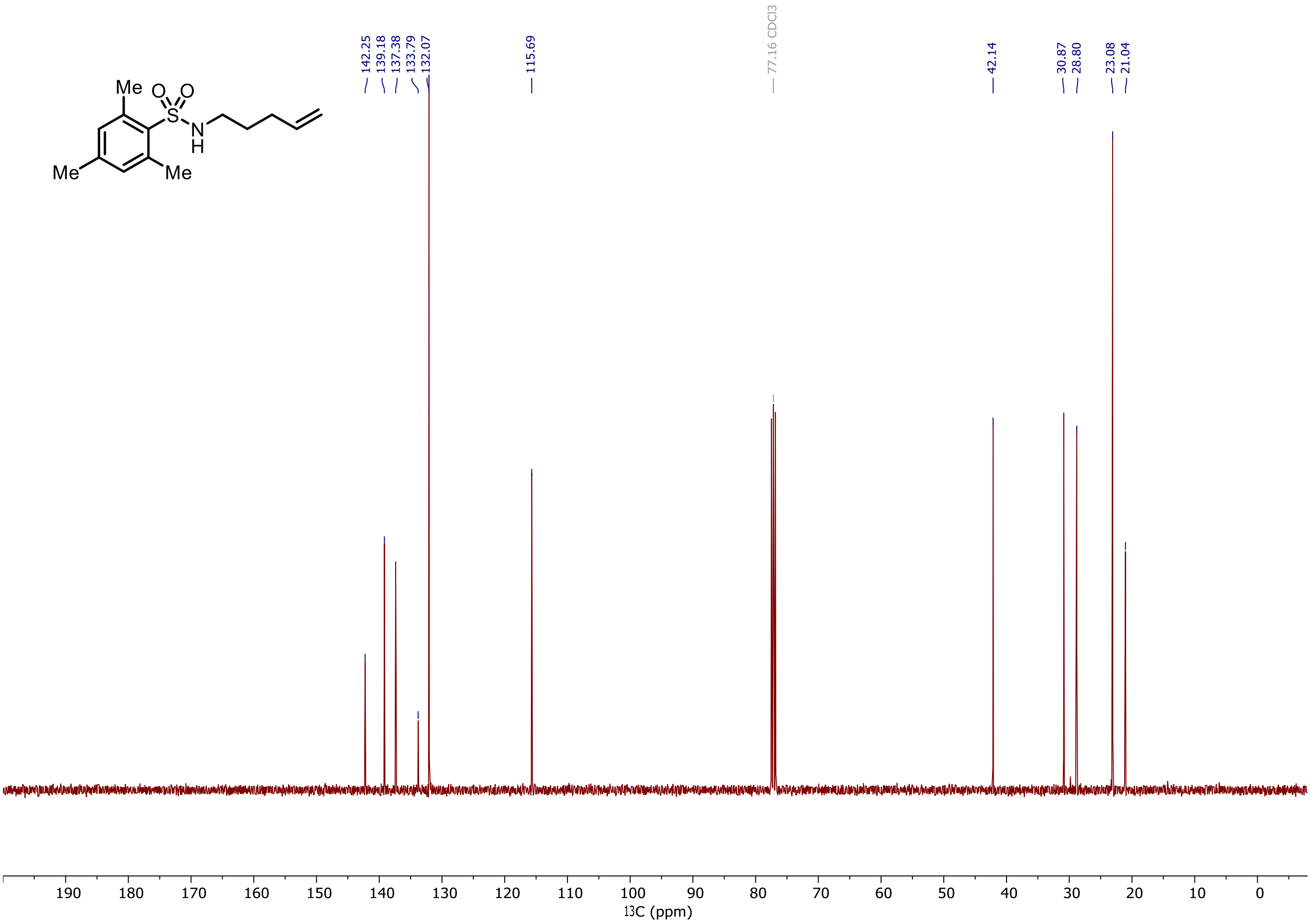

[back to table of contents]

^{1}H NMR (400 MHz, $CDCl_3$) of compound **7k**

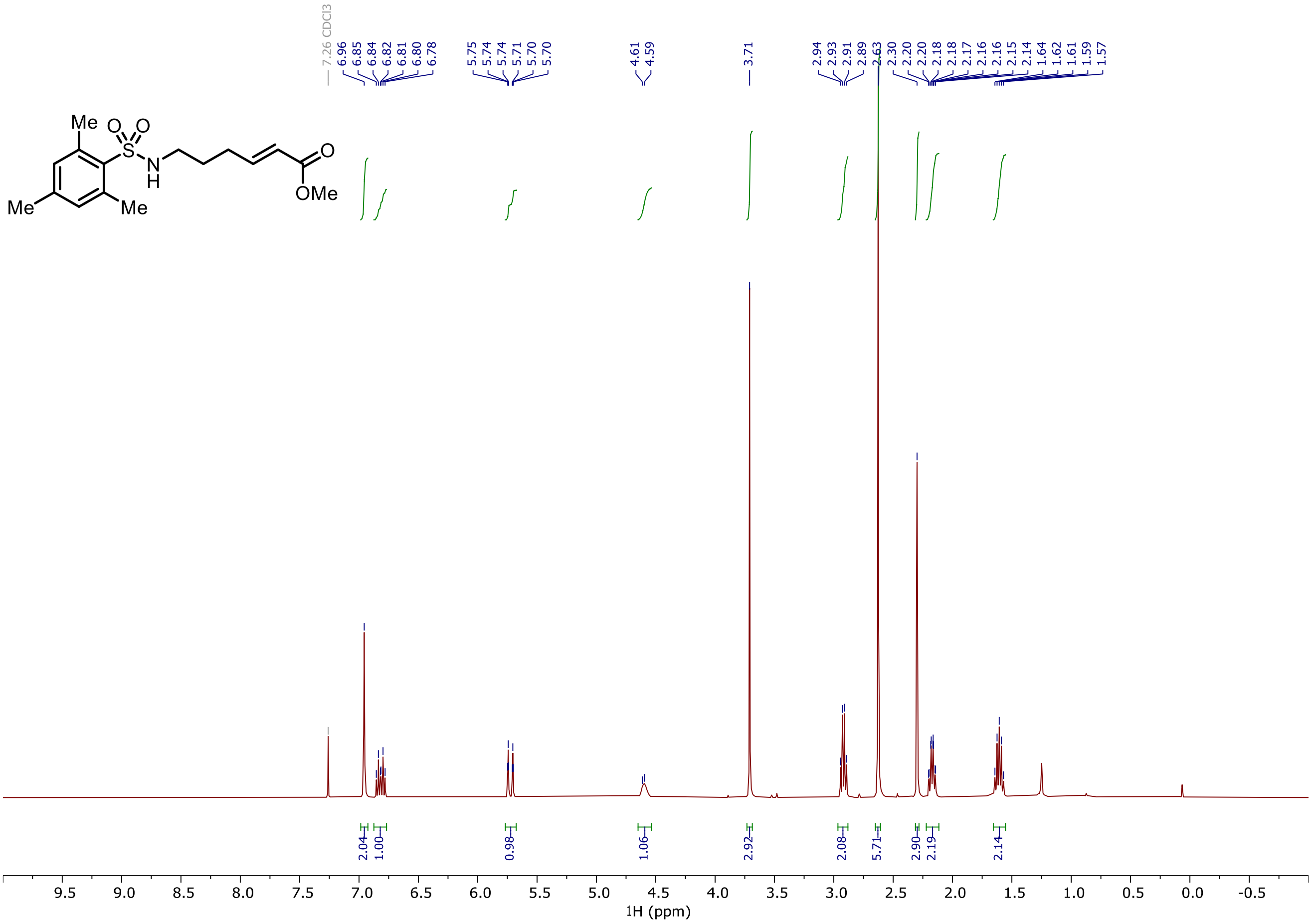

^{13}C NMR (101 MHz, $CDCl_3$) of compound **7k**

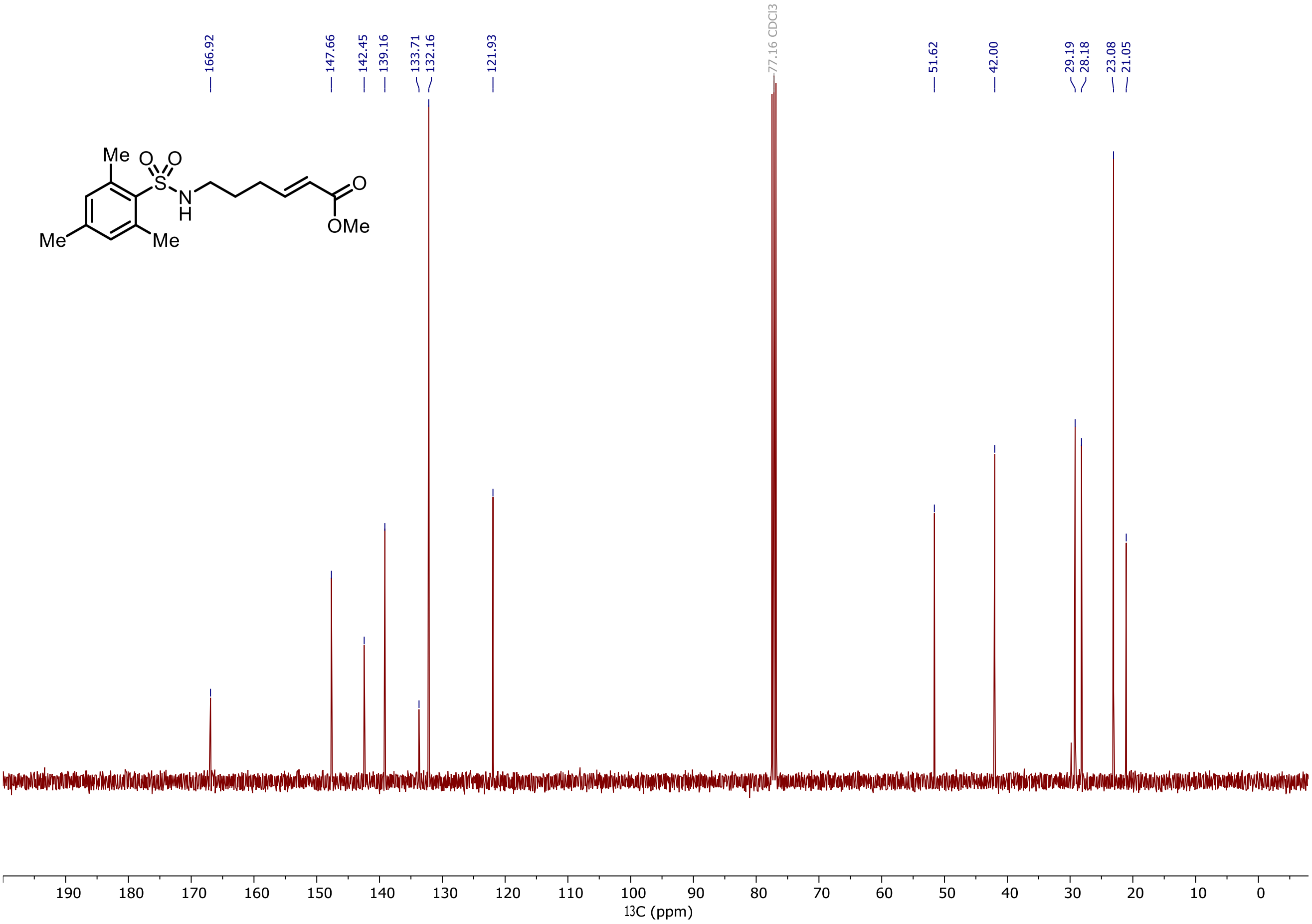

[back to table of contents]

¹H NMR (400 MHz, $CDCl_3$) of compound **S13**

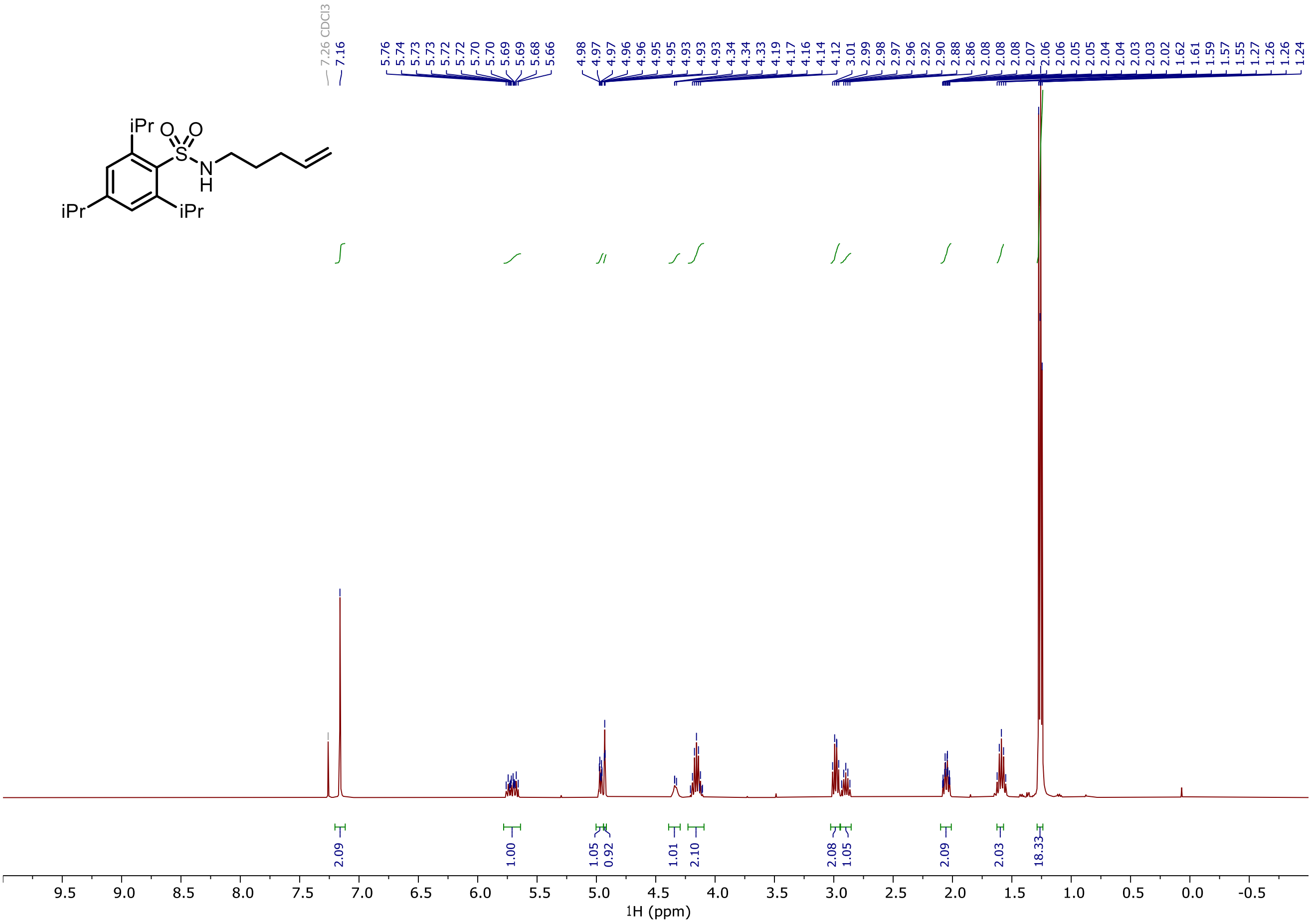

¹³C NMR (101 MHz, $CDCl_3$) of compound **S13**

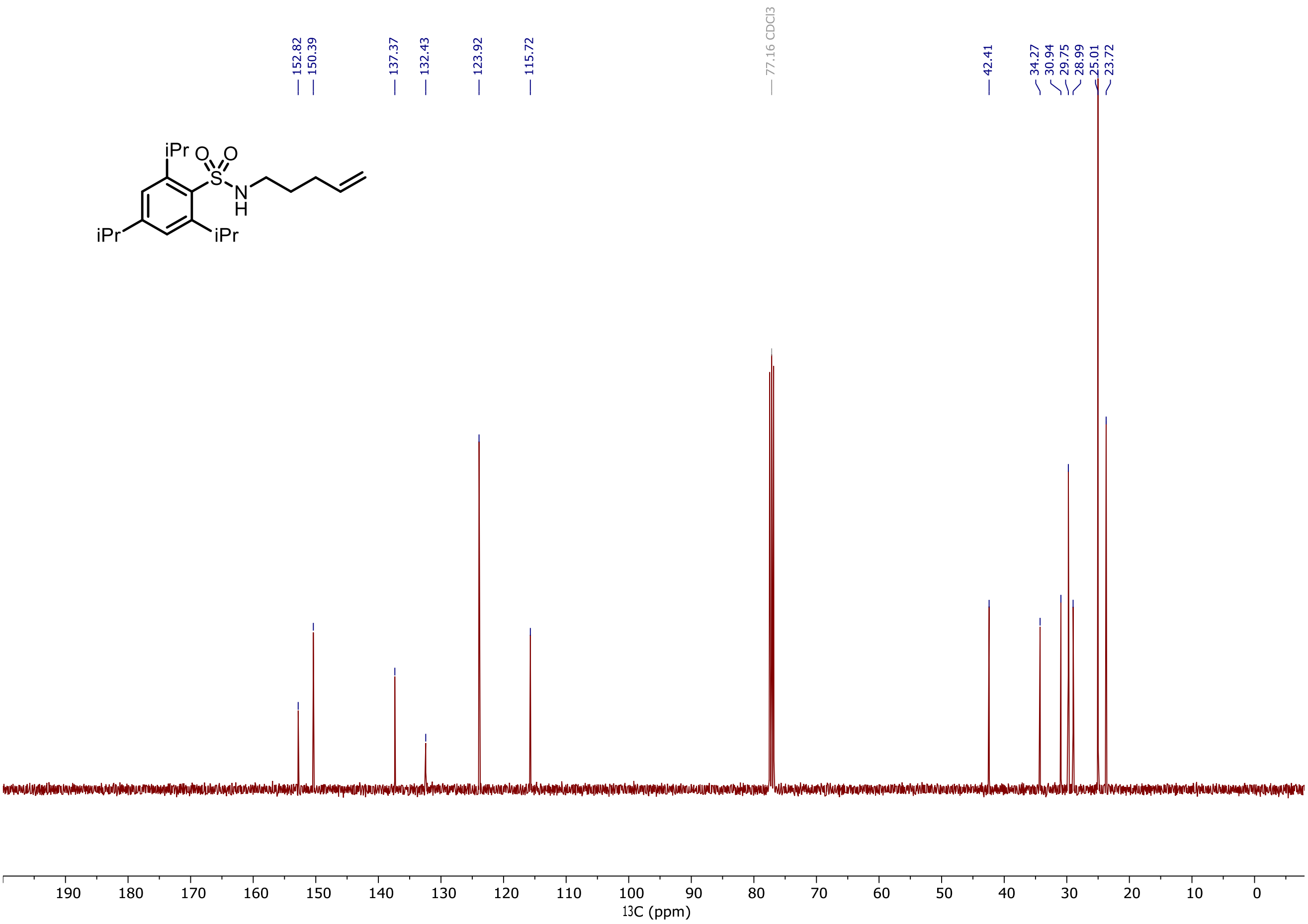

[back to table of contents]

^{1}H NMR (400 MHz, $CDCl_3$) of compound **71**

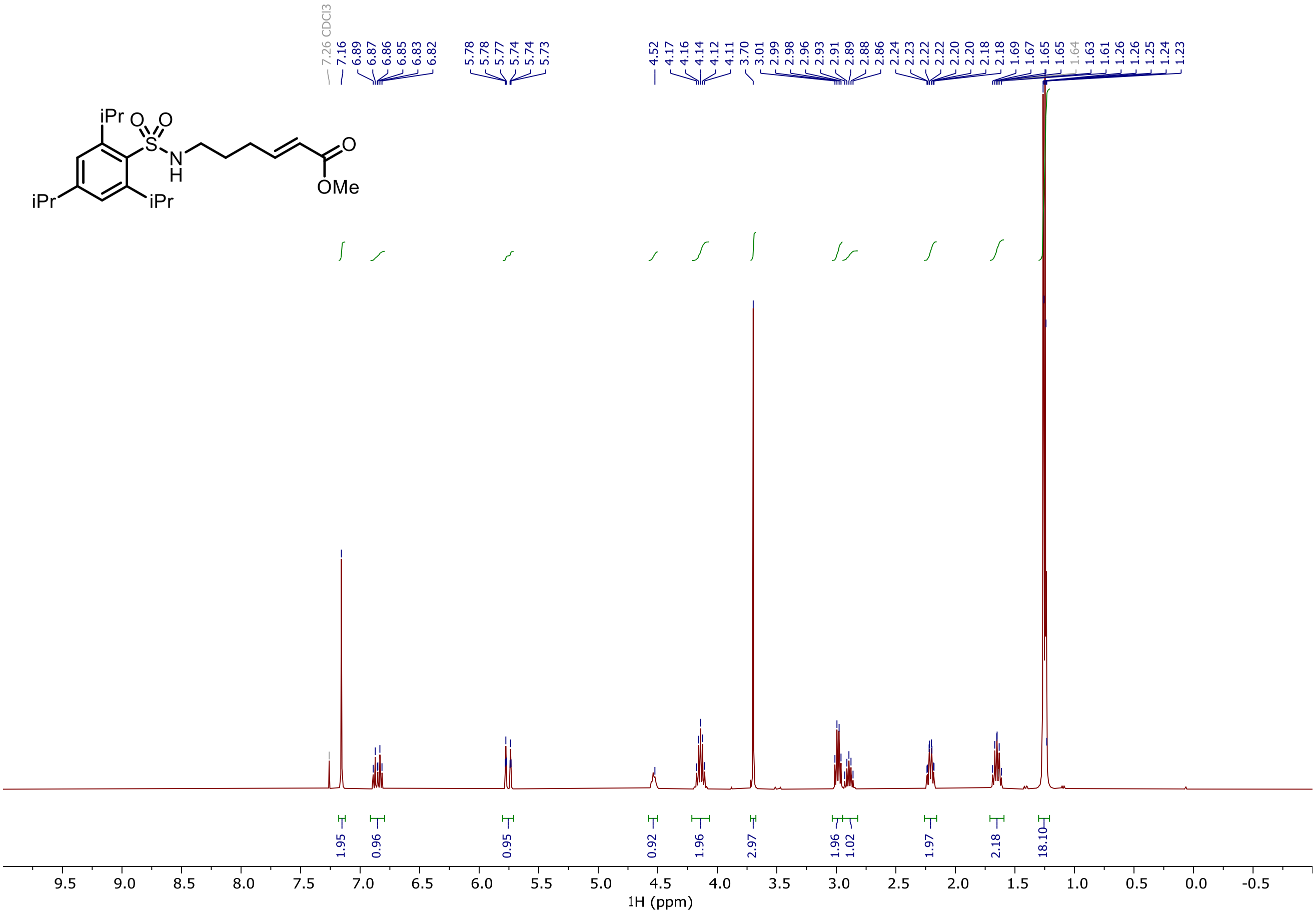

^{13}C NMR (101 MHz, $CDCl_3$) of compound **71**

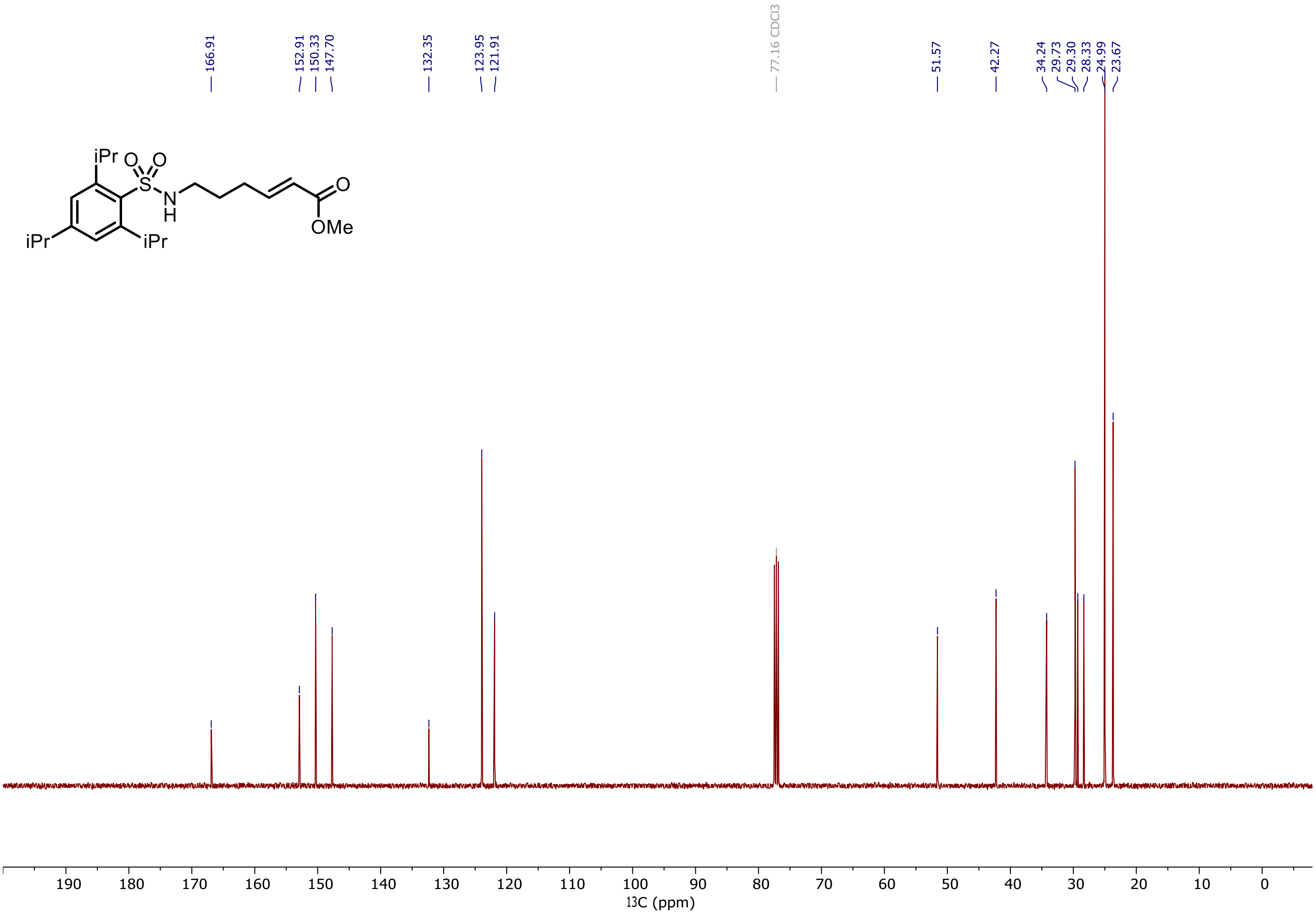

[back to table of contents]

^{1}H NMR (400 MHz, $CDCl_3$) of compound **S14**

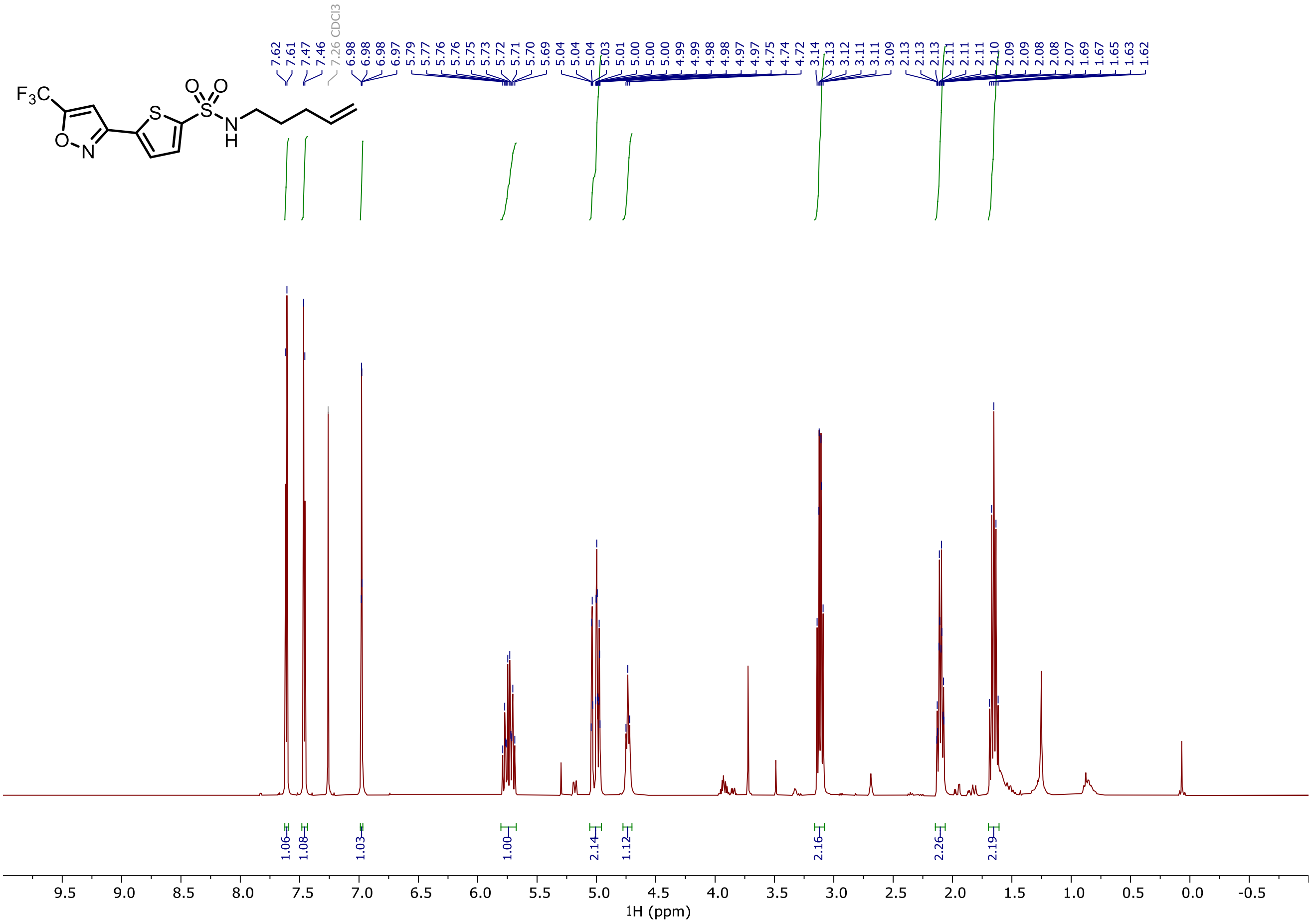

^{13}C NMR (101 MHz, $CDCl_3$) of compound **S14**

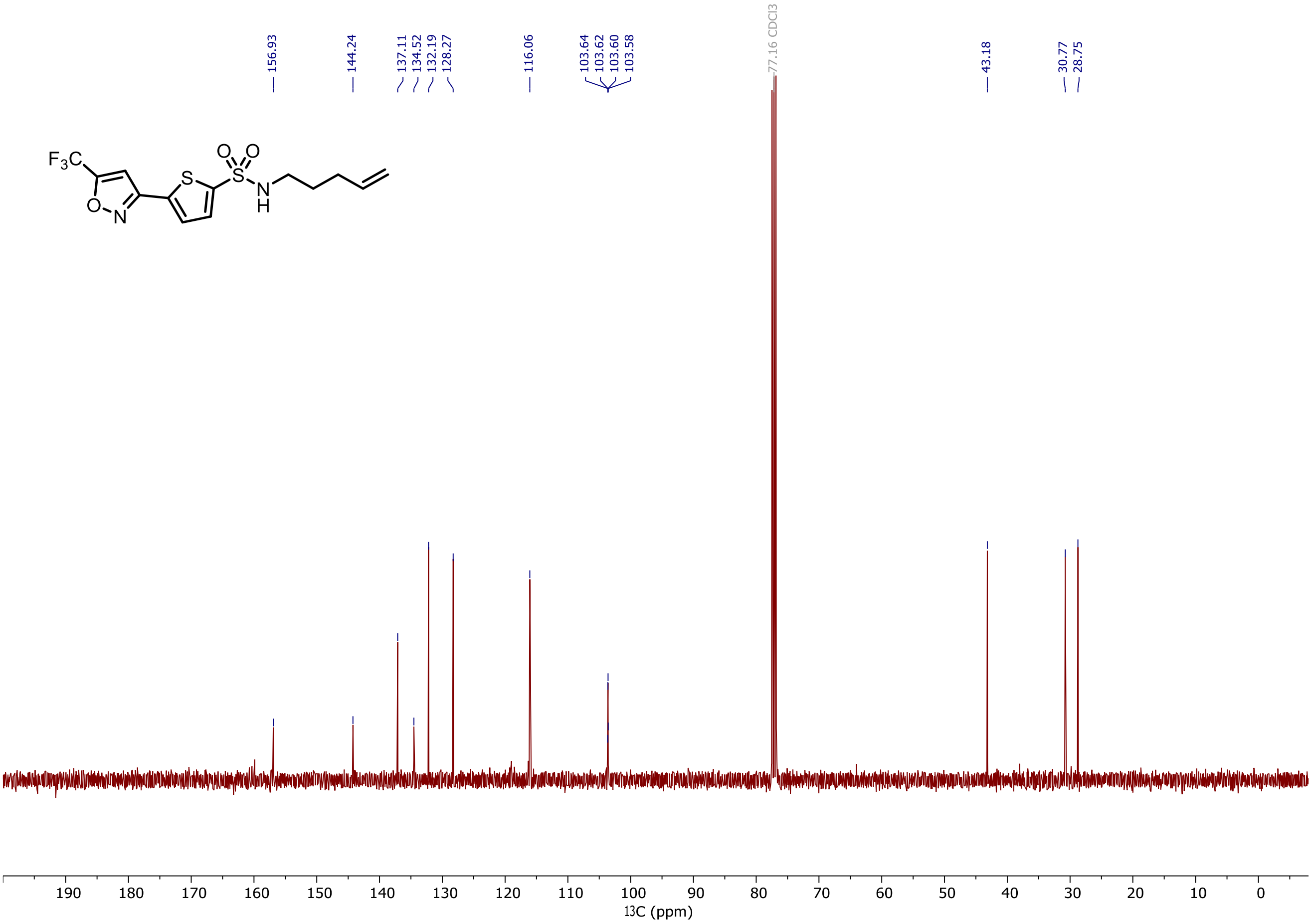

[back to table of contents]

^{19}F NMR (376 MHz, $CDCl_3$) of compound **S14** (contains PhF as internal standard, δ_F -113.15 ppm)

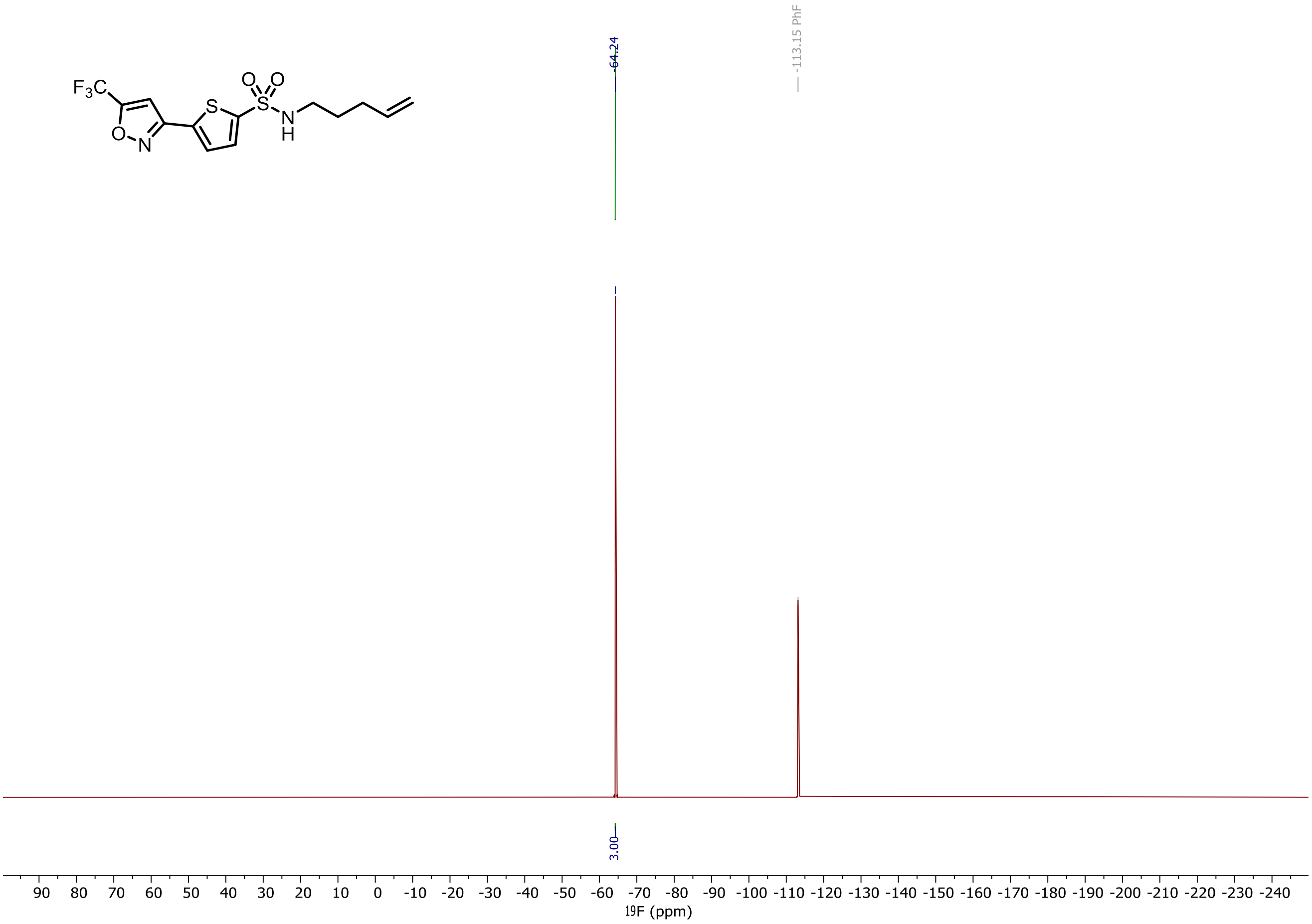

[back to table of contents]

^{1}H NMR (400 MHz, $CDCl_3$) of compound **7m**

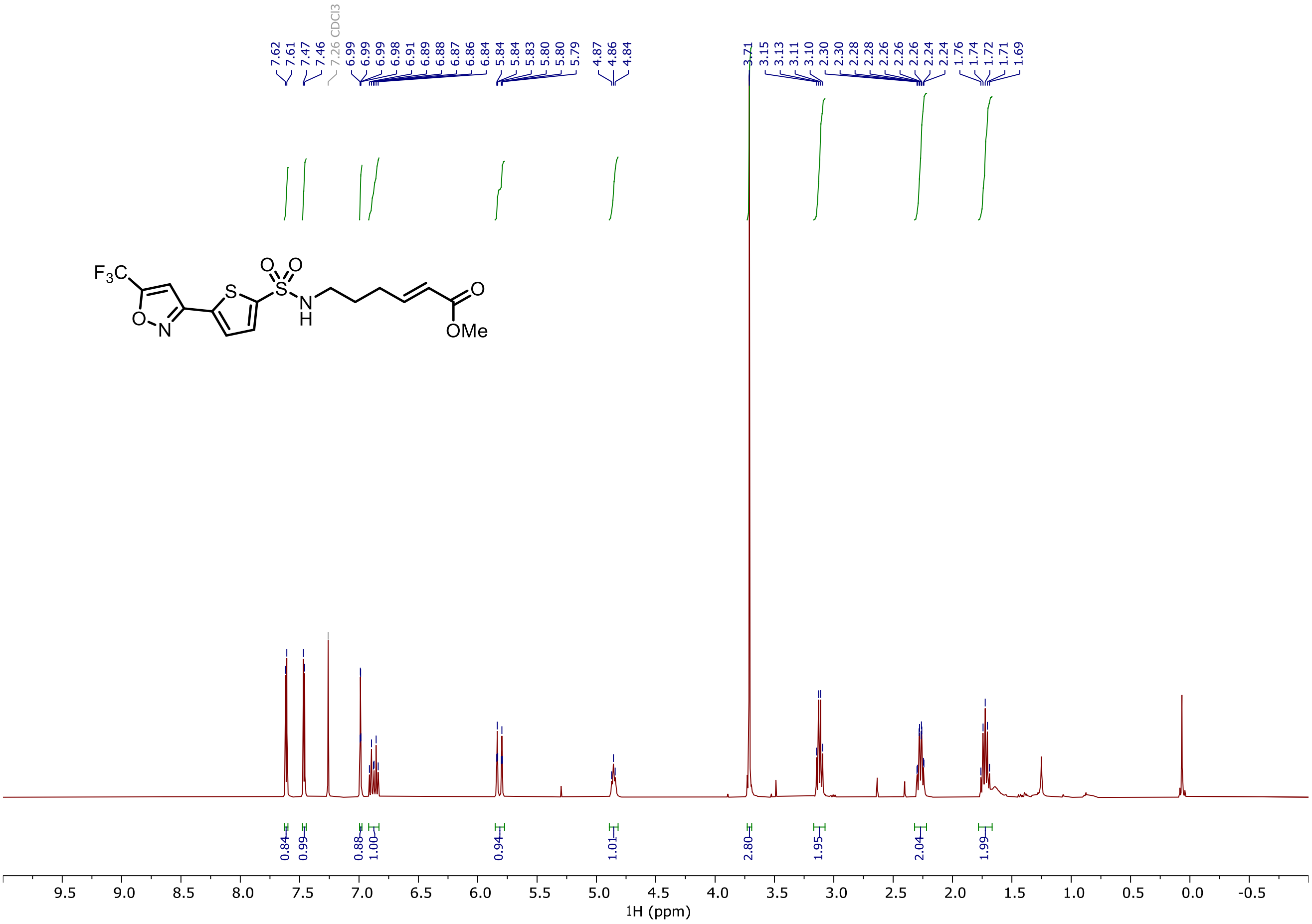

^{13}C NMR (101 MHz, $CDCl_3$) of compound **7m**

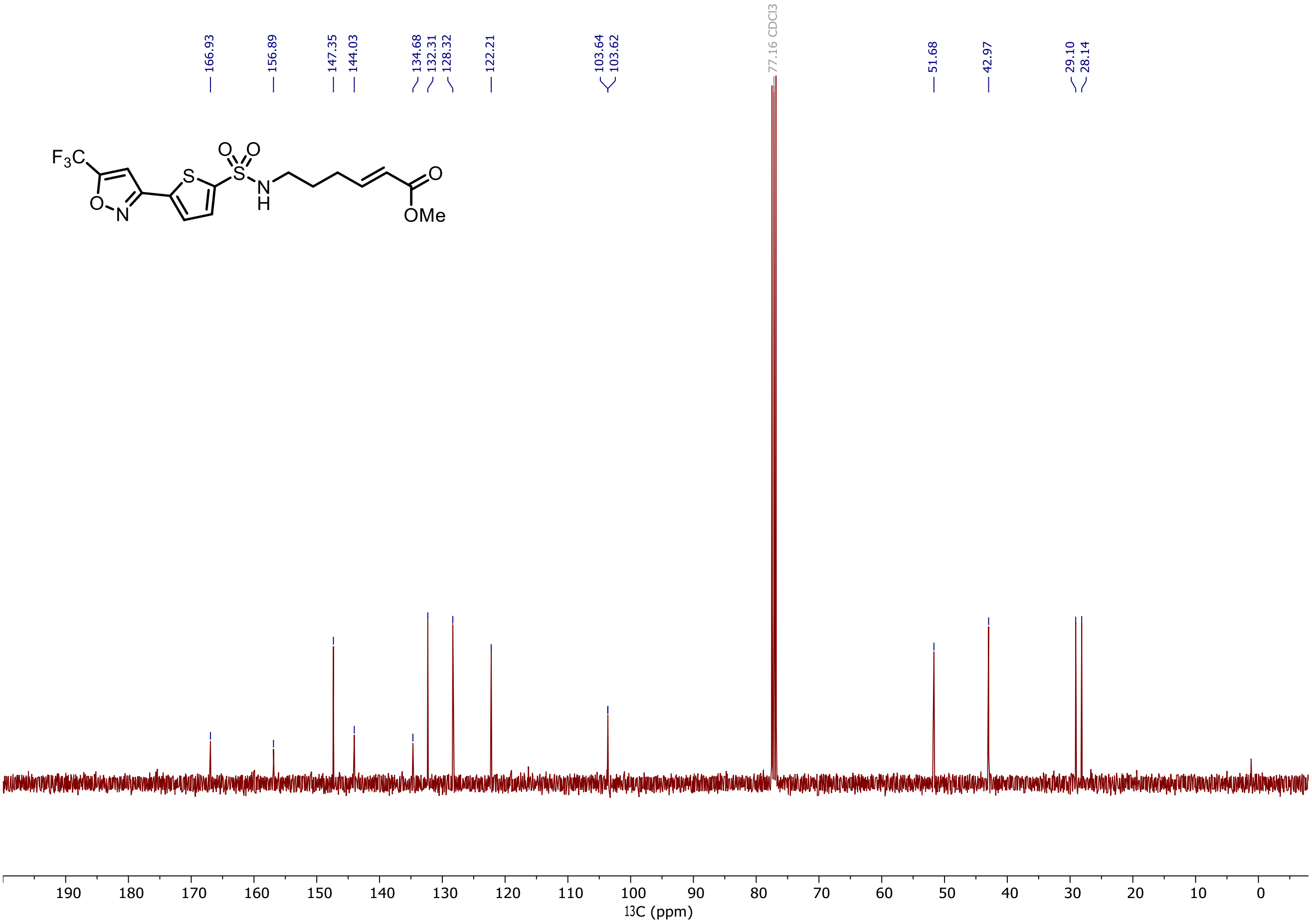

[back to table of contents]

19F NMR (376 MHz, $CDCl_3$) of compound **7m** (contains PhF as internal standard, δ_F -113.15 ppm)

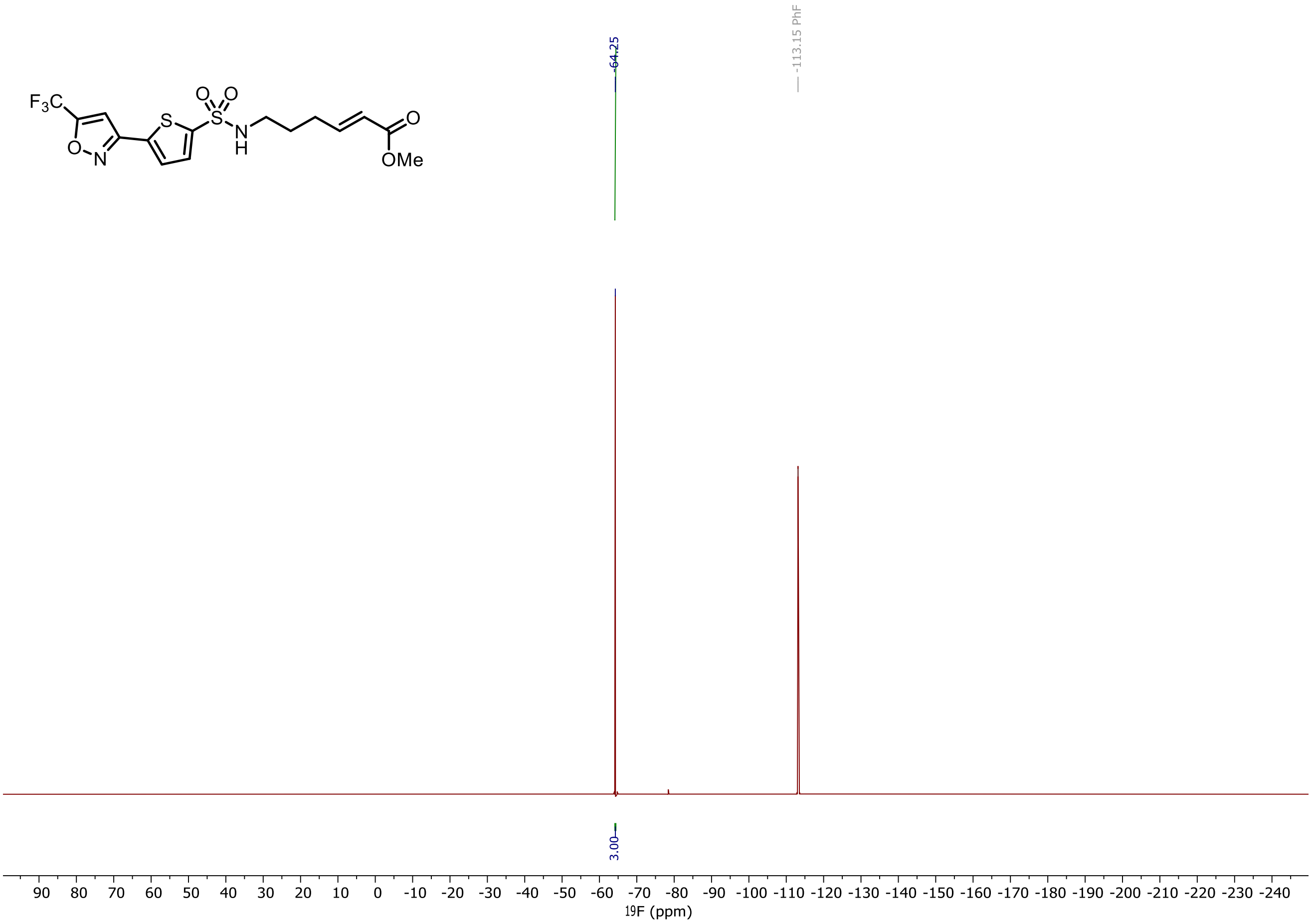

[back to table of contents]

^{1}H NMR (600 MHz, $CDCl_3$) of compound **S16**

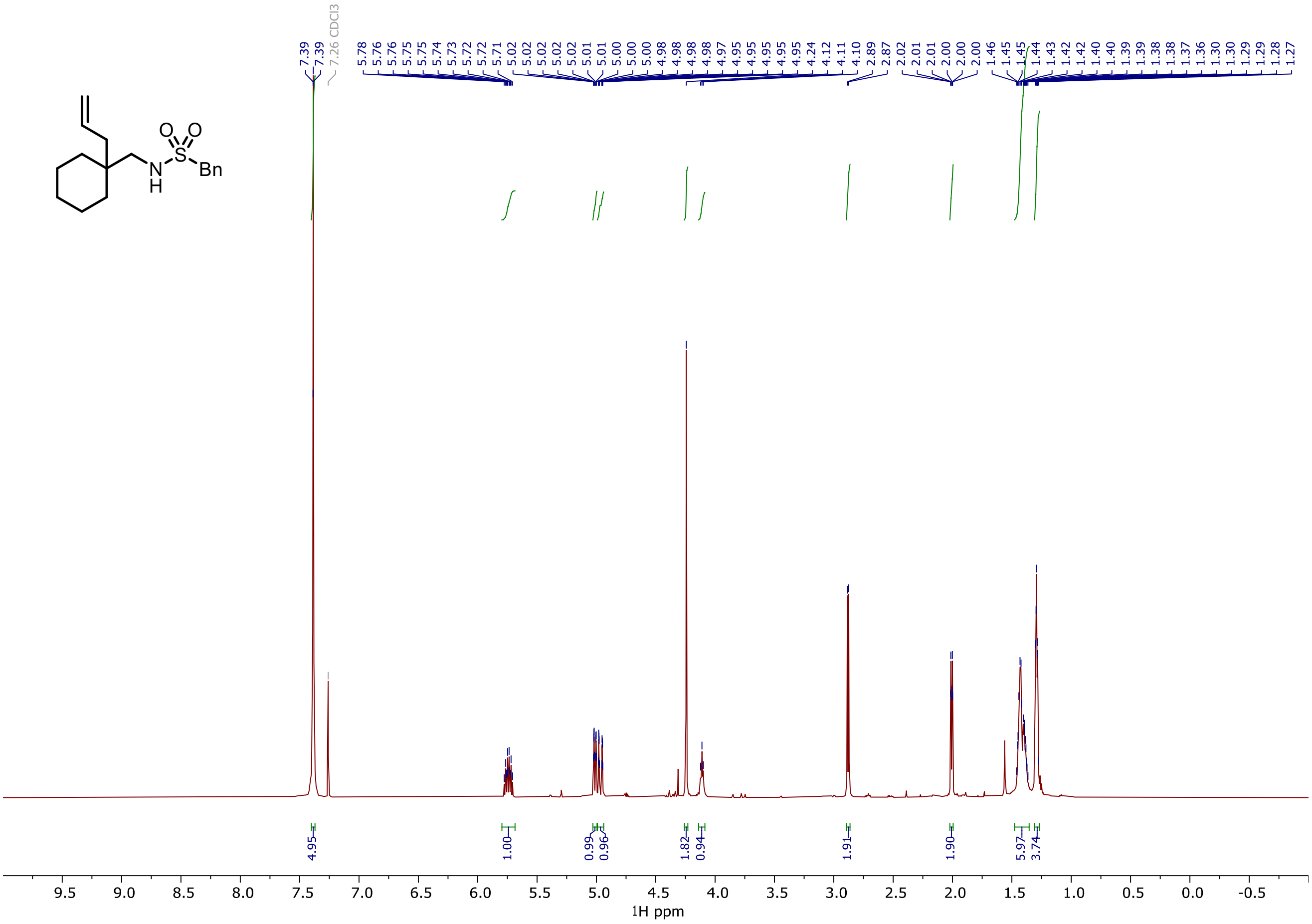

^{13}C NMR (151 MHz, $CDCl_3$) of compound **S16**

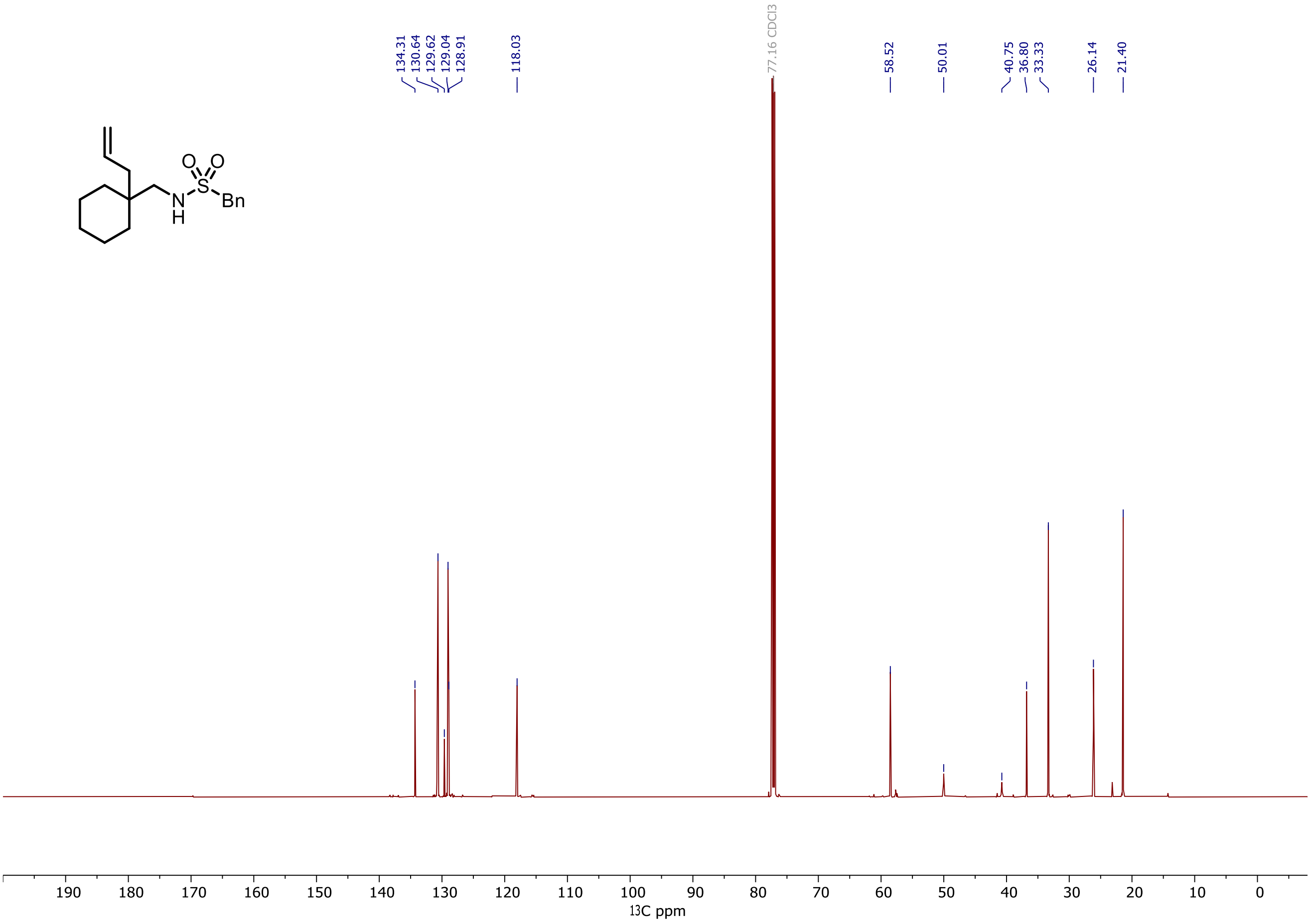

[back to table of contents]

¹H NMR (600 MHz, $CDCl_3$) of compound **7n**

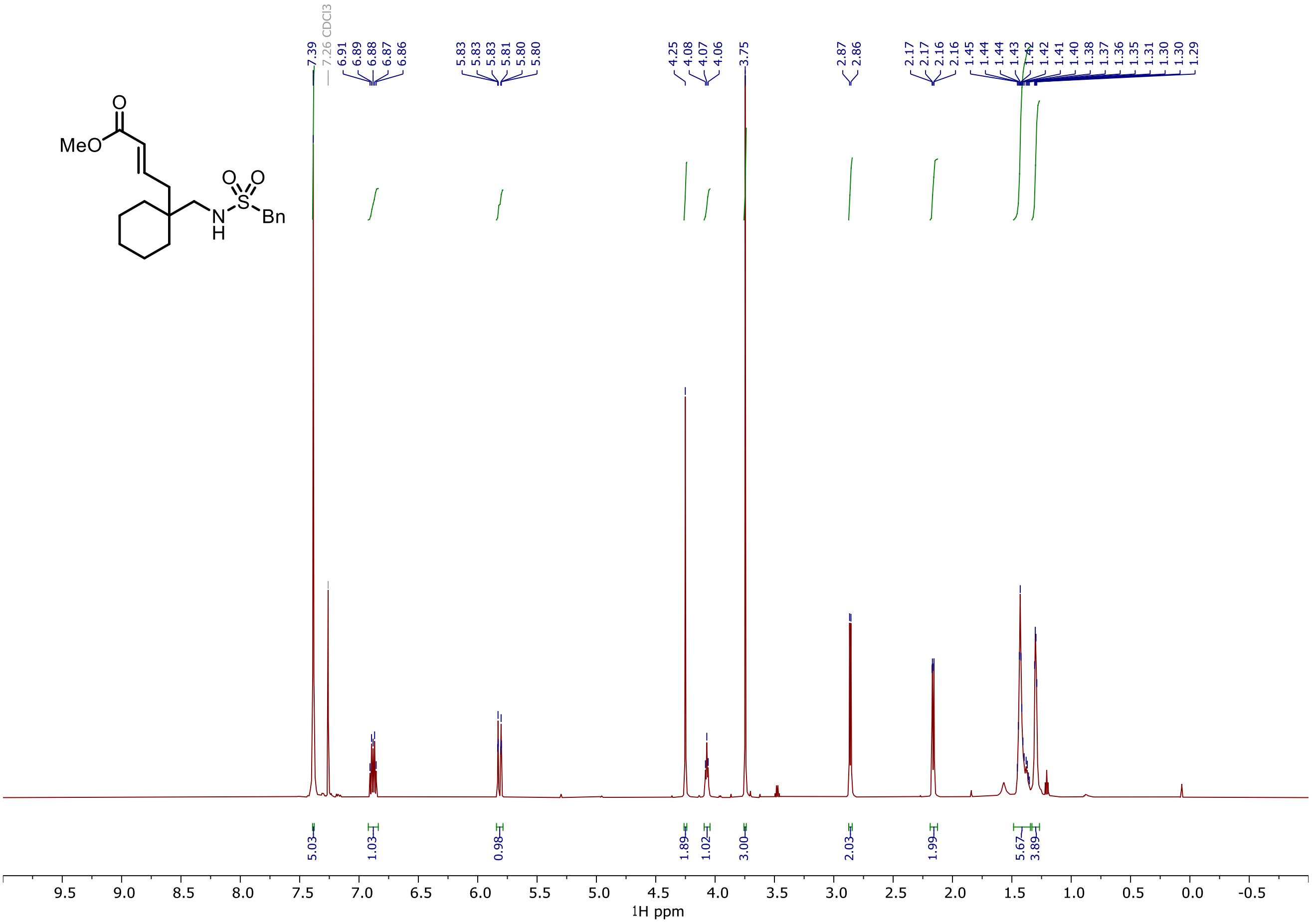

¹³C NMR (151 MHz, $CDCl_3$) of compound **7n**

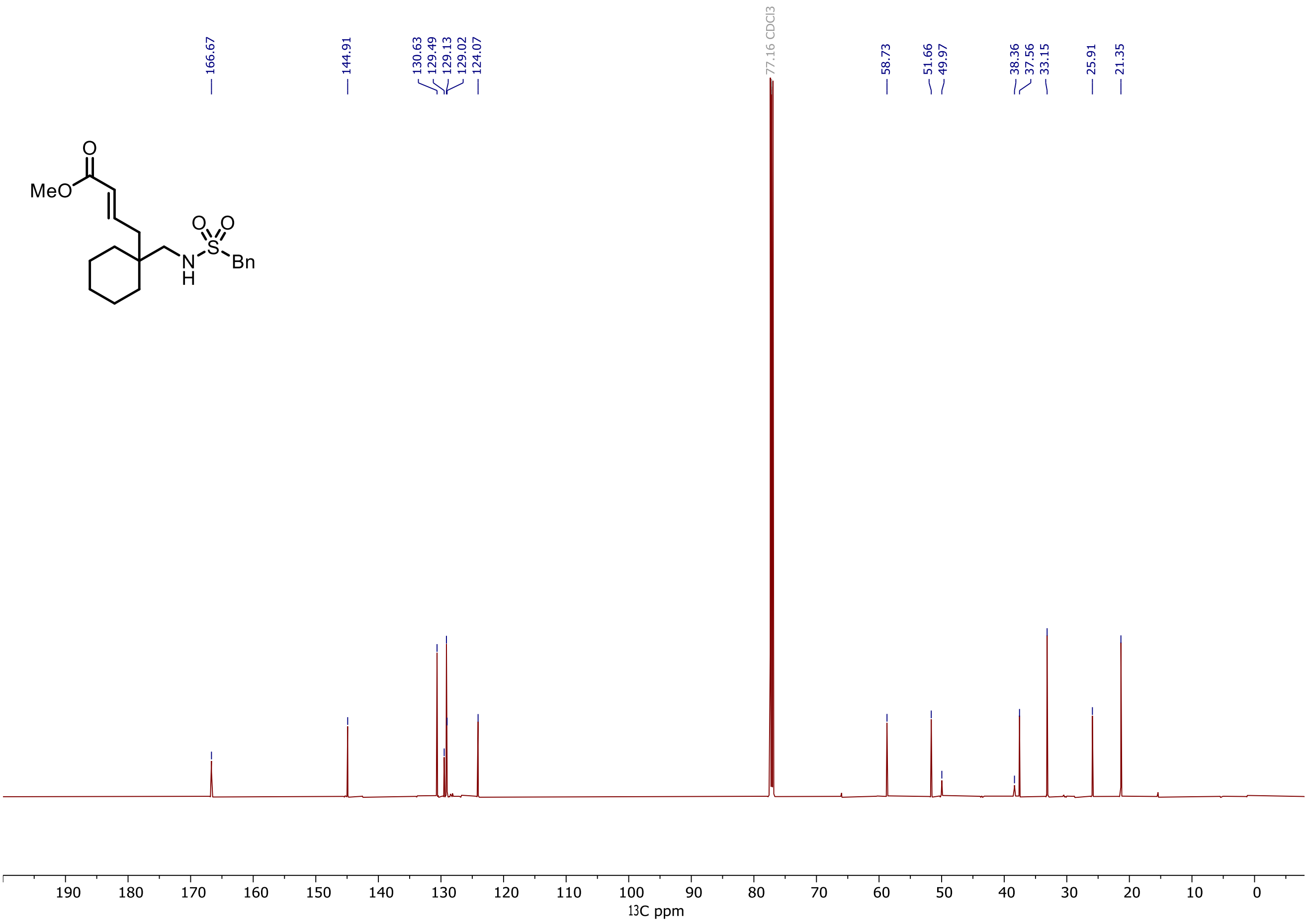

[back to table of contents]

^{1}H NMR (600 MHz, $CDCl_3$) of compound **S17**

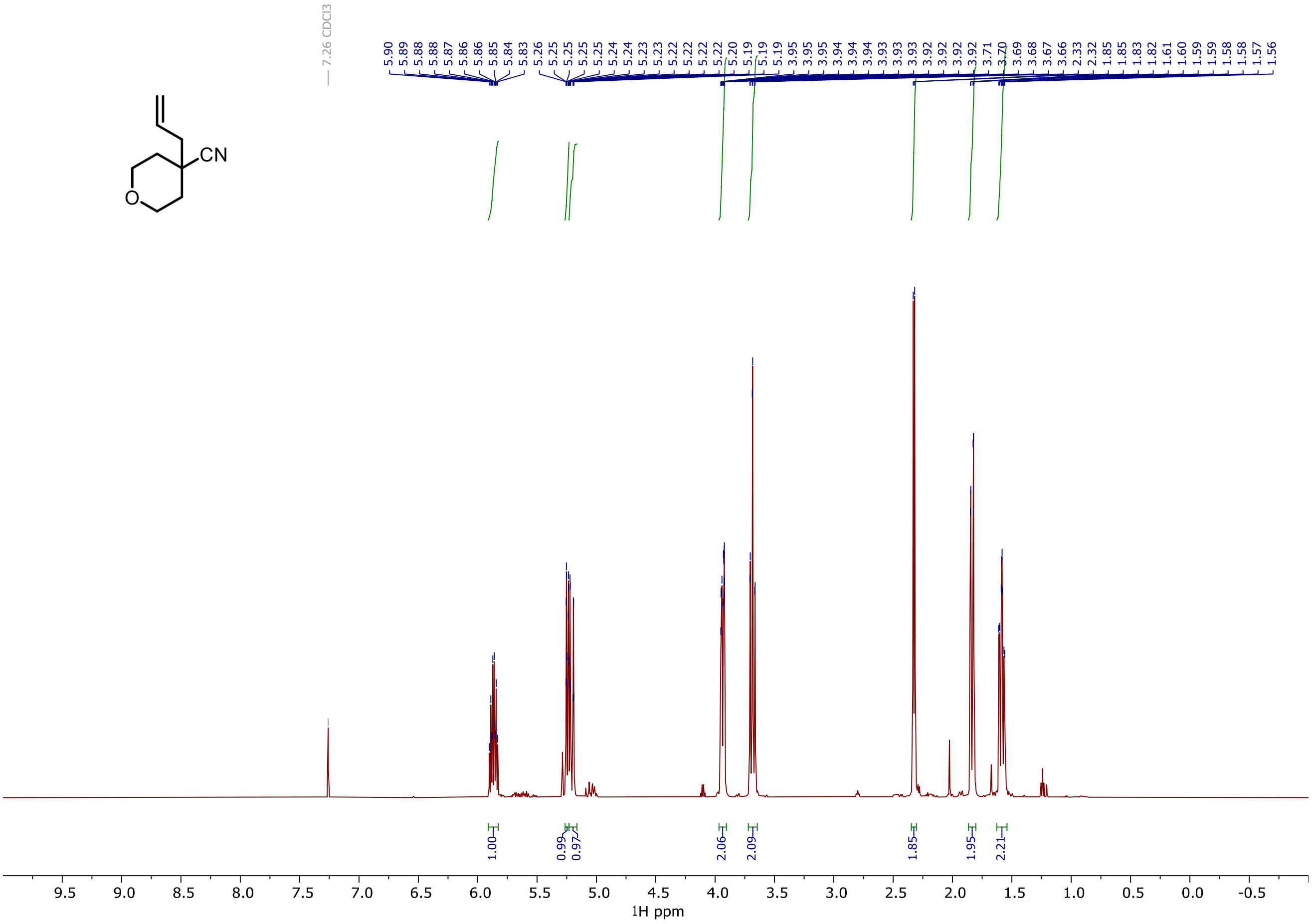

^{13}C NMR (151 MHz, $CDCl_3$) of compound **S17**

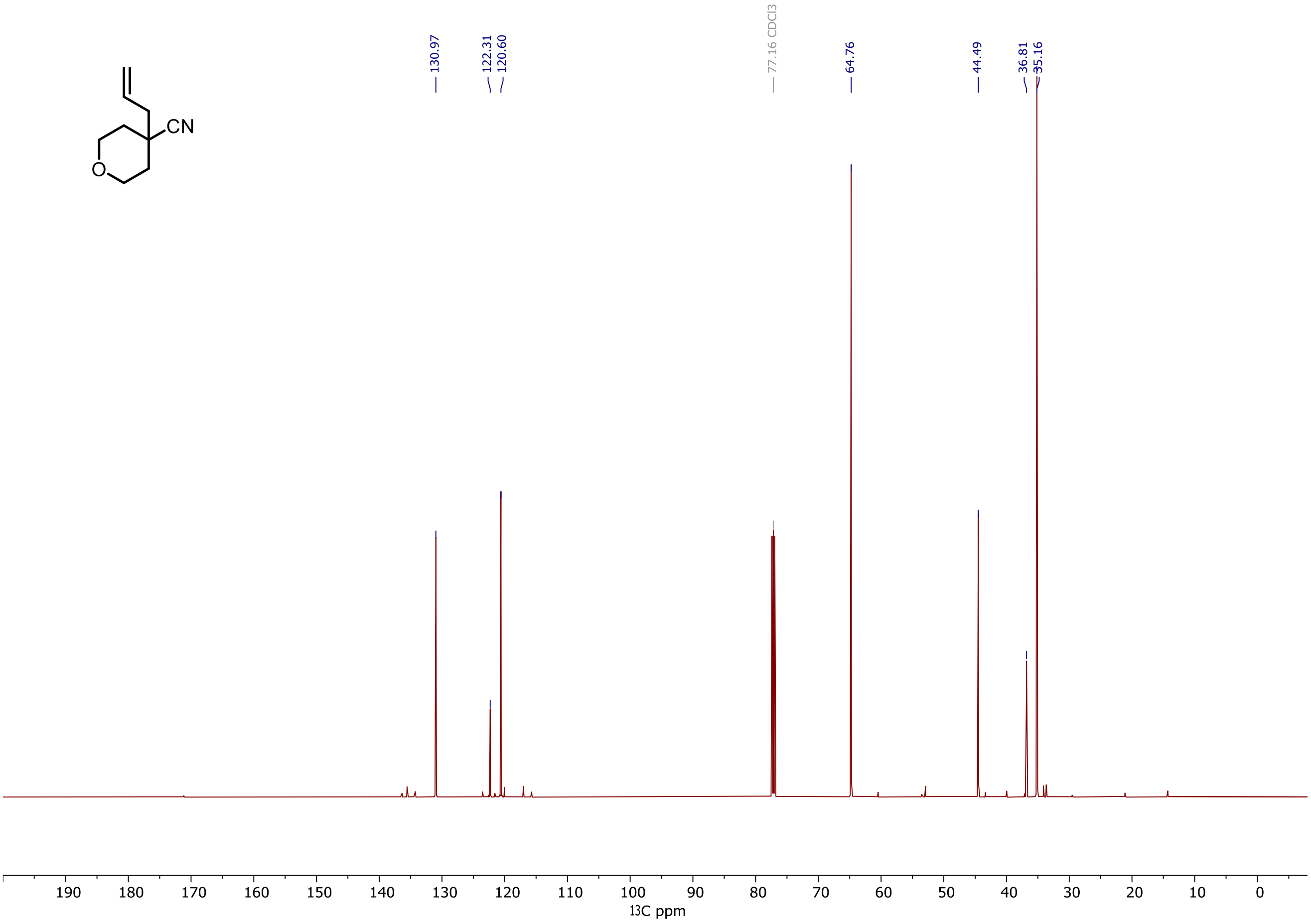

[back to table of contents]

Quantitative ^{1}H NMR (400 MHz, $CDCl_3$) of compound **S17** with 1,3,5-trimethoxybenzene as an internal standard

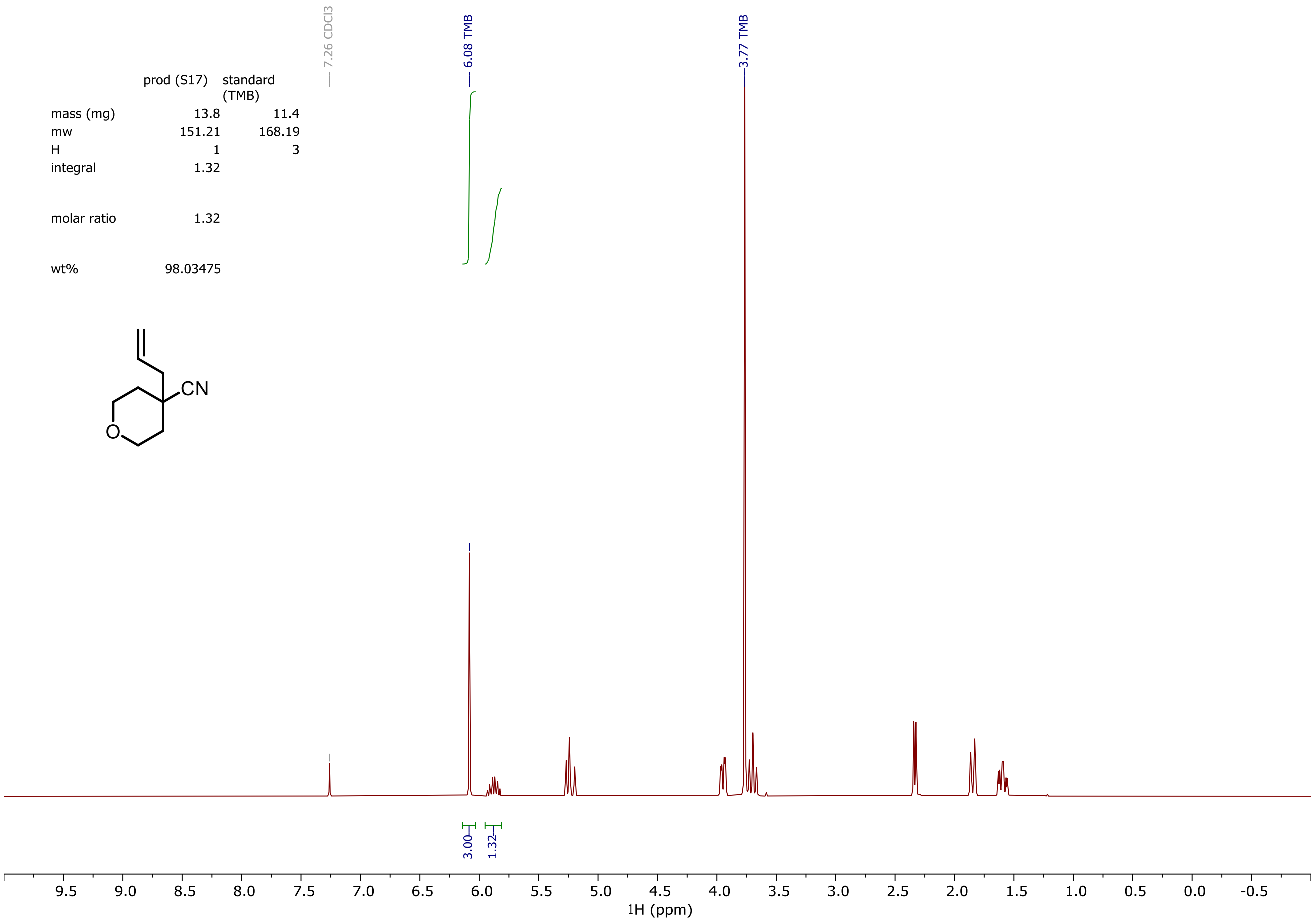

[back to table of contents]

^{1}H NMR (600 MHz, $CDCl_3$) of compound **S18**

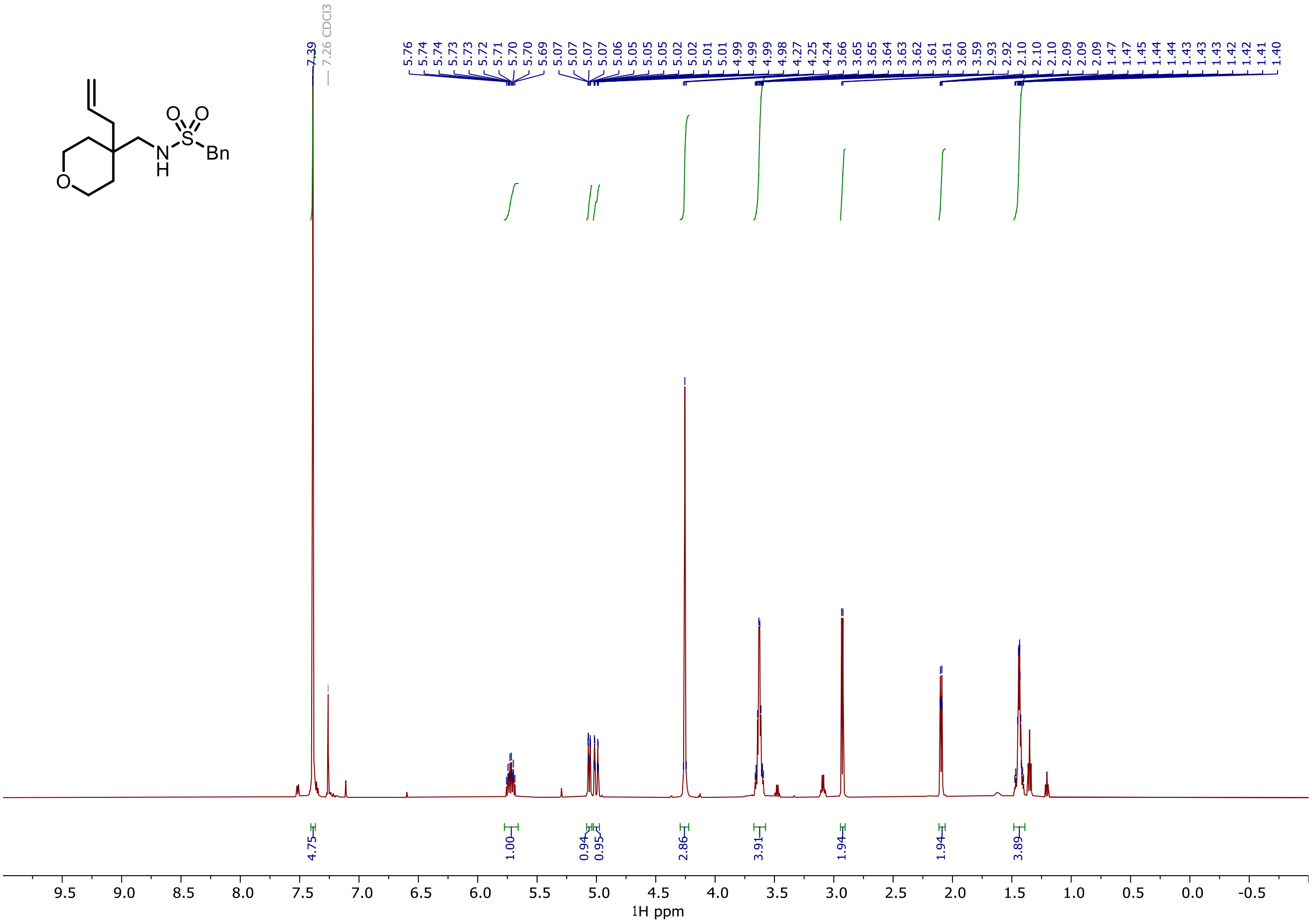

^{13}C NMR (151 MHz, $CDCl_3$) of compound **S18**

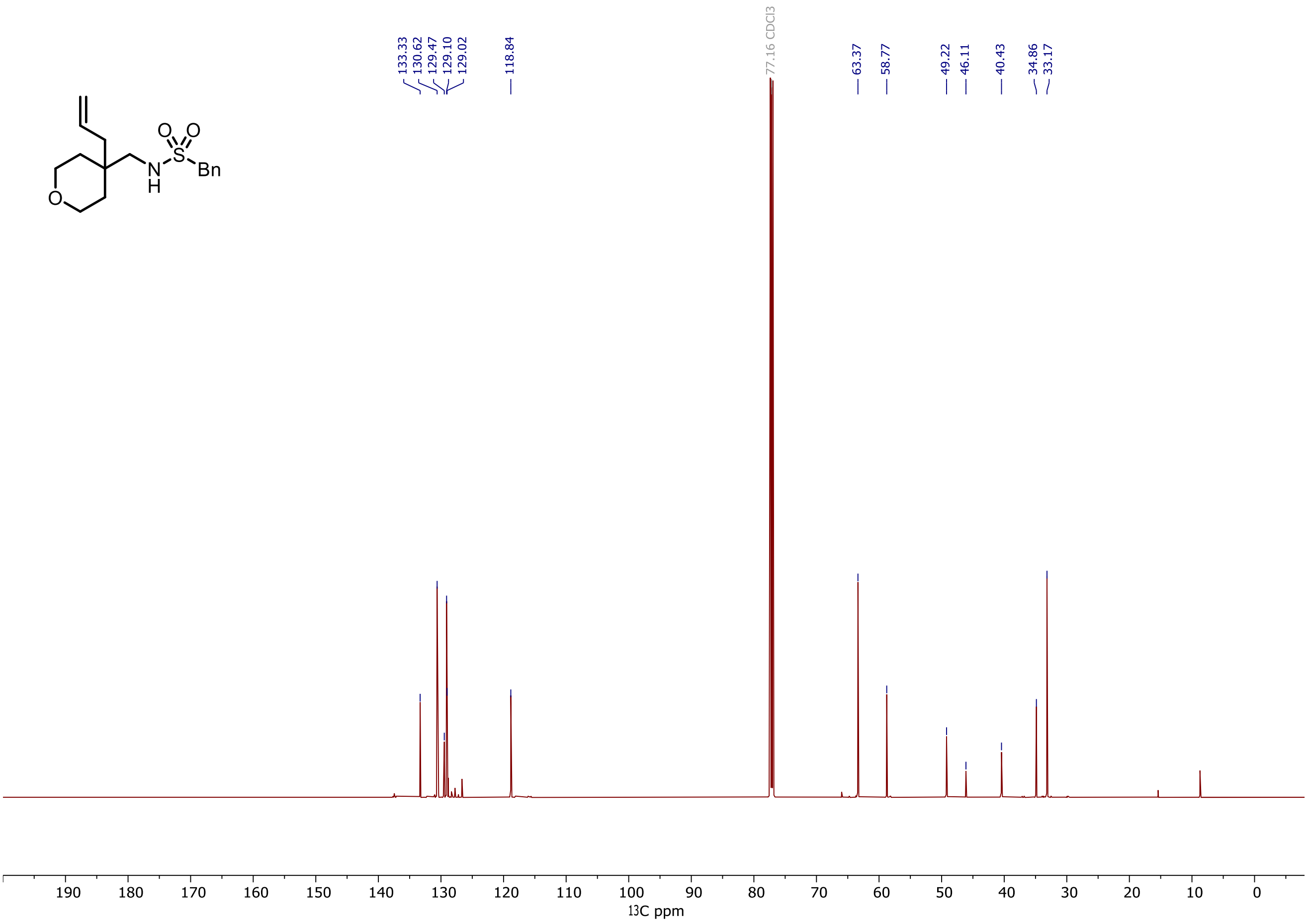

[back to table of contents]

[1]**H NMR** (600 MHz, $CDCl_3$) of compound **7o**

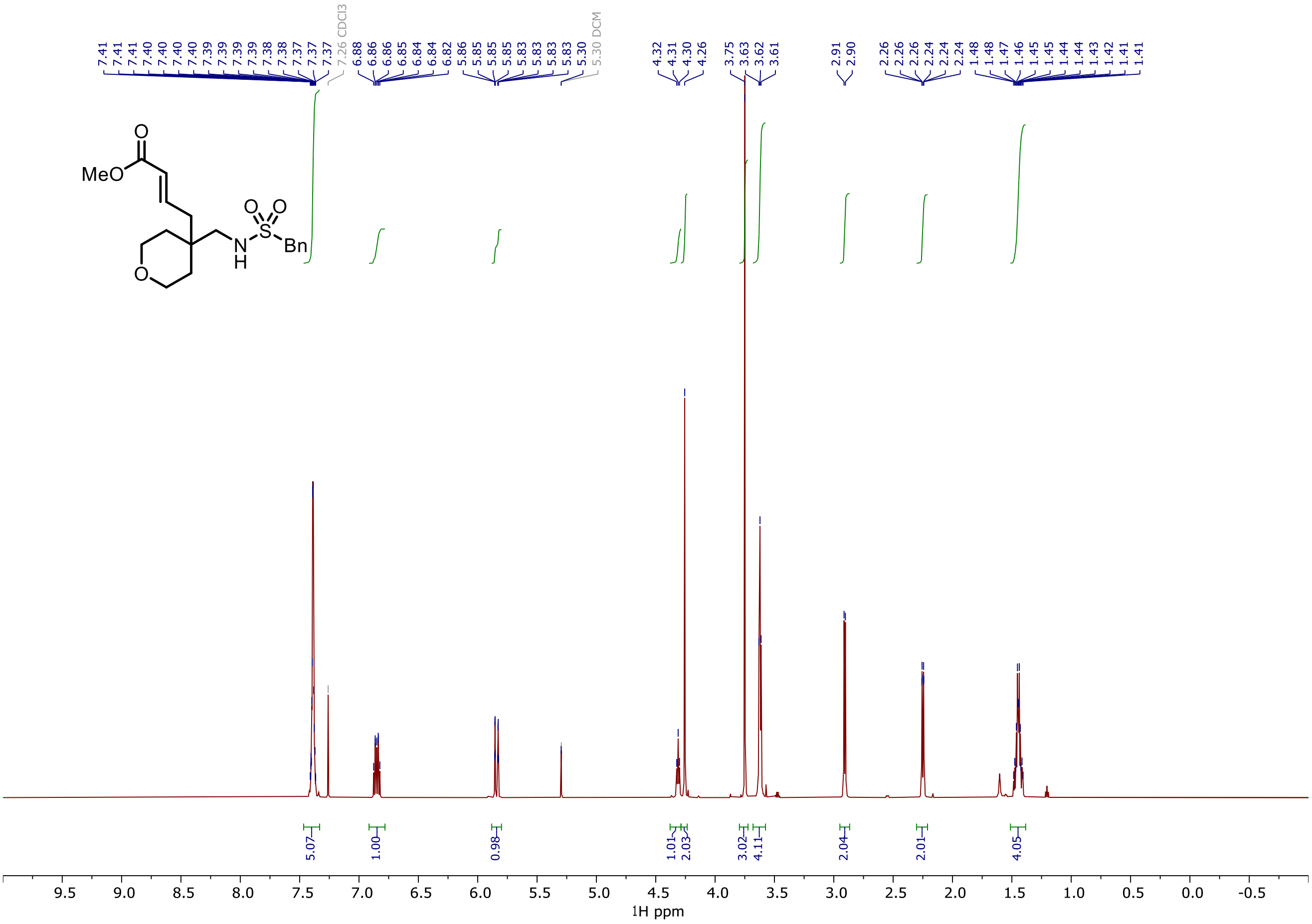

[13]**C NMR** (151 MHz, $CDCl_3$) of compound **7o**

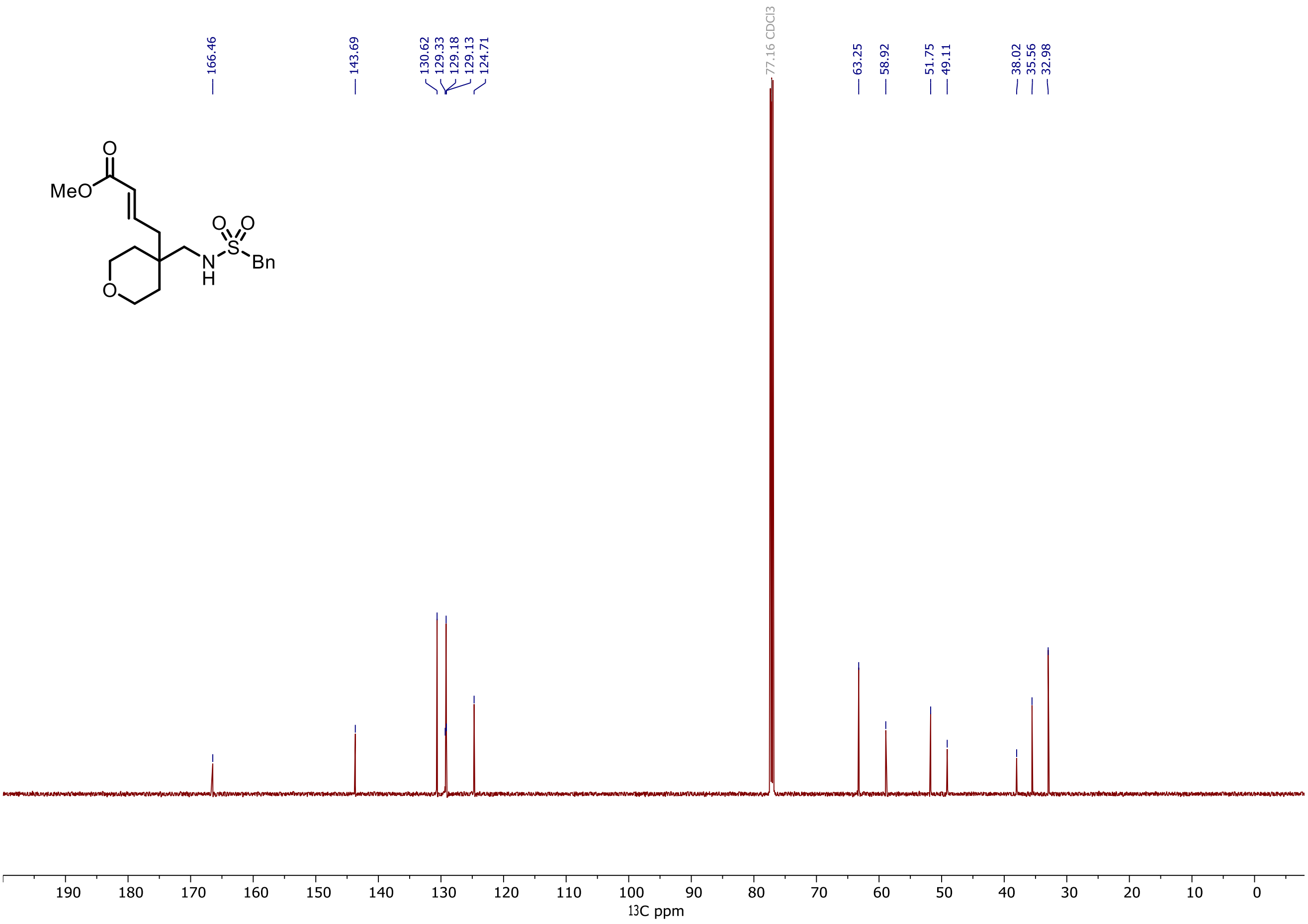

[back to table of contents]

^{1}H NMR (500 MHz, $CDCl_3$) of compound **S19** (Contains approx.. 8 wt% EtOAc)

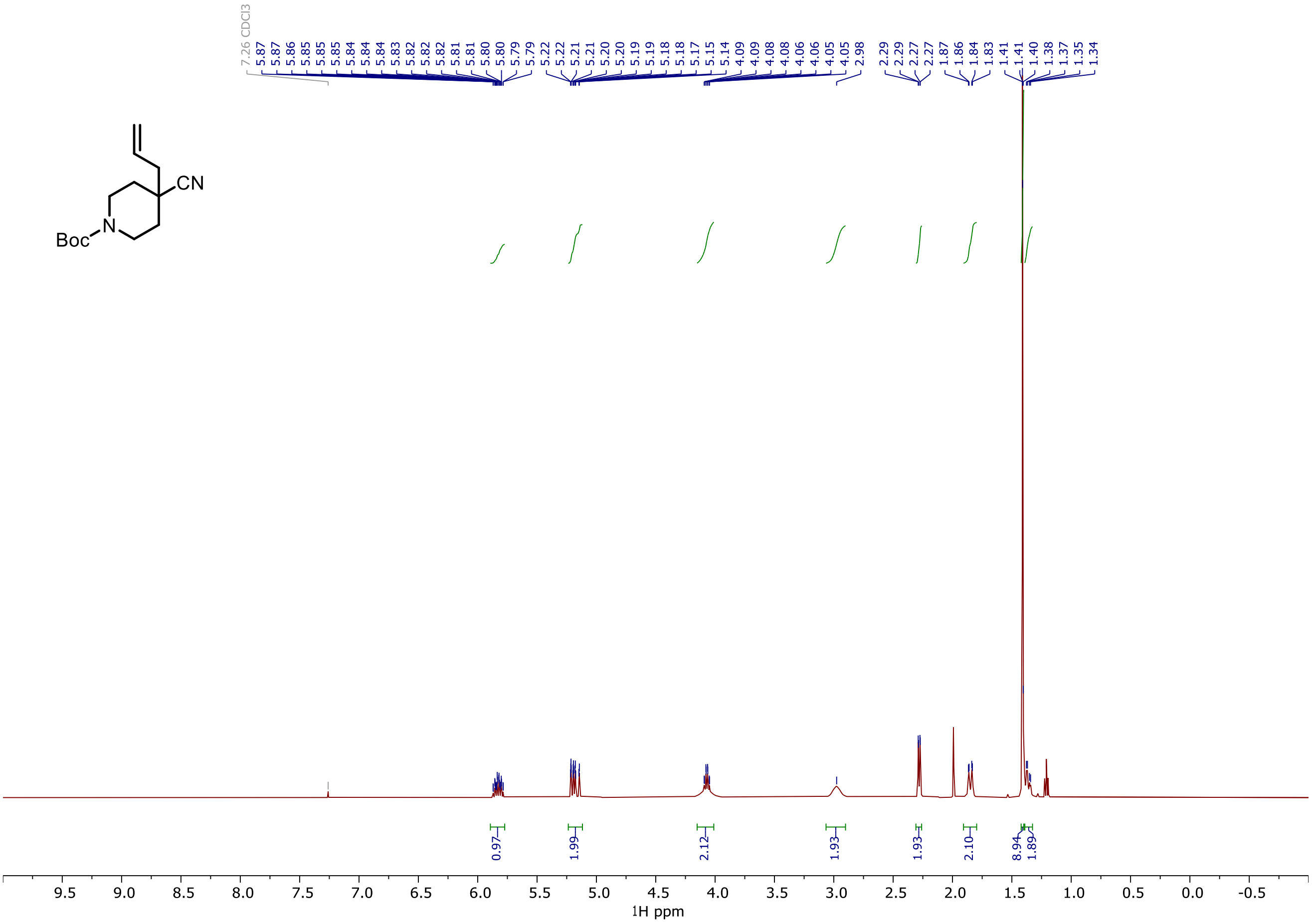

^{13}C NMR (126 MHz, $CDCl_3$) of compound **S19** (Contains approx.. 8 wt% EtOAc)

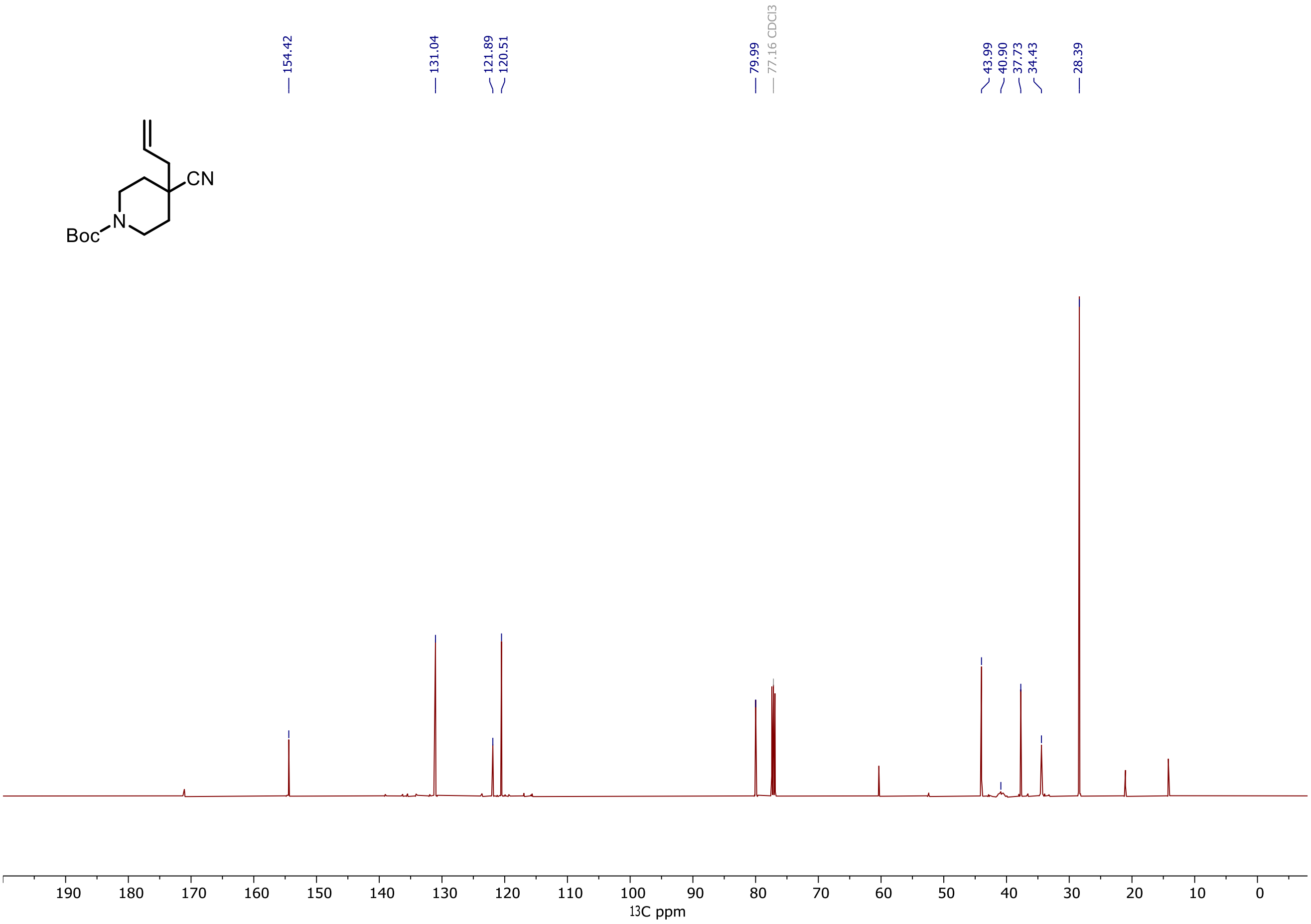

[back to table of contents]

¹H NMR (600 MHz, $CDCl_3$) of compound **S20**

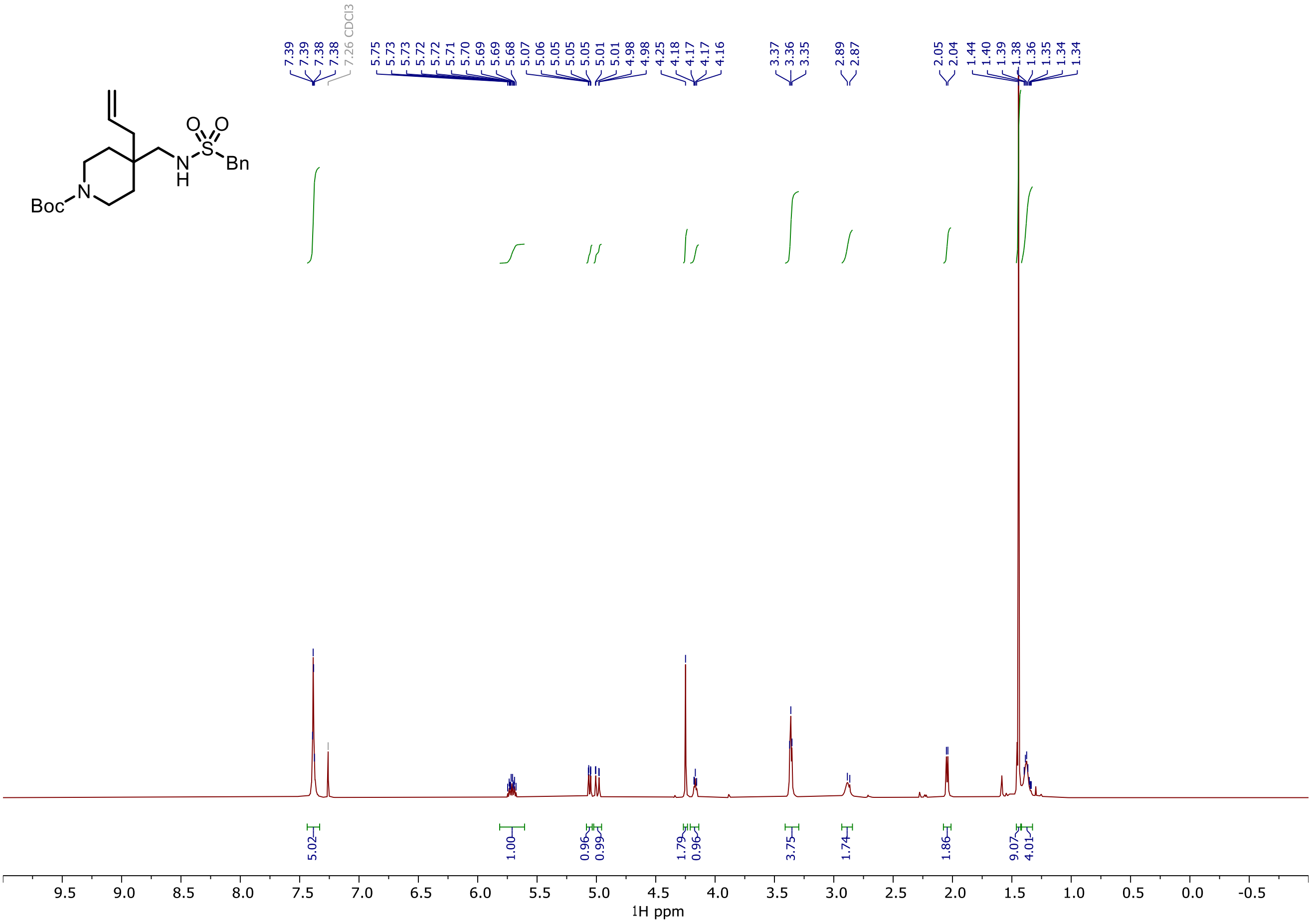

¹³C NMR (151 MHz, $CDCl_3$) of compound **S20**

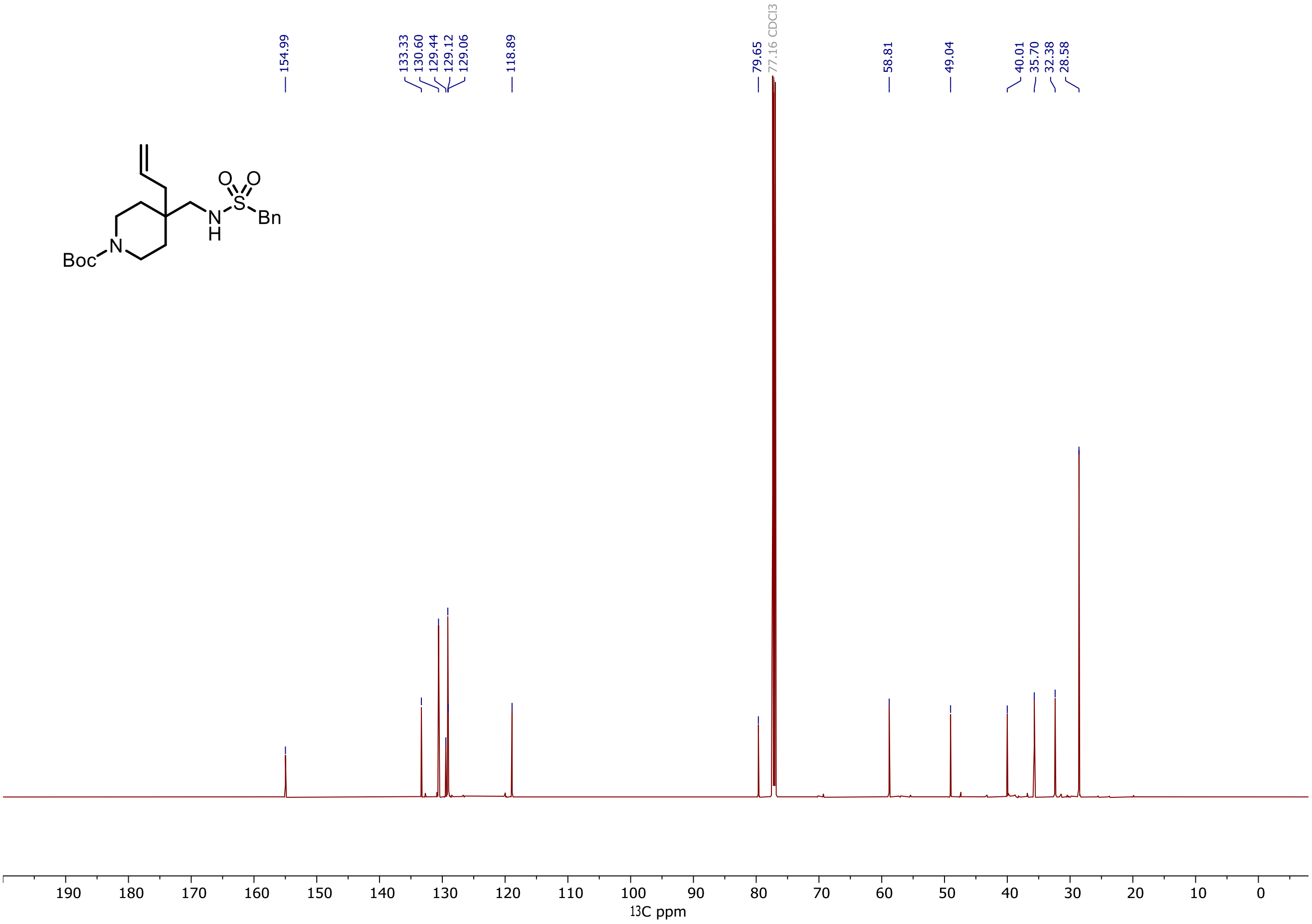

[back to table of contents]

¹H NMR (600 MHz, $CDCl_3$) of compound **7p**

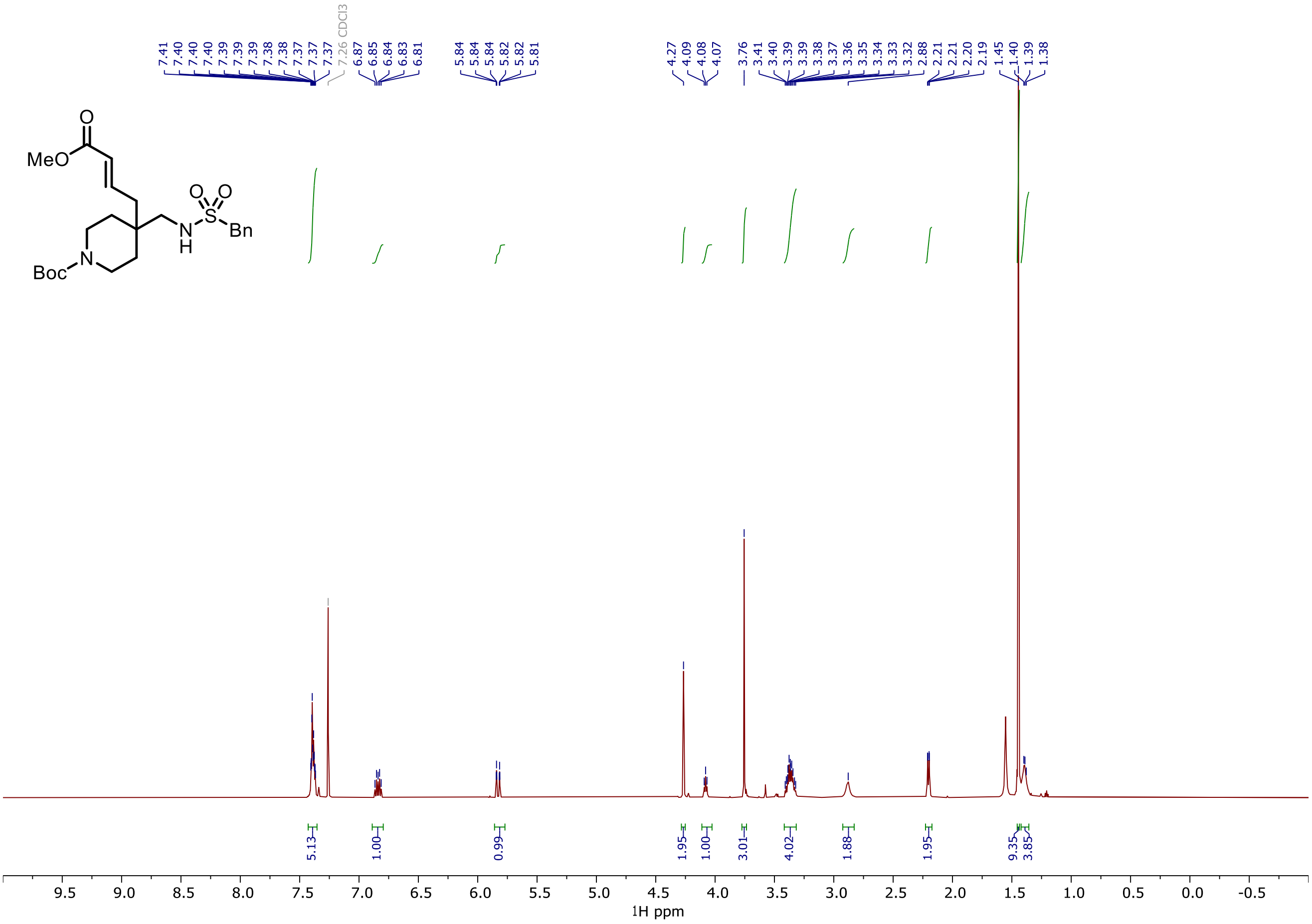

¹³C NMR (151 MHz, $CDCl_3$) of compound **7p**

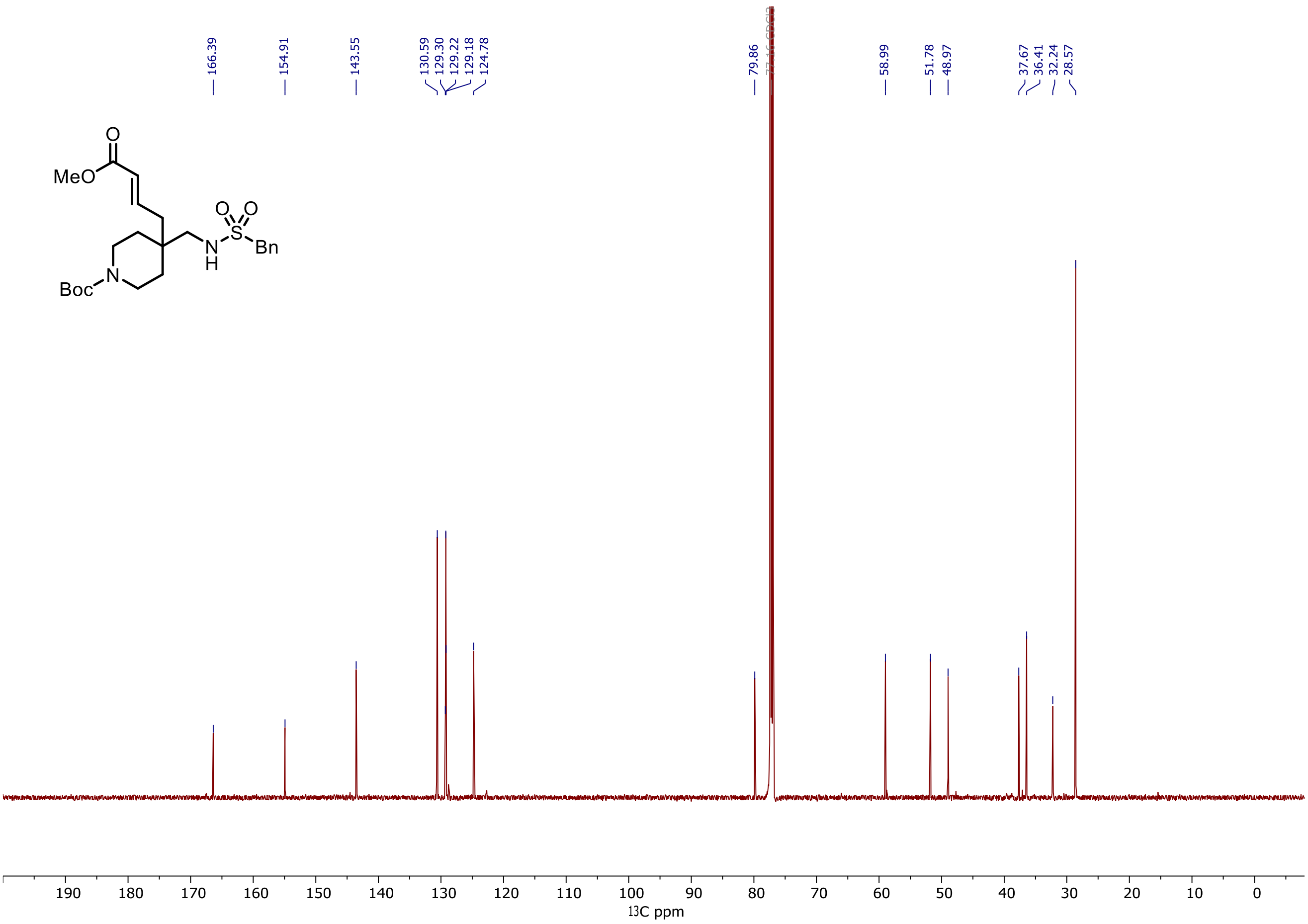

[back to table of contents]

^{1}H NMR (600 MHz, $CDCl_3$) of compound **S22**

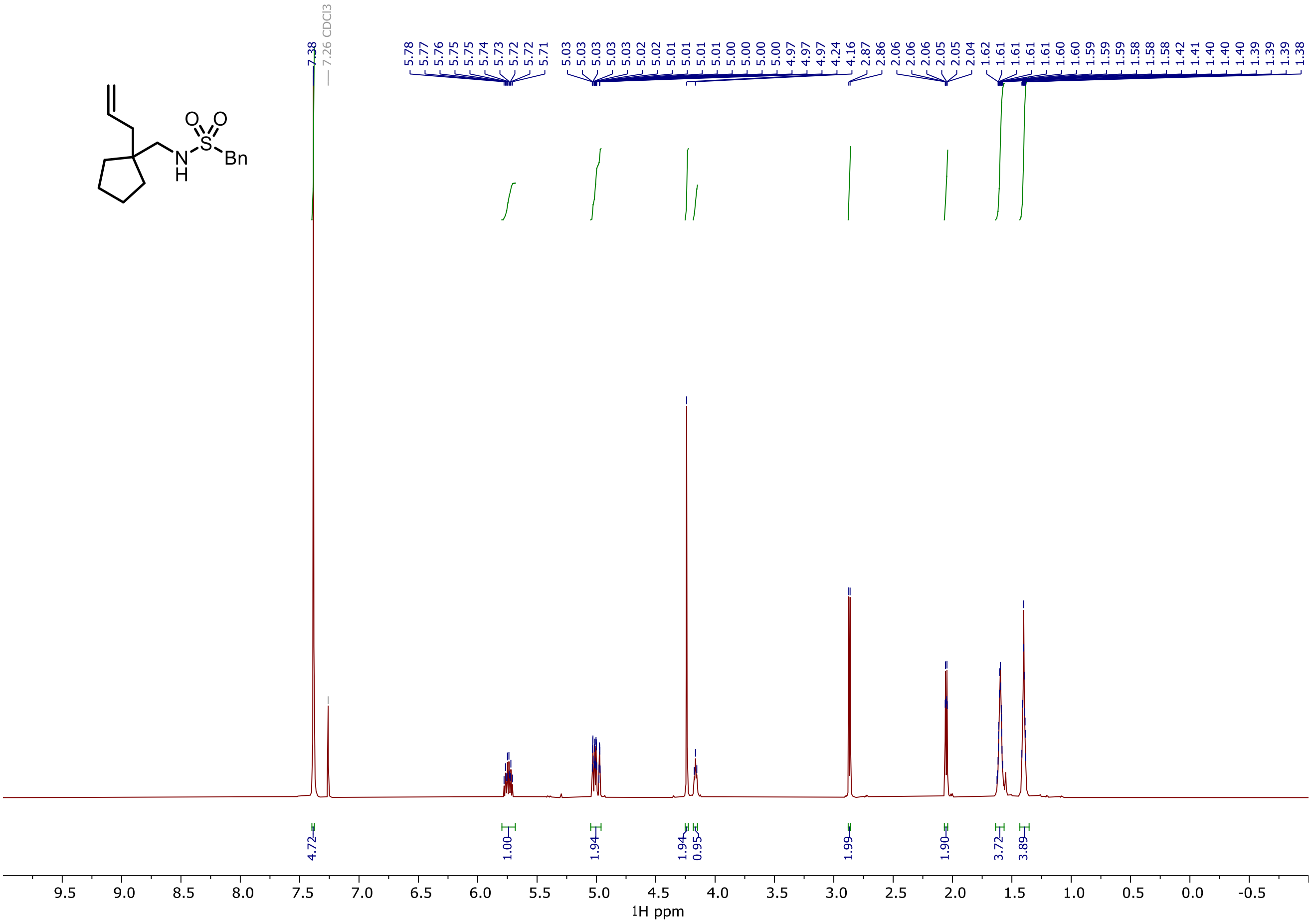

^{13}C NMR (151 MHz, $CDCl_3$) of compound **S22**

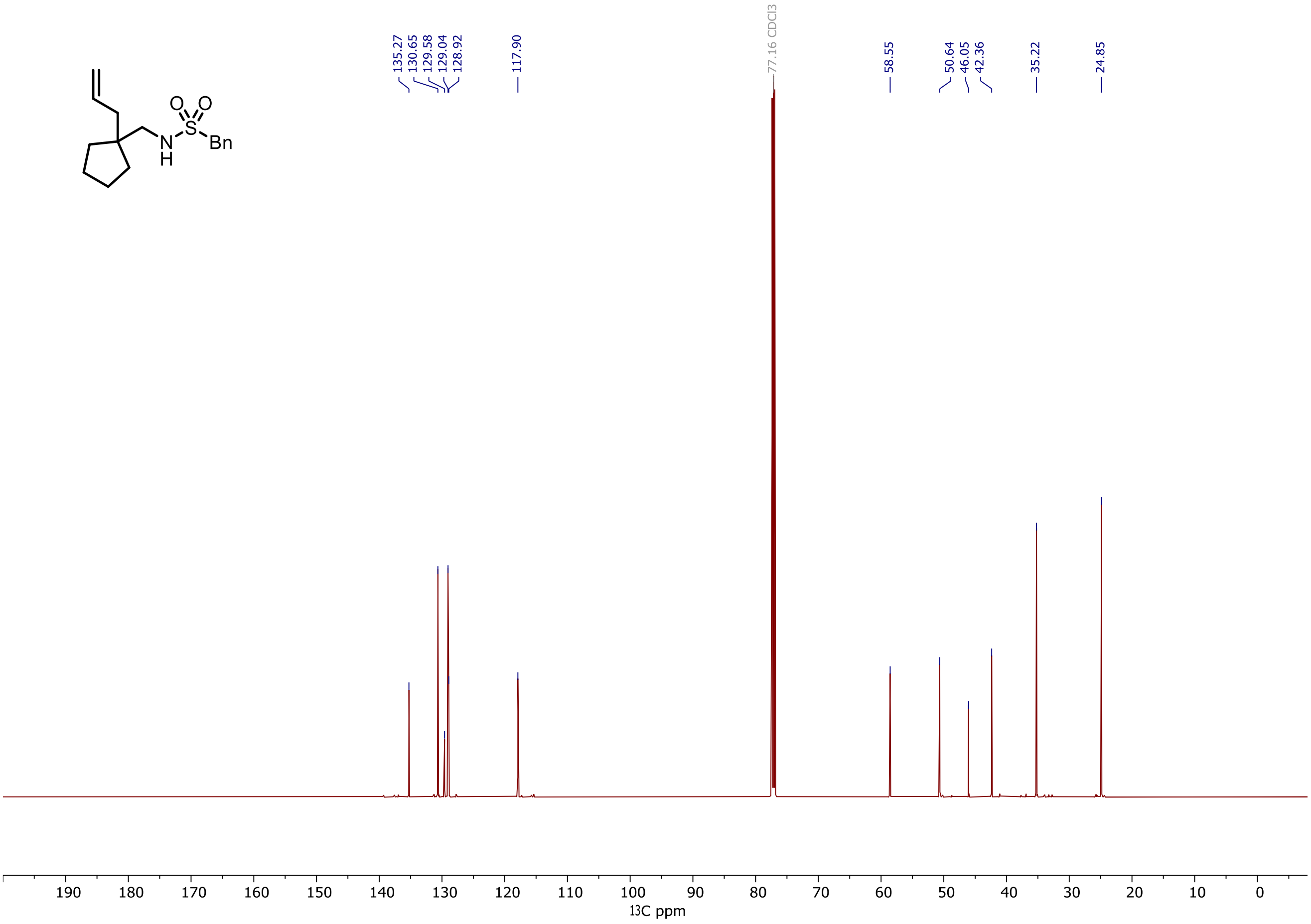

[back to table of contents]

^{1}H NMR (600 MHz, $CDCl_3$) of compound **7q**

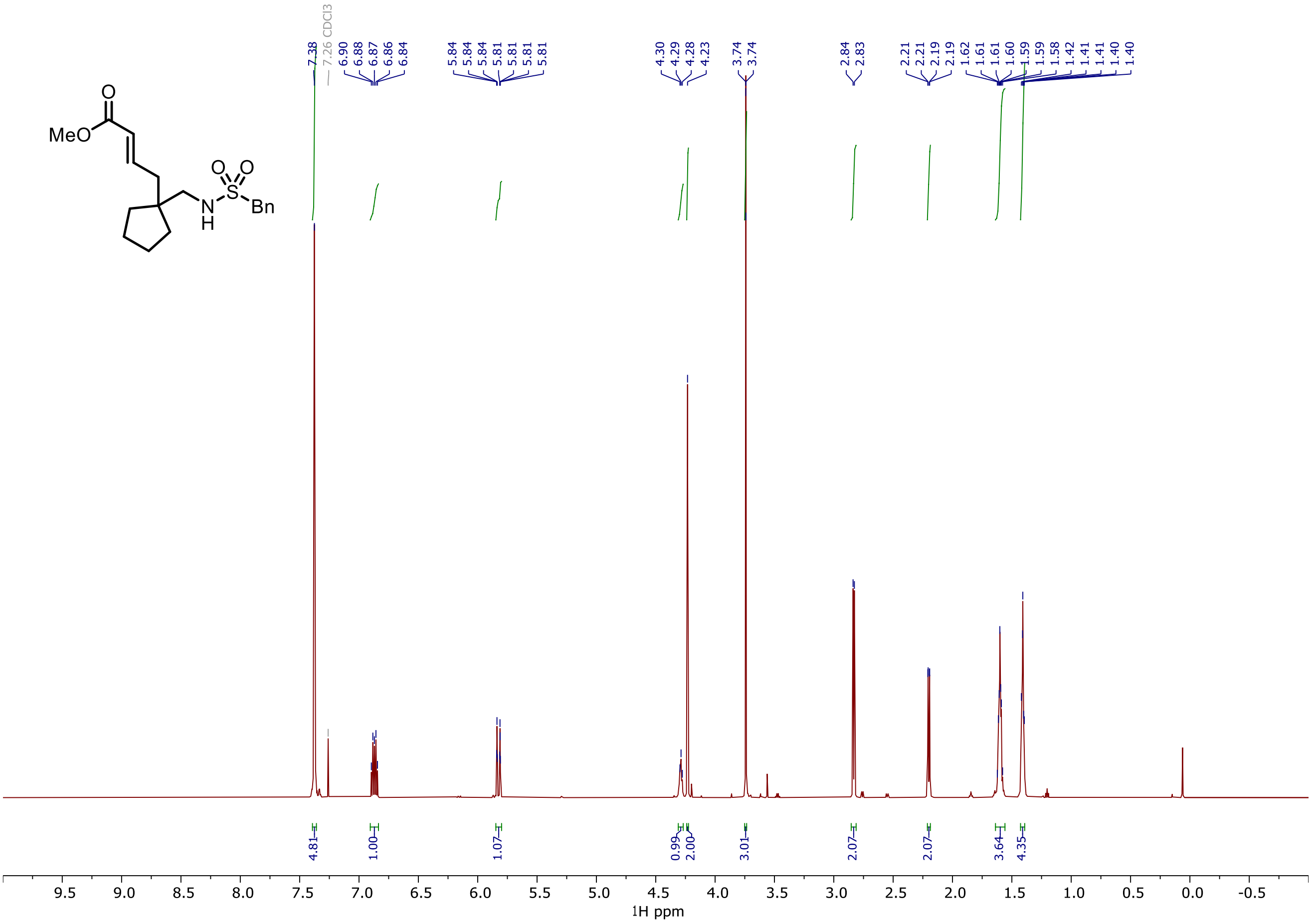

^{13}C NMR (151 MHz, $CDCl_3$) of compound **7q**

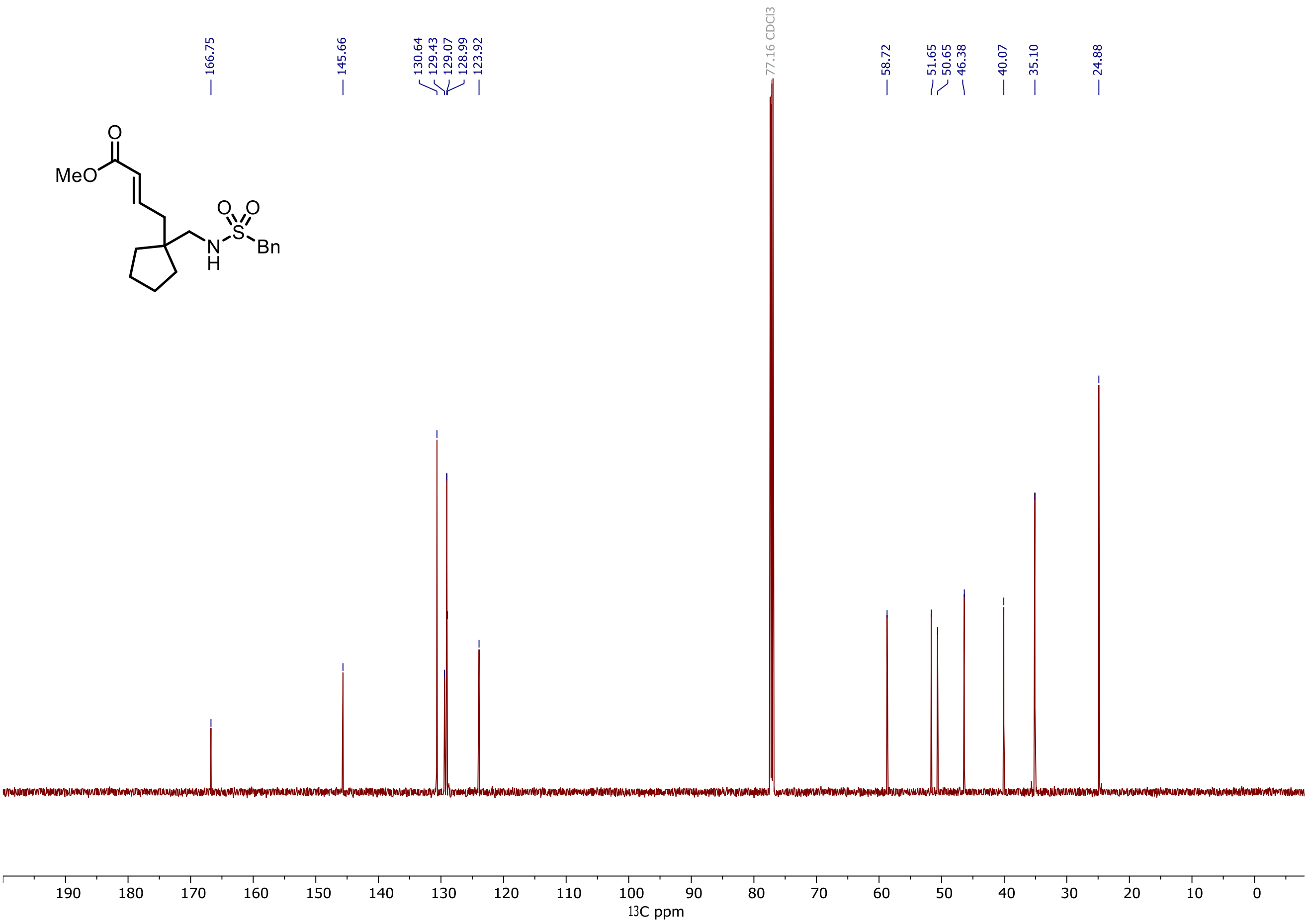

[back to table of contents]

¹H NMR (600 MHz, $CDCl_3$) of compound **S24**

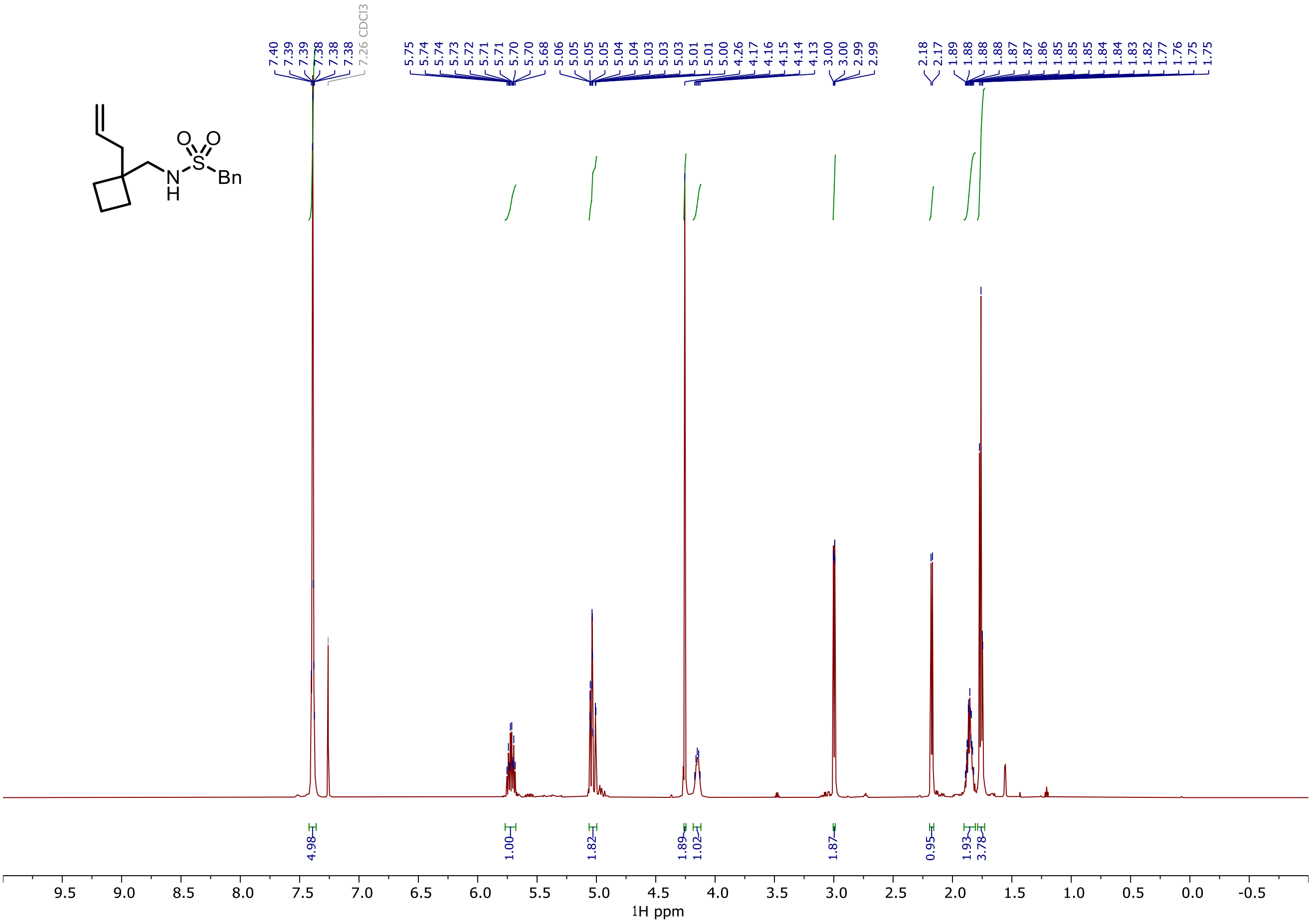

¹³C NMR (151 MHz, $CDCl_3$) of compound **S24**

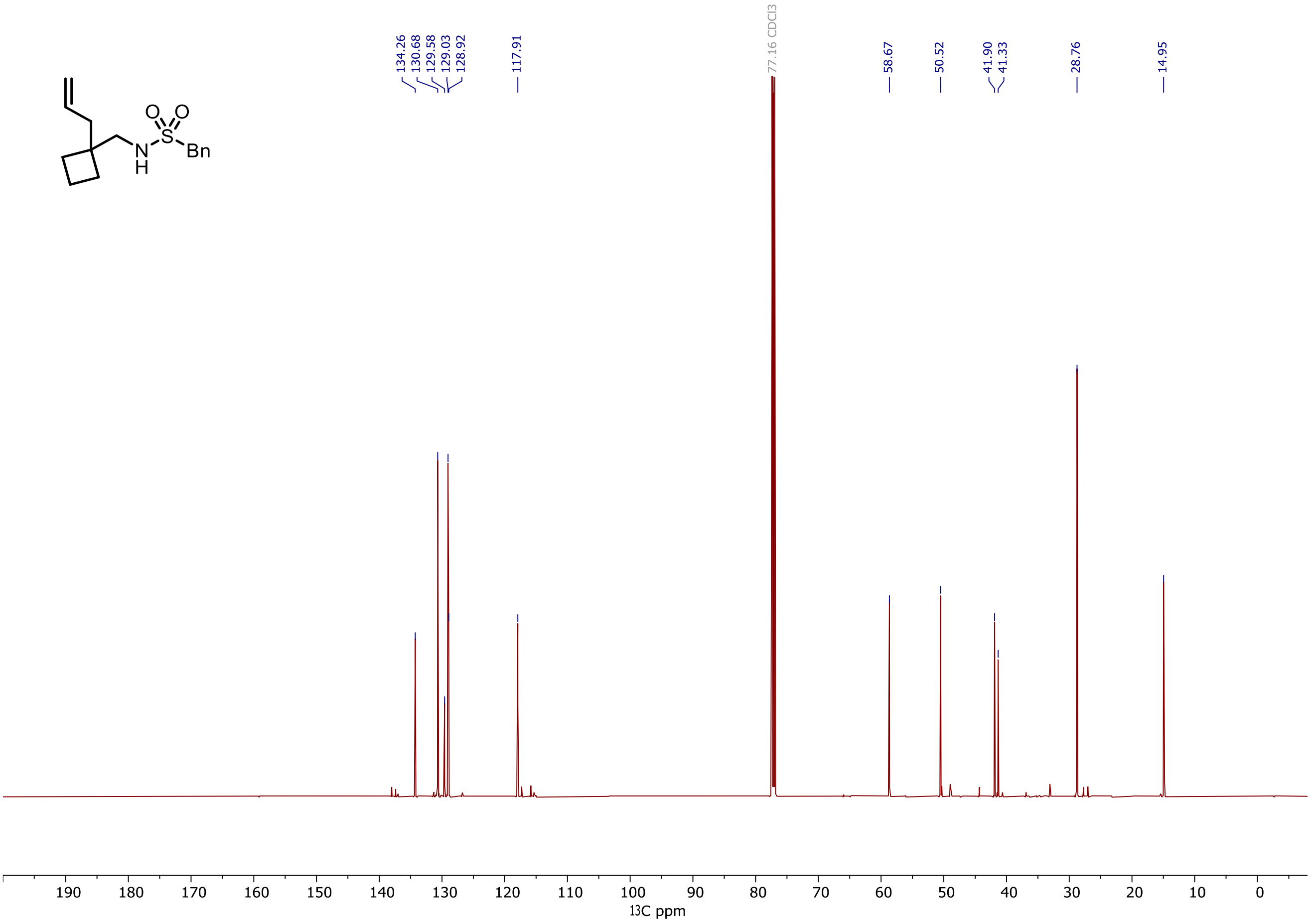

[back to table of contents]

^{1}H NMR (600 MHz, $CDCl_3$) of compound **7r**

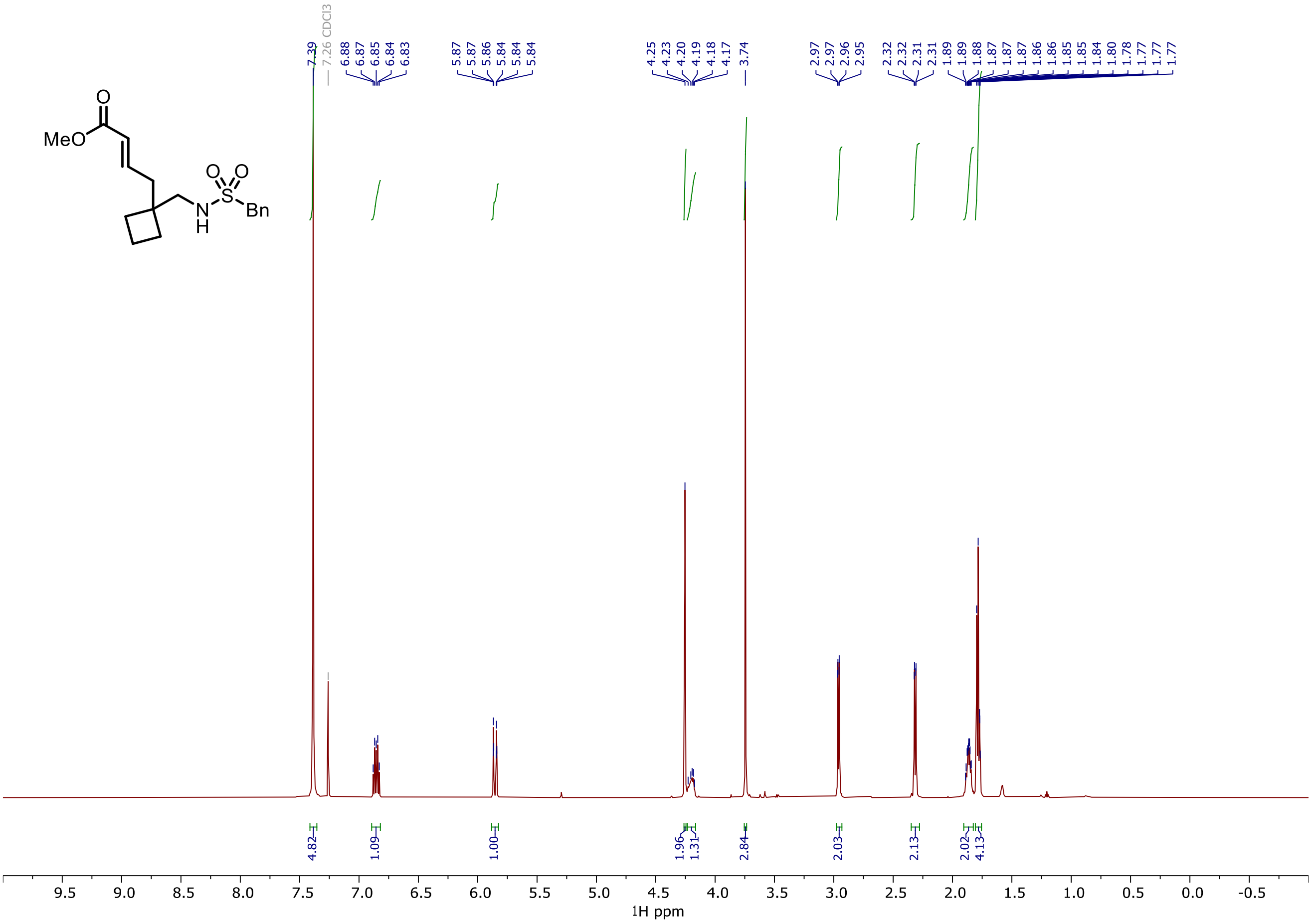

^{13}C NMR (151 MHz, $CDCl_3$) of compound **7r**

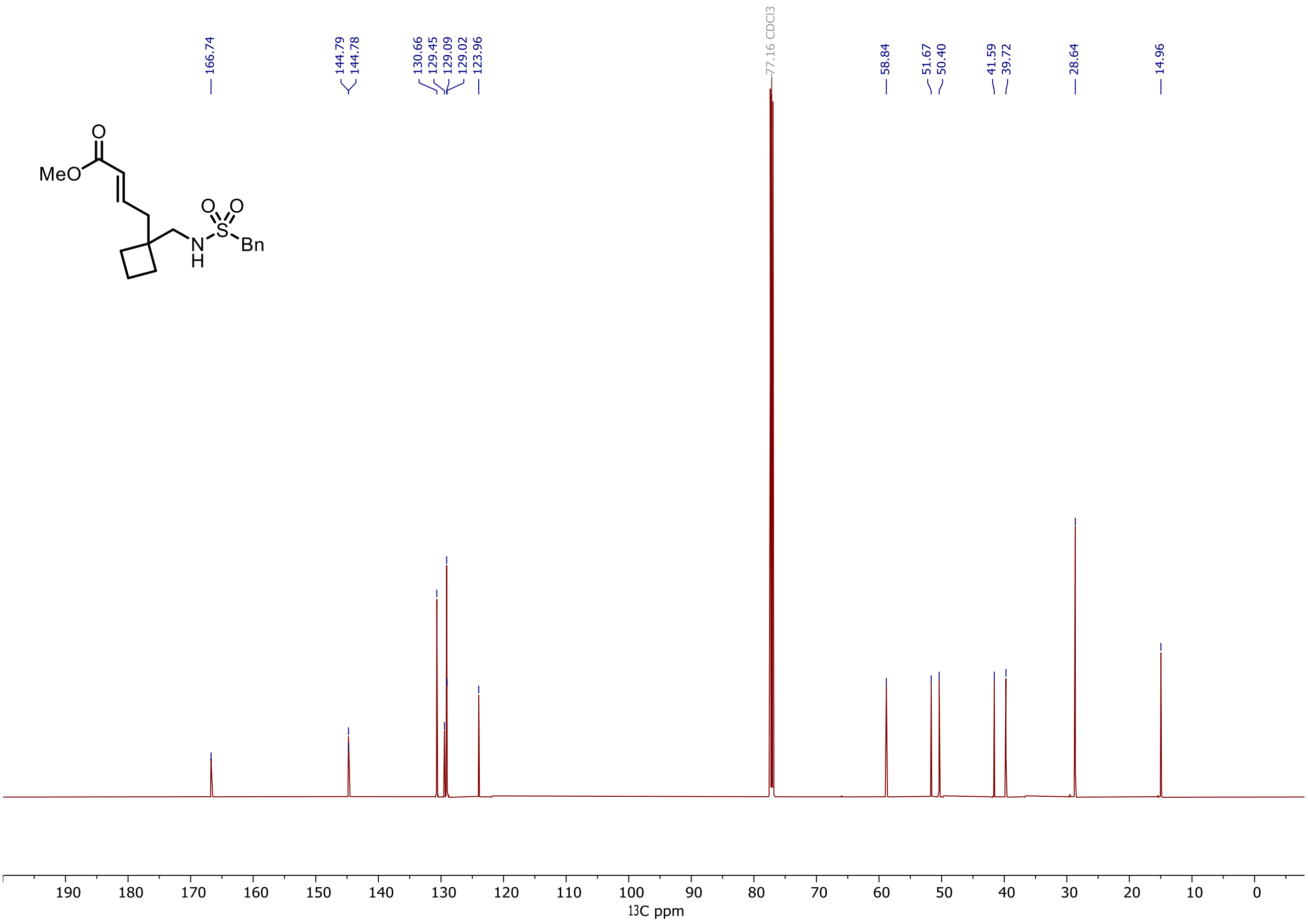

[back to table of contents]

^{1}H NMR (600 MHz, $CDCl_3$) of compound **7s**

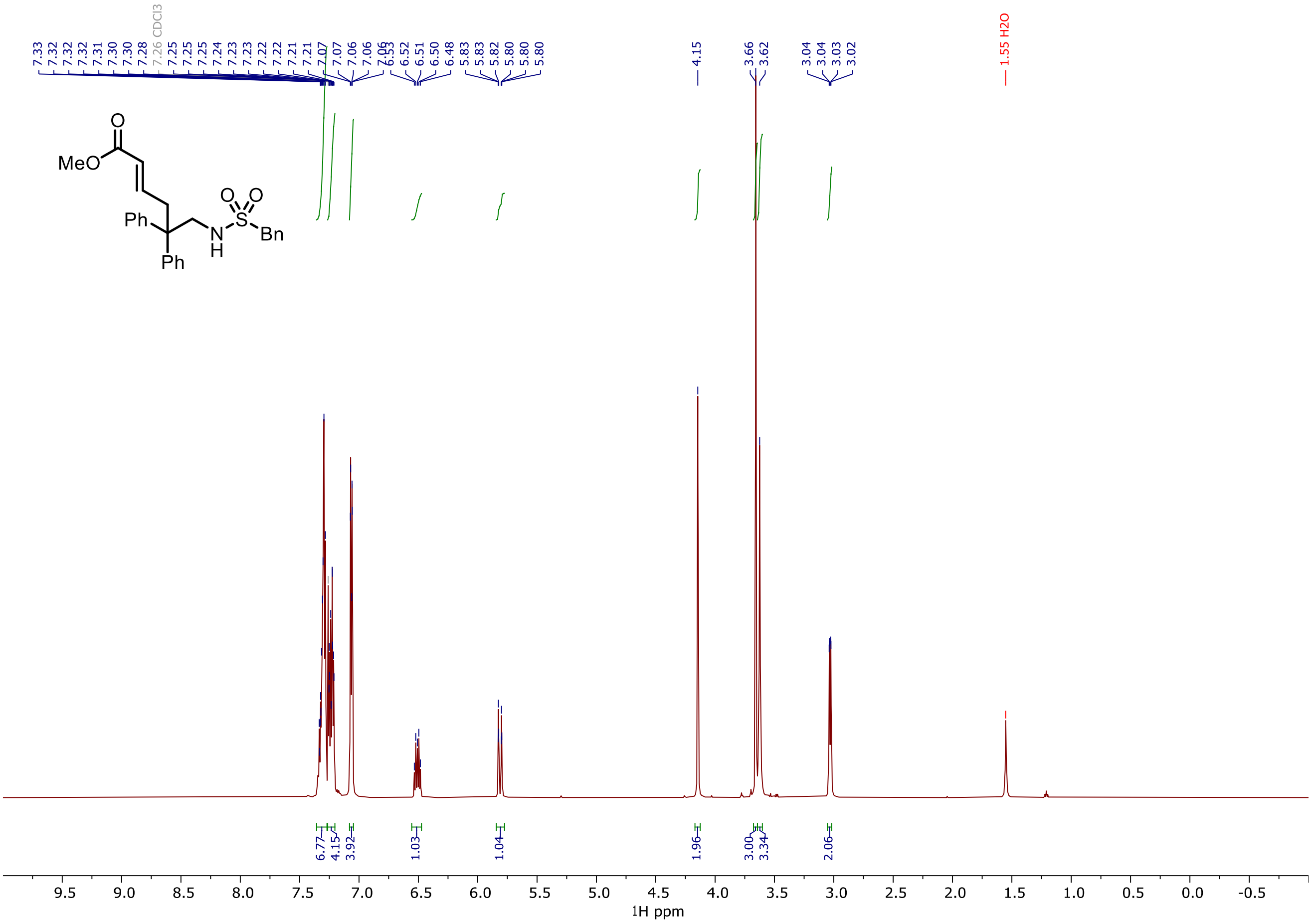

^{13}C NMR (151 MHz, $CDCl_3$) of compound **7s**

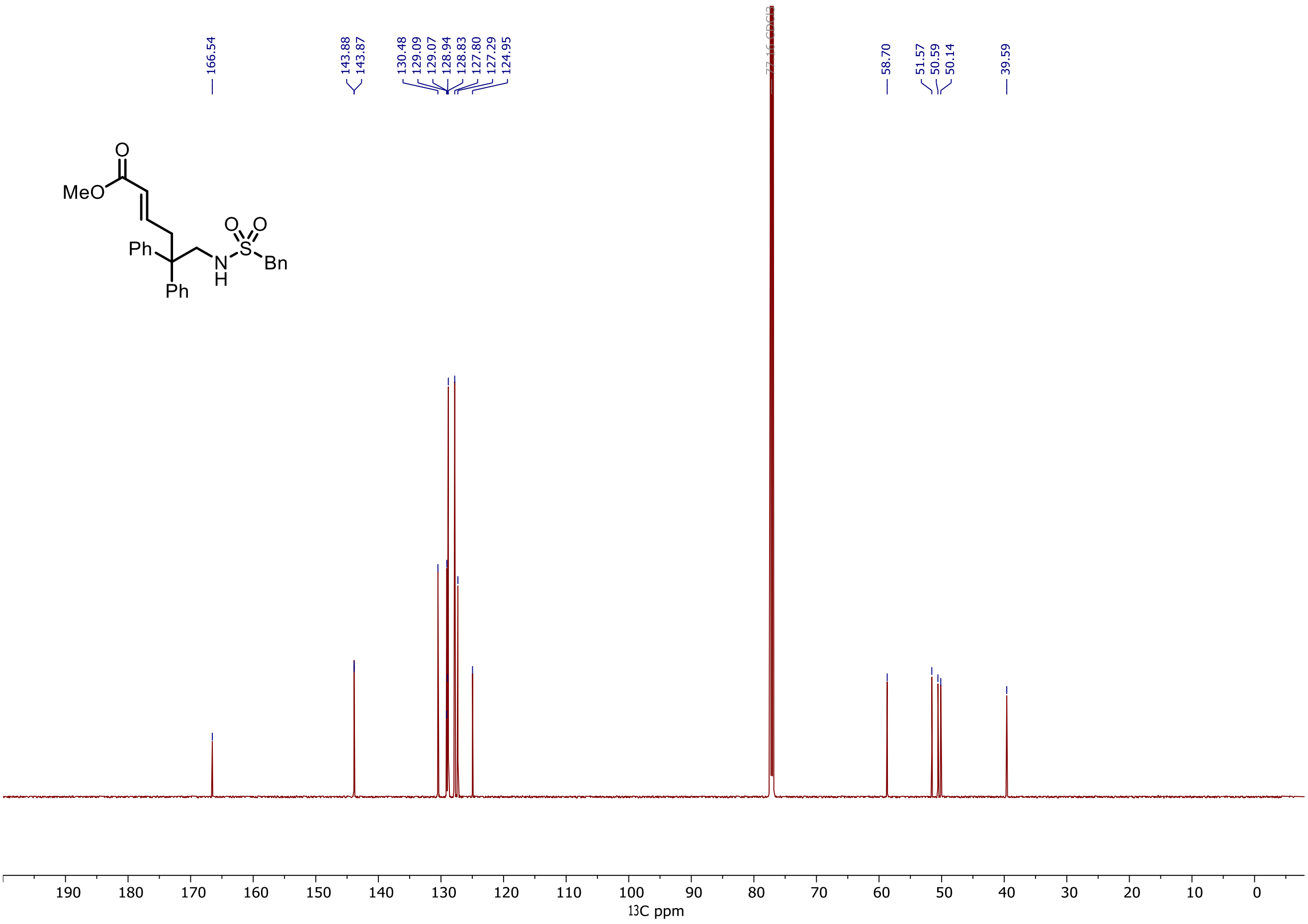

[back to table of contents]

^{1}H NMR (400 MHz, $CDCl_3$) of compound **7t**

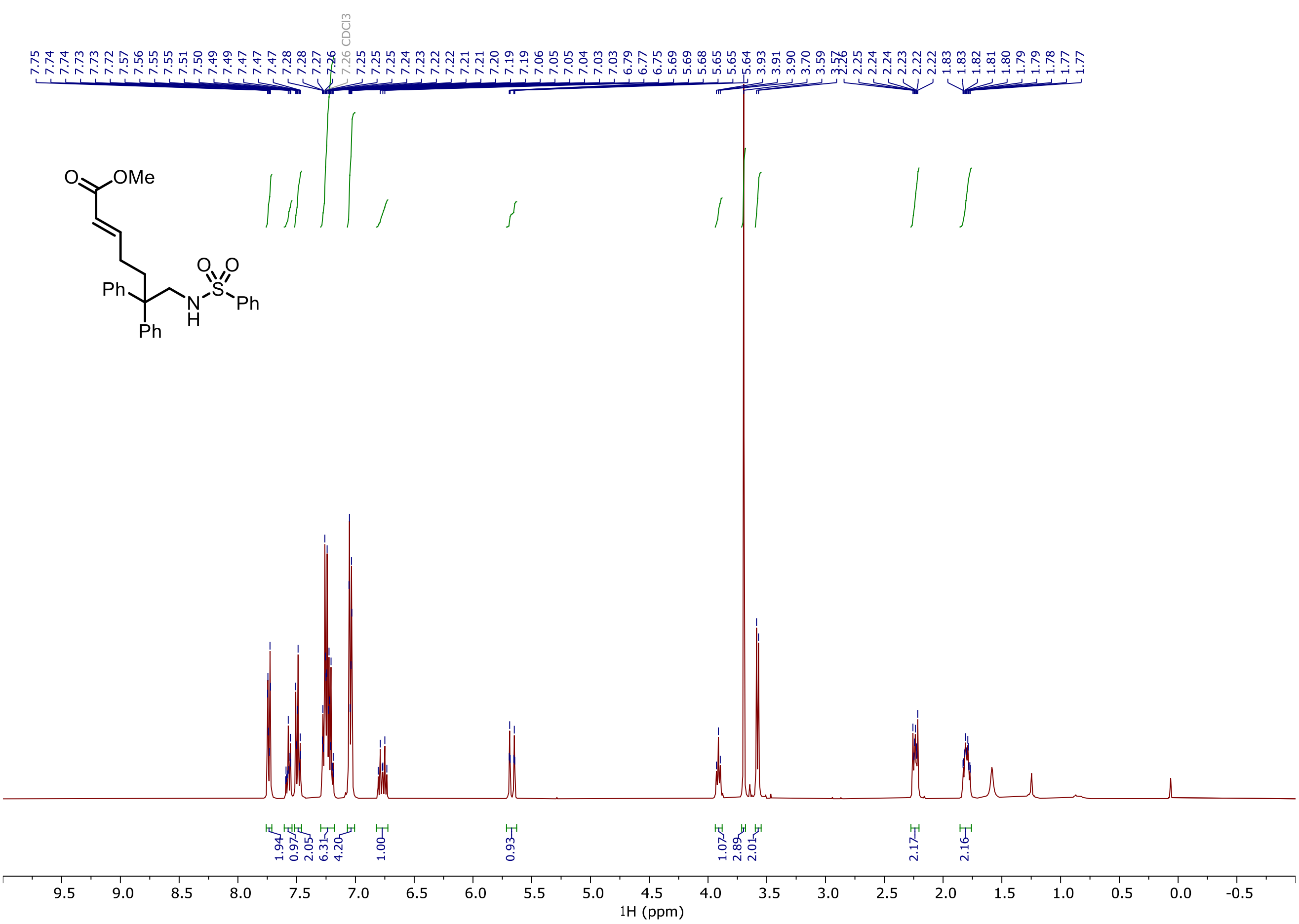

^{13}C NMR (101 MHz, $CDCl_3$) of compound **7t**

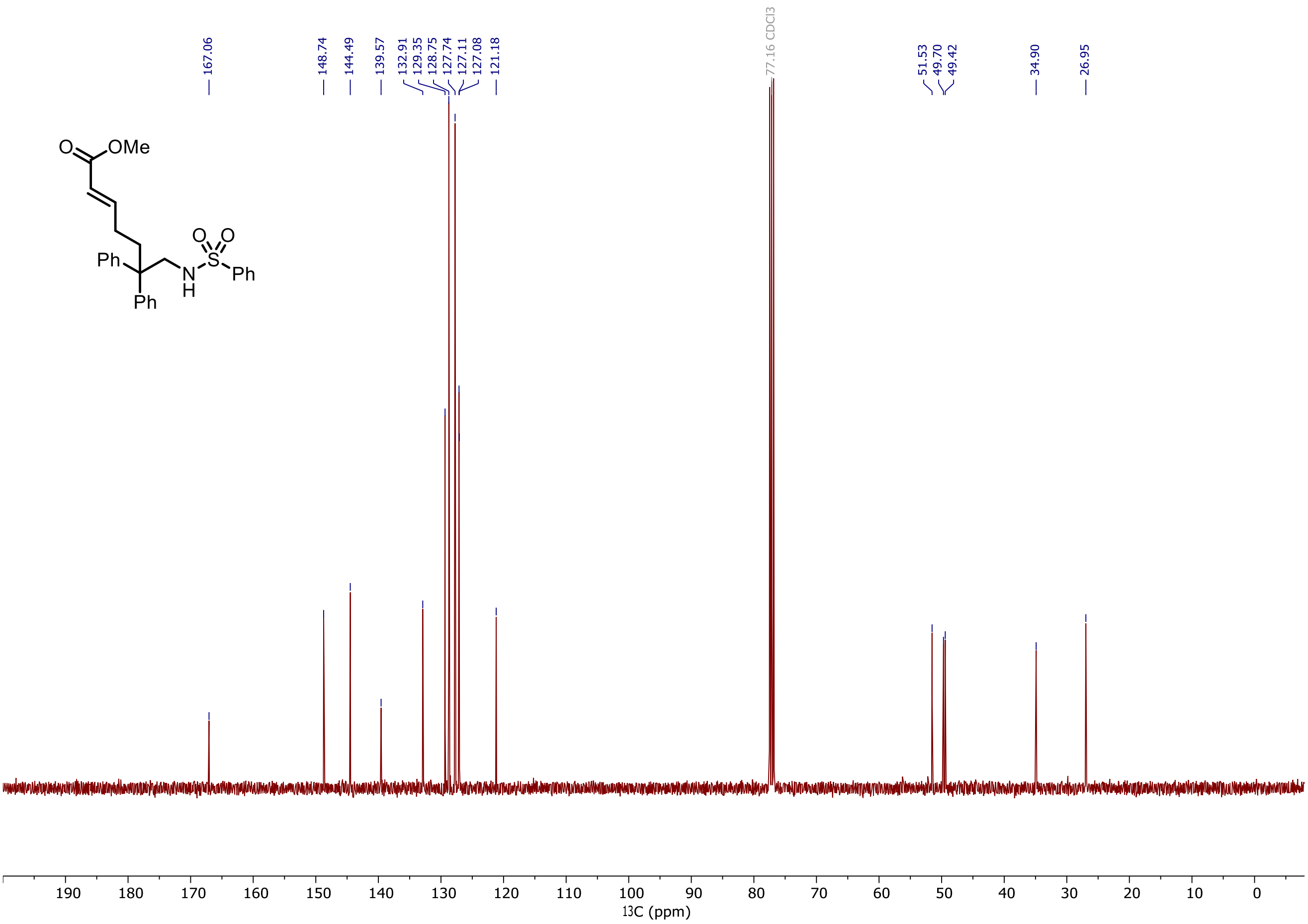

[back to table of contents]

^{1}H NMR (600 MHz, $CDCl_3$) of compound **S28**

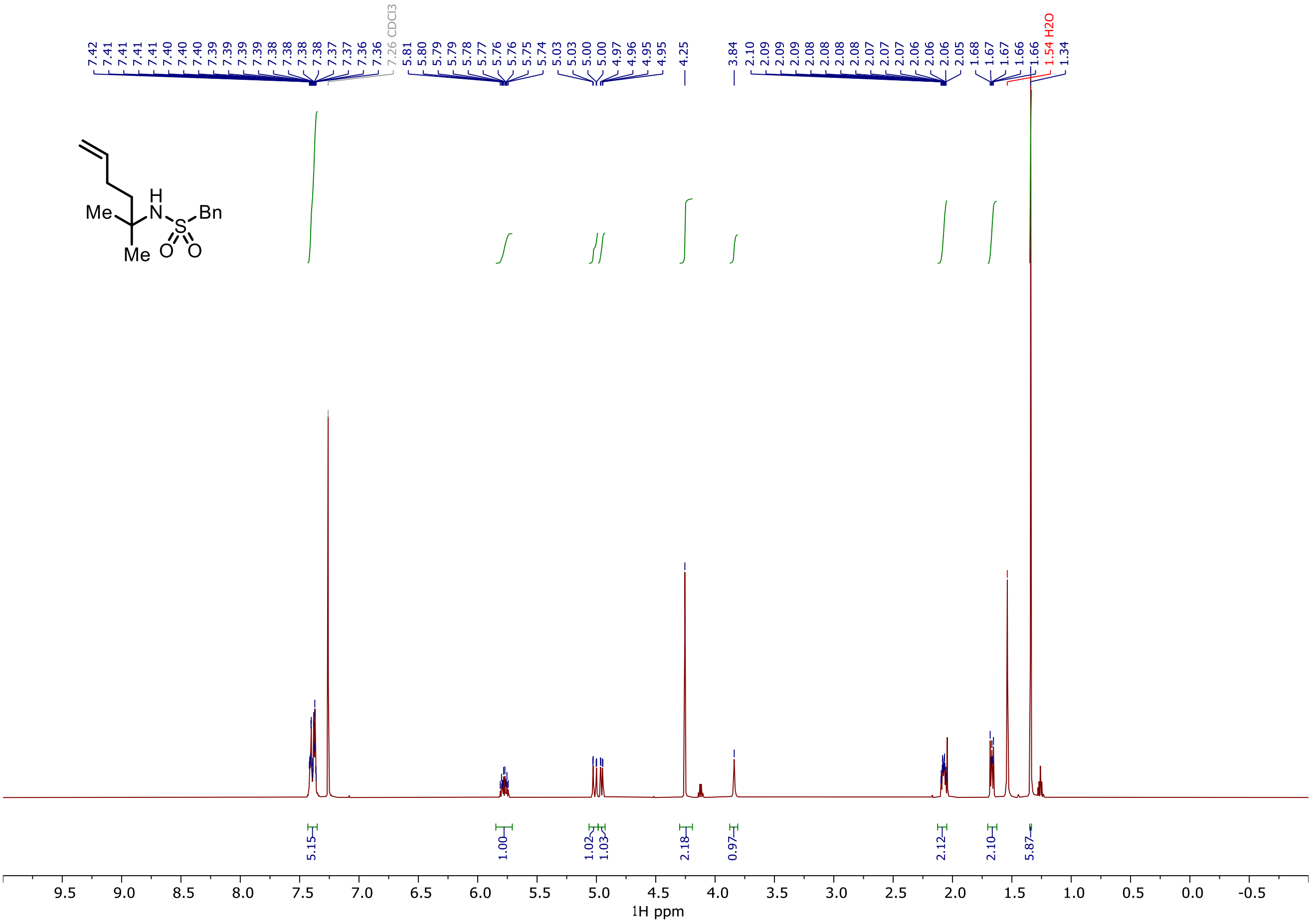

^{13}C NMR (151 MHz, $CDCl_3$) of compound **S28**

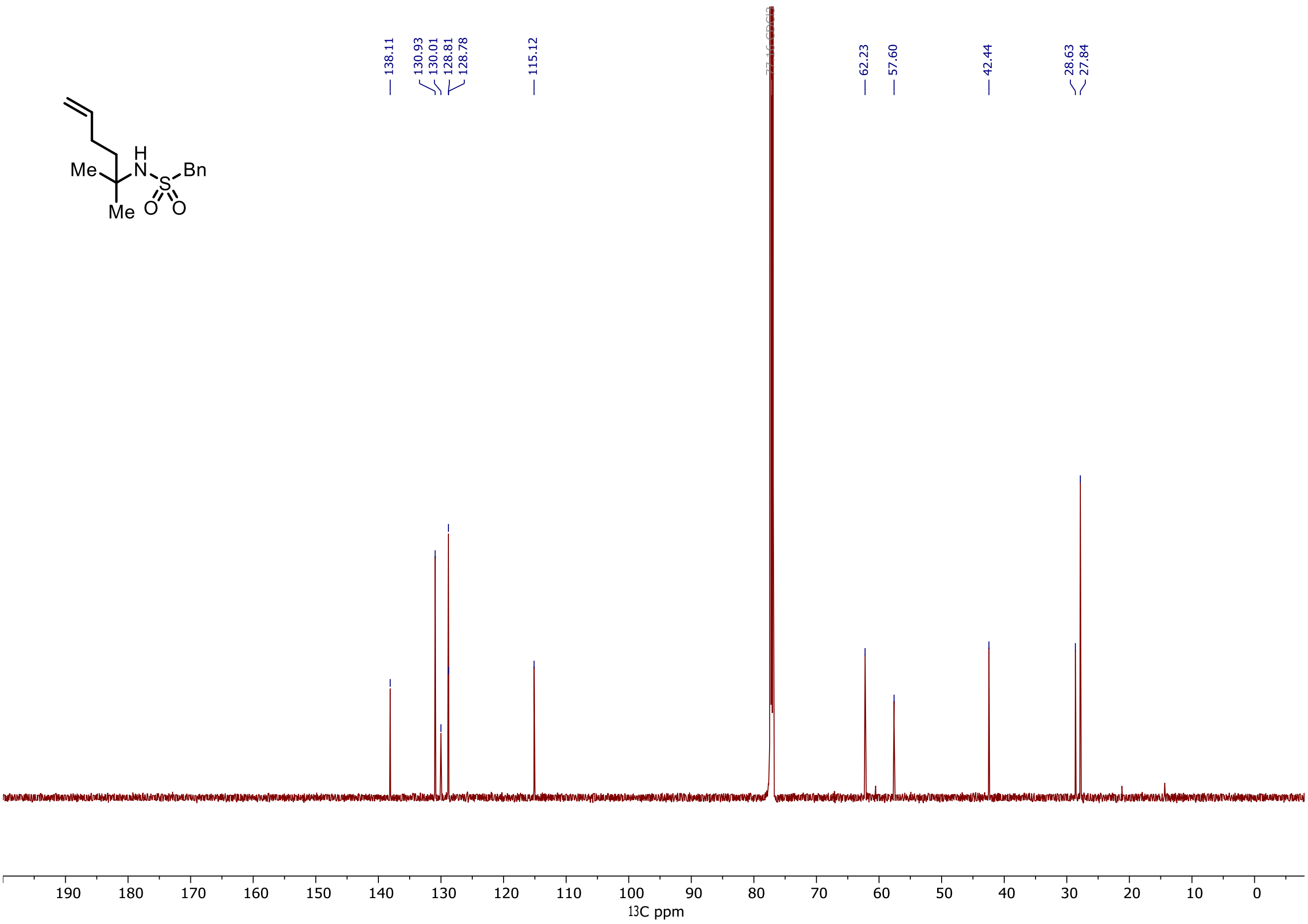

[back to table of contents]

^{1}H NMR (600 MHz, $CDCl_3$) of compound **7u**

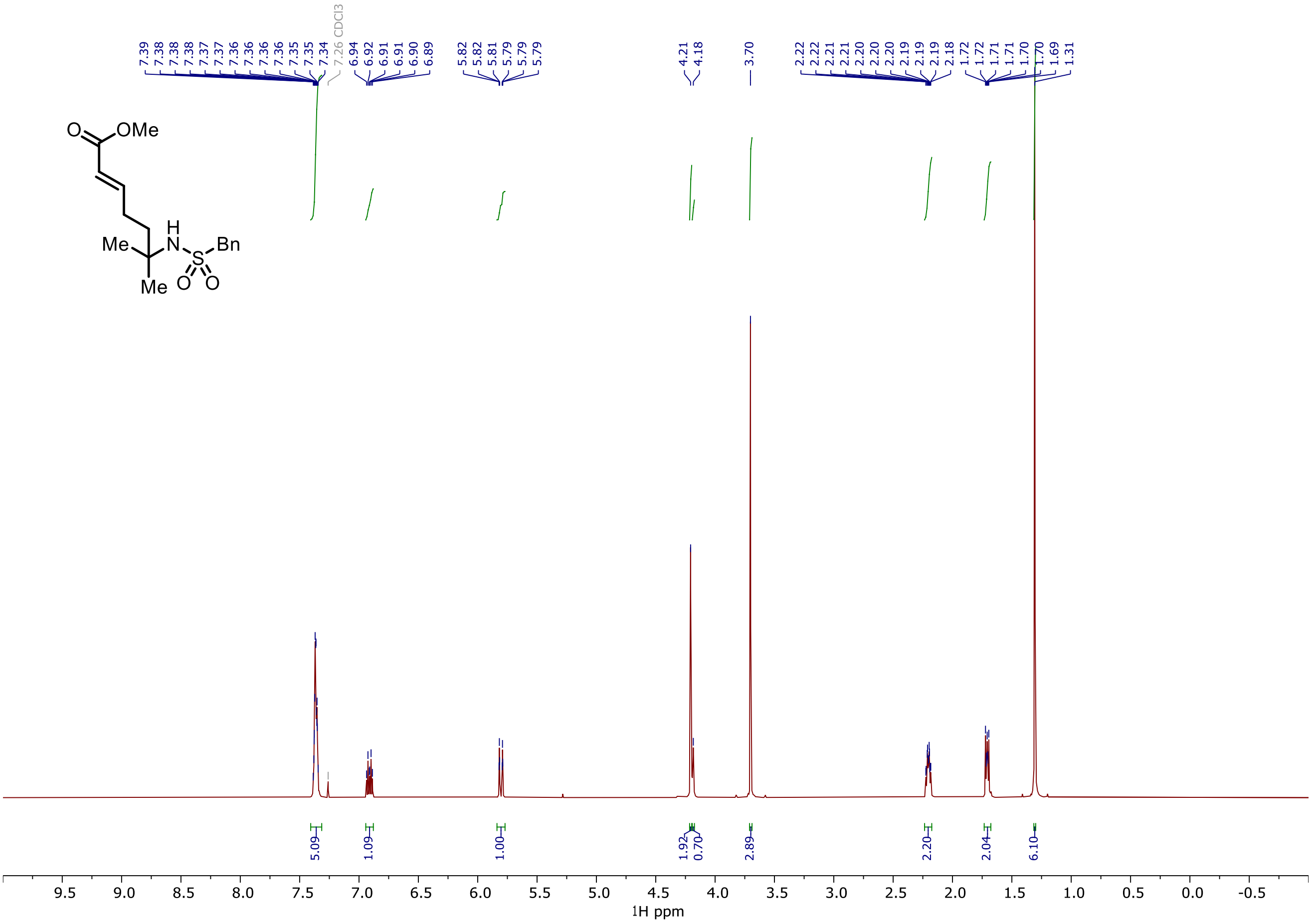

^{13}C NMR (151 MHz, $CDCl_3$) of compound **7u**

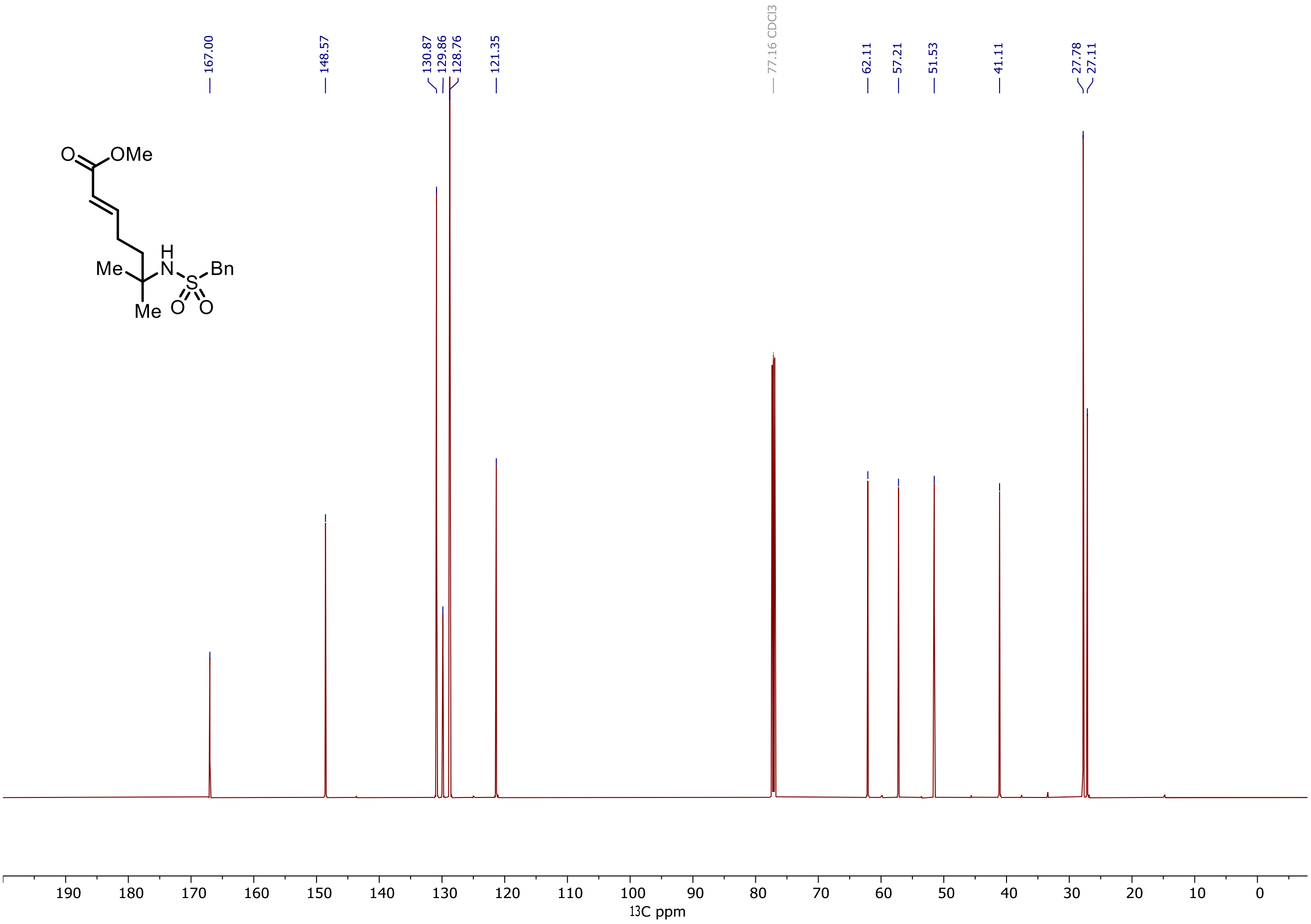

[back to table of contents]

^{1}H NMR (600 MHz, $CDCl_3$) of compound **S30** (Contains approx. 3 wt% EtOAc)

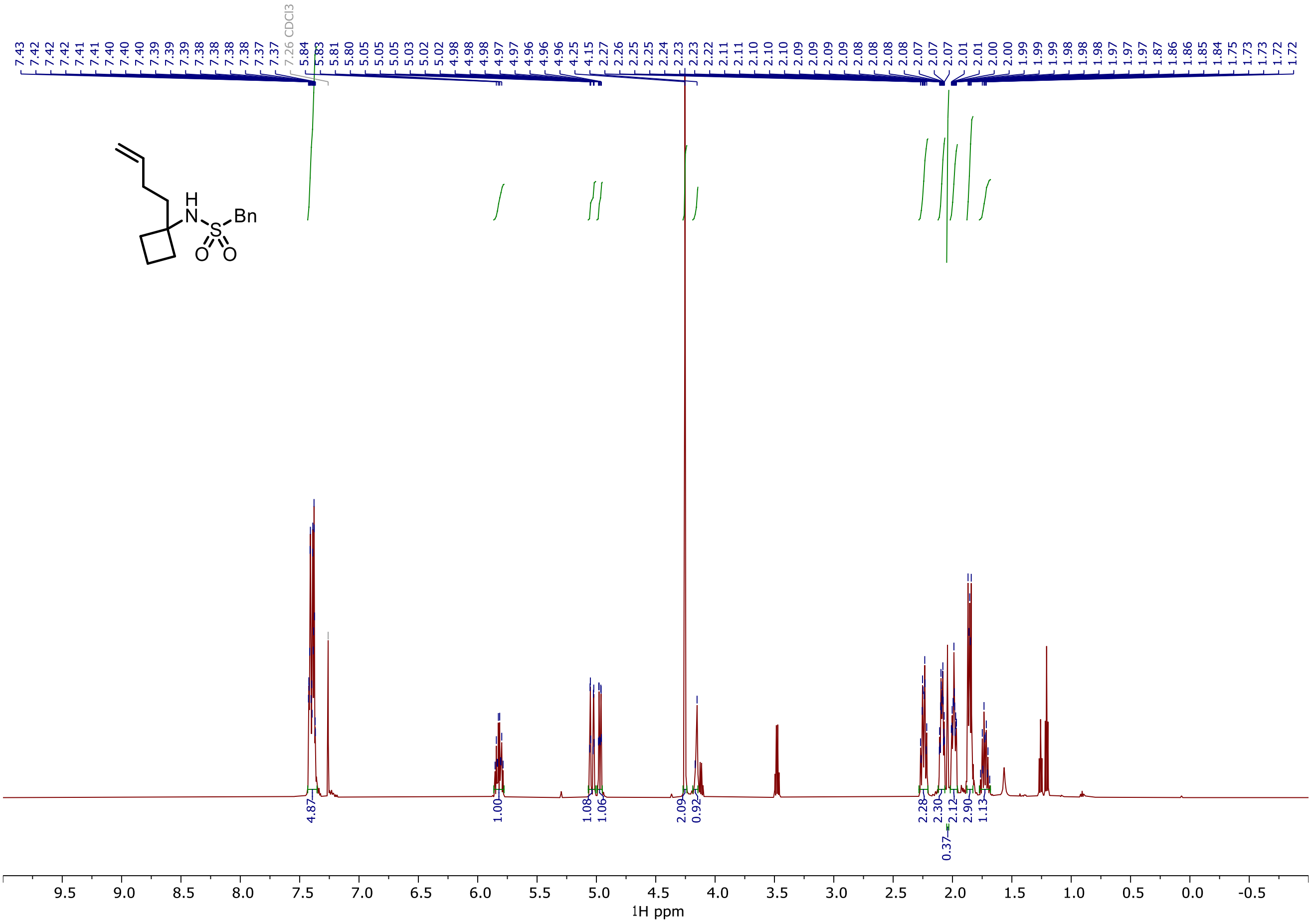

^{13}C NMR (151 MHz, $CDCl_3$) of compound **S30** (Contains approx. 3 wt% EtOAc)

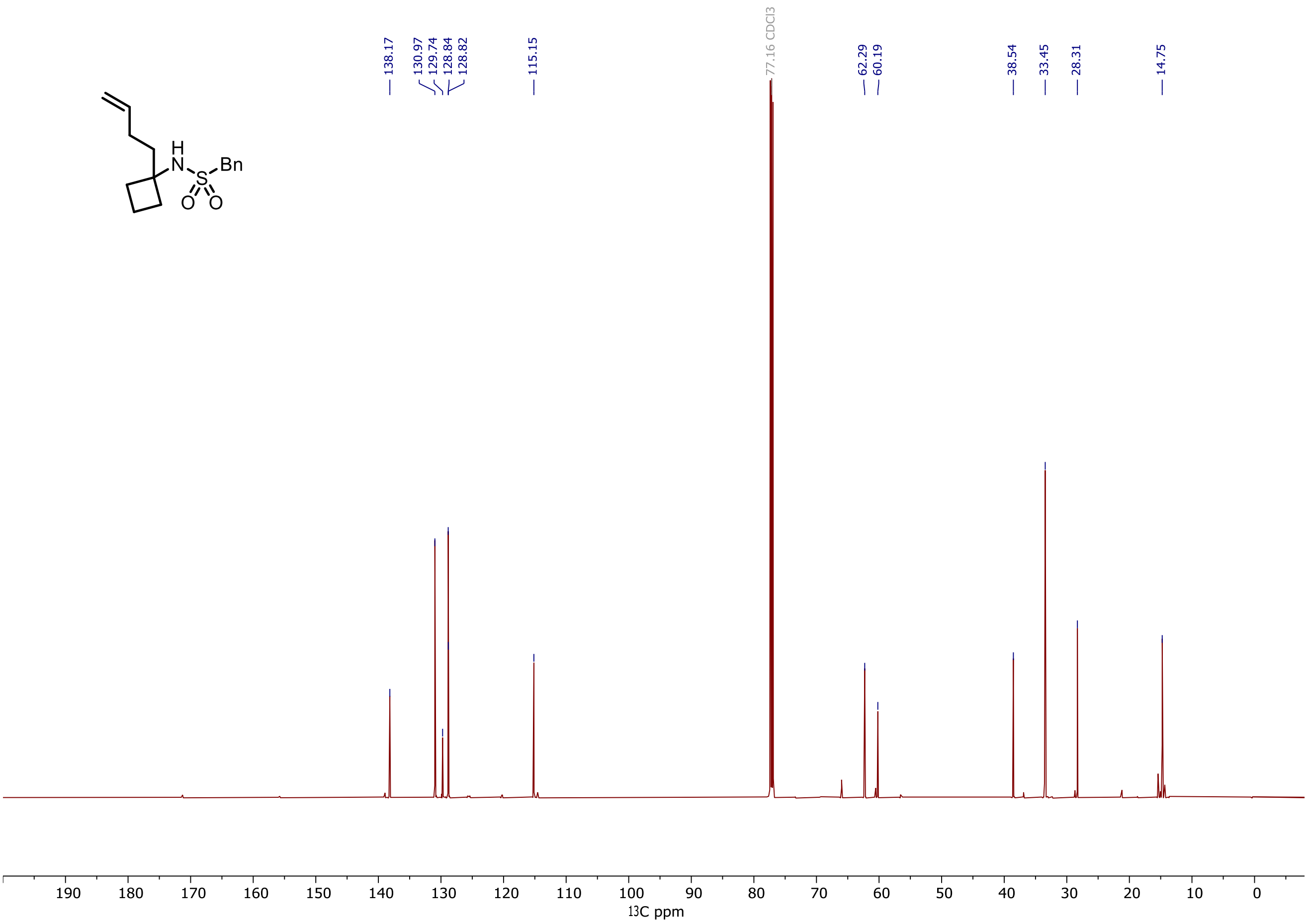

[back to table of contents]

^{1}H NMR (600 MHz, $CDCl_3$) of compound **7v**

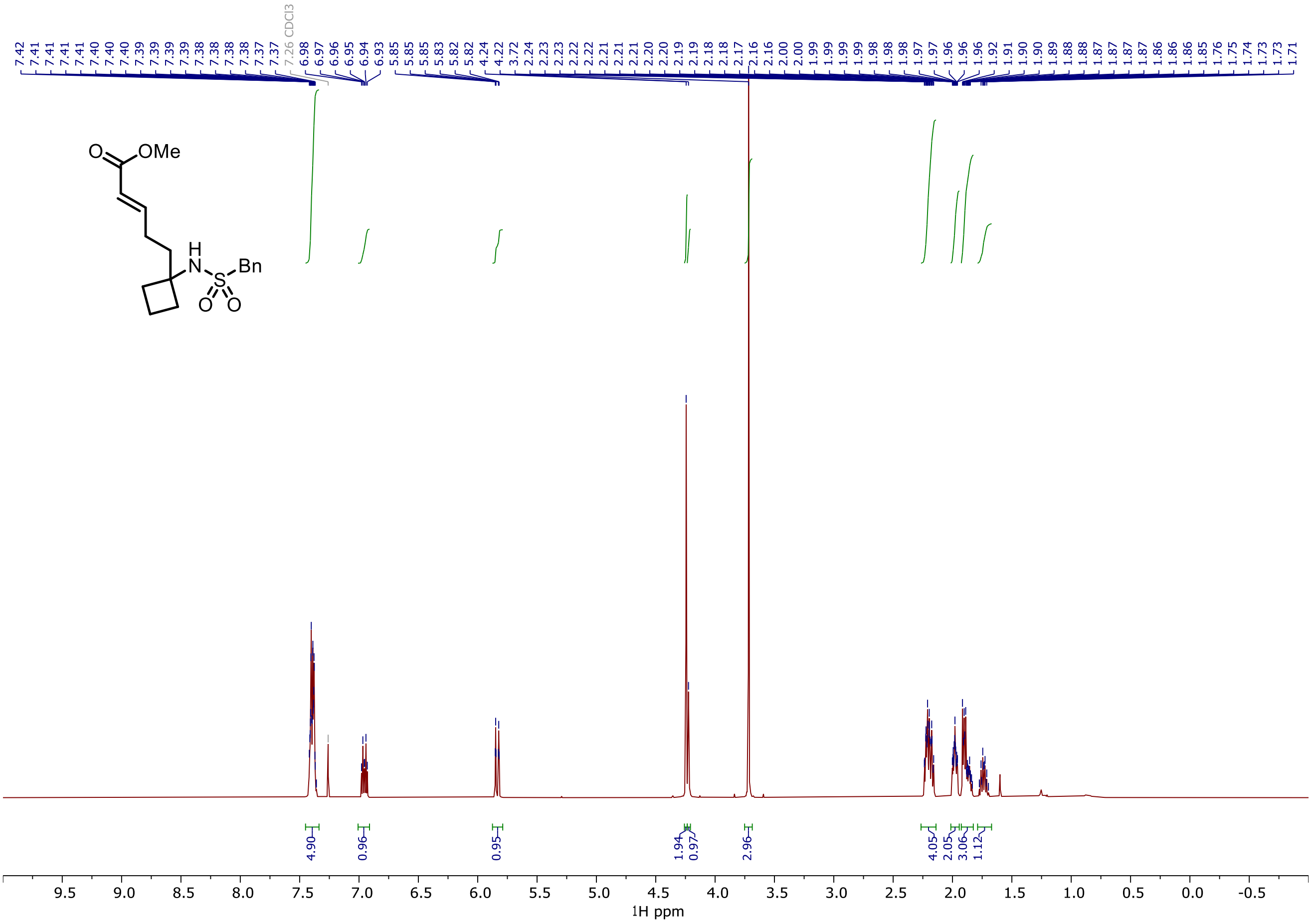

^{13}C NMR (151 MHz, $CDCl_3$) of compound **7v**

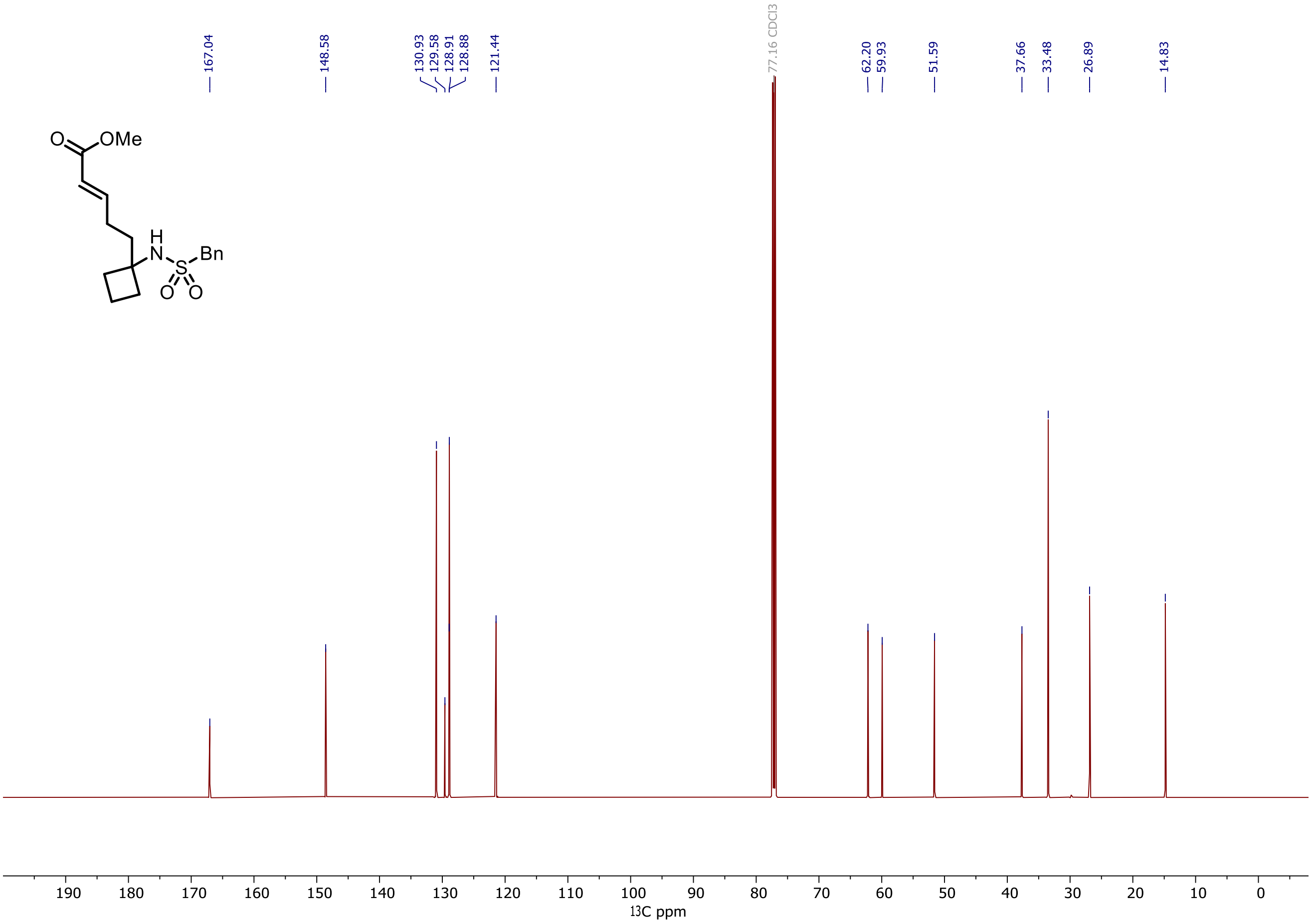

[back to table of contents]

^{1}H NMR (600 MHz, $CDCl_3$) of compound **S32**

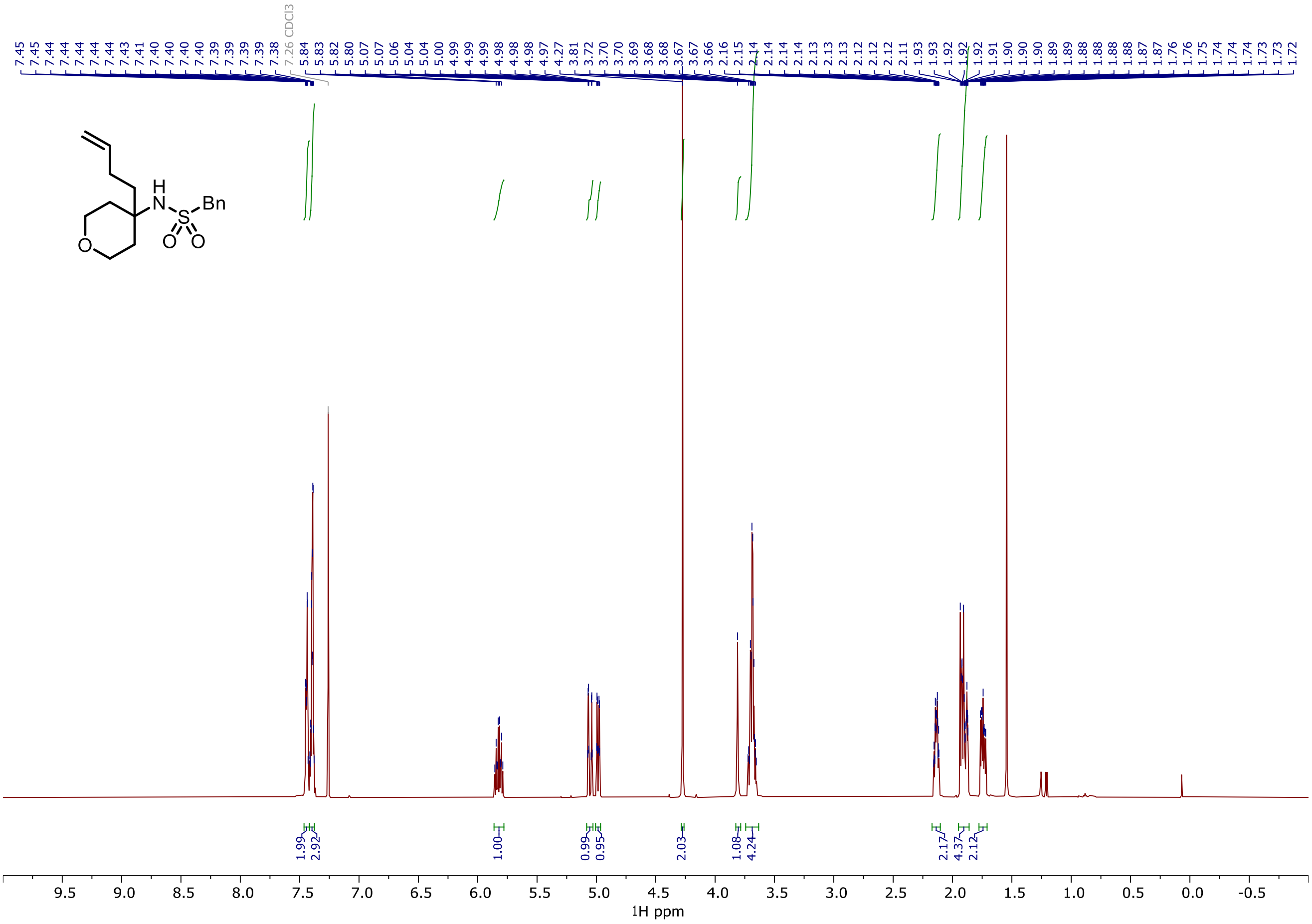

^{13}C NMR (151 MHz, $CDCl_3$) of compound **S32**

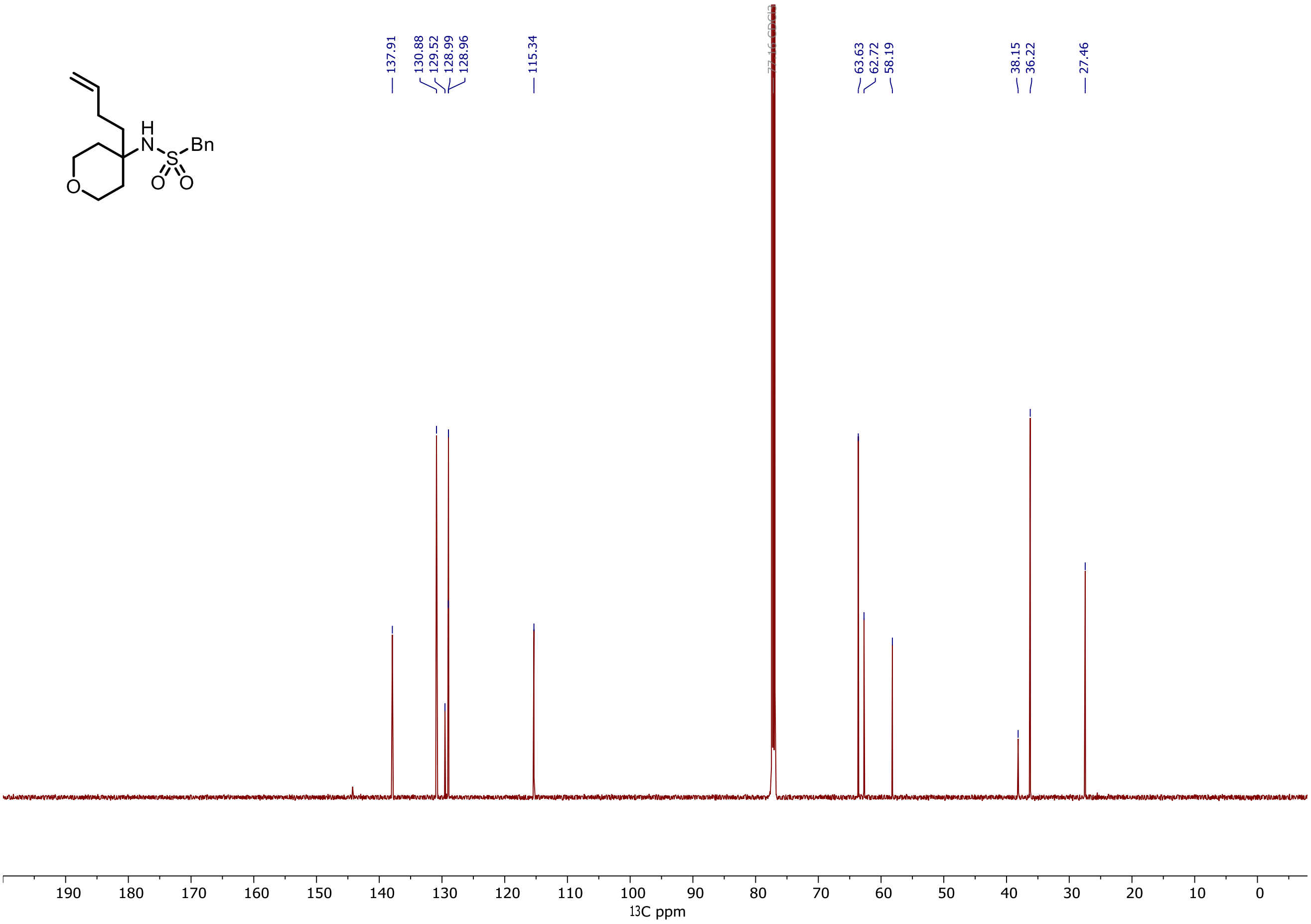

[back to table of contents]

^{1}H NMR (600 MHz, CDCl$_3$) of compound 7w

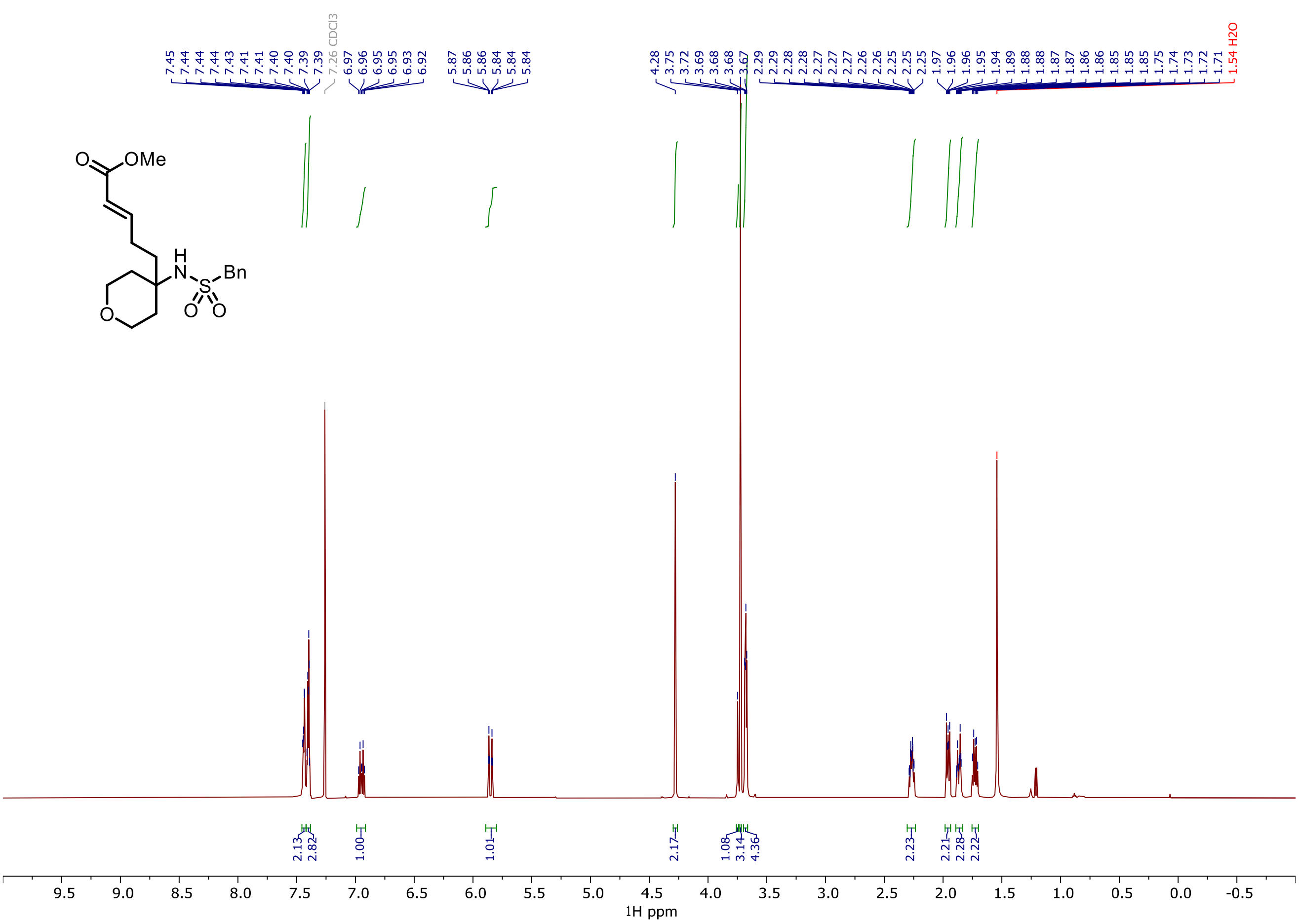

^{13}C NMR (151 MHz, CDCl$_3$) of compound 7w

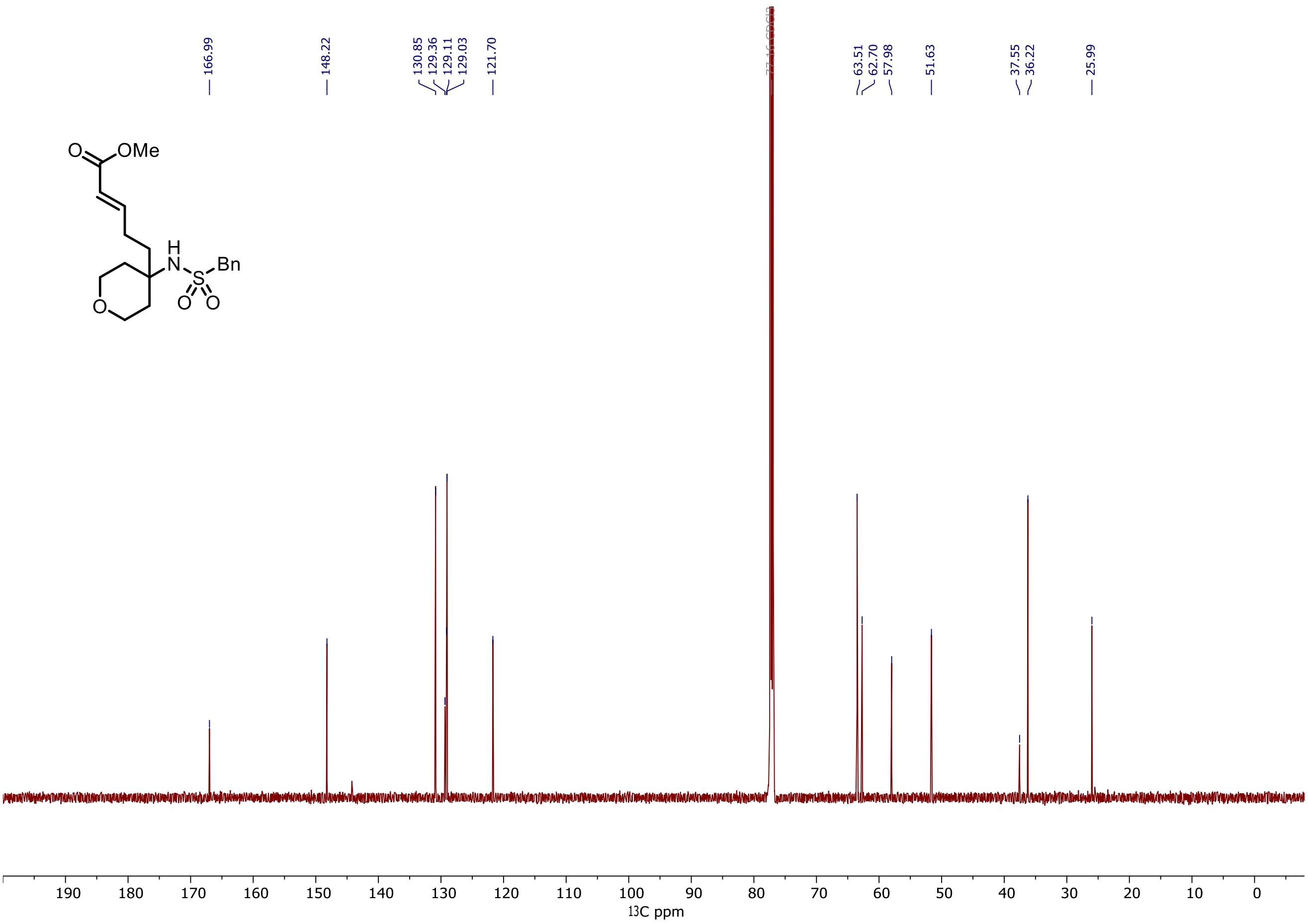

[back to table of contents]

^{1}H NMR (600 MHz, $CDCl_3$) of compound S34

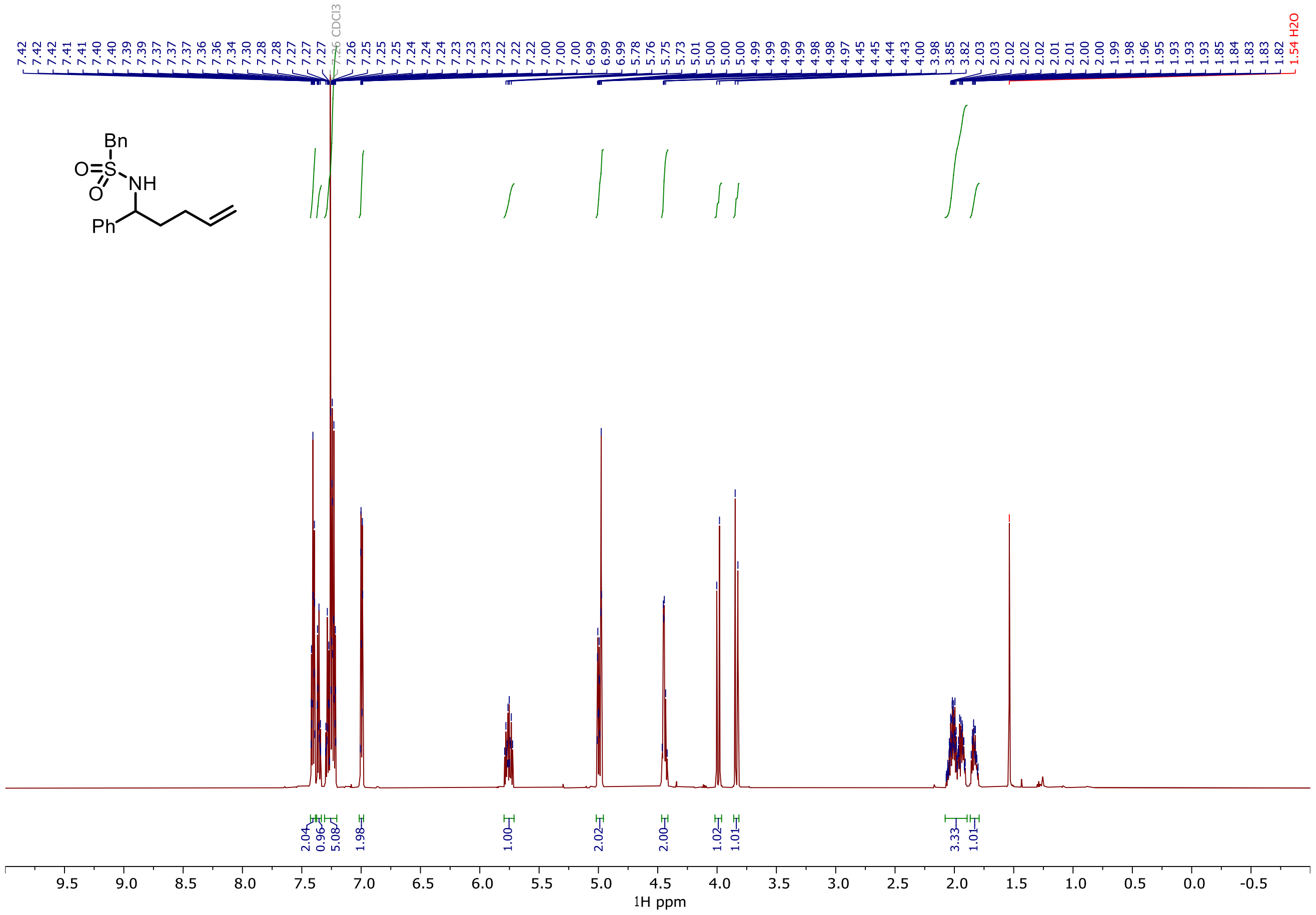

^{13}C NMR (151 MHz, $CDCl_3$) of compound S34

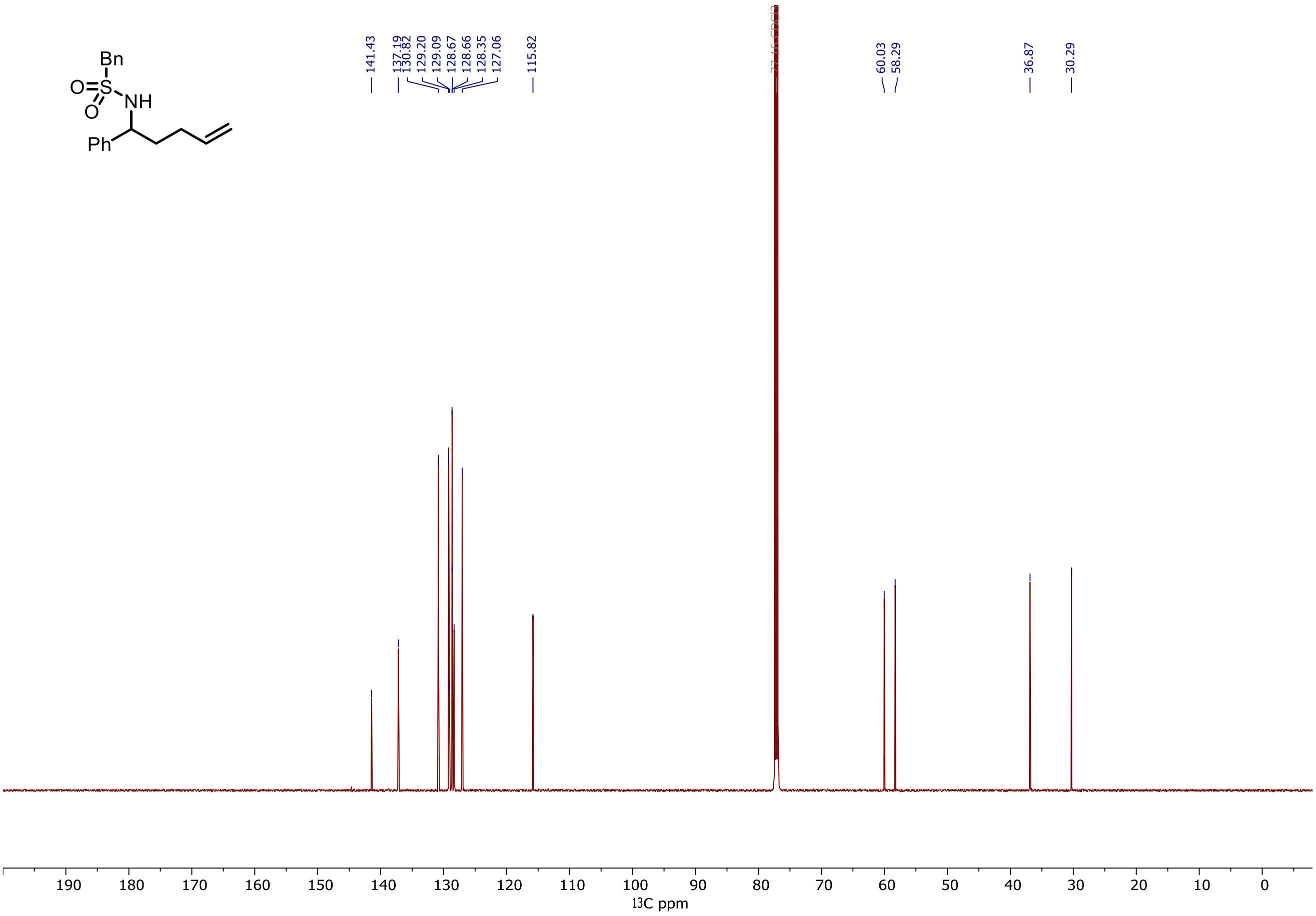

[back to table of contents]

^{1}**H NMR** (600 MHz, $CDCl_3$) of compound **7x**

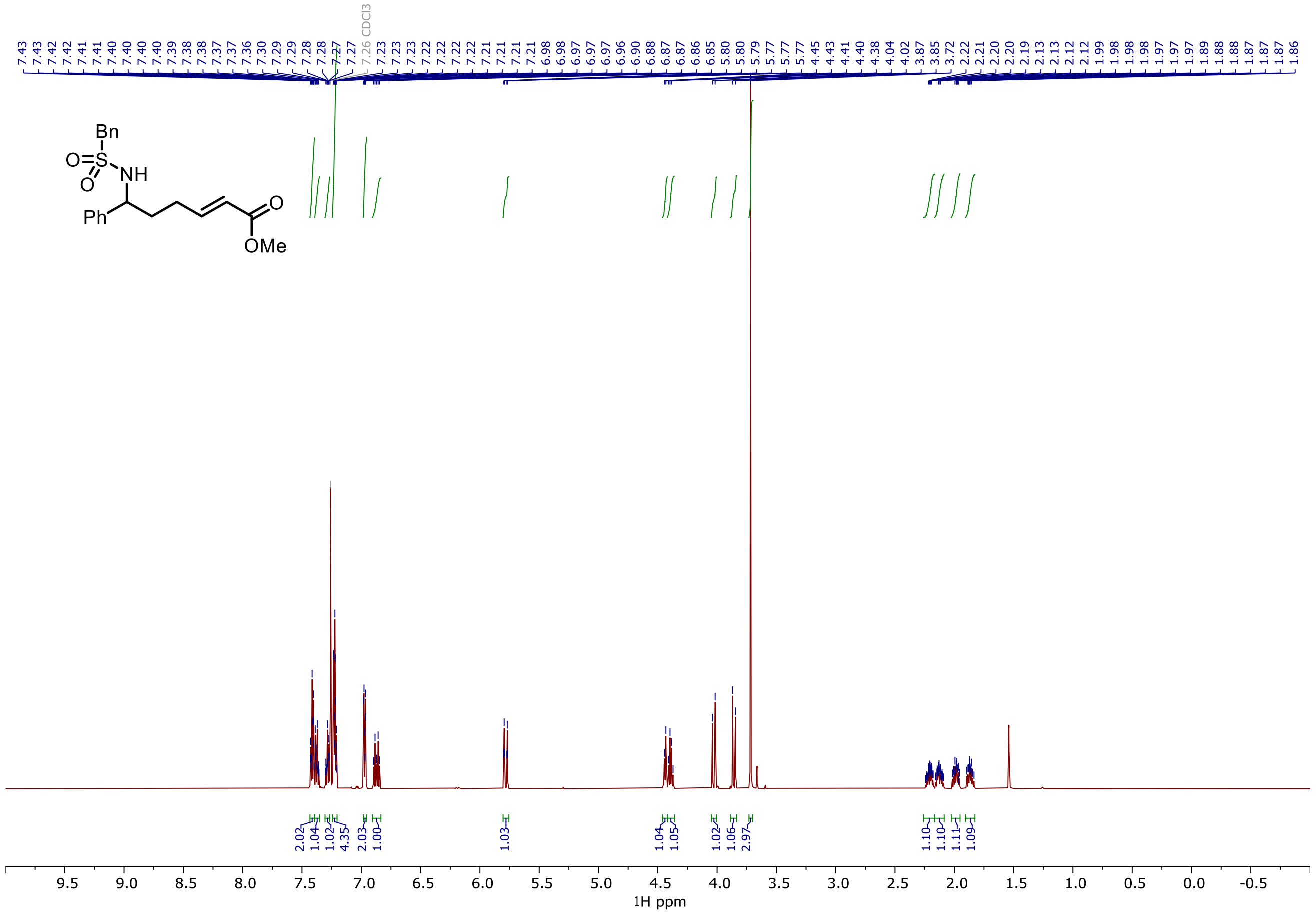

^{13}**C NMR** (151 MHz, $CDCl_3$) of compound **7x**

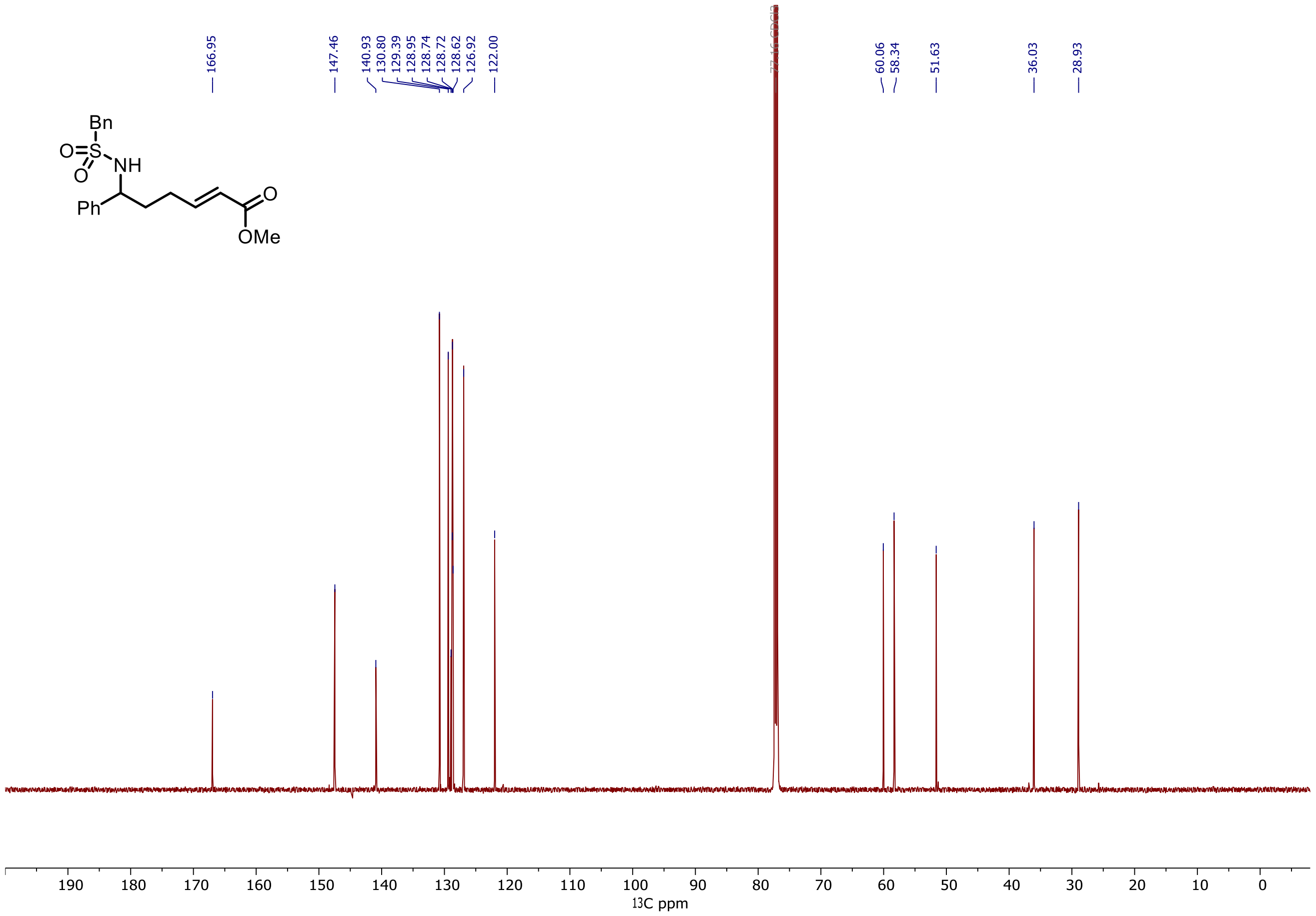

[back to table of contents]

^{1}H NMR (400 MHz, $CDCl_3$) of compound **S36**

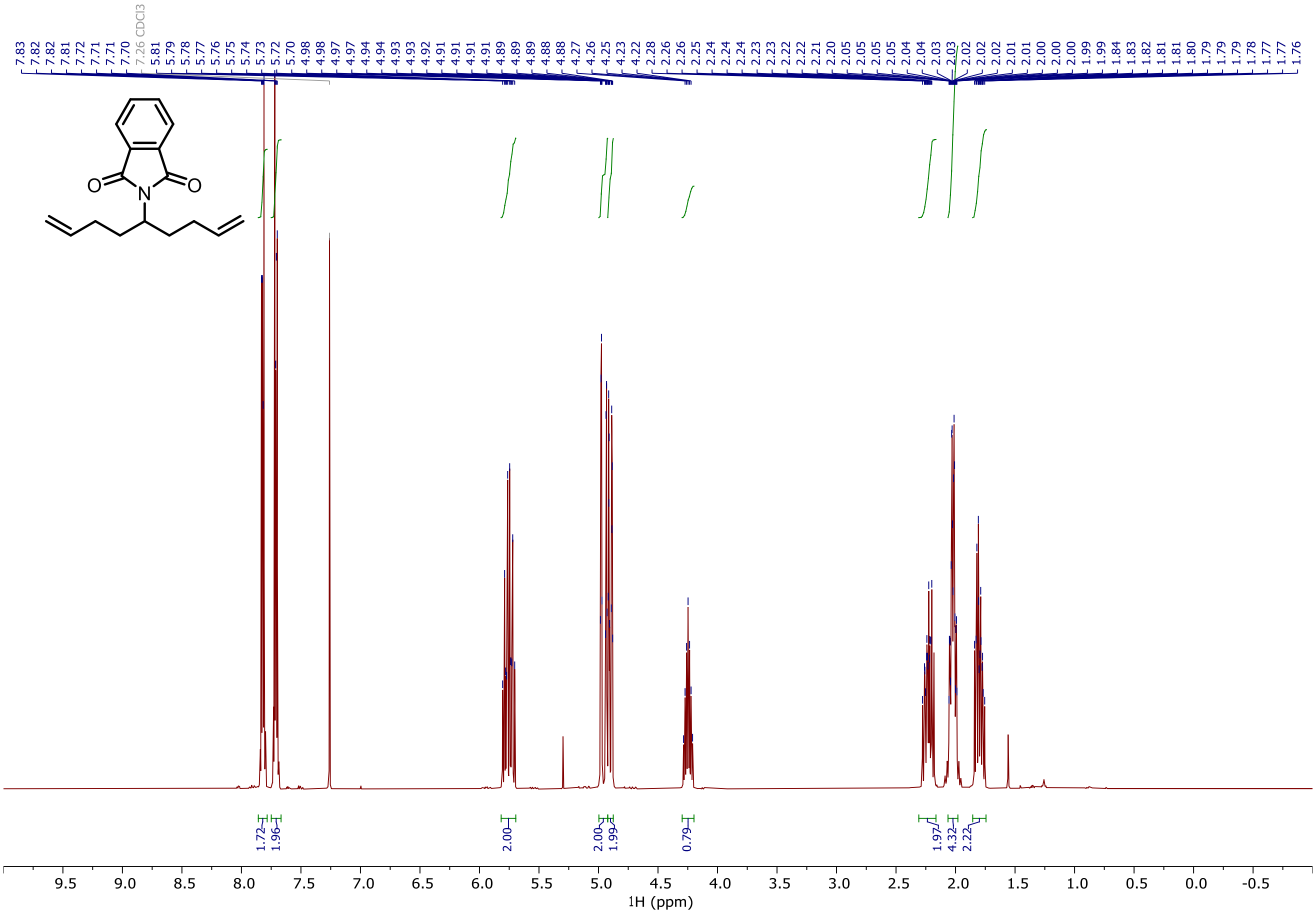

^{13}C NMR (101 MHz, $CDCl_3$) of compound **S36**

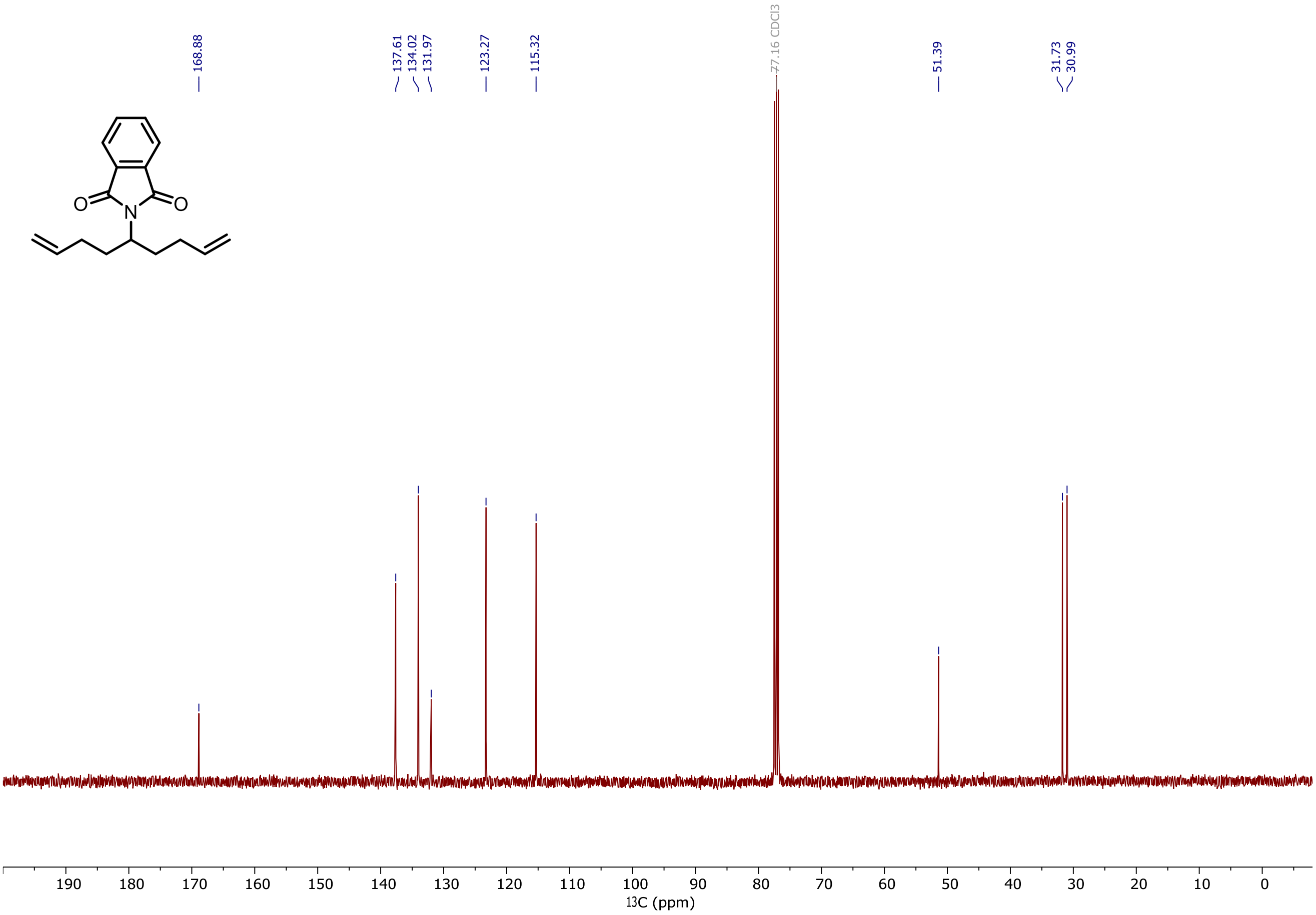

[back to table of contents]

^{1}H NMR (600 MHz, $CDCl_3$) of compound **S37**

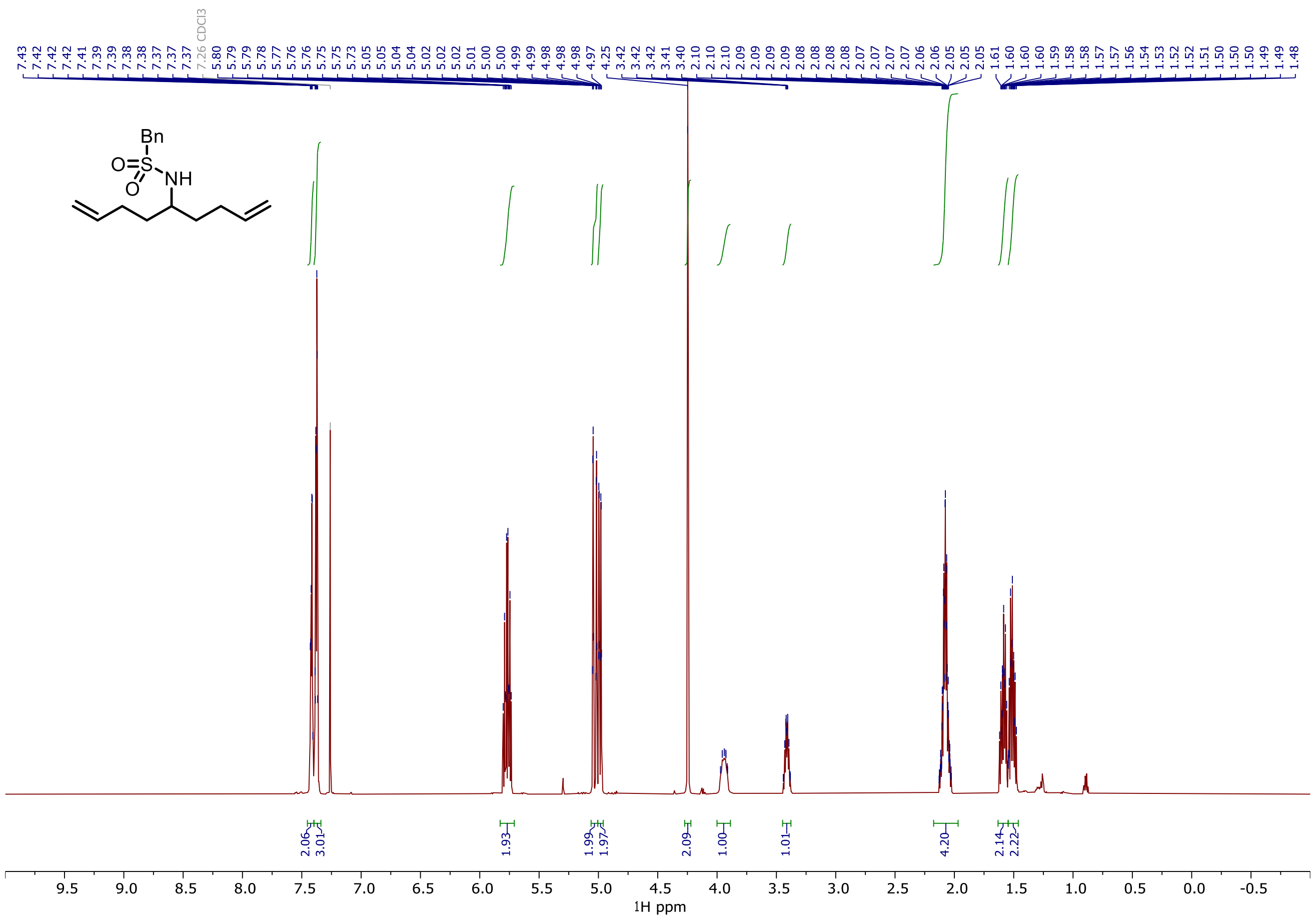

^{13}C NMR (151 MHz, $CDCl_3$) of compound **S37**

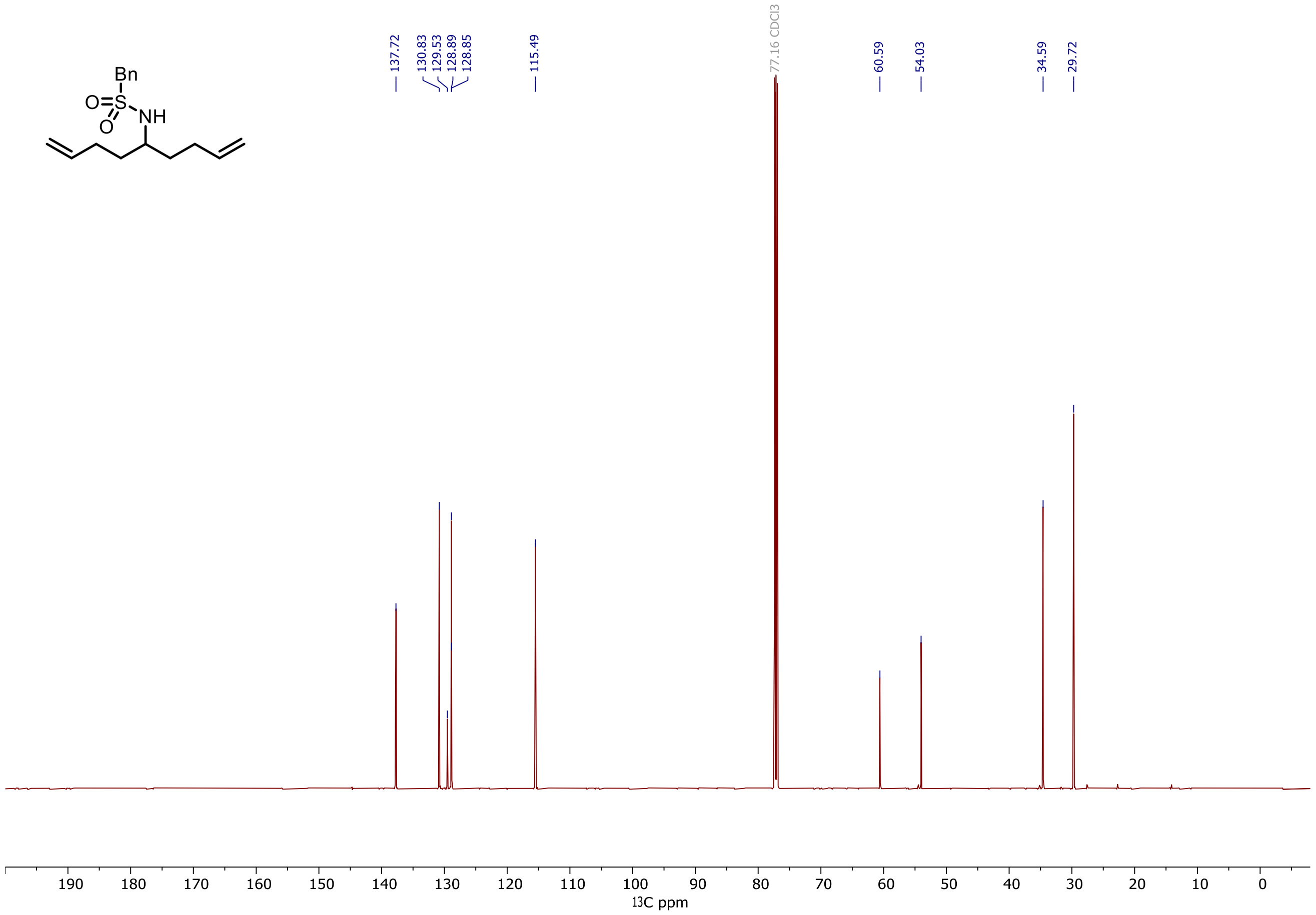

[back to table of contents]

¹H NMR (400 MHz, $CDCl_3$) of compound **7y**

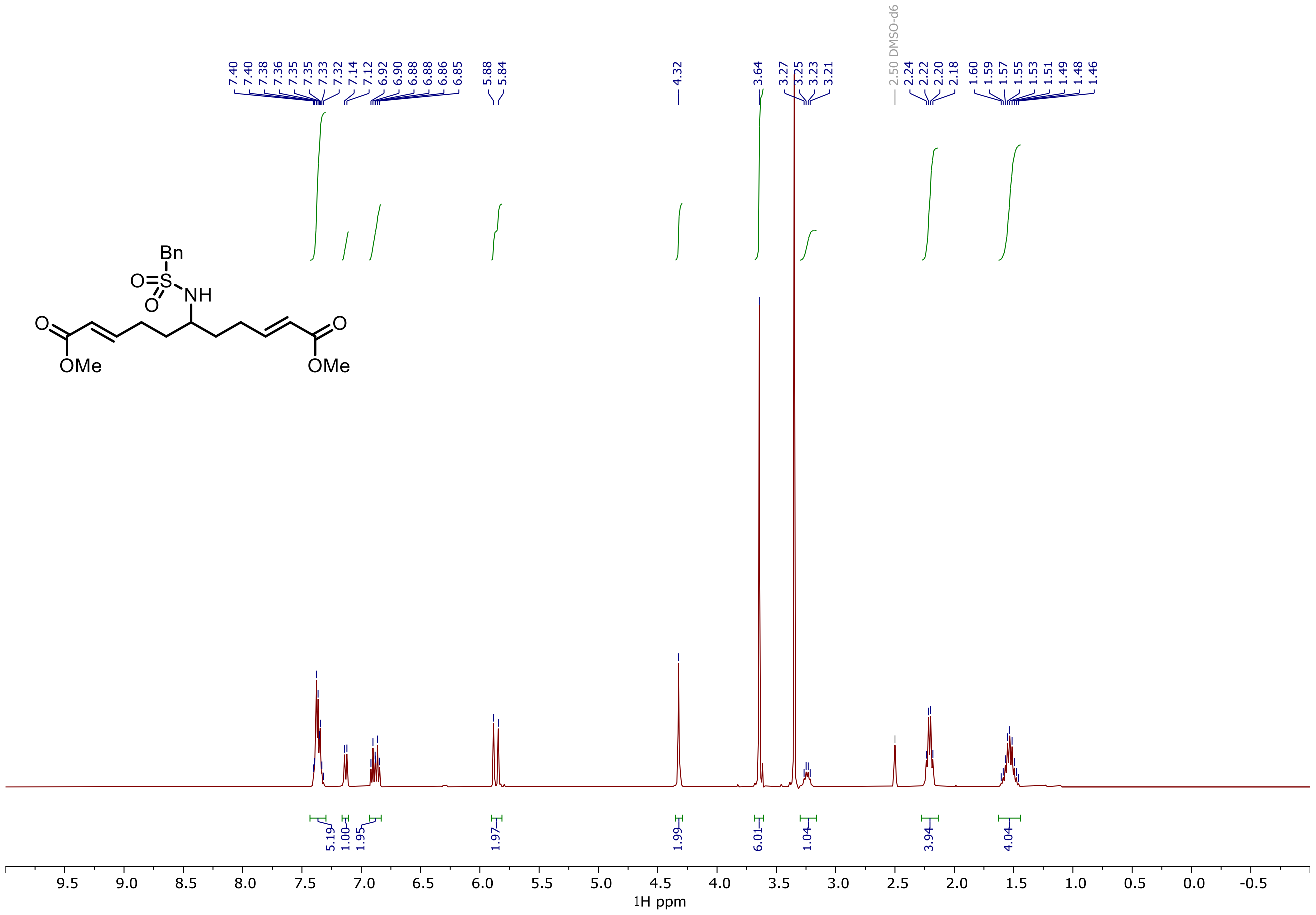

¹³C NMR (101 MHz, $CDCl_3$) of compound **7y**

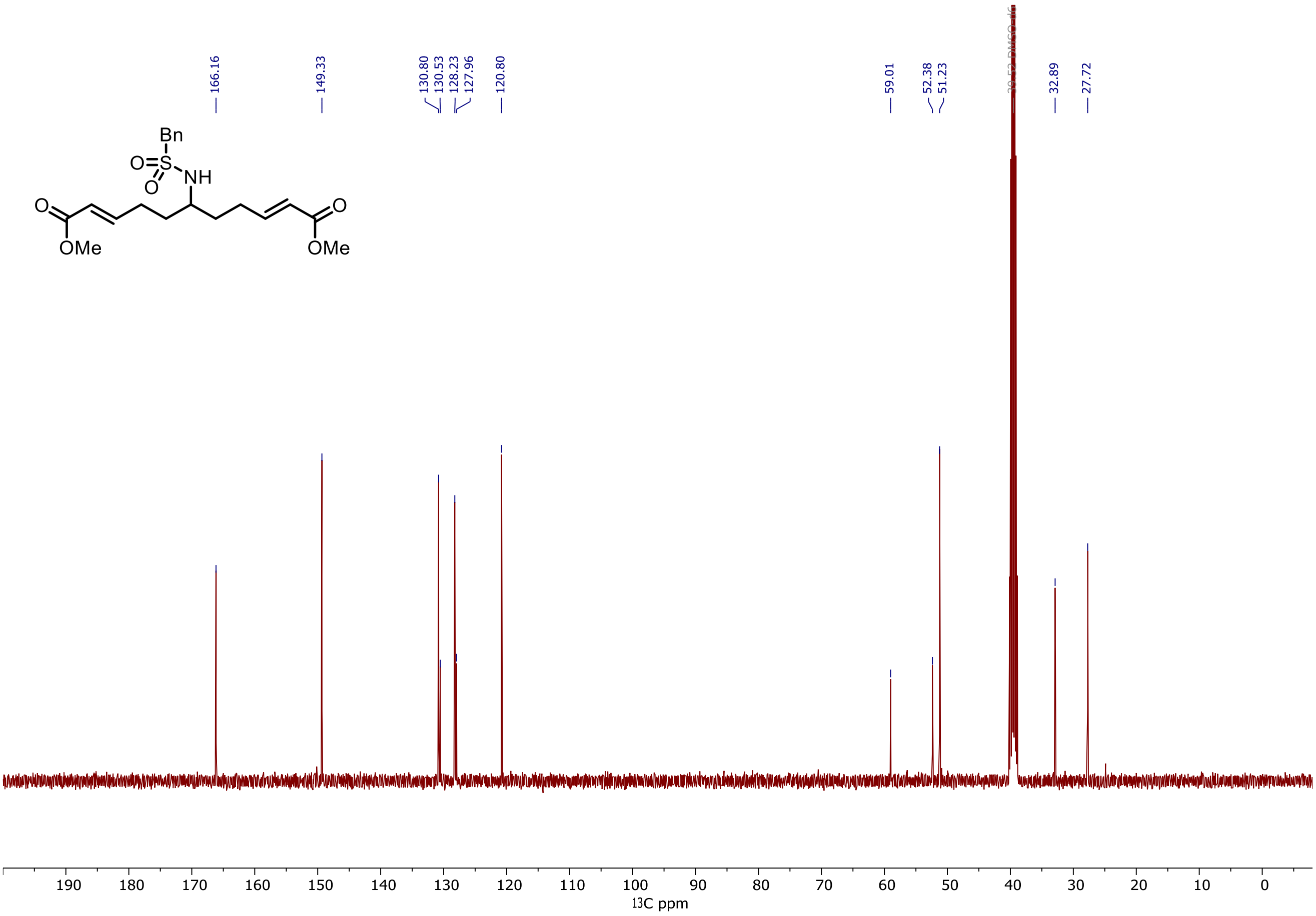

[back to table of contents]

^{1}H NMR (600 MHz, $CDCl_3$) of compound **S39**

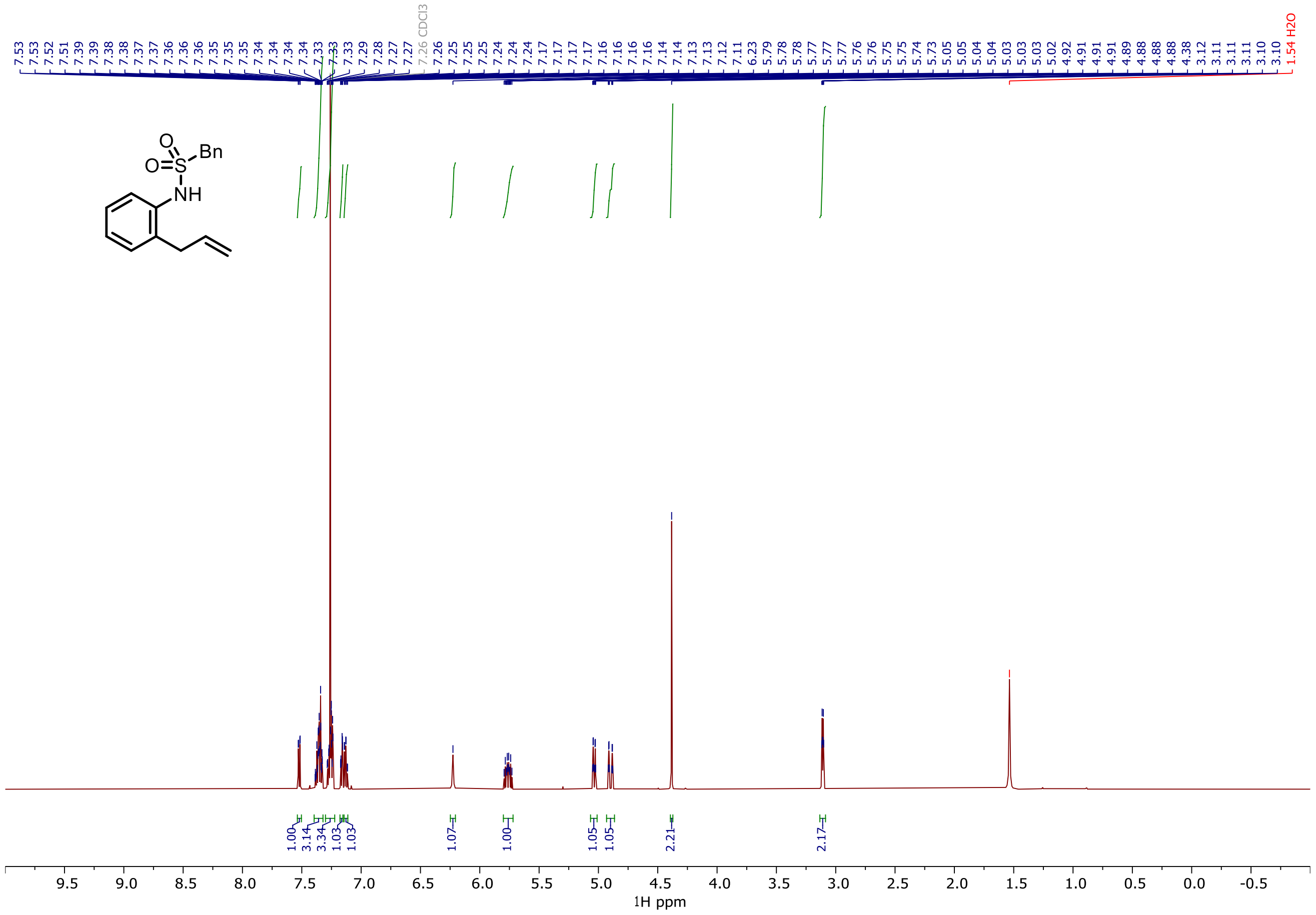

^{13}C NMR (151 MHz, $CDCl_3$) of compound **S39**

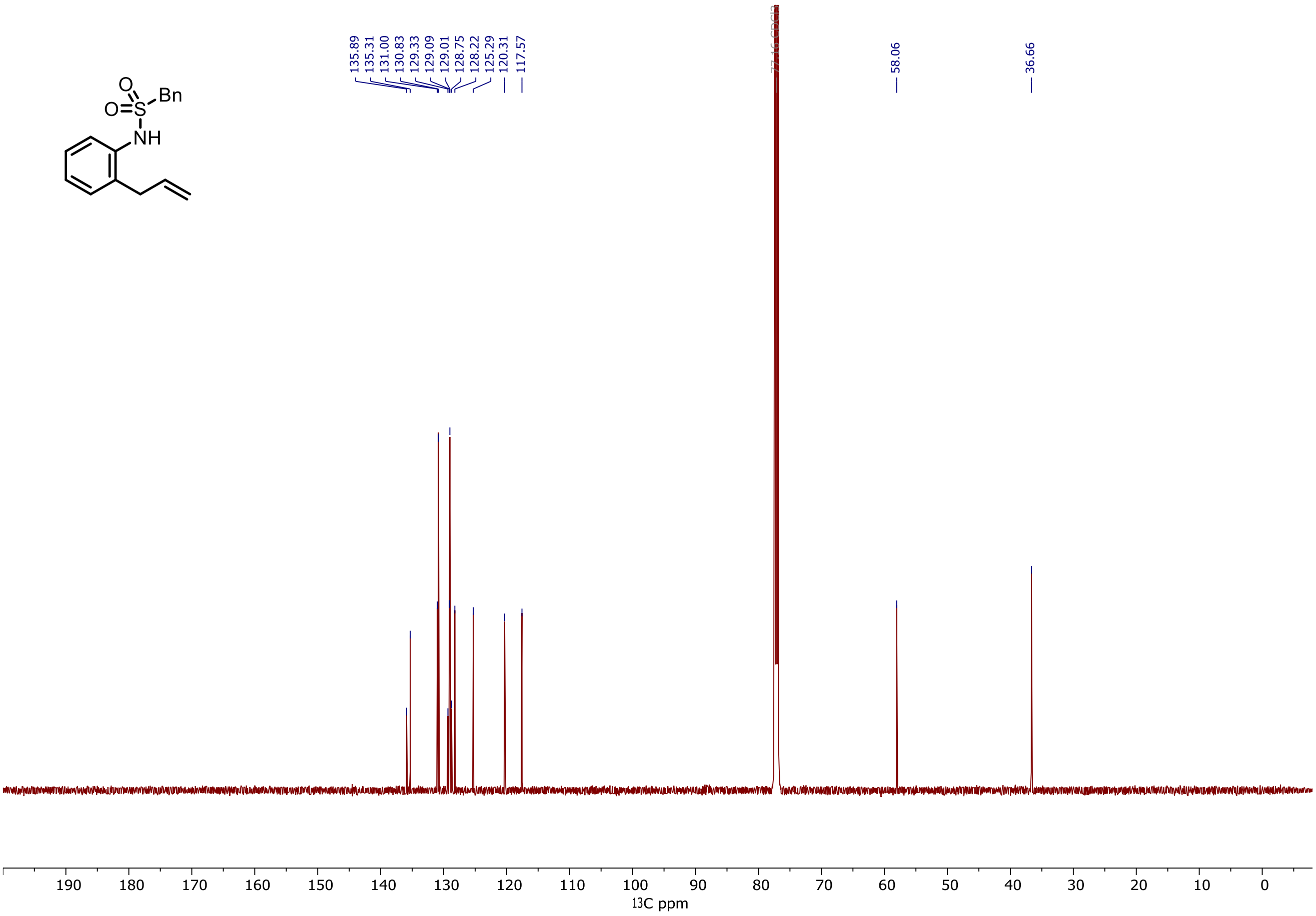

[back to table of contents]

[1]**H NMR** (400 MHz, $CDCl_3$) of compound **7z**

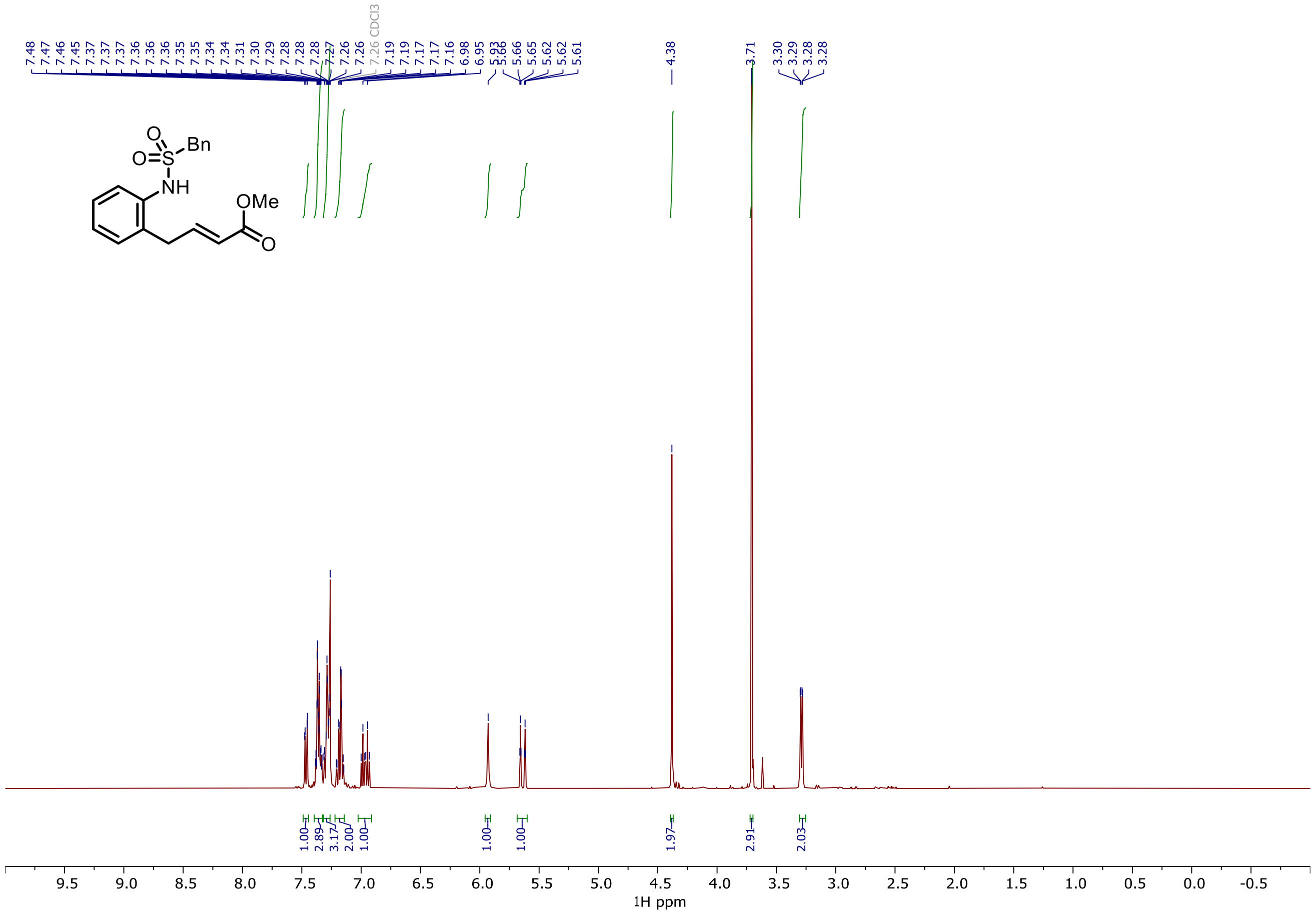

[13]**C NMR** (101 MHz, $CDCl_3$) of compound **7z**

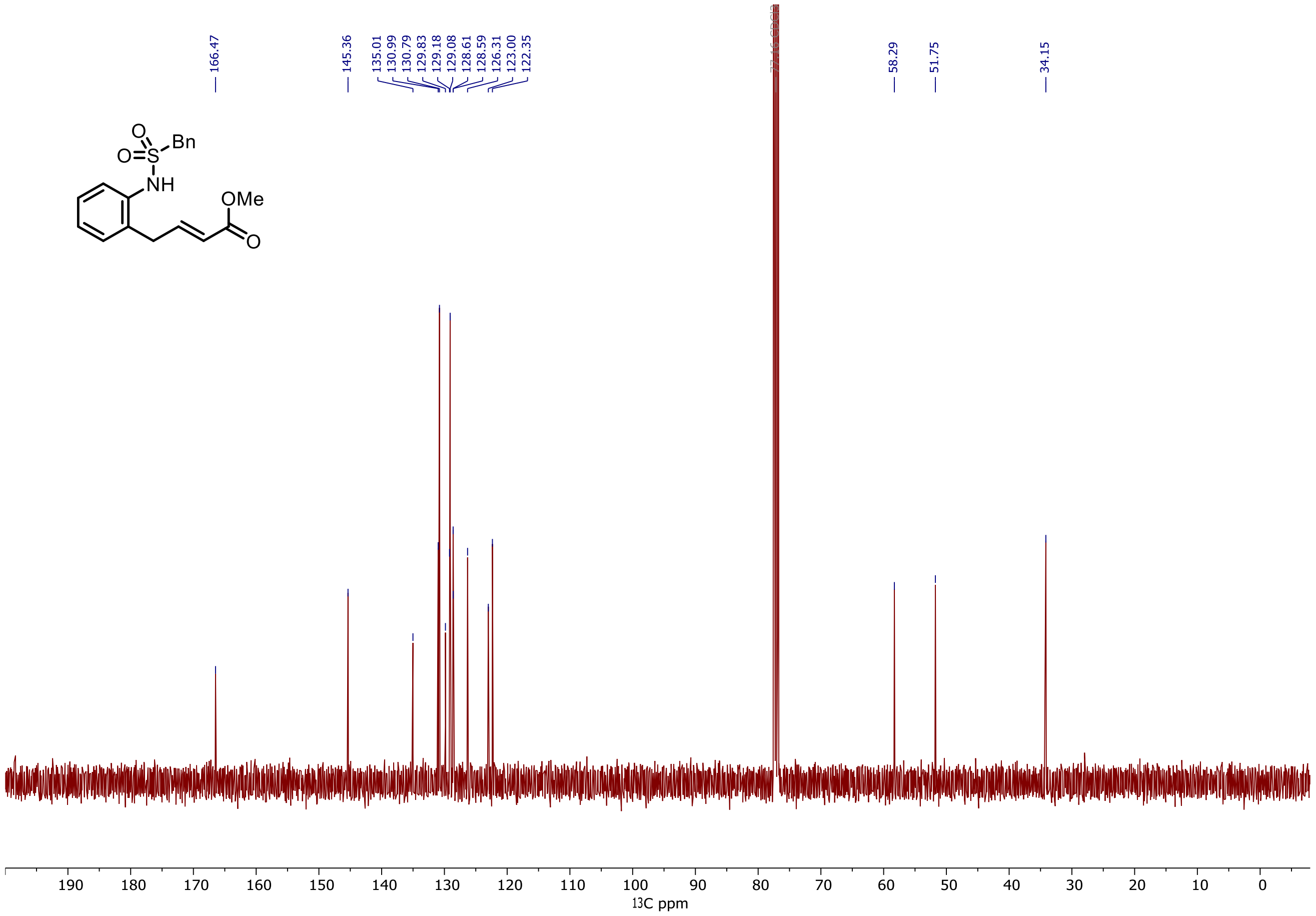

[back to table of contents]

¹H NMR (400 MHz, $CDCl_3$) of compound **S40**

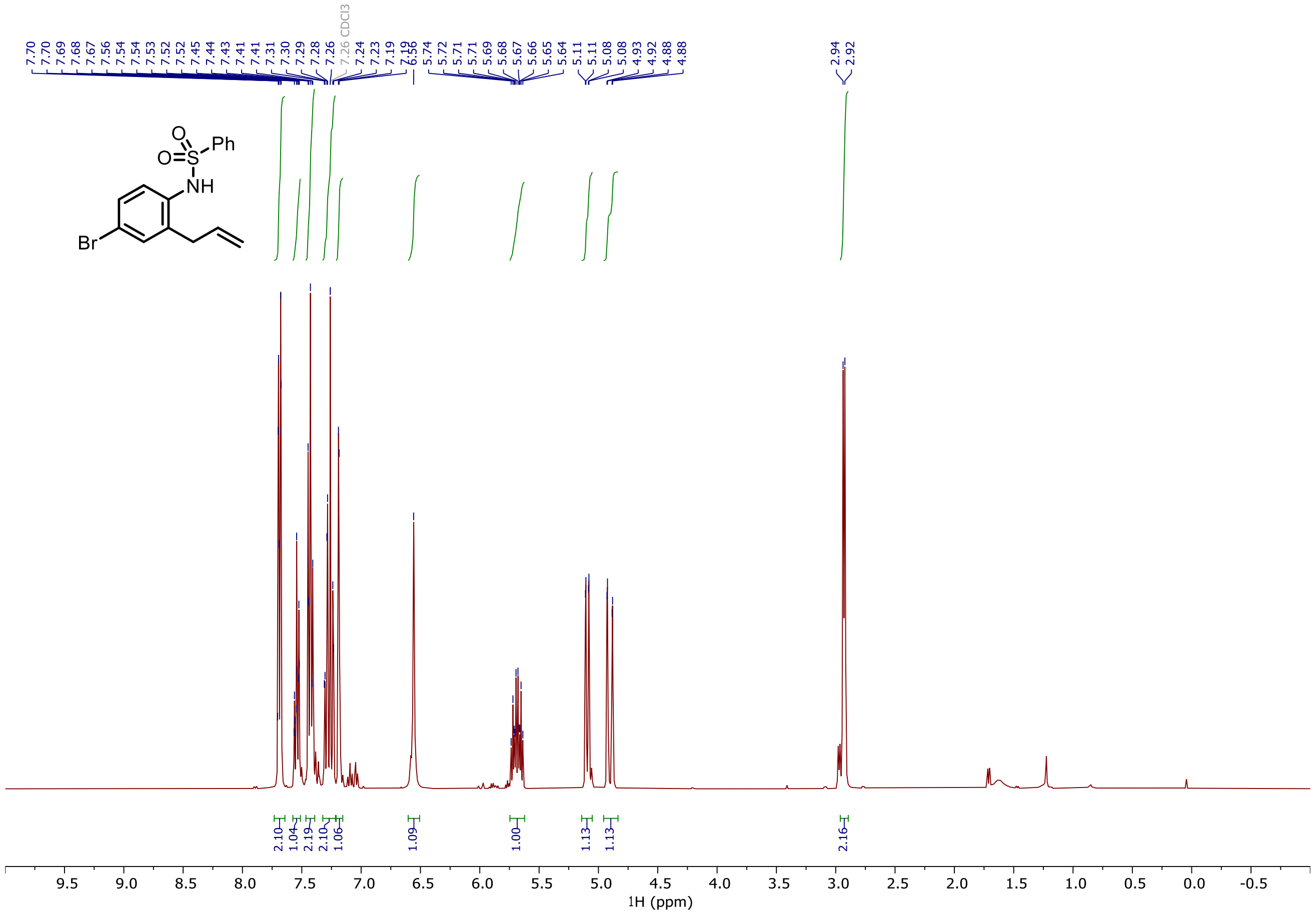

¹³C NMR (101 MHz, $CDCl_3$) of compound **S40**

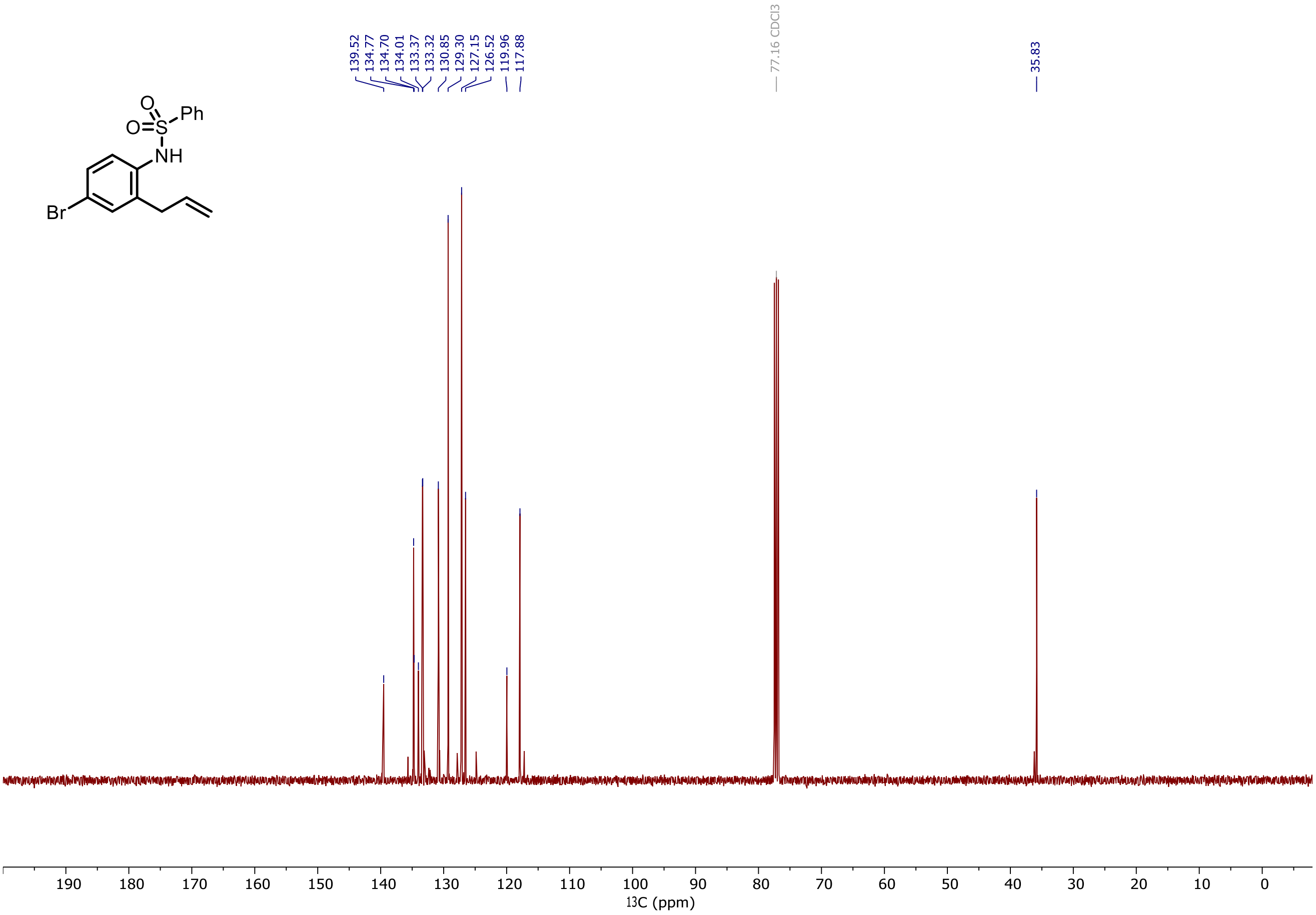

[back to table of contents]

¹H NMR (400 MHz, $CDCl_3$) of compound **7aa**

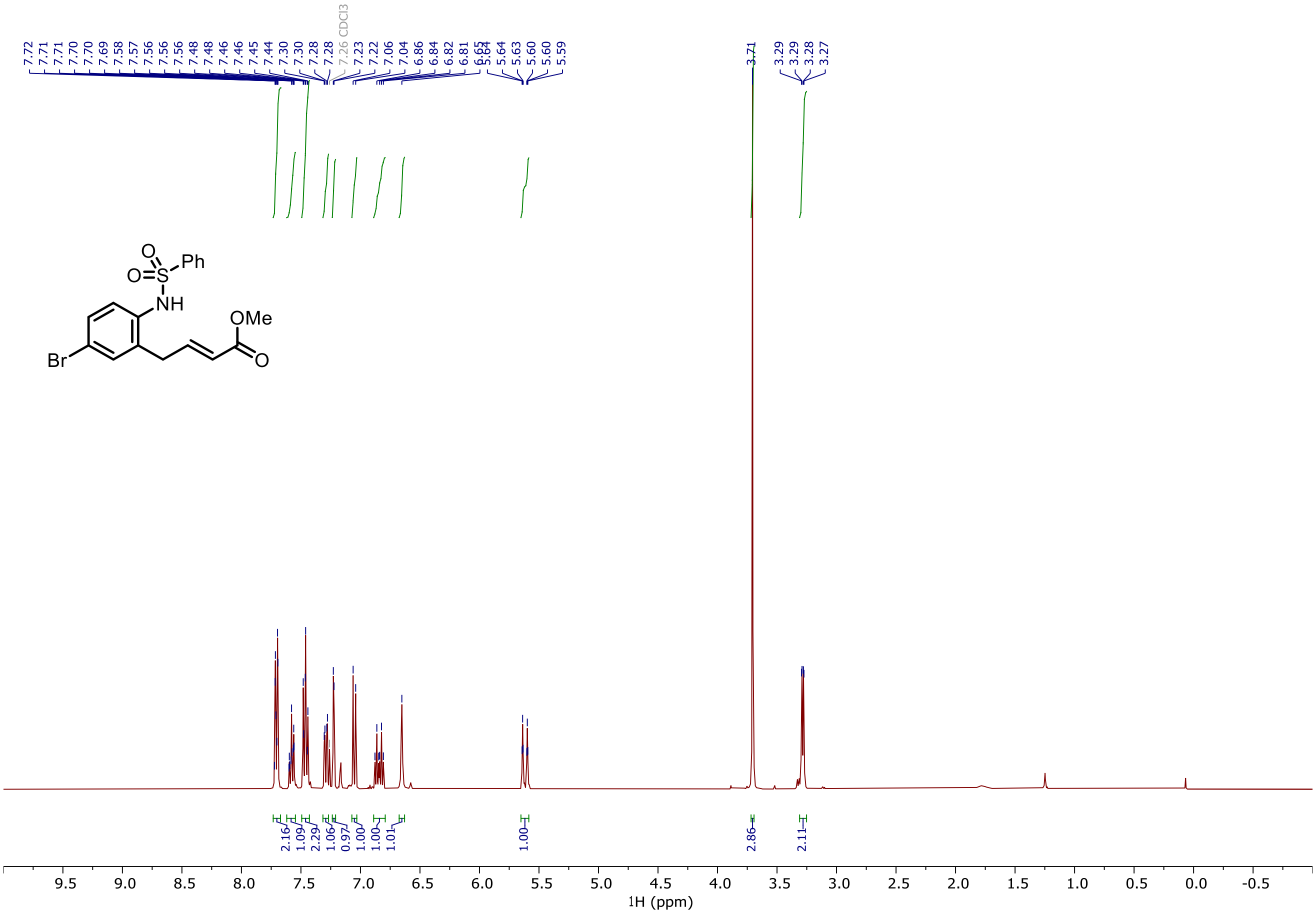

¹³C NMR (101 MHz, $CDCl_3$) of compound **7aa**

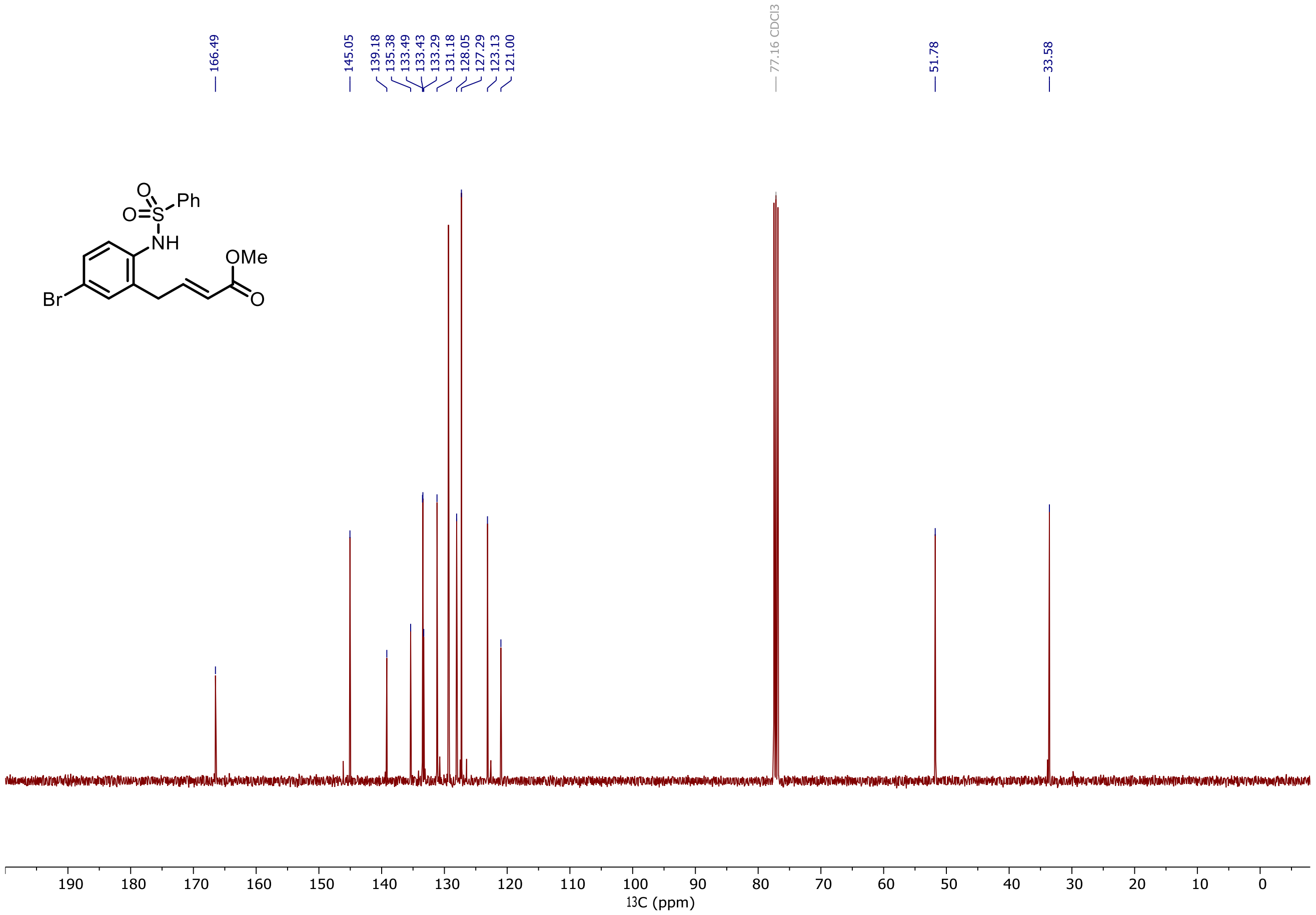

[back to table of contents]

^{1}H NMR (400 MHz, $CDCl_3$) of compound **S41**

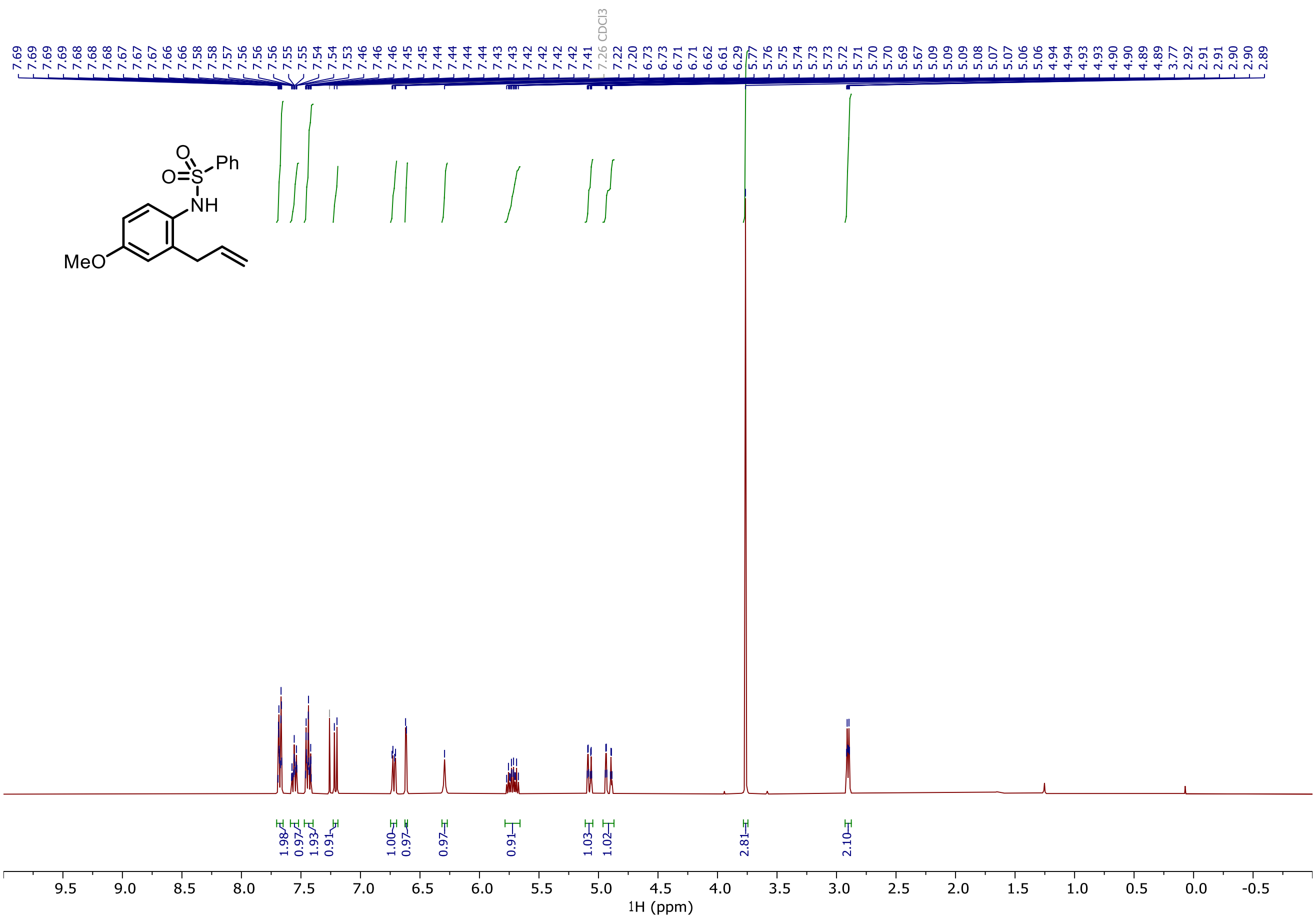

^{13}C NMR (101 MHz, $CDCl_3$) of compound **S41**

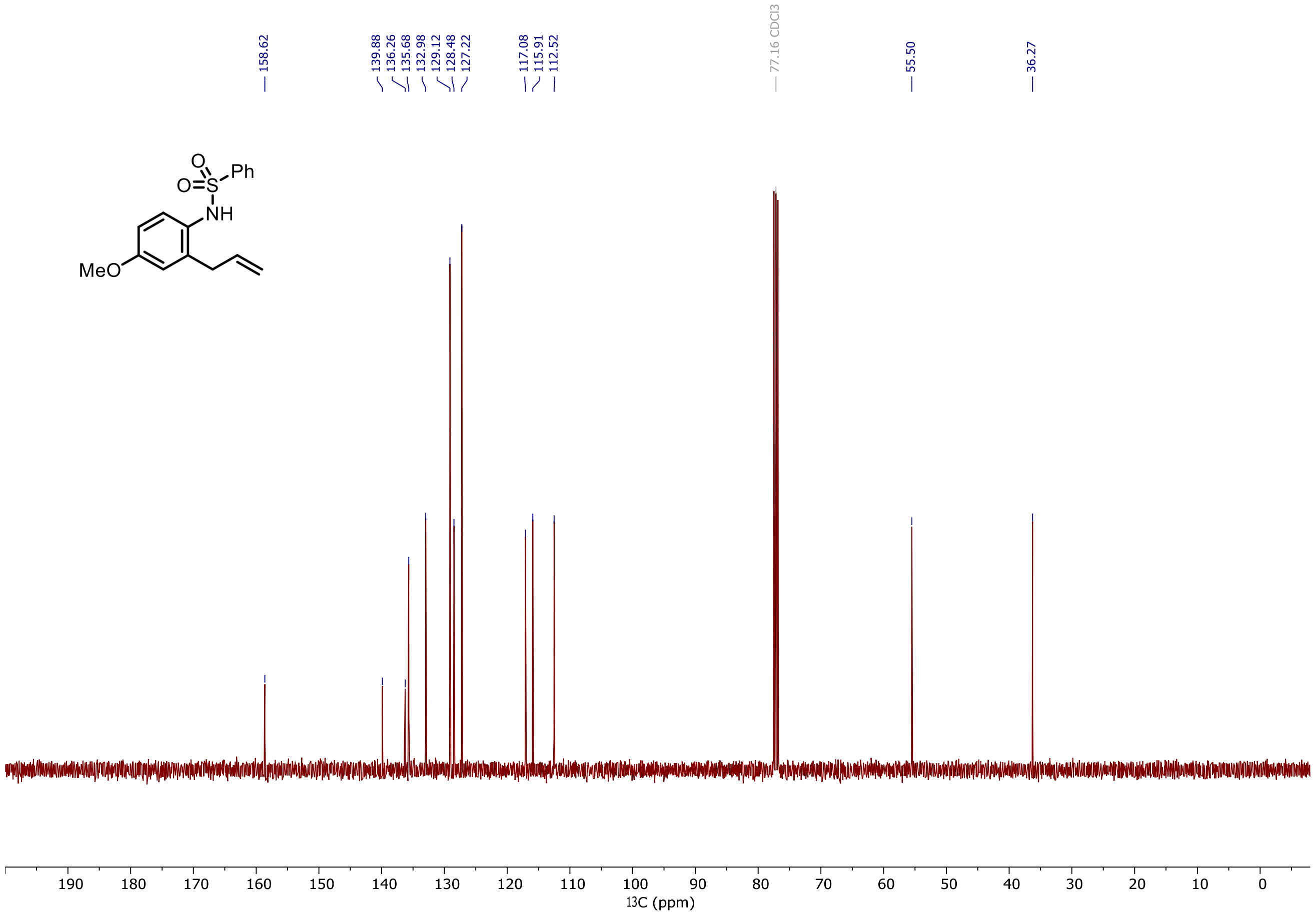

[back to table of contents]

^{1}H NMR (400 MHz, $CDCl_3$) of compound **7ab**

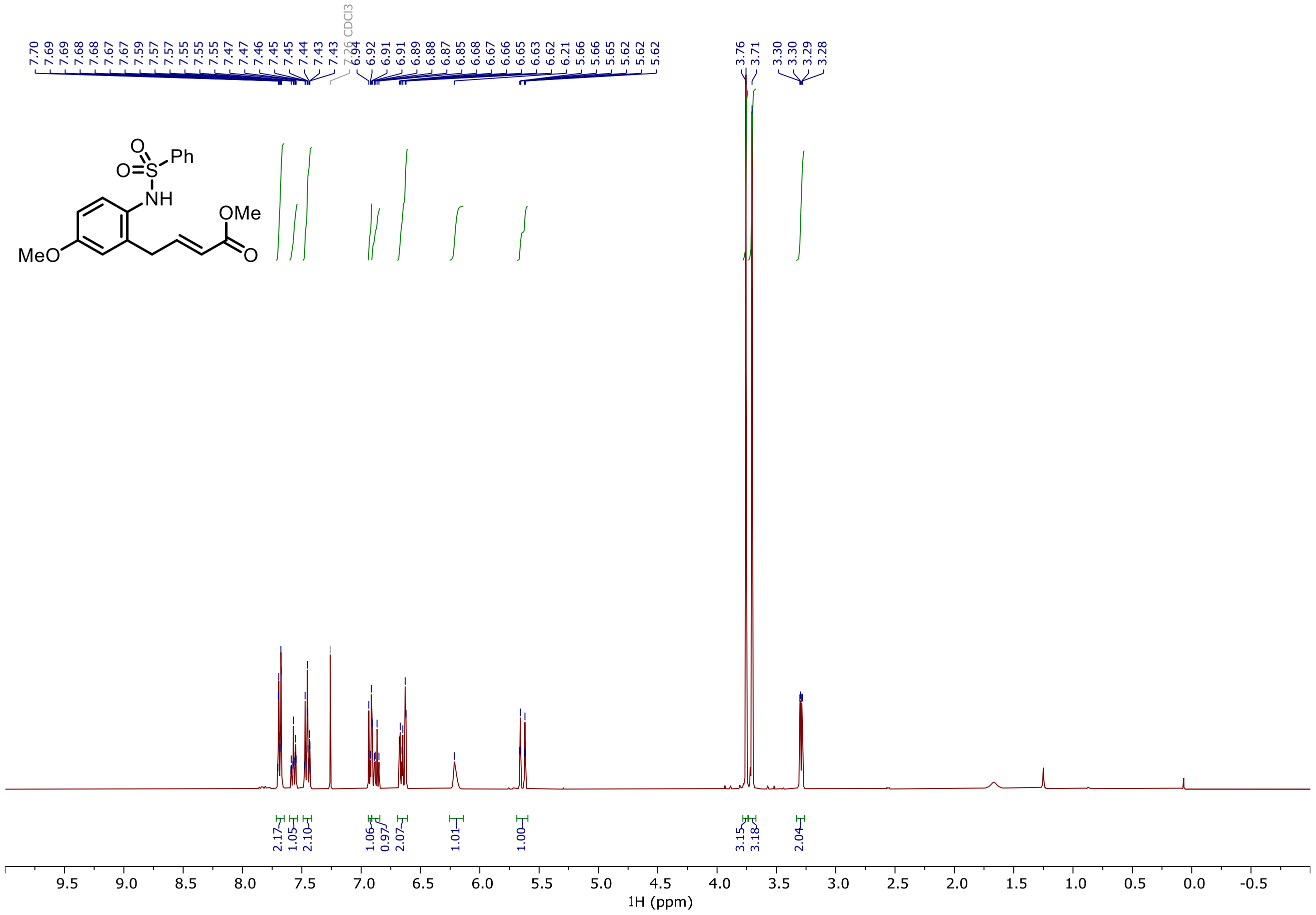

^{13}C NMR (101 MHz, $CDCl_3$) of compound **7ab**

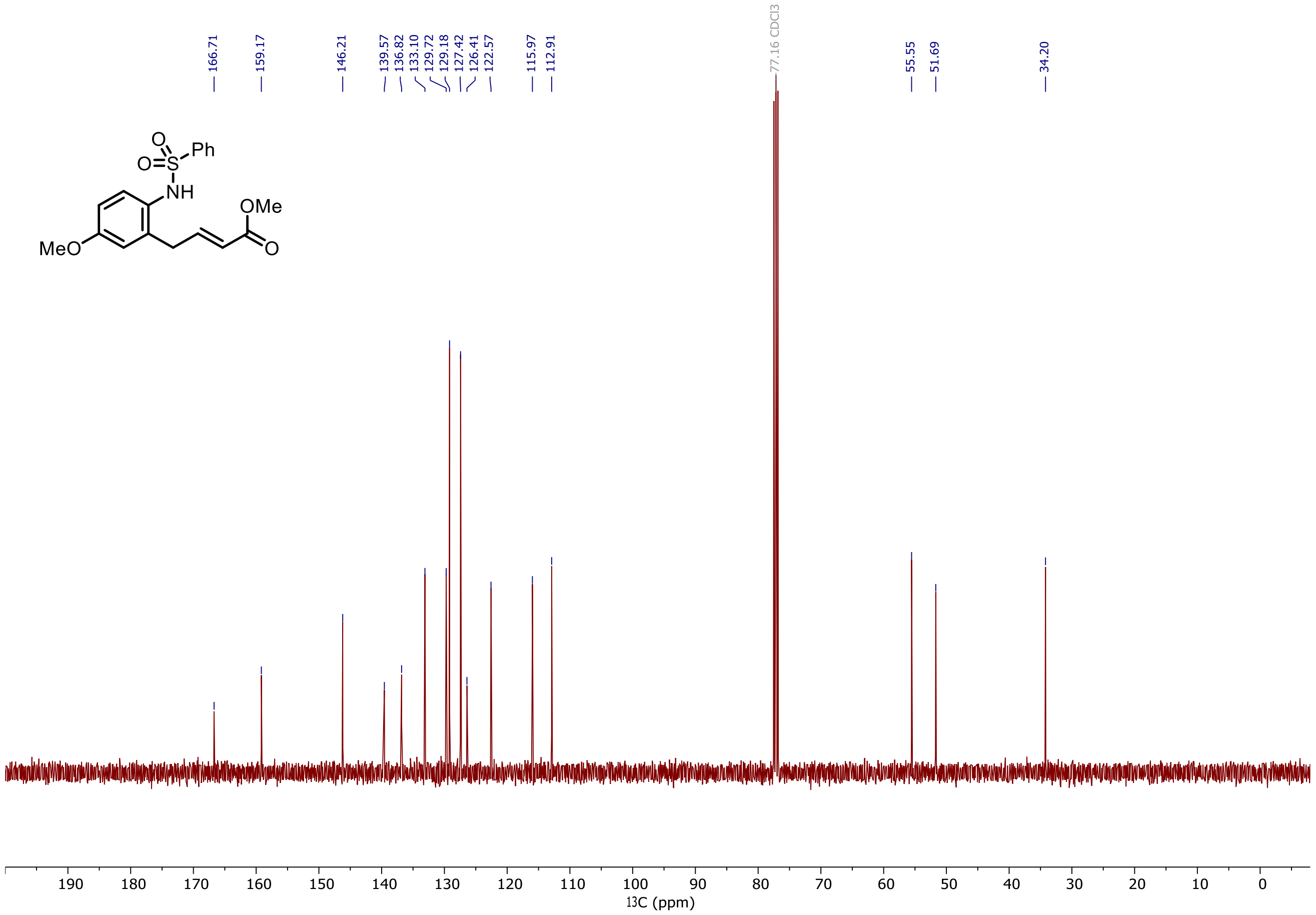

[back to table of contents]

^{1}H NMR (400 MHz, $CDCl_3$) of compound **S42**

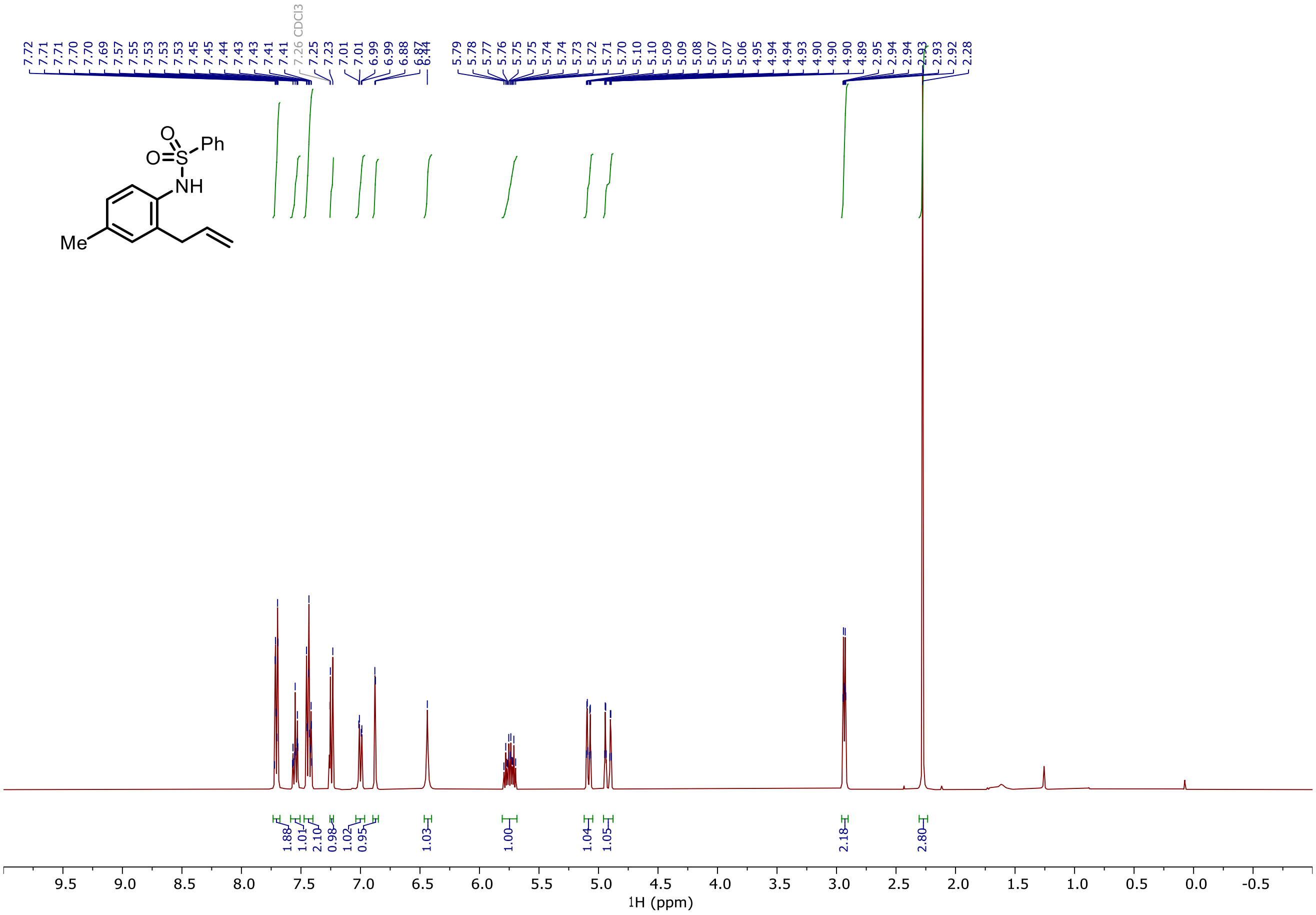

^{13}C NMR (101 MHz, $CDCl_3$) of compound **S42**

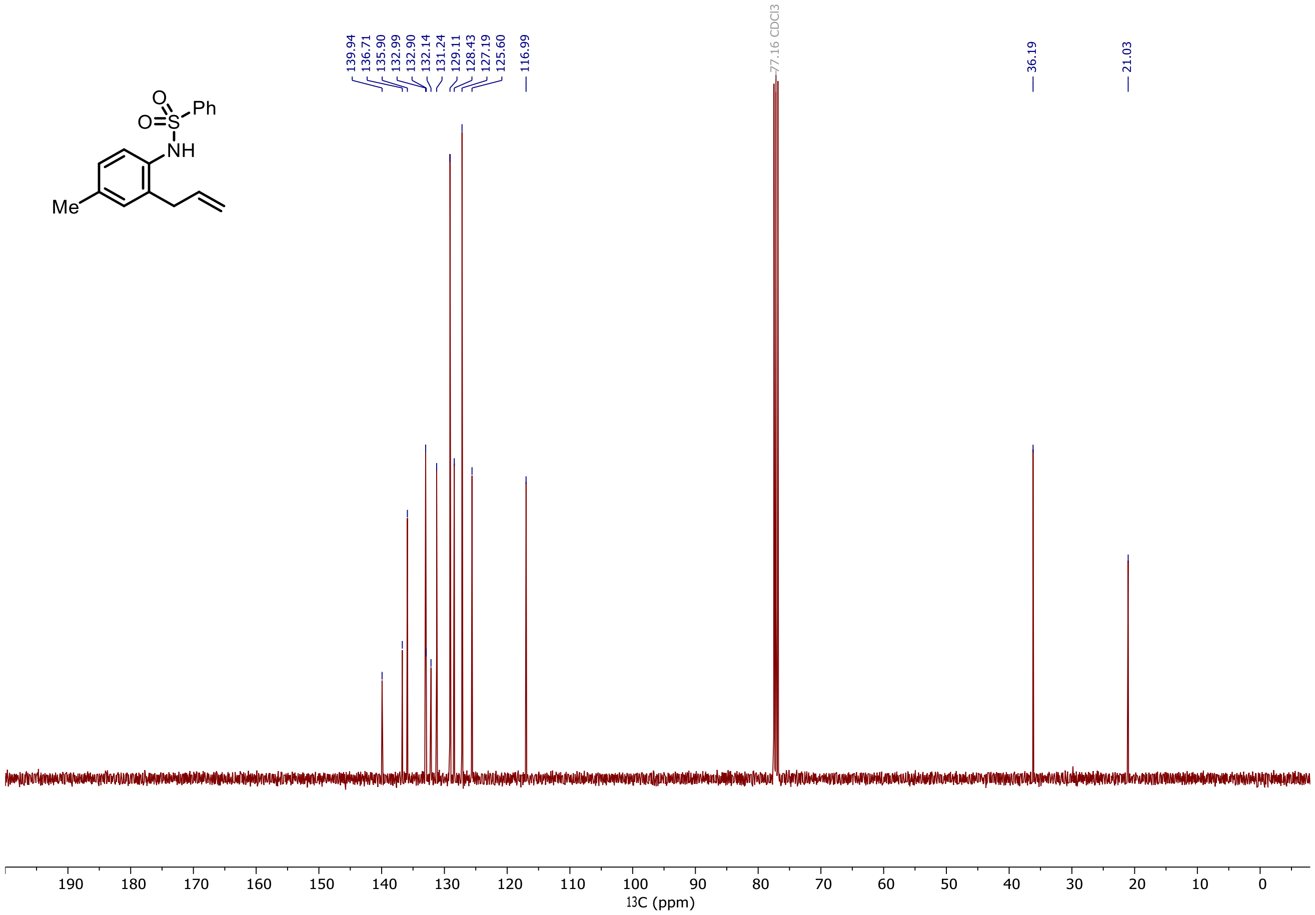

[back to table of contents]

^{1}H NMR (400 MHz, $CDCl_3$) of compound **7ac**

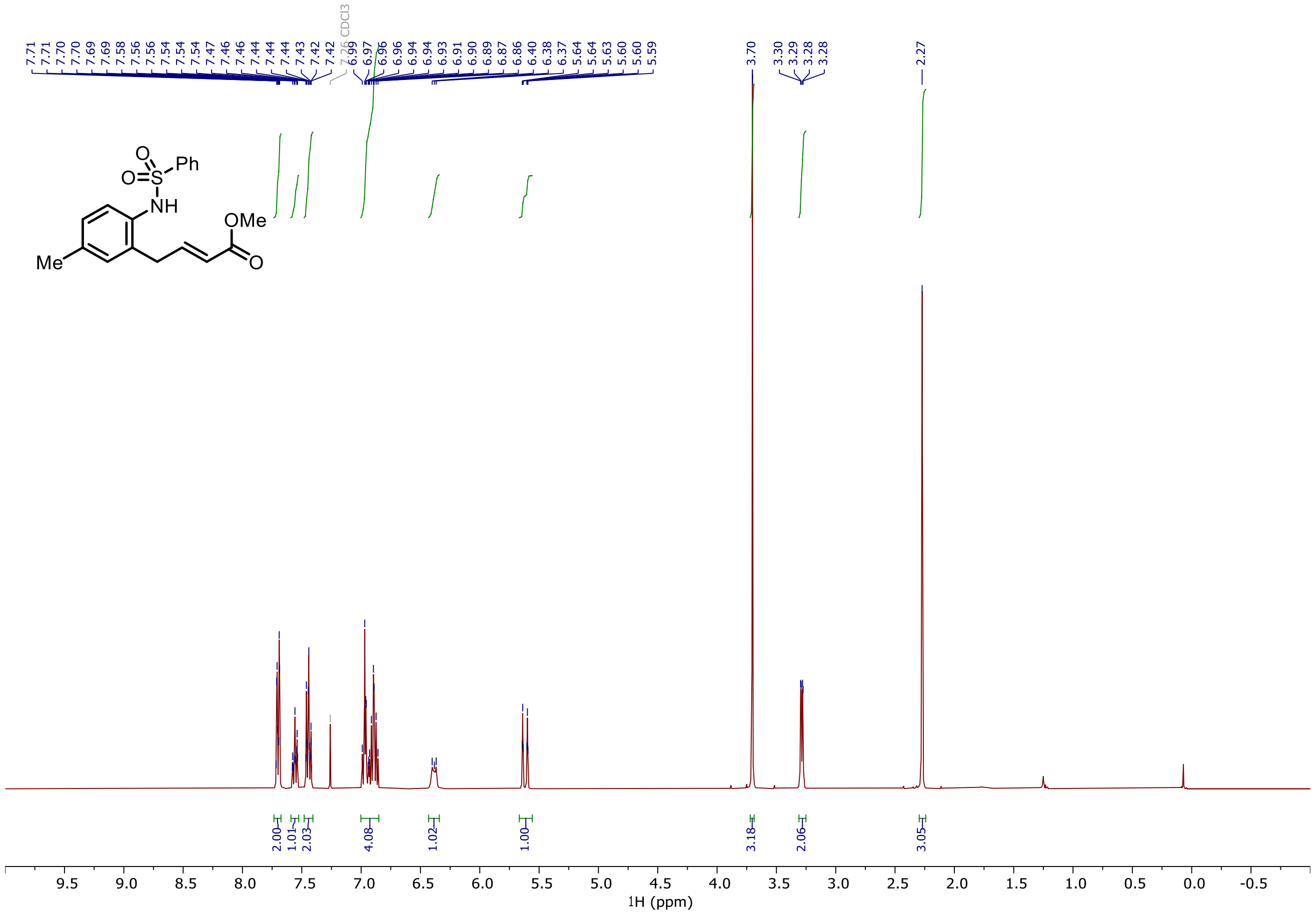

^{13}C NMR (101 MHz, $CDCl_3$) of compound **7ac**

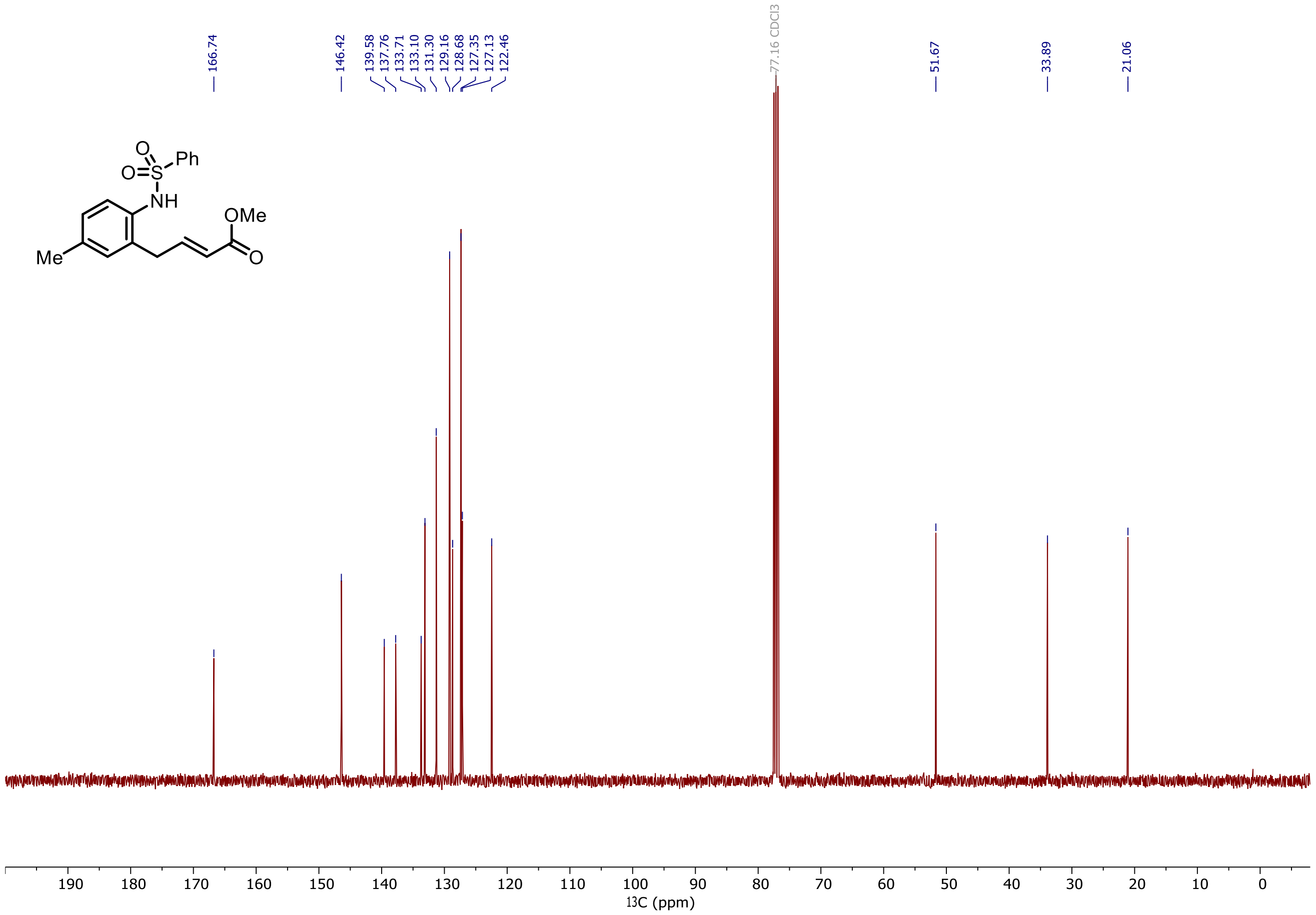

[back to table of contents]

^{1}H NMR (600 MHz, $CDCl_3$) of compound **S43**

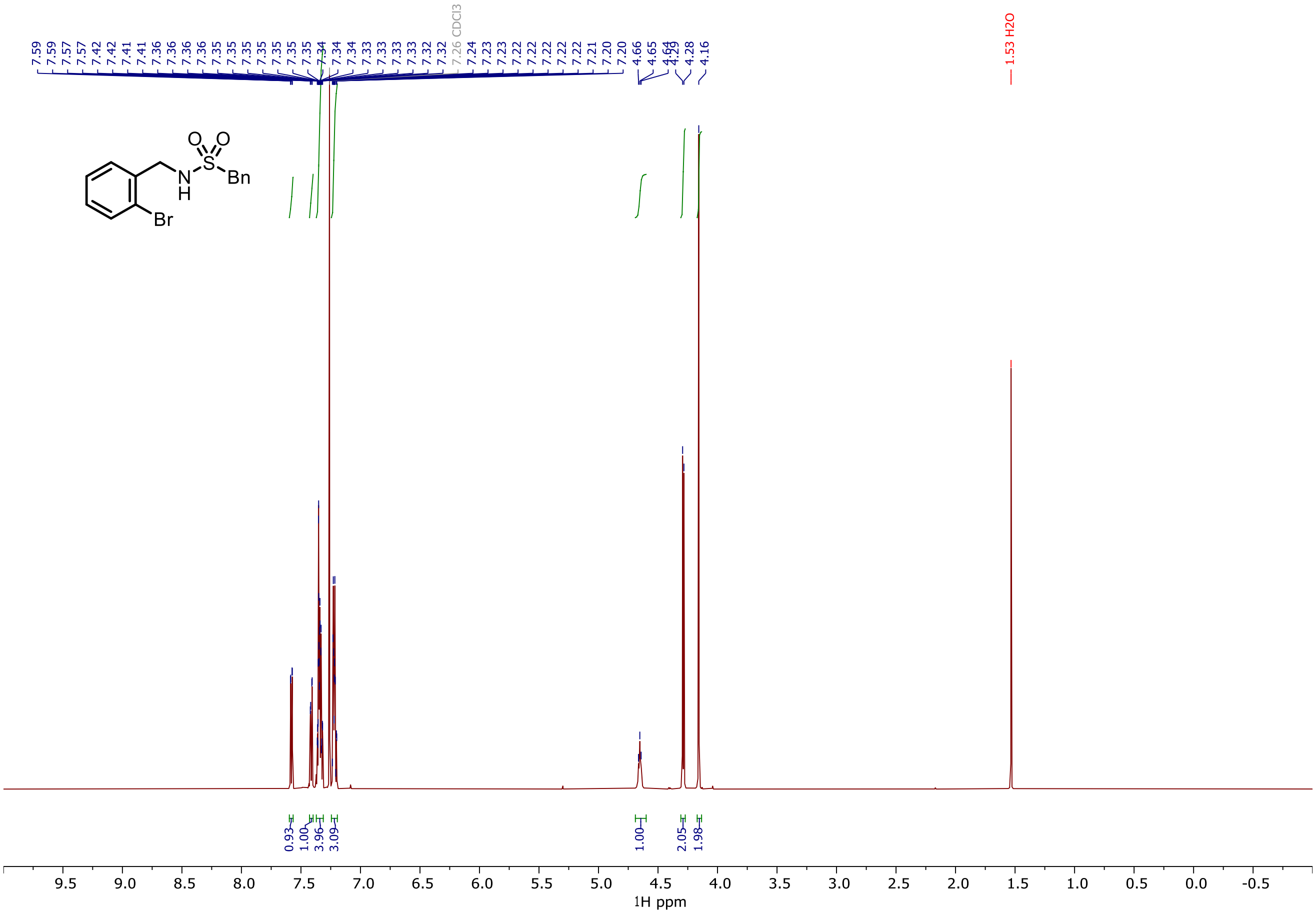

^{13}C NMR (151 MHz, $CDCl_3$) of compound **S43**

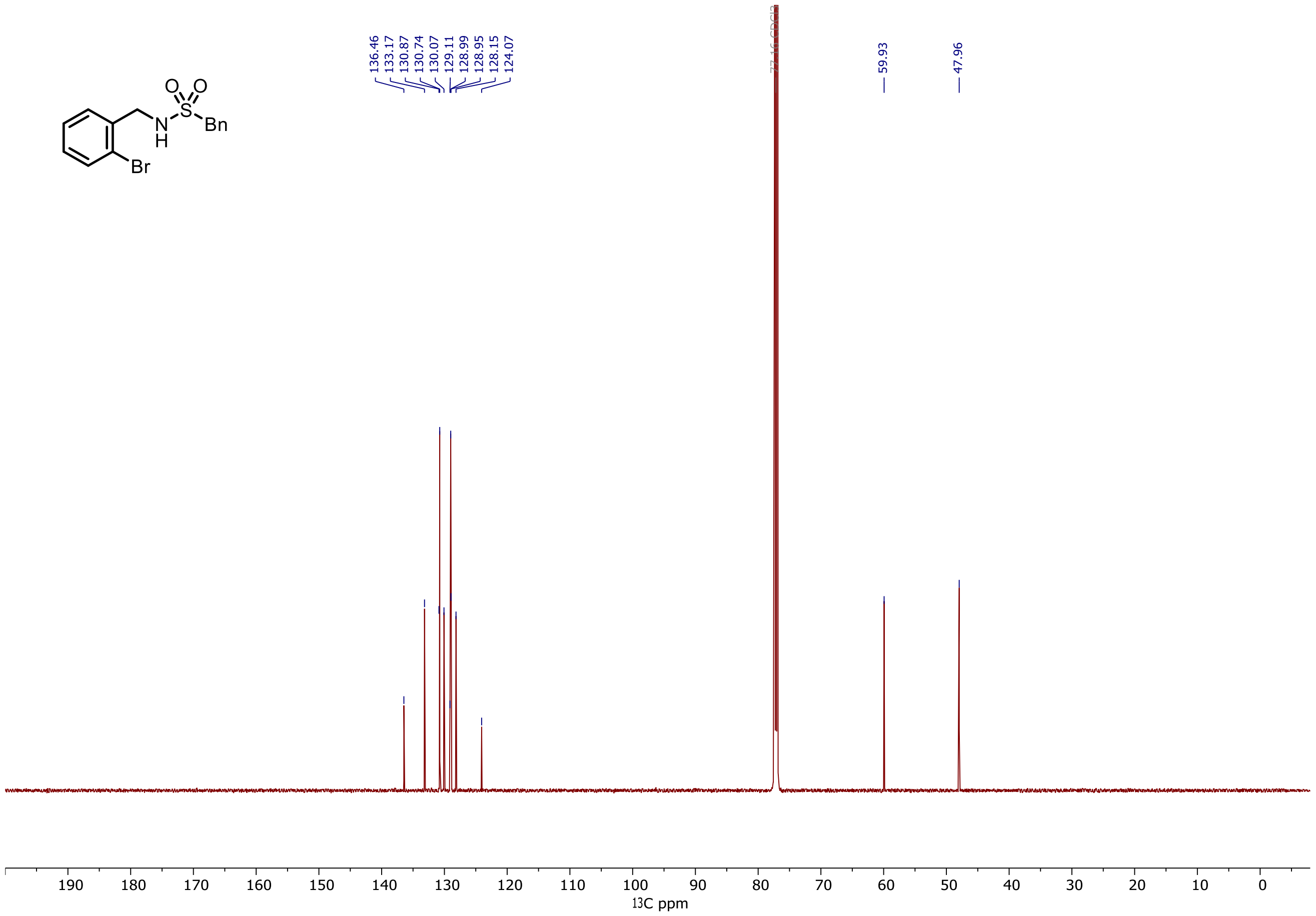

[back to table of contents]

^{1}H NMR (600 MHz, $CDCl_3$) of compound **S44**

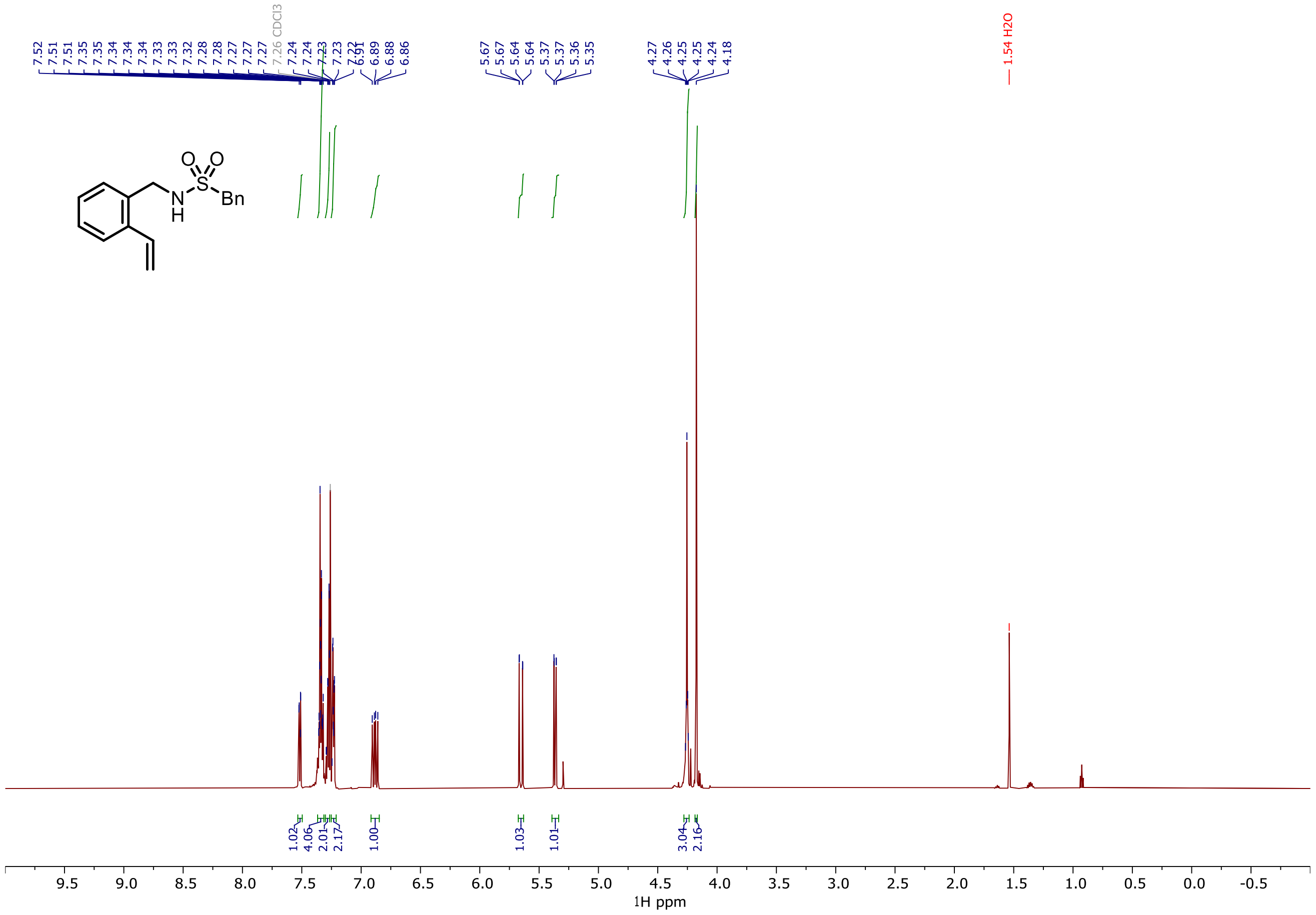

^{13}C NMR (151 MHz, $CDCl_3$) of compound **S44**

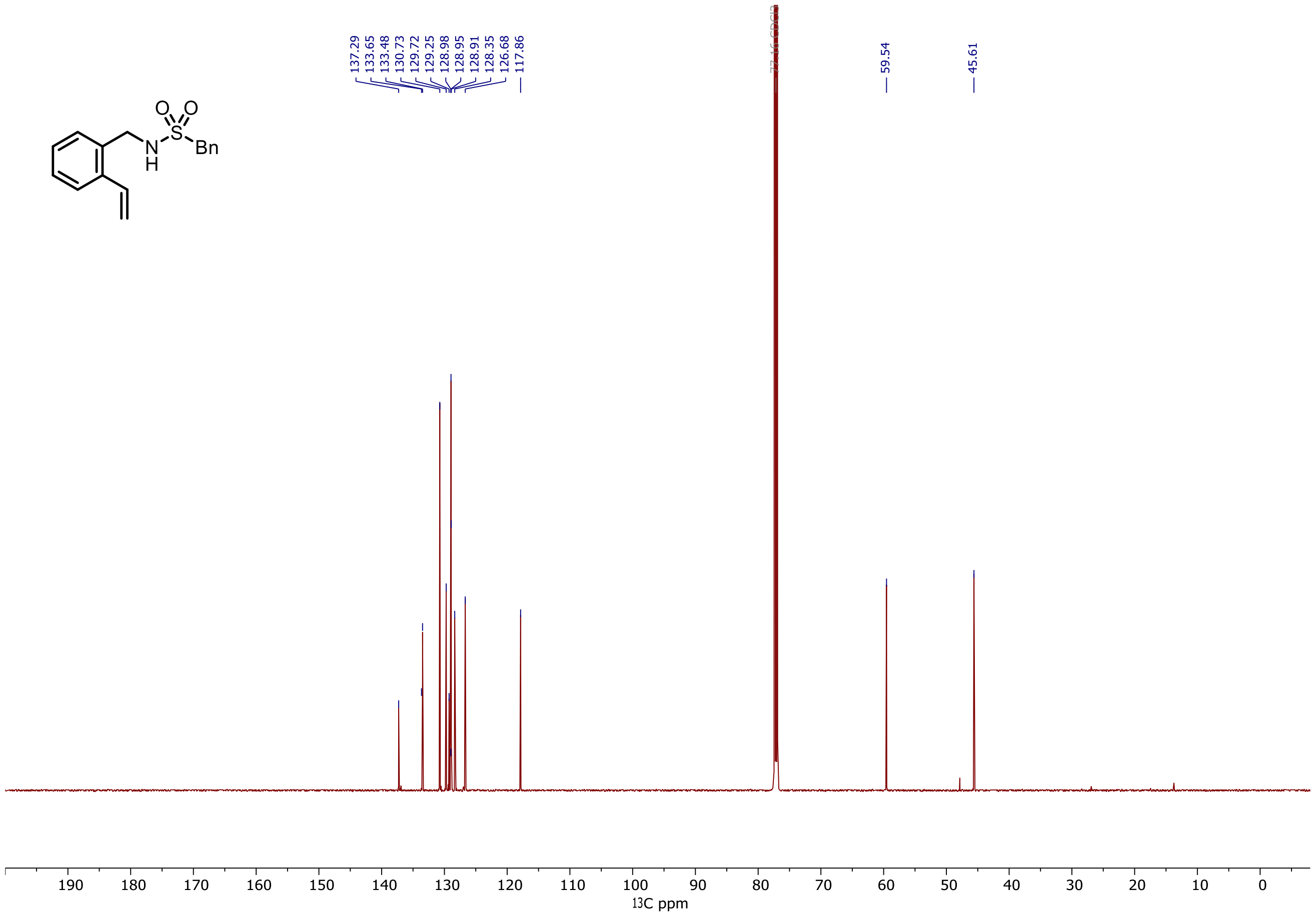

[back to table of contents]

^{1}H NMR (400 MHz, $CDCl_3$) of compound **7ad**

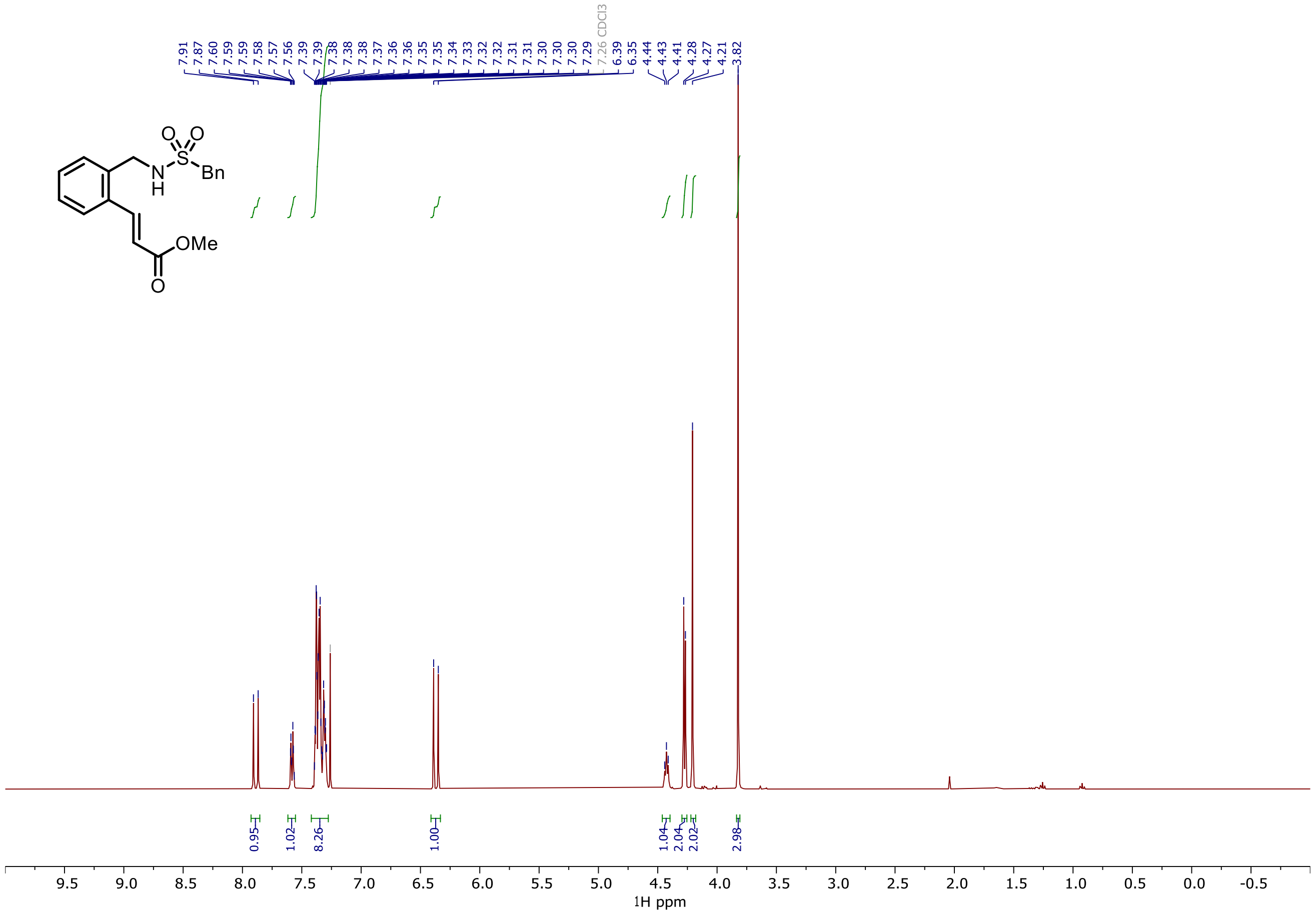

^{13}C NMR (101 MHz, $CDCl_3$) of compound **7ad**

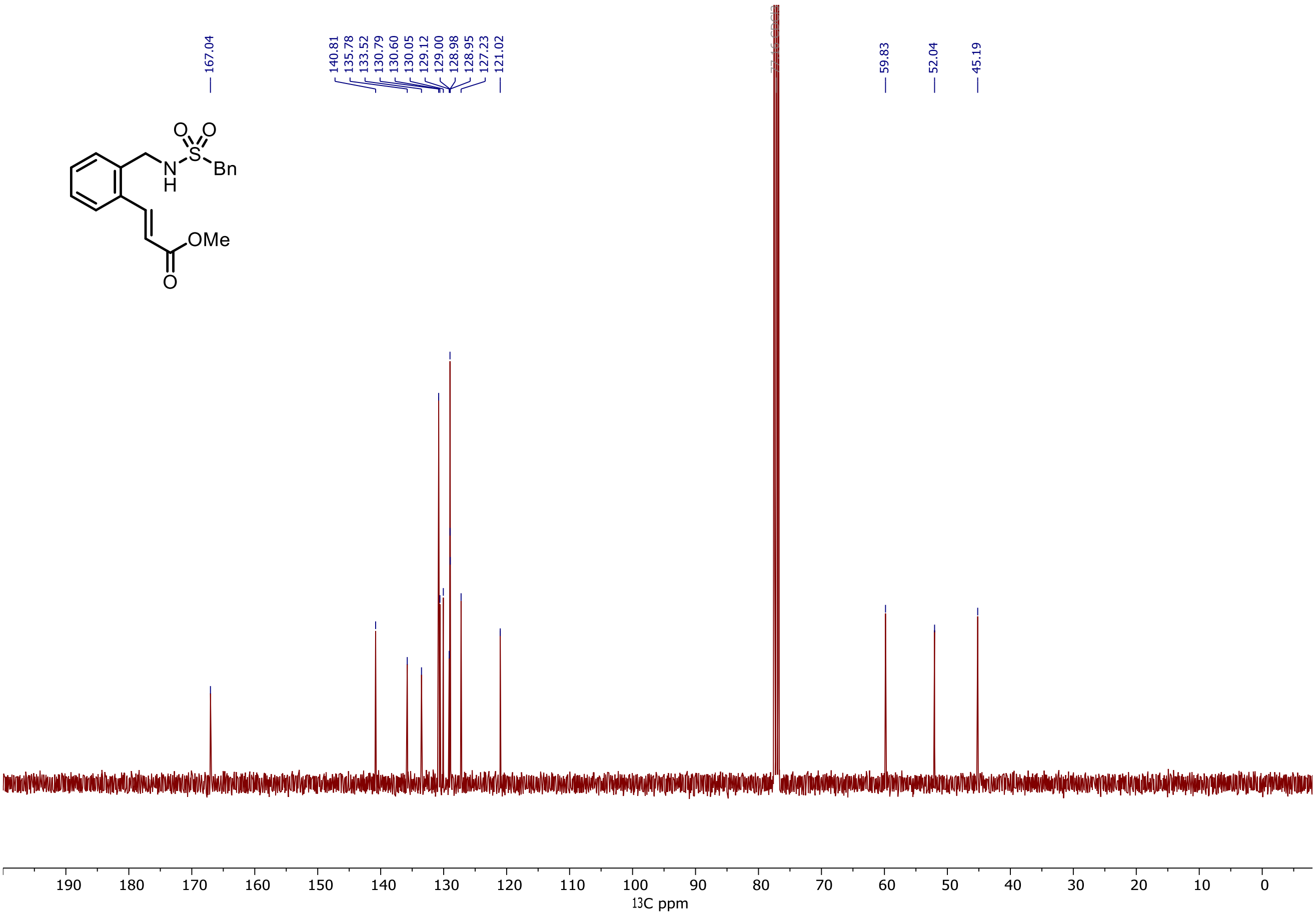

[back to table of contents]

^{1}H NMR (400 MHz, $CDCl_3$) of compound **7ae**

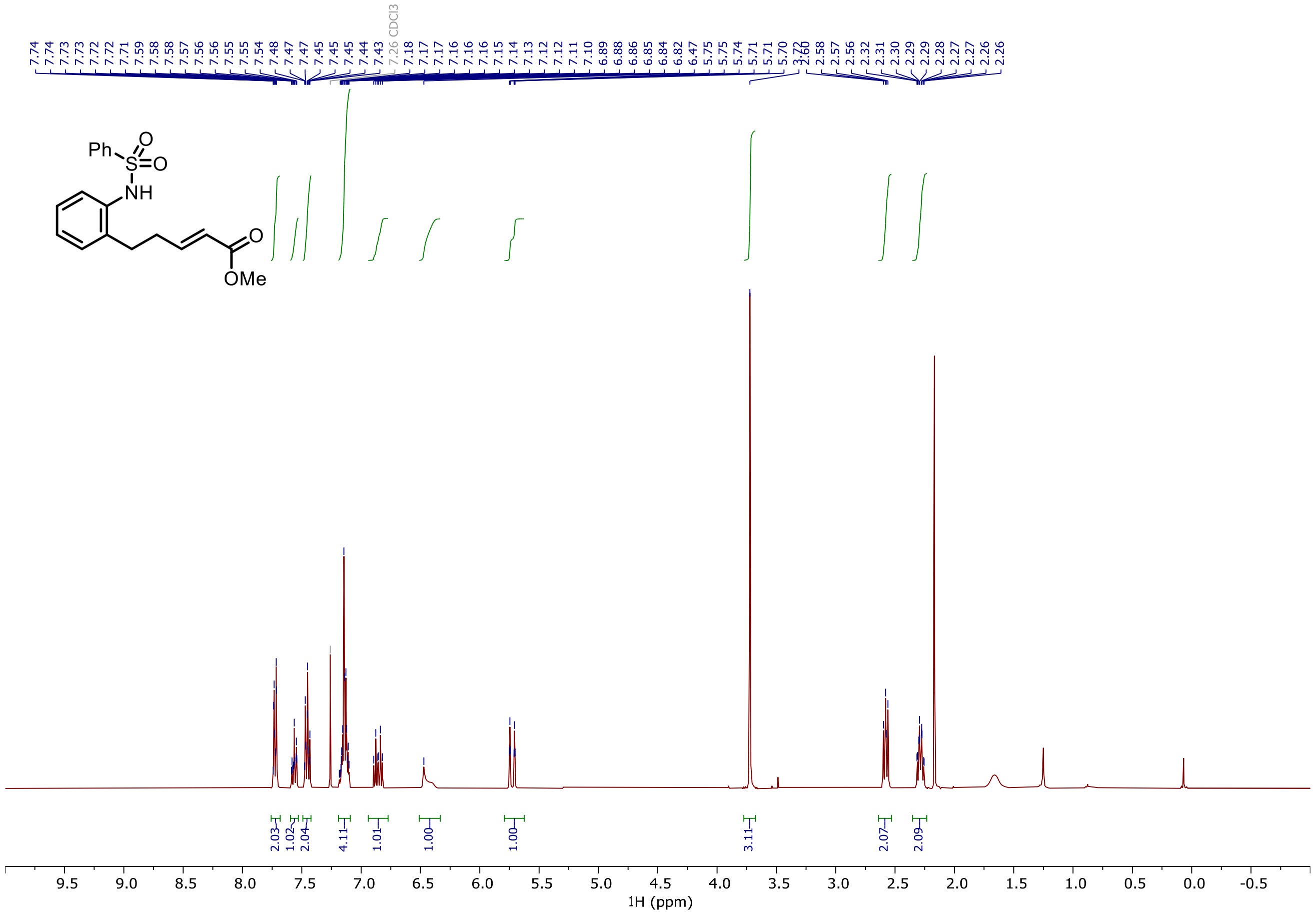

^{13}C NMR (101 MHz, $CDCl_3$) of compound **7ae**

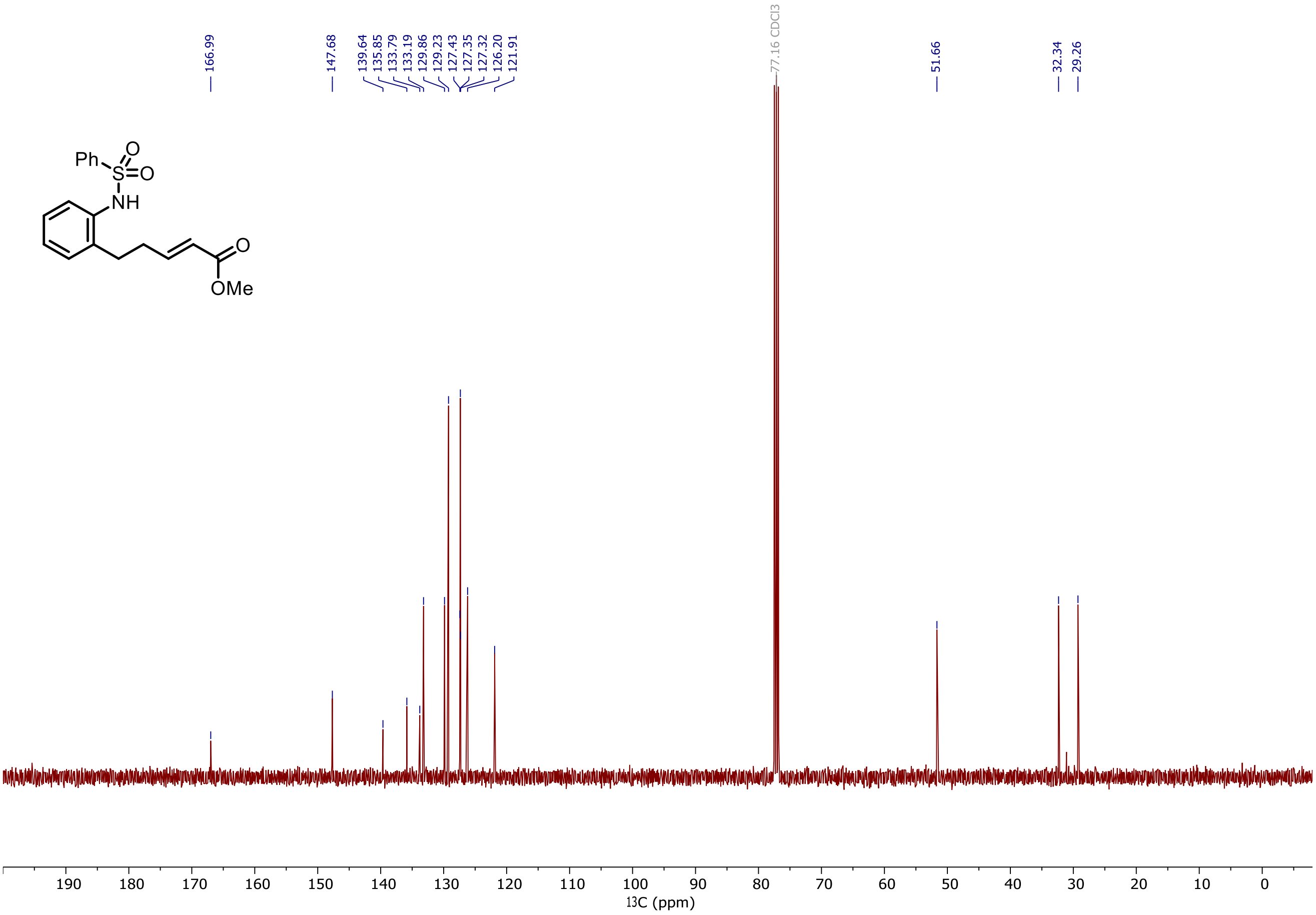

[back to table of contents]

^{1}H NMR (600 MHz, $CDCl_3$) of compound **8a**

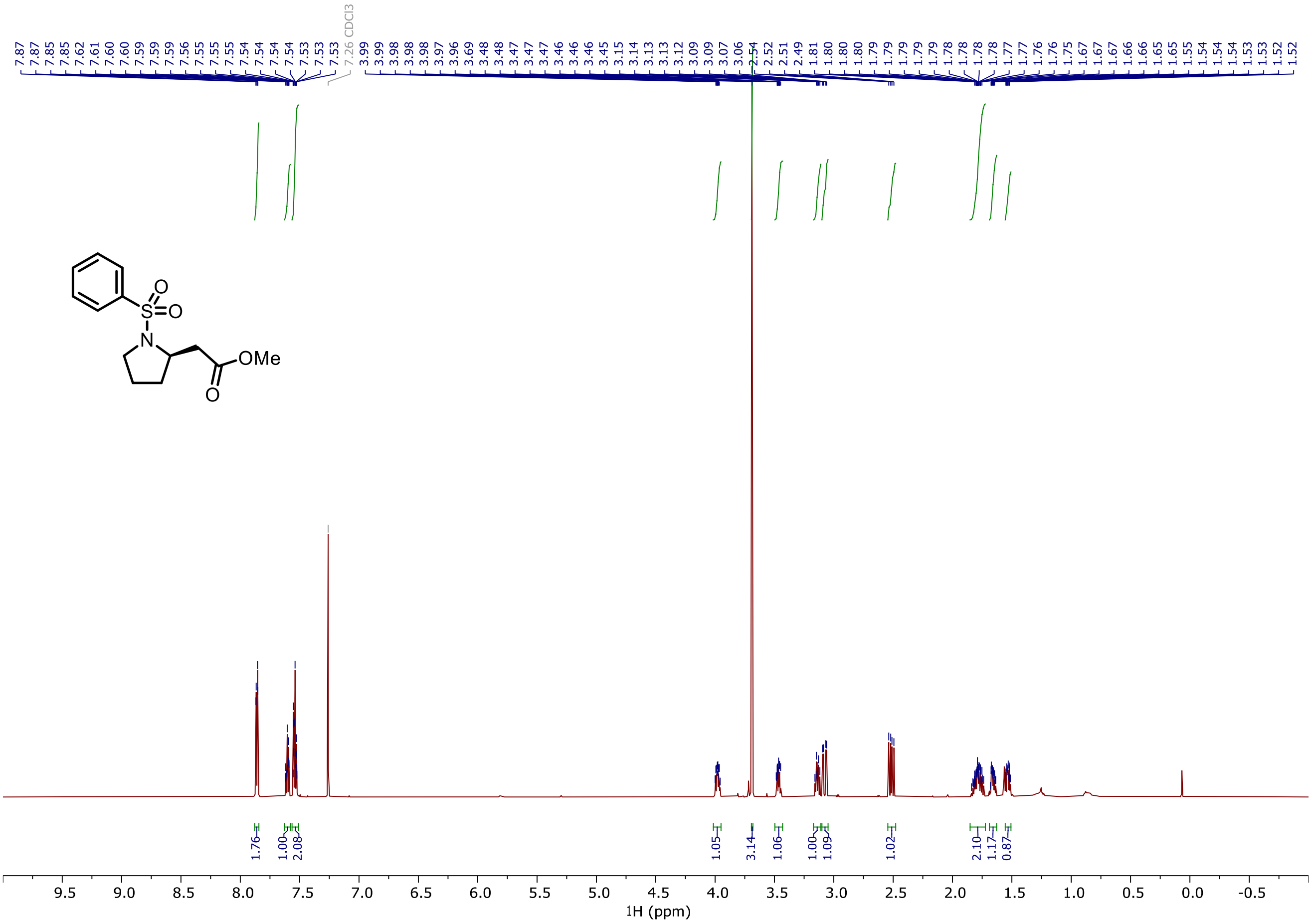

^{13}C NMR (151 MHz, $CDCl_3$) of compound **8a**

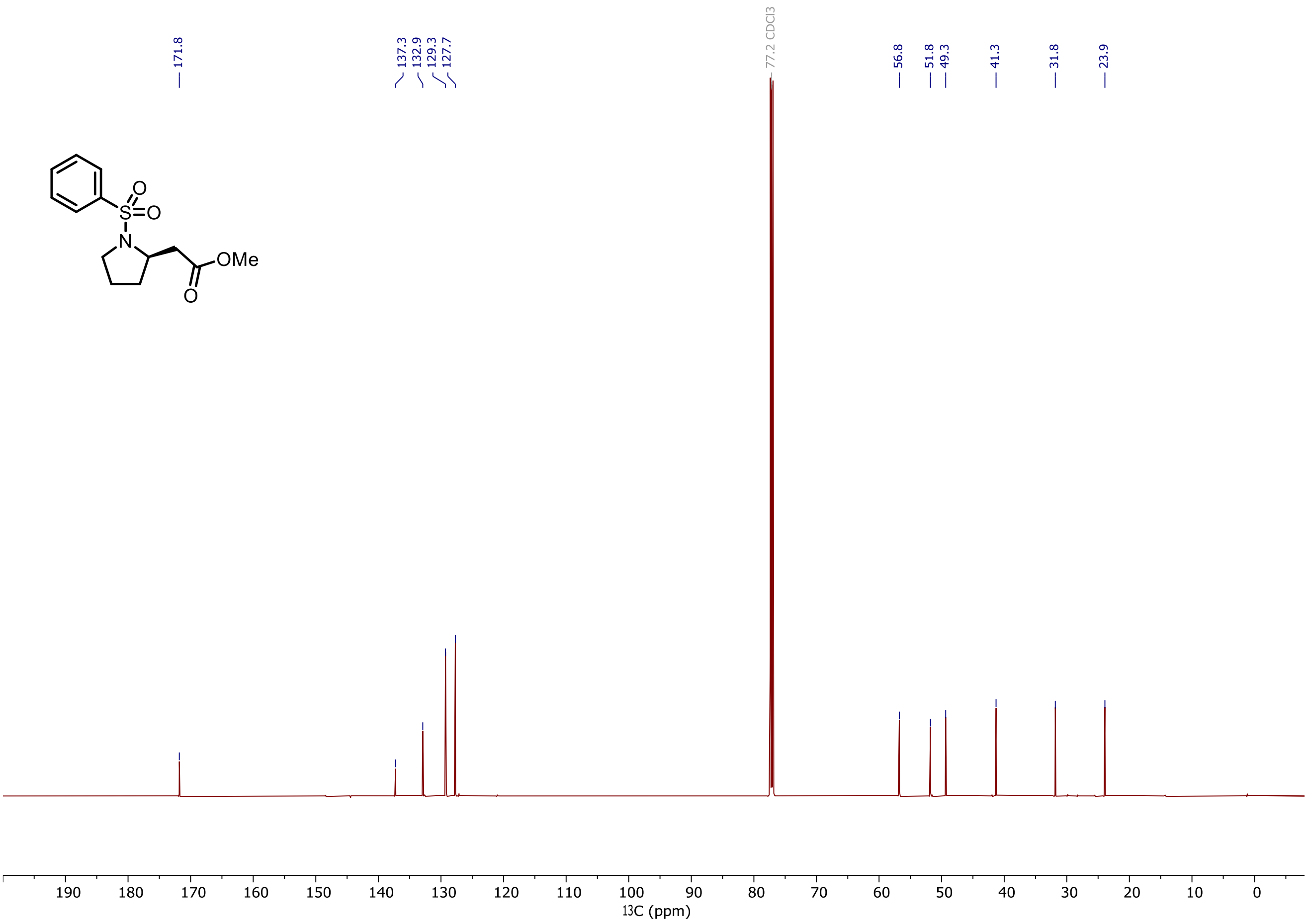

^{1}H NMR (600 MHz, $CDCl_3$) of compound 8b

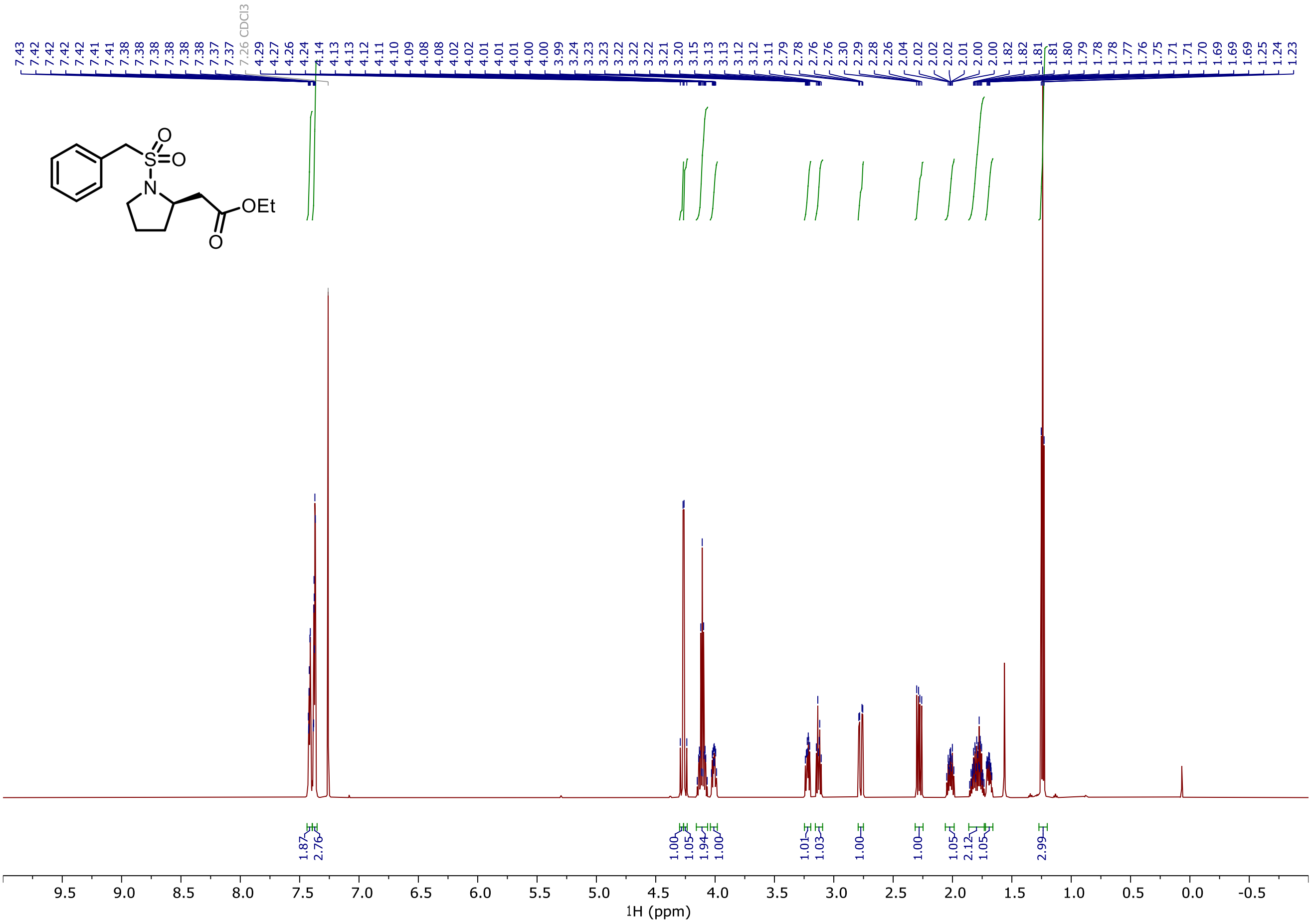

^{13}C NMR (151 MHz, $CDCl_3$) of compound 8b

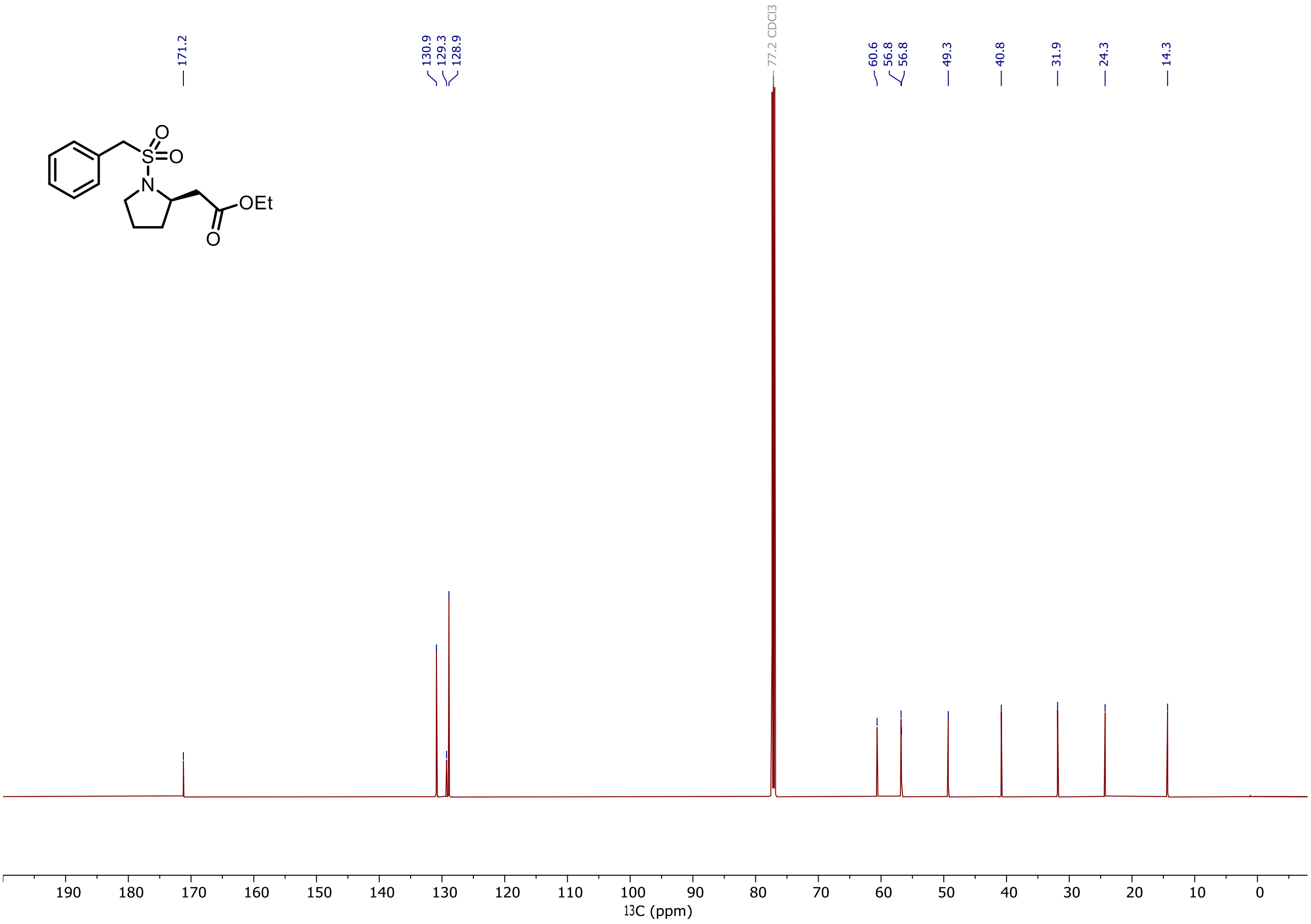

[back to table of contents]

¹H NMR (600 MHz, $CDCl_3$) of compound **8c**

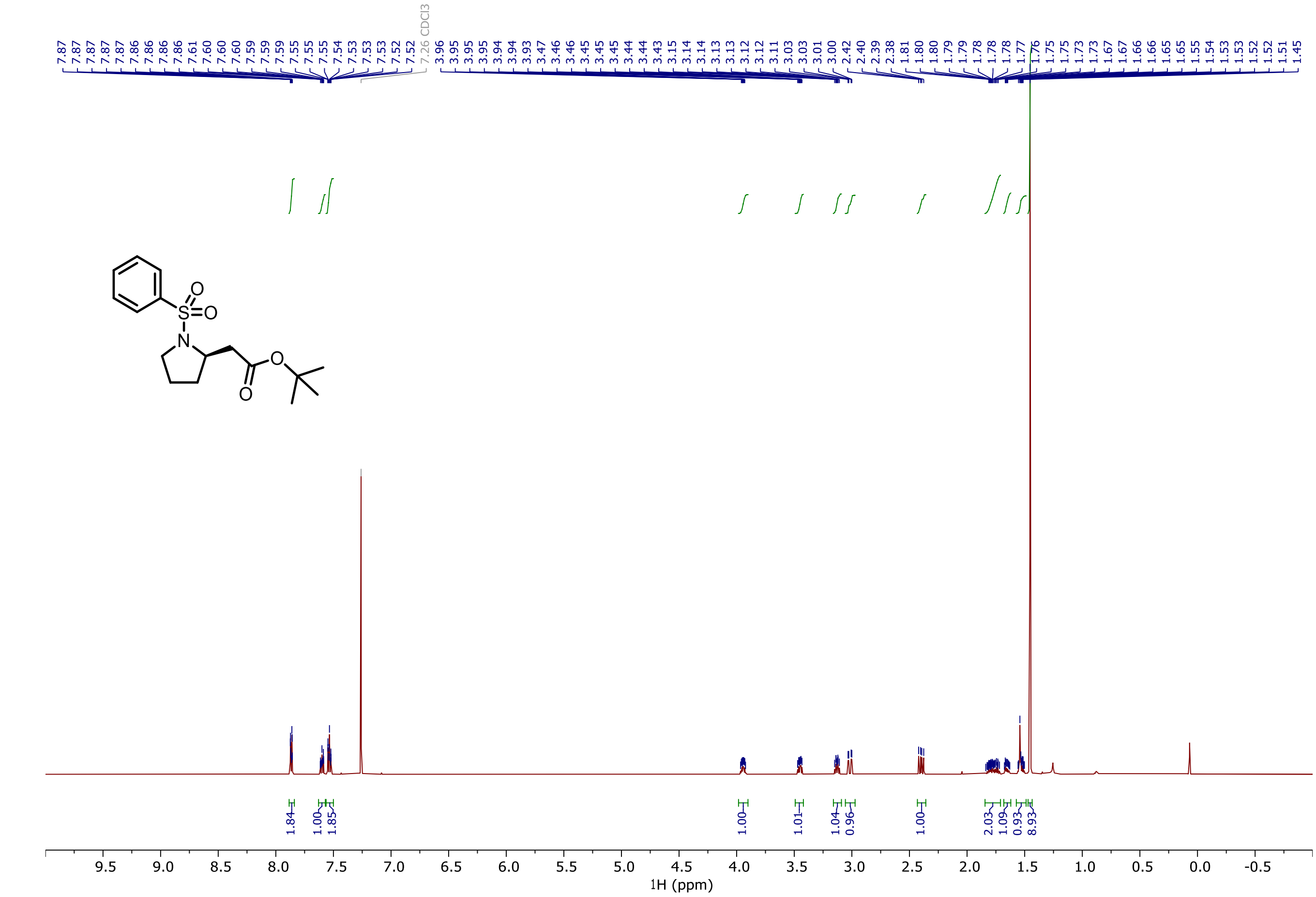

¹³C NMR (151 MHz, $CDCl_3$) of compound **8c**

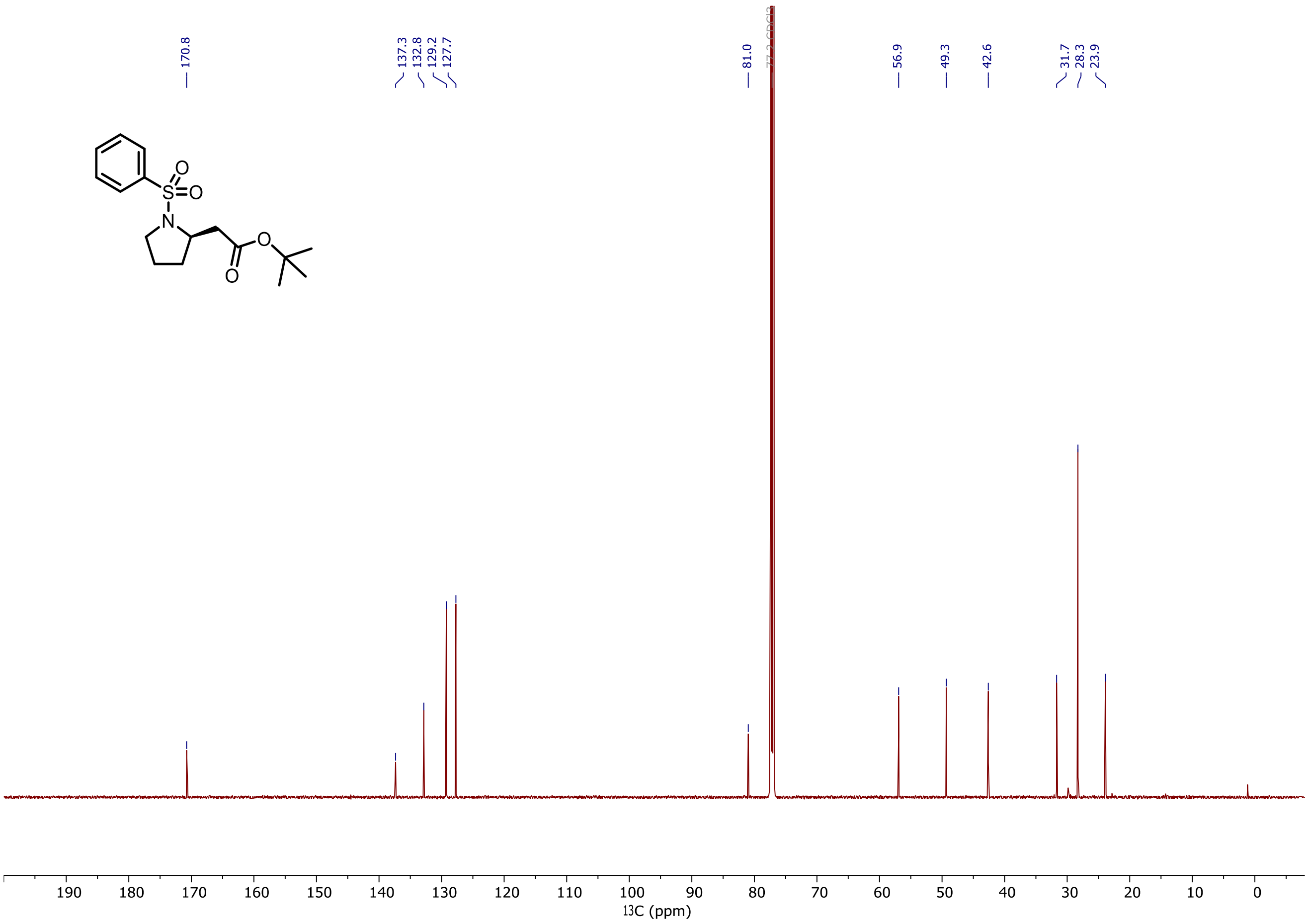

[back to table of contents]

^{1}H NMR (400 MHz, $CDCl_3$) of compound **8d**

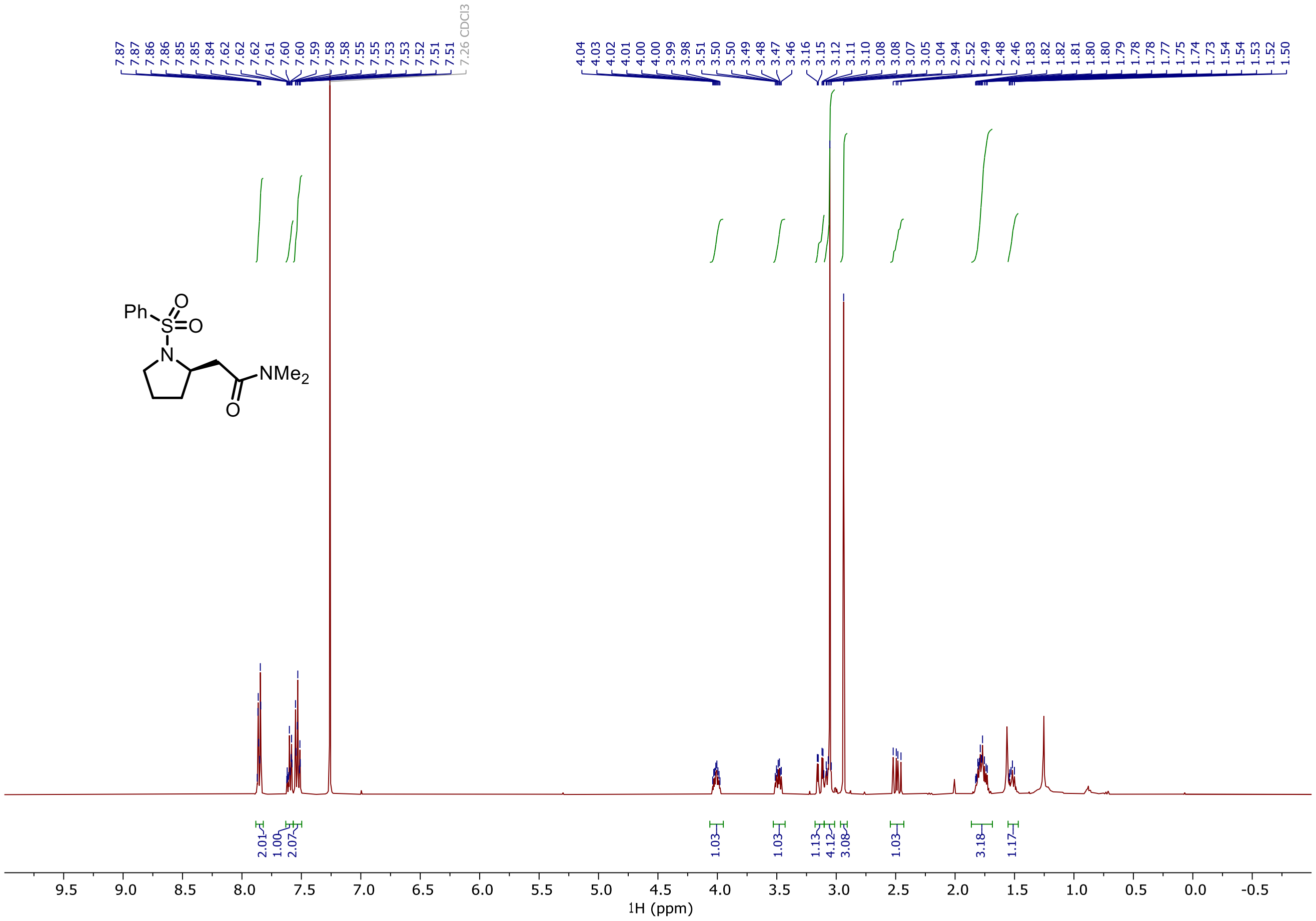

^{13}C NMR (101 MHz, $CDCl_3$) of compound **8d**

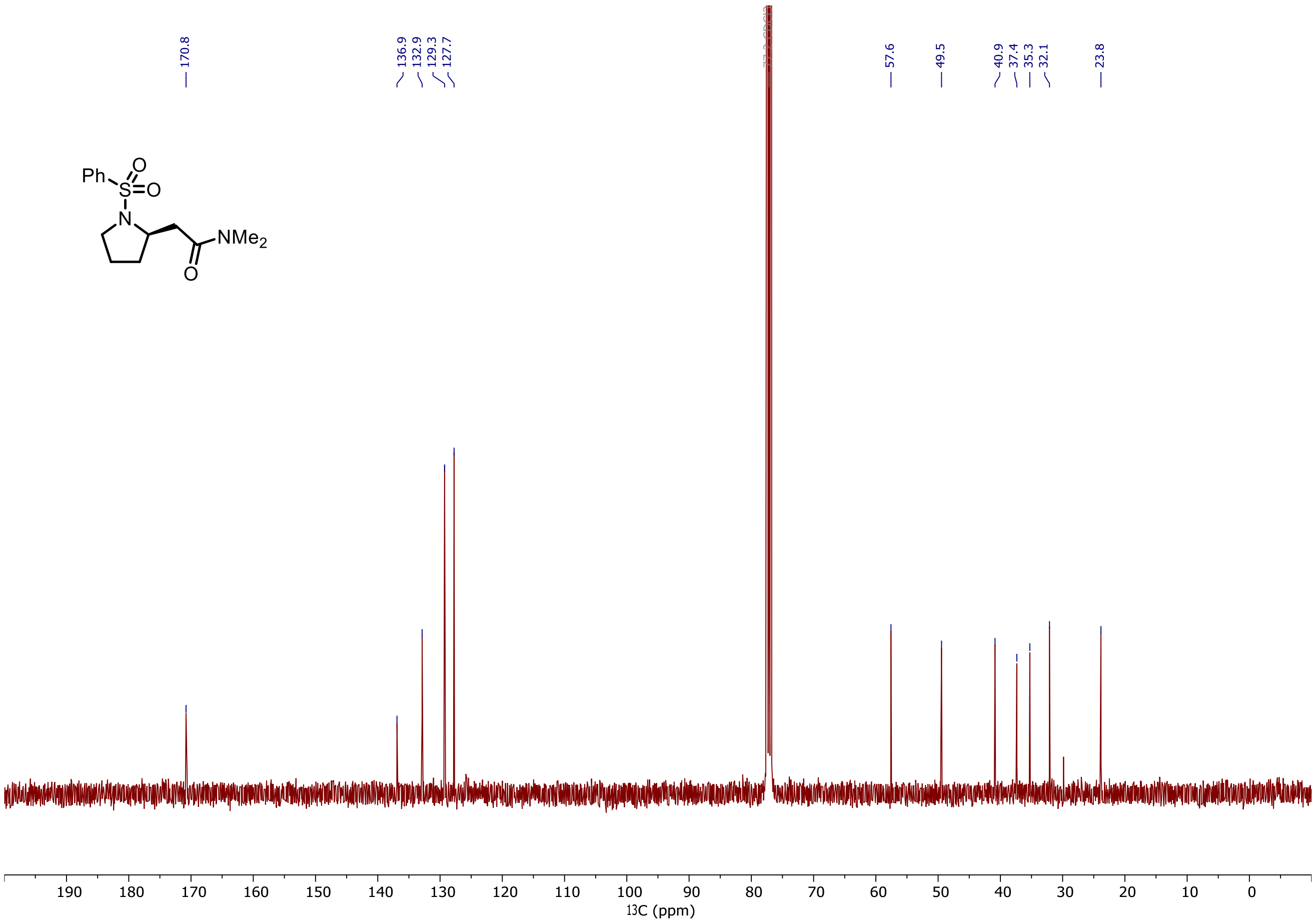

[back to table of contents]

^{1}H NMR (600 MHz, $CDCl_3$) of compound **8e**

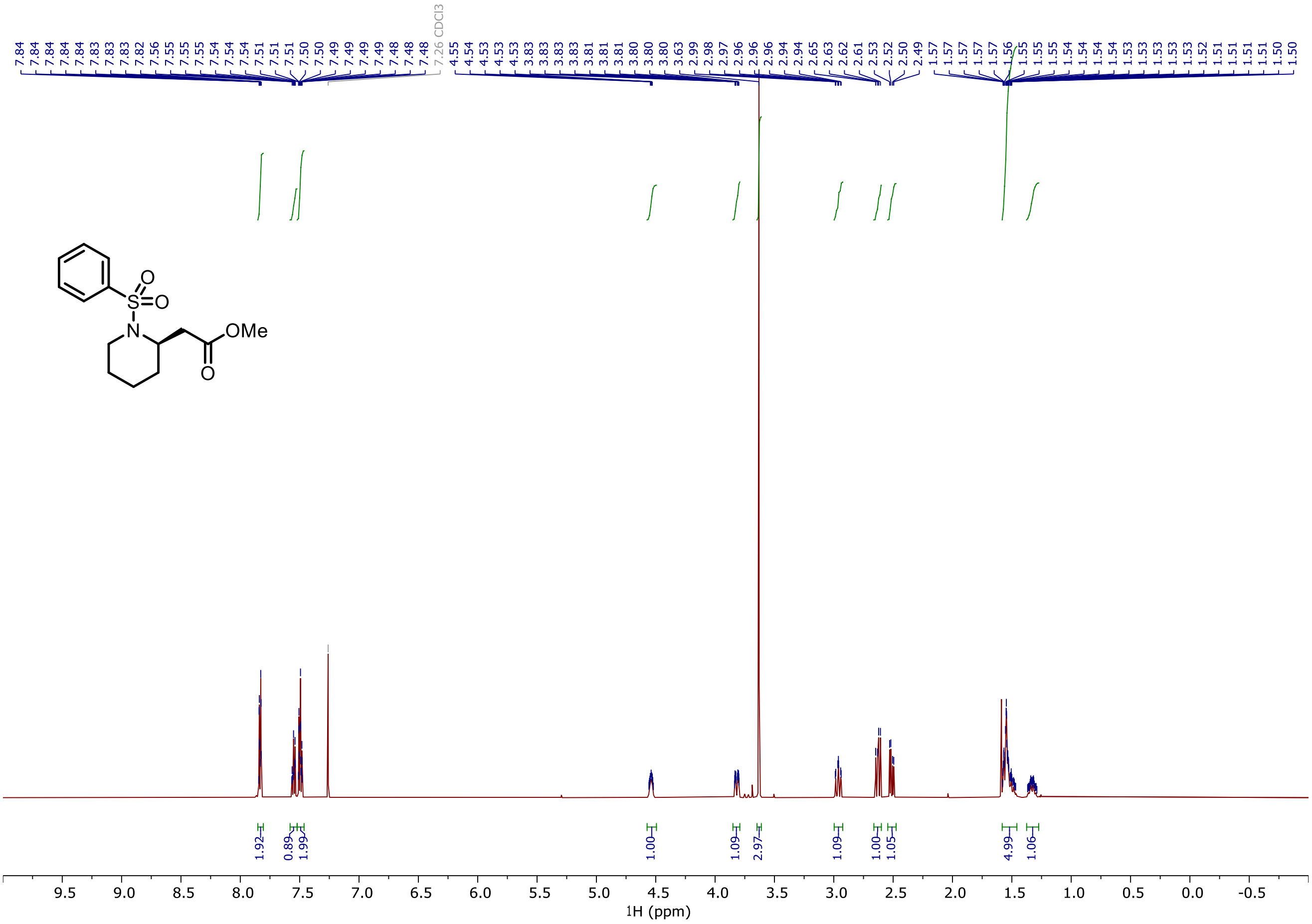

^{13}C NMR (151 MHz, $CDCl_3$) of compound **8e**

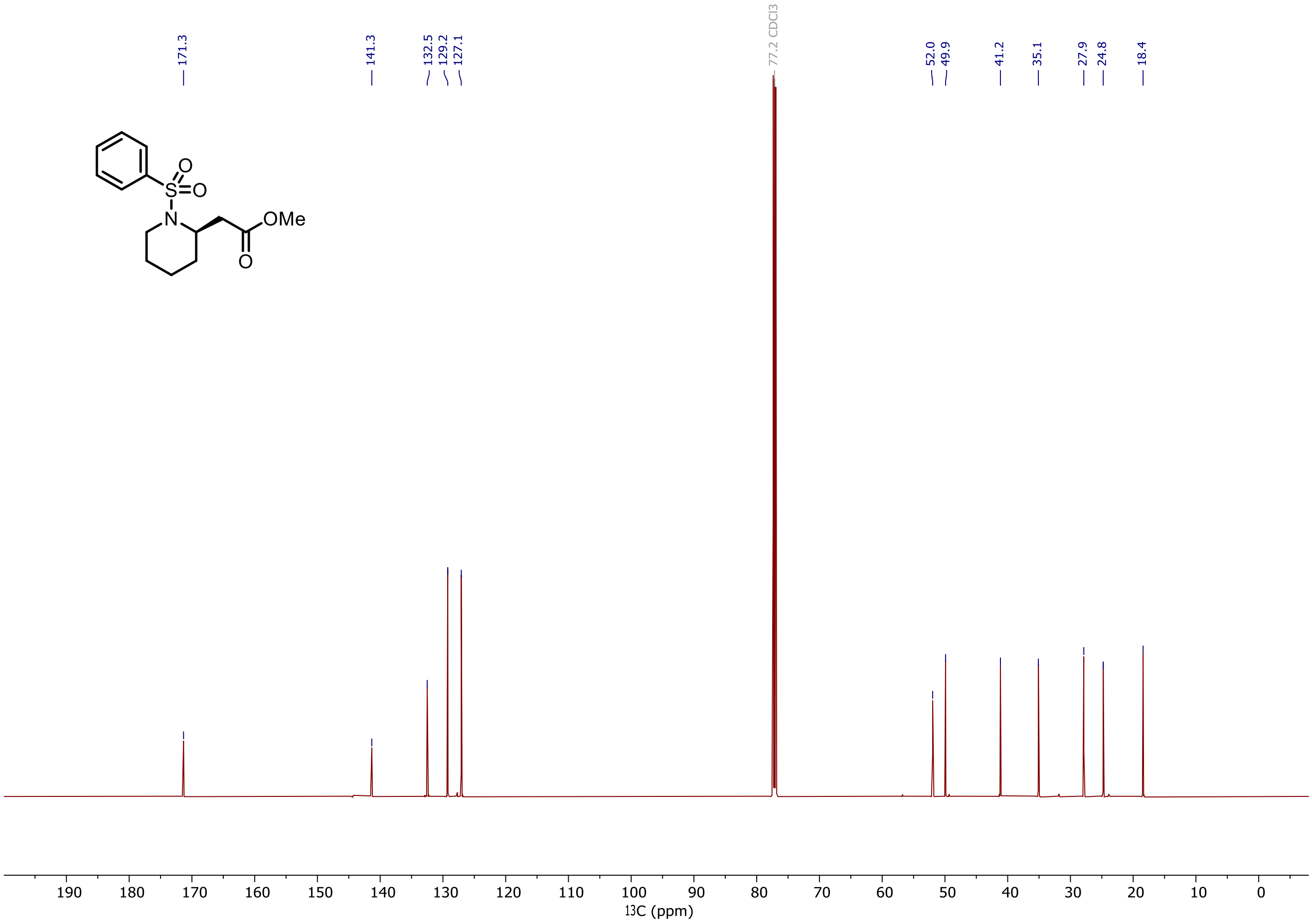

[back to table of contents]

^{1}H NMR (600 MHz, $CDCl_3$) of compound **8f**

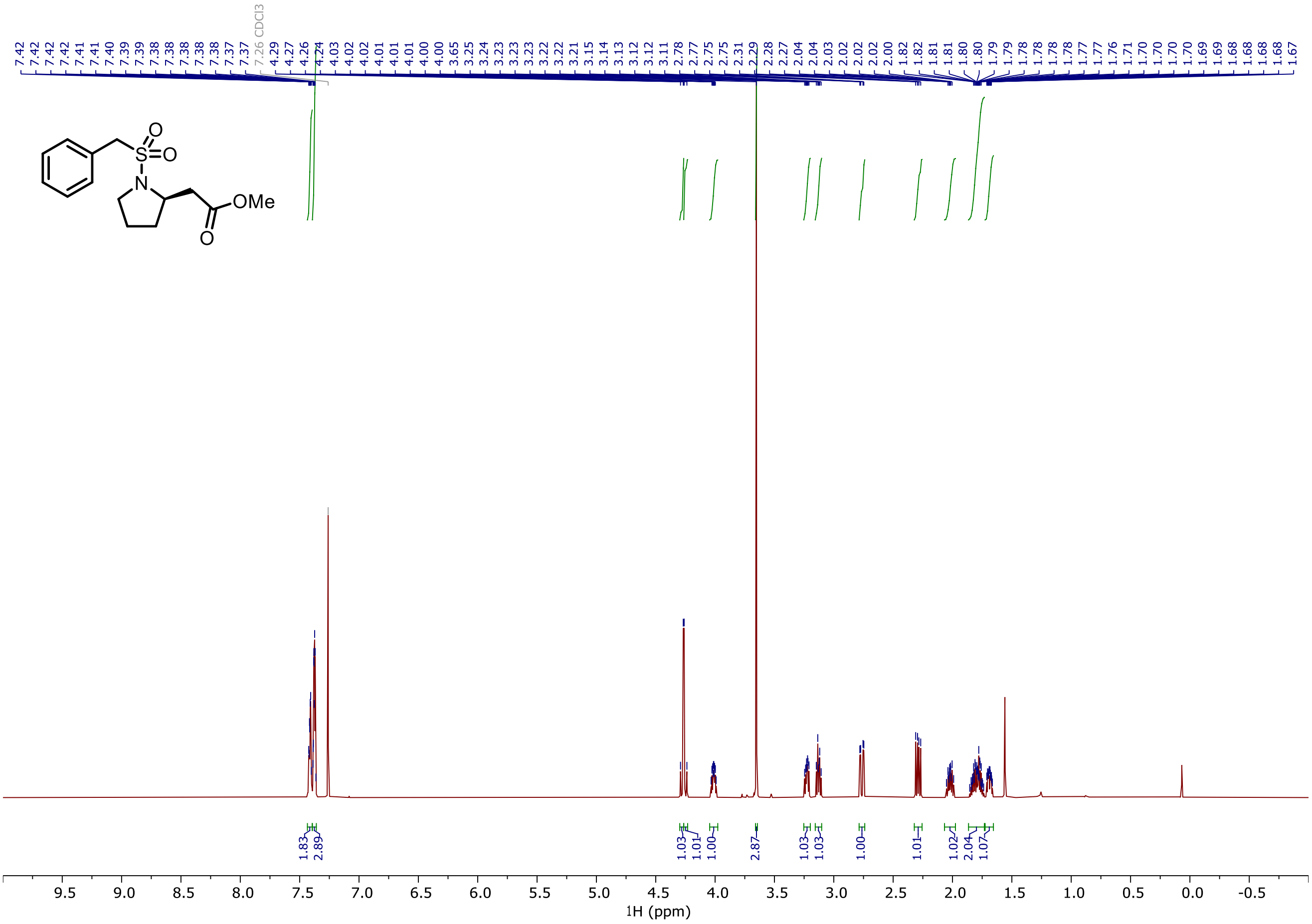

^{13}C NMR (151 MHz, $CDCl_3$) of compound **8f**

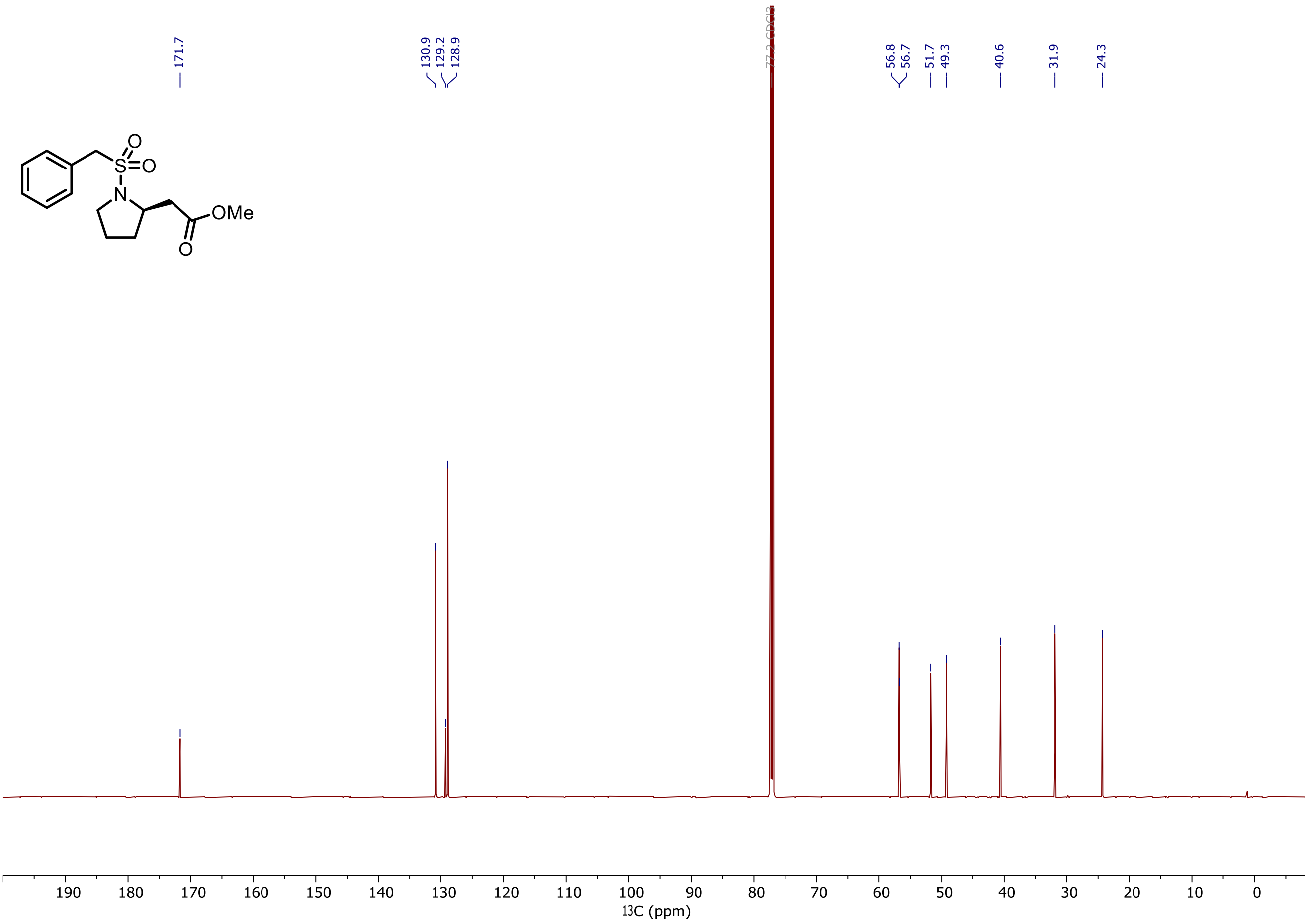

[back to table of contents]

^{1}H NMR (400 MHz, $CDCl_3$) of compound **8g**

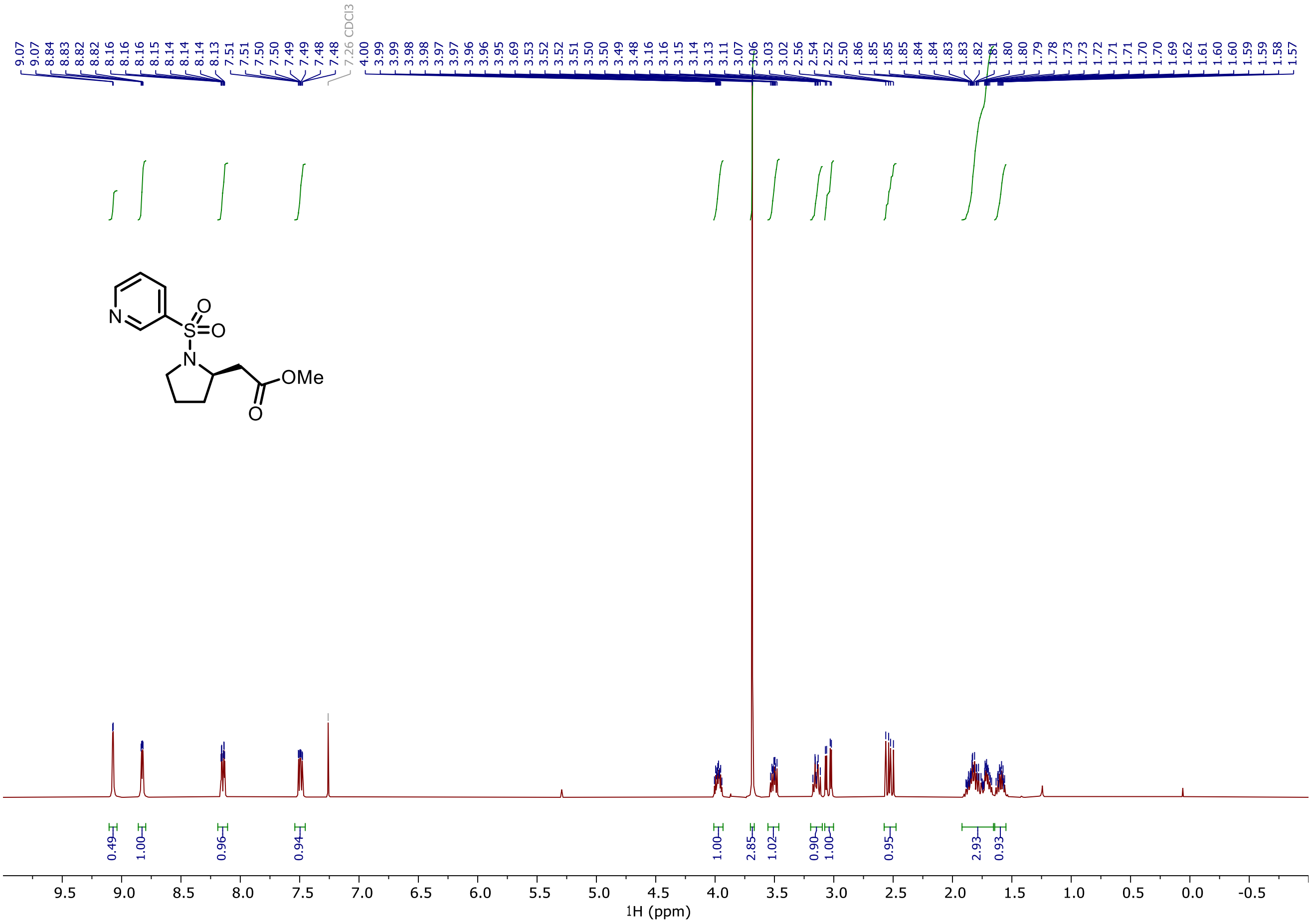

^{13}C NMR (101 MHz, $CDCl_3$) of compound **8g**

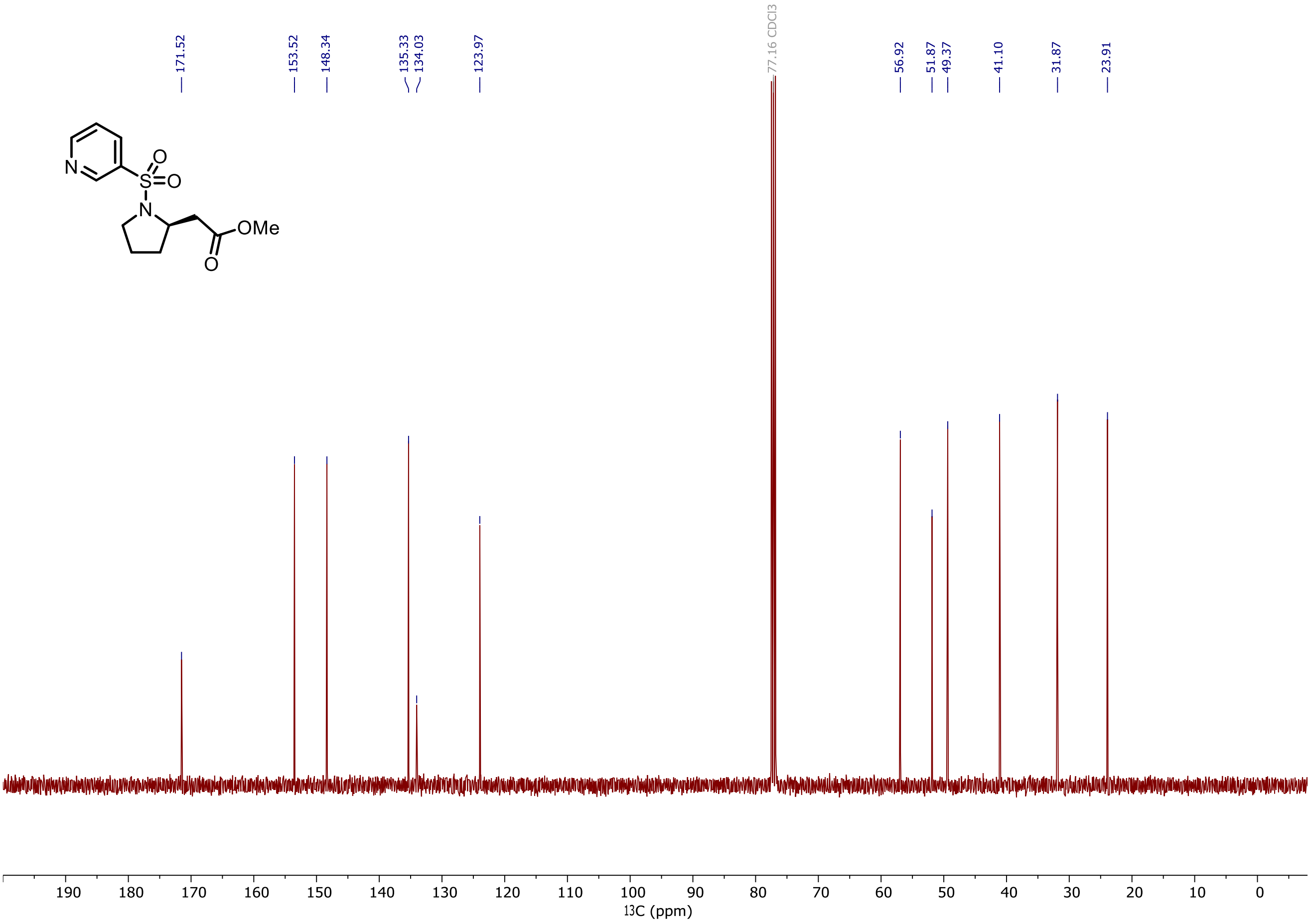

[back to table of contents]

^{1}H NMR (400 MHz, $CDCl_3$) of compound **8h**

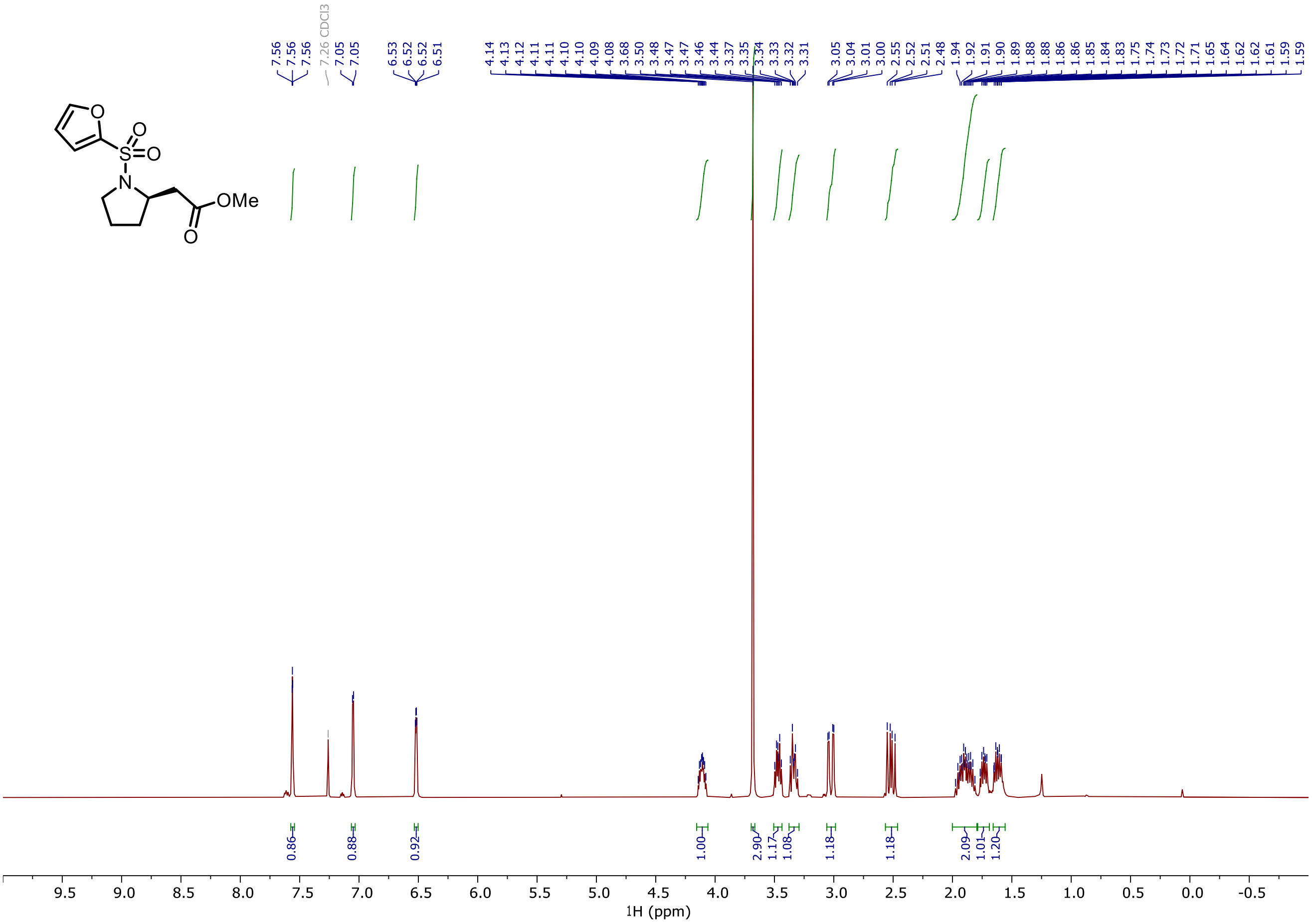

^{13}C NMR (101 MHz, $CDCl_3$) of compound **8h**

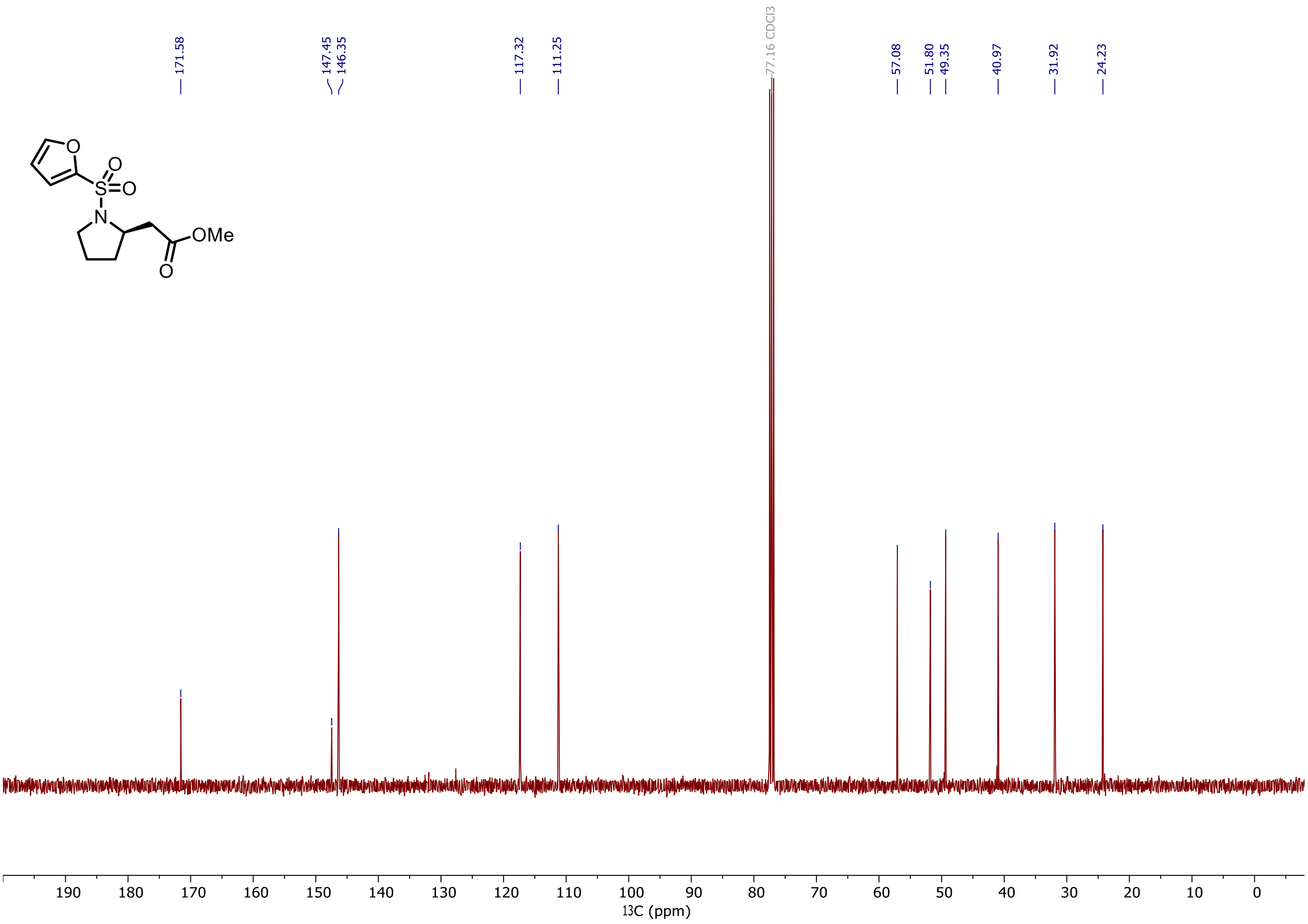

[back to table of contents]

^{1}H NMR (400 MHz, $CDCl_3$) of compound **8i**

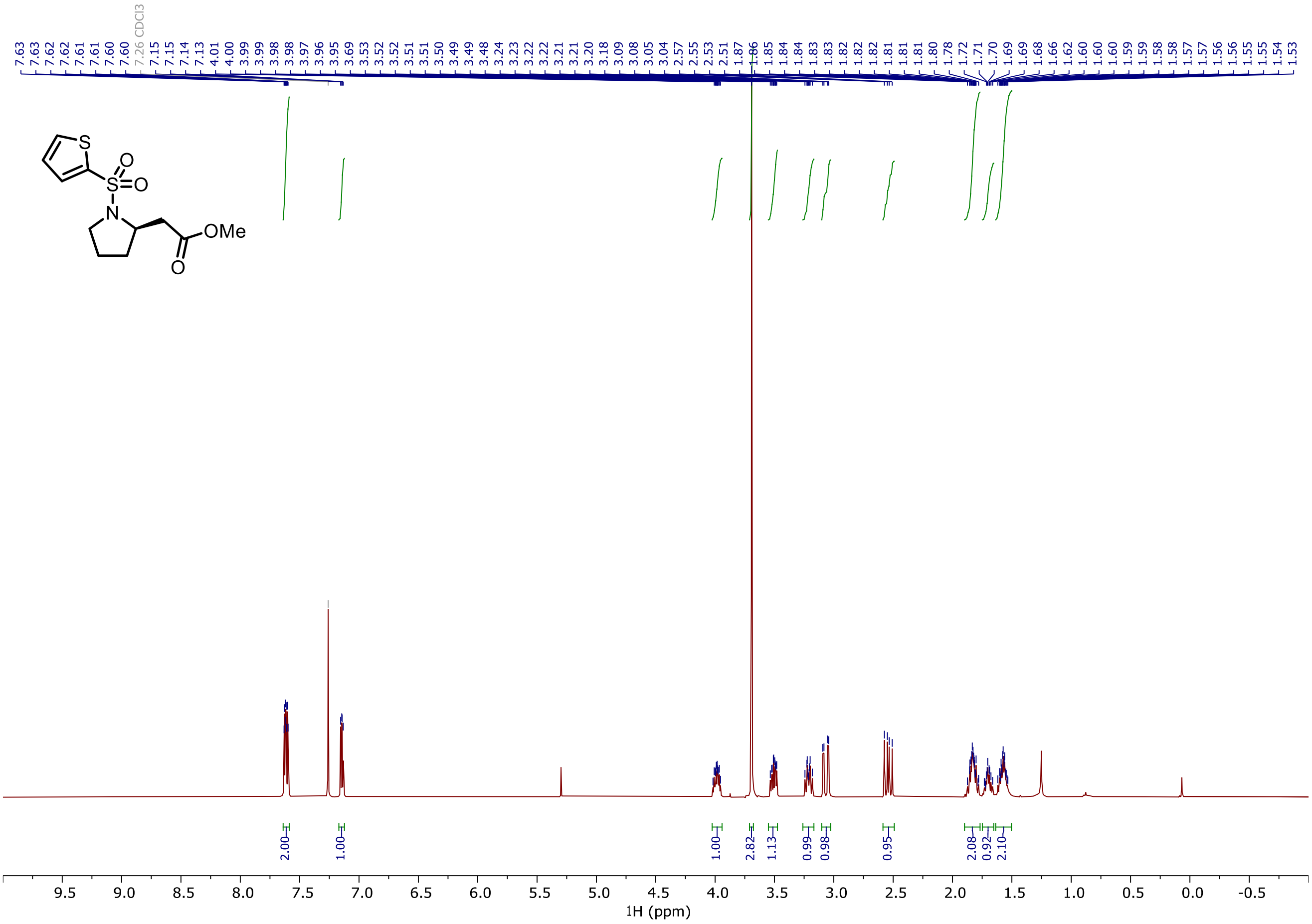

^{13}C NMR (101 MHz, $CDCl_3$) of compound **8i**

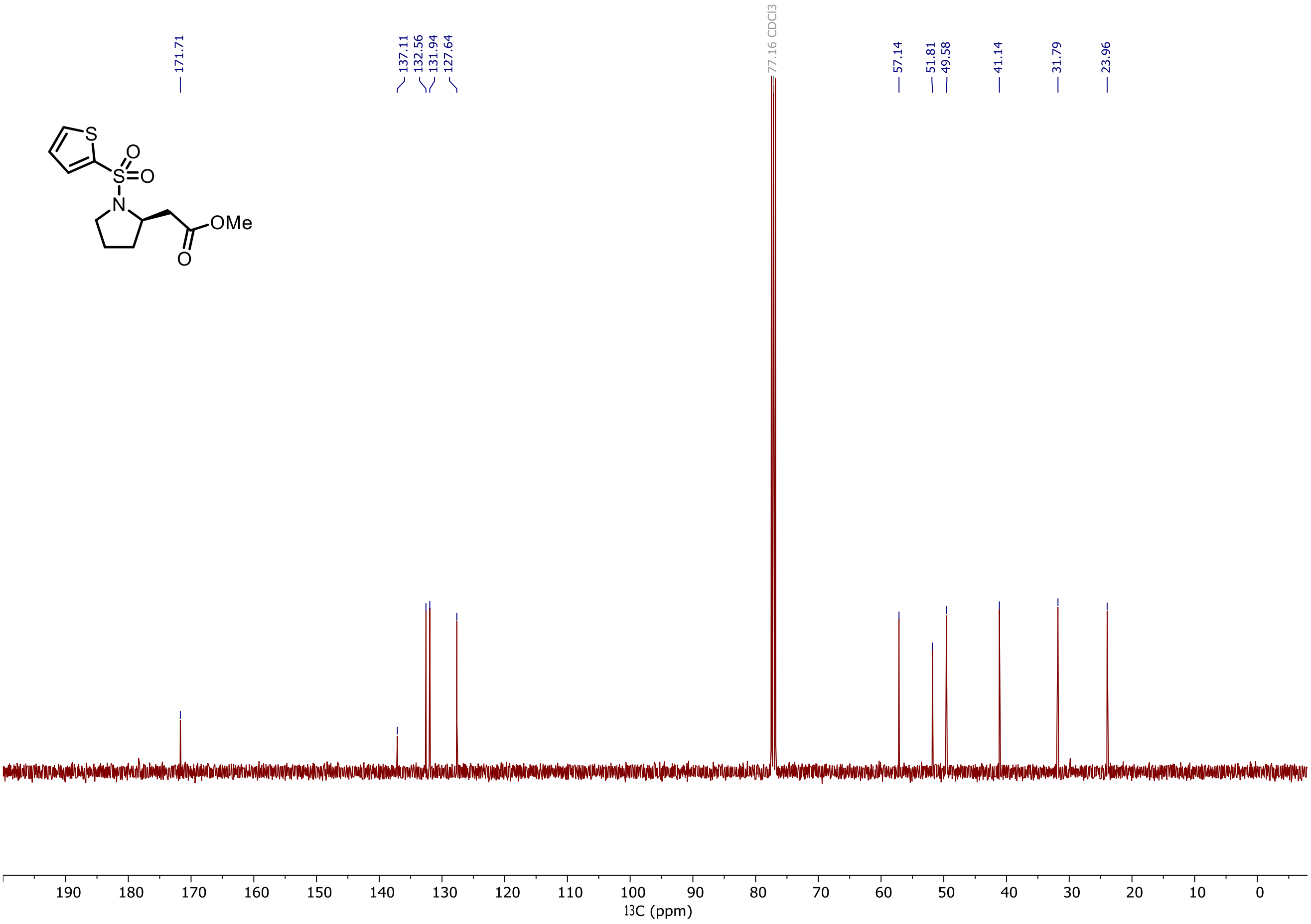

[back to table of contents]

^{1}H NMR (400 MHz, $CDCl_3$) of compound **8j**

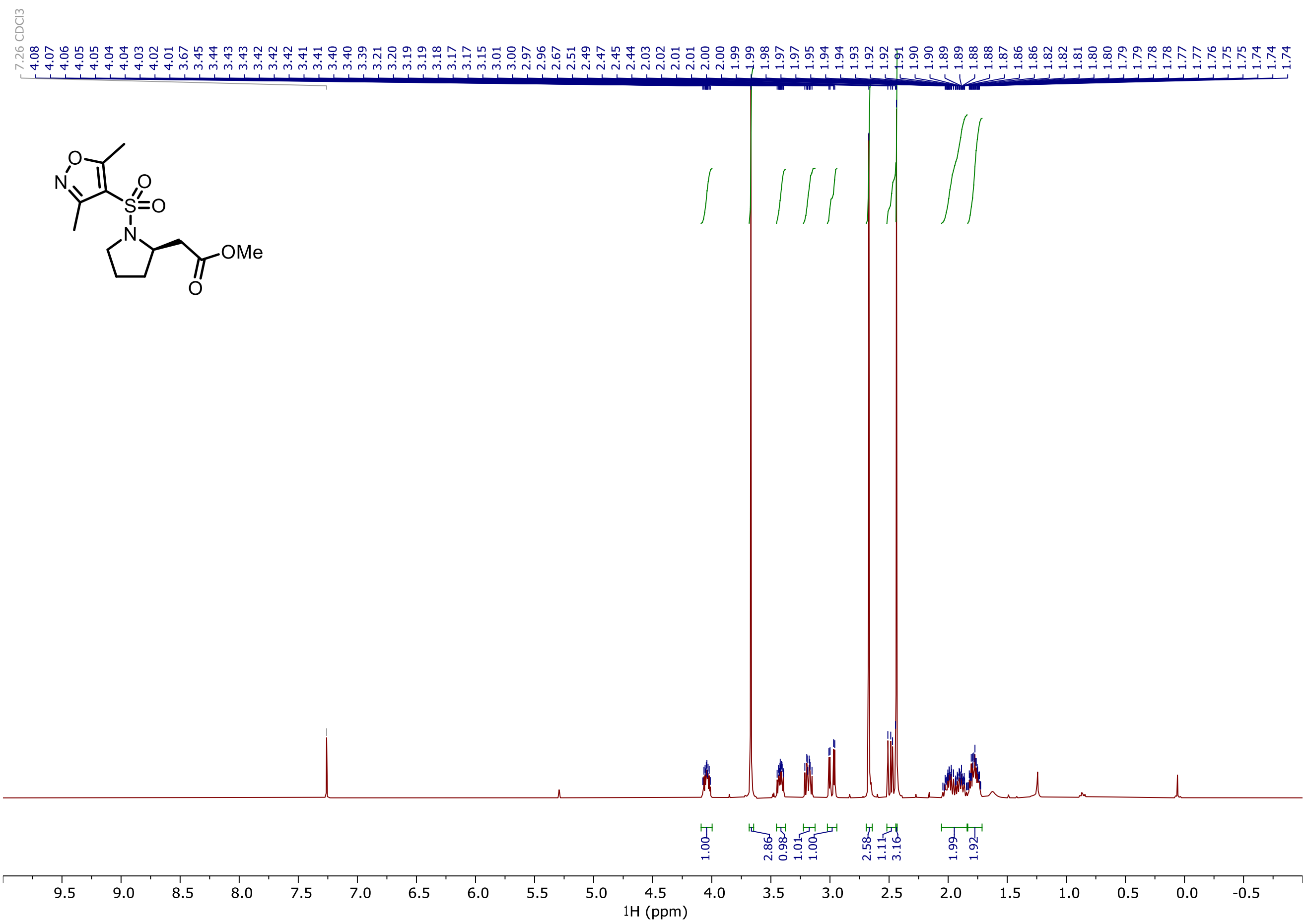

^{13}C NMR (101 MHz, $CDCl_3$) of compound **8j**

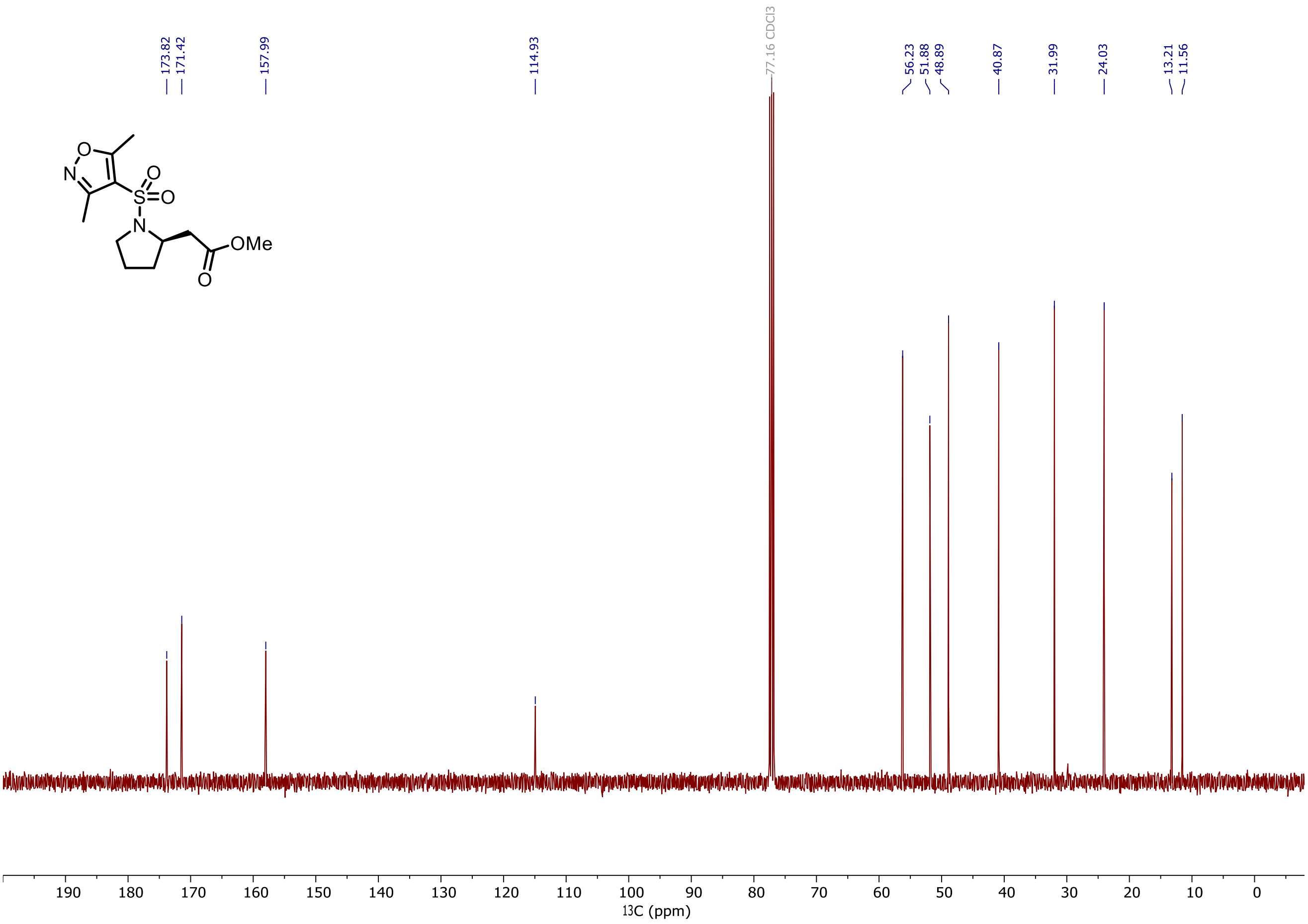

[back to table of contents]

^{1}H NMR (400 MHz, $CDCl_3$) of compound **8k**

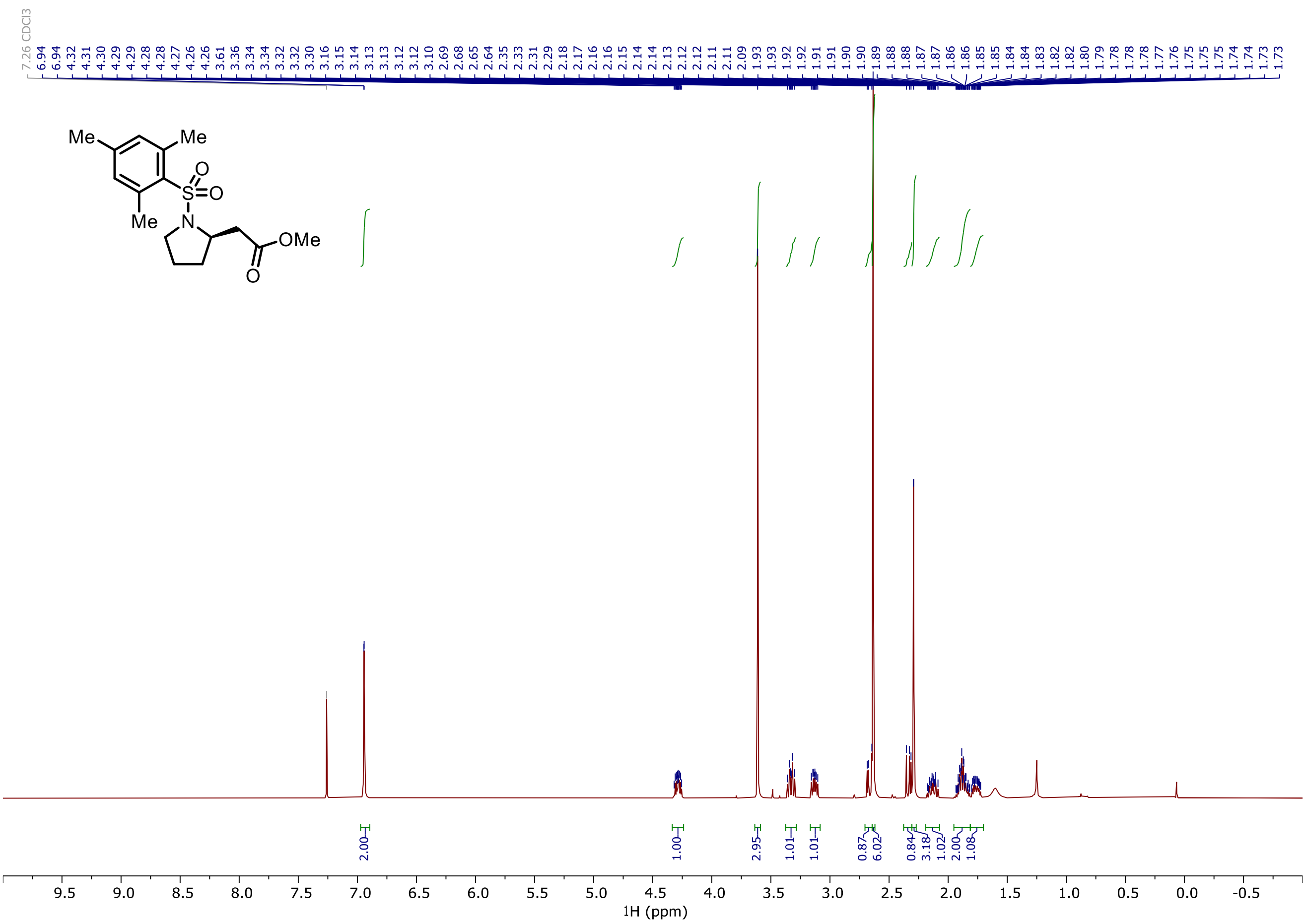

^{13}C NMR (101 MHz, $CDCl_3$) of compound **8k**

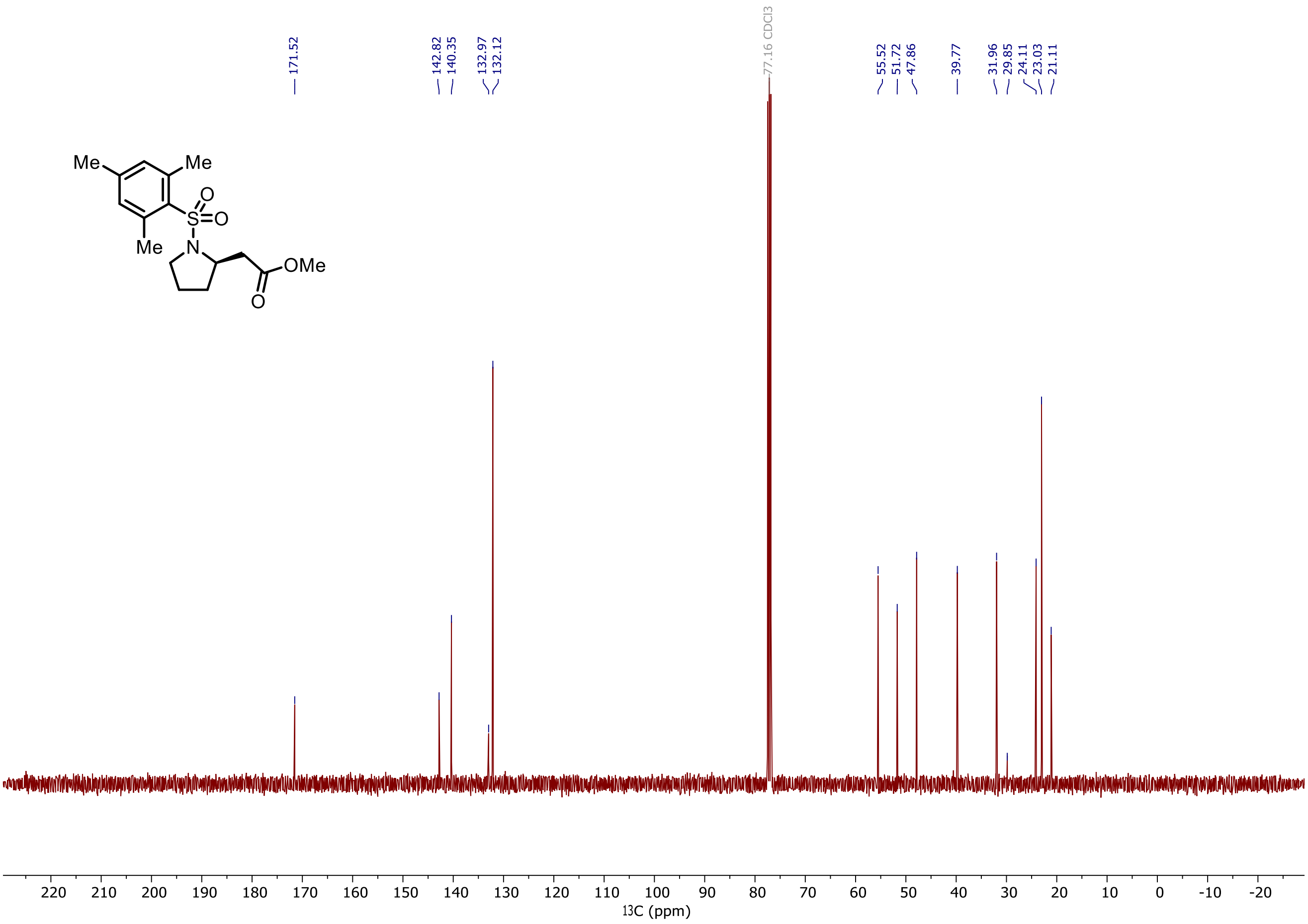

[back to table of contents]

^{1}H NMR (400 MHz, $CDCl_3$) of compound **81**

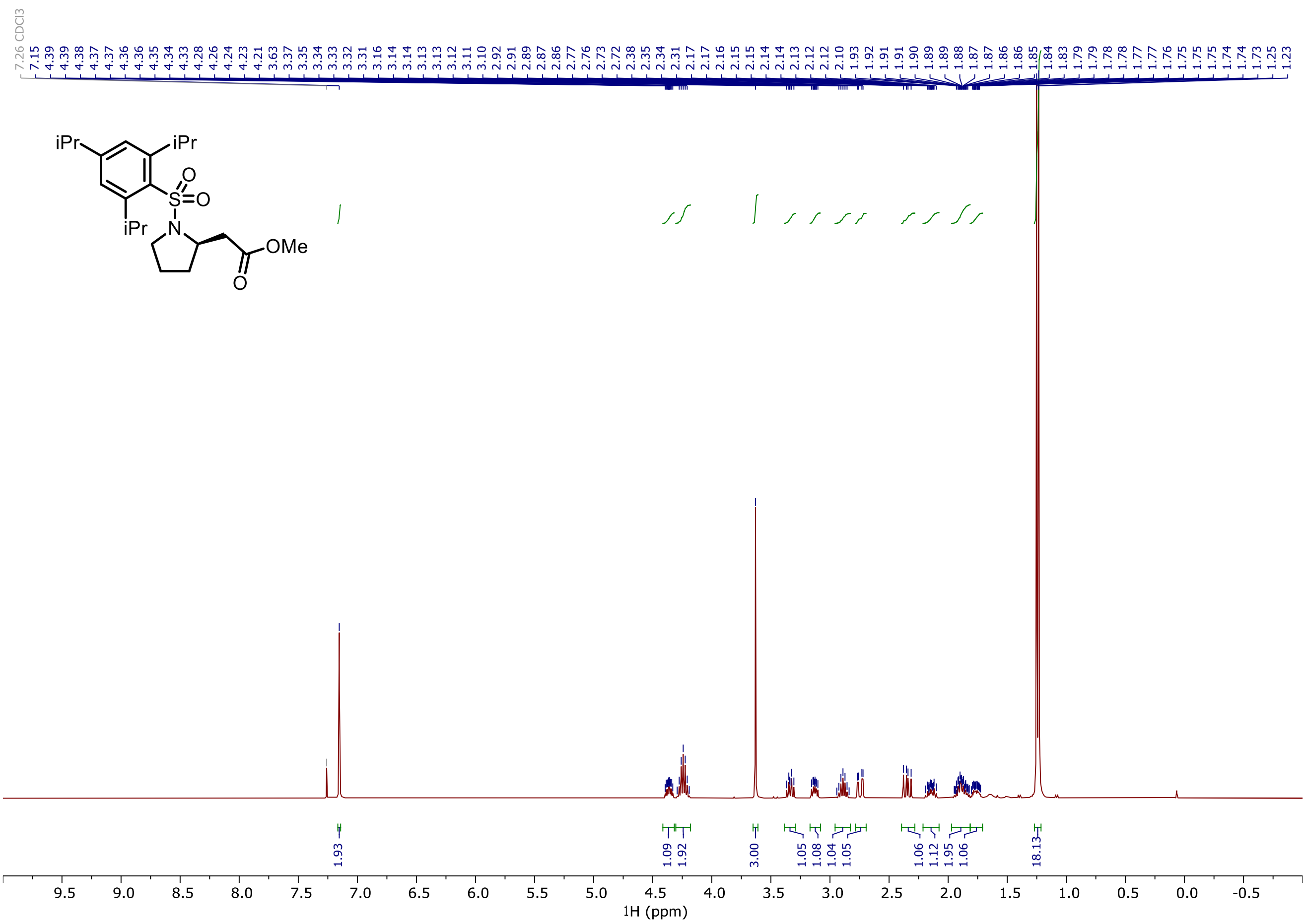

^{13}C NMR (101 MHz, $CDCl_3$) of compound **81**

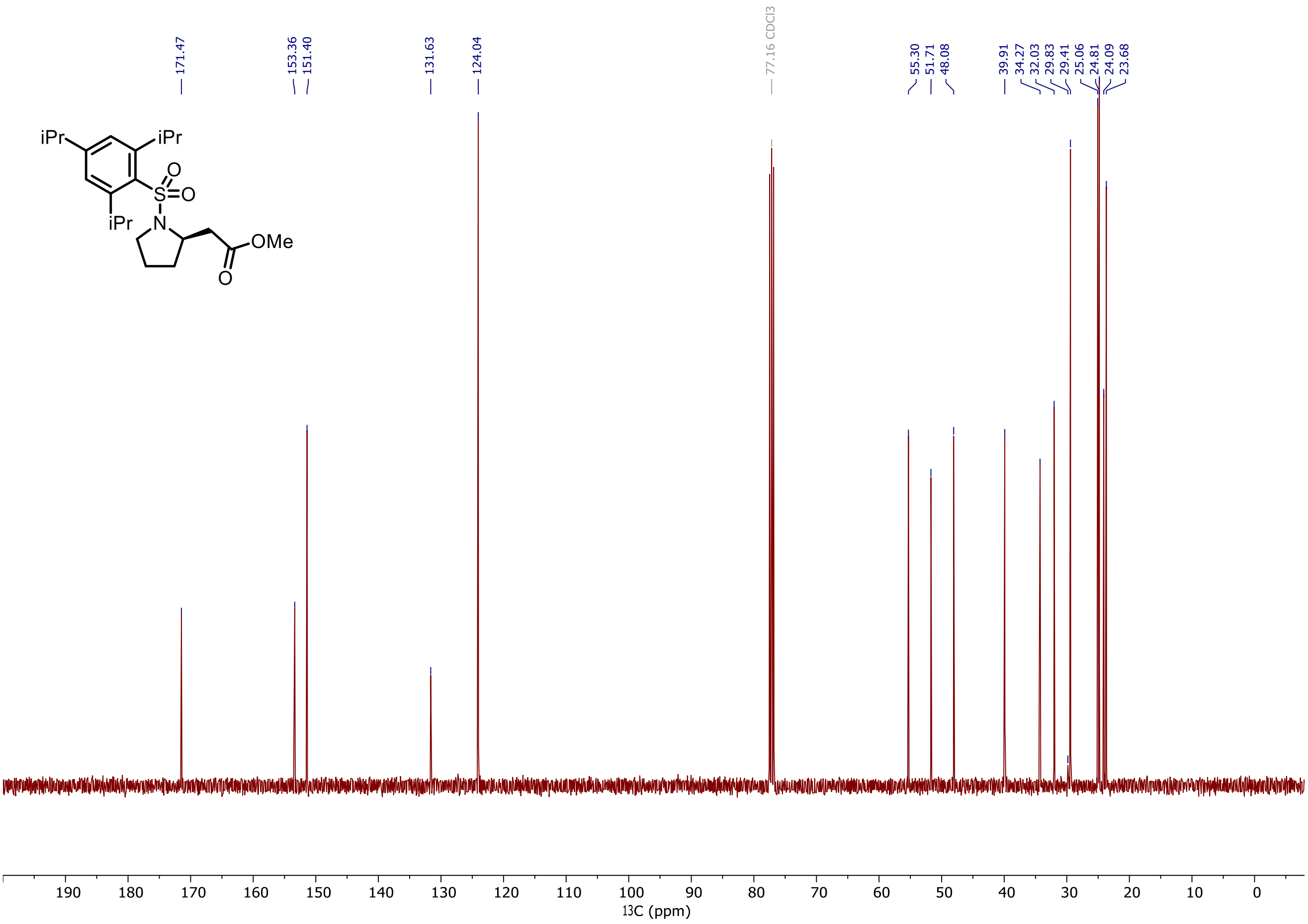

[back to table of contents]

^{1}H NMR (400 MHz, $CDCl_3$) of compound 8m

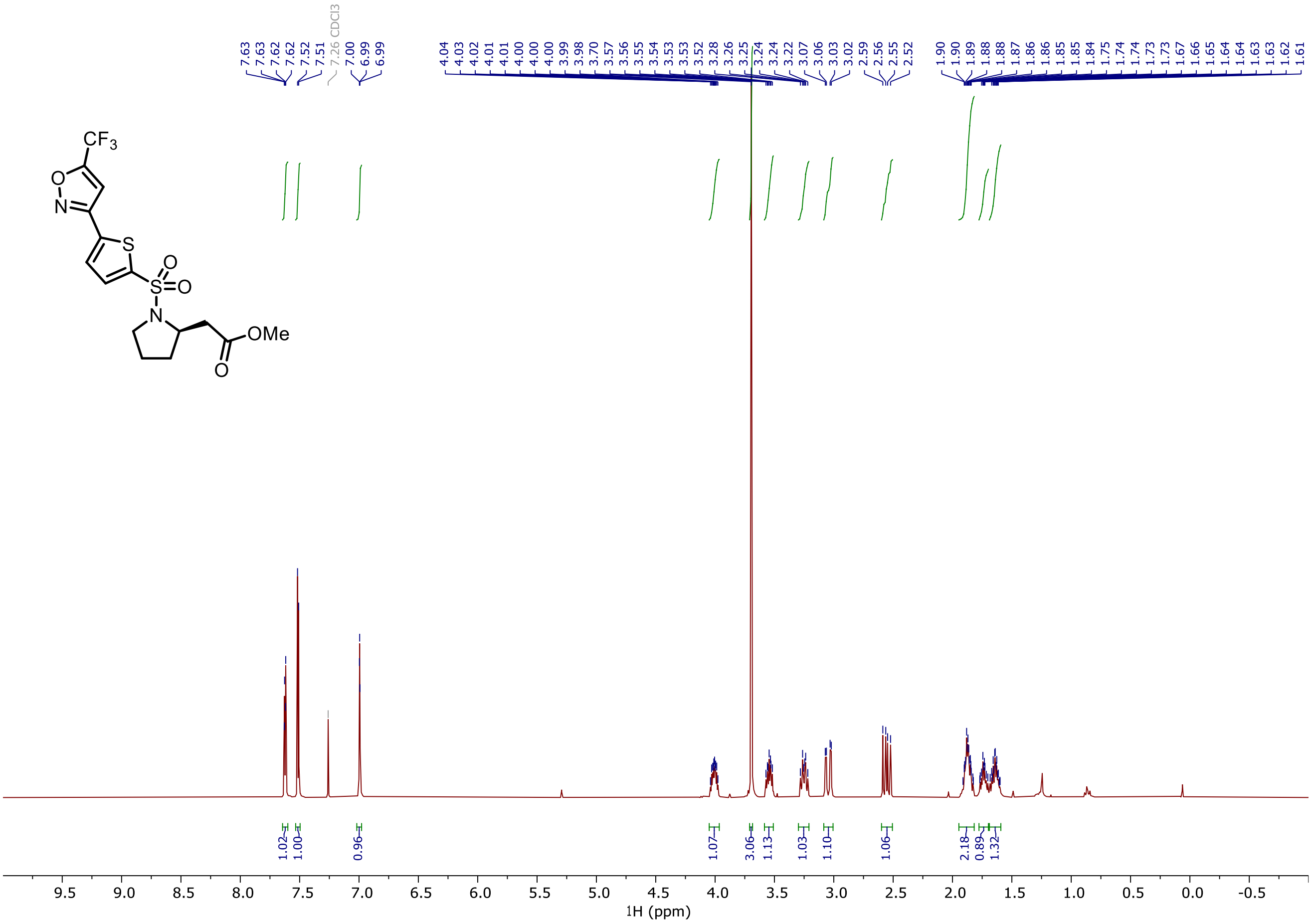

^{13}C NMR (101 MHz, $CDCl_3$) of compound 8m

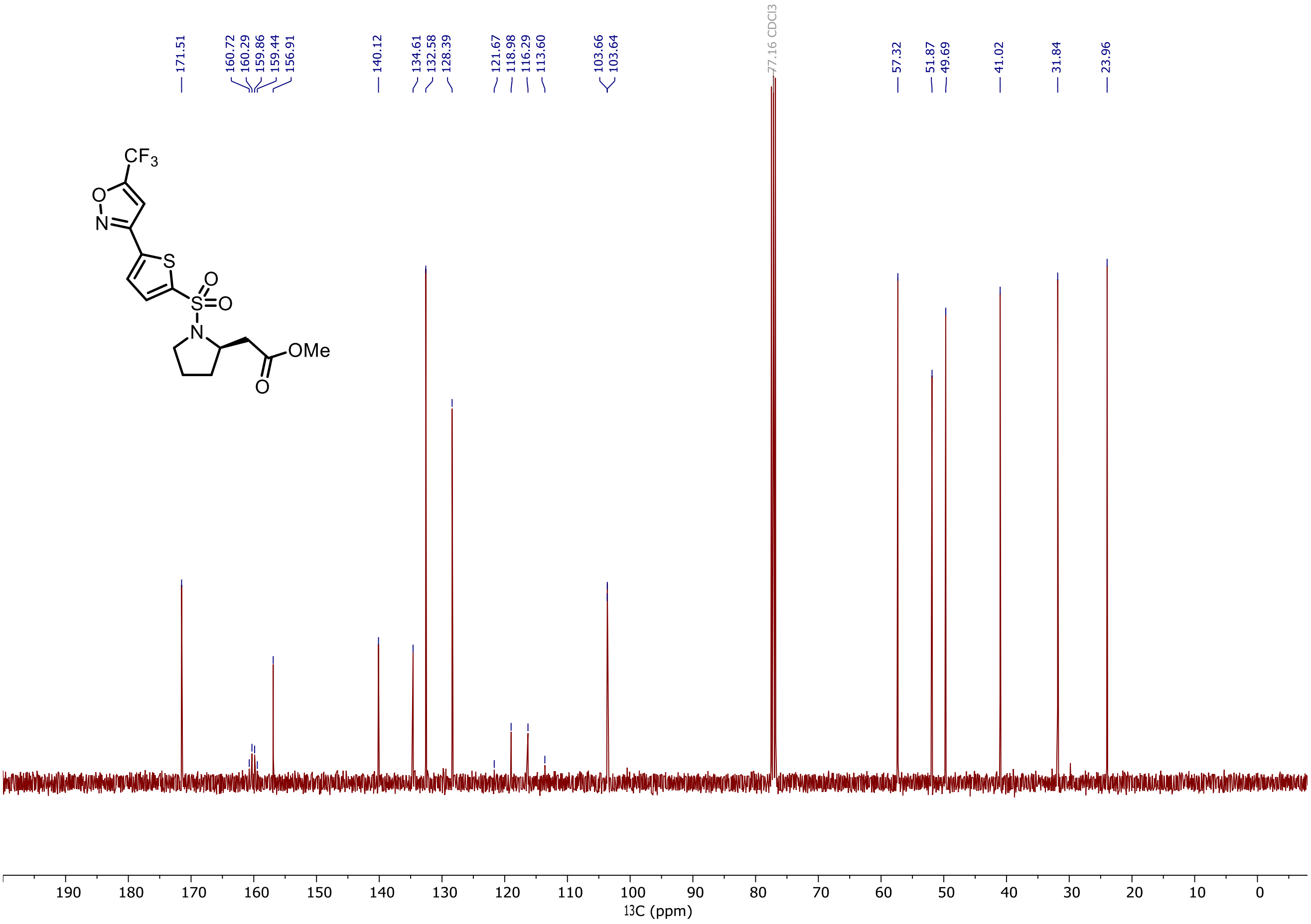

[back to table of contents]

^{19}F NMR (376 MHz, $CDCl_3$) of compound **8m** (contains PhF as internal standard, δ_F -113.15 ppm)

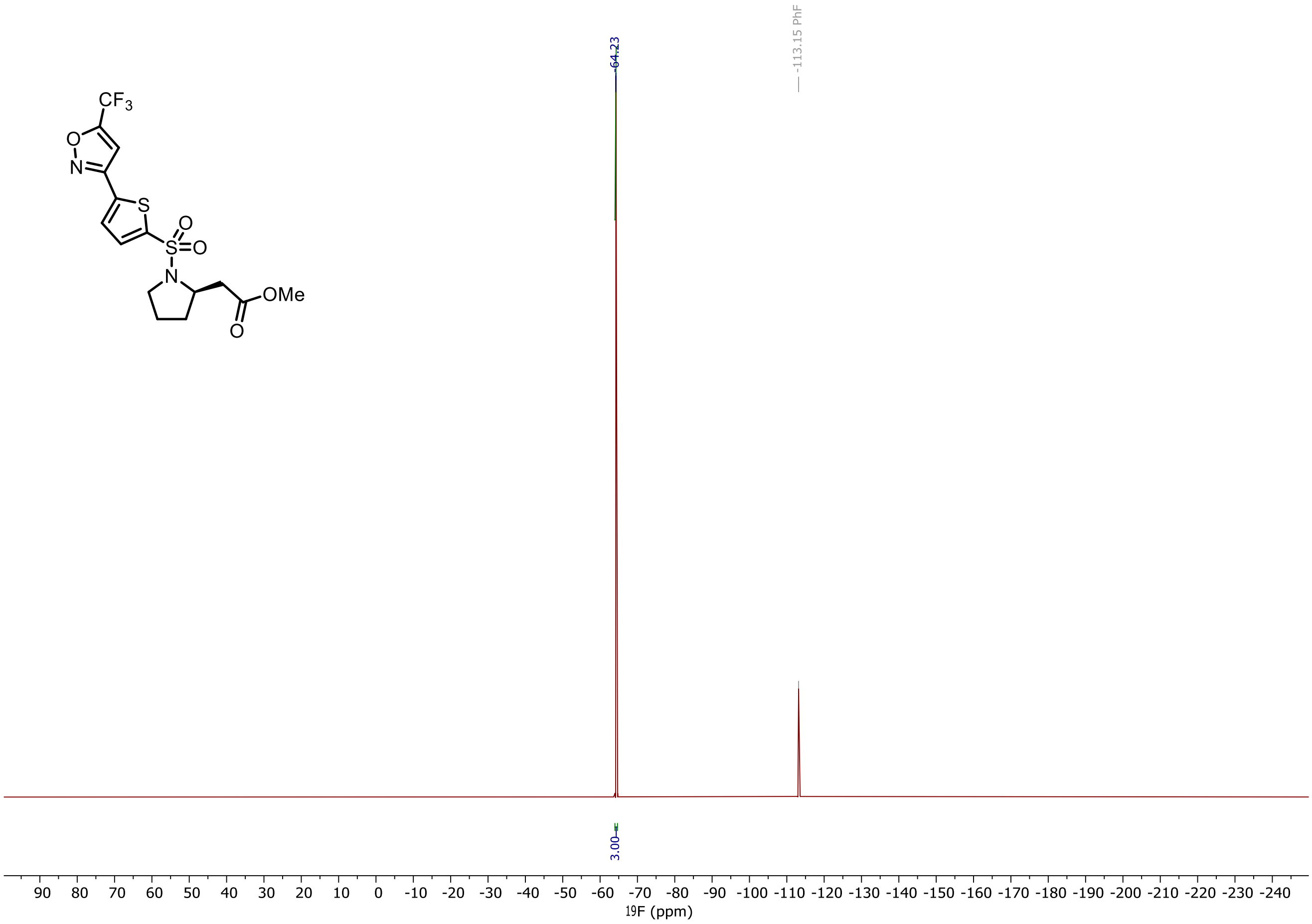

[back to table of contents]

^{1}H NMR (600 MHz, $CDCl_3$) of compound **8n**

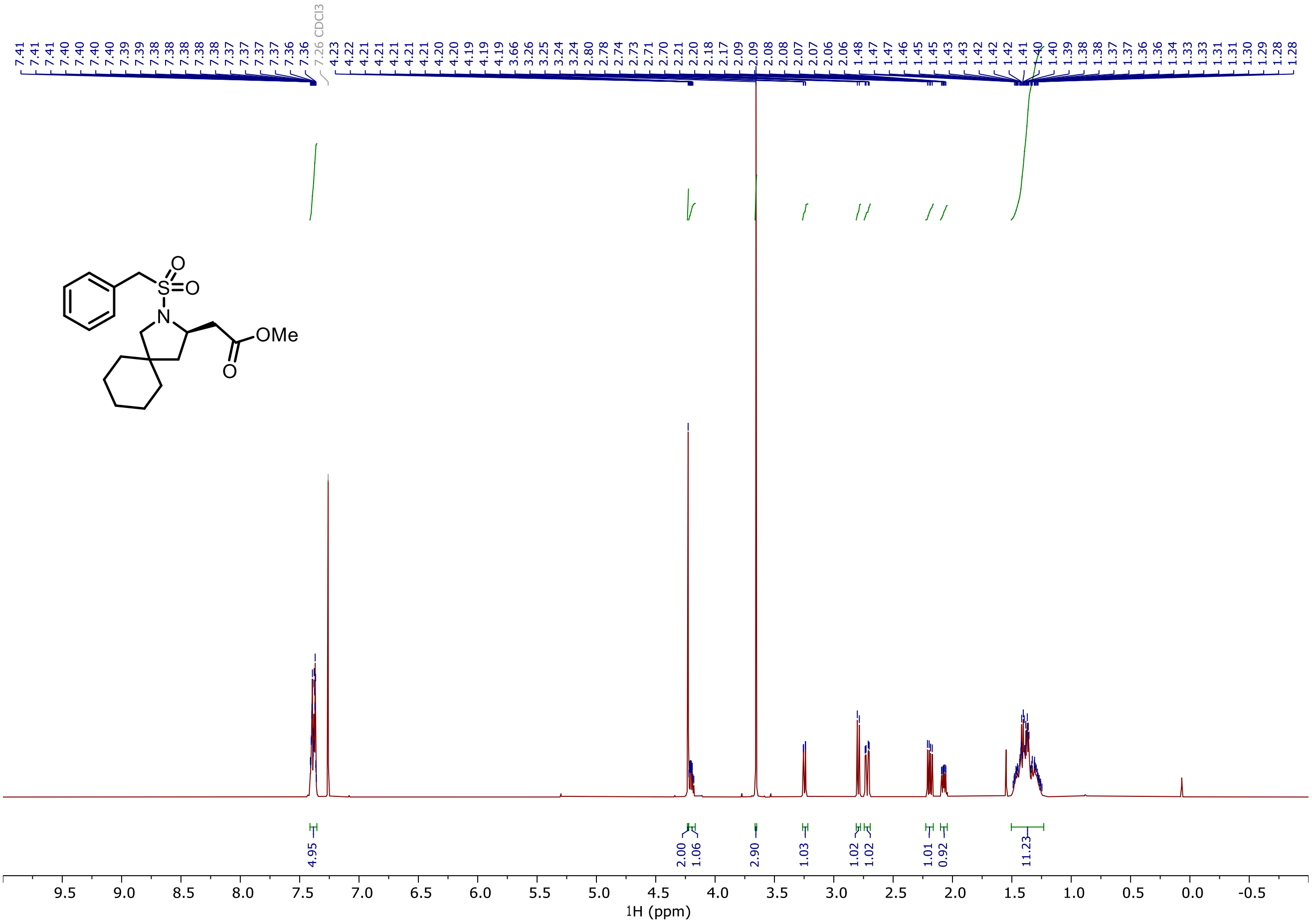

^{13}C NMR (151 MHz, $CDCl_3$) of compound **8n**

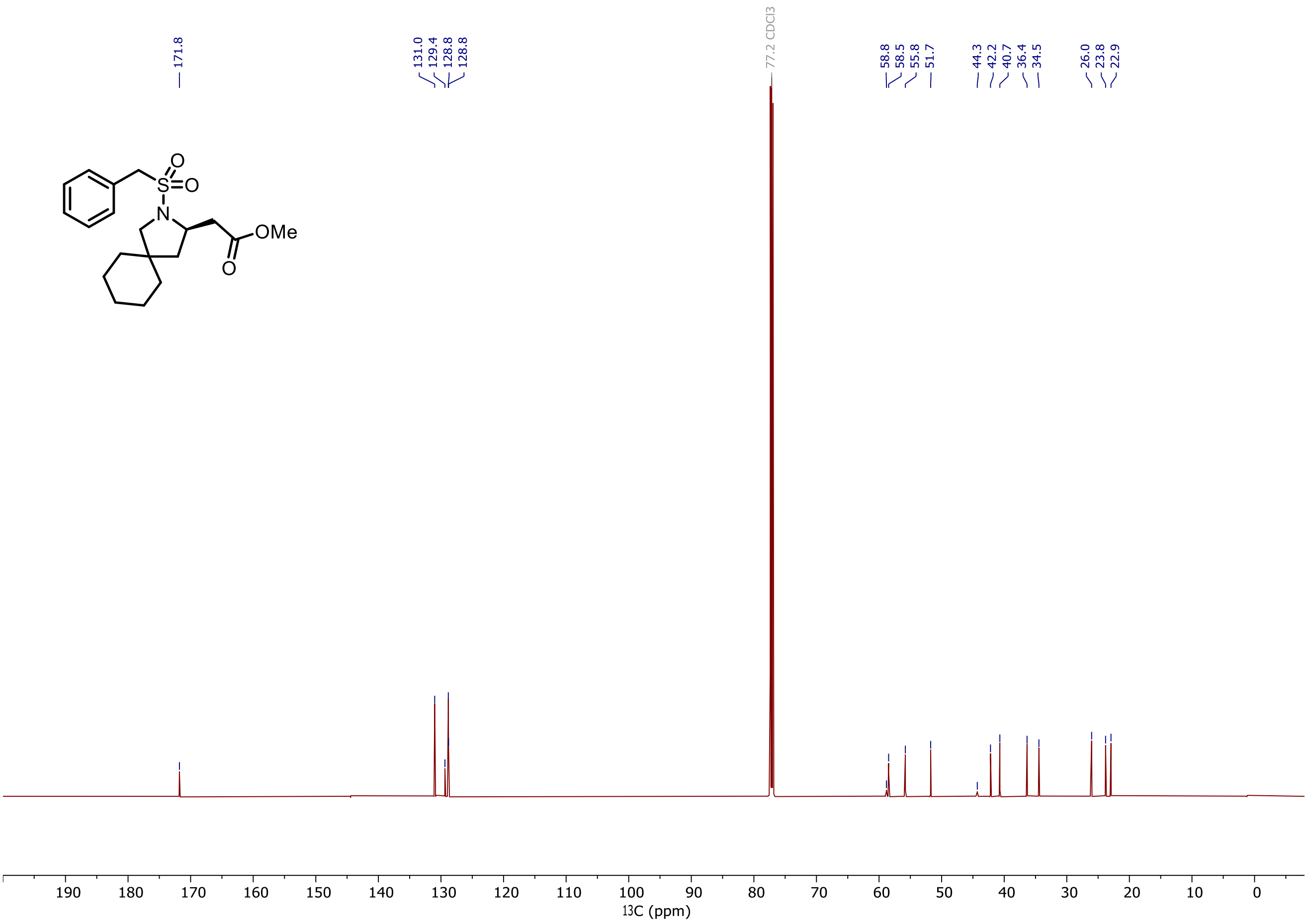

[back to table of contents]

^{1}H NMR (600 MHz, CDCl$_3$) of compound **8o**

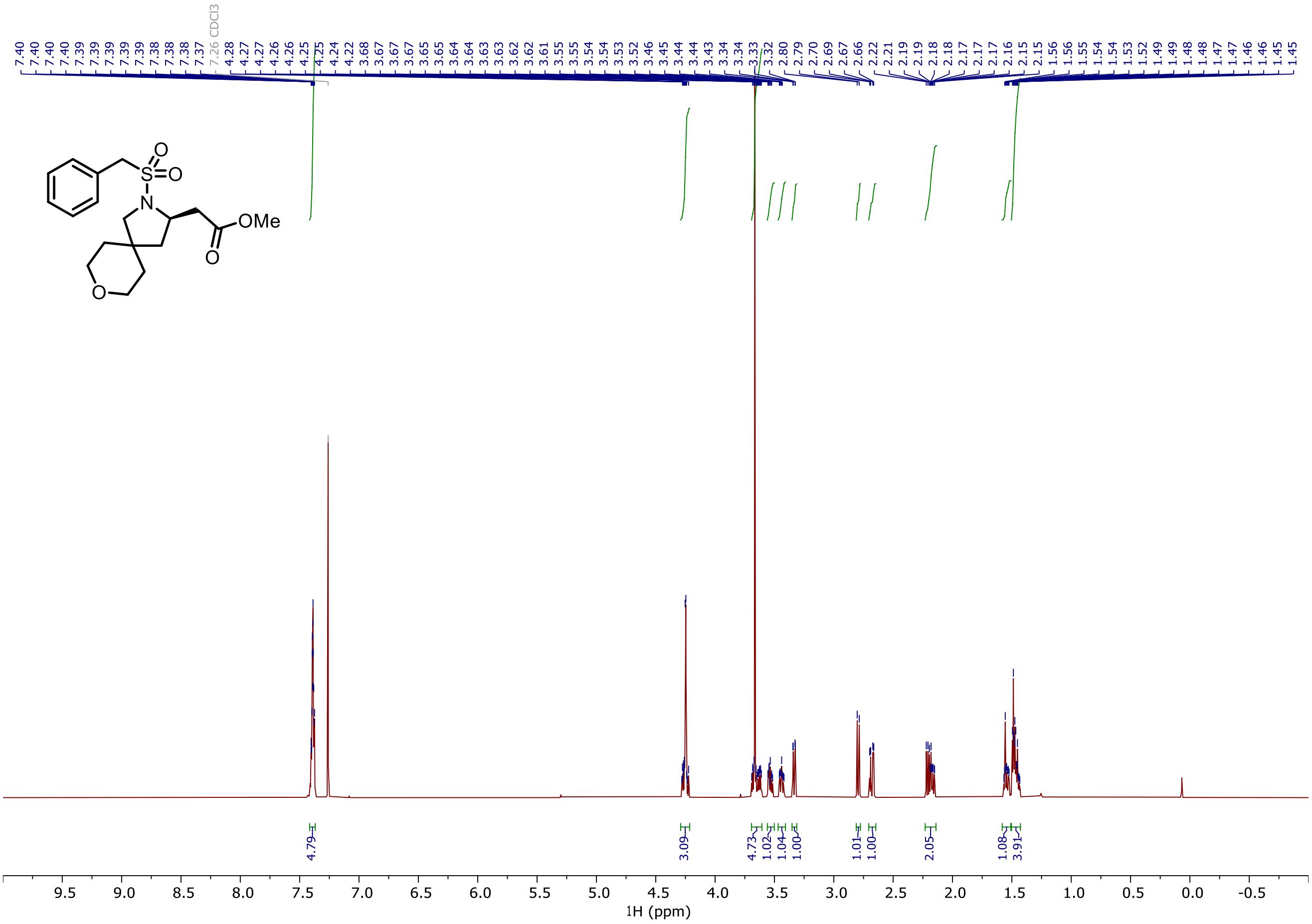

^{13}C NMR (151 MHz, CDCl$_3$) of compound **8o**

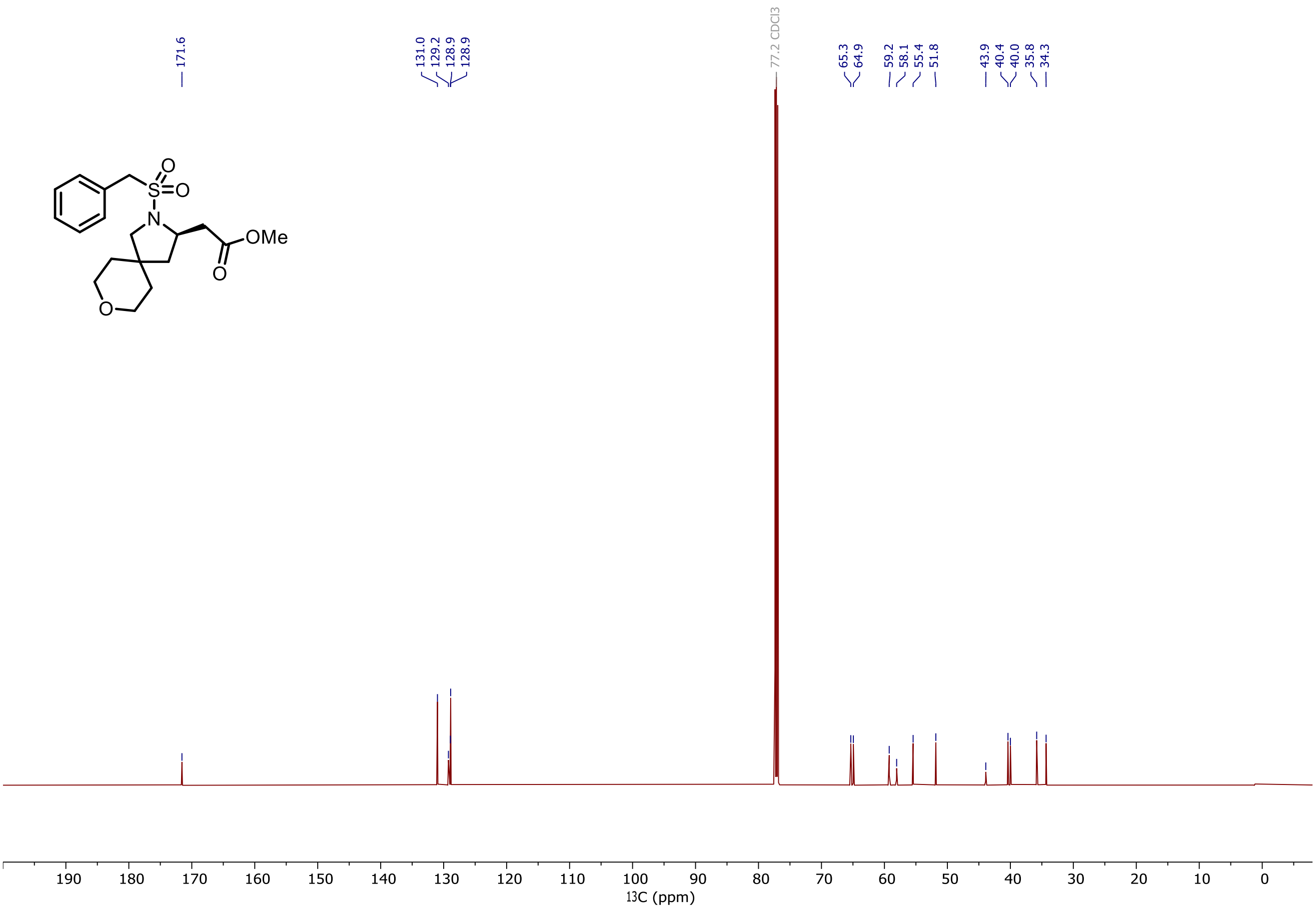

[back to table of contents]

^{1}H NMR (600 MHz, DMSO, 373 K) of compound **8p**

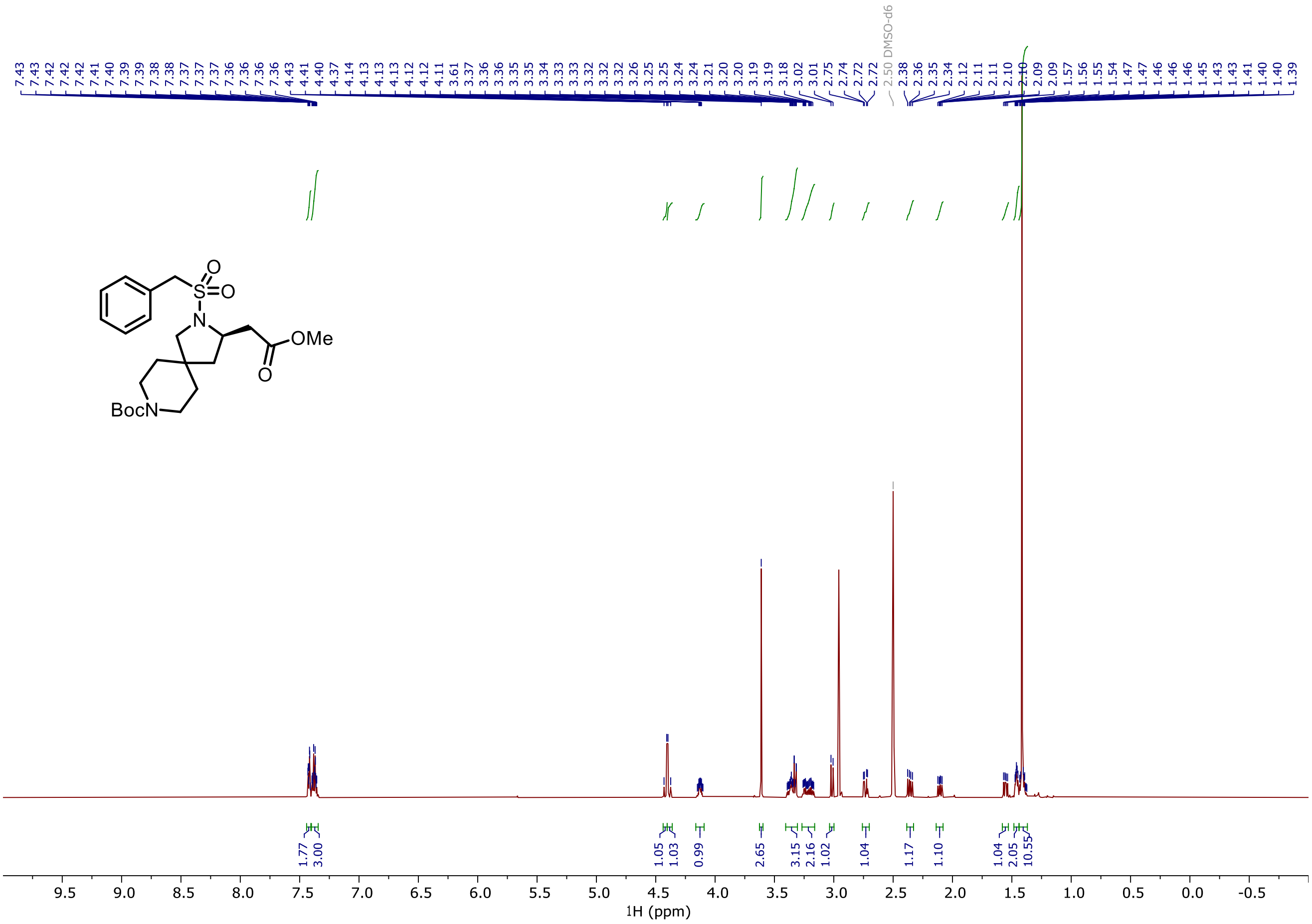

^{13}C NMR (151 MHz, DMSO, 373 K) of compound **8p**

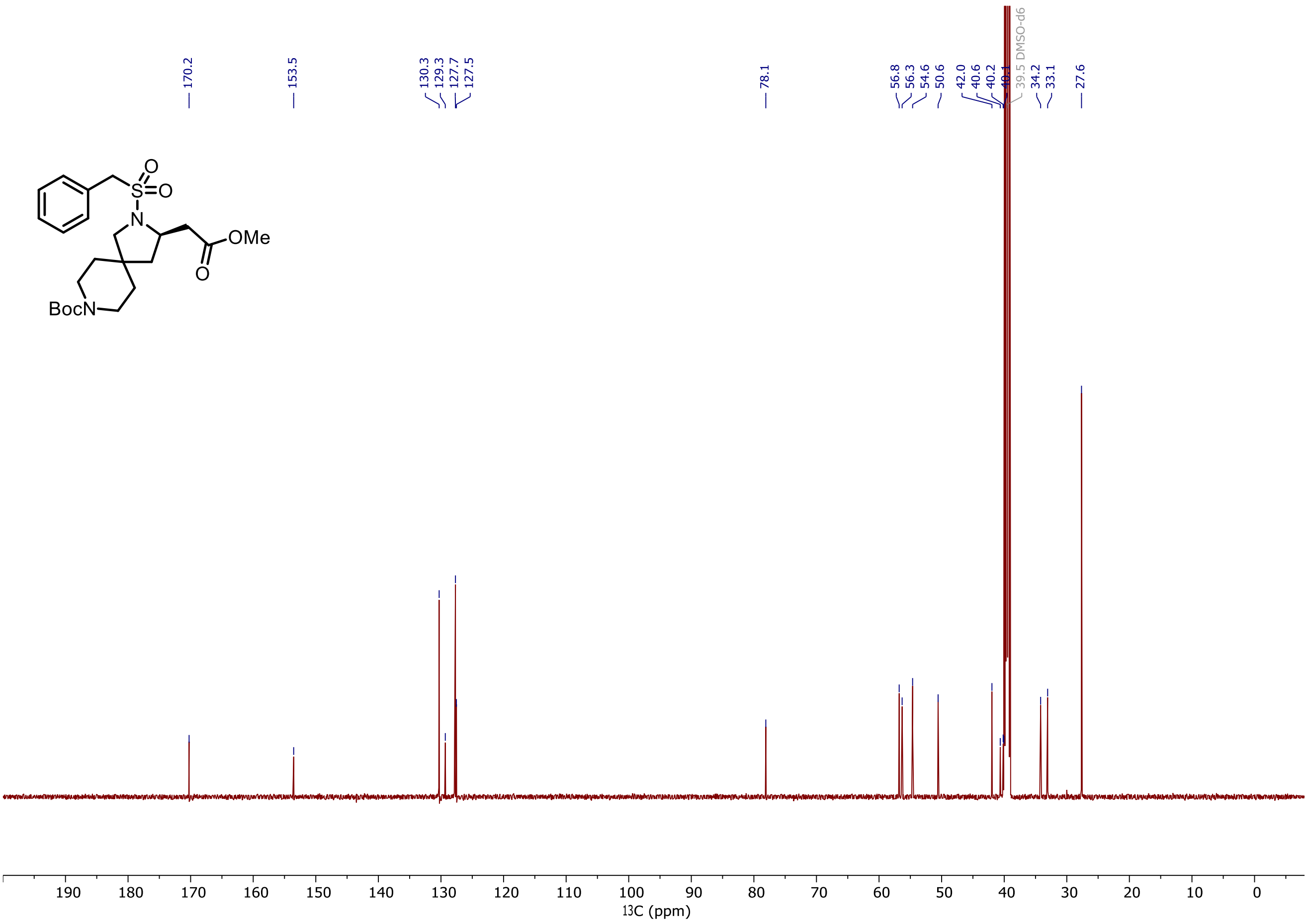

[back to table of contents]

¹H NMR (600 MHz, $CDCl_3$) of compound **8q**

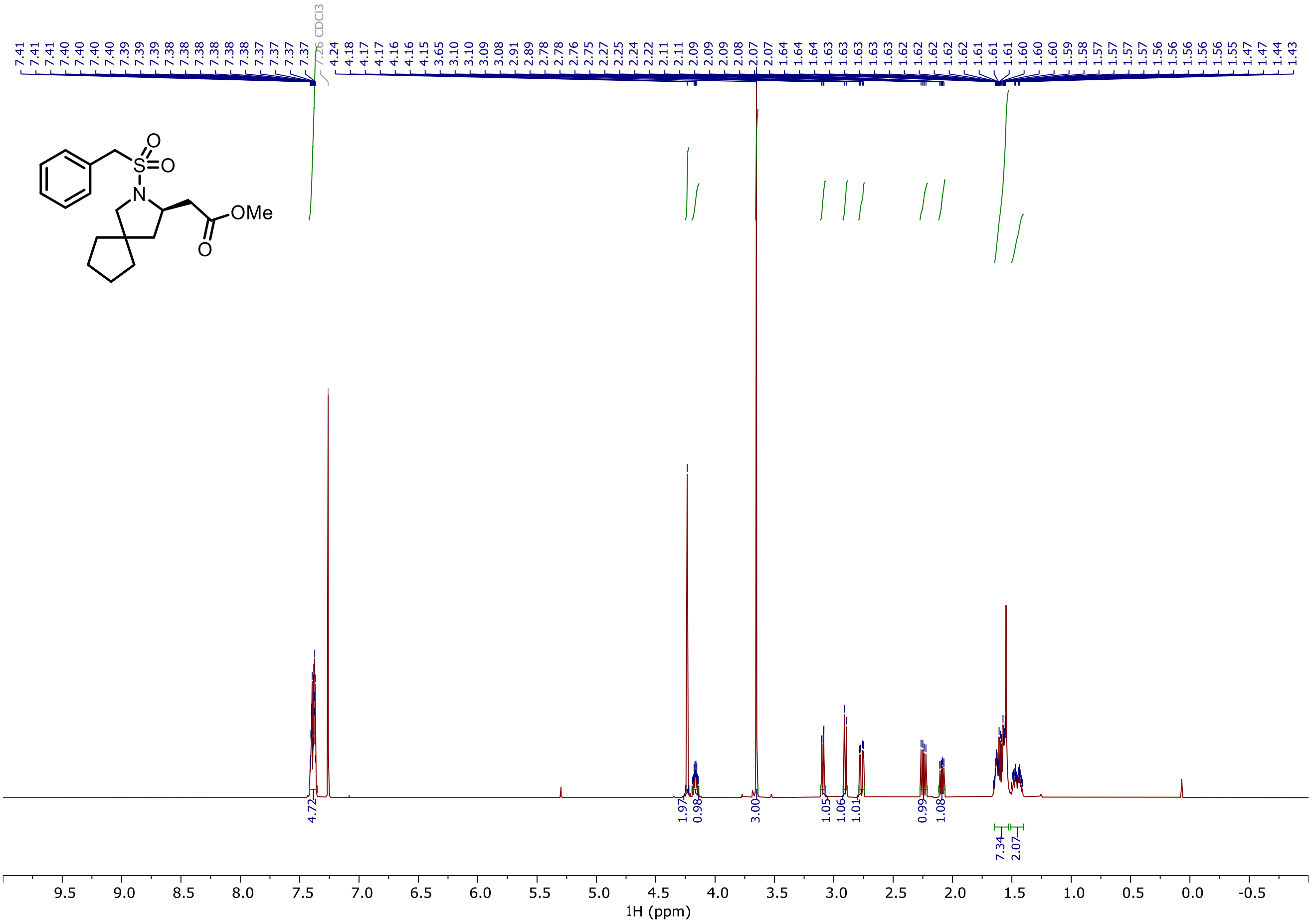

¹³C NMR (151 MHz, $CDCl_3$) of compound **8q**

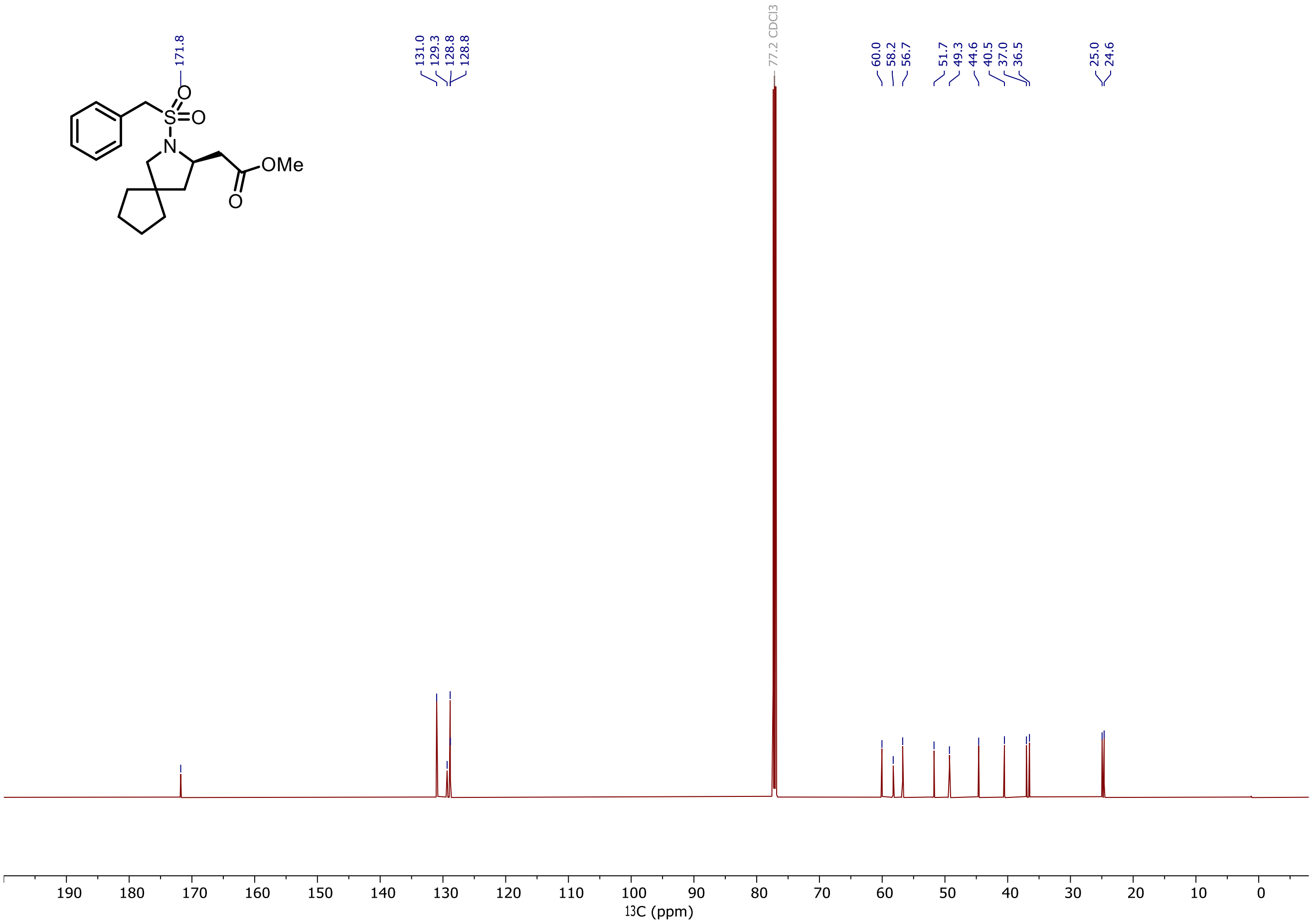

[back to table of contents]

^{1}H NMR (600 MHz, $CDCl_3$) of compound **8r**

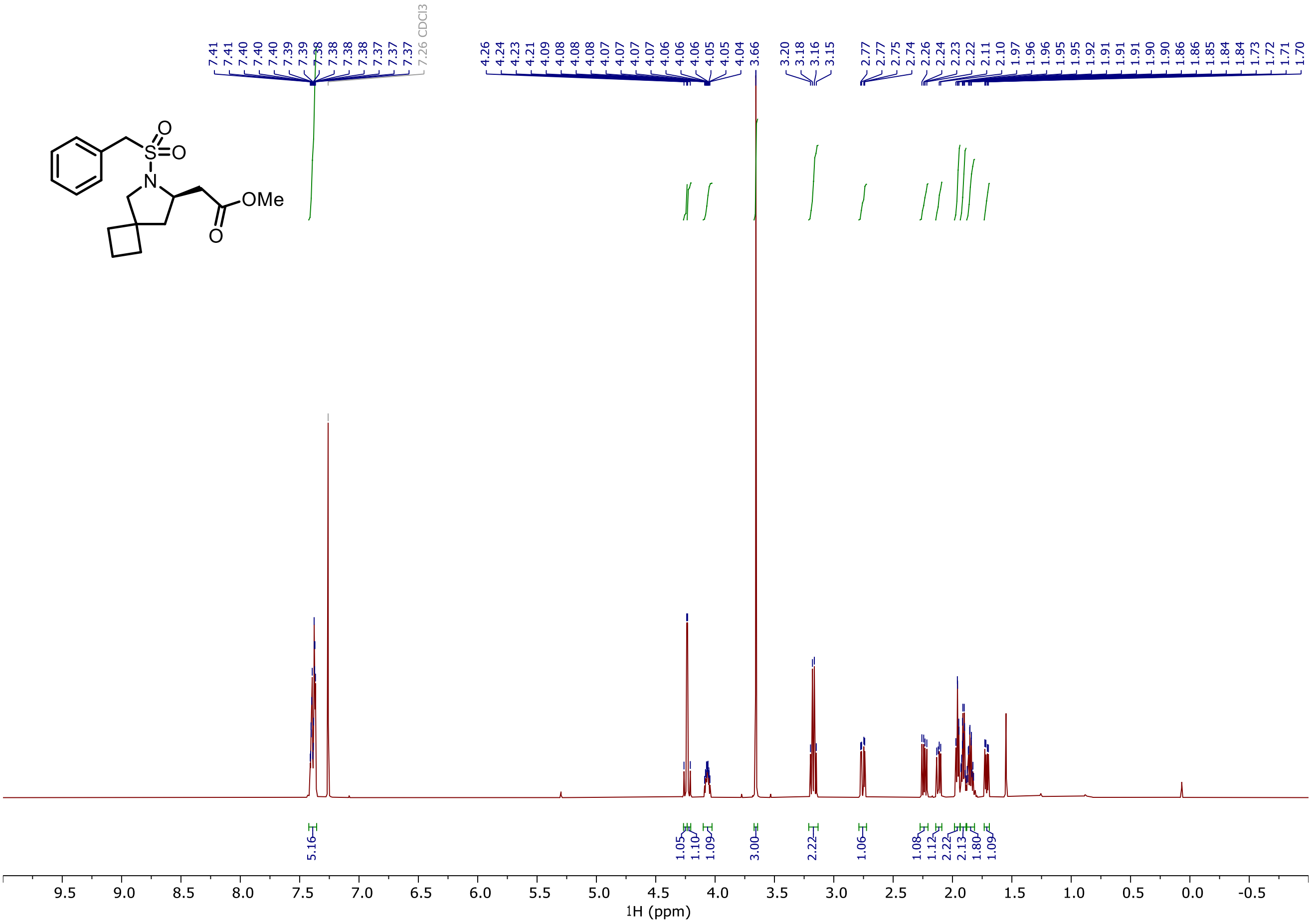

^{13}C NMR (151 MHz, $CDCl_3$) of compound **8r**

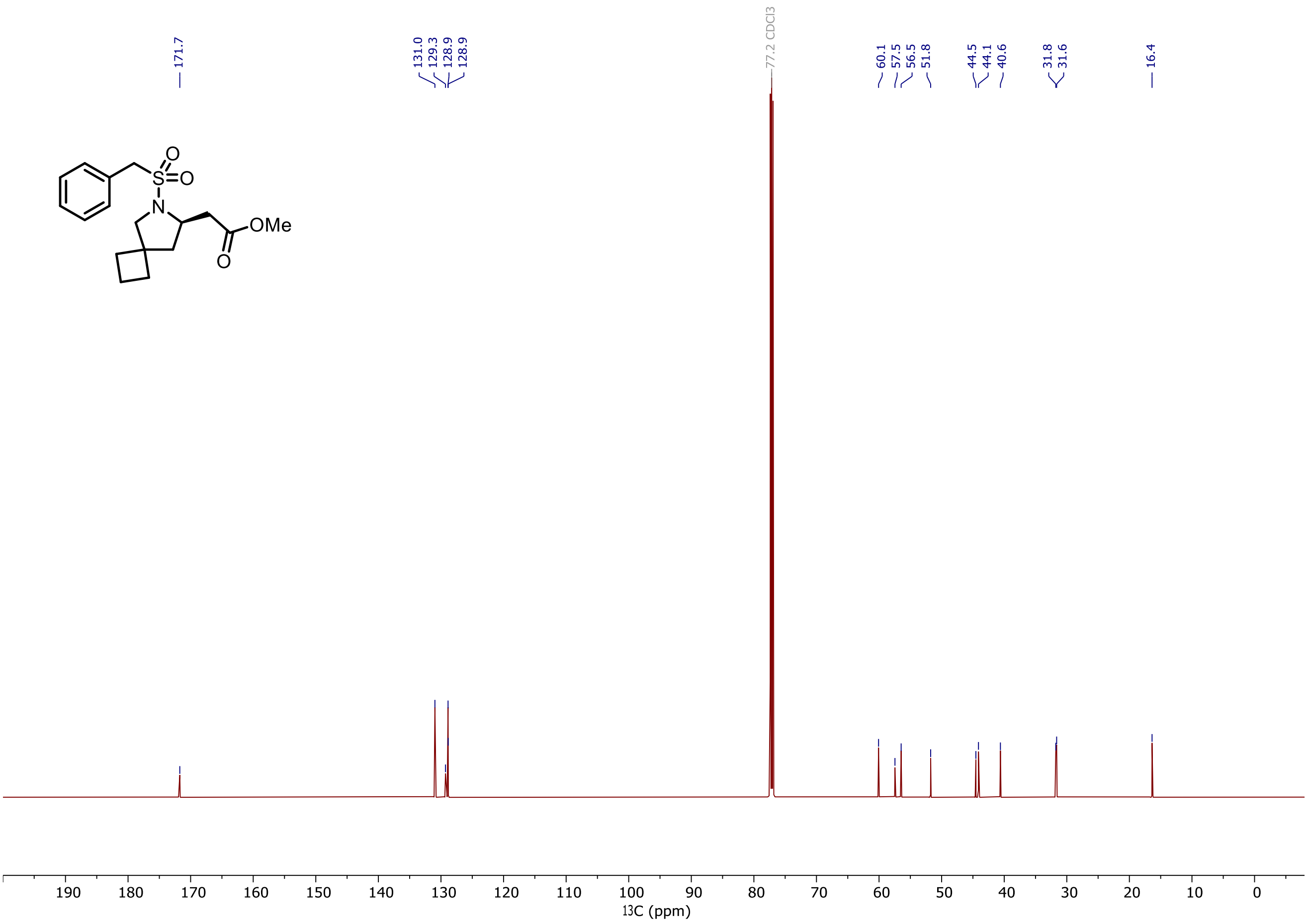

[back to table of contents]

^{1}H NMR (600 MHz, $CDCl_3$) of compound **8s**

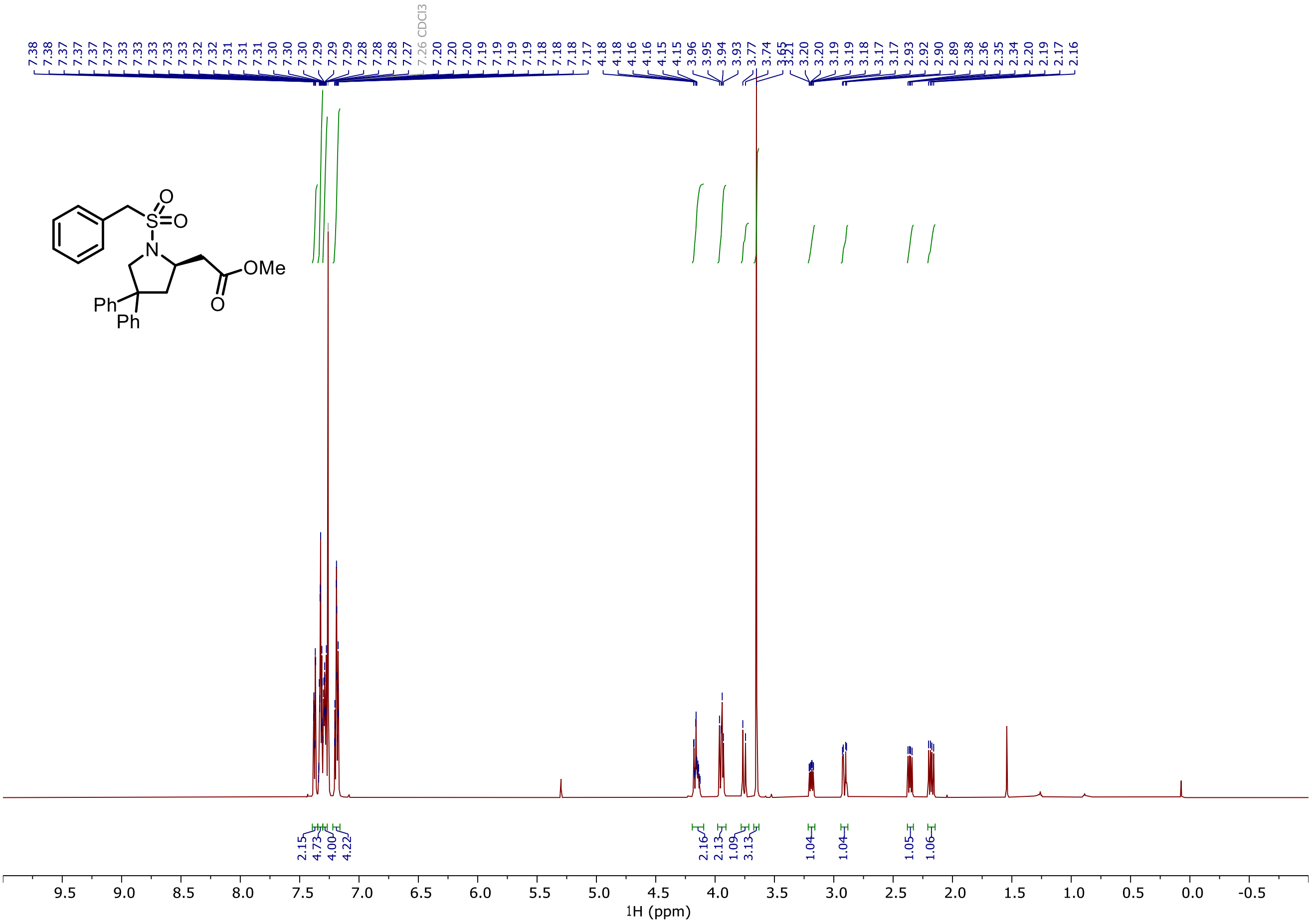

^{13}C NMR (151 MHz, $CDCl_3$) of compound **8s**

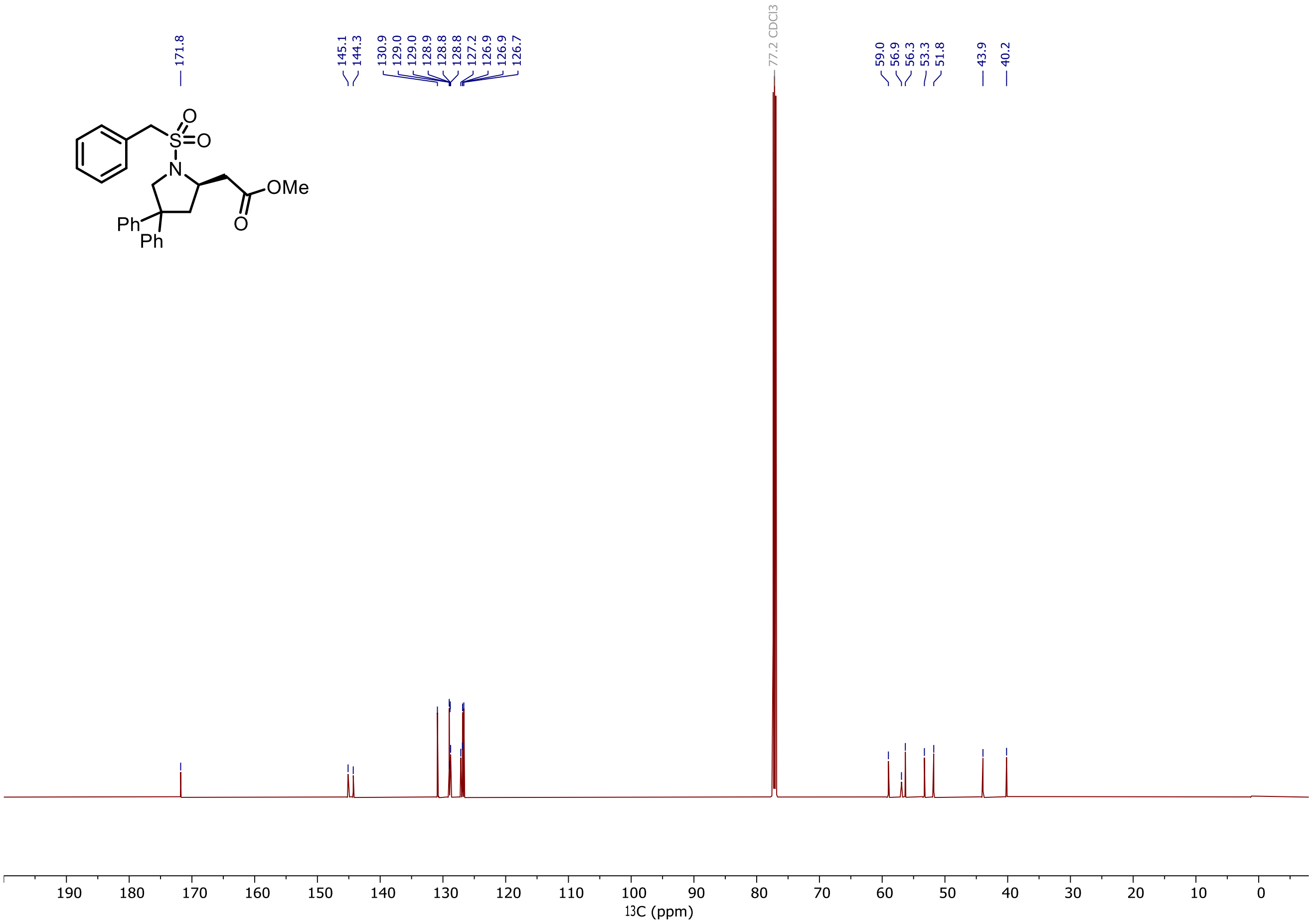

[back to table of contents]

^{1}H NMR (400 MHz, $CDCl_3$) of compound **8t**

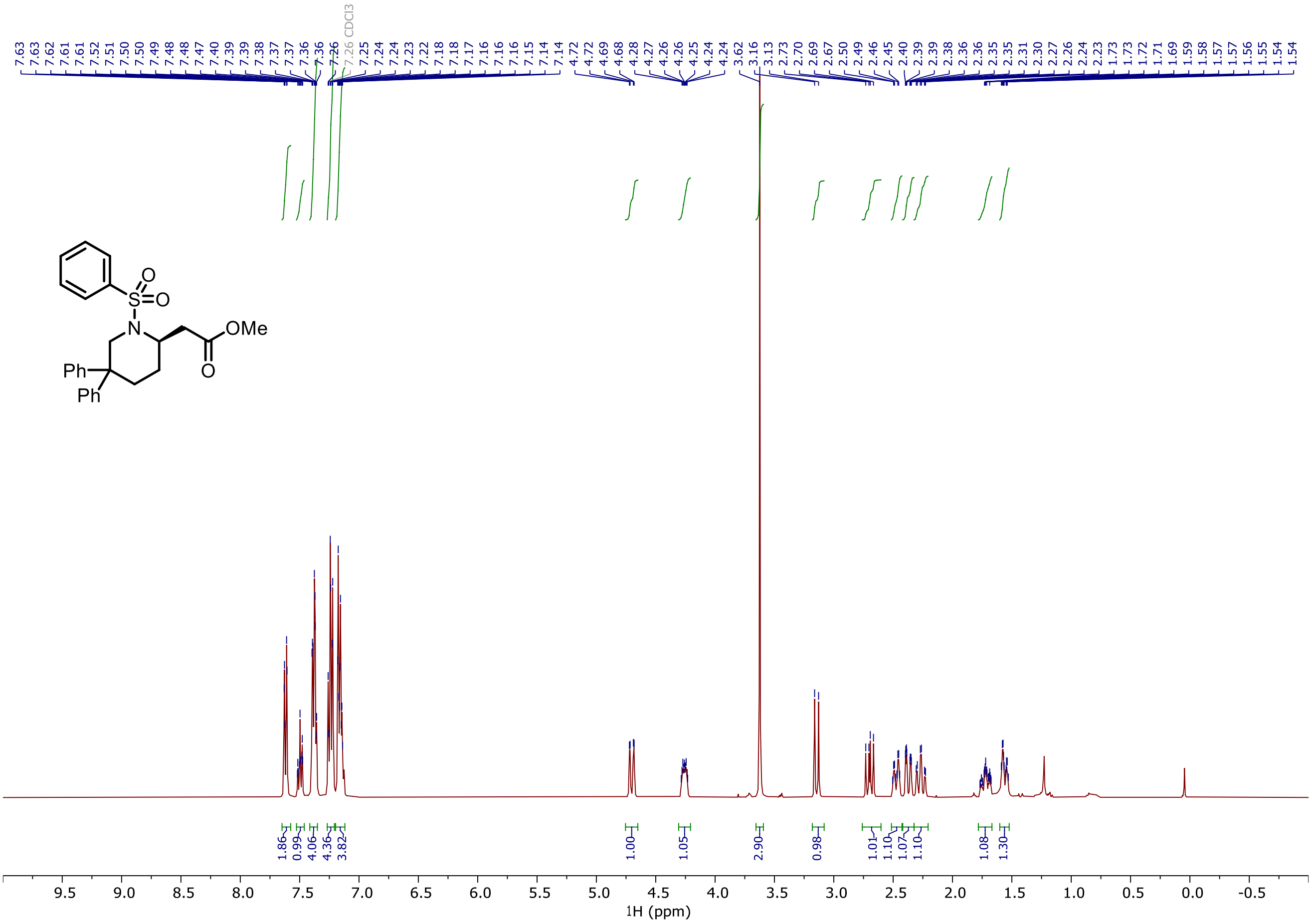

^{13}C NMR (101 MHz, $CDCl_3$) of compound **8t**

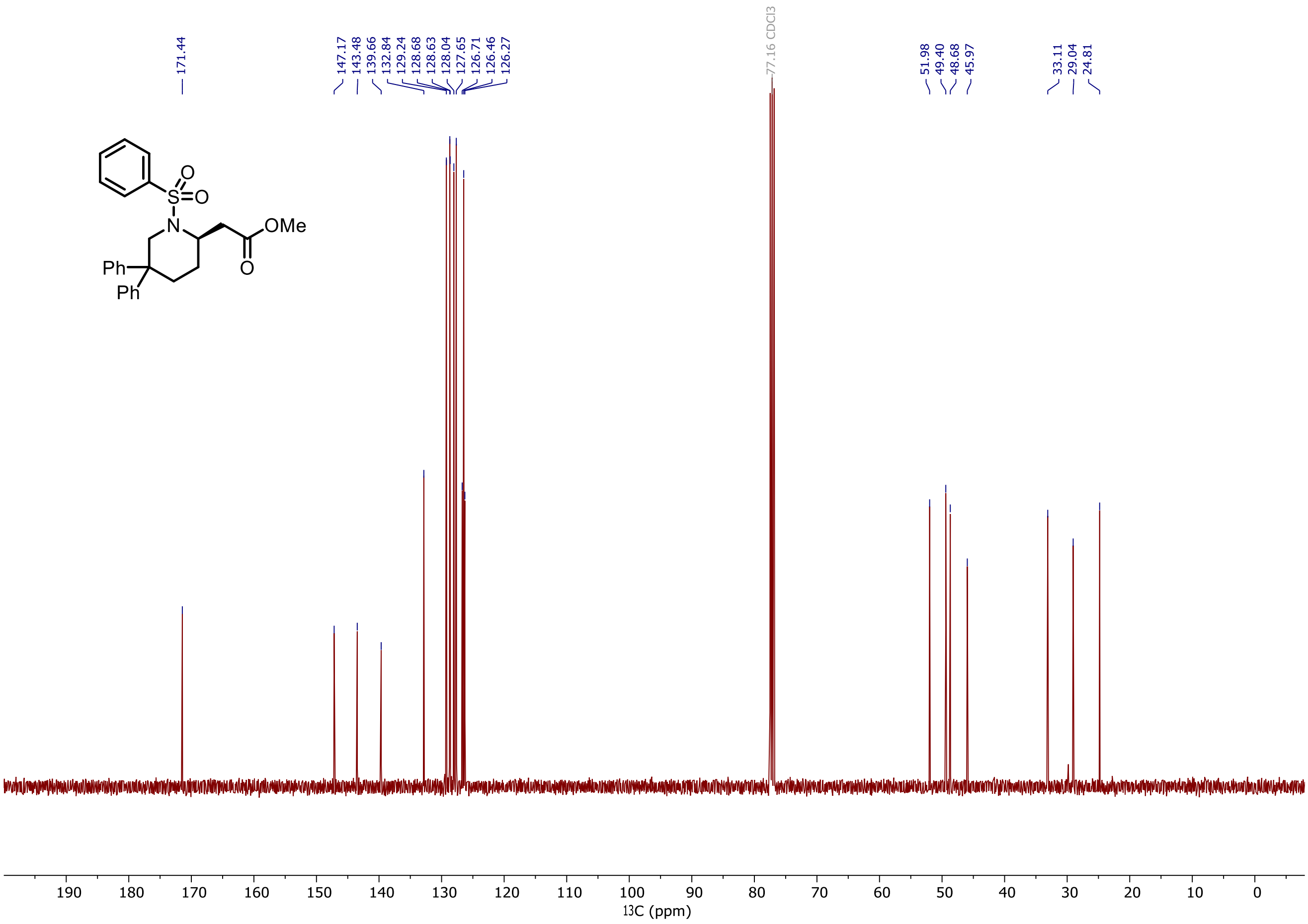

[back to table of contents]

^{1}H NMR (600 MHz, $CDCl_3$) of compound **8u**

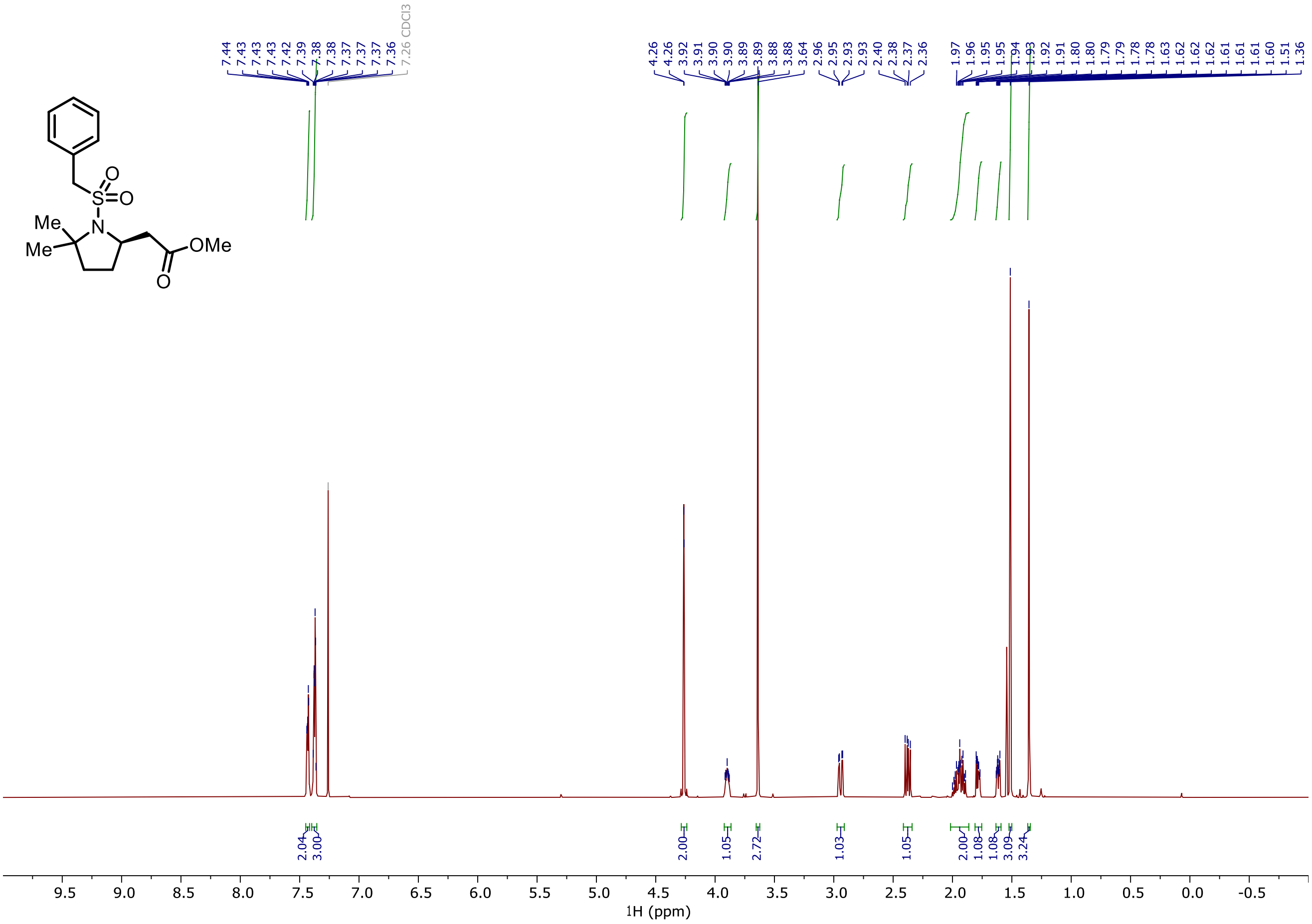

^{13}C NMR (151 MHz, $CDCl_3$) of compound **8u**

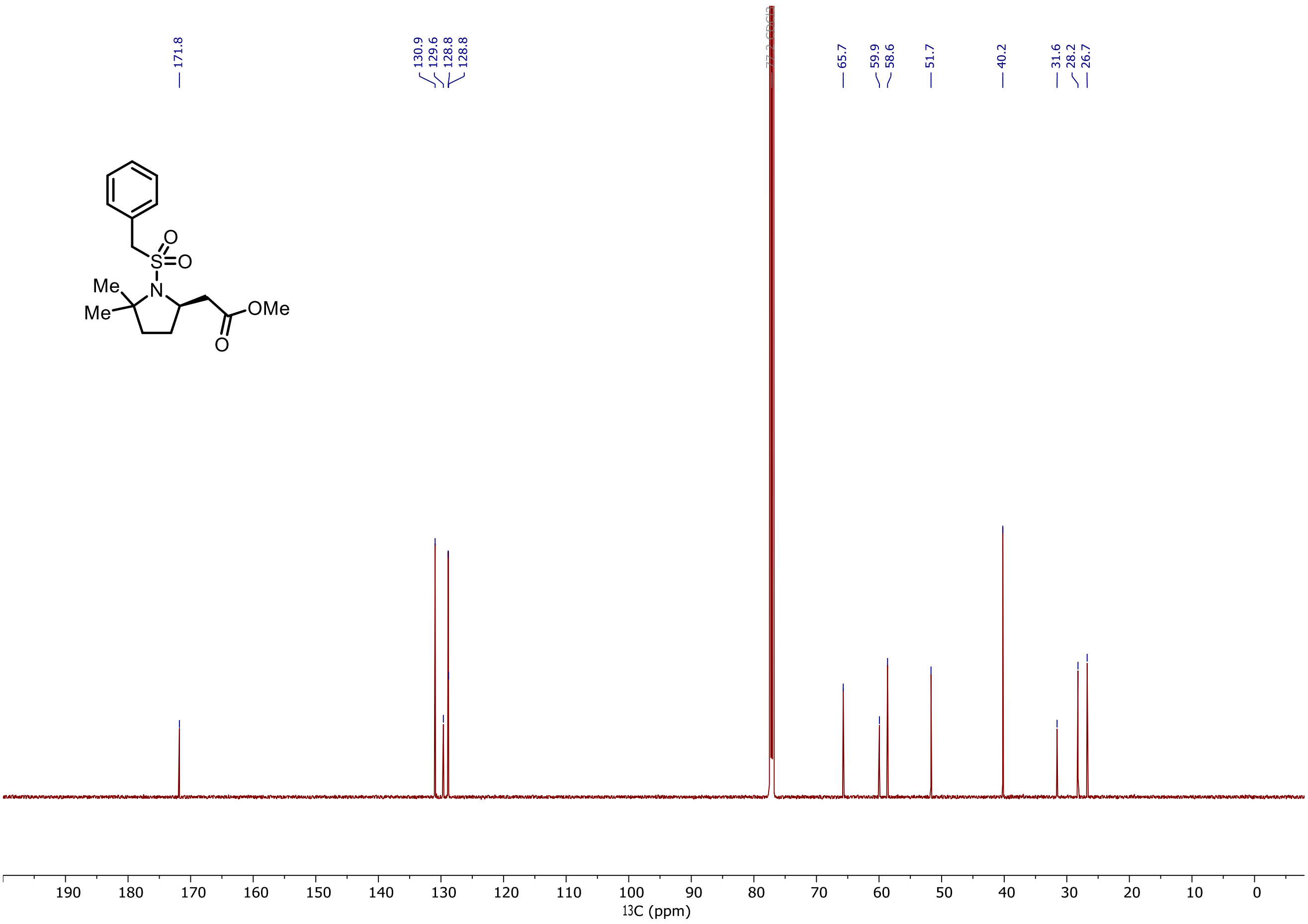

[back to table of contents]

^{1}H NMR (600 MHz, $CDCl_3$) of compound **8v**

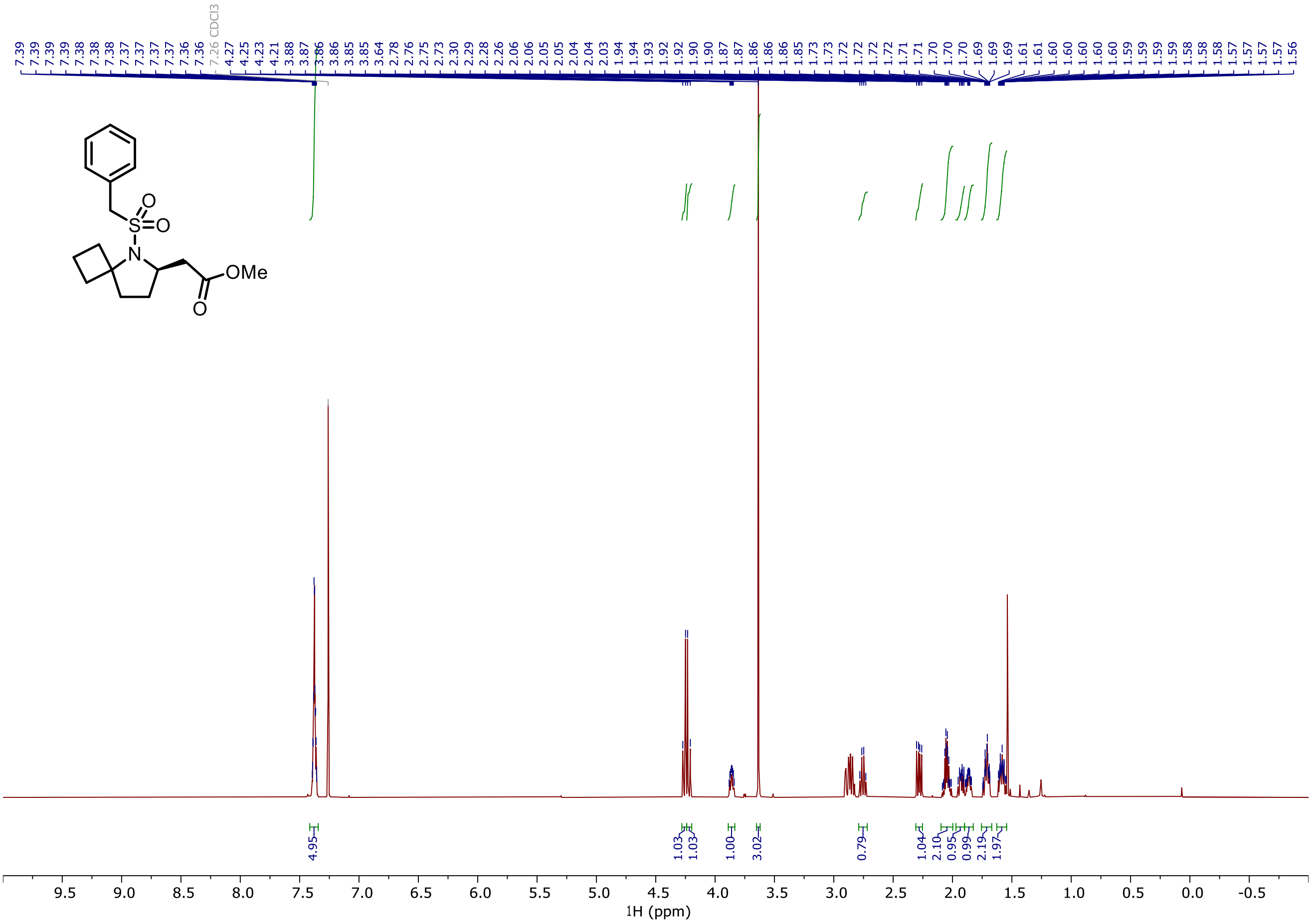

^{13}C NMR (151 MHz, $CDCl_3$) of compound **8v**

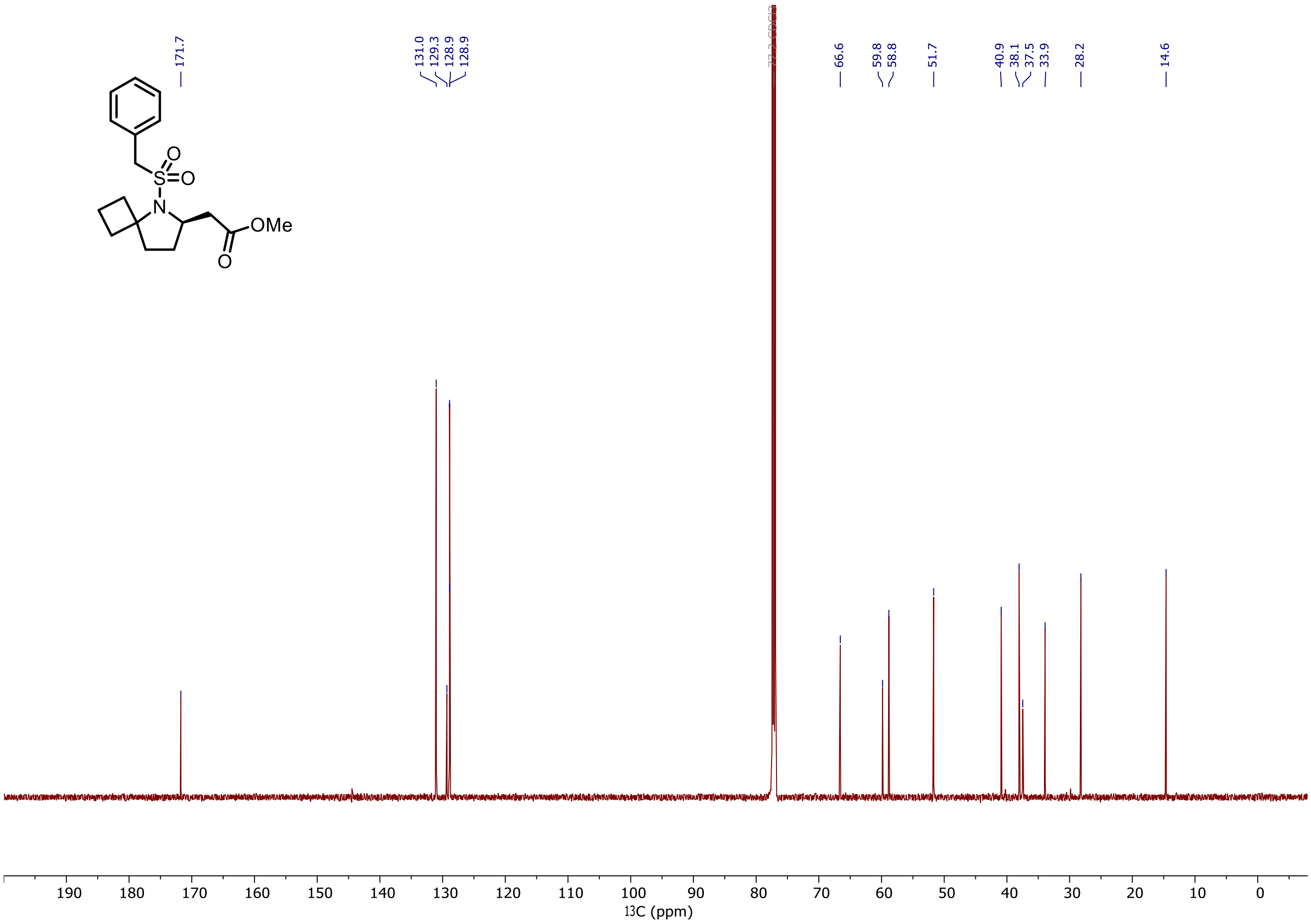

[back to table of contents]

^{1}H NMR (600 MHz, $CDCl_3$) of compound **8w**

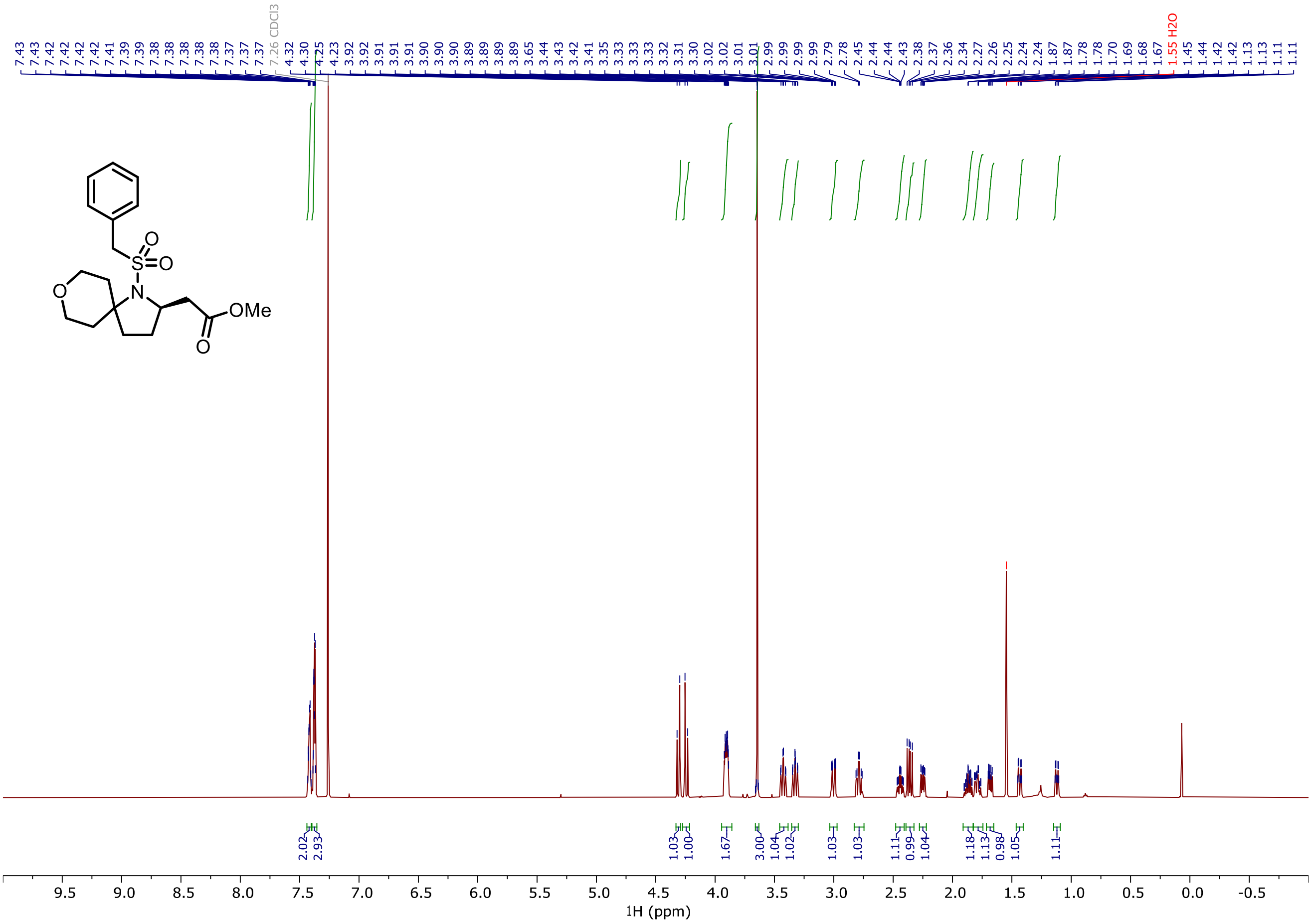

^{13}C NMR (151 MHz, $CDCl_3$) of compound **8w**

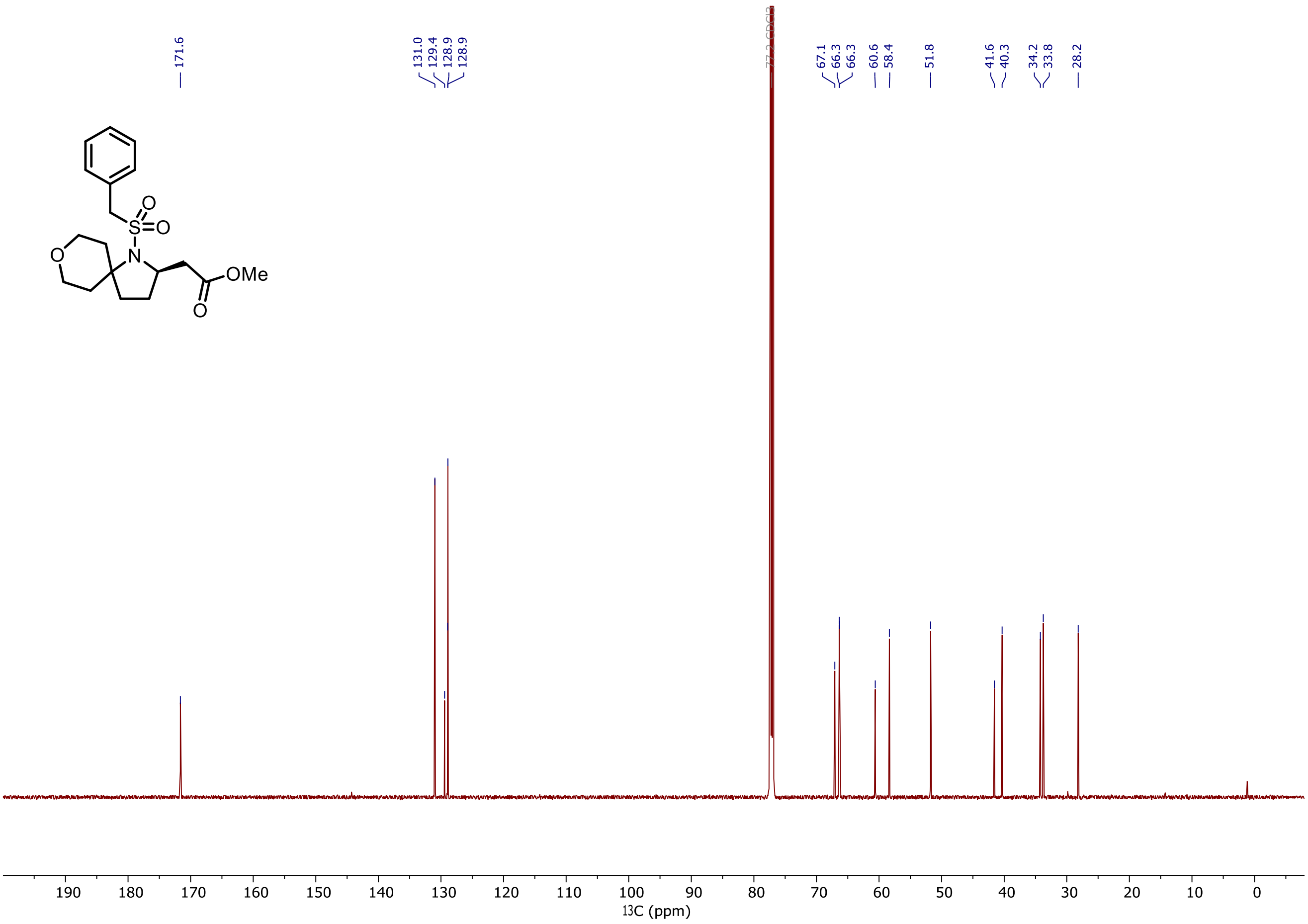

[back to table of contents]

^{1}H NMR (600 MHz, $CDCl_3$) of compound **8x**

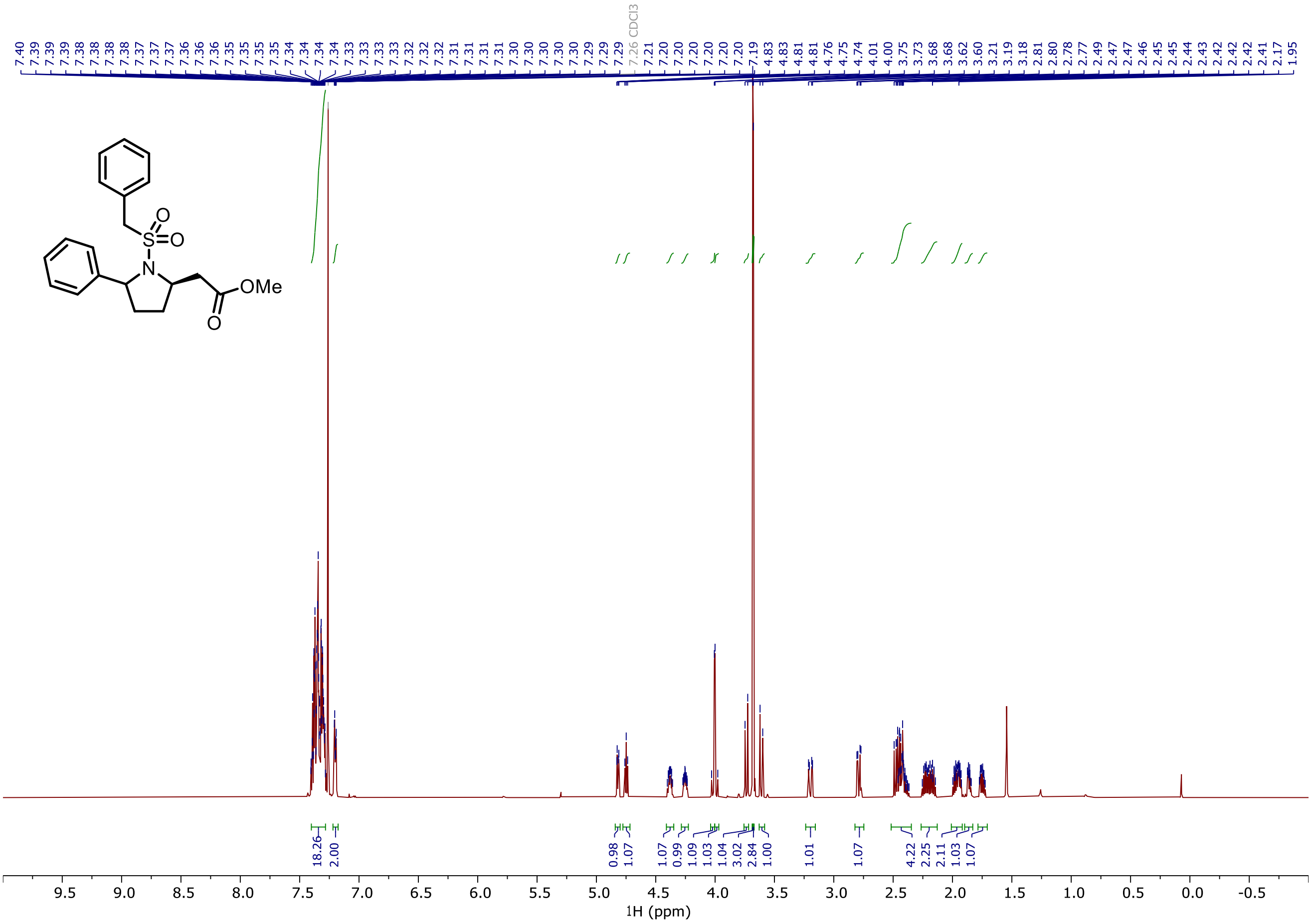

^{13}C NMR (151 MHz, $CDCl_3$) of compound **8x**

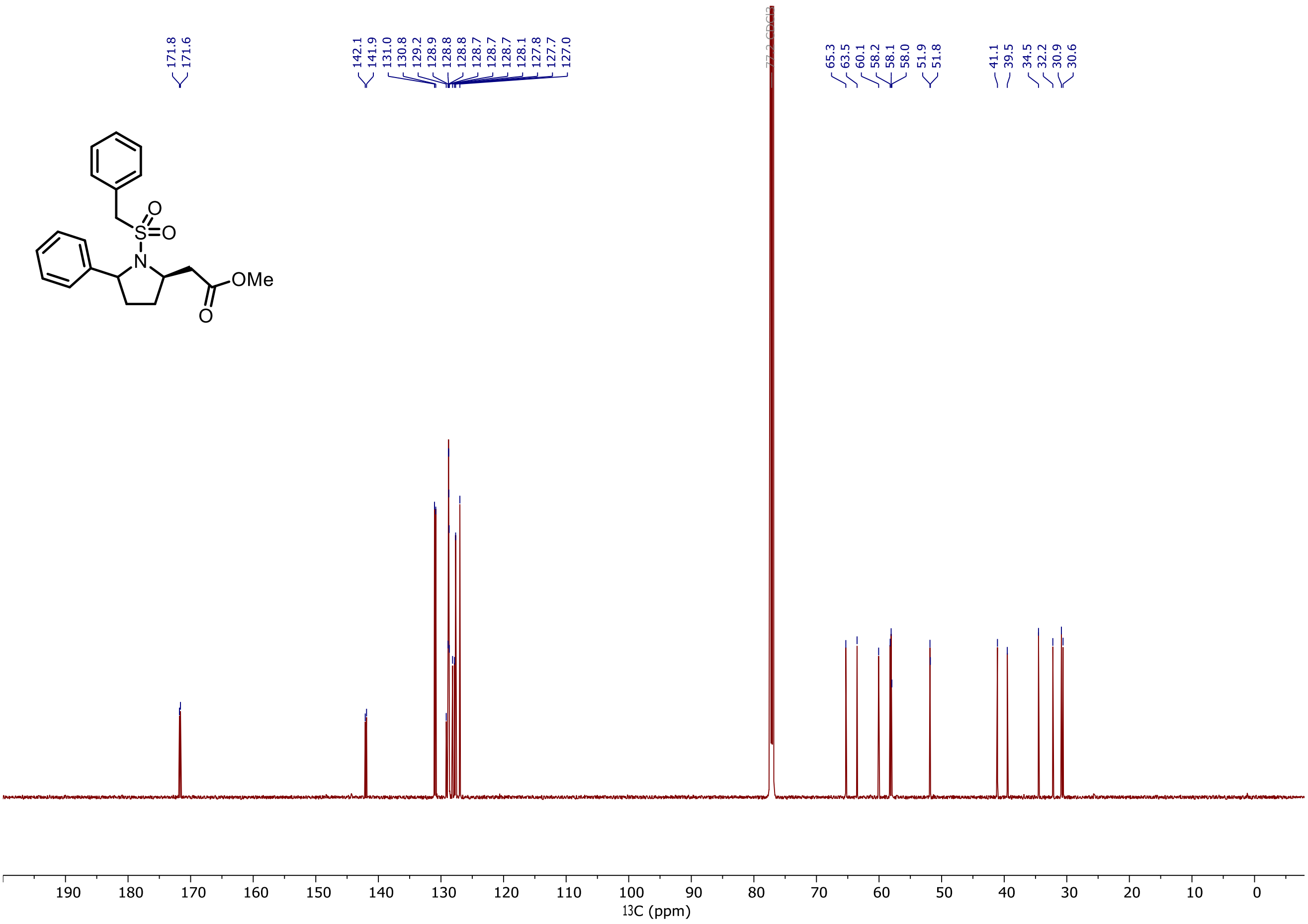

[back to table of contents]

^{1}H NMR (600 MHz, $CDCl_3$) of compound **8y**

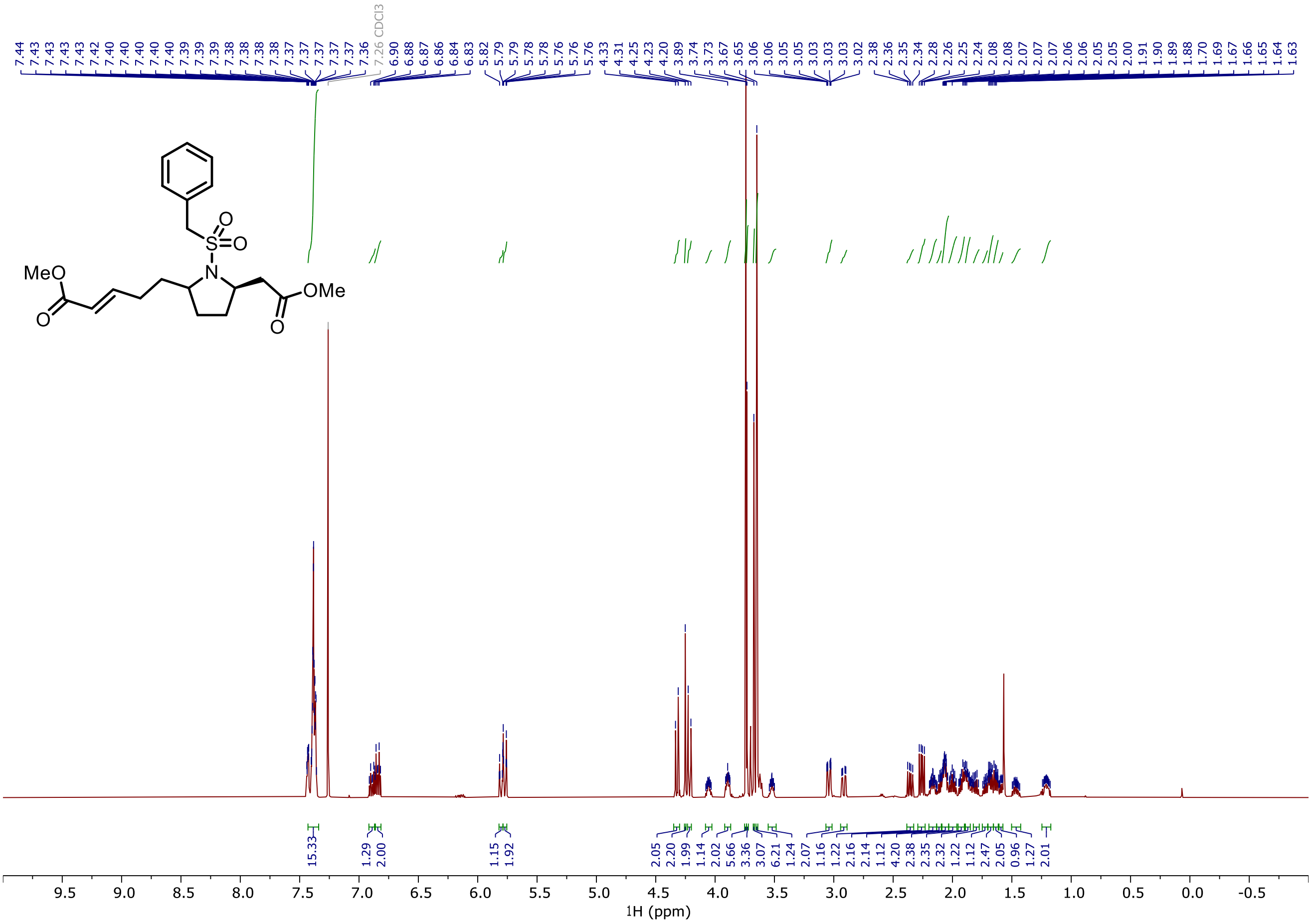

^{13}C NMR (151 MHz, $CDCl_3$) of compound **8y**

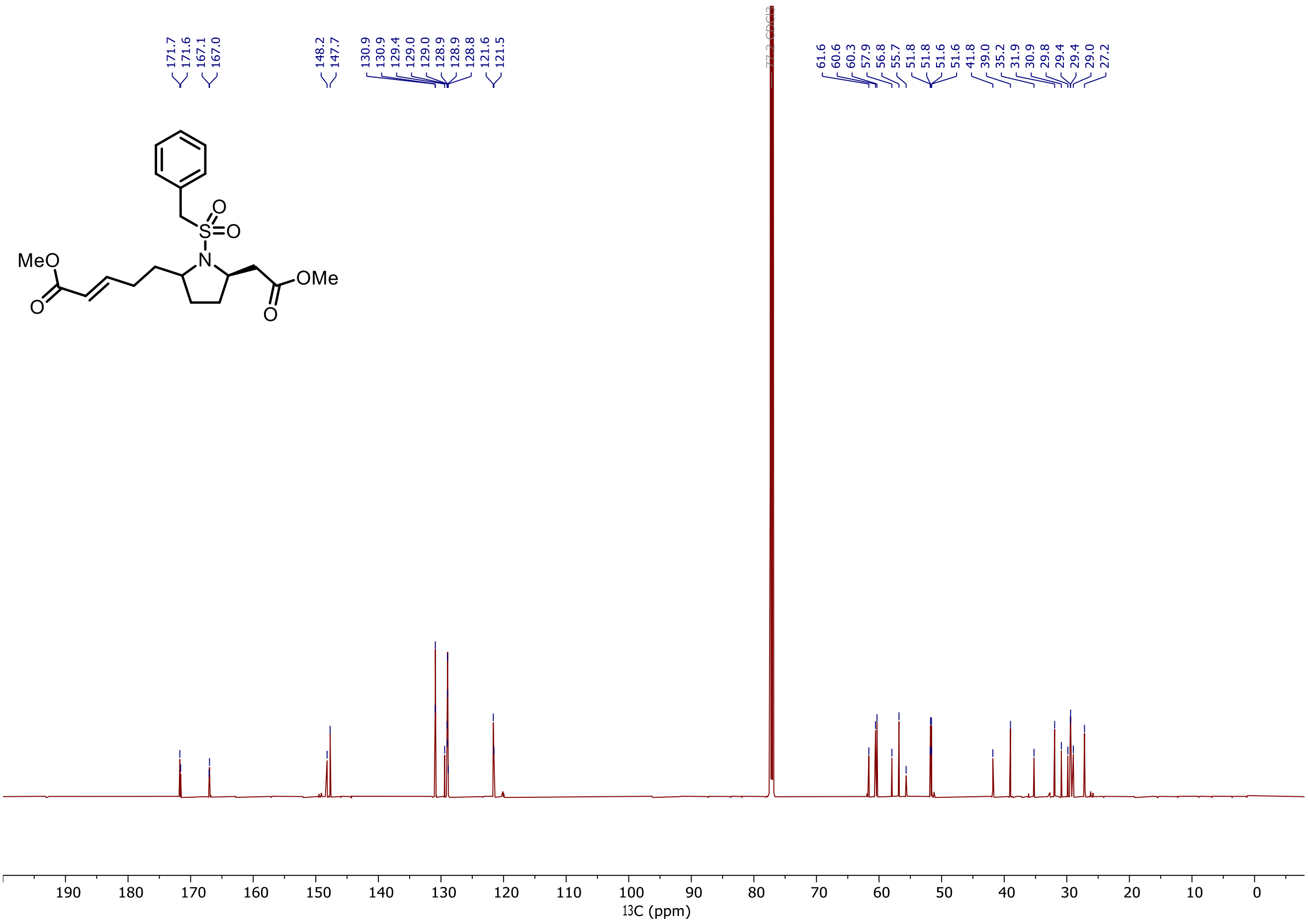

[back to table of contents]

^{1}H NMR (600 MHz, $CDCl_3$) of compound **8z**

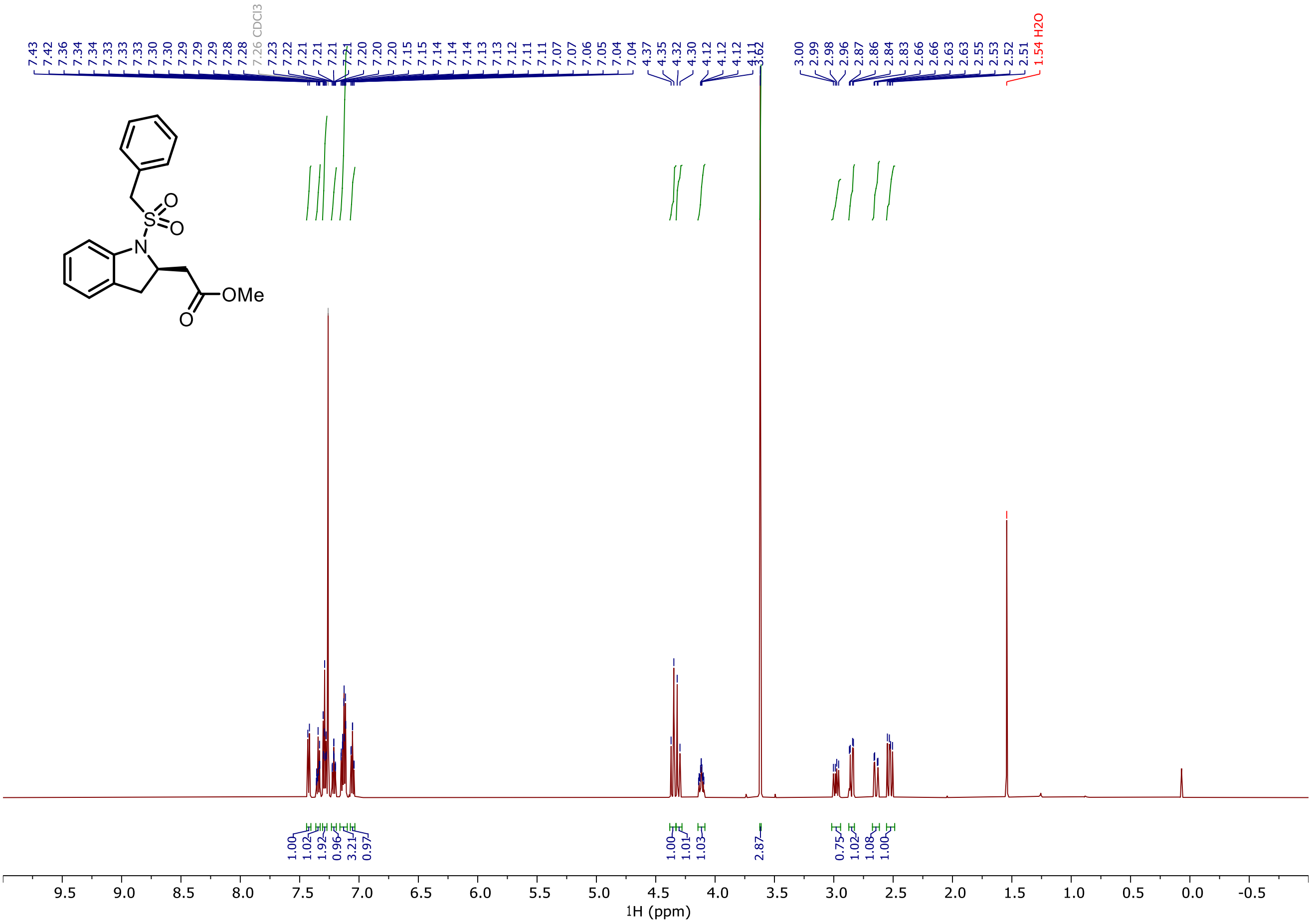

^{13}C NMR (151 MHz, $CDCl_3$) of compound **8z**

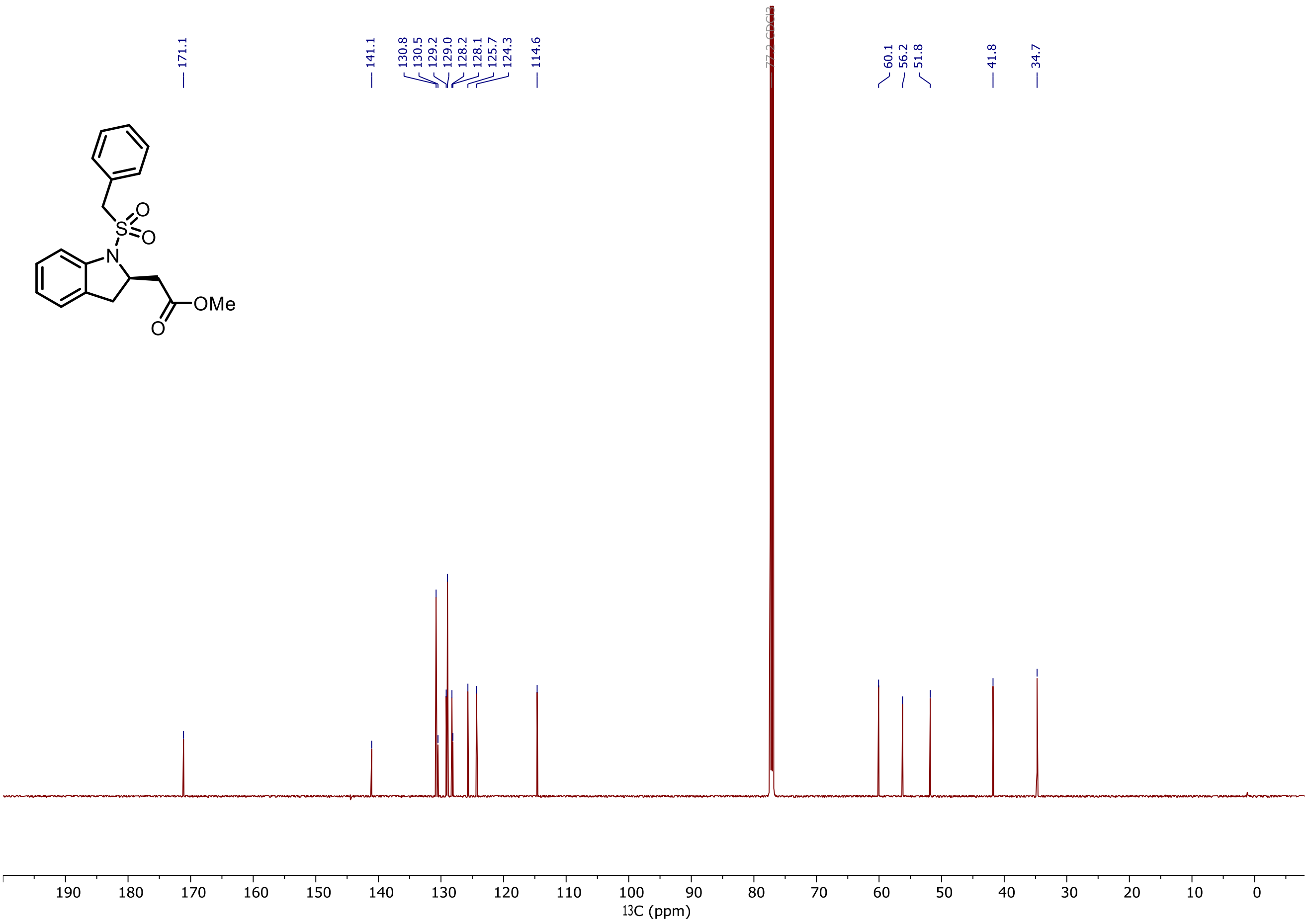

[back to table of contents]

^{1}H NMR (400 MHz, $CDCl_3$) of compound **8aa**

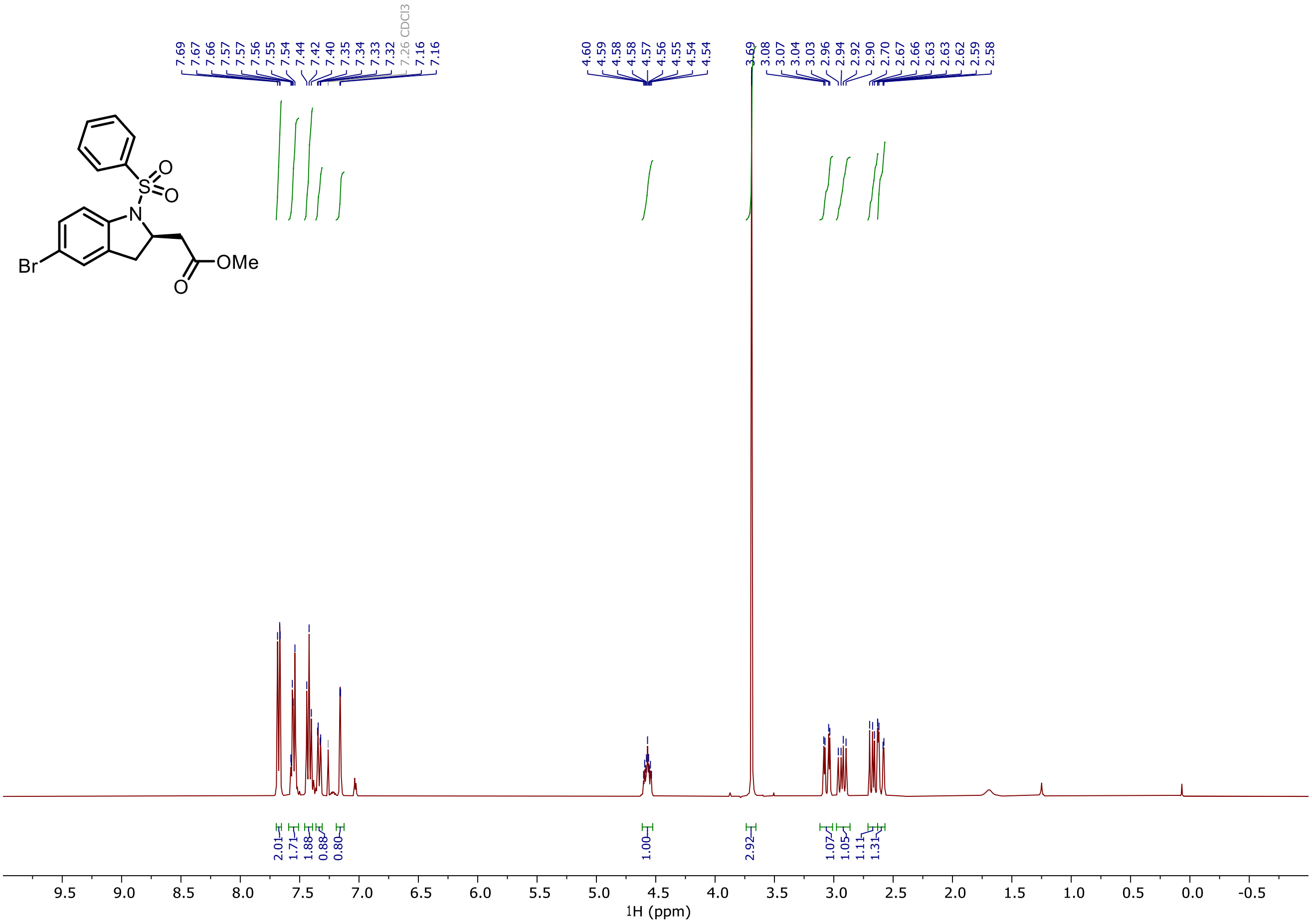

^{13}C NMR (101 MHz, $CDCl_3$) of compound **8aa**

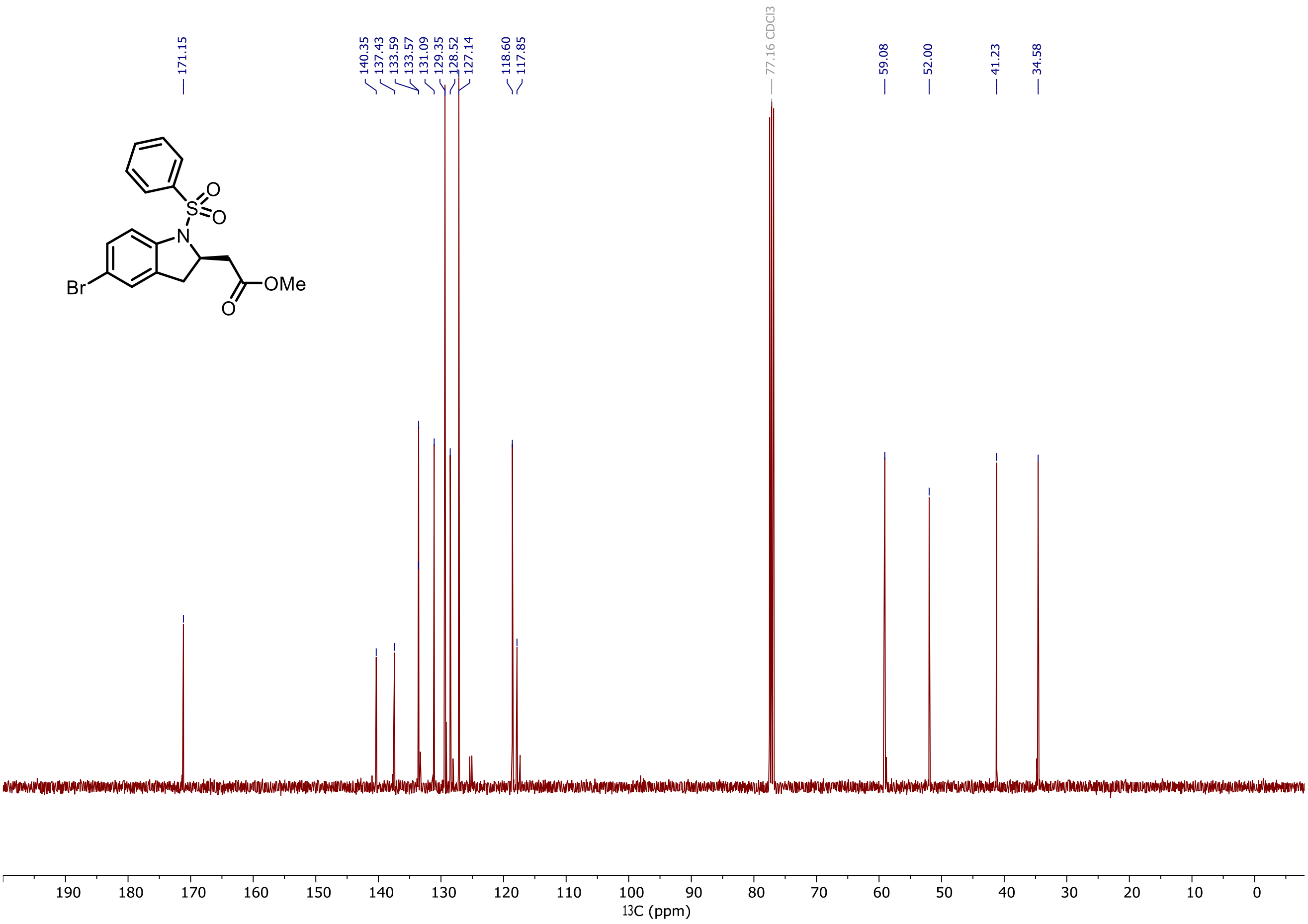

[back to table of contents]

^{1}H NMR (400 MHz, $CDCl_3$) of compound **8ab**

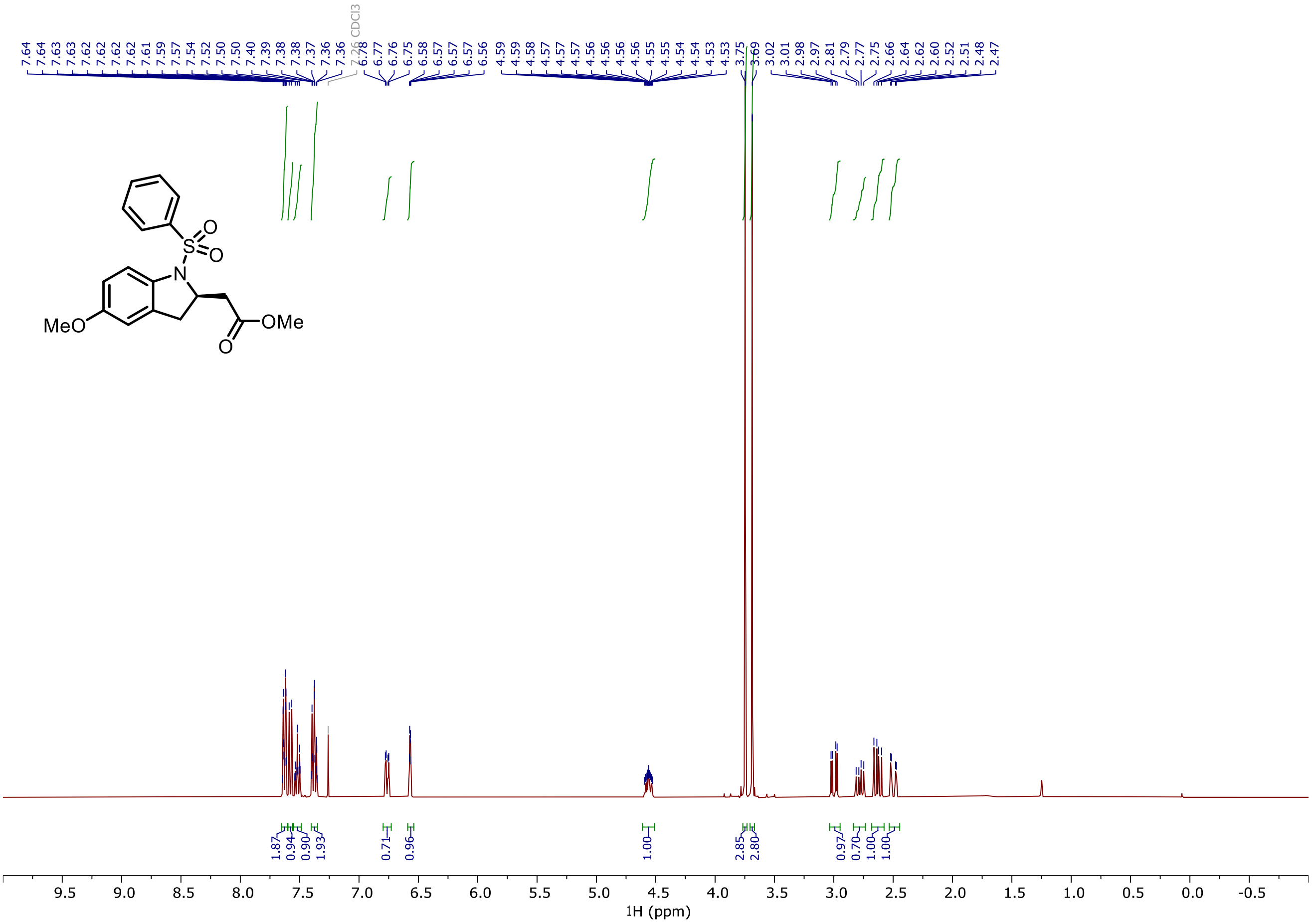

^{13}C NMR (101 MHz, $CDCl_3$) of compound **8ab**

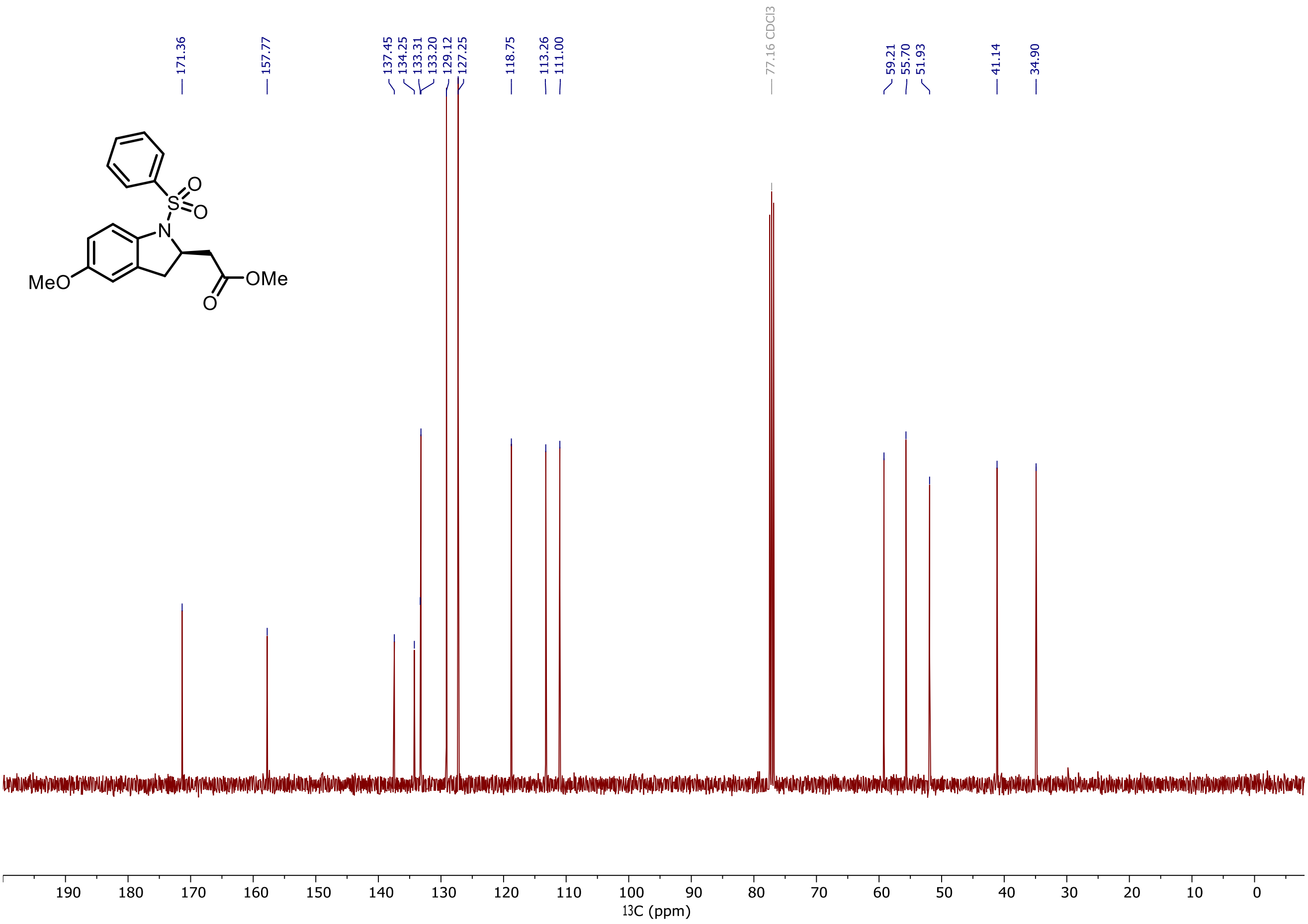

[back to table of contents]

^{1}H NMR (400 MHz, $CDCl_3$) of compound **8ac**

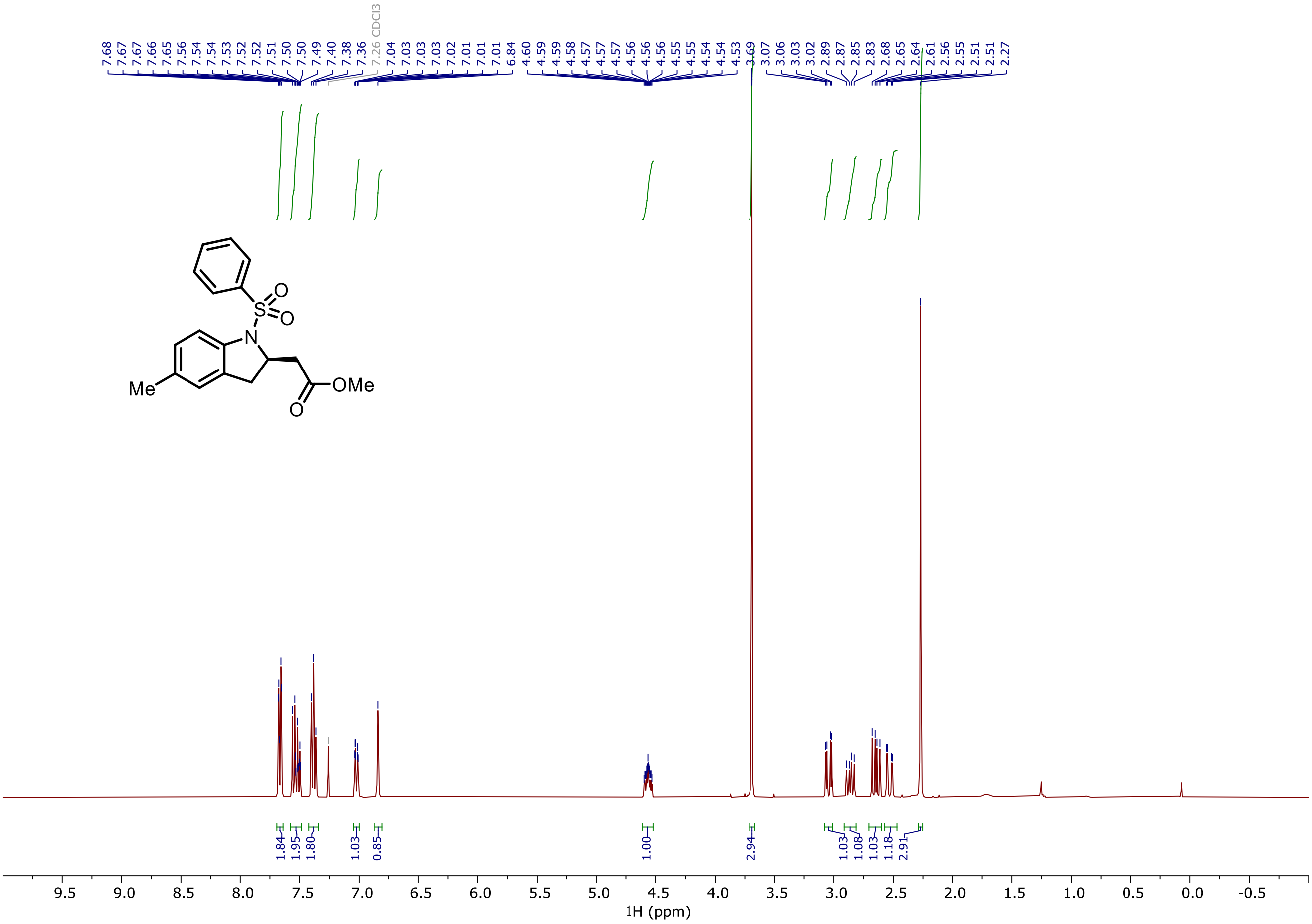

^{13}C NMR (101 MHz, $CDCl_3$) of compound **8ac**

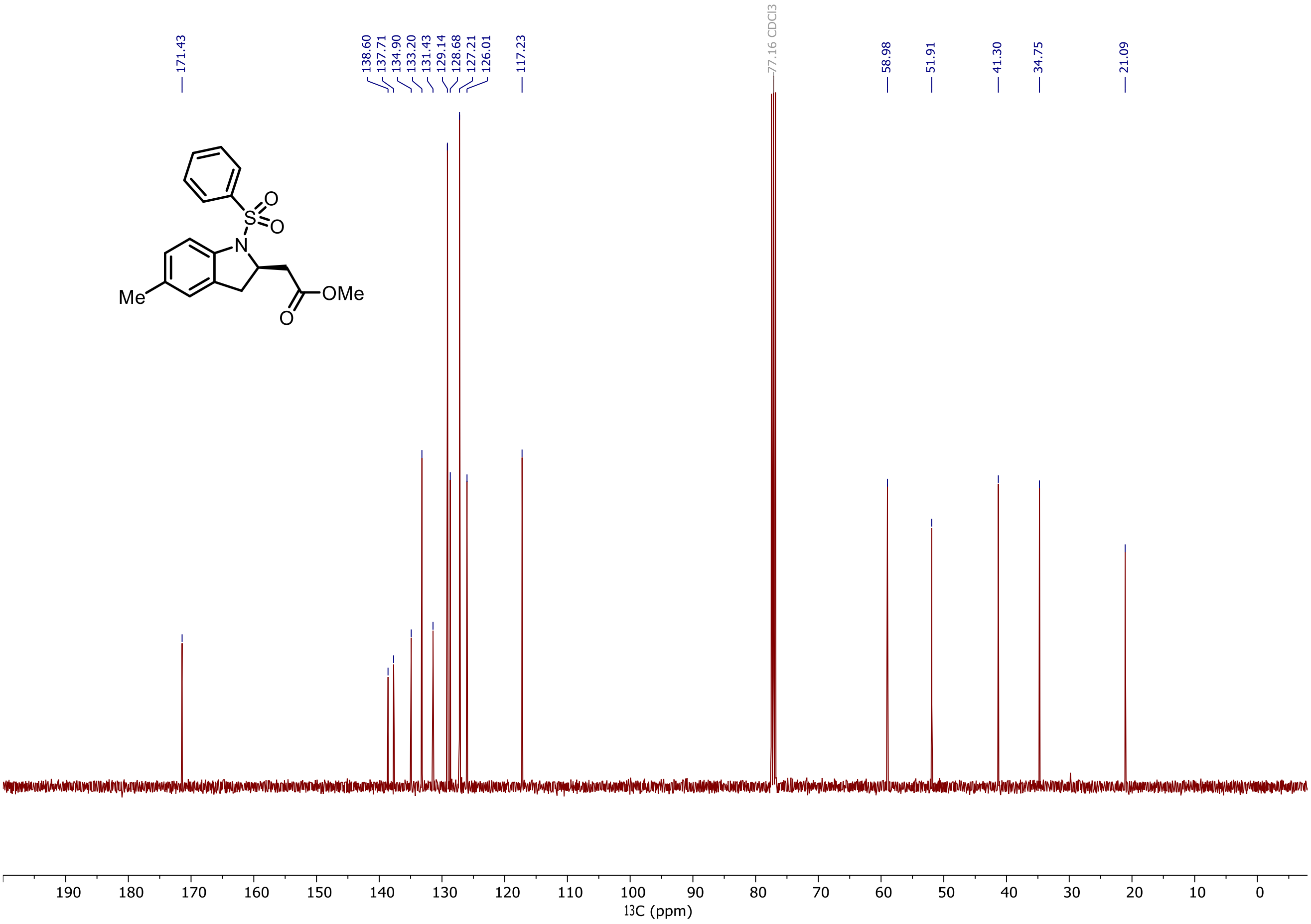

[back to table of contents]

^{1}H NMR (600 MHz, $CDCl_3$) of compound **8ad**

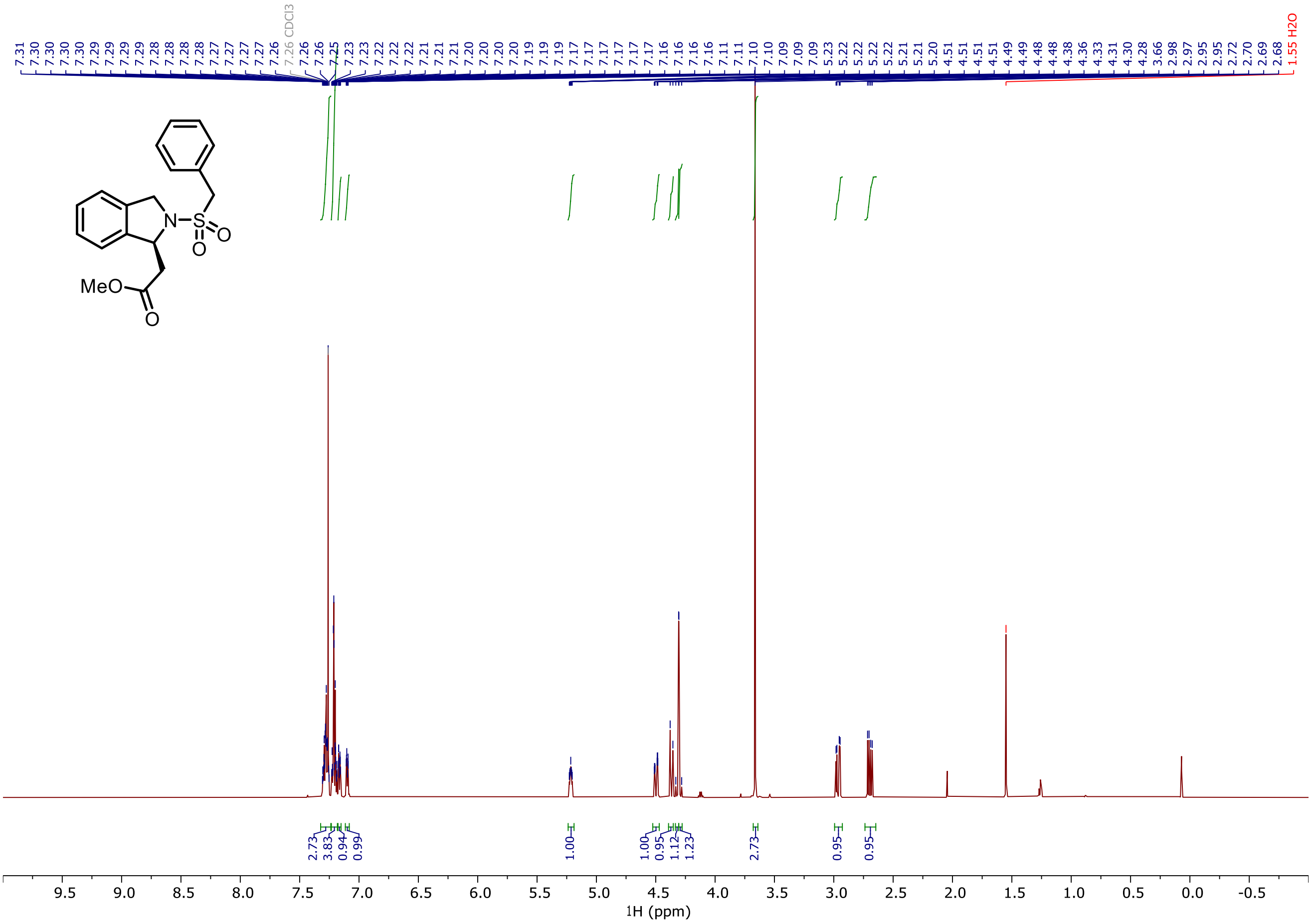

^{13}C NMR (151 MHz, $CDCl_3$) of compound **8ad**

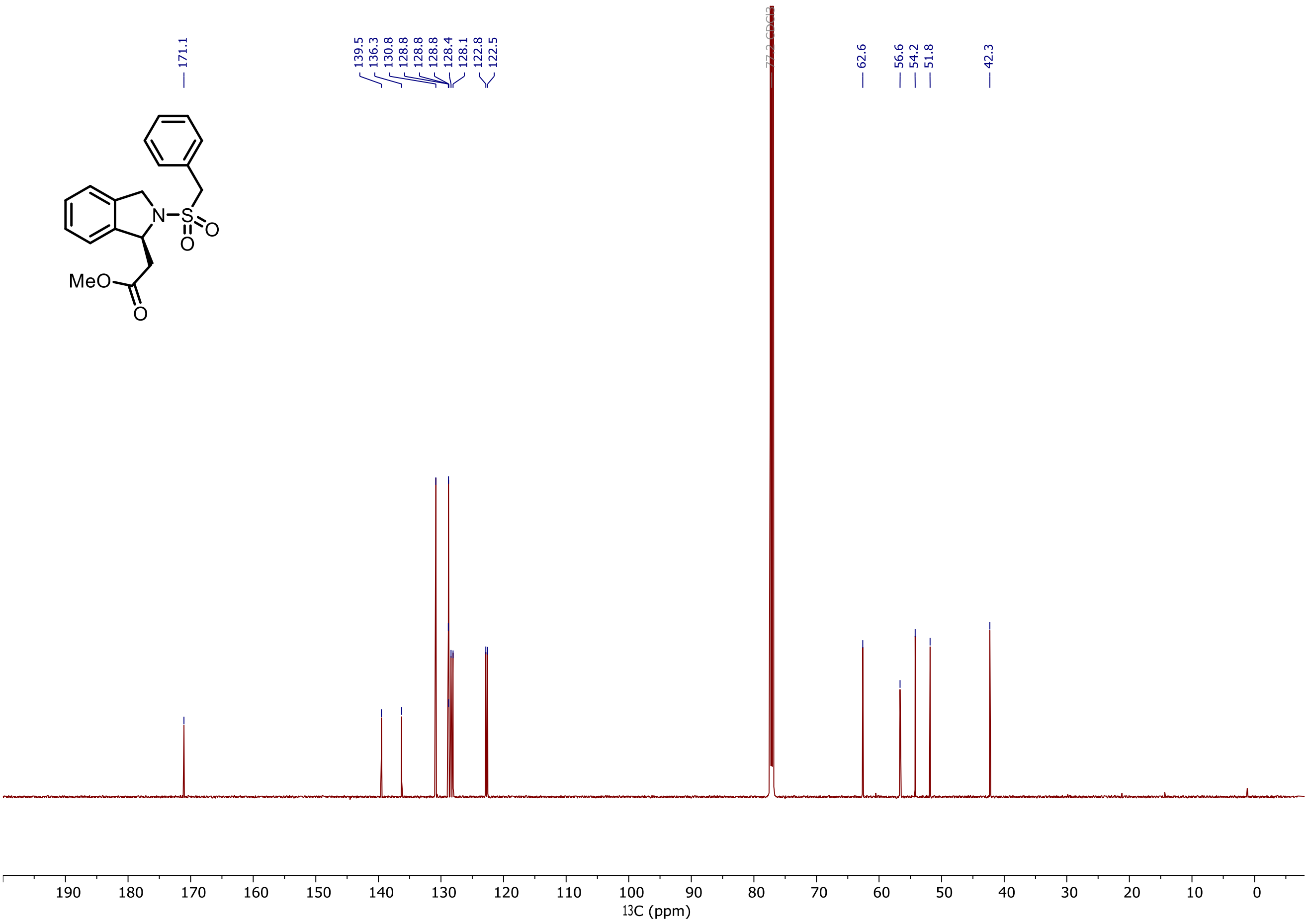

[back to table of contents]

^{1}H NMR (400 MHz, $CDCl_3$) of compound **8ae**

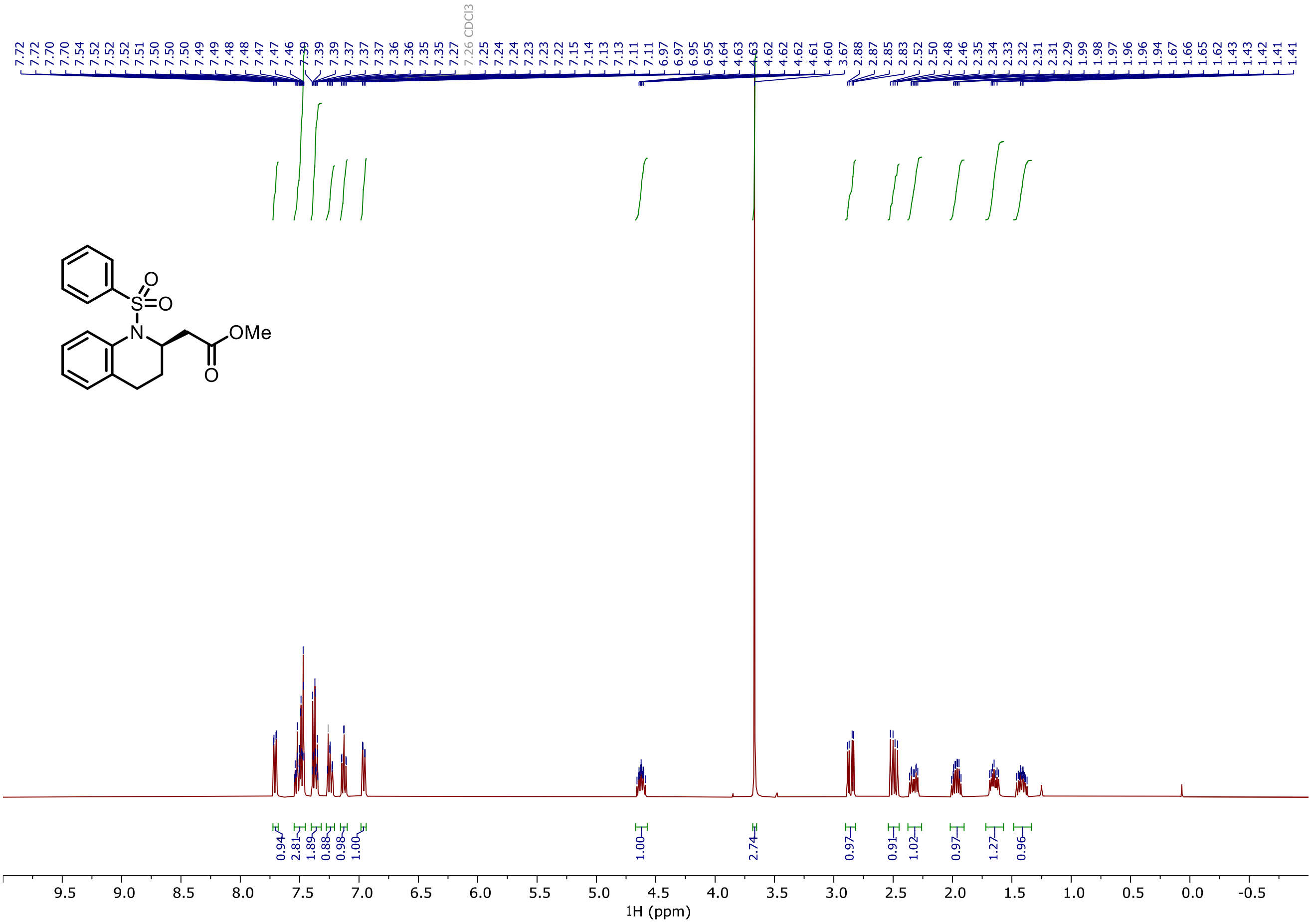

^{13}C NMR (101 MHz, $CDCl_3$) of compound **8ae**

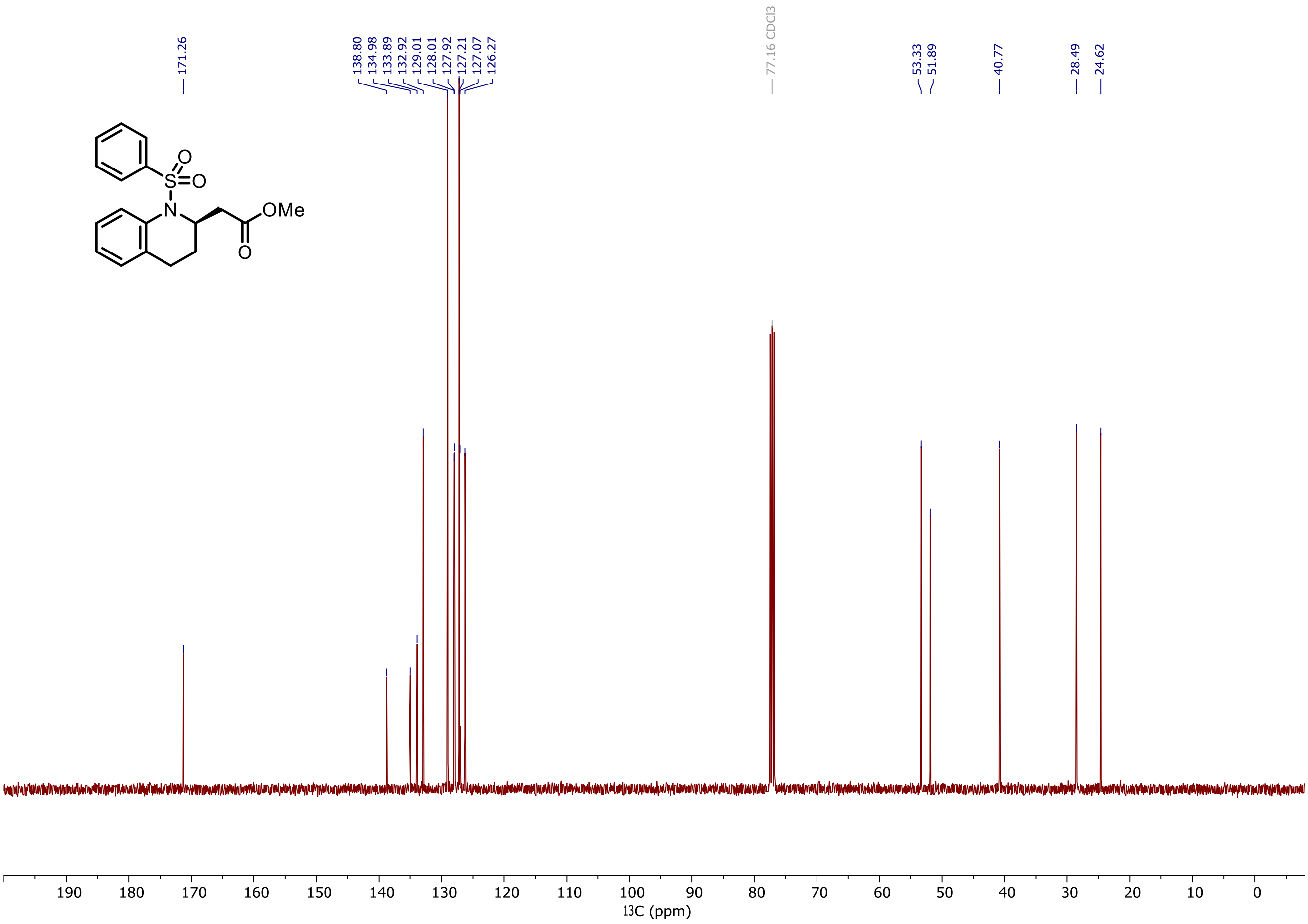

[back to table of contents]

^{1}H NMR (600 MHz, $CDCl_3$) of compound **10**

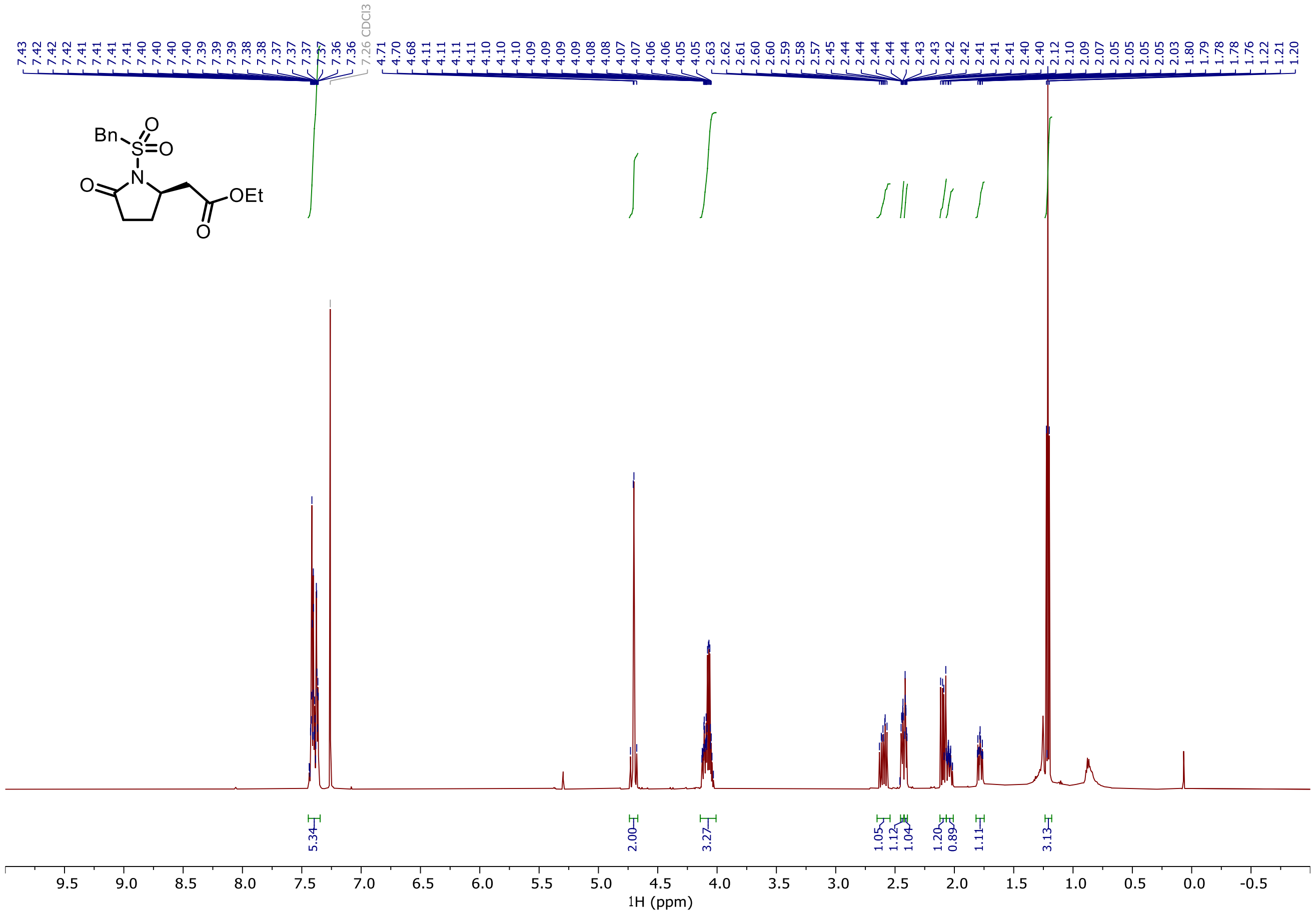

^{13}C NMR (151 MHz, $CDCl_3$) of compound **10**

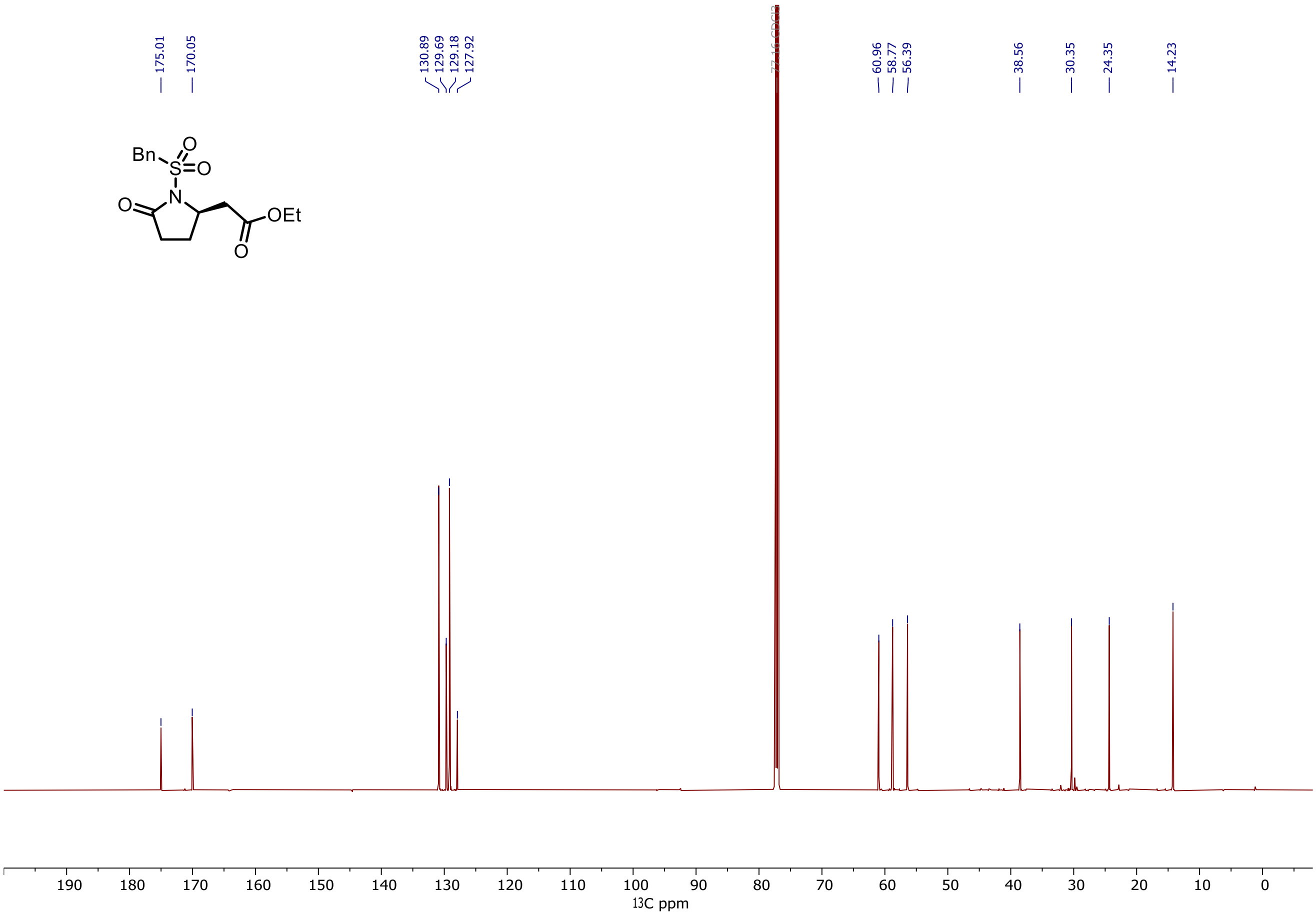

[back to table of contents]

^{1}H NMR (600 MHz, $CDCl_3$) of compound **11**

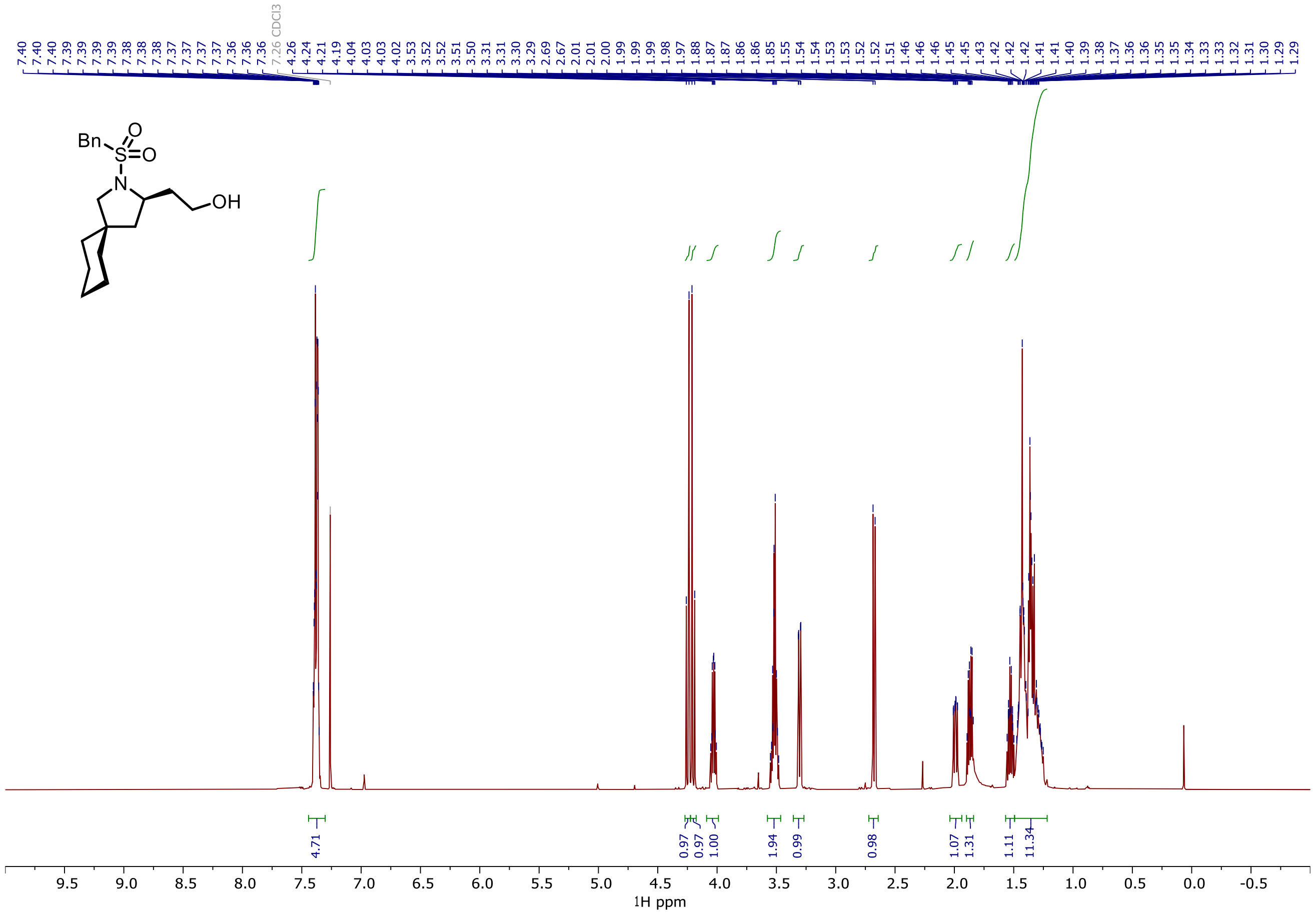

^{13}C NMR (151 MHz, $CDCl_3$) of compound **11**

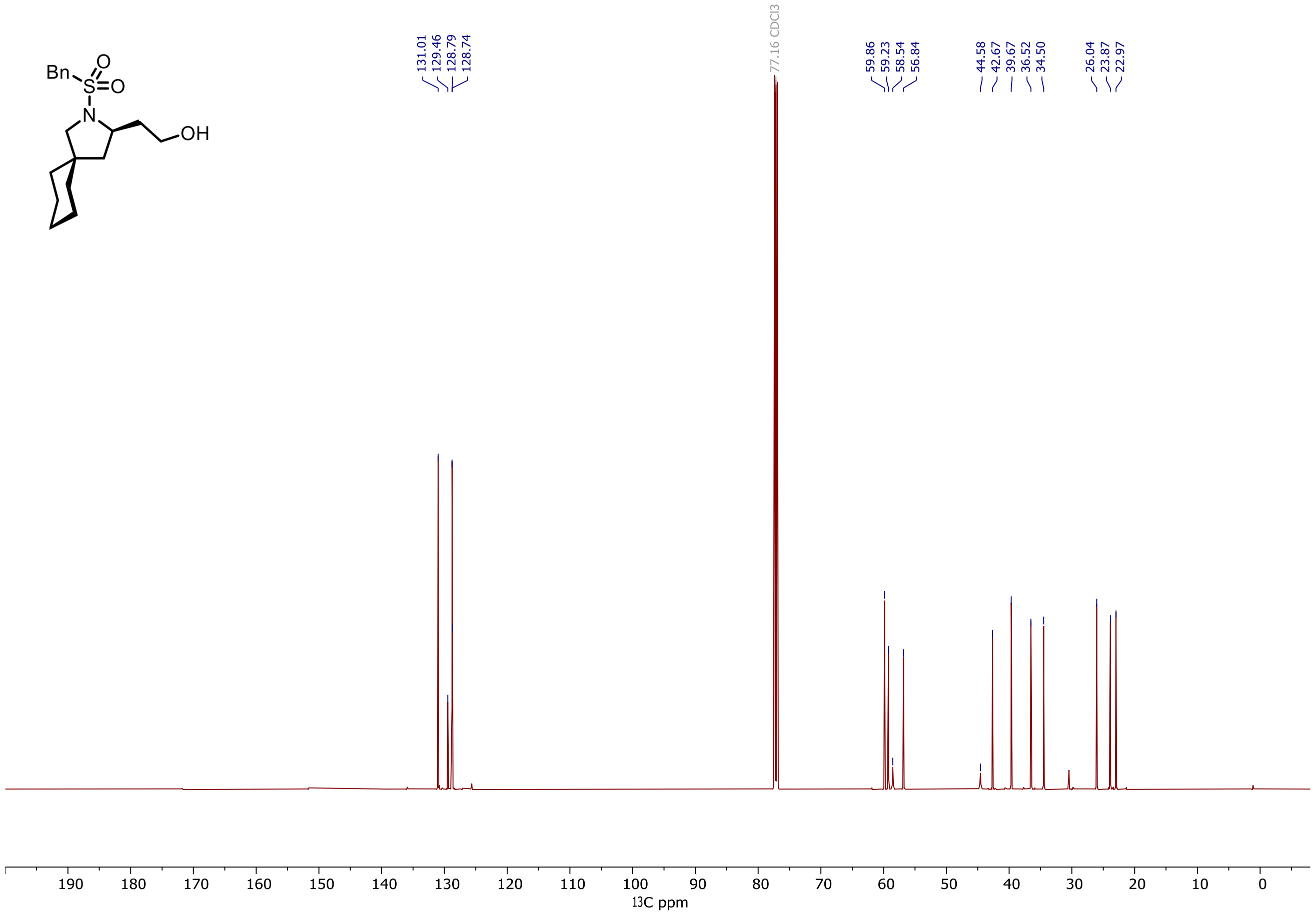

[back to table of contents]

^{1}H NMR (600 MHz, $CDCl_3$) of compound **12**

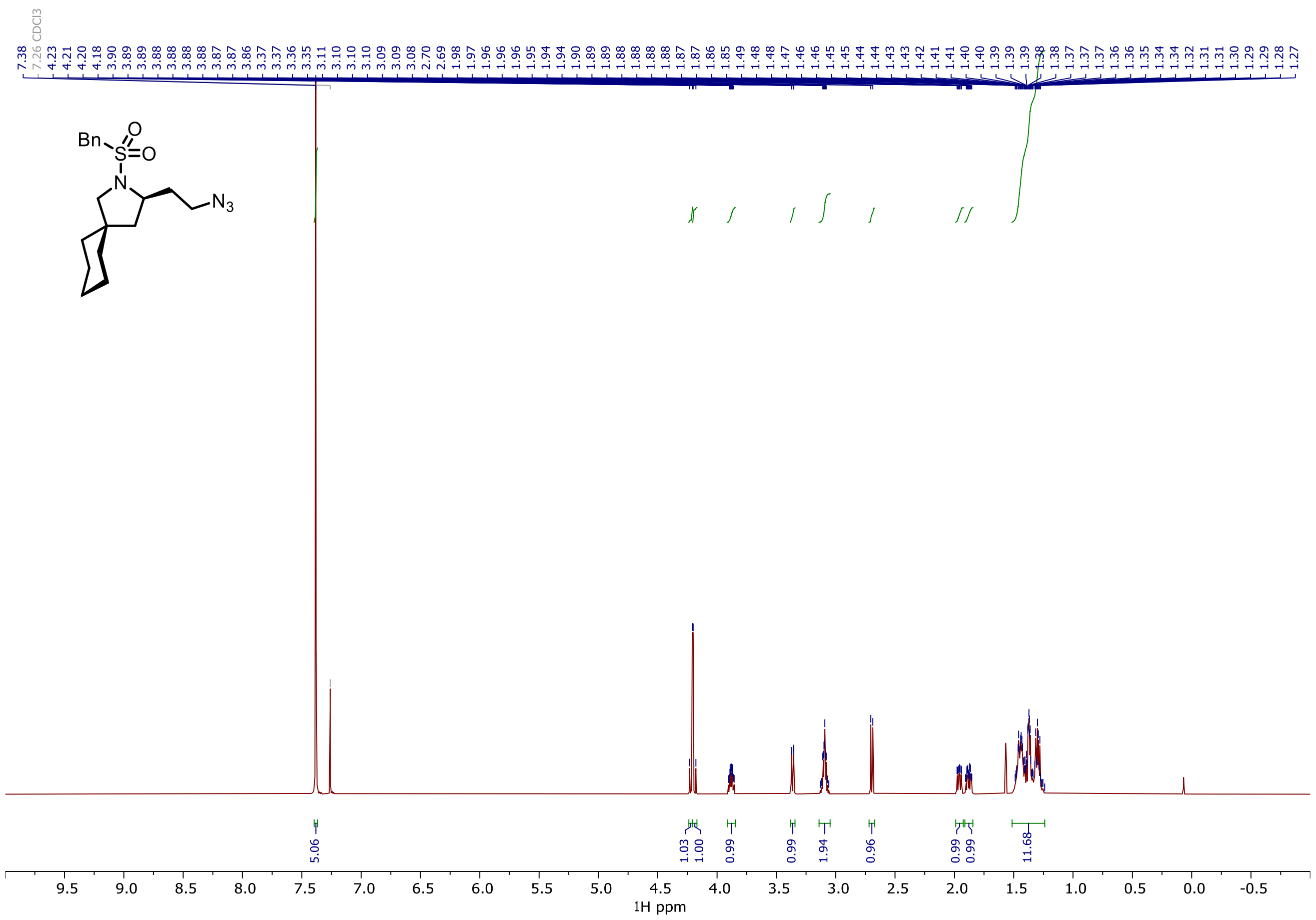

^{13}C NMR (151 MHz, $CDCl_3$) of compound **12**

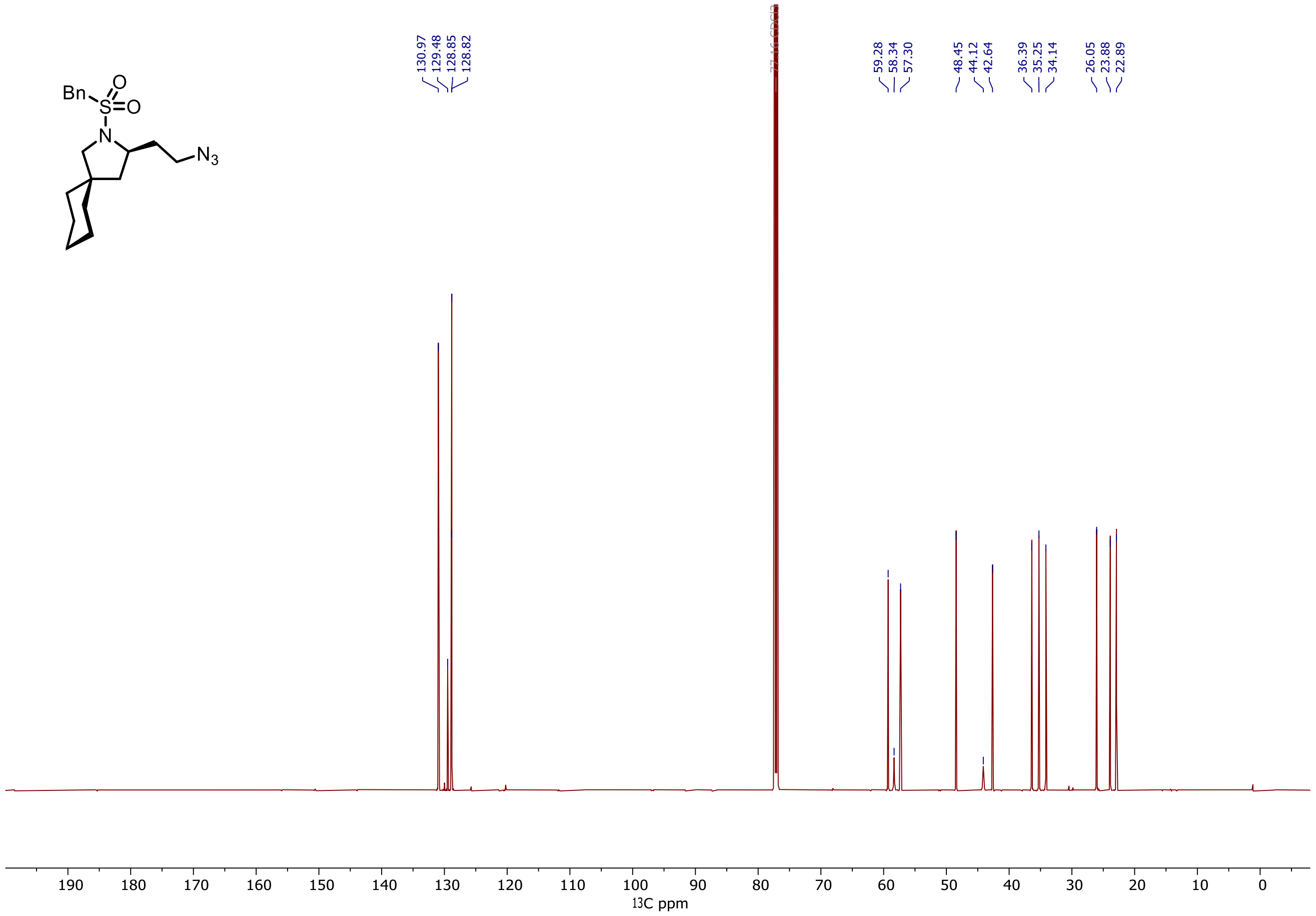

[back to table of contents]

^{1}H NMR (600 MHz, $CDCl_3$) of compound **13**

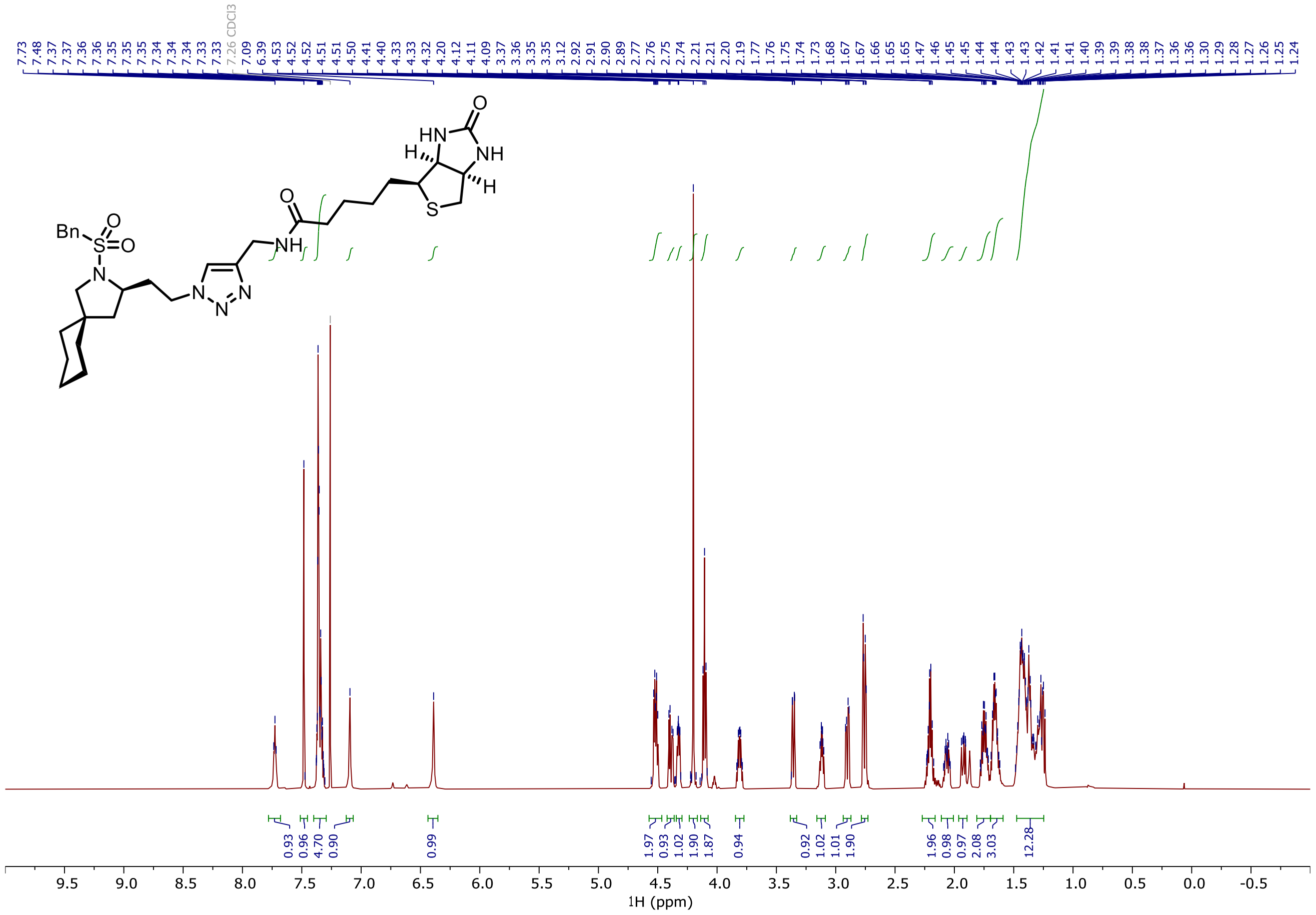

^{13}C NMR (151 MHz, $CDCl_3$) of compound **13**

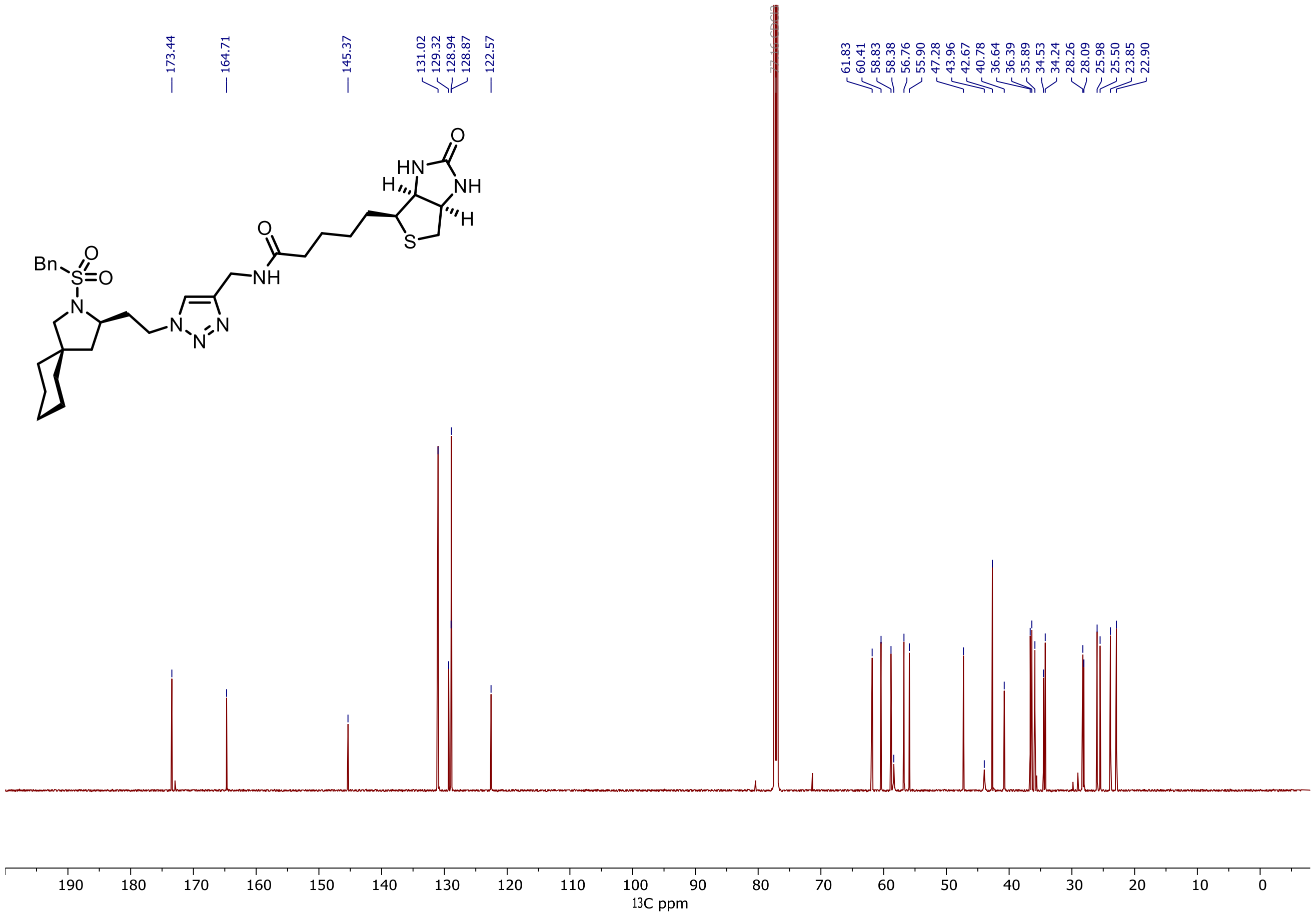

[back to table of contents]

HPLC and SFC Traces

methyl (*R*)-2-(1-(phenylsulfonyl)pyrrolidin-2-yl)acetate (8a)

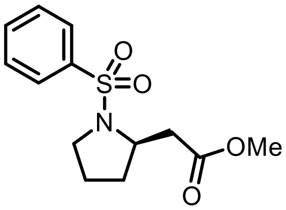

(CHIRALPAK® OD, Hexane/IPA 95/5, 1 mL/min)

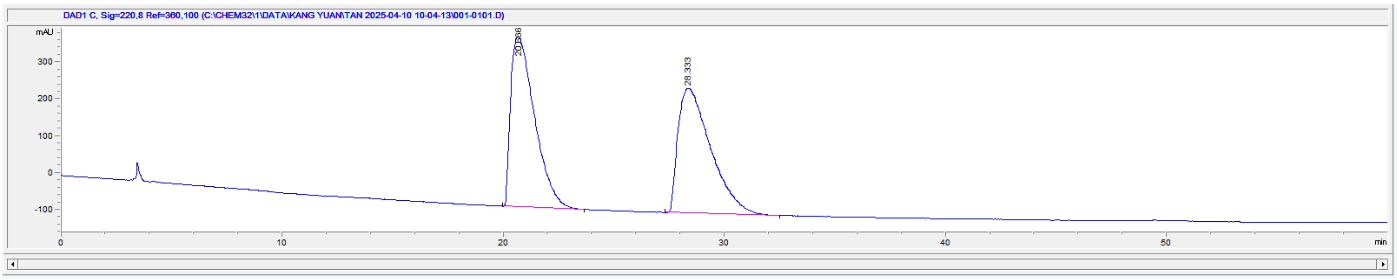

#	Time	Area	Height	Width	Area%	Symmetry
1	20.666	34773.1	461.6	1.0228	49.910	0.428
2	28.333	34898.6	337.8	1.2392	50.090	0.407

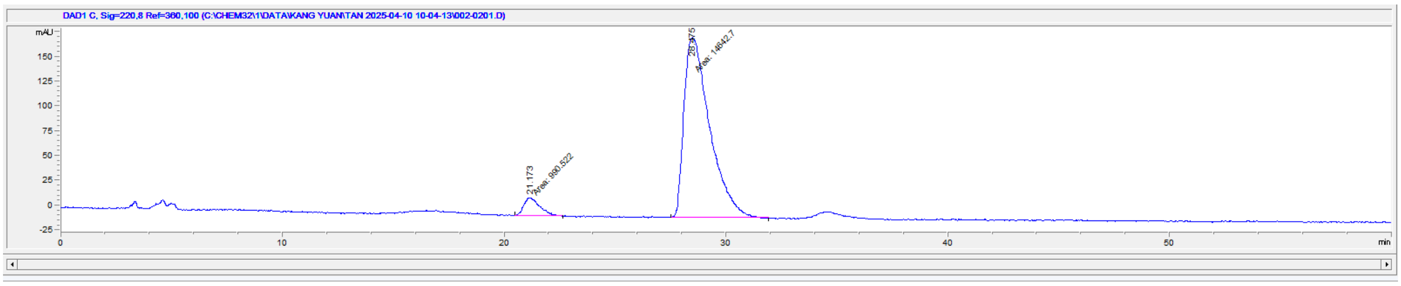

#	Time	Area	Height	Width	Area%	Symmetry
1	21.173	990.5	18.9	0.8752	6.336	0.698
2	28.475	14642.7	182.4	1.3377	93.664	0.483

HPLC Data From 150 mg Scale Reaction

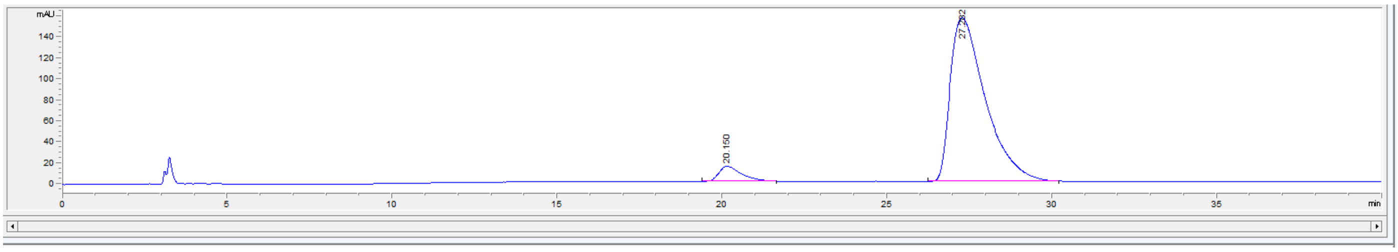

#	Time	Area	Height	Width	Area%	Symmetry
1	20.15	696.2	14.4	0.7051	5.687	0.568
2	27.282	11545.2	156	1.1294	94.313	0.48

[back to table of contents]

ethyl (*R*)-2-(1-(benzylsulfonyl)pyrrolidin-2-yl)acetate (8b)

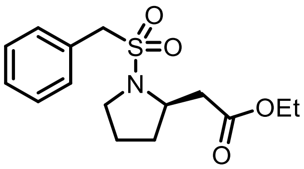

(CHIRALPAK® IA, Hexane/IPA 90/10, 1 mL/min)

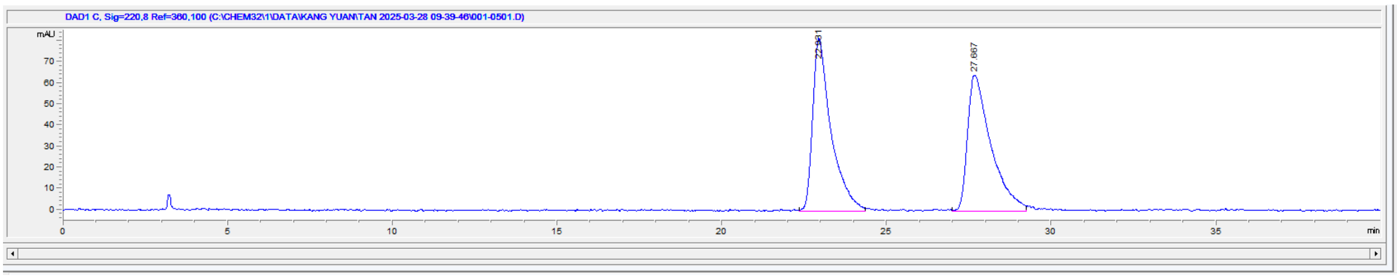

#	Time	Area	Height	Width	Area%	Symmetry
1	22.931	3266.3	82.3	0.5533	50.250	0.472
2	27.667	3233.8	64.6	0.696	49.750	0.433

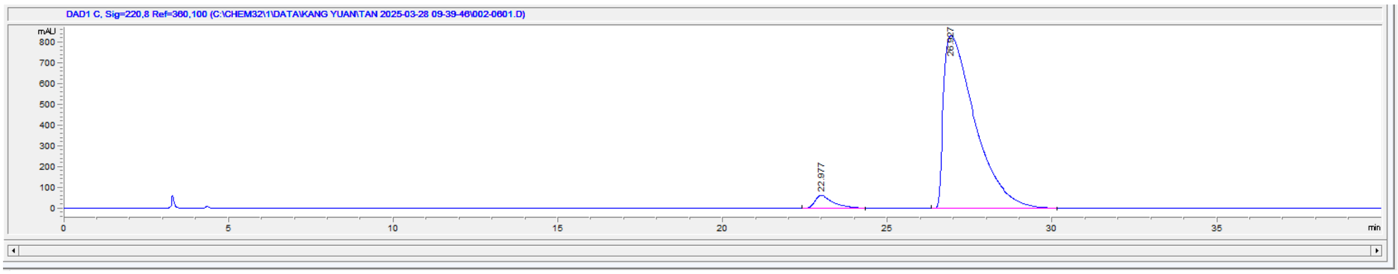

#	Time	Area	Height	Width	Area%	Symmetry
1	22.977	2508.9	64.4	0.5405	4.334	0.491
2	26.927	55380.7	835.6	0.8952	95.666	0.294

[back to table of contents]

tert-butyl (*R*)-2-(1-(phenylsulfonyl)pyrrolidin-2-yl)acetate (8c)

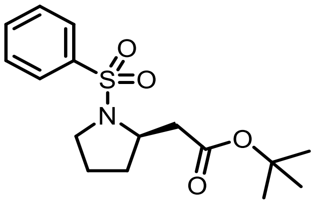

(CHIRALPAK® OD, Hexane/IPA 95/5, 1 mL/min)

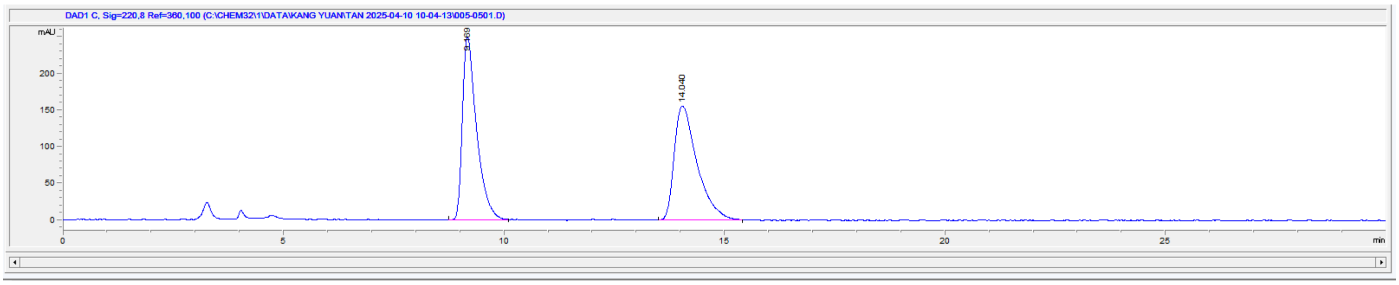

#	Time	Area	Height	Width	Area%	Symmetry
1	9.169	5665.8	250.8	0.3335	49.827	0.529
2	14.04	5705.2	156.1	0.5303	50.173	0.52

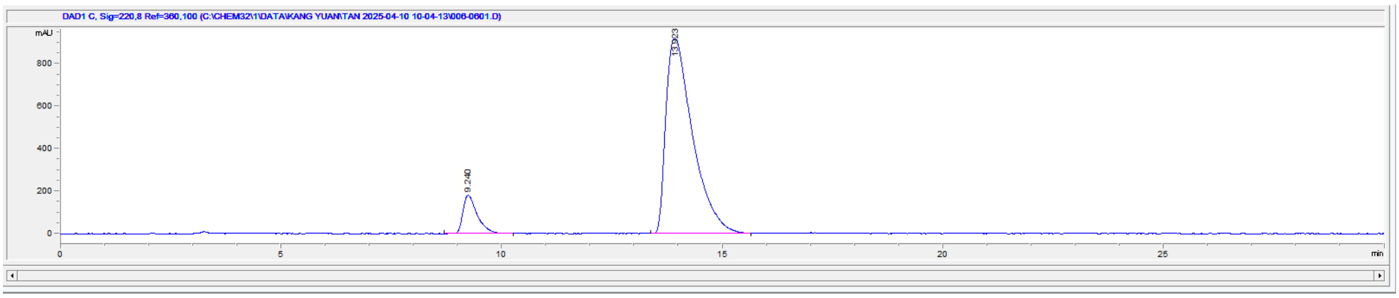

#	Time	Area	Height	Width	Area%	Symmetry
1	9.24	4133.2	181.5	0.3377	9.582	0.529
2	13.923	39003.2	922.5	0.6053	90.418	0.465

[back to table of contents]

(*R*)-*N*,*N*-dimethyl-2-(1-(phenylsulfonyl)pyrrolidin-2-yl)acetamide (8d)

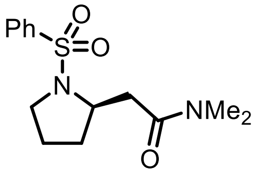

(CHIRALPAK® AS-H, Hexane/IPA 80/20, 1 mL/min)

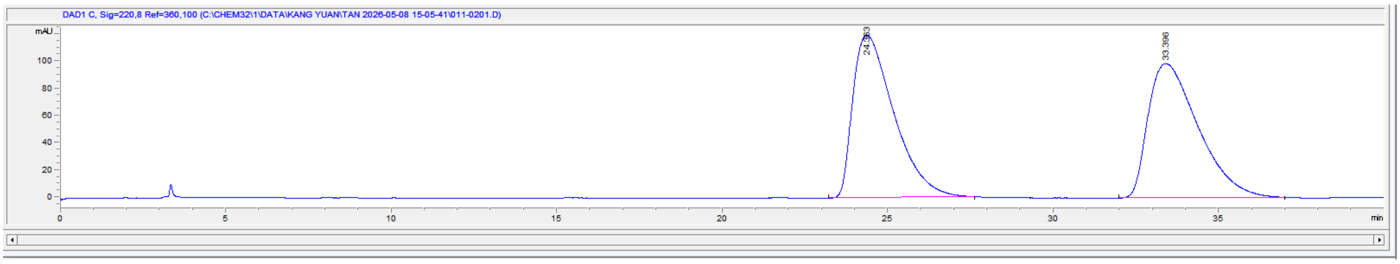

#	Time	Area	Height	Width	Area%	Symmetry
1	24.363	10334.5	119.7	1.3009	49.826	0.479
2	33.396	10406.7	98.8	1.5866	50.174	0.511

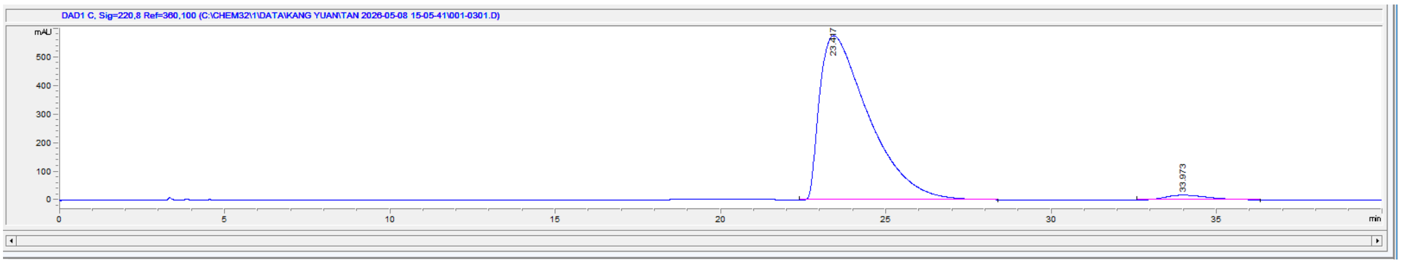

#	Time	Area	Height	Width	Area%	Symmetry
1	23.417	59194.8	574.7	1.5316	97.501	0.37
2	33.973	1517.4	16.7	1.206	2.499	0.632

[back to table of contents]

methyl (*R*)-2-(1-(phenylsulfonyl)piperidin-2-yl)acetate (8e)

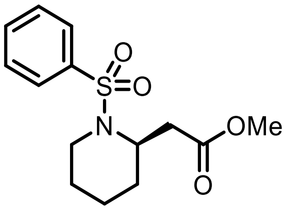

(CHIRALPAK® IA, Hexane/IPA 90/10, 1 mL/min)

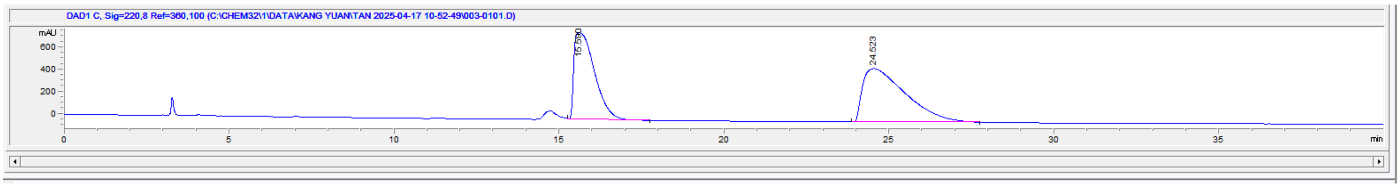

#	Time	Area	Height	Width	Area%	Symmetry
1	15.59	34201.8	786.2	0.5675	44.193	0.286
2	24.523	43190.1	482.5	1.0623	55.807	0.318

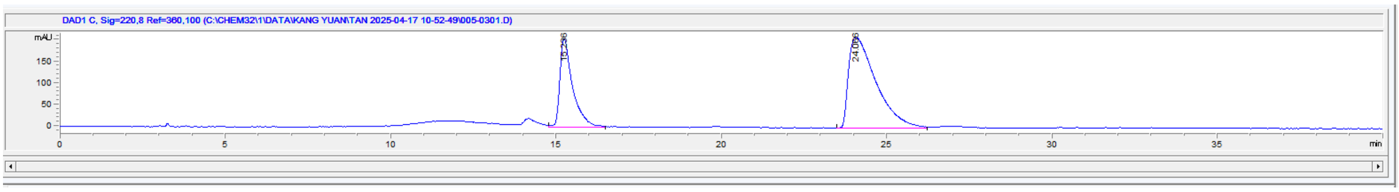

#	Time	Area	Height	Width	Area%	Symmetry
1	15.236	5621.2	206.6	0.3955	31.589	0.45
2	24.066	12173.8	210.8	0.8198	68.411	0.337

[back to table of contents]

methyl (*R*)-2-(1-(benzylsulfonyl)pyrrolidin-2-yl)acetate (8f)

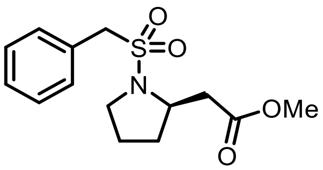

(CHIRALPAK® IA, Hexane/IPA 95/5, 1 mL/min)

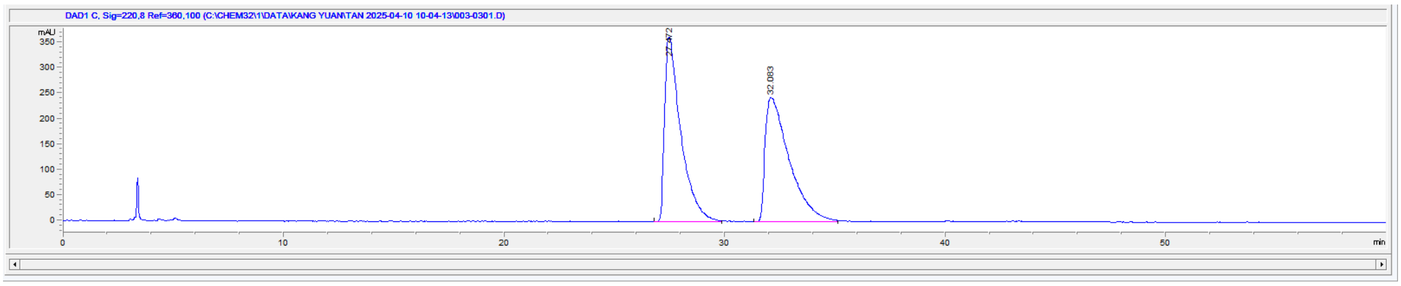

#	Time	Area	Height	Width	Area%	Symmetry
1	27.472	18652.4	364	0.7159	49.857	0.358
2	32.083	18759.7	244.6	0.9968	50.143	0.285

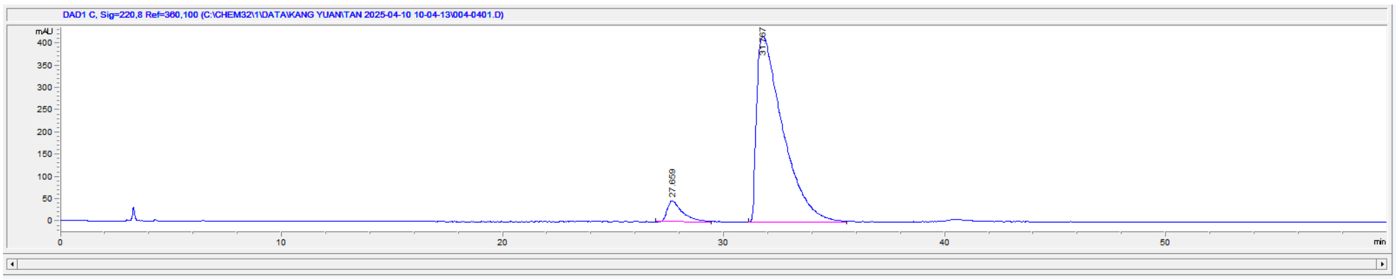

#	Time	Area	Height	Width	Area%	Symmetry
1	27.659	2346.5	47.6	0.6355	6.389	0.451
2	31.767	34378.9	418	1.1115	93.611	0.294

[back to table of contents]

methyl (*R*)-2-(1-(thiophen-2-ylsulfonyl)pyrrolidin-2-yl)acetate (8g)

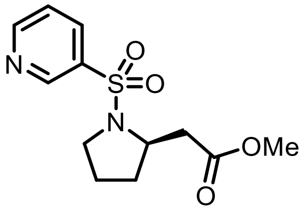

(CHIRALPAK® AD-H, Hexane/IPA 80/20, 1 mL/min)

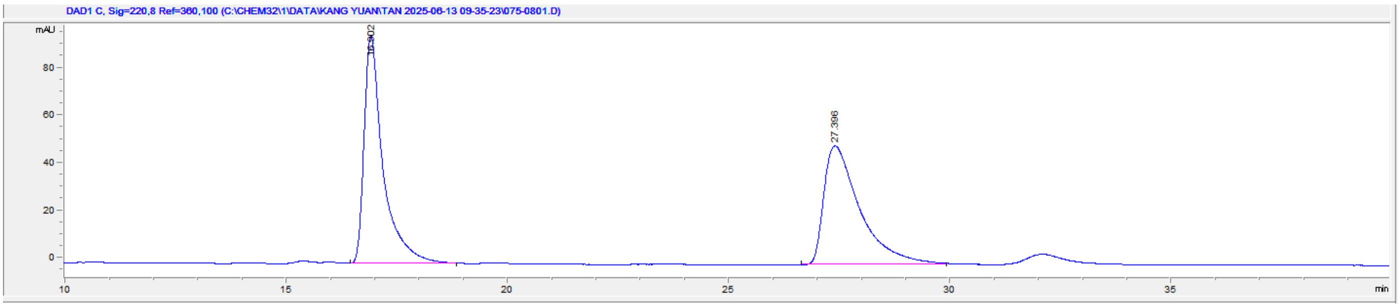

#	Time	Area	Height	Width	Area%	Symmetry
1	16.902	2858.4	95.6	0.4367	50.296	0.489
2	27.396	2824.8	49.9	0.8321	49.704	0.411

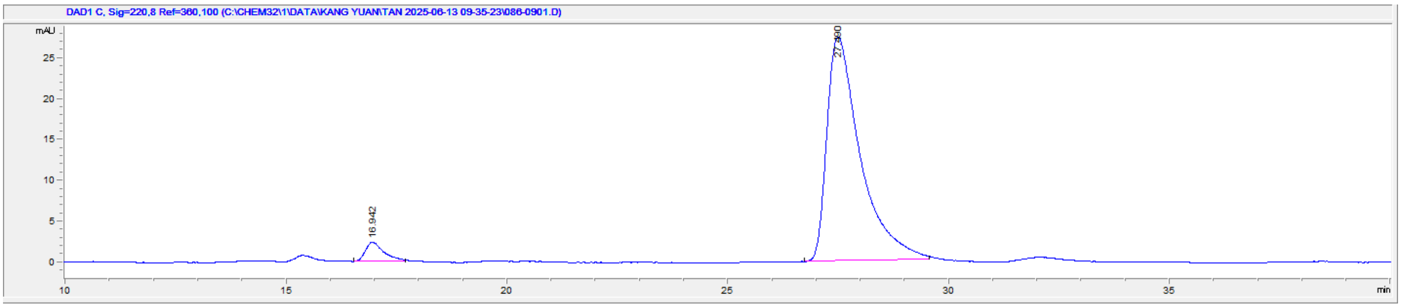

#	Time	Area	Height	Width	Area%	Symmetry
1	16.942	68.6	2.3	0.4305	4.508	0.538
2	27.49	1452.9	27.4	0.7779	95.492	0.454

[back to table of contents]

methyl (*R*)-2-(1-(furan-2-ylsulfonyl)pyrrolidin-2-yl)acetate (8h)

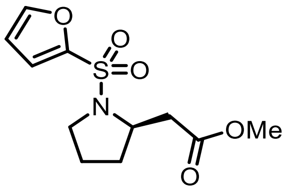

(CHIRALPAK® OD, Hexane/IPA 90/10, 1 mL/min)

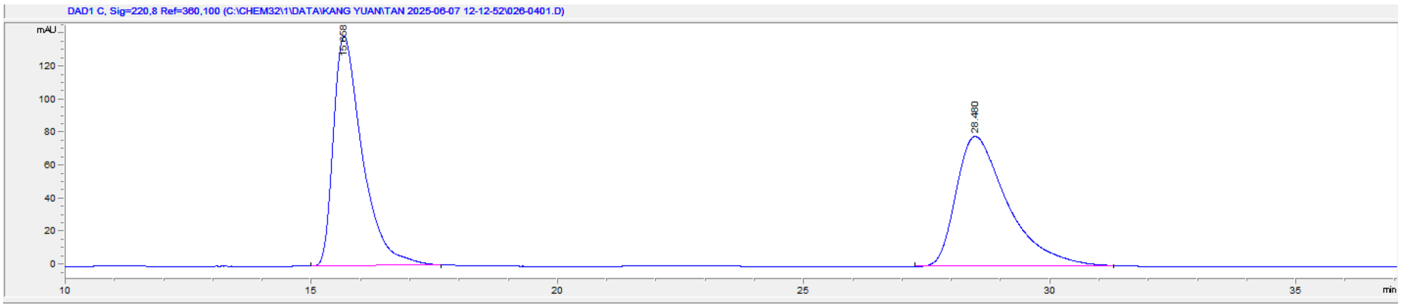

#	Time	Area	Height	Width	Area%	Symmetry
1	15.658	5702.7	139.2	0.6139	49.834	0.533
2	28.48	5740.8	78.8	1.0805	50.166	0.551

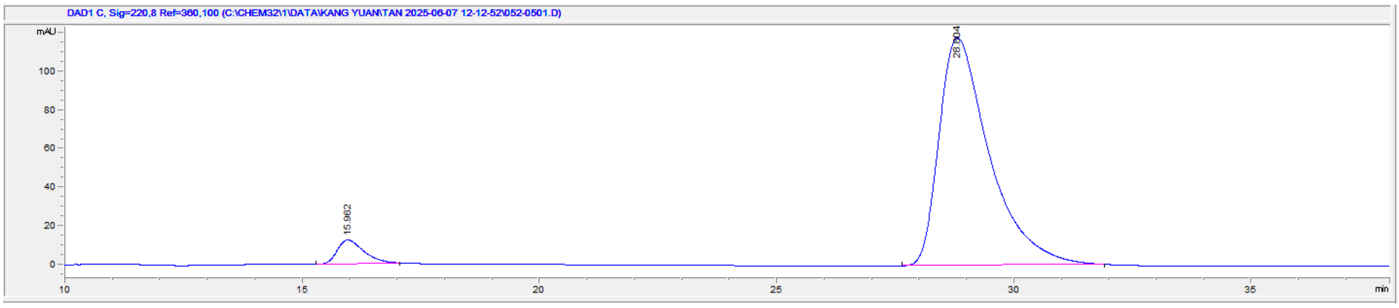

#	Time	Area	Height	Width	Area%	Symmetry
1	15.962	493.7	12.5	0.5812	5.354	0.621
2	28.804	8726.9	118	1.1068	94.646	0.526

[back to table of contents]

methyl (*R*)-2-(1-(thiophen-2-ylsulfonyl)pyrrolidin-2-yl)acetate (8i)

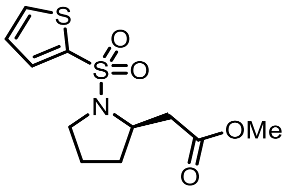

(CHIRALPAK® IA, Hexane/IPA 98/2, 1 mL/min)

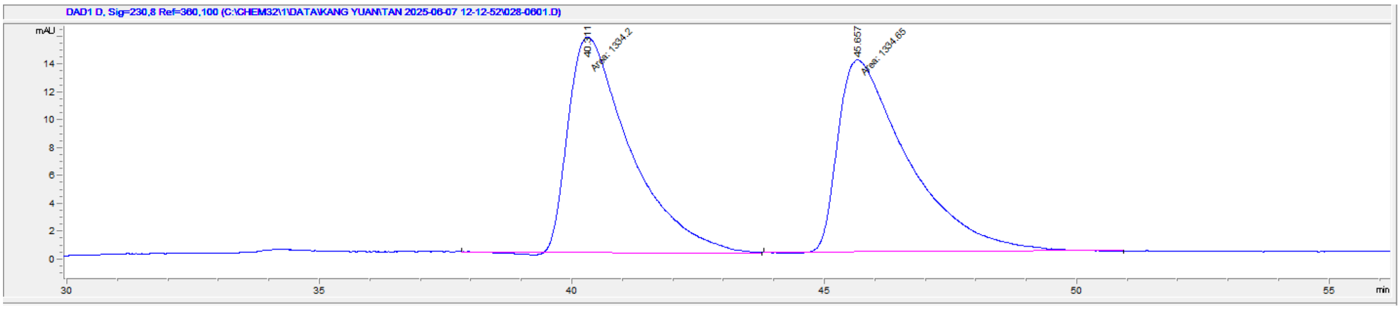

#	Time	Area	Height	Width	Area%	Symmetry
1	40.311	1334.2	15.5	1.4336	49.992	0.448
2	45.657	1334.6	13.8	1.6122	50.008	0.379

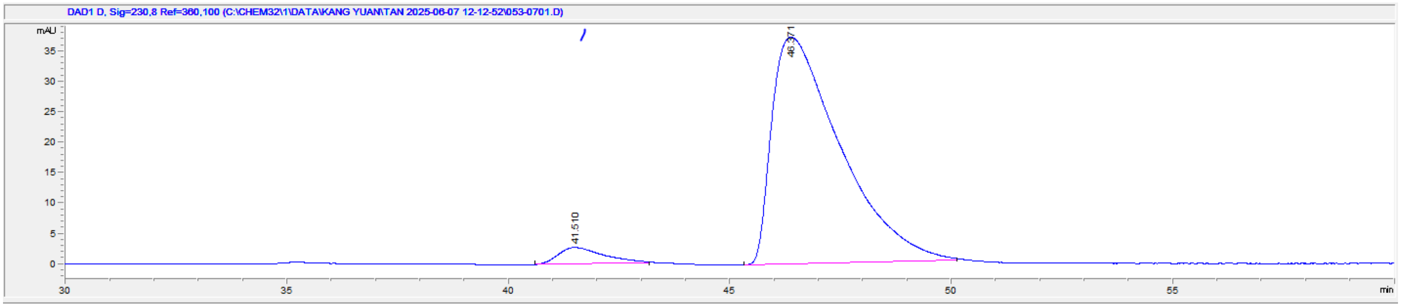

#	Time	Area	Height	Width	Area%	Symmetry
1	41.51	201.6	2.8	0.8698	4.766	0.605
2	46.371	4028.8	37.3	1.4476	95.234	0.358

[back to table of contents]

methyl (*R*)-2-(1-((3,5-dimethylisoxazol-4-yl)sulfonyl)pyrrolidin-2-yl)acetate (8j)

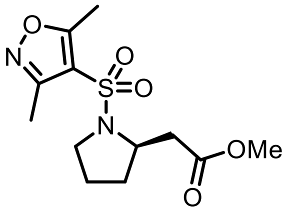

(CHIRALPAK® AS-H, Hexane/IPA 95/5, 1 mL/min)

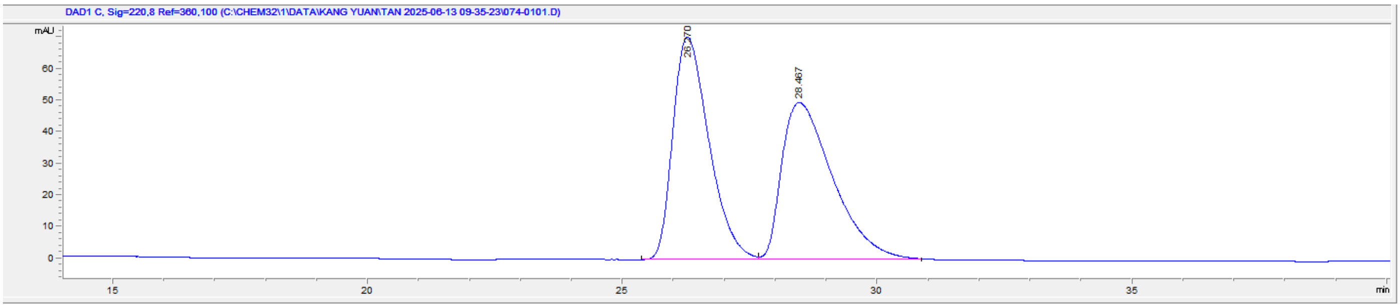

#	Time	Area	Height	Width	Area%	Symmetry
1	26.27	3469.7	70.4	0.7652	50.072	0.632
2	28.467	3459.7	49.7	1.0628	49.928	0.493

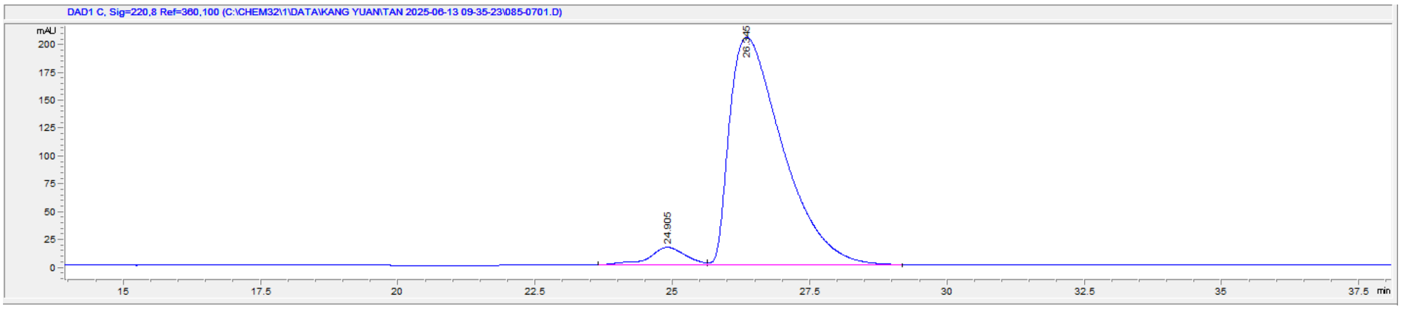

#	Time	Area	Height	Width	Area%	Symmetry
1	24.905	747.7	15.9	0.68	5.146	1.074
2	26.345	13781	204.5	1.0302	94.854	0.437

[back to table of contents]

methyl (*R*)-2-(1-(mesitylsulfonyl)pyrrolidin-2-yl)acetate (8k)

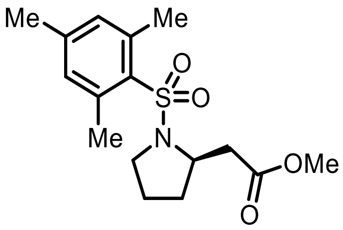

(CHIRALPAK® IA, Hexane/IPA 99/1, 1 mL/min)

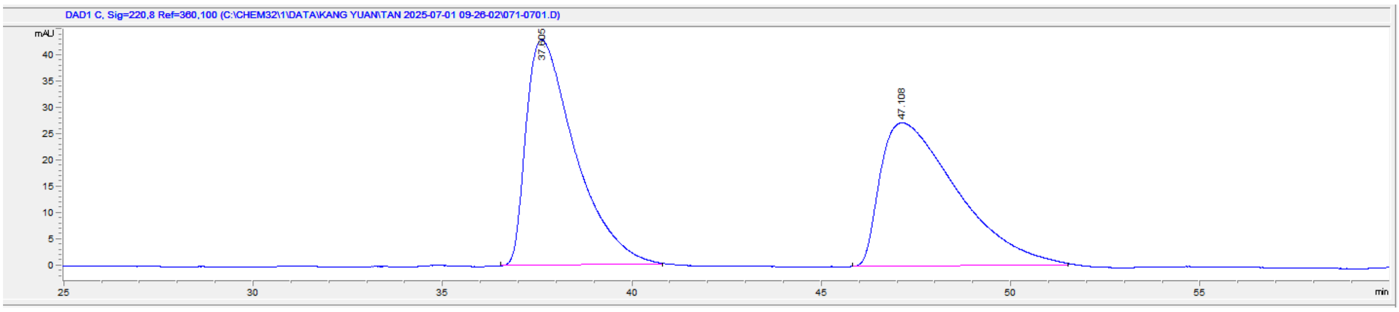

#	Time	Area	Height	Width	Area%	Symmetry
1	37.605	3892	43	1.3404	50.105	0.426
2	47.108	3875.7	27.3	1.7091	49.895	0.355

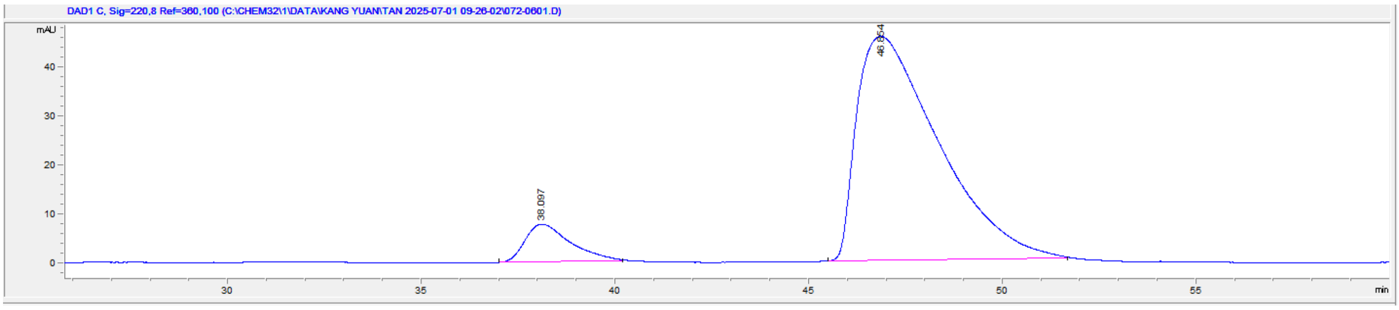

#	Time	Area	Height	Width	Area%	Symmetry
1	38.097	631	7.7	1.0262	8.555	0.522
2	46.854	6744.7	45.8	2.015	91.445	0.384

[back to table of contents]

methyl (*R*)-2-(1-((2,4,6-triisopropylphenyl)sulfonyl)pyrrolidin-2-yl)acetate (8l)

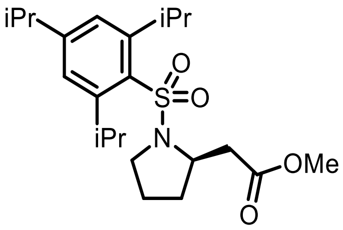

(CHIRALPAK® IA, Hexane/IPA 99/1, 1 mL/min)

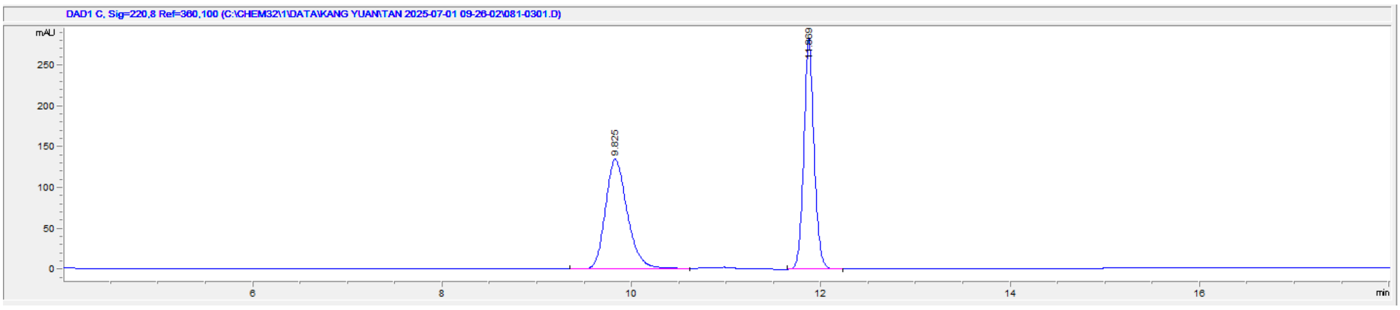

#	Time	Area	Height	Width	Area%	Symmetry
1	9.825	2186.6	134.7	0.2494	50.696	0.747
2	11.869	2126.5	282.5	0.1158	49.304	0.891

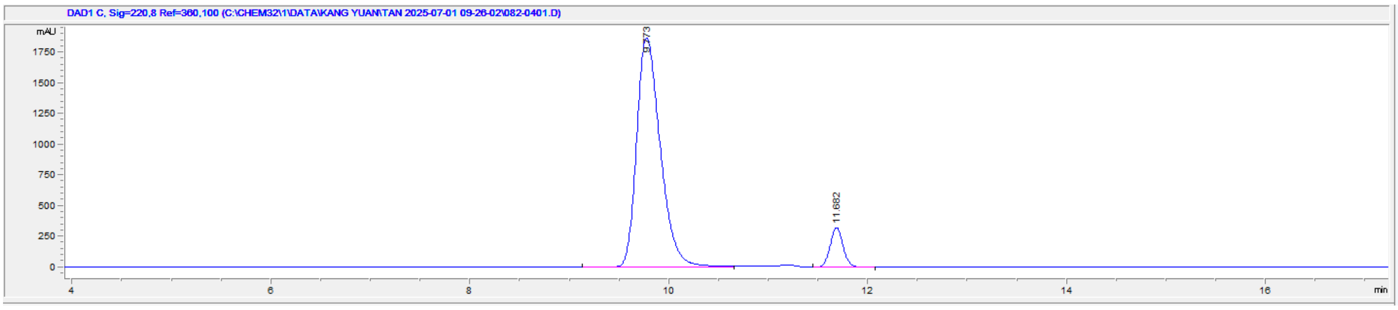

#	Time	Area	Height	Width	Area%	Symmetry
1	9.773	30973.4	1868.3	0.2534	91.463	0.678
2	11.682	2891	321.8	0.1388	8.537	0.939

[back to table of contents]

methyl (*R*)-2-(1-((5-(5-(trifluoromethyl)isoxazol-3-yl)thiophen-2-yl)sulfonyl)pyrrolidin-2-yl)acetate (8m)

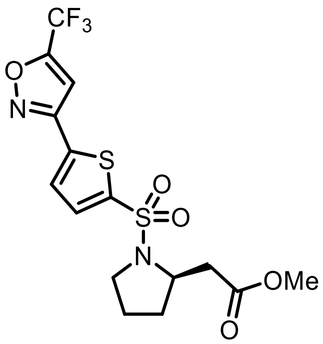

(CHIRALPAK® IA, Hexane/IPA 90/10, 1 mL/min)

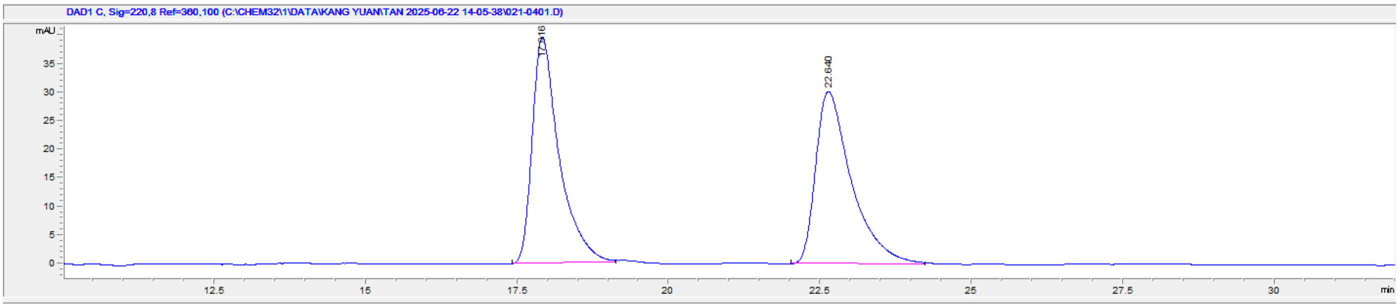

#	Time	Area	Height	Width	Area%	Symmetry
1	17.916	1228.8	39.6	0.4578	49.754	0.554
2	22.64	1241	30.2	0.6119	50.246	0.509

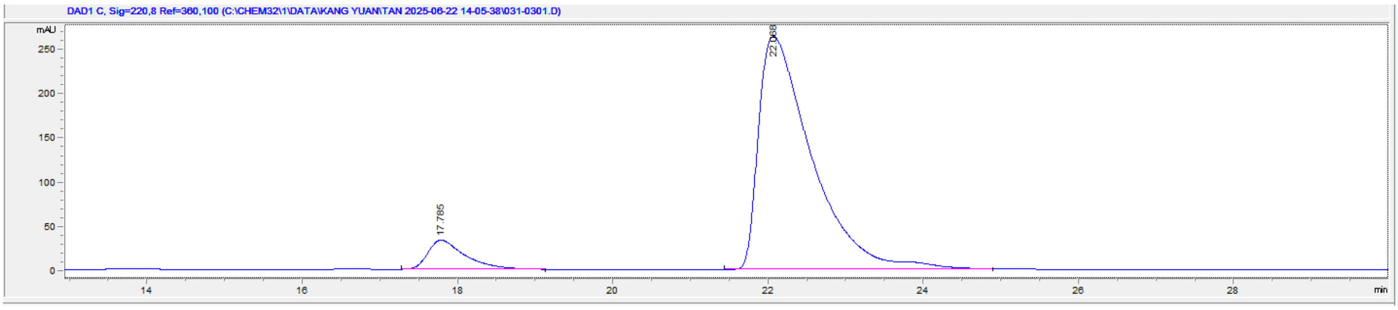

#	Time	Area	Height	Width	Area%	Symmetry
1	17.785	1107.1	33	0.4876	8.136	0.517
2	22.068	12499.6	262.4	0.7078	91.864	0.357

[back to table of contents]

methyl (*R*)-2-(2-(benzylsulfonyl)-2-azaspiro[4.5]decan-3-yl)acetate (8n)

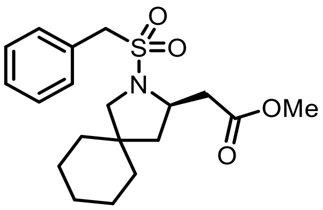

(CHIRALPAK® AD-H, Hexane/IPA 95/5, 1 mL/min)

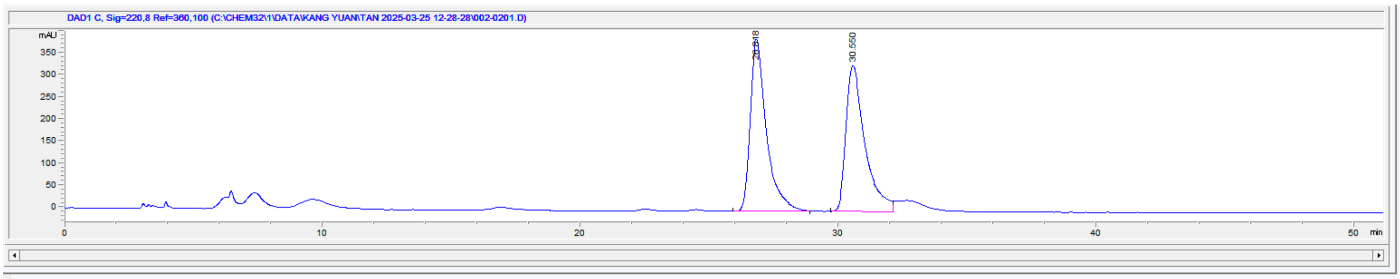

#	Time	Area	Height	Width	Area%	Symmetry
1	26.818	17175.7	390.4	0.6513	50.299	0.583
2	30.55	16971.7	330.8	0.7325	49.701	0.515

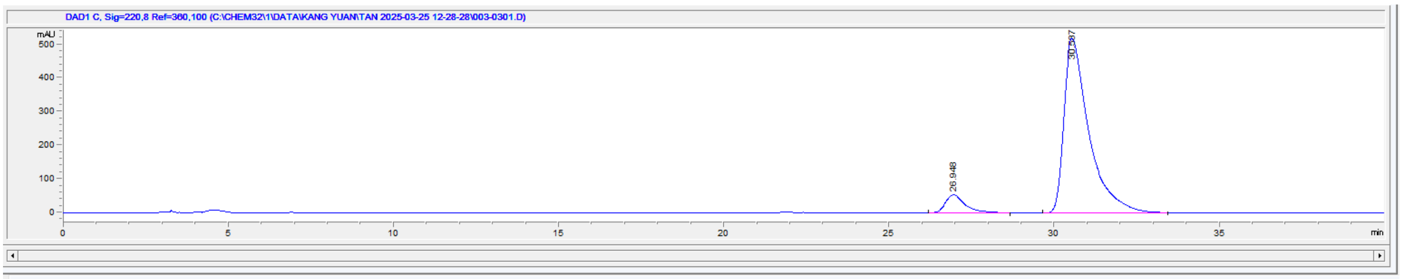

#	Time	Area	Height	Width	Area%	Symmetry
1	26.948	2311.6	54.2	0.6037	7.770	0.644
2	30.537	27440	519.3	0.7665	92.230	0.464

[back to table of contents]

HPLC Data from Preparative Scale Procedure (Before Recrystallisation)

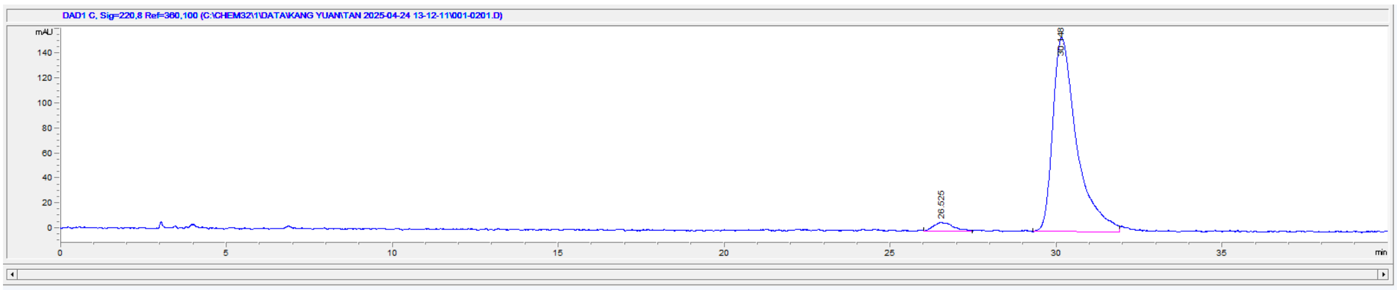

#	Time	Area	Height	Width	Area%	Symmetry
1	26.525	331.4	7.4	0.5388	4.053	0.572
2	30.148	7845.5	156.1	0.7302	95.947	0.518

HPLC Data from Preparative Scale Procedure (After Recrystallisation)

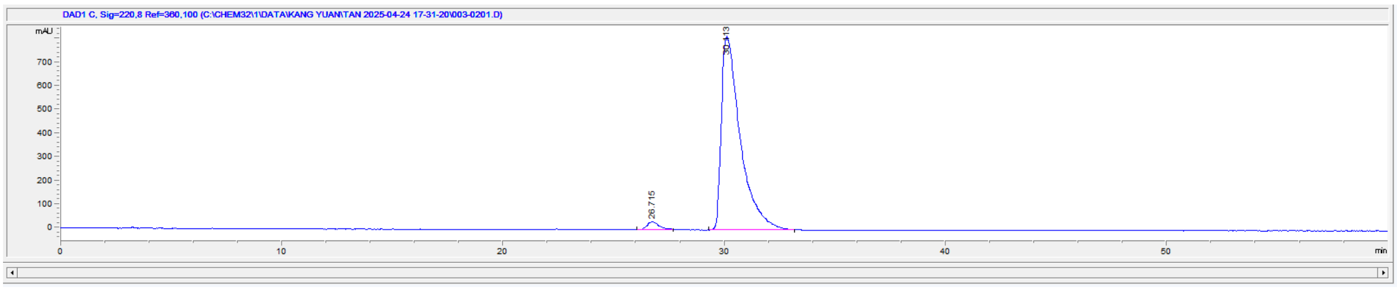

#	Time	Area	Height	Width	Area%	Symmetry
1	26.715	1390.7	33.9	0.525	2.766	0.641
2	30.113	48886.3	819	0.7904	97.234	0.387

[back to table of contents]

methyl (*R*)-2-(2-(benzylsulfonyl)-8-oxa-2-azaspiro[4.5]decan-3-yl)acetate (8o)

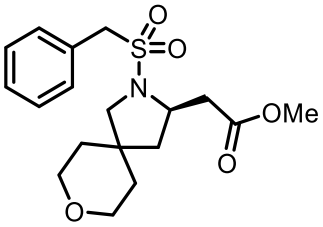

(CHIRALCEL® OD, Hexane/IPA 80/20, 1 mL/min)

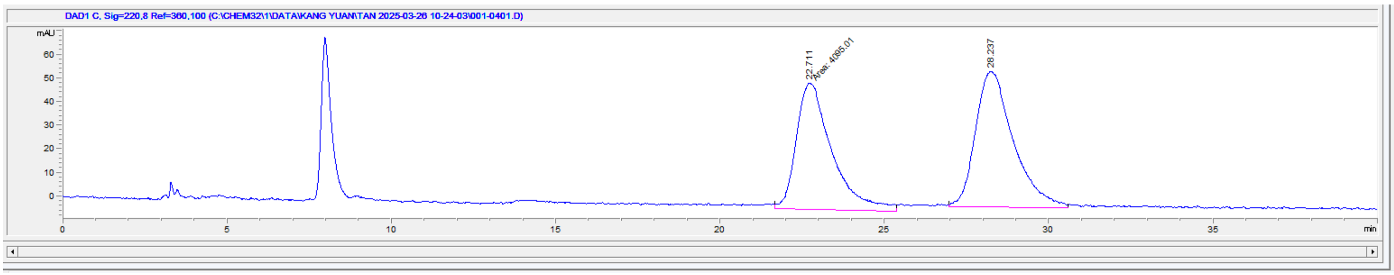

#	Time	Area	Height	Width	Area%	Symmetry
1	22.711	4095	53.9	1.2668	47.029	0.563
2	28.237	4612.4	57.6	0.9603	52.971	0.674

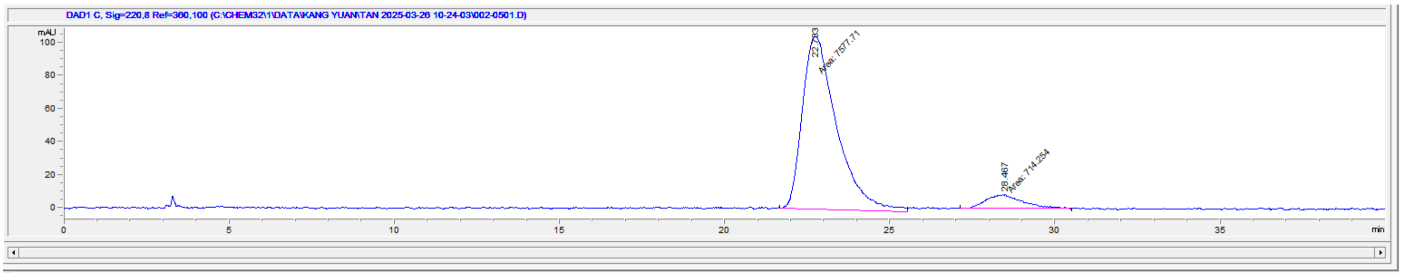

#	Time	Area	Height	Width	Area%	Symmetry
1	22.733	7577.7	105	1.2028	91.386	0.574
2	28.467	714.3	8.7	1.37	8.614	0.949

[back to table of contents]

tert-butyl (*R*)-2-(benzylsulfonyl)-3-(2-methoxy-2-oxoethyl)-2,8-diazaspiro[4.5]decane-8-carboxylate (8p)

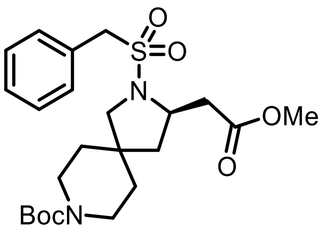

(CHIRALPAK® AD-H, Hexane/IPA 80/20, 1 mL/min)

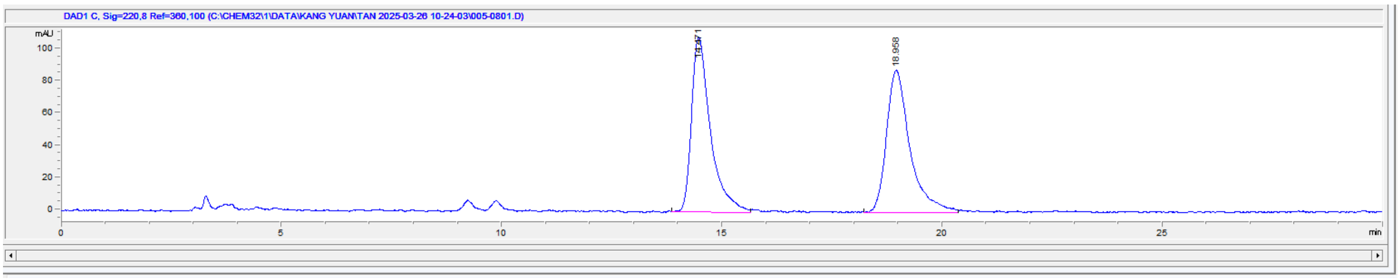

#	Time	Area	Height	Width	Area%	Symmetry
1	14.471	3280.9	108.9	0.4436	49.819	0.565
2	18.958	3304.8	88.7	0.5226	50.181	0.656

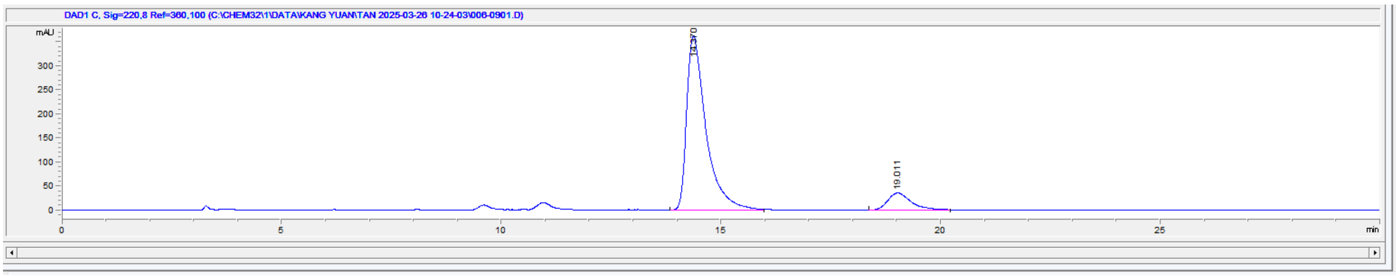

#	Time	Area	Height	Width	Area%	Symmetry
1	14.37	11182.7	362.2	0.4622	88.817	0.487
2	19.011	1408	36.5	0.5496	11.183	0.645

[back to table of contents]

methyl (*R*)-2-(2-(benzylsulfonyl)-2-azaspiro[4.4]nonan-3-yl)acetate (8q)

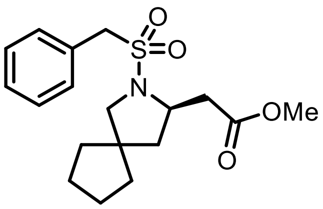

(CHIRALPAK® AD-H, Hexane/IPA 90/10, 1 mL/min)

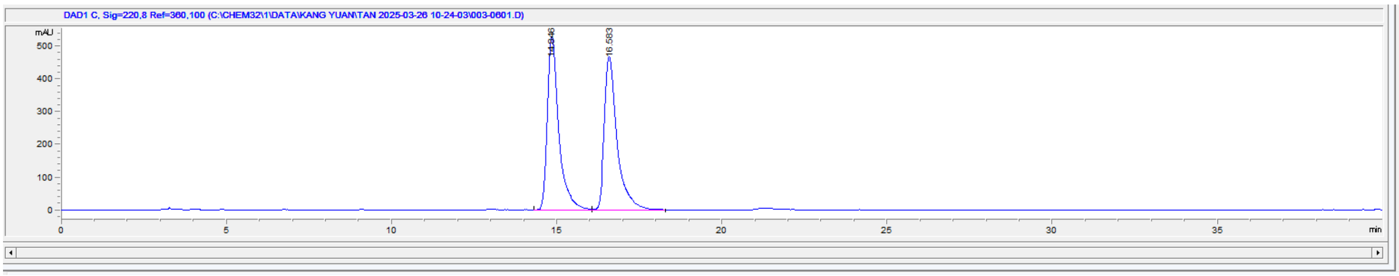

#	Time	Area	Height	Width	Area%	Symmetry
1	14.846	12640.7	531.6	0.3514	49.893	0.591
2	16.583	12695.1	469.5	0.3955	50.107	0.575

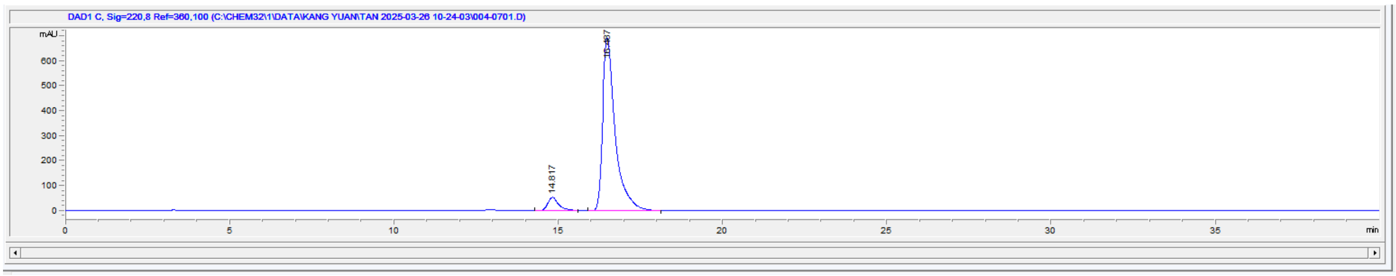

#	Time	Area	Height	Width	Area%	Symmetry
1	14.817	1262.4	54.4	0.3408	6.332	0.67
2	16.487	18673.4	692.9	0.4024	93.668	0.554

[back to table of contents]

methyl (*R*)-2-(6-(benzylsulfonyl)-6-azaspiro[3.4]octan-7-yl)acetate (8r)

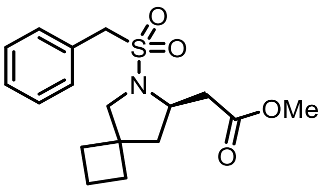

(CHIRALPAK® IA, Hexane/IPA 95/5, 1 mL/min)

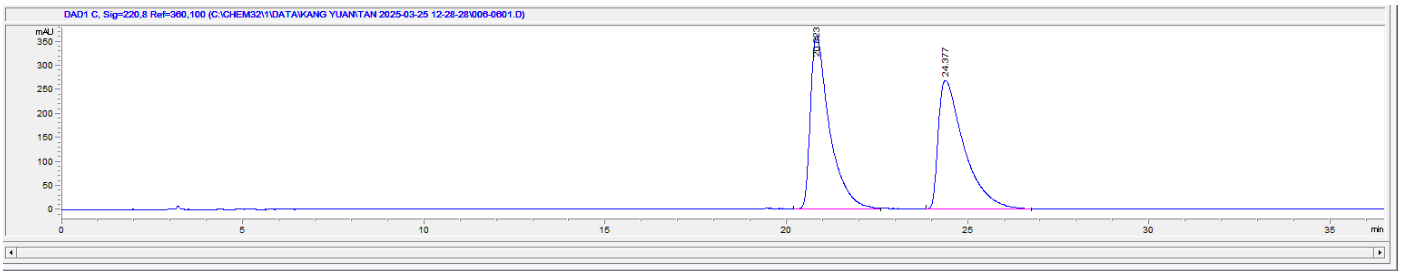

#	Time	Area	Height	Width	Area%	Symmetry
1	20.823	13455.6	362.2	0.5394	50.147	0.427
2	24.377	13376.7	269.1	0.6998	49.853	0.35

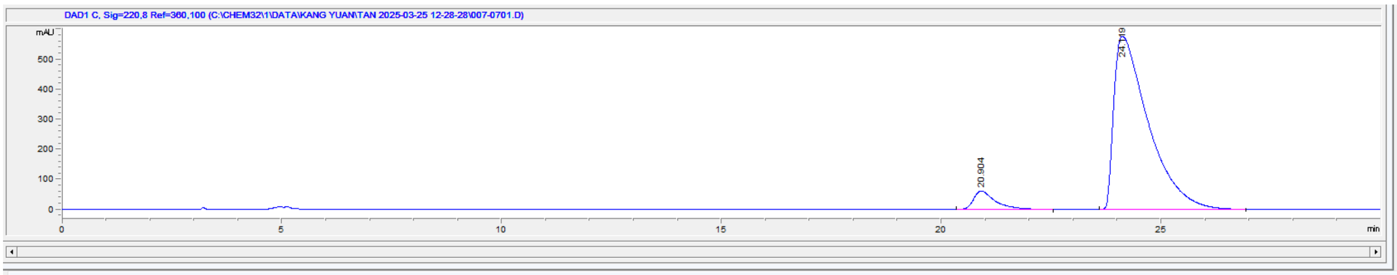

#	Time	Area	Height	Width	Area%	Symmetry
1	20.904	2250	62.1	0.5104	6.639	0.485
2	24.119	31638.8	577.2	0.7615	93.361	0.307

[back to table of contents]

methyl (*R*)-2-(1-(benzylsulfonyl)-4,4-diphenylpyrrolidin-2-yl)acetate (8s)

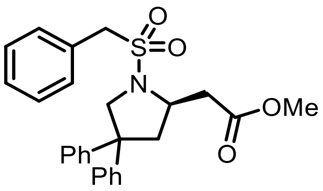

(CHIRALPAK® IA, Hexane/IPA 80/20, 1 mL/min)

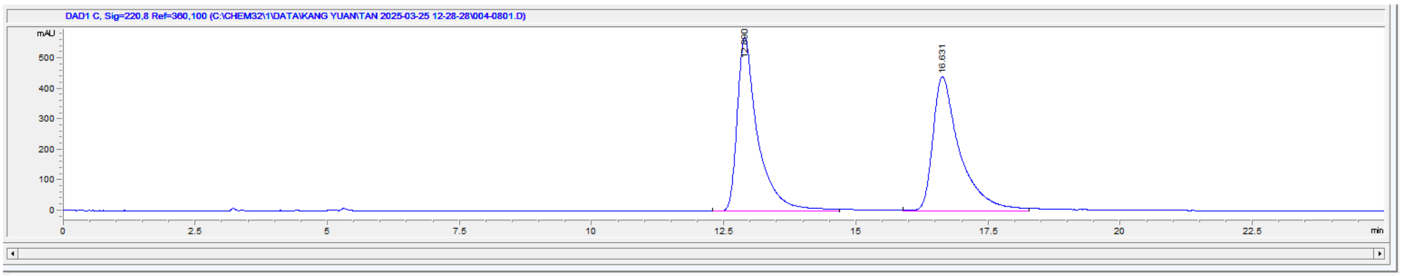

#	Time	Area	Height	Width	Area%	Symmetry
1	12.89	15636.6	571.5	0.3952	49.660	0.501
2	16.631	15850.7	441.6	0.5128	50.340	0.497

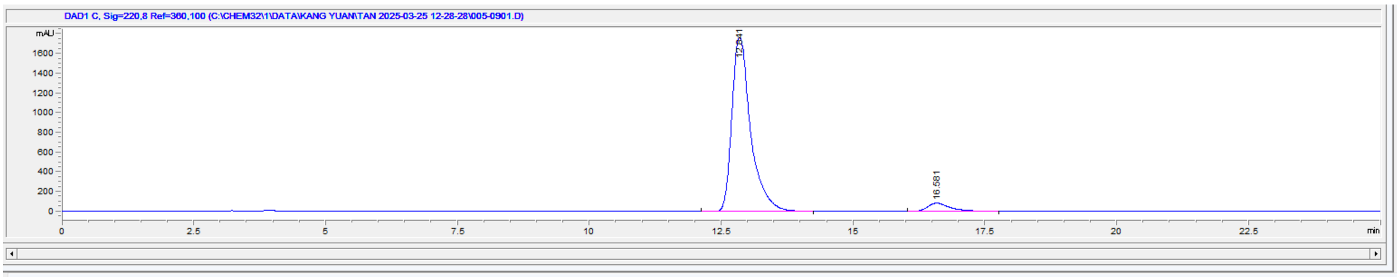

#	Time	Area	Height	Width	Area%	Symmetry
1	12.841	43834.5	1767.8	0.3733	94.349	0.625
2	16.581	2625.5	84.5	0.4706	5.651	0.593

[back to table of contents]

methyl (*R*)-2-(5,5-diphenyl-1-(phenylsulfonyl)piperidin-2-yl)acetate (8t)

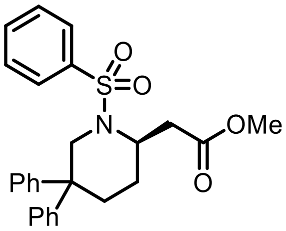

(CHIRALPAK® OD, Hexane/IPA 95/5, 1 mL/min)

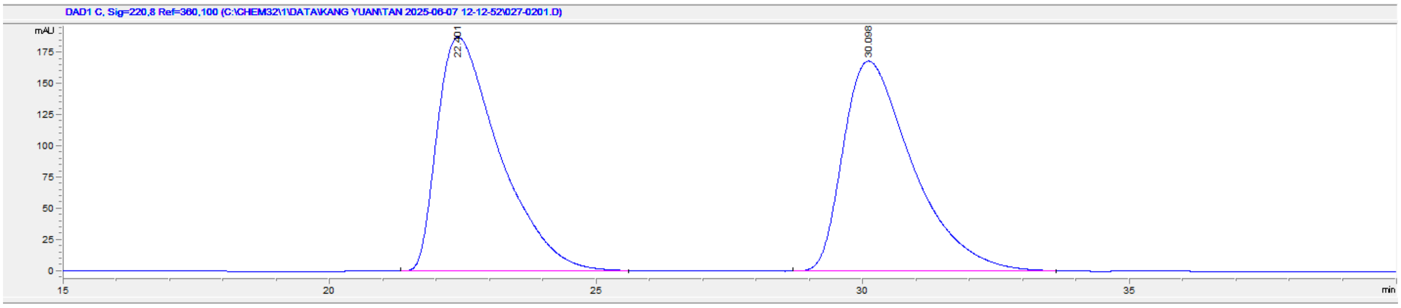

#	Time	Area	Height	Width	Area%	Symmetry
1	22.401	15221.7	187.4	1.2295	49.934	0.467
2	30.098	15261.7	168.4	1.3736	50.066	0.533

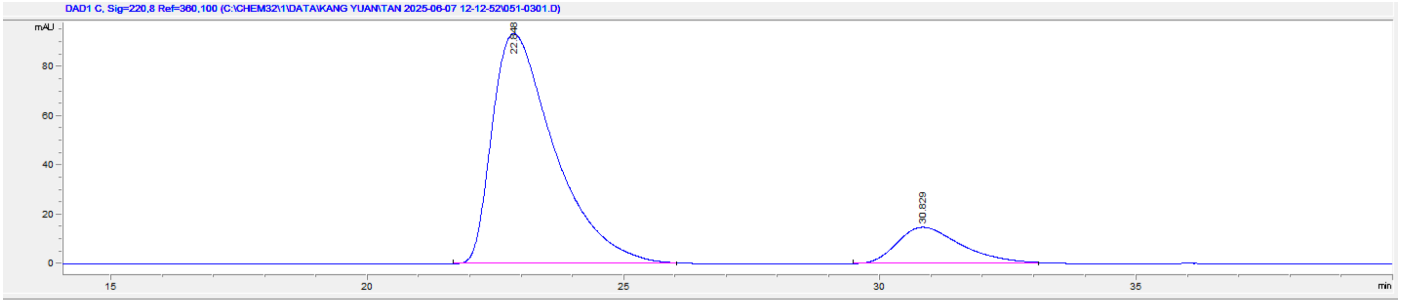

#	Time	Area	Height	Width	Area%	Symmetry
1	22.848	7896.7	93.5	1.2636	85.986	0.491
2	30.829	1287	14.8	1.1693	14.014	0.642

[back to table of contents]

methyl (*R*)-2-(1-(benzylsulfonyl)-5,5-dimethylpyrrolidin-2-yl)acetate (8u)

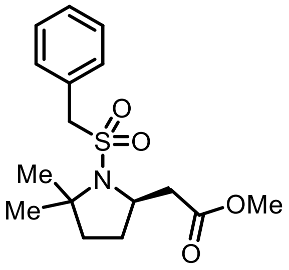

(CHIRALCEL® OD, Hexane/IPA 90/10, 1 mL/min)

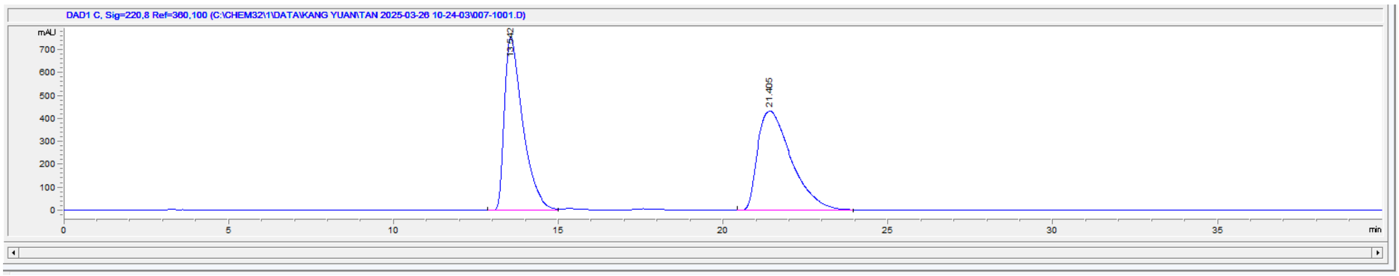

#	Time	Area	Height	Width	Area%	Symmetry
1	13.542	29905.6	759.6	0.5773	49.086	0.487
2	21.405	31019.2	433.5	1.0289	50.914	0.532

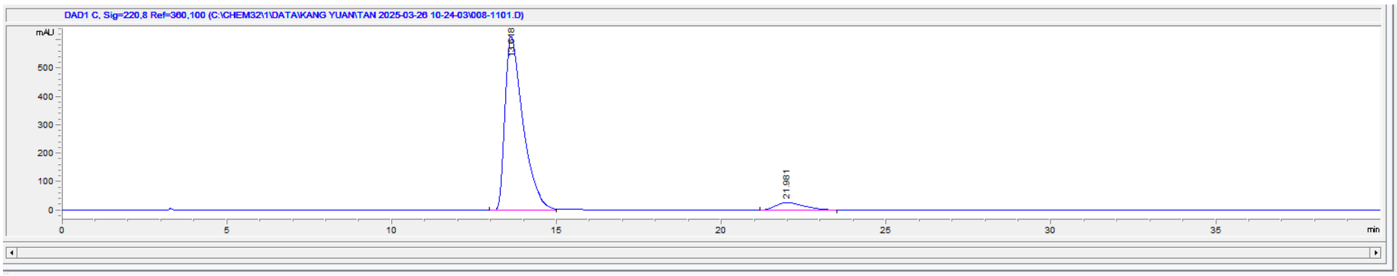

#	Time	Area	Height	Width	Area%	Symmetry
1	13.618	23768.7	613.5	0.58	93.027	0.51
2	21.981	1781.6	28.1	0.7697	6.973	0.629

[back to table of contents]

methyl (*R*)-2-(5-(benzylsulfonyl)-5-azaspiro[3.4]octan-6-yl)acetate (8v)

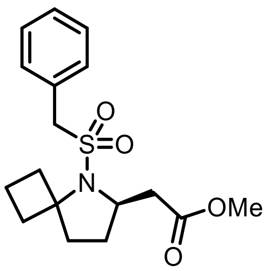

(CHIRALPAK® IA, Hexane/IPA 98/2, 1 mL/min)

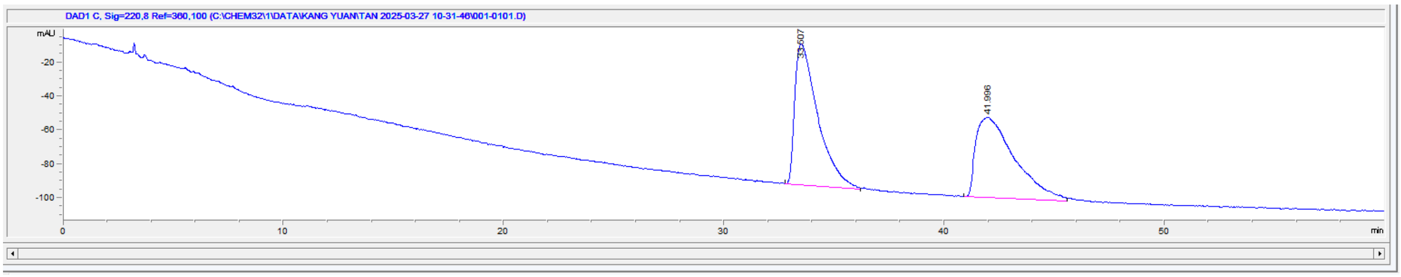

#	Time	Area	Height	Width	Area%	Symmetry
1	33.507	6015.6	83.2	0.9977	50.807	0.349
2	41.996	5824.6	47.4	1.4673	49.193	0.438

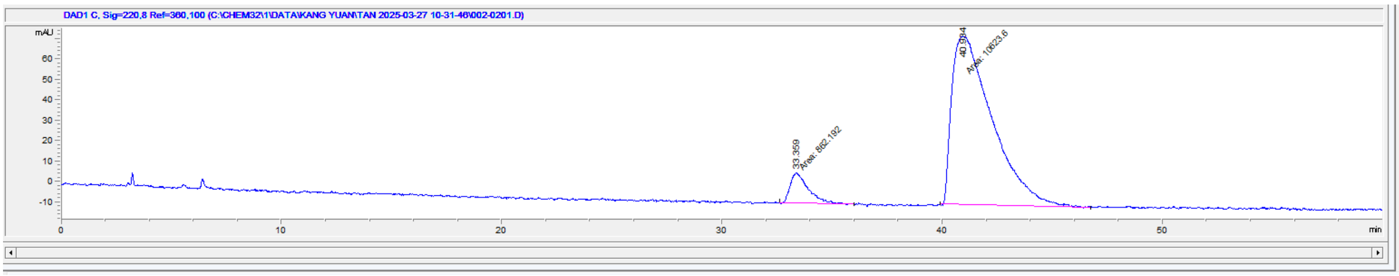

#	Time	Area	Height	Width	Area%	Symmetry
1	33.359	862.2	14.9	0.9631	7.507	0.452
2	40.934	10623.6	82.8	2.1387	92.493	0.361

[back to table of contents]

methyl (*R*)-2-(1-(benzylsulfonyl)-8-oxa-1-azaspiro[4.5]decan-2-yl)acetate (8w)

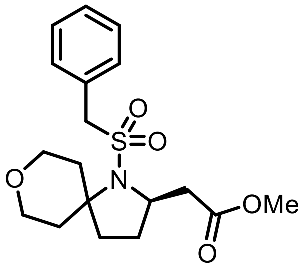

(CHIRALPAK® IA, Hexane/IPA 90/10, 1 mL/min)

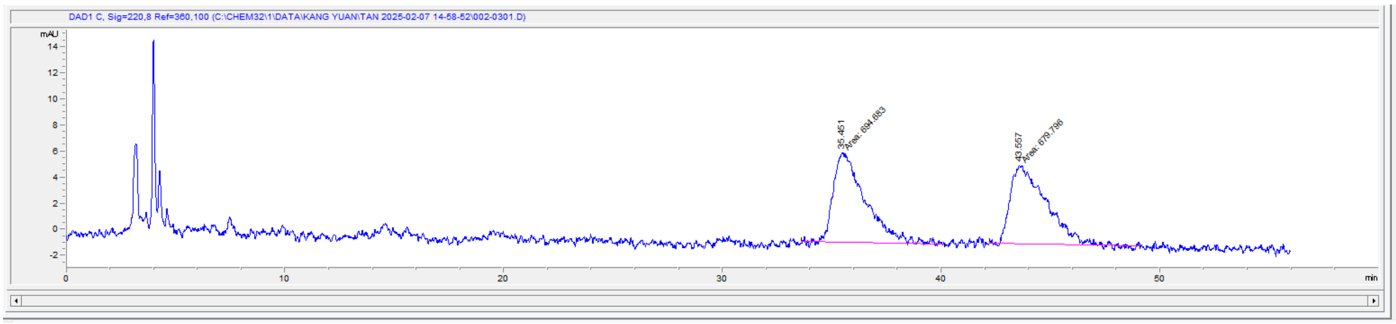

#	Time	Area	Height	Width	Area%	Symmetry
1	35.451	694.7	6.9	1.6902	50.542	0.374
2	43.557	679.8	6	1.8833	49.458	0.331

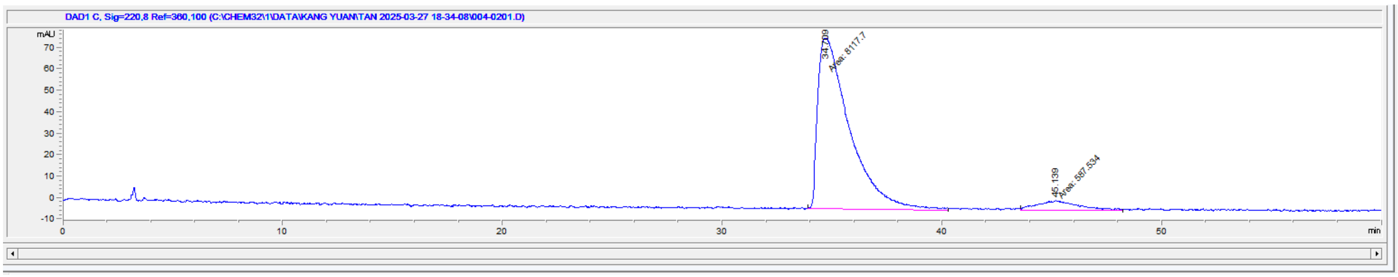

#	Time	Area	Height	Width	Area%	Symmetry
1	34.709	8117.7	79.9	1.6926	93.251	0.317
2	45.139	587.5	4.5	2.1739	6.749	0.751

[back to table of contents]

methyl 2-((2*R*)-1-(benzylsulfonyl)-5-phenylpyrrolidin-2-yl)acetate (8x)

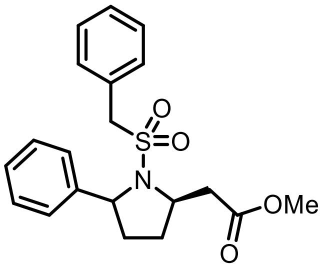

(CHIRALPAK® IH-3, Hexane/IPA 95/5, 0.3 mL/min)

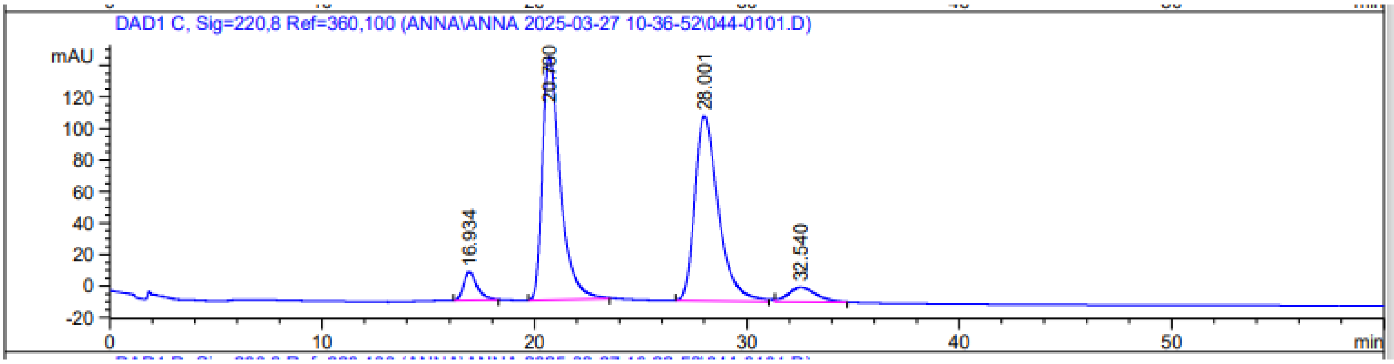

Signal 3: DAD1 C, Sig=220,8 Ref=360,100

Peak #	RetTime [min]	Type	Width [min]	Area [mAU*s]	Height [mAU]	Area %
1	16.934	BB	0.6662	798.25488	18.04235	4.0647
2	20.700	BB	0.8681	8953.29785	154.03552	45.5904
3	28.001	BB	1.1674	9046.66211	117.34631	46.0658
4	32.540	BB	1.3372	840.36554	9.23857	4.2792

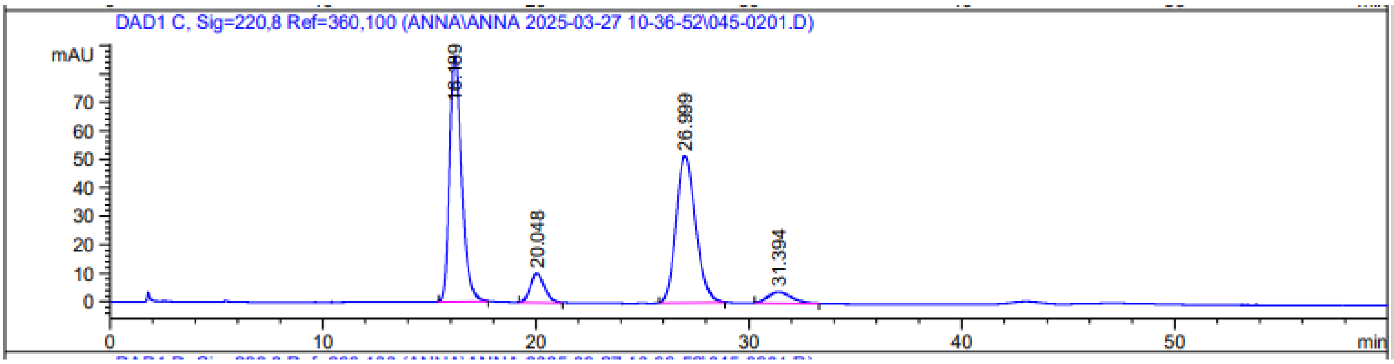

Signal 3: DAD1 C, Sig=220,8 Ref=360,100

Peak #	RetTime [min]	Type	Width [min]	Area [mAU*s]	Height [mAU]	Area %
1	16.189	BB	0.6010	3396.04175	86.32788	45.2121
2	20.048	BB	0.7119	477.32269	10.27578	6.3547
3	26.999	BB	0.9932	3329.50464	51.70626	44.3263
4	31.394	BB	1.1181	308.48074	3.97189	4.1069

[back to table of contents]

methyl (E)-5-((5*R*)-1-(benzylsulfonyl)-5-(2-methoxy-2-oxoethyl)pyrrolidin-2-yl)pent-2-enoate (8y)

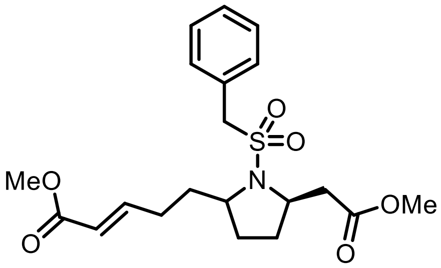

(CHIRALPAK® AD, Hexane/IPA 90/10, 1 mL/min)

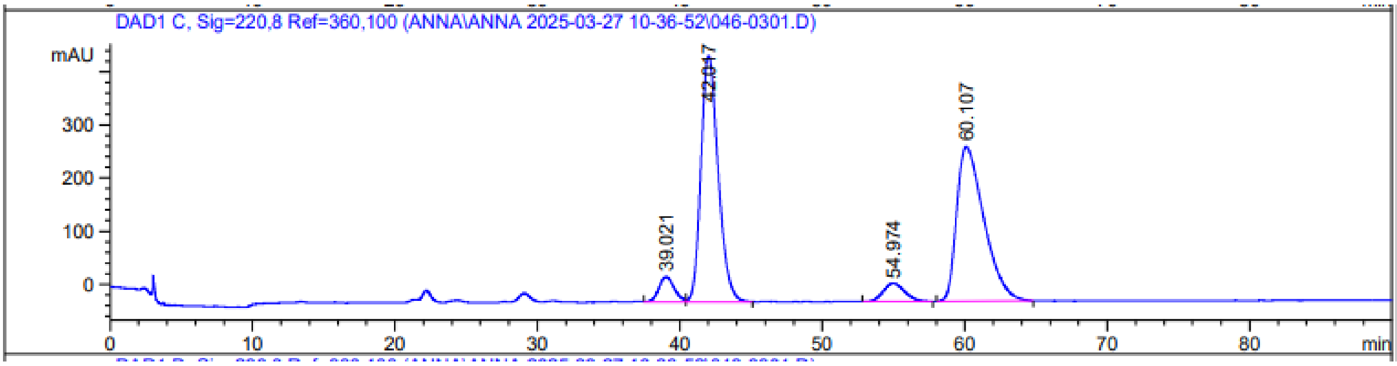

Signal 3: DAD1 C, Sig=220,8 Ref=360,100

Peak #	RetTime [min]	Type	Width [min]	Area [mAU*s]	Height [mAU]	Area %
1	39.021	VV	1.1846	3642.57690	47.61259	4.2743
2	42.017	VB	1.2930	3.90292e4	464.17209	45.7980
3	54.974	VB	1.5055	3625.85620	34.63095	4.2547
4	60.107	BB	2.0405	3.89227e4	291.74637	45.6730

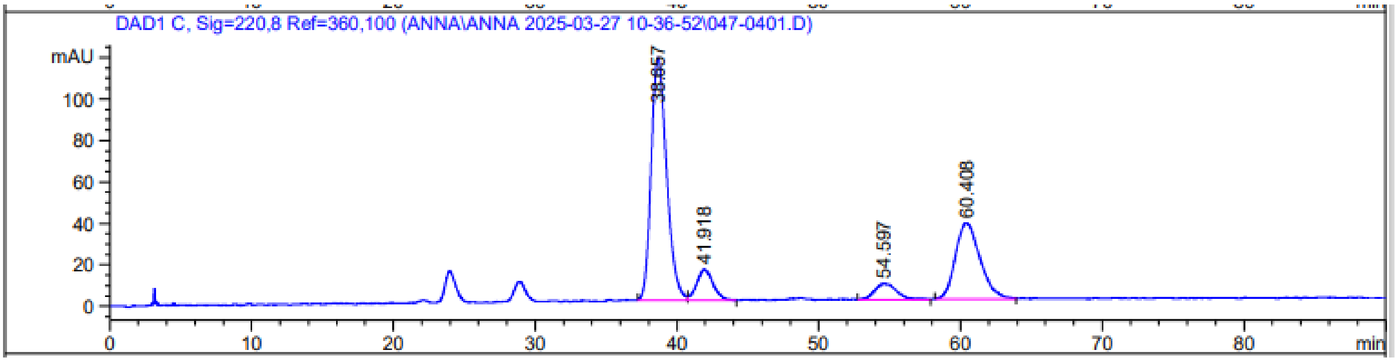

Signal 3: DAD1 C, Sig=220,8 Ref=360,100

Peak #	RetTime [min]	Type	Width [min]	Area [mAU*s]	Height [mAU]	Area %
1	38.657	BV	1.1759	8831.42871	117.10085	57.6497
2	41.918	VB	1.2410	1173.01843	14.73081	7.6572
3	54.597	VB	1.3716	844.64825	7.84843	5.5137
4	60.408	BB	1.7019	4470.02539	36.78100	29.1794

[back to table of contents]

methyl (*R*)-2-(1-(benzylsulfonyl)indolin-2-yl)acetate (8z)

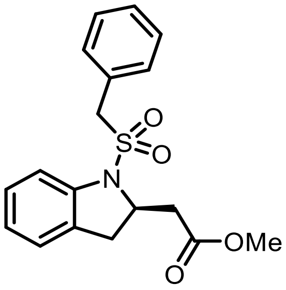

(CHIRALPAK® IA, Hexane/IPA 90/10, 1 mL/min)

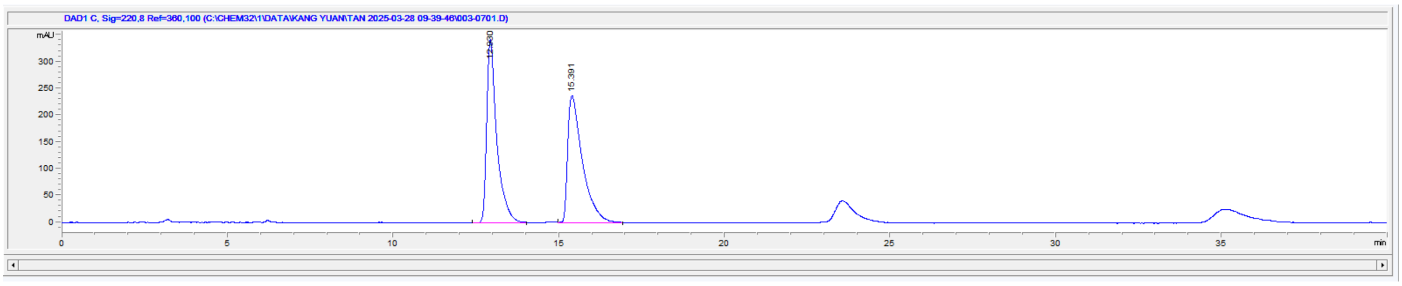

#	Time	Area	Height	Width	Area%	Symmetry
1	12.93	7366.6	342.9	0.3146	49.879	0.512
2	15.391	7402.3	238.5	0.458	50.121	0.355

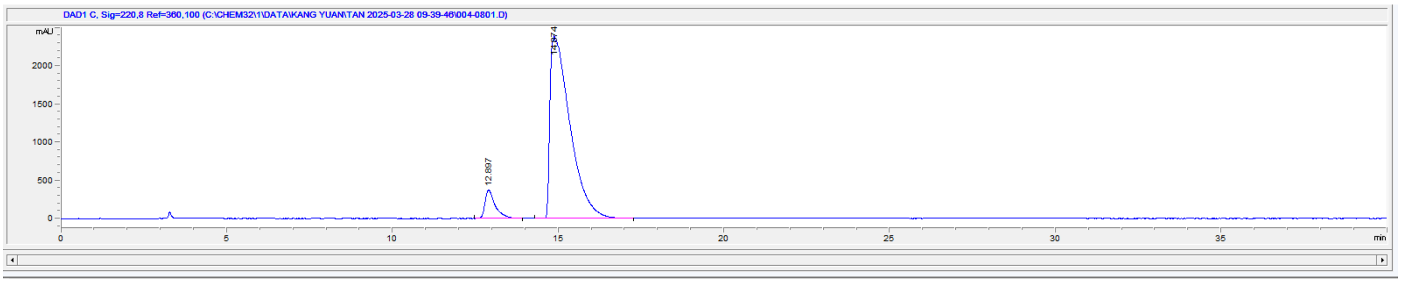

#	Time	Area	Height	Width	Area%	Symmetry
1	12.897	8123.7	375.6	0.3163	7.478	0.505
2	14.874	100515.7	2393.1	0.504	92.522	0.255

[back to table of contents]

methyl (*R*)-2-(5-bromo-1-(phenylsulfonyl)indolin-2-yl)acetate (8aa)

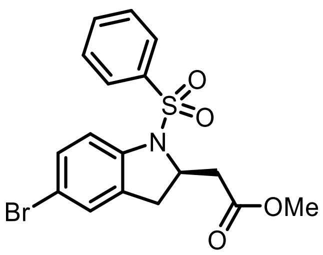

(CHIRALPAK® OD, Hexane/IPA 95/5, 1 mL/min)

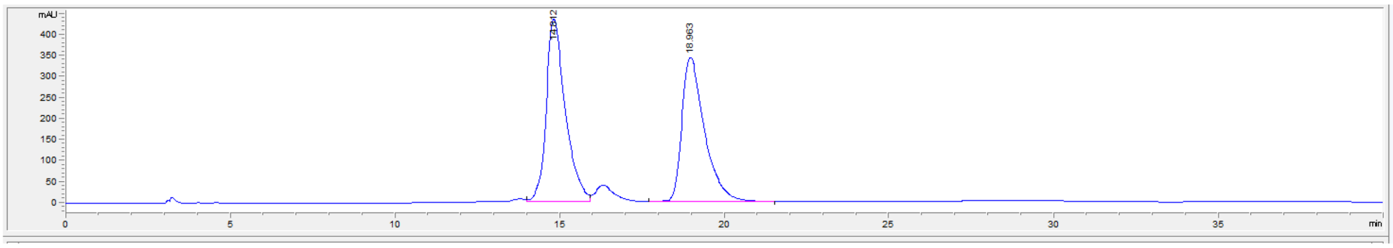

#	Time	Area	Height	Width	Area%	Symmetry
1	14.812	17636	434.9	0.6011	51.796	0.617
2	18.963	16412.8	343.4	0.7137	48.204	0.564

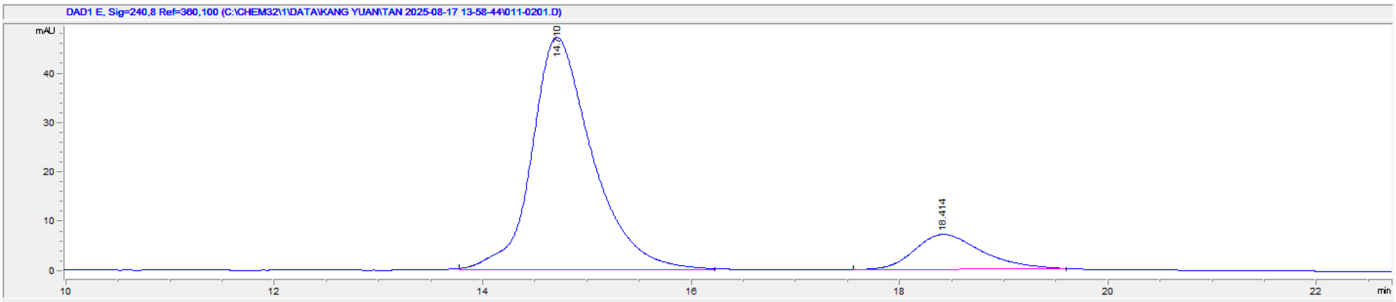

#	Time	Area	Height	Width	Area%	Symmetry
1	14.71	1902.4	47.1	0.595	85.209	0.682
2	18.414	330.2	7.1	0.6911	14.791	0.691

[back to table of contents]

methyl (*R*)-2-(5-methoxy-1-(phenylsulfonyl)indolin-2-yl)acetate (8ab)

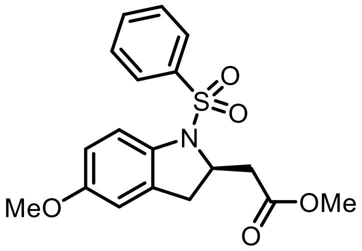

(CHIRALPAK® OD, Hexane/IPA 95/5, 1 mL/min)

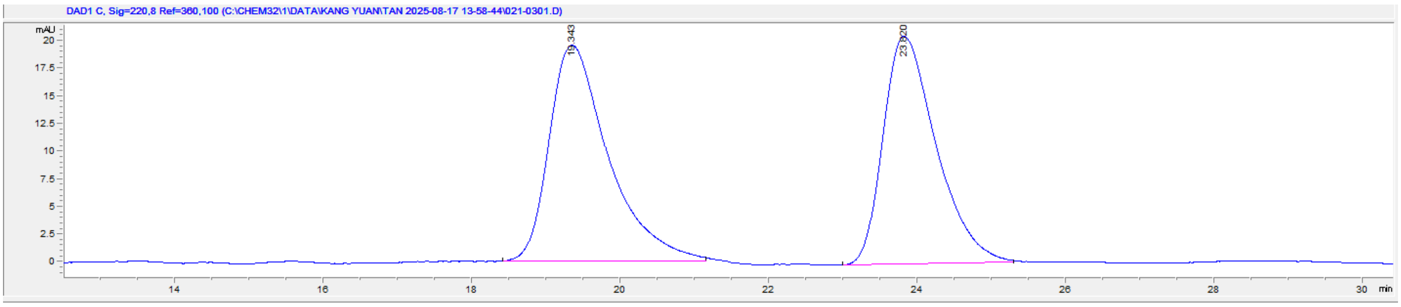

#	Time	Area	Height	Width	Area%	Symmetry
1	19.343	1089.7	19.6	0.832	51.566	0.576
2	23.82	1023.5	20.6	0.7513	48.434	0.613

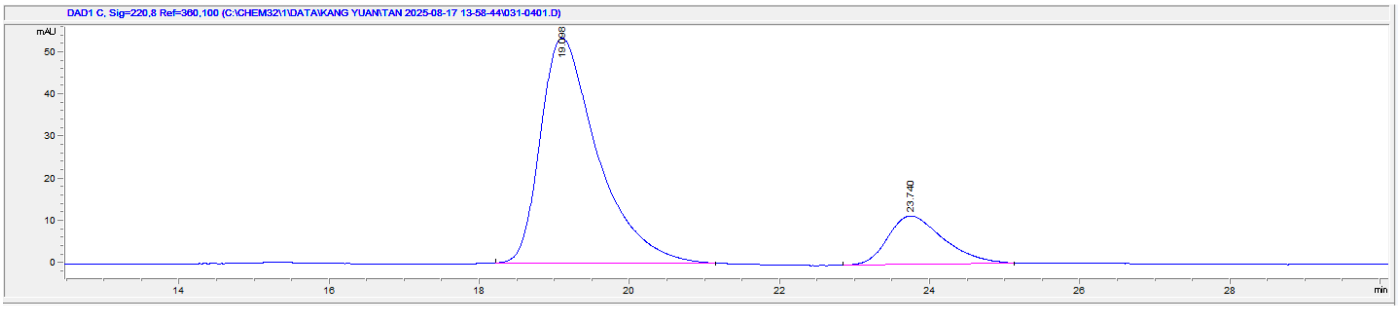

#	Time	Area	Height	Width	Area%	Symmetry
1	19.098	2841.3	53.5	0.7976	82.647	0.562
2	23.74	596.6	11.5	0.7693	17.353	0.657

[back to table of contents]

methyl (*R*)-2-(5-methyl-1-(phenylsulfonyl)indolin-2-yl)acetate (8ac)

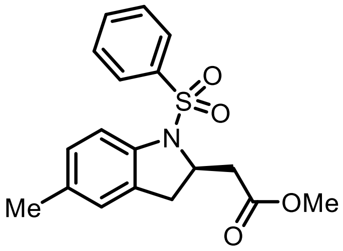

(CHIRALPAK® OD, Hexane/IPA 95/5, 1 mL/min)

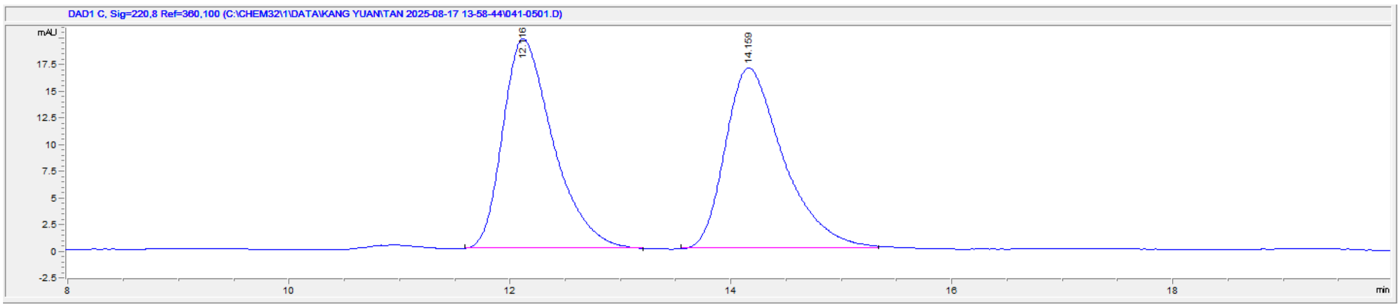

#	Time	Area	Height	Width	Area%	Symmetry
1	12.116	620.9	19.6	0.4793	50.014	0.635
2	14.159	620.6	16.9	0.5522	49.986	0.627

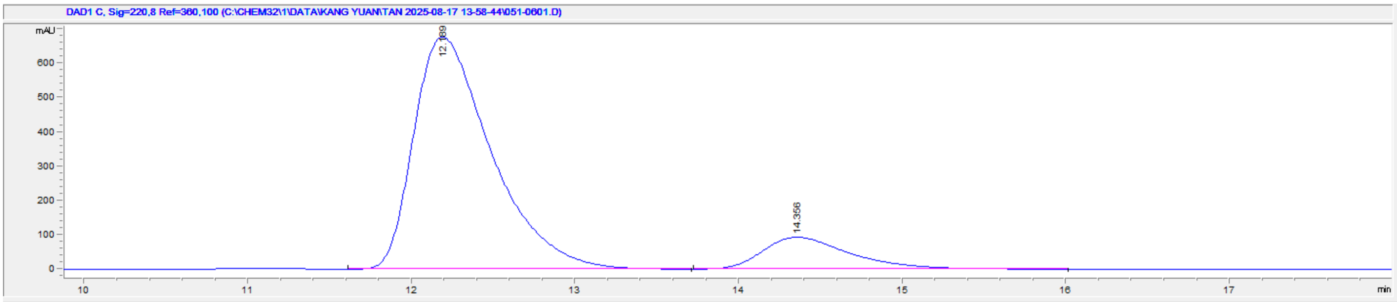

#	Time	Area	Height	Width	Area%	Symmetry
1	12.189	22308	676.1	0.501	86.477	0.553
2	14.356	3488.5	93.1	0.5631	13.523	0.606

[back to table of contents]

methyl (*S*)-2-(2-(benzylsulfonyl)isoindolin-1-yl)acetate (8ad)

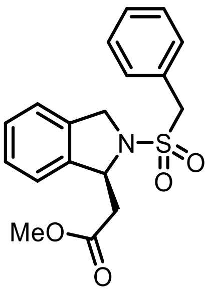

(CHIRALPAK® IA, Hexane/IPA 90/10, 1 mL/min)

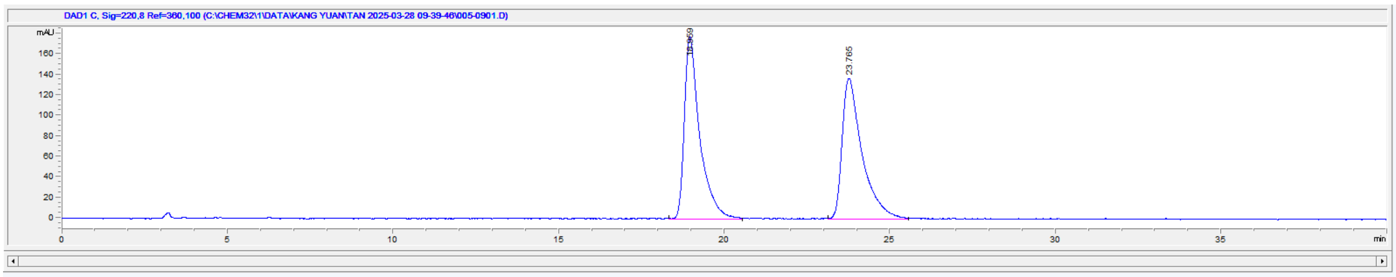

#	Time	Area	Height	Width	Area%	Symmetry
1	18.959	5809	177.9	0.4751	50.015	0.509
2	23.765	5805.5	137.5	0.6028	49.985	0.487

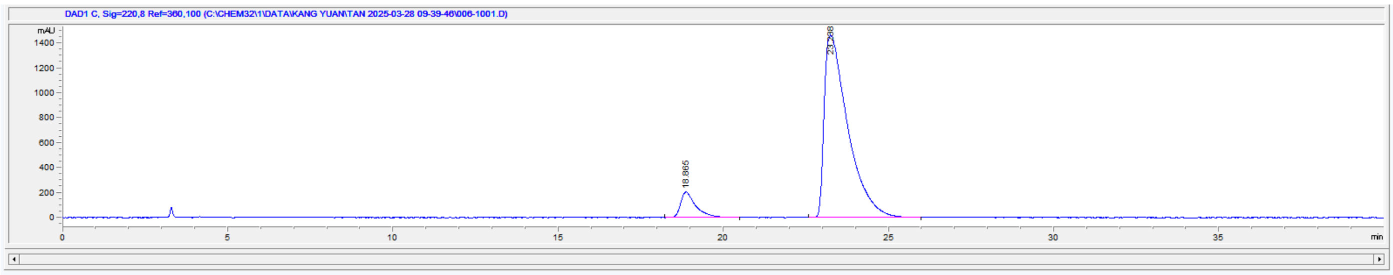

#	Time	Area	Height	Width	Area%	Symmetry
1	18.865	6685.6	206.7	0.4773	8.374	0.498
2	23.238	73153.5	1465.9	0.5982	91.626	0.34

[back to table of contents]

methyl (*R*)-2-(1-(phenylsulfonyl)-1,2,3,4-tetrahydroquinolin-2-yl)acetate (8ae)

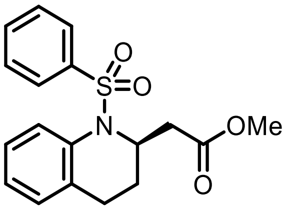

(CHIRALPAK® IA, Hexane/IPA 98/2, 1 mL/min)

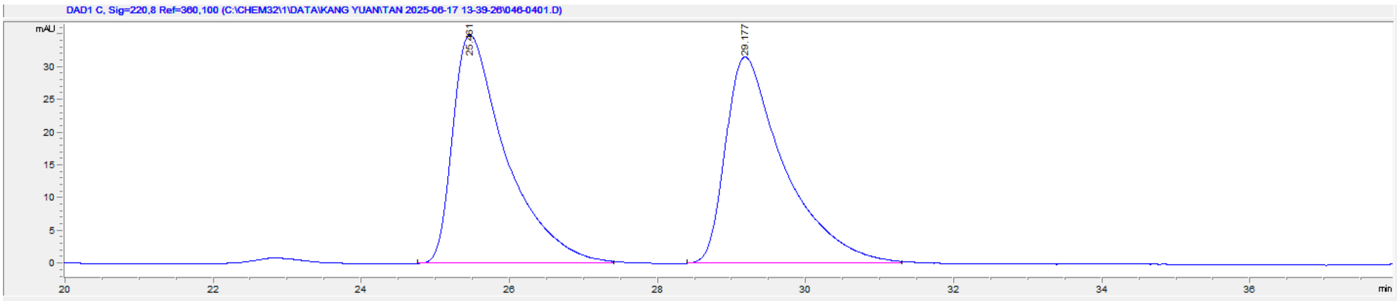

#	Time	Area	Height	Width	Area%	Symmetry
1	25.461	1742	34.8	0.7292	50.114	0.438
2	29.177	1734.1	31.5	0.7946	49.886	0.457

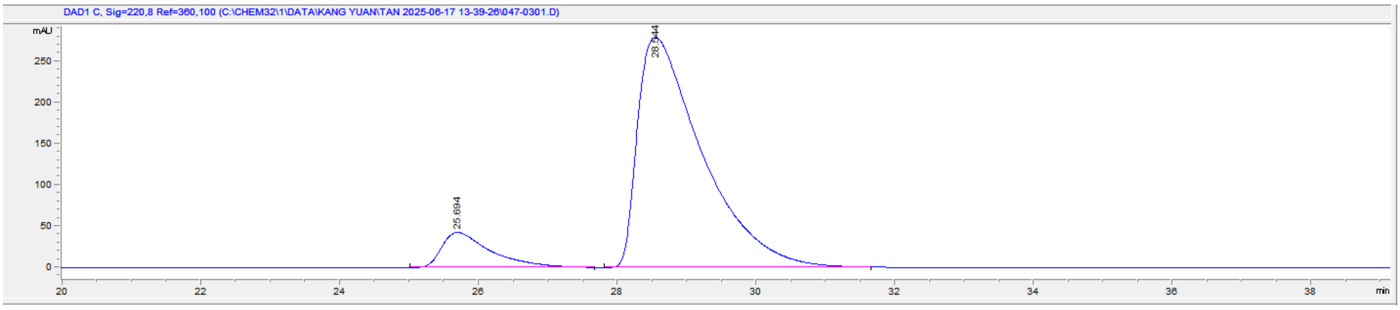

#	Time	Area	Height	Width	Area%	Symmetry
1	25.694	2106.3	42.6	0.727	10.452	0.435
2	28.544	18046.4	279.4	0.9595	89.548	0.346

[back to table of contents]

tert-butyl (*R*)-2-(2-methoxy-2-oxoethyl)pyrrolidine-1-carboxylate (9)

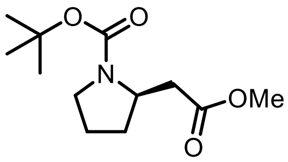

(CHIRALPAK® AD-H, Hexane/IPA 98/2, 1 mL/min)

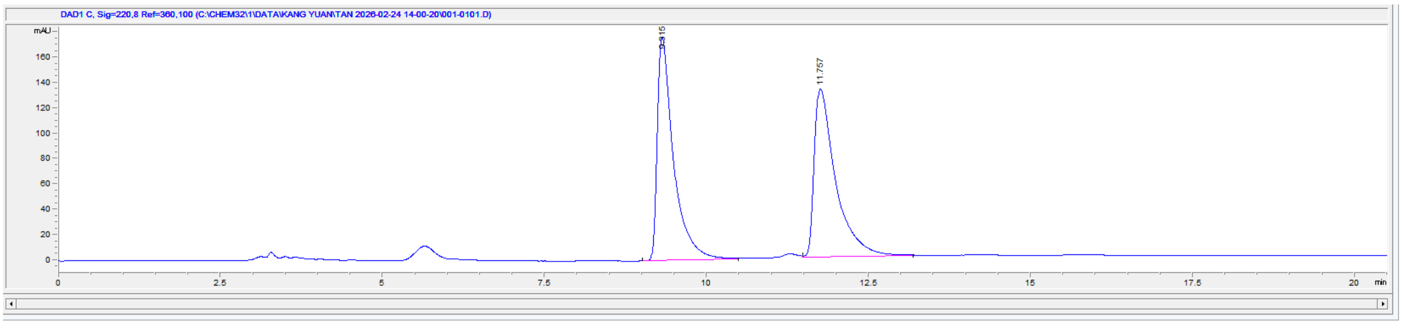

#	Time	Area	Height	Width	Area%	Symmetry
1	9.315	3095.9	176	0.2592	50.221	0.394
2	11.757	3068.7	132.8	0.3375	49.779	0.362

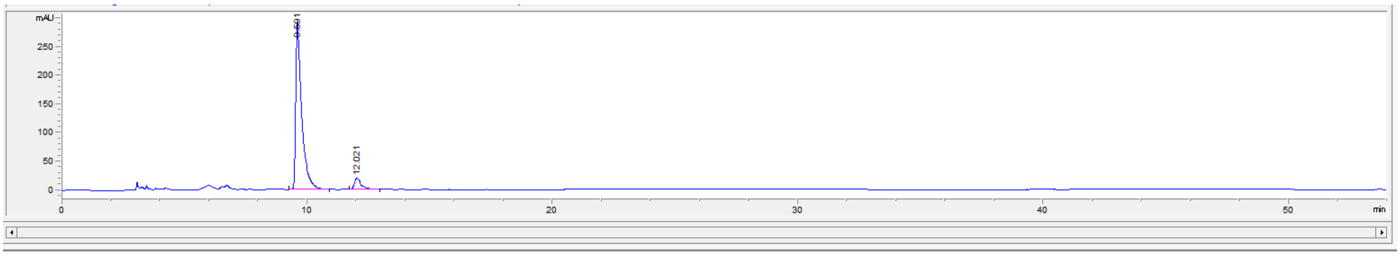

#	Time	Area	Height	Width	Area%	Symmetry
1	9.591	5147.2	293.1	0.2589	93.185	0.379
2	12.021	376.4	19.5	0.2829	6.815	0.543

[back to table of contents]

ethyl 2-(1-(benzylsulfonyl)-5-oxopyrrolidin-2-yl)acetate (10)

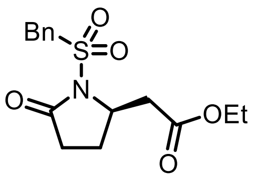

(CHIRALPAK® IA, Hexane/IPA 80/20, 1 mL/min)

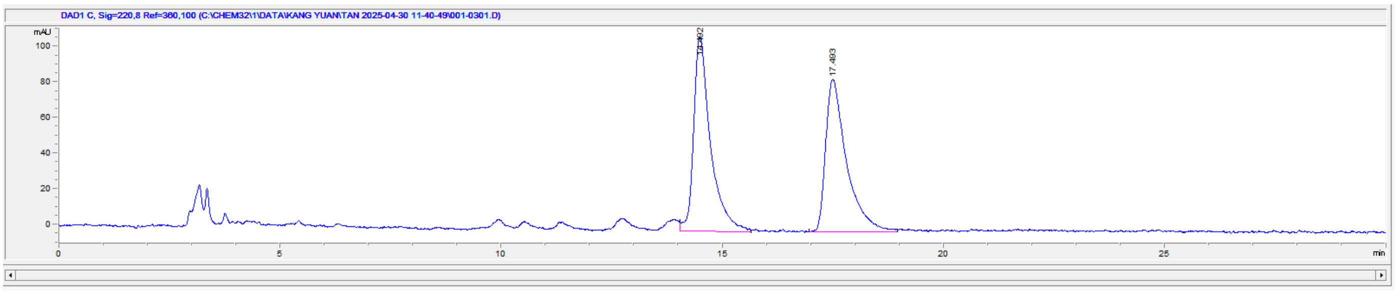

#	Time	Area	Height	Width	Area%	Symmetry
1	14.492	2916.9	109.2	0.3916	50.566	0.554
2	17.493	2851.6	85.6	0.4605	49.434	0.47

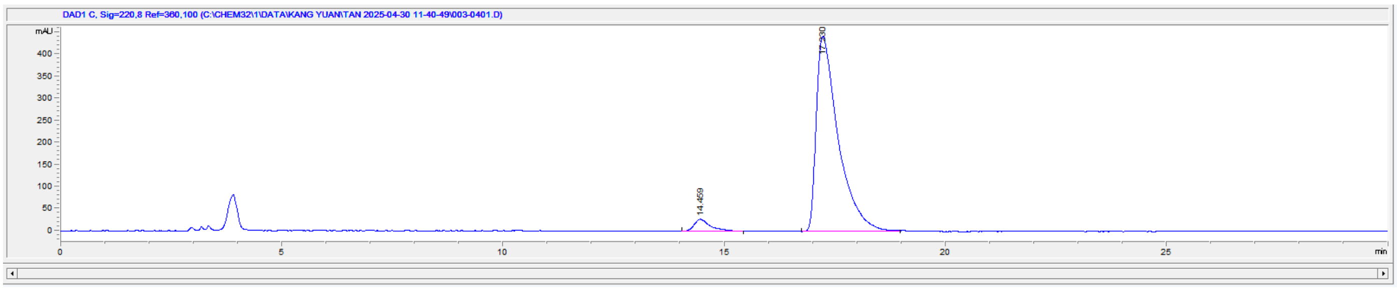

#	Time	Area	Height	Width	Area%	Symmetry
1	14.459	758.3	27.8	0.3746	4.701	0.545
2	17.23	15373.2	443	0.5029	95.299	0.396

[back to table of contents]

(*R*)-2-(2-(benzylsulfonyl)-2-azaspiro[4.5]decan-3-yl)ethan-1-ol (11)

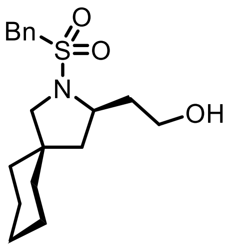

(CHIRALPAK® IC, 1500 psi, 30 °C, CO_2/MeOH 99/1 to 70/30 over 5 mins, then 70/30 to 50/50 over 0.5 min, then hold 50/50 for 2.5 min, then 50/50 to 100/0 over 0.1 min, then hold 100/0 for 0.9 min, 1.5 mL/min)

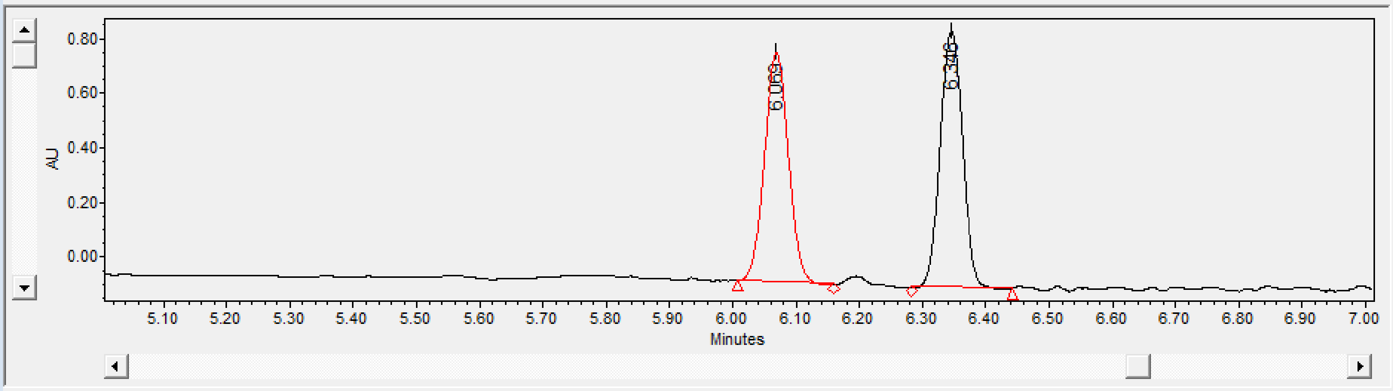

	Name	Retention Time (min)	Purity1 Angle	Purity1 Threshold	PDA/FLR Match1 Spect. Name	PDA/FLR Match1 Angle	PDA/FLR Match1 Threshold	PDA/FLR Match1 Lib. Name	Area (µV*sec)	% Area	Height (µV)	Int Type	Amount	Units	Peak Type
1		6.069							2211295	49.25	841091	bv			Unknown
2		6.346							2278947	50.75	934080	Vb			Unknown

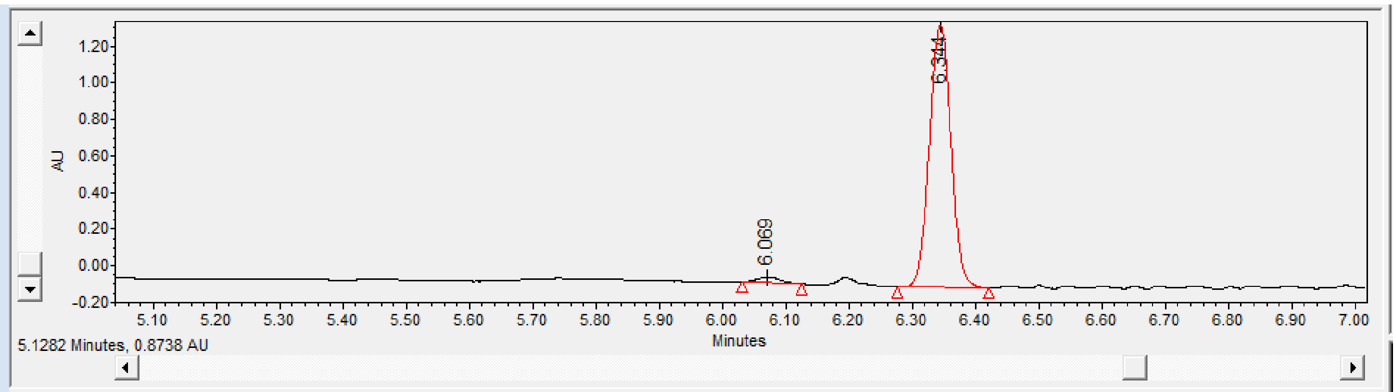

	Name	Retention Time (min)	Purity1 Angle	Purity1 Threshold	PDA/FLR Match1 Spect. Name	PDA/FLR Match1 Angle	PDA/FLR Match1 Threshold	PDA/FLR Match1 Lib. Name	Area (µV*sec)	% Area	Height (µV)	Int Type	Amount	Units	Peak Typ
1		6.069							80150	2.31	31556	bb			Unknown
2		6.344							3385029	97.69	1430730	bb			Unknown

[back to table of contents]

(*R*)-3-(2-azidoethyl)-2-(benzylsulfonyl)-2-azaspiro[4.5]decane (12)

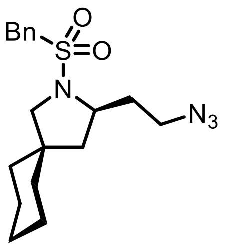

(CHIRALPAK® AS-H, Hexane/IPA 90/10, 1 mL/min)

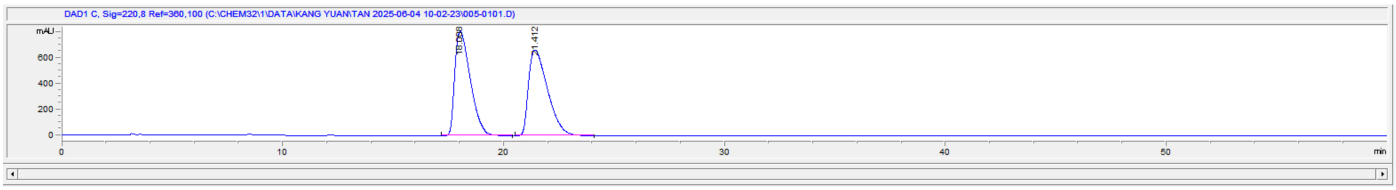

#	Time	Area	Height	Width	Area%	Symmetry
1	18.008	39468.2	807.6	0.7661	49.900	0.498
2	21.412	39626.3	664.6	0.9316	50.100	0.471

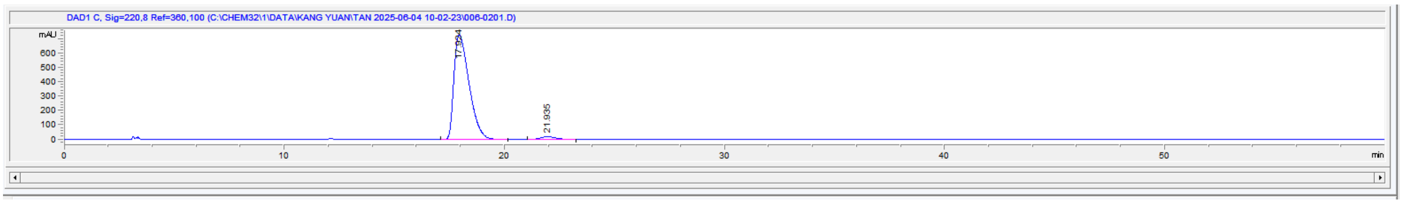

#	Time	Area	Height	Width	Area%	Symmetry
1	17.924	34457	726	0.7417	97.436	0.517
2	21.935	906.6	18.9	0.7447	2.564	0.79

[back to table of contents]

References

[1] J. Cosier, A. M. Glazer, *J. Appl. Crystallogr.* **1986**, *19*, 105–107.
[2] L. Palatinus, G. Chapuis, *J. Appl. Crystallogr.* **2007**, *40*, 786–790.
[3] P. Parois, R. I. Cooper, A. L. Thompson, *Chem. Cent. J.* **2015**, *9*, 30.
[4] R. I. Cooper, A. L. Thompson, D. J. Watkin, *J. Appl. Crystallogr.* **2010**, *43*, 1100–1107.
[5] D. Rozsar, M. Formica, K. Yamazaki, T. A. Hamlin, D. J. Dixon, *J. Am. Chem. Soc.* **2022**, *144*, 1006–1015.
[6] H.-J. Jiang, K. Liu, J. Yu, L. Zhang, L.-Z. Gong, *Angew. Chem. Int. Ed.* **2017**, *56*, 11931–11935.
[7] S. Roy, K. A. Unnikrishnan, A. Chakraborty, R. Kuniyil, I. Chatterjee, *Org. Lett.* **2024**, *26*, 1629–1634.
[8] S.-P. Luo, L.-D. Guo, L.-H. Gao, S. Li, P.-Q. Huang, *Chem. Eur. J.* **2013**, *19*, 87–91.
[9] D. Nath, M. C. Skilbeck, I. Coldham, F. F. Fleming, *Org. Lett.* **2014**, *16*, 62–65.
[10] C. J. Maddocks, K. Ermanis, P. A. Clarke, *Org. Lett.* **2020**, *22*, 8116–8121.
[11] T. Helbing, M. Kirchner, J. Becker, R. Göttlich, *Eur. J. Org. Chem.* **2022**, *2022*, e202200597.
[12] B. J. Casavant, A. S. Hosseini, S. R. Chemler, *Adv. Synth. Catal.* **2014**, *356*, 2697–2702.
[13] W. Kong, P. Feige, T. de Haro, C. Nevado, *Angew. Chem. Int. Ed.* **2013**, *52*, 2469–2473.
[14] J. A. McCauley, C. J. McIntyre, M. T. Rudd, K. T. Nguyen, J. J. Romano, J. W. Butcher, K. F. Gilbert, K. J. Bush, M. K. Holloway, J. Swestock, B.-L. Wan, S. S. Carroll, J. M. DiMuzio, D. J. Graham, S. W. Ludmerer, S.-S. Mao, M. W. Stahlhut, C. M. Fandozzi, N. Trainor, D. B. Olsen, J. P. Vacca, N. J. Liverton, *J. Med. Chem.* **2010**, *53*, 2443–2463.
[15] R. J. Maza, J. Royes, J. J. Carbó, E. Fernández, *Chem. Commun.* **2020**, *56*, 5973–5976.
[16] S. Nicolai, J. Waser, *Org. Lett.* **2011**, *13*, 6324–6327.
[17] X. Wen, M. Gao, Y. Chen, Y. Zhao, W. Qin, L. Hu, *Org. Lett.* **2022**, *24*, 8387–8391.
[18] G. Lemière, V. Gandon, K. Cariou, A. Hours, T. Fukuyama, A.-L. Dhimane, L. Fensterbank, M. Malacria, *J. Am. Chem. Soc.* **2009**, *131*, 2993–3006.
[19] H. Yamamoto, E. Ho, K. Namba, H. Imagawa, M. Nishizawa, *Chem. Eur. J.* **2010**, *16*, 11271–11274.
[20] P. Mondal, L. Thander, S. K. Chattopadhyay, *Tetrahedron Lett.* **2012**, *53*, 1328–1331.
[21] H.-X. Feng, R. Tan, Y.-K. Liu, *Org. Lett.* **2015**, *17*, 3794–3797.
[22] S. G. Davies, A. M. Fletcher, P. M. Roberts, A. D. Smith, *Tetrahedron* **2009**, *65*, 10192–10213.
[23] J. Rein, B. Górski, Y. Cheng, Z. Lei, F. Buono, S. Lin, *J. Am. Chem. Soc.* **2024**, *146*, 31412–31419.
